# Ecological impact of algal-preying protists aplanochytrids (Labyrinthulea) as a source of docosahexaenoic acid for copepods in marine environments

**DOI:** 10.1093/ismeco/ycag178

**Published:** 2026-06-26

**Authors:** Tomi Morimoto, Yoko Hamamoto, Takanori Shono, Mayumi Ueda, Akira Kuwata, Yukiko Taniuchi, Hiroshi Kuroda, Kazuaki Tadokoro, Yuki Tsujimura, Toshiki Miyaoka, Taichi Mogi, Hiroyuki Imai, Ryosuke Nakai, Satoshi Nagai, Tomoko Matsumoto, Jun Kikuchi, Daiske Honda

**Affiliations:** Graduate School of Natural Science, Konan University, Kobe 658-8501, Japan; Graduate School of Natural Science, Konan University, Kobe 658-8501, Japan; Faculty of Science and Engineering, Konan University, Kobe 658-8501, Japan; Research Institute of Environment, Agriculture and Fisheries, Osaka Prefecture, Misaki 599-0311, Japan; Fisheries Resources Institute, Japan Fisheries Research and Education Agency, Shiogama 985-0001, Japan; Fisheries Resources Institute, Japan Fisheries Research and Education Agency, Kushiro 085-0802, Japan; Institute of Low Temperature Science, Hokkaido University, Sapporo 060-0819, Japan; Fisheries Resources Institute, Japan Fisheries Research and Education Agency, Shiogama 985-0001, Japan; Research Institute of Environment, Agriculture and Fisheries, Osaka Prefecture, Misaki 599-0311, Japan; Graduate School of Natural Science, Konan University, Kobe 658-8501, Japan; Faculty of Science and Engineering, Konan University, Kobe 658-8501, Japan; Faculty of Science and Engineering, Konan University, Kobe 658-8501, Japan; Institute for Integrative Neurobiology, Konan University, Kobe 658-8501, Japan; Biomanufacturing Process Research Center, National Institute of Advanced Industrial Science and Technology (AIST), Sapporo 062-8517, Japan; Fisheries Technology Institute, Japan Fisheries Research and Education Agency, Yokohama 236-8648, Japan; RIKEN, Center for Sustainable Resource Science, Yokohama 230-0045, Japan; RIKEN, Center for Sustainable Resource Science, Yokohama 230-0045, Japan; Faculty of Science and Engineering, Konan University, Kobe 658-8501, Japan; Institute for Integrative Neurobiology, Konan University, Kobe 658-8501, Japan

**Keywords:** polyunsaturated fatty acid, *Aplanochytrium*, zooplankton, diatoms, marine ecosystem, metabarcoding

## Abstract

Docosahexaenoic acid (DHA) is an ω-3 polyunsaturated fatty acid with essential physiological functions in marine fish. Therefore, both fish and their prey must acquire DHA through the food web. Diatoms and especially dinoflagellates have traditionally been considered the primary producers of DHA. However, this assumption presents a paradox because, when considering the polyunsaturated fatty acid profiles produced by both groups, they contain substantially more eicosapentaenoic acid (EPA) than DHA, even though marine fishes exhibit nearly equivalent DHA:EPA ratios. In this study, based on biomass surveys conducted in the western North Pacific off northeastern Japan, we suggest that aplanochytrids (Labyrinthulea) are DHA suppliers comparable to dinoflagellates. Their accumulation in algae-rich regions, along with metabarcoding analysis and co-culture experiments, suggests their ecological role as predators in marine environments. Moreover, our estimates based on reported gut content analysis of calanoid copepods suggest that aplanochytrids efficiently transfer DHA to higher trophic levels, potentially surpassing diatoms and dinoflagellates in this role. This study is the first to indicate that aplanochytrids can contribute to DHA production and have the potential to prey on diverse algal groups within the grazing food chain. Therefore, for calanoid copepods, the primary prey of many marine fish species and lacking the ability to synthesize DHA de novo, relying instead on external sources, aplanochytrids represent a major and previously overlooked source of DHA that contributes substantially to its accumulation in fish. Labyrinthuleans are greater DHA contributors than recognized, thus requiring trophic flux studies including labyrinthuleans to clarify ecological significance.

## Graphical abstract

**Graphical abstract:**
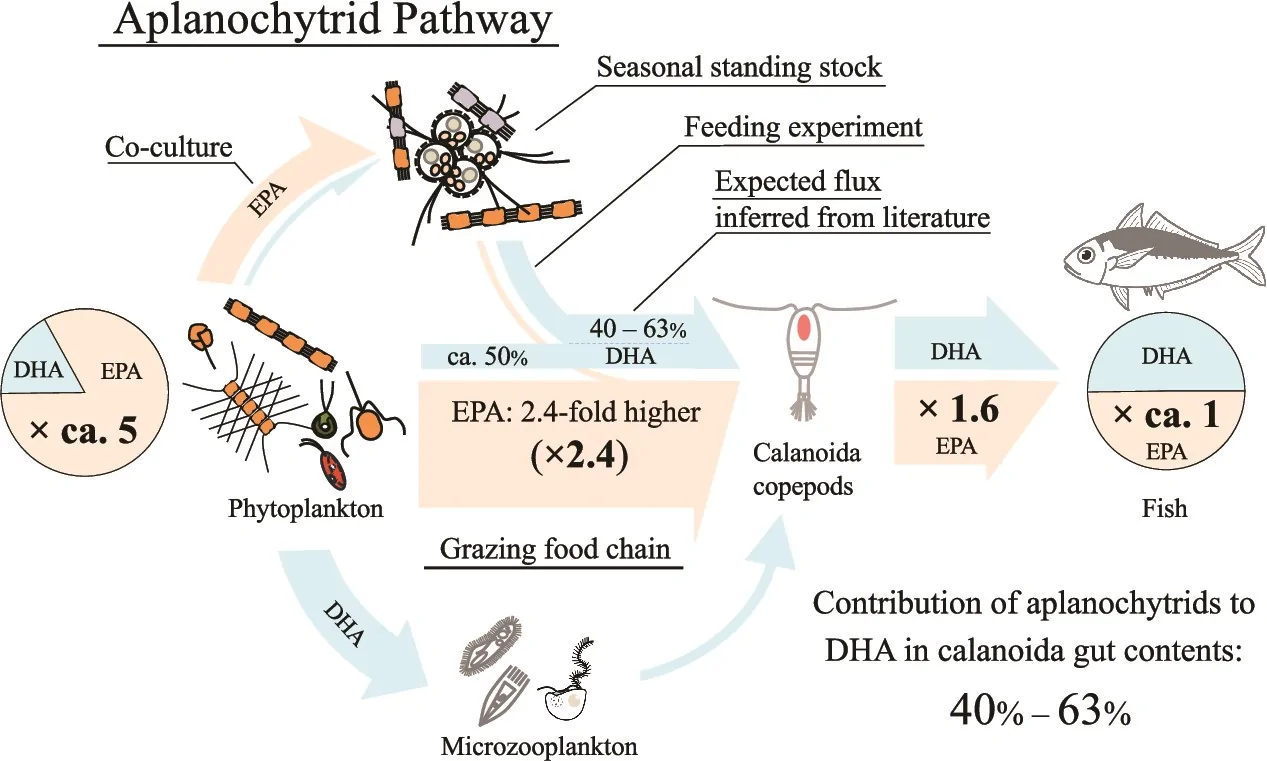

## Introduction

Polyunsaturated fatty acids (PUFAs) are essential nutrients for animals. Among these, docosahexaenoic acid (DHA) is particularly important for physiological functions [1, 2]. Fish contain especially high levels of DHA, which is indispensable for their growth [3, 4]. However, most marine fish species are unable to synthesize DHA sufficiently *de novo* [5, 6]. Calanoid copepods, which are the primary prey of fish, also lack the ability to biosynthesize DHA [7, 8]. Therefore, higher trophic consumers must acquire DHA through the food web [8, 9]. Known DHA-producing organisms include various microalgae and protists, such as diatoms, dinoflagellates, haptophytes, cryptophytes, pinguiophyceans, ichthyosporeans, and labyrinthuleans [10–13]. DHA contents per unit carbon have been documented for some of these groups, e.g. diatoms (4.27 mg/g C), dinoflagellates (32.57 mg/g C), and labyrinthuleans (57.67 mg/g C) [10]. Many of the small DHA producers, including pico- and nano-planktonic unicellular organisms, are thought to transfer a limited amount of material through the food web. This is because, before reaching higher trophic consumers such as calanoid copepods, they are consumed by microzooplankton, including heterotrophic flagellates and ciliates. Due to energy loss at each trophic level, the total amount of material transferred along this pathway is considered low [14–16]. Thus, relatively large and abundant groups, such as diatoms and especially dinoflagellates, are currently recognized as the major DHA suppliers [10, 17, 18].

However, a paradox exists in the DHA/EPA ratios between primary producers, such as diatoms and dinoflagellates, and higher trophic consumers such as fish. On a global scale, diatoms are estimated to have a biomass approximately three-fold greater than that of dinoflagellates [19–21]. In addition, diatoms accumulate approximately seven-fold more eicosapentaenoic acid (EPA) than DHA, while dinoflagellates accumulate approximately four-fold more DHA than EPA [10]. The total DHA + EPA content per carbon biomass is similar between these two groups [10]. Therefore, the composition of PUFAs produced by these two groups indicates that the proportion of EPA is at least twice as high as that of DHA. Indeed, mesozooplankton such as copepods can graze directly on primary producers without passing through microzooplankton. This PUFA supply pathway efficiently transfers DHA. However, in such cases, the dominant producers tend to be large diatoms, which generally have a higher EPA content. In coastal and upwelling areas where large diatoms dominate, the EPA proportion is expected to increase. Nevertheless, the DHA:EPA ratios found in many fish species are approximately 1:1 or even show higher proportions of DHA [10]. To resolve this paradox in the DHA and EPA ratio, considering the possibility of an unrecognized source of DHA or a hidden pathway that converts abundant EPA, mainly supplied by diatoms, into DHA is necessary.

Labyrinthulean protists are heterotrophic unicellular eukaryotes classified within the stramenopiles as a distinct class-level group [22], and they are currently divided into four major groups: labyrinthulids, aplanochytrids, oblongichytrids, and thraustochytrids [23, 24].They are known to accumulate high DHA levels and synthesize other functionally important compounds, such as astaxanthin and squalene, which has led to growing interest in their potential applications [25–30]. From an ecological perspective, labyrinthuleans have been regarded as saprotrophic organisms because they synthesize enzymes capable of degrading lignin and cellulose, and they have frequently been isolated from coastal and estuarine environments rich in detritus [31–36]. For this reason, they have attracted attention as organisms whose biomass can rival or even exceed that of bacteria in certain coastal areas [37–41]. In recent years, metabarcoding analyses have shown that labyrinthuleans are widely distributed in the ocean [42], including polar regions, and cell densities exceeding 10^5^ cells/L have been reported in pelagic waters as well [36, 37, 43]. These findings have led to the view that labyrinthuleans play an important ecological role by decomposing refractory detritus and providing PUFAs to marine food webs. Furthermore, labyrinthuleans have recently been recognized as important contributors to vertical carbon transport within the marine environment [44, 45].

Recent studies have revealed that aplanochytrids (including *Aplanochytrium*), which are a family-level clade within the Class Labyrinthulea, are capable of nutritional intake from living diatoms. This observation suggests that they play a predatory role within the grazing food web [42]. Based on environmental DNA analysis, aplanochytrids have frequently been reported as the dominant labyrinthulean group in surface waters [43, 45, 46]. Furthermore, metabarcoding analyses have indicated that their sequences are more frequently detected in the gut contents of calanoid copepods, which occupy higher trophic levels, than in the seawater from which copepods were collected [47, 48]. Although diatoms are more frequently detected overall in copepod guts, labyrinthuleans are known to contain approximately 10-fold more DHA per unit carbon than diatoms [10]. Therefore, their contribution as DHA suppliers needs to be evaluated based on more than just detection frequency.

In this study, we conducted year-round monitoring around the Oyashio [49], which is known for its high fishery productivity and abundant prey organisms for fish, to investigate the ecological role and impact of aplanochytrids. This region is optimal for our study because organic carbon is supplied predominantly by primary producers, and contributions from continental detritus to the PUFA pool are negligible [50]. Using quantitative PCR with taxon-specific primers and metabarcoding analysis, we investigated the spatial and seasonal variations in aplanochytrid distribution with respect to depth and location. Furthermore, co-culture experiments were conducted to investigate which algal taxa aplanochytrids can consume and to determine whether aplanochytrids can produce DHA when feeding on diatoms. In addition, feeding experiments were performed using an easily reared zooplankton species to examine the transfer of labyrinthuleans-derived DHA to zooplankton. By integrating these approaches, we aimed to provide foundational ecological insights into aplanochytrids and to examine their potential role as DHA suppliers in marine environments.

## Materials and methods

### Sampling sites and collection

Sampling was conducted at six stations along the ~600 km survey transect operated by the Japan Fisheries Research and Education Agency, spanning from 42° 50′N, 144° 50′E to 38° 00′N, 147° 15′E (Fig. 1). The five research cruises were conducted from July 2021 to May 2022 aboard the research vessels “*Hokko-maru*” and “*Wakataka-maru*”. Environmental parameters were measured using an SBE 911plus CTD system (Sea-Bird Scientific, Bellevue, WA, USA) during water sampling. A 1000 ml aliquot of collected seawater was filtered using an Isopore™ disk polycarbonate membrane (pore size: 0.8 μm, diameter: 47 mm; Merck Millipore, Darmstadt, Germany). The filters were stored at −80°C until further processing.

**Figure 1:**
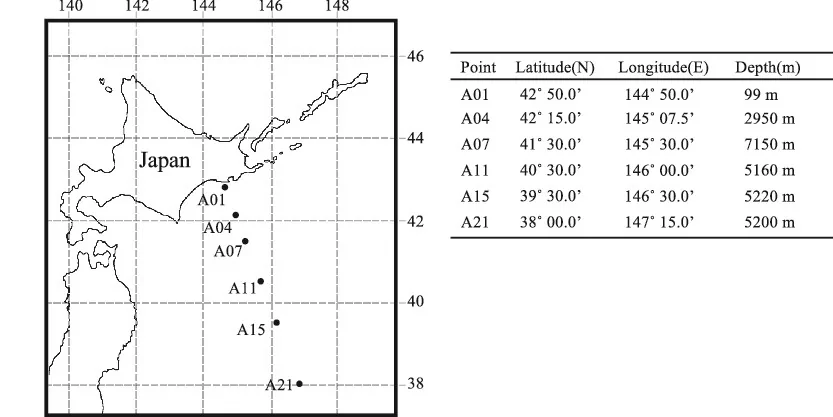
Sampling locations in the study area in the western North Pacific off northeastern Japan. Stations sampled between July 2021 and May 2022 are shown. Cruises conducted were WK2107 and HK2110 (2021) and HK2201, WK2203, and HK2205 (2022) aboard R/V *Wakataka-maru* (WK) and R/V *Hokko-maru* (HK). Water was collected at five depths: surface, above the chlorophyll maximum, the euphotic-zone lower limit (200 m), 1000 m, and 3000 m. Sampling at station A15 occurred only in October 2021 during a phytoplankton bloom associated with a branch of the Oyashio Current.

### Hydrographic classification of the study area

To classify the water masses observed along the A Line, we applied the water system classification method of Kusaka *et al.* [51] for waters off the southeastern Pacific coast of Hokkaido, in which water masses are defined based on specific ranges of temperature, salinity, and density. In this method, water masses are categorized into six water systems: Coastal Oyashio Water (COW), Surface Coastal Oyashio Water (S-COW), Oyashio Water (OW), Modified Soya Warm Current Water (M-SWCW), Kuroshio Water (KW), and Cold Low (CL). Samples with missing values in temperature, salinity, or density were treated as not determined (ND).

It should be noted that M-SWCW can appear even in offshore regions beyond the shelf break due to mixing of KW with OW, S-COW, and COW, in addition to its typical presence of M-SWCW trapped against the coast during summer–autumn [51]. This limitation arises because the water-system classification does not explicitly account for water mass mixing, which is a known weakness of this approach [52]. Therefore, offshore occurrences of M-SWCW should be interpreted as subarctic waters influenced by the Kuroshio. In addition, most waters below 200 m depth were classified as CL, indicating that this classification method has limited ability to resolve spatial variations in deeper water masses. Accordingly, in this study, we applied the water system classification only to waters in the surface layer shallower than 200 m. Depths of 200–800 m are hereafter referred to as the “intermediate layer,” and depths ≥1000 m as the “deep layer.”

### Aplanochytrid-specific primer design

A PCR-specific primer set was designed to selectively amplify a particular region of the 18S rRNA gene in aplanochytrids and not in other taxa. The primer and probe design was based on nucleotide sequences deposited in GenBank (NCBI; https://www.ncbi.nlm.nih.gov/genbank/) and sequences obtained from 11 cultured strains (Tables S1 and S2; [53–55]). A TaqMan probe and primers used for quantitative PCR (qPCR) targeting environmental aplanochytrids were validated using Primer Express Software Version 3.0 (Applied Biosystems, Foster City, CA, USA).

### Evaluation of primer specificity

To evaluate primer specificity, PCR was conducted using extracted DNA from multiple cultured aplanochytrid strains, nine strains of closely related labyrinthuleans, and two algal strains as outgroups (Table S1). Ex Taq (TAKARA Bio Inc., Shiga, Japan) was used as the PCR reagent, and reactions were performed using the aplanochytrid-specific primer set at a final concentration of 200 nM. The PCR protocol and conditions are detailed in Table S3. PCR was conducted using a Veriti Thermal Cycler (Applied Biosystems), and the presence of amplicons was confirmed using agarose gel electrophoresis.

### Estimation of 18S rDNA copy number per cell

To convert the copy number quantified using qPCR into cell numbers, the 18S rDNA copy number per cell of aplanochytrids was estimated. The qPCR reagent used was Power SYBR® Green Master Mix (Thermo Fisher Scientific, Waltham, MA, USA), and the reaction mixture was prepared with the aplanochytrid-specific primer set at a final concentration of 250 nM. The qPCR instrument used was the StepOnePlus Real-Time PCR System (Thermo Fisher Scientific).

A total of nine *Aplanochytrium* strains were used, and stationary-phase cells cultured in 1/10 SeaAct Medium or d-GPY medium were employed (Table S4). Aplanochytrid cell count was determined using a Fuchs–Rosenthal hemocytometer (Hausser Scientific, Horsham, PA, USA), and DNA was extracted from 100 cells of each strain for copy number quantification across nine strains (Table S1). To correct for DNA extraction efficiency, two types of DNA extracts were prepared [56]. A fixed amount of plasmid DNA (pGEM-3Z Vector; Promega Corporation, Madison, WI, USA) was added before and after extraction. The difference in the copy number of the pGEM-3Z Vector between pre- and post-extraction additions was used to determine DNA recovery efficiency, which was then applied to correct the copy number of aplanochytrids. DNA extraction was performed using a DNeasy PowerSoil Kit (Qiagen, Hilden, Germany).

### Quantitative PCR assay

For the biomass survey, a specific primer targeting the 18S rDNA gene of aplanochytrids was used (Table S2), and a total of 105 environmental samples were measured in triplicate (*n* = 3). Based on the agarose gel electrophoresis of PCR products using genomic DNA from labyrinthuleans, including aplanochytrids and outgroup taxa, amplification was confirmed only for the target group, aplanochytrids. Therefore, it was confirmed that the primers possessed sufficient specificity for selectively detecting and quantifying aplanochytrids from environmental samples (Fig. S1; Supplemental Data 1). Each TaqMan probe was synthesized by Thermo Fisher Scientific. In this study, DNA was extracted from the biological material collected on filters from environmental samples, following the same procedure as that used for estimating 18S rDNA copy number. However, two modifications were made to the procedure. First, DNA extraction was conducted using a QuickGene DNA Tissue Kit S (Kurabo Industries Ltd., Osaka, Japan) according to the manufacturer’s instructions. Second, to evaluate DNA recovery efficiency, a 200-bp artificial DNA fragment (Thermo Fisher Scientific) was added before and after extraction instead of plasmid DNA (Table S5).

TaqMan™ Environmental Master Mix 2.0 (Applied Biosystems) was used as the qPCR reagent. The reaction mixture was prepared using the specific primer set (final concentration: 900 nM) and a TaqMan probe (final concentration: 500 nM). The PCR protocol is described in Table S3. To determine DNA recovery efficiency and aplanochytrid abundance, calibration curves were generated using a six-point serial dilution ranging from 10^1^ to 10^6^ copies (Supplemental Data 2). Artificial DNA fragments were used as the template DNA for generating both standard curves (Table S5). The abundance of aplanochytrids in seawater was calculated by converting the qPCR-derived 18S rDNA copy numbers using the measured copy number per cell.

### Calculation of cell abundance and carbon biomass

Cell abundance of aplanochytrids (cells/L) was estimated from qPCR-derived 18S rRNA gene copy numbers. Conversion from copy number to cell abundance was performed using minimum and maximum values of 18S rRNA gene copies per aplanochytrid cell determined in this study. Cell abundance calculated from these minimum and maximum copy numbers were averaged, and this mean value was used as “the representative cell abundance”.

Taxonomic studies have reported cell sizes of aplanochytrids as small as 5 μm; however, this likely reflects the use of newly released aplanospores, which represent a unicellular and uninucleate stage, in those studies [57–59]. On the contrary, vegetative cells have been reported to reach ~10 μm or more [60, 61]; our observations of diatom-feeding cells also confirmed comparable sizes (Fig. S2). Accordingly, carbon biomass was estimated from the calculated cell abundance using a reported carbon content of 165 pg C per cell for labyrinthuleans with a cell diameter of ~10 μm [62].

### Metabarcoding analysis

Metabarcoding analysis was conducted in two formats. The universal primers were used to examine the relationships between labyrinthuleans and other eukaryotic taxa, whereas the labyrinthulean-specific primers were used to resolve the detailed community structure of labyrinthuleans. The primers used are listed in Table S2. Template DNA was the same as that extracted for the qPCR assays. PCR amplification was conducted using KOD Plus Ver. 2.0 polymerase (TOYOBO Co., Ltd., Osaka, Japan). The PCR protocol is provided in Table S3. Next-generation sequencing was outsourced to Bioengineering Lab. Co., Ltd. (Kanagawa, Japan), and sequence analysis was performed using QIIME2 with BLAST-based taxonomic assignment.

### Co-culture of aplanochytrids with various microalgal strains

Microalgal strains were obtained from the National Institute for Environmental Studies (NIES) (Table S1) and cultured for 2 weeks under conditions recommended by NIES. After centrifugation and washing, microalgal suspensions were prepared. The aplanochytrid strain SEK717, known to graze on diatoms [42], was cultured in 1/10 SeaAct or d-GPY medium for 1 week (Table S4). After washing with artificial seawater (Marine Art SF-1, OSAKA YAKKEN Co., Ltd., Osaka, Japan), co-cultures with microalgae were established.

Continuous microscopic observations were conducted using a glass chamber with a differential interference contrast (DIC) microscope (Axiovert 200 DIC; Carl Zeiss AG, Oberkochen, Germany) equipped with an MCI170 camera (Leica Microsystems, Wetzlar, Germany).

Co-culture samples with *Skeletonema* (NIES-324) were collected on Days 0, 1, 3, and 6 to monitor temporal changes in DHA and EPA during the co-culture. Cell densities of *Skeletonema* and aplanochytrids were quantified using a Fuchs–Rosenthal hemocytometer. Cultured cells were harvested and dried at 105°C for 3 h [63]. The dried cells were then mixed with chloroform containing C19:0 fatty acid as an internal standard and 10% hydrochloric acid in methanol [64], followed by heating at 80°C for 2 h. After cooling to room temperature, n-hexane was added, and distilled water was subsequently added. The mixture was gently inverted, centrifuged, and the oil phase was collected for gas chromatographic analysis.

### Feeding experiment using *Artemia*

To verify the transfer of DHA derived from labyrinthuleans to zooplankton, *Artemia* was used as a crustacean zooplankton. Although calanoid copepods would be the most relevant organisms for this purpose, they are difficult to maintain under laboratory conditions. *Artemia*, which is easy to rear and handle in the laboratory, was therefore used (Spectrum Brands Japan, Inc., Tokyo, Japan). The labyrinthulean strain *Aurantiochytrium limacinum* ATCC-MYA-1381 was used as food (Table S1), because aplanochytrids exhibited limited growth on simple organic media and were therefore unsuitable for this experiment. Artificial seawater was prepared using Marine Art SF-1 (OSAKA YAKKEN Co., Ltd.). *Artemia* were hatched by incubating 0.1 g of cysts in 500 ml of artificial seawater under continuous aeration. Individuals that hatched within 24 h were collected, starved for 120 h, and used for experiments at 6 days post-incubation, a period within which *Artemia* can survive under starvation conditions. *Aurantiochytrium* cells at the stationary phase, cultured in d-GPY medium at 25°C with shaking for ~7 days, were used.

Next, 3 ml of the *Aurantiochytrium* culture were added to 47 ml of Marine Art artificial seawater, after which 100 *Artemia* individuals were introduced (*n* = 3). The mixture was then incubated for 2 h. Subsequently, prior to fatty acid analysis, to restrict the analysis to fatty acids derived from *Artemia* body tissues, the recovered *Artemia* were transferred into 5 ml of artificial seawater containing 1:1000 diluted fluorescent beads (4.5 μm in diameter; Polysciences, Inc., Warrington, PA, USA) and incubated for 2 h to allow ingestion of the beads. Subsequently, the *Artemia* were collected. The prepared samples consisted of: (i) *Artemia* that had ingested *Aurantiochytrium* and were subsequently filled with fluorescent beads, (ii) *Artemia* that were unfed, (iii) *Artemia* that had ingested only fluorescent beads, and (iv) the *Aurantiochytrium* culture. Each of these samples was analyzed using gas chromatography with an internal standard solution containing C19:0 fatty acid, in the same method as in the analysis of the two-membered culture with diatoms.

## Results

### Environmental data in the North Pacific

Observations were conducted on five occasions across 1 year in the western North Pacific off northeastern Japan, specifically in July, October, January, March, and May. The surface water temperature ranged approximately from 0°C to 22°C. Salinity across all stations ranged from approximately 32.5 to 34.5. The results of the water mass classification for each sample are shown in Supplemental Data 2.

### Distribution of aplanochytrids in the in the study area

The cell abundance of aplanochytrids in the study area was estimated from qPCR copy numbers. As the copy number per cell varies among strains (Table S6), each value was expressed as the average of the minimum and maximum estimates, and the mean of three replicates (*n* = 3) is shown in Fig. 2, which visualizes the data presented in Supplemental Data 2. The measured copy number per cell ranged from 150 ± 50 in strain SEK753 to 980 ± 240 in strain SEK603, corresponding to an approximately seven-fold difference among strains. The highest cell abundance of aplanochytrids was ~47 000 cells/L at station A07 in July 2021, where the water mass was classified as S-COW. The lowest abundance was 0 cells/L in the deep layer. Aplanochytrids were consistently present in the intermediate layer or shallower at all stations, with peak abundances most often observed in the surface layer (Fig. S3). Metabarcoding analysis indicated that water temperature had a stronger influence on aplanochytrid read abundance than chlorophyll (Fig. S4). Although aplanochytrid cell abundance showed a positive correlation with chlorophyll concentration (Spearman’s ρ = 0.66, *P* < .001; Fig. S1), this relationship was weak and showed considerable variability among stations (Fig. S4). In most cases, their cell abundance sharply decreased below 200 m compared with that in the surface layer. Across seasons, the average cell abundance in the deep layer was ~11 cells/L, indicating low presence. The average cell abundance in the intermediate layer and shallower across all stations and all seasons was ~2000 ± 6776.61 cells/L, based on 66 samples, reflecting variability among samples.

**Figure 2:**
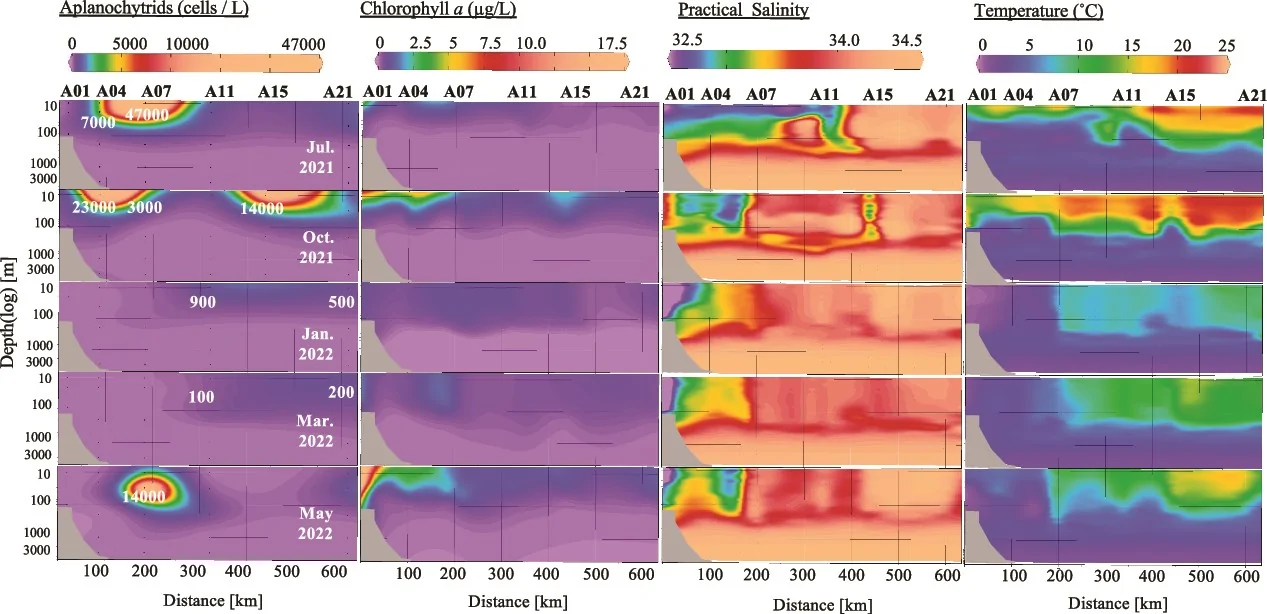
Seasonal variation in the cell abundance of aplanochytrids and environmental parameters observed in the western North Pacific off northeastern Japan. Each panel presents the cell abundance of aplanochytrids and associated environmental data. Gray areas indicate the seafloor. Depth is plotted on a logarithmic scale. Approximate values of observed aplanochytrid cell abundance are highlighted in white text within the plots. Cell abundance (cells/L) was estimated based on the copy number detected using qPCR.

In many cases, aplanochytrids showed particularly high cell abundance in July 2021 (summer) and October 2021 (autumn) in water masses that were not classified as KW, which are generally considered oligotrophic (Fig. 2; Supplemental Data 2). An exception was observed at station A07 in May 2022, where a marked peak occurred despite the water mass being classified as KW. Furthermore, although classified as COW or S-COW, no marked peak was observed at the coastal station A01.

### Biomass of aplanochytrids in the study area

Based on the previously reported carbon content per cell of labyrinthuleans [62], the biomass of aplanochytrids in the study area was estimated to be 0.36 ± 1.23 mg C/m^3^ as the overall average within the intermediate layer, which was the upper 200 m of the water column. Similarly, the average biomass estimated from cell abundance was ~1.88 ± 2.37 mg C/m^3^ based on nine samples from stations A04 and A07 in summer, autumn, and spring, most of which were influenced by subarctic water. These samples were collected from the surface layer above the chlorophyll maximum, which was generally located at depths shallower than ~50 m.

### Metabarcoding analyses

Among the metabarcoding analyses conducted in this study, the results targeting all eukaryotes revealed characteristic patterns for each water mass when focusing on the relative proportions of DHA producers and labyrinthuleans (Fig. 3). First, the proportion of labyrinthuleans, measured as the relative abundance of sequencing reads, tended to be approximately two-fold higher in surface waters of the non-Kuroshio region than in those of the Kuroshio region during spring, summer, and autumn (Fig. 3). Such a difference was observed for diatoms or dinoflagellates. haptophytes, which are DHA producers with unknown biomass levels, showed a vertical distribution pattern similar to that of diatoms and dinoflagellates; however, in the surface waters of the non-Kuroshio region during spring, summer, and autumn, their average read proportion was only approximately 1.7-fold higher than that of labyrinthuleans. In contrast, for cryptophytes, the average read proportion was only 0.27% in the surface waters of the non-Kuroshio region during spring, summer, and autumn, and even in these waters, their proportion was approximately 0.7-fold that of labyrinthuleans. In the 1000–3000 m depth range, where sunlight is limited, the average read proportion of phytoplankton decreased substantially.

**Figure 3:**
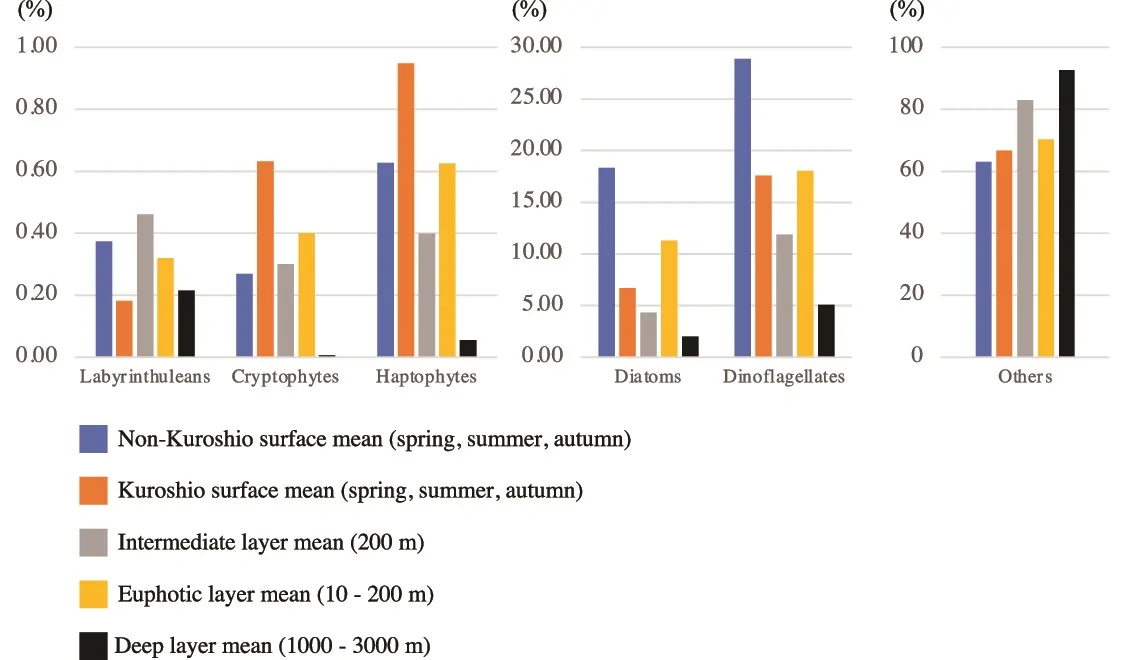
Regional variation in the relative read abundance of DHA-supplying groups. Each panel shows the differences in the relative read abundance of each group across water masses based on 18S rRNA gene metabarcoding analysis, with values calculated relative to the total eukaryotic reads. non-Kuroshio surface mean: average of samples collected at 10 m depth and at the chlorophyll maximum layer during spring, summer, and autumn in regions other than those classified as Kuroshio water. The chlorophyll maximum layer in the non-Kuroshio water masses was located shallower than 30 m. Kuroshio surface mean: average of samples collected at 10 m depth and at the chlorophyll maximum layer during spring, summer, and autumn in regions classified as Kuroshio water. At these stations, the chlorophyll maximum layer was located shallower than 50 m. Intermediate layer mean: average of samples collected at 200 m depth across all stations, corresponding to the photosynthetic limit layer. Euphotic layer mean: average of samples collected from the 10 to 200 m depth across all stations. Deep layer mean: average of samples collected at 1000 m and 3000 m depth across all stations. Although not statistically significant, the read abundance of labyrinthuleans tended to be higher in the surface waters of the non-Kuroshio region.

Environmental sequences obtained from metabarcoding analysis using labyrinthulean-specific primers were classified by performing BLAST searches against the dataset of 1 181 identified labyrinthulean species [65]. As a result, 1 129 680 labyrinthulean reads were obtained, which were assigned to 19 distinct lineages. The most frequently detected lineages included Aplanochytriidae (333 252 reads, 25.2%), Oblongichytriidae (228 214 reads, 16.7%), LAB14 (299 503 reads, 22.6%), and Thraustochytriidae (111 012 reads, 9.2%). The number of unclassified labyrinthuleans reads was 23 516, accounting for 2.1% of the total. A heatmap was generated based on this taxonomic information (Fig. 4). Consistent with these results, a non-metric multidimensional scaling analysis combined with environmental vector fitting (envfit) showed that the relative abundance of Aplanochytriidae tended to be higher in surface waters (Fig. S4).

**Figure 4:**
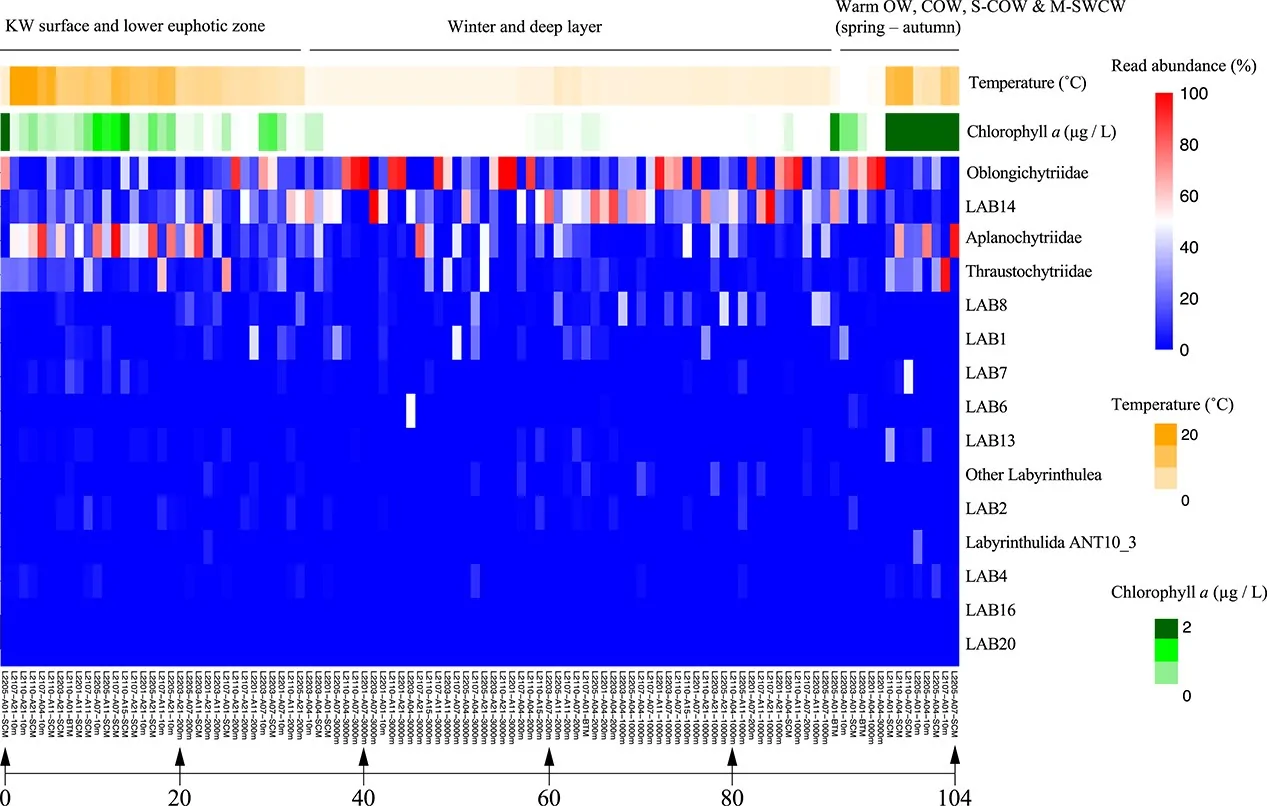
Heatmap based on 18S rRNA gene metabarcoding analysis using labyrinthulean-specific primers. Clustering was performed based on water temperature and chlorophyll concentration. Relative read abundances are shown as proportions of the total labyrinthulean reads. The relative read abundance of aplanochytrids was dominant in surface samples where chlorophyll concentrations, which serve as an indicator of the presence of algal prey, were relatively high. In samples from below the euphotic zone (approximately depth − 200 m) to the upper mesopelagic layer, Oblongichytriidae and the unclassified labyrinthuleans clade LAB14 were dominant. The heatmap was generated using R (version 4.5.0; R Foundation for Statistical Computing, Vienna, Austria). Labels on the heatmap provide a rough indication of sample clustering patterns and do not necessarily reflect water mass classification.

### Assessment of algal predation by aplanochytrids

The feeding behavior of aplanochytrids was evaluated in three stages: first, the formation of aggregates around algal cells; second, the contraction of algal cell contents or cell color fading; and finally, an increase in aplanochytrid cell abundance (Table S7). The formation of aggregates with algal cells is an important behavior, because it reflects the function of the ectoplasmic nets of aplanochytrid cells as the feeding apparatus, which adhere and attract prey cells, forming aggregates [42]. On the contrary, algal cells that the nets do not even adhere to are not targeted for prey (Figs S6 and S7).

#### Green algae

Aggregate formation with *Aplanochytrium* was observed in co-cultures with the genera *Pyramimonas* and *Tetraselmis*. In particular, with *Pyramimonas*, algal cell content contraction occurred in a manner similar to that observed in *Skeletonema* [42] (Figs S2 and S8). In contrast, in the genera *Ostreococcus* and *Mantoniella*, both of which have relatively small cell sizes, no aggregate formation was observed (Fig. S6).

#### Haptophytes

Aggregate formation was observed between *Aplanochytrium* and *Calyptosphaera*. However, no contraction or other changes in algal cells were observed in either of the two haptophyte strains used in this study (Fig. S9).

#### Dinoflagellates

Aggregates larger than 100 μm were observed in co-culture with a *Prorocentrum* strain. Although direct predation was not confirmed, after co-culture, cell content contraction and color changes were noted in the *Prorocentrum* cells (Fig. S10). Similarly, in *Heterocapsa* strains, aggregate formation and both content contraction and color change were observed. In both cases, an increase in the cell abundance of aplanochytrids was detected (Fig. S11).

#### Chlorarachniophytes

Aggregate formation with *Aplanochytrium* was observed, but no visible changes in *Chlorarachnion* cells or in the cell abundance of *Aplanochytrium* after co-culture were detected (Fig. S12).

#### Cyanobacteria

As in the case of small-celled green algae (*Ostreococcus* and *Mantoniella*), no aggregate formation was observed in the small-celled *Cyanobium* strains, and no specific behavioral changes in *Aplanochytrium* were detected (Fig. S7).

### Docosahexaenoic acid production by aplanochytrids

Changes in cell abundance during co-culture (Fig. S13A) and DHA and EPA levels (Fig. S13B) were determined. The ratio of DHA to EPA in aplanochytrids was 9:1. In contrast, in *Skeletonema*, the ratio was 1:9 (Fig. S13B). Examination of the temporal dynamics in cell abundance and fatty acid composition during co-culture showed that diatom cell abundance decreased significantly on Day 3, while that of aplanochytrids increased over time (Fig. S13A). Additionally, on Day 3, when the diatom cell abundance had markedly declined, the proportion of DHA in the culture medium peaked, while that of EPA reached a minimum (Fig. S13B).

### Docosahexaenoic acid transfer experiment using *Artemia*

The EPA:DHA ratio of *Aurantiochytrium* was 1:99. *Artemia* individuals that were unfed or fed only with fluorescent beads contained EPA but no detectable DHA; thus, no capacity for DHA biosynthesis was detected in the *Artemia* used in this study. In contrast, DHA was detected in *Artemia* fed with *Aurantiochytrium*, and an increase in EPA content was also observed (Fig. S13C). This increase shows DHA transfer from *Aurantiochytrium*, a labyrinthulean, to zooplankton.

## Discussion

### Comparison of docosahexaenoic acid supply between aplanochytrids and primary producers

Aplanochytrids, which were confirmed to ingest nutrients from living diatoms through co-culture experiments [42], were further shown in the present study to prey on a broad range of algal taxa, including diatoms, dinoflagellates, and green algae. At least when feeding on diatoms, aplanochytrids were able to produce DHA, and DHA produced by labyrinthuleans was shown to be transferred to zooplankton, *Artemia*. In addition, their annually surveyed distribution patterns of aplanochytrids were generally consistent with those of algal cells and closely mirrored those of micro- and meso-zooplankton, which previous studies have reported to be more abundant in the upper part of the euphotic zone, as shown in Fig. 2 of [66], which tend to be more abundant in the upper part of the euphotic zone. Additionally, within the study area, they were more commonly found in areas with relatively higher water temperatures (Fig. S4) and higher chlorophyll concentrations (Fig. S5). The results of this study do not contradict interpreting aplanochytrids as predators in marine environments that contribute to DHA production within marine grazing food webs.

To estimate the ecological impact of aplanochytrids, we compared their distribution with previously reported biomass data of phytoplankton in the same and adjacent waters, where seasonal fluctuations associated with the inflow of the Oyashio Current have been observed [66]. According to the results of the metabarcoding analysis of surface waters in the non-Kuroshio region, during spring, summer, and autumn, cryptophytes accounted for only an average of 0.27% of the total sequence reads and were thus considered negligible in the present estimation (Fig. 3). Haptophytes accounted for 0.63% of the reads, approximately 1.63-fold greater than the proportion of labyrinthuleans. However, considering that the DHA content per unit carbon in haptophytes was less than half that in labyrinthuleans, and that haptophytes and cryptophytes are not directly consumed by copepods [47], these groups were excluded from the present comparison of potential DHA suppliers to higher trophic levels [10, 47, 48].

Accordingly, a comparison of DHA standing stocks was conducted among aplanochytrids, diatoms, and dinoflagellates (Table 1; [67]). We calculated the DHA standing stock of each group based on the reported biomass values of diatoms and dinoflagellates and their respective DHA contents per unit carbon. Aplanochytrids accounted for 8.1% of the total biomass of microzooplankton in autumn, 6.0% in summer, and 1.1% in spring. While aplanochytrids are considered minor constituents in the marine ecosystem as a whole, these values represent a remarkably high proportion for a single phylogenetic group within the highly diverse microzooplankton community. When compared on a seasonal basis, aplanochytrids accounted for 87.6% of the total DHA standing stock among the three groups in summer, 19.6% in autumn, and 2.2% in spring. Based on the total values for the three seasons, the amount of DHA contained in aplanochytrids was comparable to that of dinoflagellates and equivalent to 20.7% that of diatoms. DHA is a key component for the reproduction of calanoid copepods [68]. Thus, the DHA supply derived from aplanochytrids observed in summer can support the maintenance of subsequent copepod generations for species such as *Neocalanus plumchris* [69].

**Table 1:** Comparison of aplanochytrid and plankton biomass at each trophic level.

			Biomass [mgC/m^3^]	DHA [mg/m^3^]	Reference
			(% of total microzooplankton biomass)	(Relative DHA content (%) among the three groups)	
**Summer**	Mesozooplankton		38	-	[67]
	Microzooplankton+HNF		49.2	-	[67]
	Micro-phytoplankton				[67]
		Diatoms	3.8	**0.02 (5.2%)**	
		Dinoflagellates	0.8	**0.02 (7.1%)**	
	Pico+Nano-phytoplankton		58.1	-	[67]
	Aplanochytrids		**4.39 (6.0%)**	**0.26 (87.6%)**	This study
**Autumn**	Mesozooplankton		9.7	-	[67]
	Microzooplankton+HNF		27	-	[67]
	Micro-phytoplankton				[67]
		Diatoms	104.7	**0.42 (63.2%)**	
		Dinoflagellates	4.4	**0.11 (17.3%)**	
	Pico+Nano-phytoplankton		29.4	-	[67]
	Aplanochytrids		**2.16 (8.1%)**	**0.13 (19.6%)**	This study
**Spring**	Mesozooplankton		124.3	-	[67]
	Microzooplankton+HNF		63.2	-	[67]
	Micro-phytoplankton				[67]
		Diatoms	402.5	**1.61 (86.4%)**	
		Dinoflagellates	8.2	**0.21 (11.4%)**	
	Pico+Nano-phytoplankton		7.3	-	[67]
	Aplanochytrids		**0.70 (1.1%)**	**0.04 (2.2%)**	This study
				**Diatoms: 2.04 (72.6%)**	
				**Dinoflagellate: 0.35 (12.4%)**	
				**Aplanochytrids:0.42 (15.0%)**	

Carbon content (mg C/m^3^) in the grazing food chain in the western North Pacific off northeastern Japan. The carbon content of aplanochytrids was calculated as the average value observed at stations A04 and A07 in this study. The carbon contents of diatoms and dinoflagellates were approximately estimated based on Fig. 3 [67], which shows variations in phytoplankton biomass indicative of seasonal blooms, comparable to those observed in this study. The biomass of dinoflagellates was inferred from the sum of “Naked flagellates” and “Thecate flagellates” categorized as micro-phytoplankton [67], corresponding to “Naked dinoflagellates” and “Thecate dinoflagellates” in a subsequent study using the same classification method [67]. The biomass of microzooplankton represents the combined biomass of microzooplankton and heterotrophic nanoflagellates (HNF), based on Fig. 4 in Ref. [67]. DHA contents were assumed to be 5.9% for aplanochytrids, 3.3% for dinoflagellates, and 0.4% for diatoms according to Ref. [10]. These DHA contents were derived from laboratory-cultured cells. Bold values indicate those measured or calculated in the present study. DHA, docosahexaenoic acid.

These findings indicate that although aplanochytrid biomass was relatively low, they may be significant contributors to DHA production in marine ecosystems. Their total contribution was comparable to that of dinoflagellates, which accounted for 12.4% of the total DHA standing stock among the three groups. However, in the present estimation, diatoms still had a substantially greater biomass. Therefore, the paradox in marine ecosystems remained unresolved, as primary production was clearly richer in EPA than in DHA, whereas fish contain DHA in amounts that are comparable to or greater than those of EPA [10].

### Evaluation of docosahexaenoic acid contributors to higher trophic levels based on previous studies

Calanoid copepods are widely recognized as the principal prey of marine fish [70–74]. Several metabarcoding analyses targeting copepod gut contents, conducted in regions such as the East China Sea and coastal waters, have reported the ingestion of aplanochytrids by calanoid copepods [47, 48]. In the reported metabarcoding analyses of copepod gut contents, the detection frequencies of aplanochytrids were equivalent to 25.8% [47] and 22.0% [48] those of diatoms. These values were approximately 7- and 50-fold higher than the relative abundance of aplanochytrids in the surrounding seawater (Table 2; [75]). Given that calanoid copepods optimally feed on prey between 40 and 100 μm [76], they are unlikely to directly prey on individual aplanochytrid cells, which are typically ~10 μm in size. However, direct observations of aplanochytrids in the digestive tracts of higher trophic consumers have been reported [77, 78]. Aplanochytrids may form aggregates large enough to be ingested by copepods in marine environments, as metabarcoding analyses of size-fractionated samples from the Tara Oceans project detected aplanochytrids in fractions ranging from 20 to 2000 μm, suggesting the presence of aggregated forms [42].

**Table 2:** Reported relative read abundance in ambient seawater and calanoid copepod gut contents, and reported DHA content per unit carbon biomass of DHA producers, compiled from the literature.

	Dinoflagellates	Diatoms	Aplanochytrids	Chlorophyta	Haptophyta	Cryptophyta
	(Alveolata)	(Stramenopiles)	(Stramenopiles)	(Archaeplastida)	(Haptista)	(Cryptista)
Coastal water						
Average OTU% in gut content	2.91	13.54	3.49	0.71	0.01	0.0001
Hirai et al. [47]						
Coastal water						
Average OTU% in marine environment	18.75	9.79	0.5	11.98	0.05	0.02
Hirai et al. [47]						
East China Sea – Kuroshio water						
Average OTU% in gut content	22.11	9.48	2.09	3.85	0.72	0.07
(Non-Opistokonta, Kobari et al. [48])						
East China Sea – Kuroshio water						
Average OTU% in marine environment	33.39	5.88	0.04	1.98	3.01	0.68
(Non-Opistokonta, Kobari et al. [48])						
DHA (% g C)	3.3	0.4	5.9	0.01*	1.8	0.8
[10, 75]						

Read abundances are compiled from values reported by Hirai *et al.* [47] and Kobari *et al.* [48]. *The DHA content of green algae is based on the average for species capable of *de novo* DHA synthesis, as reported by [75]. OTU, operational taxonomic unit.

Based on the reported metabarcoding analyses of calanoid copepod gut contents [44], the current study evaluated the relative contribution of each DHA supplier ingested by copepods (Table 3). The “cell number ratio”, which represents the relative abundance of cells among diatoms, dinoflagellates, and aplanochytrids, was calculated by dividing the relative ratio of each group derived from the metabarcoding analysis by the 18S rDNA copy number ratio per cell (Table S6), which was estimated using previously reported values for diatoms and dinoflagellates and a newly obtained value for aplanochytrids in this study (Table 3). To calculate the carbon ratio for each group, we multiplied the cell number ratio by the carbon biomass ratio per cell [79]. At this stage, diatoms accounted for ~90% of the total carbon among the three groups. Finally, we calculated the DHA ratio, which represents the relative contribution of each group to total DHA in the gut contents by multiplying the carbon ratio by the DHA content ratio per unit of carbon.

**Table 3:** Estimated DHA ratios in the gut contents of the calanoid copepod *Calanus sinicus* observed by Hirai *et al.* [47].

						Aplanochytrids		Diatoms		Dinoflagellates	
						(Stramenopiles)		(Stramenopiles)		(Alveolata)	
	(1)						(D)		(A)		(A)
Cell number ratio	Average read ratio of each group	/	18S rDNA (copies/cell) ratio	=	Cell number ratio	0.226/0.086 = 2.625		0.622/0.029 = 20.837		0.152/0.884 = 0.171	
	(see Table S6)										
											
	(2)						(C)		(A)		(A)
Carbon ratio	Cell number ratio	･	Carbon content	=	Carbon ratio	2.625 ･ 0.332 = 0.871		20.837 ･ 0.303 = 6.320		0.152 ･ 0.365 = 0.063	
			(pg/cell) ratio								
											
	(3)						(B)		(B)		(B)
DHA ratio	Carbon ratio	･	DHA content (% of C.B.) ratio	=	DHA ratio	0.871 ･ 0.610 = 0.532		6.320 ･ 0.345 = 0.285		0.063 ･ 0.045 = 0.022	
											
											
% of DHA ratio	Values were converted to the percentage of					0.532 /(0.532 + 0.285 + 0.022) 100					
in 3 groups	the total among the 3 groups.										
						= 63		34		3	

C.B., Carbon biomass. Due to the short read lengths in the reported metabarcoding sequencing data, weighted averages based on species-level taxonomy were not applied. (1) The proportion of 18S rDNA copies in DNA extracts was divided by the copy number per cell to estimate the relative cell abundance of each taxonomic group based on metabarcoding analysis. (2) The contribution of each taxonomic group to carbon biomass was calculated by multiplying the relative cell abundance obtained in (A) by the carbon content per cell. (3) The proportion of DHA contained in gut-derived carbon was calculated by multiplying the carbon biomass of each taxonomic group by its DHA content per unit carbon.

(A) Calculations were based on Martin *et al.* [79]. For dinoflagellates, for which sufficient correction was achieved in Martin *et al.* [79], the median values reported therein were adopted. For diatoms, which have been underestimated, only the mean carbon content values were used. (B) The mean values of brackish or marine species were adopted from Gladyshev *et al.* [10]. (C) Calculated based on Kimura *et al.* [62]. (D) Calculated based on the present study. For detailed numerical values, see Table 2 and the Supplementary Data. The estimation results indicated that aplanochytrids were the largest DHA contributors to calanoid copepods.

Although the carbon biomass of aplanochytrids was only approximately one-eighth that of diatoms among the three groups, their DHA content per unit of carbon was approximately 10-fold higher than that of diatoms, resulting in a dominant DHA contribution (63%) in the gut contents. Furthermore, when the same calculations were performed using the average read ratios of gut contents from calanoid copepods reported in East China Sea–Kuroshio water and summarized in Table 2, which compiles data from [47, 48], aplanochytrids were again identified as the largest DHA source, contributing ~40% in this calculation. This reproducibility across distinct regions suggests that the potential contribution of aplanochytrids as DHA suppliers to calanoid copepods reflect a general feeding behavior of calanoid copepods in marine ecosystems. On the contrary, as organisms involved in the detrital food chain, Fungi and Radiolaria were abundantly detected in the gut contents of calanoid copepods [48]. Radiolaria are important suppliers of PUFA in the deep sea and are known to produce odd-chain specific fatty acids [82], but there have been no reports of them producing DHA. Within Fungi, instances where Ichthyosporeans and some marine yeasts produce DHA have been reported [83], but they are rare. Thus, considering the frequent detection of aplanochytrids in the gut contents of calanoid copepods [47, 48], it is reasonable to suggest that aplanochytrids serve as a major DHA source for calanoid copepods. In general, energy is lost at each trophic step through predation in ecosystems [84]. However, previous studies have shown that DHA accumulates more efficiently than general nutrients such as sugars and non-essential fatty acids during trophic transfer [10]. Moreover, in calanoid copepods, DHA is retained even under starvation conditions [85]. Thus, the “Aplanochytrid Pathway,” in which DHA is transferred directly to calanoid copepods, can represent an efficient and previously under-evaluated pathway, distinct from pathways mediated by DHA-non-producing microzooplankton consumers.

According to data from the Tara Oceans project, labyrinthuleans account for an average of 0.1% of total reads in coastal regions located within 50 km from land [86]. In contrast, metabarcoding analyses conducted in the upwelling regions of Spain and Chile reported labyrinthuleans comprising ~2%–4% of the total reads [80,81]. In contrast to the relatively low ratio of labyrinthuleans reads reported in the Tara Oceans coastal dataset (Table 4), metabarcoding analyses from upwelling regions had shown higher relative abundances of labyrinthuleans. Consistent with these observations, and similar to diatoms in eutrophic environments, labyrinthuleans, including aplanochytrids, tended to be more abundant in productive regions, including surface waters of the non-Kuroshio region during spring, summer, and autumn as defined in this study (Table 4). Our quantitative survey also showed that peaks in aplanochytrid cell abundance were most frequently observed in the surface waters of the non-Kuroshio region (Fig. 2). On the other hand, to estimate the relative carbon contribution of each group, we focused on surface seawater data from stations A04 and A07 during spring, summer, and autumn, for which absolute abundances were comparable with those reported in previous studies. The same gut-content–based calculation was then applied to the metabarcoding-derived seawater data in this study. As a result, the relative proportion of aplanochytrids summed across three seasons was comparable to the quantitatively estimated proportions shown in Table 1 (Fig. S14). Similarly, the same calculation was applied to ambient seawater from regions corresponding to those analyzed for copepod gut contents based on Table 2. As a result, comparable proportions were observed in coastal regions, whereas lower values were obtained in the East China Sea–Kuroshio region than in the surface waters at stations A04 and A07 during spring, summer, and autumn (Fig. S14). These results suggest that selective ingestion of aplanochytrids by calanoid copepods may occur not only in regions where aplanochytrids were relatively abundant, such as stations A04 and A07, but also in regions where their abundance was lower. Therefore, the ecological importance of aplanochytrids as DHA contributors is likely to be higher in productive regions where calanoid copepods and fish are abundant. Calanoid copepods represent one of the most heavily preyed upon groups in mesozooplankton communities [48]. Therefore, our estimation that aplanochytrids are the largest contributors of DHA to calanoid copepods indicates that aplanochytrids can be the primary suppliers responsible for DHA accumulation not only in the entire mesozooplankton community but also in fish that prey upon them.

**Table 4:** Comparison of metabarcoding analysis between samples collected in this study and those from other marine areas reported in previous studies.

	Dinoflagellates	Diatoms	Cryptophyta	Haptophyta	Labyrithulea
Mediterranean	16.0%	1.7%	0.4%	1.5%	0.1%
(Tara Ocean Project) [84]					
Red sea	12.4%	0.8%	1.0%	0.7%	0.1%
(Tara Ocean Project) [84]					
Atlantic Ocean	16.5%	5.6%	1.6%	0.9%	0.1%
(Tara Ocean Project) [84]					
Upwelling area in Spain	18%	16%	1%	1%	4%
[80]					
Upwelling area in Chile	22.1%	20.4%	0.2%	1.5%	1.8%
[81]					
Non-Kuroshio surface mean	28.9%	18.4%	0.3%	0.6%	0.4%
(spring, summer, autumn, This study)					

Values for the Tara Ocean are within ~50 km off the coast, 0.8–20 μm fraction data. Values for Ríos-Castro *et al.* [80] are approximate percentage reads from Figs. 5 and 6 of that study. Sampling in Ríos-Castro *et al.* [80] was conducted throughout the year, and no extreme seasonal differences were observed in the total number of metabarcoding reads per sample. The dataset of Yarimizu *et al.* [81] was based on samples collected between January and May 2019, which corresponds to the austral summer through autumn.

As an exception, aplanochytrids have been scarcely detected in the gut contents of copepods collected from the Baltic Sea [87], which is a known low-salinity region [88, 89]. In contrast, unidentified labyrinthuleans were abundantly detected in the gut contents of cladocerans, such as *Bosmina* and *Evadne,* in the same environment. Based on these results, the ecological role of aplanochytrids can be interpreted to be not uniformly important across all marine environments. Furthermore, in regions with characteristic environmental conditions such as low salinity, it is also reasonable to speculate that other labyrinthuleans adapted to local conditions function as DHA sources, depending on the composition of zooplankton communities and surrounding environmental factors.

This study is the first to report that aplanochytrids are major DHA producers, even in comparison with primary producers. Although previous ecological studies have insufficiently evaluated aplanochytrids, the present findings show that they can serve as an important source of DHA in marine ecosystems. This conclusion is supported not only by their high DHA content but also by the feeding behavior of their predators, calanoid copepods, which are observed feeding on aplanochytrids, seemingly in preference. Furthermore, aplanochytrids take up nutrients from diverse algal sources, form aggregates, and produce DHA from organic carbon sources, including EPA, thereby transferring DHA directly to higher trophic levels, namely calanoid copepods. Moreover, the total amount of DHA transferred through this pathway can be comparable to that derived from primary producers. Therefore, in light of this mechanism, aplanochytrids represent a key group of organisms that resolve the paradox highlighted in the introduction: despite EPA generally being more abundant than DHA at the level of primary production, fish accumulate more DHA than EPA. In this study environment, in the survey area at stations A04 and A07 during spring to autumn, even when considering aplanochytrids, the concentration of EPA in seawater was approximately five-fold higher than that of DHA ([66] and this study). In this study, our results suggest that in the gut contents of calanoid copepods, the difference narrowed to 2.4-fold between EPA and DHA due to the contribution of aplanochytrids ([47, 48] and this study). Furthermore, a previous study reported that the fatty acid composition of calanoid copepods showed only approximately 1.6-fold [90] higher level of EPA compared with DHA. Given that marine fish exhibit nearly equivalent ratios of EPA and DHA, these stepwise shifts indicate that aplanochytrids are key organisms contributing to the relative increase in DHA along the grazing food chain. Taken together, and considering that labyrinthuleans tend to be detected at higher proportions in coastal and upwelling regions where fish production is abundant, we conclude that aplanochytrids are a major source of DHA supporting fish production. Nevertheless, because the differences of 1.6-fold [90] and 2.4-fold between EPA and DHA still remain, this paradox has not been completely resolved. Furthermore, not only for the present study area but also for food-web studies of mesozooplankton, including calanoid copepods across the global ocean, available data remain limited. To fully understand the paradox, further investigations are required, not only into other lineages of labyrinthuleans but also into organisms that have not yet been recognized as potential contributors.

## Supplementary Material

Morimoto_ISME_C_3rdrev_Supplementary_Material_RevisedFigureS14

Supplementary_materials_ycag178

## Data Availability

The 18S rRNA gene sequence data are available from the DNA Data Bank of Japan (DDBJ; accession number, BioProject PRJDB37751). The R script used for data processing and visualization are available at the GitHub repository (https://github.com/daiskehonda/ecological_impact_of_algal-preying_protists_aplanochytrium).
