# Supplementary material for: Ecological impact of algal-preying protists aplanochytrids (Labyrinthulea) as a source of docosahexaenoic acid for copepods in marine environments
Source: ISME Commun. 2026 Jun 26;6(1):ycag178. doi: 10.1093/ismeco/ycag178 (PMC13685785; doi:10.1093/ismeco/ycag178)
Supplement: Supplementary file 1 [file morimoto_isme_c_3rdrev_supplementary_material_revisedfigures14.docx]

**Figure S14. Calculated carbon biomass proportions of three groups in three marine regions.** Carbon contribution ratios of three groups were calculated for each marine region.For all regions except Average of Stations A04 and A07 (spring, summer, autumn) (standing stock–based) [66], values were estimated using metabarcoding data obtained in each region in combination with the calculation procedure described in Table 3. Values for Average of Stations A04 and A07 (spring, summer, autumn) (standing stock–based) [66] were derived from the numerical estimates reported in Table 1. The proportion of aplanochytrids was comparable between the Kuroshio coastal region, where selective ingestion of aplanochytrids has been reported [47], and Average of Stations A04 and A07 (spring, summer, autumn) (metabarcoding-based) examined in this study, accounting for 2.0% and 1.6% of total carbon biomass, respectively.In contrast, the East China Sea–Kuroshio region showed a lower proportion of aplanochytrid carbon biomass (0.2%) [48]; nevertheless, selective ingestion of aplanochytrids has also been reported in this region.
