# Supplementary material for: Ecological impact of algal-preying protists aplanochytrids (Labyrinthulea) as a source of docosahexaenoic acid for copepods in marine environments
Source: ISME Commun. 2026 Jun 26;6(1):ycag178. doi: 10.1093/ismeco/ycag178 (PMC13685785; doi:10.1093/ismeco/ycag178)
Supplement: Supplementary file 2 [file supplementary_materials_ycag178.zip › Morimoto_ISME_C_3rdrev_Supplementary_Material.docx]

**Ecological impact of algal-preying protists *Aplanochytrium* (Labyrinthulea) as a source of docosahexaenoic acid for copepods in marine environments**

Tomi Morimoto, Yoko Hamamoto, Takanori Shono, Mayumi Ueda, Akira Kuwata, Yukiko Taniuchi, Hiroshi Kuroda, Kazuaki Tadokoro, Yuki Tsujimura, Toshiki Miyaoka, Taichi Mogi, Hiroyuki Imai, Ryosuke Nakai, Satoshi Nagai, Tomoko Matsumoto, Jun Kikuchi and Daiske Honda

**Supplementary Material**

**Table S1.** List of strains used in this study.

|  | Name |  |  | Strain |
| --- | --- | --- | --- | --- |
|  |  |  |  |  |
| Green algae |  |  |  |  |
|  | *Pyramimonas parkeae* | |  | NIES-254 |
|  | *Tetraselmis subcordiformis* | |  | NIES-2572 |
|  | *Ostreococcus tauri* | |  | NIES-2673 |
|  | *Mantoniella squamata* | |  | NIES-1409 |
| Haptophyta |  |  |  |  |
|  | *Emiliania huxleyi* | |  | NIES-3367 |
|  | *Calyptotrosphaera sphaeroidea* | | | NIES-2854 |
|  | *Pleurochrysis roscoffensis* | |  | NIES-8 |
| Dinoflagellate |  |  |  |  |
|  | *Heterocapsa triquetra* | |  | NIES-7 |
|  | *Prorocentrum micans* | |  | NIES-12 |
| Diatom |  |  |  |  |
|  | *Skeletonema marinoi-dohrnii* complex | | | NIES-324 |
|  | Ditylum brightwellii | |  | NIES-350 |
|  | *Eucampia* sp. |  |  | NIES-2668 |
| Raphidophytes |  |  |  |  |
|  | *Heterosigma akashiwo* | |  | SEK293 |
|  | *Ochromonas danica* | |  | SEK368 |
| Chlorarachniophyta | |  |  |  |
|  | *Chlorarachnion reptans* | |  | NIES- 624 |
|  |  |  |  |  |
| Cyanobacteria |  |  |  |  |
|  | *Cyanobium* sp. | |  | NIES-981 |
|  |  |  |  |  |
| Labyrinthulea |  |  |  |  |
|  | *Aplanochytrium* sp. | |  | SEK753 |
|  | *Aplanochytrium keruguelense* | |  | KMPB-N-NA 107 |
|  | *Aplanochytrium* sp. | |  | SEK754 |
|  | *Aplanochytrium* sp. | |  | SEK755 |
|  | *Aplanochytrium* sp. | |  | SEK757 |
|  | *Aplanochytrium* sp. | |  | SEK602 |
|  | *Aplanochytrium* sp. | |  | AB973544 |
|  | *Aplanochytrium* sp. | |  | SEK756 |
|  | *Aplanochytrium* sp. | |  | AB973543 |
|  | *Aurantiochytrium limacinum* | |  | ATCC-MYA-1381 |
|  | *Parietichytrium sarkarianum* | |  | NBRC 104108 |
|  | *Botryochytrium radiatum* | |  | NBRC 110809 |
|  | *Ulkenia amoiboidea* | |  | NBRC 110830 |
|  | *Thraustochytrium aureum* | |  | ATCC34304 |
|  | *Schizochytrium aggregatum* | |  | ATCC 28209 |
|  | *Sichyidochytrium* sp. | |  | NBRC 102979 |
|  | *Oblongichytrium* sp. | |  | SEK715 |
|  | *Thraustochytrium striatum* | |  | SEK533 |

**Table S2**. Information on primers and probes used in this study.

|  |  | Name |  | Sequence |  | Reference |
| --- | --- | --- | --- | --- | --- | --- |
| qPCR |  |  |  |  |  |  |
|  | pGEM-3z | pGEM-3z-F |  | CGGCATCAGAGCAGATTGTA | | [53] |
|  |  | pGEM-3z-R |  | CTGGCGTAATAGCGAAGAGG | | [53] |
|  |  | pGEM-3z-TaqMan | | AGAAAATACCGCATCAGG | | This study |
|  |  |  |  |  |  |  |
|  | Aplanochytrium | Shono-Aplanochytrium-F | | CCTTGGTTGAAAAGCCTGGG | | This study |
|  |  | Shono-Aplanochytrium-R | | ACCGGACCATTCAATCGG | | This study |
|  |  | Shono-Aplanochytrium-TaqMan | | ACCCTTGCAATTATTGGTCT | | This study |
|  |  |  |  |  |  |  |
| Meta18S analysis |  |  |  |  |  |  |
|  | Eukaryote |  |  |  |  |  |
|  |  | SSU-F1289 |  | TGGAGYGATTTGTCTGGTTDATTCCG | | [54] |
|  |  | SSU-R1772 |  | TCACCTACGGAAACCTTGTTACG | | [54] |
|  |  |  |  |  |  |  |
|  | Labyrinthuleans |  |  |  |  |  |
|  |  | LABY-A |  | GGGATCGAAGATGATTAG | | [55] |
|  |  | LABY-Y |  | CWCRAACTTCCTTCCGGT | | [55] |
|  |  |  |  |  |  |  |

The probes were purified using HPLC with FAM as the fluorescent dye and Minor Groove Binder as the Tm enhancer.

**Table S3**. PCR protocol used in this study.

| **Analysis** | **Target** | **PCR chemistry** | **Primer set** | **Adapter sequence** | **TaqMan probe** | **PCR cycles & conditions** |  |
| --- | --- | --- | --- | --- | --- | --- | --- |
| meta-18S | Eukaryote | KOD -Plus- Ver.2 | Universal primers | with adapter | **-** | 94 °C 2 min (1 cycle); 94 °C 15 s, 60 °C 30 s, 68 °C 40 s (30 cycles); 68 °C 7 min (1 cycle) | |
| meta-18S | Labyrinthuleans* | KOD -Plus- Ver.2 | LABY-A, LABY-Y | without adapter | **-** | 94 °C 2 min (1 cycle); 94 °C 15 s, 60 °C 30 s, 68 °C 40 s (15 cycles); 68 °C 7 min (1 cycle) | |
| meta-18S | Labyrinthuleans | KOD -Plus- Ver.2 | LABY-A, LABY-Y | with adapter | **-** | 94 °C 2 min (1 cycle); 94 °C 15 s, 60 °C 30 s, 68 °C 40 s (30 cycles); 68 °C 7 min (1 cycle) | |
|  |  |  |  |  |  |  |  |
| qPCR | Estimation of 18S rDNA Copy Number per Cell | Power SYBR® Green Master Mix | Shono-Aplanochytrium-F,  Shono-Aplanochytrium-R | - | - | 50 °C 2 min (1 cycle); 95 °C 2 min (1 cycle); 95 °C 15 s, 55 °C 30 s (40 cycles) |  |
| qPCR | Estimation of DNA recovery efficiency | Power SYBR® Green Master Mix | pGEM-3z-F, pGEM-3z-R |  | - | 50 °C 2 min (1 cycle); 95 °C 2 min (1 cycle); 95 °C 15 s, 55 °C 30 s (40 cycles) |  |
| qPCR | Estimation of cell abundance | TaqManTM Environmental Master Mix 2.0 | Shono-Aplanochytrium-F,  Shono-Aplanochytrium-R | - | Shono-Aplanochytrium-TaqMan | 50 °C 2 min (1 cycle); 95 °C 10 min (1 cycle); 95 °C 15 s, 60 °C 1 min (40 cycles) |  |
| qPCR | Estimation of DNA recovery efficiency | TaqManTM Environmental Master Mix 2.0 | pGEM-3z-F, pGEM-3z-R |  | pGEM-3z-TaqMan | 50 °C 2 min (1 cycle); 95 °C 10 min (1 cycle); 95 °C 15 s, 60 °C 1 min (40 cycles) |  |
|  |  |  |  |  |  |  |  |
| PCR | Specificity check | Ex Taq | Shono-Aplanochytrium-F  Shono-Aplanochytrium-R | - | - | 94 °C 30 s (1 cycle); 94 °C 30 s, 65 °C 30 s, 72 °C 30 s (30 cycles); 72 °C 7 min (1 cycle) | |

*To amplify labyrinthulean sequences, PCR was first performed using LABY-A and LABY-Y without adapter sequences. The resulting PCR product was then used as a template for PCR with LABY-A and LABY-Y containing adapter sequences.

**Table S4.** List of media used in this study.

| d-GPY |  |
| --- | --- |
| Glucose | 2.0 g |
| Polypepton | 2.0 g |
| Yeast extract | 2.0 g |
| Sea water | 500 mL |
| Distilled water | 500 mL |
| Autoclave at 121 ℃ for 20 min | |
|  |  |
| 1/10 SeaAct Medium | |
| Glucose | 2.0 g |
| Seafood extract | 2.0 g |
| Marine Art SF-1 | 2.0 g |
| Distilled water | 1 000 mL |
| Autoclave at 121 ℃ for 20 min | |

**Table S5**. Information on the artificial DNA fragments used in this study.

| Name |  | Sequence |
| --- | --- | --- |
|  |  |  |
| pGEM-Fragment | | 5′-GTCGGGGCTGGCTTAACTATGCGGCATCAGAGCAGATTGTACTGAGAGTGCACCATATGCGGTGTGAAATACCGCACAGATGCGTAAGGAGAAAATACCGCATCAGGCGCCATTCGCCATTCAGGCTGCGCAACTGTTGGGAAGGGCGATCGGTGCGGGCCTCTTCGCTATTACGCCAGCTGGCGAAAGGGGGATGTGCT-3′ |
|  |  |  |
| Aplano-Fragment | | 5′-CGAGTTTATAACCTTGGTTGAAAAGCCTGGGTAATCTTTTGAACTTTCGTCGTGATGGGGCTAGACCCTTGCAATTATTGGTCTCCAACGAGGAATTCCTAGTAAACGCTAGTCATCAGCTTGCATTGATTACGTCCCTGCCCTTTGTACACACCGCCCGTCGCACCTACCGATTGAATGGTCCGGTGAAATCTTCGGAC-3’ |
|  |  |  |

**Table S6**. Median copy number of 18S rDNA in the two groups and aplanochytrids.

| Group | Strain | Copy number | Reference |
| --- | --- | --- | --- |
|  |  |  |  |
| Dinoflagellata |  | 4919 | [79] |
| (Alveolata) |  |  |  |
|  |  |  |  |
| Bacillariophyta |  | 166 | [79] |
| (Stramenopiles) |  |  |  |
|  |  |  |  |
| Aplanochytrids | SEK753 | 150 ± 50 | (This study) |
| (Stramenopiles) | KMPB-N-NA 107 | 190 ± 20 |  |
|  | SEK754 | 400 ± 220 |  |
|  | SEK755 | 450 ± 230 |  |
|  | SEK757 | 480 ± 160 |  |
|  | SEK602 | 580 ± 130 |  |
|  | SEK717 | 790 ± 230 |  |
|  | SEK756 | 790 ± 230 |  |
|  | SEK603 | 980 ± 240 |  |

The median value among the strains of aplanochytrids was 480 copies per cell for SEK757, and this value was used for the calculations in Table 3.

**Table S7**. Results of two-membered culture with Aplanochytrids.

|  | Name |  |  | Strain |  | Aggregate | Predation | Cell proliferation |
| --- | --- | --- | --- | --- | --- | --- | --- | --- |
|  |  |  |  |  |  |  |  |  |
| Green algae |  |  |  |  |  |  |  |  |
|  | *Pyramimonas parkeae* | |  | NIES-254 |  | + | + | + |
|  | *Tetraselmis subcordiformis* | |  | NIES-2572 |  | + | - | - |
|  | *Ostreococcus tauri* | |  | NIES-2673 |  | - | - | - |
|  | *Mantoniella squamata* | |  | NIES-1409 |  | - | - | - |
| Haptophyta |  |  |  |  |  |  |  |  |
|  | *Calyptotrosphaera sphaeroidea* | | | NIES-2854 |  | + | - | - |
|  | *Pleurochrysis roscoffensis* | |  | NIES-8 |  | - | - | - |
| Dinoflagellate |  |  |  |  |  |  |  |  |
|  | *Heterocapsa triquetra* | |  | NIES-7 |  | + | - | + |
|  | *Prorocentrum micans* | |  | NIES-12 |  | + | - | + |
| Diatom |  |  |  |  |  |  |  |  |
|  | *Skeletonema marinoi-dohrnii* complex | | | NIES-324 |  | + | + | + |
|  | Ditylum brightwellii | |  | NIES-350 |  | - | - | - |
|  | *Eucampia* sp. |  |  | NIES-2668 |  | + | + | + |
| Chlorarachniophyta | |  |  |  |  |  |  |  |
|  | *Chlorarachnion reptans* | |  | NIES- 624 |  | + | - | - |
| Cyanobacteria |  |  |  |  |  |  |  |  |
|  | *Cyanobium* sp. | |  | NIES-981 |  | - | - | - |
|  |  |  |  |  |  |  |  |  |

+: comfirmed, -: not comfirmed

**Figure S1. Agarose electrophoresis image of PCR using genomic DNA from 16 strains, including 5 *Aplanochytrium* species.** M: Maker, 1: Aplano-Fragment, 2: Negative control, 3: *Aurantiochytrium limacinum* (SR21), 4: *Parietichytrium sarkarianum* (NBRC 104108), 5: *Botryochytrium radiatum* (NBRC 110809), 6: *Ulkenia amoiboidea* (NBRC 110830), 7: *Thraustochytrium aureum* (ATCC34304), 8: *Schizochytrium aggregatum* (ATCC 28209), 9: *Sichyidochytrium* sp. (NBRC 102979), 10: *Oblongichytrium* sp. (SEK715), 11: *Thraustochytrium striatum* (SEK533), 12: *Aplanochytrium kergelence* (KMPB N-BA-107), 13: *Aplanochytrium* sp. (SEK602), 14: *Aplanochytrium* sp. (SEK717), 15: *Aplanochytrium* sp. (SEK755), 16: *Aplanochytrium* sp. (SEK756), 17: *Heterosigma akashiwo* (SEK293), 18: *Skeletonema marinoi-dohrnii* complex (NIES324)

**Figure S2. Predation on *Pyramimonas parkeae*.** The green cell was shrunken throughout, as observed in the *Skeletonema marinoi–dohrnii* complex. Arrowheads indicate shrinking cells. Panels (a–f) represent a time-lapse sequence covering 12 h in total.

Figure S3. (A)

Figure S3. (B)

Figure S3. (C)

Figure S3. (D)

Figure S3. (E)

Figure S3. **Seasonal changes in the vertical distribution of aplanochytrids and chlorophyll a concentration in surface waters of the study region.**Vertical distributions of aplanochytrids and chlorophyll a concentration in the surface layer of the study region are shown for (A) July 2021, (B) October 2021, (C) January 2022, (D) March 2022, and (E) May 2022. Unlike Figure 1, depth is presented on a linear scale. Gray shading indicates the seafloor, and black dots denote sampling depths. In most samples, maxima in aplanochytrid cell density were observed in surface waters and generally coincided with elevated chlorophyll a concentrations.

Figure S4. **Non-metric multidimensional scaling (NMDS) analysis with environmental vector fitting (envfit) based on metabarcoding data using labyrinthulean-specific primers.** Arrows represent environmental variables significantly associated with community structure (p ≤ 0.05), including depth (blue), temperature (red), relative abundance of Chlorophyta (green), relative abundance of Dinophyceae (orange), and relative abundance of Calanoida (black). Point colors indicate the dominant taxonomic group in each sample, defined as the group accounting for more than 40% of the relative abundance and having the highest proportion. Relative abundance was calculated from eukaryotic metabarcoding data as the proportion of reads assigned to each taxon relative to the total number of eukaryotic reads in each sample. Chlorophyll concentration was also included in the analysis but did not show a significant relationship with the NMDS ordination (p = 0.992, r² = 0.0002) and is therefore not displayed.

**Figure S5. Spearman’s rank correlation between aplanochytrid cell density and chlorophyll a concentration.**Spearman’s rank correlation analysis revealed a moderate positive correlation between aplanochytrid cell density and chlorophyll a concentration (ρ = 0.66, p < 0.001).

**Figure S6. Two-membered culture with Aplanochytrids and Mamiellophyceae.** Aplanochytrid cells did not respond to Mamiellophyceae. (A,B) *Ostreococcus tauri*, (C,D) *Mantoniella squamata.* Red circle, alanochytrids.

**Figure S7. Two-membered culture with aplanochytrids and cyanobacteria.** Aggregate formation was observed with *Cyanobium* sp., but no increase in the number of aplanochytrid cells was detected. (A) Observation at 1 h; (B) observation at 33 h.

**Figure S8. Two-membered culture with aplanochytrids and green algae.** Aggregate formation with algae was observed in both *Pyramimonas parkeae* and *Tetraselmis subcordiformis.* (A,B) *Pyramimonas parkeae,* (C,D) *Tetraselmis subcordiformis.*

**Figure S9. Two-membered cultures of aplanochytrids and haptophytes.** (A,B) *Calyptrosphaera sphaeroidea*, (C,D) *Pleurochrysis roscoffensis*. Arrowheads indicate aggregates of aplanochytrids and *Calyptrosphaera.* (B) Aggregate formation with both algae was observed. (D) An increase in aplanochytrid cells was not observed until the end of the two-membered culture.

**Figure S10. Two-membered cultures of aplanochytrids and *Prorocentrum*.** (A) A large number of aplanochytrid cells were attached to *Prorocentrum micans*. (B) Fluorescence image of (A) with DAPI staining. (C) Aplanochytrids formed large aggregates. The cell density of aplanochytrids increased.

**Figure S11. Two-membered cultures of aplanochytrids and *Heterocapsa*.** (B) In co-culture with *Heterocapsa triquetra*, aggregates were formed and changes such as shrinkage of cell contents were observed, similar to those in the co-culture with *Prorocentrum*. Subsequently, cell density increased.

**Figure S12. Two-membered culture with aplanochytrids and *Chlorarachniophyte*.** (B) Aggregate formation with *Chlorarachnion reptans* was observed. However, an increase in aplanochytrid cells was not observed.

Figure S13 (A)

Figure S13 (B)

Figure S13 (C)

**Figure S13. Docosahexaenoic acid (DHA) production by labyrinthuleans and transfer to zooplankton.** (A) Temporal changes in cell abundance in a two-membered culture of *Skeletonema* and aplanochytrids. The bars indicate the cell density of diatoms, with orange representing the number of live cells and grey representing the number of dead cells. The error bars represent the standard deviation. (B) Eicosapentaenoic acid (EPA) and DHA ratios in two-membered cultures of aplanochytrids and *Skeletonema.* The graphs show the ratios of DHA and EPA in monocultures and the changes in the ratios during co-cultivation. Since a fixed amount of internal standard was added, the fatty acid levels can be compared across days. (C) EPA/DHA ratio of *Aurantiochytrium* and *Artemia.* Left: ratios of DHA and EPA in *Aurantiochytrium* alone. Right: ratios of DHA and EPA in *Artemia*. Error bars represent standard deviation.

**Figure S14. Calculated carbon biomass proportions of three groups in three marine regions.** Carbon contribution ratios of three groups were calculated for each marine region.For all regions except the Oyashio-influenced region (standing stock–based) [66], values were estimated using metabarcoding data obtained in each region in combination with the calculation procedure described in Table 3.The Oyashio-influenced region (standing stock–based) [66] were derived from the numerical estimates reported in Table 1. The proportion of aplanochytrids was comparable between the Kuroshio coastal region, where selective ingestion of aplanochytrids has been reported [47], and the Oyashio-influenced region examined in this study, accounting for 2.0% and 1.5% of total carbon biomass, respectively.In contrast, the East China Sea–Kuroshio region showed a lower proportion of aplanochytrid carbon biomass (0.2%) [48]; nevertheless, selective ingestion of aplanochytrids has also been reported in this region.
