# Supplementary material for: Ecological impact of algal-preying protists aplanochytrids (Labyrinthulea) as a source of docosahexaenoic acid for copepods in marine environments
Source: ISME Commun. 2026 Jun 26;6(1):ycag178. doi: 10.1093/ismeco/ycag178 (PMC13685785; doi:10.1093/ismeco/ycag178)
Supplement: Supplementary file 2 [file supplementary_materials_ycag178.zip › Morimoto_ISME_C_3rdrev_supplemental_data.zip!/Morimoto_ISME_C_3rdrev_supplemental_data/Supplemental_Data_1/Primer_Information.docx]

**Color annotations:**

Green region: Extension sequence for the second PCR

Yellow region: First PCR primer sequence

**Primer:**

Shono-Aplanochytrium-F

ACACTCTTTCCCTACACGACGCTCTTCCGATCTCCTTGGTTGAAAAGCCTGGG

Shono-Aplanochytrium-R

GTGACTGGAGTTCAGACGTGTGCTCTTCCGATCTACCGGACCATTCAATCGG
