# Supplementary material for: Ecological impact of algal-preying protists aplanochytrids (Labyrinthulea) as a source of docosahexaenoic acid for copepods in marine environments
Source: ISME Commun. 2026 Jun 26;6(1):ycag178. doi: 10.1093/ismeco/ycag178 (PMC13685785; doi:10.1093/ismeco/ycag178)
Supplement: Supplementary file 2 [file supplementary_materials_ycag178.zip › Morimoto_ISME_C_3rdrev_supplemental_data.zip!/Morimoto_ISME_C_3rdrev_supplemental_data/Supplemental_Data_1/Kathablepharidae_18S_Alignment.docx]

>JN934894.1_Katablepharis_sp._POVA-MDc39_18S_ribosomal_RNA_gene_partial_sequence

---------------------------------------------------------------------------------------------------ATGGCTCATTAAATCAGTTATCGTTTATTTGATGATCTCTTATTACTTGGATAACCGTAGTAATTCTAGAGCTAATACATGCACACAATCCCGACTTCTGGAAGGGATGTATTTATTAGATACAAAACCAAT--GCGCTTCGGCGGTGATTGGTGATTCATAATAACTTTGCGAATCGTATGACCTCGTGTCGACGATGAATCATTCAAATTTCTGCCCTATCAACTTTCGATGGTAGGATAGAGGCCTACCATGGTGGTAACGGGTAACGGAGAATTAGGGTTCGATTCCGGAGAGGGAGCCTGAGAGACGGCTACCACATCCAAGGAAGGCAGCAGGCGCGCAAATTACCCAATCCTGACTCAGGGAGGTAGTGACAATAAATAACAATACAGGGCCTTTTCAGGTCTTGTAATTGGAATGAGAACAATTTAAATCCCTTATCGAGGATCCATTAGAGGGCAAGTCTGGTGCCAGCAGCCGCGGTAATTCCAGCTCTAATAGCGTATATTAATGTTGTTGCA--GTTAAAAAGCTCGTAGTTGGATTTTGGTGTTCGACAAATGGT-CTGCTTCG-GTTTGTACTGTTT-GTTGGCGCTTCTTTTCTGGGGAACT-CACTGCTCTTAACTGA-GGGGTGTTGTAACGCAGATCGTTTACTTTGAAAAAATTAGAGTGTTCAAAGCAGGCCAACGCATTTTGAATACATTAGCATGGAATAATGGAATAGGACTTTGGTCTTATTTTGTTGGTTTCTAAGGCCGAGGTAATGATTAATAGGGATAGTTG------------------------------------------------------------------------------------------GGGGCGTTTATATTCC-GTTGTCAGAGGTGAAATTCTTGGATTTACGGAAGATAAACTACTGCGAAAGCATTCGCCAAGGATGTTTTCATTAATCAAGAACGAAAGTTAGGGGATCGAAGACGATCAGATACCGTCGTAGTCTTAACCATAAACCATGCCGACTAGGGATCAGCAGACGTTCTTTTACGACTCTGTTGGCACCTTGTGAGAAATCAAAGTTTTTGGGTTCCGGGGGGAGTATGGTCGCAAGGC-TGAAACTTAAAGGAATTGACGGAAGGGCACCACCAGGAGTGGAGCCTGCGGCTTAATTTGA-CTCAACACGGGGAAACTTACCAGGTCCAGACATGAGAAGGATTGACAGATTGAGAGCTCTTTCTTGATTCTATGGGTGGTGGTGCATGGCCGTTCTTAGTTGGTGGAGTGATTTGTCTGGTTAATTCCGTTAACGAACGAGACCTTAACCTACTAAATAGTAACGTTCACATTTTTGTGGGCGGACTACTTCTTAGAGGGACTATTCGTGCCTAGCGAGTGGAAGTTTGAGGCAATAACAGGTCTGTGATGCCCTTAGATGTTCTGGGCCGCACGCGCGCTACACTGATGAAAGCAACAAGCCTACAACCTTGGT**C**GAAA**G**GCCTGGGTAATCTTGC-AAATTTCATCGTGATGGGGATAGA**TTA**TTGCAA**C**TATT**AA**TCTTCAACGAGGAATTCCTAGTAAGCGTGAGTCATCAGCTCGCGTTGATTACGTCCCTGCCCTTTGTACACACCGCCCGTCGCTCCTACCGATTGAATGGTCCGGTGAATTCTTCGGATTGT-GCGCAGCAA-AGTTTCGGCGGAGCTGTGC--GTGAAAAGTTGAATAAACCTTATCATTTAGAGGAAGTAAA------------------------------------------------------------------------------------------------------------------------------------------------------------------------------------------------------------------------------------------------------------------------------------------------------------------------------------------------------------------------------------------------------------------------------------------------------------------------------------------------------------------------------------------------------------------------------------------------------------------------------------------------------------------

>AB772585.1_Hatena_arenicola_gene_for_18S_ribosomal_RNA_partial_sequence_isolate:_HY-001

---------------------------------------------AGCCATGCATGTCT-------AAGTGTAAATAATCTTATACTGTGAAACTGCGAATGGCTCATTAAATCAGTTATCGTTTATTTGATGGTCTCTTACTACTTGGATAACCGTAGTAATTCTAGAGCTAATACATGCACAAAATCCCGACTTCTGGAAGGGATGTATTTATTAGATAAAAAACCAAT--GGCTTCGGTC--TTTTTGGTGATTCATAATAACTTCGCGAATCGCATGGTCTTGCACCGGCGATGACTCATTCAAATTTCTGCCCTATCAACTTTCGATGGTAGGATAGAGGCCTACCATGGTGGTAACGGGTAACGGAGAATTAGGGTTCGATTCCGGAGAGGGAGCATGAGAGACGGCTACCACATCCAAGGAAGGCAGCAGGCGCGCAAATTACCCAATCCTGACACAGGGAGGTAGTGACAATAAATAACAATACAGGGCCTTTTCAGGTCTTGTAATTGGAATGAGAACAATTTAAATCCCTTATCGAGGATCCATTAGAGGGCAAGTCTGGTGCCAGCAGCCGCGGTAATTCCAGCTCTAATAGCGTATATTAATGTTGTTGCA--GTTAAAAAGCTCGTAGTTGAAATTTGGAGTTTGACAGAAGGTCTGCCTAAGGGTGTGTACTTTCT-GTTGGCTCTTCTTTTCTGGGGACCT-CACTGCTCTTCACTGA-GGGGTGTTGGAACGCAGATCGTTTACTTTGAAAAAATTAGAGTGTTCAAAGCAGGCCATCGCATT--GAATACATTAGCATGGAATAATGGAATAGGACTTTGGTCTTATTTTGTTGGTTTCTAAGGCCGAGGTAATGATTAATAGGGATAGTTG------------------------------------------------------------------------------------------GGGGCGTTTATATTCC-GTTGTCAGAGGTGAAATTCTTGGATTTACGGAAGATAAACTAGTGCGAAAGCATTCGCCAAGGATGTTTTCATTAATCAAGAACGAAAGTAGGGGGATCGAAGACGATCAGATACCGTCGTAGTCTCTACCATAAACAATGCCGACTGGGGATCAGCGGACGTTCTCTAACGACTCCGTTGGCACCCTGTGAGAAATCAAAGTTTTTGGGTTCCGGGGGGAGTATGGTCGCAAGGC-TGAAACTTAAAGGAATTGACGGAAGGGCACCACCAGGAGTGGAGCCTGCGGCTTAATTTGA-CTCAACACGGGGAAACTTACCAGGTCCAGACATAGTAAGGATTGACAGATTGAGAGCTCTTTCTTGATTCTATGGGTGGTGGTGCATGGCCGTTCTTAGTTGGTGGAGTGATTTGTCTGGTTAATTCCGTTAACGAACGAGACCTTAACCTGCTAAATAGTTACGCAAA-ATTTGT-TTCGTGGACAACTTCTTAGAGGGACTAATCGTGCCTAGCGATTGGAAGTTTGAGGCAATAACAGGTCTGTGATGCCCTTAGATGTTCTGGGCCGCACGCGCGCTACACTGATGAAAGCAACAAGTCTATAACCT**GCA**T**C**GAAA**G**G**TG**TGGGTAATCTTGC-AAATTTCATCGTGATGGGGATAGA**TTA**TTGCAATTATT**AA**TCTTCAACGAGGAATTCCTAGTAAGCGTGATTCATCAGATCGCGTTGATTACGTCCCTGCCCTTTGTACACACCGCCCGTCGCTCCTACCGATTG**G**ATGGTCCGGTGAAATCTTCGGACCGCAGTGACAGTACTTGTTCGCAAGTGCCGATACCGTGGGAAGTTGATTAAACCTTACCATCTAGAGGAAGGAGAAG----------------------------------------------------------------------------------------------------------------------------------------------------------------------------------------------------------------------------------------------------------------------------------------------------------------------------------------------------------------------------------------------------------------------------------------------------------------------------------------------------------------------------------------------------------------------------------------------------------------------------------------------------------------

>JF791095.1_Leucocryptos_marina_clone_3907_18S_ribosomal_RNA_gene_partial_sequence

-------TGATCCTGCCAGTAGTCATATGCTTGTCTCAAAGATTAAGCCATGCATGTCTCCCAA----GTGTAAATAAGTCTATACTGTGAAACTGCGAATGGCTCATTAAATCAGTTATCGTTTATTTGATGATCTCTTATTACTTGGATAACCGTAGTAATTCTAGAGCTAATACATGCACACAATCCCGACTTCTGGAAGGGATGTATTTATTAGATACAAAACCAAT--GCGCTTCGGCGGTGATTGGTGATTCATAATAACTTTGCGAATCGTATGACCCCGTGTCGACGATGAATCATTCAAATTTCTGCCCTATCAACTTTCGATGGTAGGATAGAGGCCTACCATGGTGGTAACGGGTAACGGAGAATTAGGGTTCGATTCCGGAGAGGGAGCCTGAGAGACGGCTACCACATCCAAGGAAGGCAGCAGGCGCGCAAATTACCCAATCCTGACTCAGGGAGGTAGTGACAATAAATAACAATACAGGGCCTTTTCAGGTCTTGTAATTGGAATGAGAACAATTTAAATCCCTTATCGAGGATCCATTAGAGGGCAAGTCTGGTGCCAGCAGCCGCGGTAATTCCAGCTCTAATAGCGTATATTAATGTTGTTGCAACGTTAAAAAGCTCGTAGTTGGATTTTGGTGTTCGACAAATGGT-CTGCTTCG-GTTTGTACTGTTT-GTTGGCGCTTCTTTTCTGGGGAACT-CACTGCTCTTAACTGAAGGGGTGTTGTAACGCAGATCGTCTACTTTGAAAAAATTAGAGTGTTCAAAGCAGGCCAACGCATTTTGAATACATTAGCATGGAATAATGGAATAGGACTTTGGTCTTATTTTGTTGGTTTCTAAGGCCGAGGTAATGATTAATAGGGATAGTTG------------------------------------------------------------------------------------------GGGGCGTTTATATTCCCGTTGTCAGAGGTGAAATTCTTGGATTTACGGAAGATAAACTACTGCGAAAGCATTCGCCAAGGATGTTTTCATTAATCAAGAACGAAAGTTAGGGGATCGAAGACGATCAGATACCGTCGTAGTCTTAACCATAAACCATGCCGACTAGGGATCAGCAGACGTTCTTTTACGACTCTGTTGGCAACTTGTGAGCATTCAAAGGTTTTGGGTTCCGGGGGGAGTATGGTCGCAAGGCCTGAAACTTAAAGGAATTGACGTAAGGGCACCACCAGGAGTGGAACCTGCGGCCTAATTTGA-CTCAAAACGGGGAAACTTACCAGGTCCAGACATGAGAAGGATTGACAGATTGAGAGCTCTTTCTTGATTCTATGGGTGGTGGTGCATGGCCGTTCTTAGTTGGTGGAGTGATTTGTCAGGCTAATTCCGTTAACGAACGAGACCTTAACCTGCTAAATAGTTACACCTATCTTCGGATAGGTGGGCAACTTCTTAGAGGGACTTTGCGTGTCTAACGCAAGGAAGTTTGAGGCAATAACAGGTCTGTGATGCCCTTAGATGTTCTGGGCTGCACGCGCGCTACACTGATGCGTTCAACGAGT-TATGGCCTTGTCCGATAGGATTGGGTAATCTTCTGAAAACGCATCGTGATGGGGATAGA**TTA**TTGCAATTATT**AA**TCTTCAACGAGGAATTCCTAGTAAGCGCGAGTCATCAGCTCGTGCTGATTACGTCCCTGCCCTTTGTACACACCGCCCGTCGCTCCTACCGATTGA**G**TG**A**TCCGGTGAATCATTCGGACCGCAGCAGAACTC-AGTTCCTG-GGAGCTGCA---GCGGGAAGTTTCGTGAACCTTATCACTTAGAGGAAGGAGAAGTCGTAACAAGGTTTCCGTAGGT------------------------------------------------------------------------------------------------------------------------------------------------------------------------------------------------------------------------------------------------------------------------------------------------------------------------------------------------------------------------------------------------------------------------------------------------------------------------------------------------------------------------------------------------------------------------------------------------------------------------------------------

>JF791094.1_Leucocryptos_marina_clone_3902_18S_ribosomal_RNA_gene_partial_sequence

-------TGATCCTGCCAGTAGTCATATGCTTGTCTCAAAGATTAAGCCATGCATGTCTCCCAACTAAGTGTAAATAAGTCTATACTGTGAAACTGCGAATGGCTCATTAAATCAGTTATCGTTTATTTGATGATCTCTTATTACTTGGATAACCGTAGTAATTCTAGAGCTAATACATGCACACAATCCCGACTTCTGGAAGGGATGTATTTATTAGATACAAAACCAAT--GCGCTTCGGCGGTGATTGGTGATTCATAATAACTTTGCGAATCGTATGACCCCGTGTCGACGATGAATCATTCAAATTTCTGCCCTATCAACTTTCGATGGTAGGATAGAGGCCTACCATGGTGGTAACGGGTAACGGAGAATTAGGGTTCGATTCCGGAGAGGGAGCCTGAGAGACGGCTACCACATCCAAGGAAGGCAGCAGGCGCGCAAATTACCCAATCCTGACTCAGGGAGGTAGTGACAATAAATAACAATACAGGGCCTTTTCAGGTCTTGTAATTGGAATGAGAACAATTTAAATCCCTTATCGAGGATCCATTAGAGGGCAAGTCTGGTGCCAGCAGCCGCGGTAATTCCAGCTCTAATAGCGTATATTAATGTTGTTGCA--GTTAAAAAGCTCGTAGTTGGATTTTGGTGTTCGACAAATGGTGCTGCTTCGTGTTTGTACTGTTT-GTTGGCGCTTCTTTTCTGGGGAACT-CACTGCTCTTAACTGA-GGGGTGTTGTAACGCAGATCGTCTACTTTGAAAAAATTAGAGTGTTCAAAGCAGGCCAACGCATTTTGAATACATTAGCATGGAATAATGGAATAGGACTTTGGTCTTATTTTGTTGGTTTCTAAGGCCGAGGTAATGATTAATAGGGATAGTTG------------------------------------------------------------------------------------------GGGGCGTTTATATTCC-GTTGTCAGAG-TGAAATTCTTGGATTTACGGAAGATAAACTACTGCGAAAGCATTCGCCAAGGATGTTTTCATTAATCAAGAACGAAAGTTAGGG-ATCGAAGACGATCAGATACCGTCGTAGTCTTAACCATAAATCATGCCGACTAGGGATCAGCAGACGTTCTTTTACGACTCTGTTGGCACCTTGGGAGAAATCACAGTTTTTGGGTCGGGGGGGGAGTATGGTCGCAAGGC-TGAACCTTAAATGAATTGACGGTAGGGCACATTCAGGAGTGGAGCTTGCGGCTTAATTTGATCTCAACACGTGGAACCTTACCCGGTCCAGACATGAGAAGGATTGACAGATTGAGAGCTCTTTCTTGATTCTATGGGTGGTGGTGCATGGCCGTTCTTAGTTGGTGGAGTGATTTGTCAGGCTAATTCCGTTAACGAACGAGACCTTAACCTGCTAAATAGTTACACCTATCTTCGGATAGGTGGGCAACTTCTTAGAGGGACTTTGCGTGTCTAACGCAAGGAAGTTTGAGGCAATAACAGGTCTGTGATGCCCTTAGATGTTCTGGGCTGCACGCGCGCTACACTGATGCGTTCAACGAGT-TATGGCCTTGTCCGATAGGATTGGGTAATCTTCTGAAAACGCATCGTGATGGGGATAGA**TTA**TTGCAATTATT**AA**TCTTCAACGAGGAATTCCTAGTAAGCGCGAGTCATCAGCTCGTGCTGATTACGTCCCTGCCCTTTGTACACACCGCCCGTCGCTCCTACCGATTGA**G**TG**A**TCCGGTGAATCATTCGGACCGCAGCAGAACTC-AGTTCCTG-GGAGCTGCA---GCGGGAAGTTTCGTGAACCTTATCACTTAGAGGAAGGAGAAGTCGTAACAAGGTTTCCGTAGGT------------------------------------------------------------------------------------------------------------------------------------------------------------------------------------------------------------------------------------------------------------------------------------------------------------------------------------------------------------------------------------------------------------------------------------------------------------------------------------------------------------------------------------------------------------------------------------------------------------------------------------------

>HQ191366.1_Uncultured_Katablepharidaceae_clone_PA2009C5_18S_ribosomal_RNA_gene_internal_transcribed_spacer_1_5.8S_ribosomal_RNA_gene_and_internal_transcribed_spacer_2_complete_sequence_and_28S_ribosomal_RNA_gene_partial_sequence

-------------------------------------AAAGATTAAGCCATGCATGTCT-------AAGTGTAAATAAGTCTATACTGTGAAACTGCGAATGGCTCATTAAATCAGTTATCGTTTATTTGATGATCTCTTACTACTTGGATAACCGTAGTAATTCTAGAGCTAATACATGCACAAAATCCCGACTTACG-AAGGGATGTATTTATTAGATACAAA-CCAAC--A----TCG--------TGGTGATTCATAATAACTTTGCGAATCGTATG-CCTCACGGCGACGATGAATCATTCAAATTTCTGCCCTATCAACTTTCGATGGTAGGATAGAGGCCTACCATGGTGGTAACGGGTAACGGAGAATTAGGGTTCGATTCCGGAGAGGGAGCCTGAGAGACGGCTACCACATCCAAGGAAGGCAGCAGGCGCGCAAATTACCCAATCCTGACACAGGGAGGTAGTGACAATAAATAACAATACAGGGCCTTTTCAGGTCTTGTAATTGGAATGAGAACAATTTAAATCCCTTATCGAGGATCCATTAGAGGGCAAGTCTGGTGCCAGCAGCCGCGGTAATTCCAGCTCTAATAGCGTATATTAATGTTGTTGCA--GTTAAAAAGCTCGTAGTTGGATCTTGGTGTCTGTTAAGCT-TGTTGCCTAACGGTAATACGAGTTTGACGGCACTTCTTTTCTGAACGACTATGCTGATCTTAACGGG-TGGGCATAGGAGT-CAGATCGTTTACTTTGAAAAAATTAGAGTGTTCAAAGCAGGCCAACGCATT--GAATACATTAGCATGGAATAATGGAATAGGACTTTGGTCATATTTTGTTGGTT-CGAAGACCGAGGTAATGATTAATAGGGATAGTTGGGGGCGTTTATATTCCGTTGTCAGAGGTGAAATTCTTGGATTTACGGAAGATAAACTACTGCGAAAGCATTCGCCAAGGATGTATAGTTGGGGGCGTTTATATTCC-GTTGTCAGAGGTGAAATTCTTGGATTTACGGAAGATAAACTACTGCGAAAGCATTCGCCAAGGATGTTTTCATTAATCAAGAACGAAAGTTAGGGGATCGAAGACGATCAGATACCGTCGTAGTCTTAACCATAAACCATGCCGACTAGGGATCAGCAGACGTTCTTTAACGACTCTGTTGGCACCTTGTGAGAAATCAAAGTTTTTGGGTTCCGGGGGGAGTATGGTCGCAAGGC-TGAAACTTAAAGGAATTGACGGAAGGGCACCACCAGGAGTGGAGCCTGCGGCTTAATTTGA-CTCAACACGGGGAAACTTACCAGGTCCAGACATAGTAAGGATTGACAGATTGAGAGCTCTTTCTTGATTCTATGGGTGGTGGTGCATGGCCGTTCTTAGTTGGTGGAGTGATTTGTCTGGTTAATTCCGTTAACGAACGAGACCTTAACCTGCTAAATAGTTACCTTCACAATCTG-TGGGGGGACAACTTCTTAGAGGGACTATTCGTGCCTAGCGAGTGGAAGTTTGAGGCAATAACAGGTCTGTGATGCCCTTAGATGTTCTGGGCCGCACGCGCGCTACACTGATGAAAGCAACAAGCAT-TCACCTCGTTCGACAGGACCGGGTAATCTTGC-AAATTTCATCGTGATGGGGATAGA**TTA**TTGCAA**C**TATT**AA**TCTTCAACGAGGAATTCCTAGTAAGCGTGAGTCATCAGCTCGCGTTGATTACGTCCCTGCCCTTTGTACACACCGCCCGTCGCTCCTACCGATTGAATG**A**TCCGGTGAAATCTTCGGACTGCGGCTGAGGC---GGTTCGCCGTCGATTCT---GCGGGAAGTTGATTAAACCTTATCATTTAGAGGAAGGAGAAGTCGTAACAAGGTTTCCGTAGGTGAACCTGCGGAAGGATCATTGACACCAATCCATTACCAACCCGGCGCATCGTTATTGTCTTTTTACCTTTTTAACCCTTTCAACCATCCATTCTAATCTAGTGCGGCGAAGGCCGTGCGAGTCGAAAATCCGAAACAACTCTTAACAATGGATATCGTGGCTCTCGCAACGATGAAGAACGCAGCGAAATGCGATACGTAATGTGAATTGCAGAATTCAGTGAATCATCAAAACTTTGAACGCACATTGCGCTGTGCTCGCGCACAGCATGCCTGTTTGAGTGTCGGCTCCCCAACCTCACCACCCAACCCGCCTCCCTCCTAACCGAAGGGAGGCTGGGGAGGGCTGGTGGACGTGAGCGTTTCAGTTGGCGGCGCGGCCATCTCGCATGGTCGACCGGCAGGCGAATCGCTTGAAGTGGAGCGGCAACCGTGCGGCGCGCCTCGTCTAATAGCGAGGCAGCTTGGCAAACGCAACCAGTGTGTTAGCCTCCTCTCGGGGAAGTACCATTGGCTGCGGCATGACACGCGTCCGGACGGTCTCAATCGCCTTGACGTAAGGAGACTTACCAATCAAAGCCTCGACCTCAAATCAGGTAAGACTACCCGCTGAATTTAA

>GU067879.1_Uncultured_Katablepharidaceae_clone_ESS270406.038_18S_ribosomal_RNA_gene_partial_sequence

ACCTGGTTGATCCTGCCAGTAGTCATATGCTTGTCTCAAAGATTAAGCCATGCATGTCT-------AAGTGTAAATAAGTCTATACTGTGAAACTGCGAATGGCTCATTAAATCAGTTATCGTTTATTTGATGATCTCTTACTACTTGGATAACCGTAGTAATTCTAGAGCTAATACATGCACAAAATCCCGACTTACG-AAGGGATGTATTTATTAGATACAAA-CCAAC--A----TCG--------TGGTGATTCATAATAACTTTGCGAATCGTATG-CCTCACGGCGACGATGAATCATTCAAATTTCTGCCCTATCAACTTTCGATGGTAGGATAGAGGCCTACCATGGTGGTAACGGGTAACGGAGAATTAGGGTTCGATTCCGGAGAGGGAGCCTGAGAGACGGCTACCACATCCAAGGAAGGCAGCAGGCGCGCAAATTACCCAATCCTGACACAGGGAGGTAGTGACAATAAATAACAATACAGGGCCTTTTCAGGTCTTGTAATTGGAATGGGAACAATTTAAATCCCTTATCGAGGATCCATTAGAGGGCAAGTCTGGTGCCAGCAGCCGCGGTAATTCCAGCTCTAATAGCGTATATTAATGTTGTTGCA--GTTAAAAAGCTCGTAGTTGGATTTTGGTG-CCGTCACGCT-TGCCGCCTCACGGTAGTGCGATCGGGACGACACTTCTTTTCTGAACGACTATGCTGATCTTCACGGA-TGGGCATAGGAGT-CAGATCGTTTACTTTGAAAAAATTAGAGTGTTCAAAGCAGGCCAACGCATT--GAATACATTAGCATGGAATAATGGAATAGGACTTTGGTCATATTTTGTTGGTT-CGAAGACCGAGGTAATGATTAATAGGGATAGTTG------------------------------------------------------------------------------------------GGGGCGTTTATATTCC-GTTGTCAGAGGTGAAATTCTTGGATTTACGGAAGATAAACTACTGCGAAAGCATTCGCCAAGGATGTTTTCATTAATCAAGAACGAAAGTTAGGGGATCGAAGACGATCAGATACCGTCGTAGTCTTAACCATAAACCATGCCGACTAGGGATCAGCAGACGTTCTTTAACGACTCTGTTGGCACCTTGTGAGAAATCAAAGTTTTTGGGTTCCGGGGGGAGTATGGTCGCAAGGC-TGAAACTTAAAGGAATTGACGGAAGGGCACCACCAGGAGTGGAGCCTGCGGCTTAATTTGA-CTCAACACGGGGAAACTTACCAGGTCCAGACATAGTAAGGATTGACAGATTGAGAGCTCTTTCTTGATTCTATGGGTGGTGGTGCATGGCCGTTCTTAGTTGGTGGAGTGATTTGTCTGGTTAATTCCGTTAACGAACGAGACCTTAACCTGCTAAATAGTTACCTTCACTTTCTG-TGGGGGGACAACTTCTTAGAGGGACTATTCGCGCCTAGCGAGTGGAAGTTTGAGGCAATAACAGGTCTGTGATGCCCTTAGATGTTCTGGGCCGCACGCGCGCTACACTGATGAAAGCAACAAGCAT-TCACCTCGTTCGACAGGACCGGGTAATCTTGC-AAATTTCATCGTGATGGGGATAGA**TTA**TTGCAA**C**TATT**AA**TCTTCAACGAGGAATTCCTAGTAAGCGTGAGTCATCAGCTCGCGTTGGTTACGTCCCTGCCCTTTGTACACACCGCCCGTCGCTCCTACCGATTGAATG**A**TCCGGTGAAATCTTCGGACTGCGGCTGAGGC---GGTTCGCCGTCGATGCT---GCGGGAAGTTGATTAAACCTTATCATTTAGAGGAAGGAGAAGTCGTAACAAGGTTTCCGTAGGTGAACCTGCAGAAGGATCA------------------------------------------------------------------------------------------------------------------------------------------------------------------------------------------------------------------------------------------------------------------------------------------------------------------------------------------------------------------------------------------------------------------------------------------------------------------------------------------------------------------------------------------------------------------------------------------------------------------------

>AY919672.1_Kathablepharis_remigera_18S_ribosomal_RNA_gene_partial_sequence

--------------------AGTCATATGCTTGTCTCAAAGATTAAGCCATGCATGTCT-------AAGTGTAAATAATTTTATACTGTGAAACTGCGAATGGCTCATTAAATCAGTTATCGTTTATTTGATGGTCTCTTACTACTTGGATAACCGTAGTAATTCTAGAGCTAATACATGCACAAAATCCCGACTTCTGGAAGGGATGTATTTATTAGATTAAAAACCTTCCCGGTTCACGCCGGTCTTTGGTGATTCATAATAACTTCGCGAATCGCATGGCTTTACGCTGGCGATGATTCATTCAAATTTCTGCCCTATCAACTTTCGATGGTAGGATAGAGGCCTACCATGGTGGTAACGGGTAACGGAGAATTAGGGTTCGATTCCGGAGAGGGAGCCTGAGAGACGGCTACCACATCCAAGGAAGGCAGCAGGCGCGCAAATTACCCAATCCTGACACAGGGAGGTAGTGACAATAAATAACAATACAGGGCCTTTACAGGTCTTGTAATTGGAATGAGAACAATTTAAATCCCTTATCGAGTACCCATTAGAGGGCAAGTCTGGTGCCAGCAGCCGCGGTAATTCCAGCTATAATGRCGTATATTAATGTTGCTGCA--GTTAAAAAGCTYGTAGTTGGATTTTGGAGTTTGACAGACGGTCTGCCAATTGGTGTGTACTGTCT-GTTGGCTCTTCTTCTCTGGGAACCG-CRCTGCTCTTTATTGA-GGGGTGTTGGAACGCAGATT-TTTACTTTGAAAAAATTAGAGTGTTCAAAGCAGGCCAACGCATT--GAATACATTAGCATGGAATAATGGAATAGGACTTTGGTCTTATTTTGTTGGTTTCTAAGGCCGAGGTAACGAGTAATAGGGATAGTTG------------------------------------------------------------------------------------------GGGGCGTTTATATTCC-GTTGTCAGAGGTGAAATTCTTGGATTTACGGAAGATAAACTACTGCGAAAGCATTCGCCAAGGATGTTTTCATTAATCAAGAACGAAAGTTAGGGGATCGAAGACGATCAGATACCGTCGTAGTCTTAACCATAAACCATGCCGACTAGGGATCAGCAGACGTTCTTTAGCGACTCTGTTGGCACCTTGTGAGAAATCAAAGTTTTTGGGTTCCGGGGGGAGTATGGTCGCAAGGC-TGAAACTTAAAGGAATTGACGGAAGGGCACCACCAGGAGTGGAGCCTGCGGCTTAATTTGA-CTCAACACGGGGAAACTTACCAGGTCCAGACATAGTAAGGATTGACAGATTGAGAGCTCTTTCTTGATTCTATGGGTGGTGGTGCATGGCCGTTCTTAGTTGGTGGAGTGATTTGTCTGGTTAATTCCGTTAACGAACGAGACCTTAACCTACTAAATAGTTACACAAACATTTTG-TTTGTGGACAACTTCTTAGAGGGACTATTTCGTGCTAGTGAATGGAAGTTTGAGGCAATAACAGGTCTGTGATGCCCTTAGATGTTCTGGGCCGCACGCGCGCTACASTGATGAAAGCAACAAGTTTATAWC**B**T**GCA**T**M**GAAA**G**G**TG**TGGGTAATCTTGC-AARTTTCATCGTGATGGGGATAGA**TTA**TTGCAA**C**T**T**TT**AA**TCTTCAACGAGGAATTCCTAGTAAGCGTGAGTCATCAGCTCGCGTTGATTACGTCCCTGCCCTTTGTACACACCGCCCGTCGCTCCTACCGATTGAATG**A**TCCGGTGAAATCTTCGGACCGTGGCGATGTTGTTGGTTCGCCAGCAACGCTGTTGTGGGAAGTTGATTAAACCTTATCATTTAGACGAAGGAGAAGTTGT------------------------------------------------------------------------------------------------------------------------------------------------------------------------------------------------------------------------------------------------------------------------------------------------------------------------------------------------------------------------------------------------------------------------------------------------------------------------------------------------------------------------------------------------------------------------------------------------------------------------------------------------------------

>KY980342.1_Leucocryptos_marina_isolate_BH65_15_small_subunit_ribosomal_RNA_gene_partial_sequence

-----------------------------------------------------------------------------------------GAAACTGCGAATGGCTCATTAAATCAGTTATCGTTTATTTGATGATCTCTTATTACTTGGATAACCGTAGTAATTCTAGAGCTAATACATGCACACAATCCTGACTTCTGGAAGGGATGTATTTATTAGATACAAAACCAAT--GCGCTTCGGCGGTGATTGGTGATTCATAATAACTTTGCGAATCGTATGGCCTTGCGCTGACGATGAATCATTCAAATTTCTGCCCTATCAACTTTCGATGGTAGGATAGAGGCCTACCATGGTGGTAACGGGTAACGGAGAATTAGGGTTCGATTCCGGAGAGGGAGCCTGAGAGACGGCTACCACATCCAAGGAAGGCAGCAGGCGCGCAAATTACCCAATCCTGACTCAGGGAGGTAGTGACAATAAATAACAATACAGGGCCTTTTTAGGTCTTGTAATTGGAATGAGAACAATTTAAATCCCTTATCGAGGATCCATTAGAGGGCAAGTCTGGTGCCAGCAGCCGCGGTAATTCCAGCTCTAATAGCGTATATTAATGTTGTTGCA--GTTAAAAAGCTCGTAGTTGGATTTTGGTGTTTGACAAATGGTCTGCCTTCGGGTTTGCACTGTTT-GTTGGCACTTCTTTTCTGGGGAACT-CACTGCTCTTAATTGA-GGGGTGCTGTAACGCAGATCGTTTACTTTGAAAAAATTAGAGTGTTCAAAGCAGGCCAACGCATT--GAATACATTAGCATGGAATAATGGAATAGGACTTTGGTCTTATTTTGTTGGTTTCTAAGGCCGAGGTAATGATTAATAGGGATAGTTG------------------------------------------------------------------------------------------GGGGCGTTTATATTCC-GTTGTCAGAGGTGAAATTCTTGGATTTACGGAAGATAAACTACTGCGAAAGCATTCGCCAAGGATGTTTTCATTAATCAAGAACGAAAGTTAGGGGATCGAAGACGATCAGATACCGTCGTAGTCTTAACCATAAACCATGCCGACTAGGGATCAGCAGACGTTCTTTAACGACTCTGTTGGCACCTTGTGAGAAATCAAAGTTTTTGGGTTCCGGGGGGAGTATGGTCGCAAGGC-TGAAACTTAAAGGAATTGACGGAAGGGCACCACCAGGAGTGGAGCCTGCGGCTTAATTTGA-CTCAACACGAGGAAACTTACCAGGTCCAGACATGAGAAGGATTGACAGATTGAGAGCTCTTTCTTGATTCTATGGGTGGTGGTGCATGGCCGTTCTTAGTTGGTGGAGTGATTTGTCTGGTTAATTCCGTTAACGAACGAGACCTTAACCTACTAAATAGTTACCTTCACAATTTG-TGGGGGGCCAACTTCTTAGAGGGACTATTCGTGCCTAGCGAGTGGAAGTTTGAGGCAATAACAGGTCTGTGATGCCCTTAGATGTTCTGGGCCGTACGCGCGCTACACTGATGAAAGCAACAAGTCTATAACCTTGGT**C**GAAA**G**GCCTGGGTAATCTTGC-AAATTTCATCGTGATGGGGATAGA**TTA**TTGCAA**C**TATT**AA**TCTTCAACGAGGAATTCCTAGTAAGCGTGAGTCATCAGCTCGCGTTGATTACGTCCCTGCCCTTTGTACACACCGCCCGTCGCTCCTACCGATTGAATGGTCCGGTGAATTCTTCGGATTGT-GCGCAGCAA-TGGTTCGCCTGCGCTGTGC--GCGAGAAGTTGAATAAACCTTATCATTTAGAGGAAGGAGAAGTCGTAACAAGGTAACC------------------------------------------------------------------------------------------------------------------------------------------------------------------------------------------------------------------------------------------------------------------------------------------------------------------------------------------------------------------------------------------------------------------------------------------------------------------------------------------------------------------------------------------------------------------------------------------------------------------------------------------------

>denovo21_1705-ALA21-ap_M04999:118:000000000-CRPWJ:1:1101:9854:4356_1:N:0:321

---------------------------------------------------------------------------------------------------------------------------------------------------------------------------------------------------------------------------------------------------------------------------------------------------------------------------------------------------------------------------------------------------------------------------------------------------------------------------------------------------------------------------------------------------------------------------------------------------------------------------------------------------------------------------------------------------------------------------------------------------------------------------------------------------------------------------------------------------------------------------------------------------------------------------------------------------------------------------------------------------------------------------------------------------------------------------------------------------------------------------------------------------------------------------------------------------------------------------------------------------------------------------------------------------------------------------------------------------------------------------------------------------------------------------------------------------------------------------------------------------------------------------------------------------------------------------------------------------------------------------------------------------------------------------------------TAATCTTGC-AAATTTCATCGTGATGGGGATAGA**TTA**TTGCAA**C**TATT**AA**TCTTCAACGAGGAATTCCTAGTAAGCGTGAGTCATCAGCTCGCGTTGATTACGTCCCTGCCCTTTGTACACACCGCCCGTCGCTCCTA----------------------------------------------------------------------------------------------------------------------------------------------------------------------------------------------------------------------------------------------------------------------------------------------------------------------------------------------------------------------------------------------------------------------------------------------------------------------------------------------------------------------------------------------------------------------------------------------------------------------------------------------------------------------------------------------------------------------------------------------------------------------------
