# Supplementary material for: Ecological impact of algal-preying protists aplanochytrids (Labyrinthulea) as a source of docosahexaenoic acid for copepods in marine environments
Source: ISME Commun. 2026 Jun 26;6(1):ycag178. doi: 10.1093/ismeco/ycag178 (PMC13685785; doi:10.1093/ismeco/ycag178)
Supplement: Supplementary file 2 [file supplementary_materials_ycag178.zip › Morimoto_ISME_C_3rdrev_supplemental_data.zip!/Morimoto_ISME_C_3rdrev_supplemental_data/Supplemental_Data_2/Standard Curve/Aplanochytrid/2110/Aplano2110AlineN=2.pdf]

# Experiment Results Report

## 230724Aplano2110AlineN=2

## Experiment Summary

| | |
|---|---|
| Experiment Name | : 230724Aplano2110AlineN=2 |
| Experiment Type | : Quantitation - Standard Curve |
| File Name | : 230724Aplano2110AlineN=2.eds |
| Run Started | : 2023 Jul 24 12:03:30 PM |
| Run Finished | : 2023 Jul 24 1:42:37 PM |
| Run Duration | : 99 minutes 7 seconds |
| Date Modified | : 2023 Jul 24 1:59:51 PM |
| User | : |
| Number of wells used | : 71 |
| Number of wells with results | : 71 |
| Instrument Name | : |
| Instrument Type | : Applied Biosystems StepOnePlus™ Instrument |
| Comments | : |

# Results Summary

| Sample | Target | Quantity (Mean) | Quantity (Std Dev) | C (Mean) | C (Std Dev) |
|---|---|---|---|---|---|
| A01 BTM saki | Aplano | 461.07 | 47.02 | 28.79 | 0.15 |
| A01 SCM saki | Aplano | 354.11 | 13.70 | 29.20 | 0.06 |
| A04 1000m saki | Aplano | 9.06 | 3.98 | 34.99 | 0.79 |
| A04 200m saki | Aplano | 39.99 | 26.20 | 32.81 | 1.08 |
| A04 3000m saki | Aplano | 10.01 | 7.30 | 34.95 | 1.25 |
| A04 SCM saki | Aplano | 8708.06 | 881.66 | 24.25 | 0.16 |
| A07 1000m saki | Aplano | 13.26 | 11.03 | 35.44 | 3.04 |
| A07 200m saki | Aplano | | | | |
| A07 3000m saki | Aplano | 5.51 | 3.72 | 35.84 | 1.14 |
| A07 SCM saki | Aplano | 1897.29 | 239.49 | 26.61 | 0.20 |
| A11 1000m saki | Aplano | 19.91 | 19.17 | 34.13 | 1.82 |
| A11 200m saki | Aplano | 4.85 | 1.45 | 35.89 | 0.49 |
| A11 3000m saki | Aplano | 16.18 | | 33.97 | |
| A11 SCM saki | Aplano | 225.12 | 55.00 | 29.93 | 0.40 |
| A21 1000m saki | Aplano | 4.77 | | 35.86 | |
| A21 10m saki | Aplano | 447.94 | 95.79 | 28.86 | 0.35 |
| A21 200m saki | Aplano | 14.89 | 14.02 | 34.54 | 1.40 |
| A21 3000m saki | Aplano | 180050416.00 | 31495374.00 | 8.88 | 0.27 |
| A21 SCM saki | Aplano | 882.05 | 49.79 | 27.79 | 0.09 |

# Plate Layout

| | 1 | 2 | 3 | 4 | 5 | 6 | 7 | 8 | 9 | 10 | 11 | 12 |
|---|---|---|---|---|---|---|---|---|---|---|---|---|
| A | ⚠1<br>S Aplano<br>2E6<br>FAM-NFQ-MGB<br>CT: 17.18 | ⚠1<br>S Aplano<br>2E6<br>FAM-NFQ-MGB<br>CT: 13.84 | A01 SCM saki<br>U Aplano<br>367.52<br>FAM-NFQ-MGB<br>CT: 29.14 | A01 SCM saki<br>U Aplano<br>354.65<br>FAM-NFQ-MGB<br>CT: 29.19 | A01 SCM saki<br>U Aplano<br>340.15<br>FAM-NFQ-MGB<br>CT: 29.26 | A07 SCM saki<br>U Aplano<br>1.63E3<br>FAM-NFQ-MGB<br>CT: 26.83 | A07 SCM saki<br>U Aplano<br>2.09E3<br>FAM-NFQ-MGB<br>CT: 26.45 | A07 SCM saki<br>U Aplano<br>1.97E3<br>FAM-NFQ-MGB<br>CT: 26.54 | A21 10m saki<br>U Aplano<br>519.57<br>FAM-NFQ-MGB<br>CT: 28.6 | A21 10m saki<br>U Aplano<br>339.13<br>FAM-NFQ-MGB<br>CT: 29.26 | A21 10m saki<br>U Aplano<br>485.11<br>FAM-NFQ-MGB<br>CT: 28.71 | |
| B | S Aplano<br>2E5<br>FAM-NFQ-MGB<br>CT: 19.63 | S Aplano<br>2E5<br>FAM-NFQ-MGB<br>CT: 19.71 | A01 BTM saki<br>U Aplano<br>445.88<br>FAM-NFQ-MGB<br>CT: 28.84 | A01 BTM saki<br>U Aplano<br>423.53<br>FAM-NFQ-MGB<br>CT: 28.92 | A01 BTM saki<br>U Aplano<br>513.81<br>FAM-NFQ-MGB<br>CT: 28.62 | ⚠1<br>A07 200m saki<br>U Aplano<br>FAM-NFQ-MGB<br>CT: Undetermined | ⚠1<br>A07 200m saki<br>U Aplano<br>FAM-NFQ-MGB<br>CT: Undetermined | A07 200m saki<br>U Aplano<br>FAM-NFQ-MGB<br>CT: Undetermined | A21 SCM saki<br>U Aplano<br>824.67<br>FAM-NFQ-MGB<br>CT: 27.89 | A21 SCM saki<br>U Aplano<br>913.86<br>FAM-NFQ-MGB<br>CT: 27.73 | A21 SCM saki<br>U Aplano<br>907.63<br>FAM-NFQ-MGB<br>CT: 27.74 | |
| C | S Aplano<br>2E4<br>FAM-NFQ-MGB<br>CT: 23.21 | S Aplano<br>2E4<br>FAM-NFQ-MGB<br>CT: 22.67 | A04 SCM saki<br>U Aplano<br>7.73E3<br>FAM-NFQ-MGB<br>CT: 24.43 | A04 SCM saki<br>U Aplano<br>8.96E3<br>FAM-NFQ-MGB<br>CT: 24.2 | A04 SCM saki<br>U Aplano<br>9.43E3<br>FAM-NFQ-MGB<br>CT: 24.12 | ⚠1<br>A07 1000m saki<br>U Aplano<br>18.07<br>FAM-NFQ-MGB<br>CT: 33.8 | ⚠1<br>A07 1000m saki<br>U Aplano<br>0.65<br>FAM-NFQ-MGB<br>CT: 38.95 | ⚠1<br>A07 1000m saki<br>U Aplano<br>21.07<br>FAM-NFQ-MGB<br>CT: 33.56 | ⚠1<br>A21 200m saki<br>U Aplano<br>8.11<br>FAM-NFQ-MGB<br>CT: 35.04 | ⚠1<br>A21 200m saki<br>U Aplano<br>5.55<br>FAM-NFQ-MGB<br>CT: 35.63 | ⚠1<br>A21 200m saki<br>U Aplano<br>31.01<br>FAM-NFQ-MGB<br>CT: 32.96 | |
| D | S Aplano<br>2E3<br>FAM-NFQ-MGB<br>CT: 26.86 | S Aplano<br>2E3<br>FAM-NFQ-MGB<br>CT: 26.74 | ⚠1<br>A04 200m saki<br>U Aplano<br>34.69<br>FAM-NFQ-MGB<br>CT: 32.79 | ⚠1<br>A04 200m saki<br>U Aplano<br>68.43<br>FAM-NFQ-MGB<br>CT: 31.74 | ⚠1<br>A04 200m saki<br>U Aplano<br>16.84<br>FAM-NFQ-MGB<br>CT: 33.91 | ⚠1<br>A07 3000m saki<br>U Aplano<br>2.88<br>FAM-NFQ-MGB<br>CT: 36.64 | ⚠1<br>A07 3000m saki<br>U Aplano<br>8.14<br>FAM-NFQ-MGB<br>CT: 35.03 | ⚠2<br>A07 3000m saki<br>U Aplano<br>FAM-NFQ-MGB<br>CT: Undetermined | ⚠2<br>A21 1000m saki<br>U Aplano<br>FAM-NFQ-MGB<br>CT: Undetermined | A21 1000m saki<br>U Aplano<br>4.77<br>FAM-NFQ-MGB<br>CT: 35.86 | ⚠1<br>A21 1000m saki<br>U Aplano<br>FAM-NFQ-MGB<br>CT: Undetermined | |
| E | S Aplano<br>200<br>FAM-NFQ-MGB<br>CT: 30.13 | S Aplano<br>200<br>FAM-NFQ-MGB<br>CT: 29.95 | ⚠1<br>A04 1000m saki<br>U Aplano<br>4.74<br>FAM-NFQ-MGB<br>CT: 35.87 | ⚠1<br>A04 1000m saki<br>U Aplano<br>9.86<br>FAM-NFQ-MGB<br>CT: 34.74 | ⚠1<br>A04 1000m saki<br>U Aplano<br>12.57<br>FAM-NFQ-MGB<br>CT: 34.36 | A11 SCM saki<br>U Aplano<br>271.88<br>FAM-NFQ-MGB<br>CT: 29.61 | A11 SCM saki<br>U Aplano<br>164.53<br>FAM-NFQ-MGB<br>CT: 30.38 | A11 SCM saki<br>U Aplano<br>238.96<br>FAM-NFQ-MGB<br>CT: 29.81 | ⚠1<br>A21 3000m saki<br>U Aplano<br>2.02E8<br>FAM-NFQ-MGB<br>CT: 8.68 | A21 3000m saki<br>U Aplano<br>1.58E8<br>FAM-NFQ-MGB<br>CT: 9.07 | ⚠1<br>A21 3000m saki<br>U Aplano<br>FAM-NFQ-MGB<br>CT: Undetermined | |
| F | S Aplano<br>20<br>FAM-NFQ-MGB<br>CT: 33.67 | S Aplano<br>20<br>FAM-NFQ-MGB<br>CT: 33.24 | ⚠1<br>A04 3000m saki<br>U Aplano<br>FAM-NFQ-MGB<br>CT: Undetermined | ⚠1<br>A04 3000m saki<br>U Aplano<br>4.85<br>FAM-NFQ-MGB<br>CT: 35.84 | ⚠1<br>A04 3000m saki<br>U Aplano<br>15.17<br>FAM-NFQ-MGB<br>CT: 34.07 | A11 200m saki<br>U Aplano<br>3.31<br>FAM-NFQ-MGB<br>CT: 36.43 | A11 200m saki<br>U Aplano<br>5.04<br>FAM-NFQ-MGB<br>CT: 35.78 | A11 200m saki<br>U Aplano<br>6.2<br>FAM-NFQ-MGB<br>CT: 35.46 | | | | |
| G | | | | | | ⚠1<br>A11 1000m saki<br>U Aplano<br>6.35<br>FAM-NFQ-MGB<br>CT: 35.42 | ⚠1<br>A11 1000m saki<br>U Aplano<br>33.47<br>FAM-NFQ-MGB<br>CT: 32.85 | ⚠2<br>A11 1000m saki<br>U Aplano<br>FAM-NFQ-MGB<br>CT: Undetermined | | | | |
| H | N Aplano<br>FAM-NFQ-MGB<br>CT: Undetermined | N Aplano<br>FAM-NFQ-MGB<br>CT: Undetermined | | | | ⚠1<br>A11 3000m saki<br>U Aplano<br>FAM-NFQ-MGB<br>CT: Undetermined | A11 3000m saki<br>U Aplano<br>FAM-NFQ-MGB<br>CT: Undetermined | A11 3000m saki<br>U Aplano<br>16.18<br>FAM-NFQ-MGB<br>CT: 33.97 | | | | |

# Standard Curves

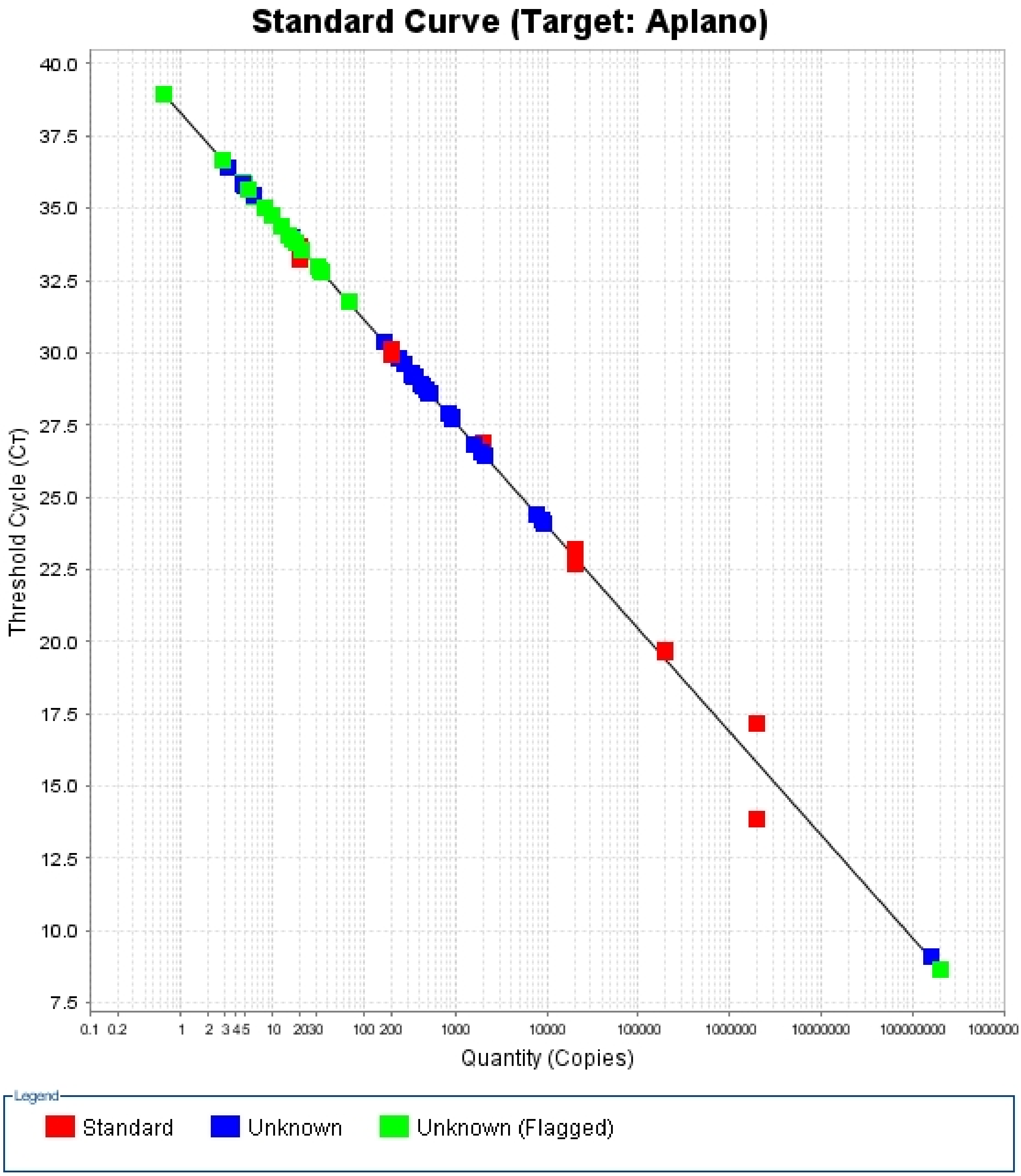


slope:-3.563 Y-Intercept:38.279 $R^2$:0.986 Eff%:90.837

# Results Table

| Well | | Sample | Target | Task | Quantity | C |
|---|---|---|---|---|---|---|
| A1 | | | Aplano | STANDARD | 2000000 | 17.1793 |
| A2 | | | Aplano | STANDARD | 2000000 | 13.8365 |
| A3 | | A01 SCM saki | Aplano | UNKNOWN | 367.523 | 29.1389 |
| A4 | | A01 SCM saki | Aplano | UNKNOWN | 354.6499 | 29.1941 |
| A5 | | A01 SCM saki | Aplano | UNKNOWN | 340.1472 | 29.2587 |
| A6 | | A07 SCM saki | Aplano | UNKNOWN | 1629.5629 | 26.8344 |
| A7 | | A07 SCM saki | Aplano | UNKNOWN | 2091.1323 | 26.4485 |
| A8 | | A07 SCM saki | Aplano | UNKNOWN | 1971.1873 | 26.5399 |
| A9 | | A21 10m saki | Aplano | UNKNOWN | 519.5685 | 28.6032 |
| A10 | | A21 10m saki | Aplano | UNKNOWN | 339.1337 | 29.2633 |
| A11 | | A21 10m saki | Aplano | UNKNOWN | 485.1114 | 28.7093 |
| B1 | | | Aplano | STANDARD | 200000 | 19.6348 |
| B2 | | | Aplano | STANDARD | 200000 | 19.7055 |
| B3 | | A01 BTM saki | Aplano | UNKNOWN | 445.8789 | 28.8398 |
| B4 | | A01 BTM saki | Aplano | UNKNOWN | 423.5339 | 28.9194 |
| B5 | | A01 BTM saki | Aplano | UNKNOWN | 513.808 | 28.6204 |
| B6 | | A07 200m saki | Aplano | UNKNOWN | | Undetermined |
| B7 | | A07 200m saki | Aplano | UNKNOWN | | Undetermined |
| B8 | | A07 200m saki | Aplano | UNKNOWN | | Undetermined |
| B9 | | A21 SCM saki | Aplano | UNKNOWN | 824.6727 | 27.8883 |
| B10 | | A21 SCM saki | Aplano | UNKNOWN | 913.8602 | 27.7294 |
| B11 | | A21 SCM saki | Aplano | UNKNOWN | 907.6264 | 27.74 |
| C1 | | | Aplano | STANDARD | 20000 | 23.2129 |
| C2 | | | Aplano | STANDARD | 20000 | 22.6653 |
| C3 | | A04 SCM saki | Aplano | UNKNOWN | 7727.2349 | 24.426 |
| C4 | | A04 SCM saki | Aplano | UNKNOWN | 8962.2559 | 24.1965 |
| C5 | | A04 SCM saki | Aplano | UNKNOWN | 9434.6963 | 24.117 |
| C6 | | A07 1000m saki | Aplano | UNKNOWN | 18.0743 | 33.8001 |
| C7 | | A07 1000m saki | Aplano | UNKNOWN | 0.6486 | 38.9489 |
| C8 | | A07 1000m saki | Aplano | UNKNOWN | 21.0681 | 33.5629 |

| Well | Sample | Target | Task | Quantity | C |
|---|---|---|---|---|---|
| C9 | A21 200m saki | Aplano | UNKNOWN | 8.1075 | 35.0406 |
| C10 | A21 200m saki | Aplano | UNKNOWN | 5.5534 | 35.6261 |
| C11 | A21 200m saki | Aplano | UNKNOWN | 31.0125 | 32.9647 |
| D1 | | Aplano | STANDARD | 2000 | 26.865 |
| D2 | | Aplano | STANDARD | 2000 | 26.7407 |
| D3 | A04 200m saki | Aplano | UNKNOWN | 34.6949 | 32.791 |
| D4 | A04 200m saki | Aplano | UNKNOWN | 68.4323 | 31.74 |
| D5 | A04 200m saki | Aplano | UNKNOWN | 16.8419 | 33.9094 |
| D6 | A07 3000m saki | Aplano | UNKNOWN | 2.875 | 36.6448 |
| D7 | A07 3000m saki | Aplano | UNKNOWN | 8.1409 | 35.0343 |
| D8 | A07 3000m saki | Aplano | UNKNOWN | | Undetermined |
| D9 | A21 1000m saki | Aplano | UNKNOWN | | Undetermined |
| D10 | A21 1000m saki | Aplano | UNKNOWN | 4.7674 | 35.8623 |
| D11 | A21 1000m saki | Aplano | UNKNOWN | | Undetermined |
| E1 | | Aplano | STANDARD | 200 | 30.1317 |
| E2 | | Aplano | STANDARD | 200 | 29.9503 |
| E3 | A04 1000m saki | Aplano | UNKNOWN | 4.7426 | 35.8704 |
| E4 | A04 1000m saki | Aplano | UNKNOWN | 9.8637 | 34.7372 |
| E5 | A04 1000m saki | Aplano | UNKNOWN | 12.5703 | 34.362 |
| E6 | A11 SCM saki | Aplano | UNKNOWN | 271.8843 | 29.6053 |
| E7 | A11 SCM saki | Aplano | UNKNOWN | 164.5274 | 30.3825 |
| E8 | A11 SCM saki | Aplano | UNKNOWN | 238.9595 | 29.805 |
| E9 | A21 3000m saki | Aplano | UNKNOWN | 202321008 | 8.6846 |
| E10 | A21 3000m saki | Aplano | UNKNOWN | 157779824 | 9.0693 |
| E11 | A21 3000m saki | Aplano | UNKNOWN | | Undetermined |
| F1 | | Aplano | STANDARD | 20 | 33.6675 |
| F2 | | Aplano | STANDARD | 20 | 33.2403 |
| F3 | A04 3000m saki | Aplano | UNKNOWN | | Undetermined |
| F4 | A04 3000m saki | Aplano | UNKNOWN | 4.8463 | 35.8369 |
| F5 | A04 3000m saki | Aplano | UNKNOWN | 15.1707 | 34.0711 |
| F6 | A11 200m saki | Aplano | UNKNOWN | 3.3124 | 36.4257 |
| F7 | A11 200m saki | Aplano | UNKNOWN | 5.0413 | 35.7759 |

| Well | | Sample | Target | Task | Quantity | C |
|---|---|---|---|---|---|---|
| F8 | | A11 200m saki | Aplano | UNKNOWN | 6.2028 | 35.455 |
| G6 | | A11 1000m saki | Aplano | UNKNOWN | 6.355 | 35.4175 |
| G7 | | A11 1000m saki | Aplano | UNKNOWN | 33.4656 | 32.8469 |
| G8 | | A11 1000m saki | Aplano | UNKNOWN | | Undetermined |
| H1 | | | Aplano | NTC | | Undetermined |
| H2 | | | Aplano | NTC | | Undetermined |
| H6 | | A11 3000m saki | Aplano | UNKNOWN | | Undetermined |
| H7 | | A11 3000m saki | Aplano | UNKNOWN | | Undetermined |
| H8 | | A11 3000m saki | Aplano | UNKNOWN | 16.1763 | 33.9718 |

# QC Summary

| | | | | | |
|---|---|---|---|---|---|
| Total Wells | 96 | Processed Wells | 71 | Targets Used | 1 |
| Well Setup | 71 | Flagged Wells | 30 | Samples Used | 19 |

| Flag | Name | Frequency | Locations |
|---|---|---|---|
| AMPNC | Amplification in negative control | 0 | |
| BADROX | Bad passive reference signal | 0 | |
| BLFAIL | Baseline algorithm failed | 0 | |
| CTFAIL | C algorithm failed | 0 | |
| EXPFAIL | Exponential algorithm failed | 9 | B6, B7, D8, D9, D11, E11, F3, G8, H6 |
| HIGHSD | High standard deviation in replicate group | 20 | A1, A2, C6, C7, C8, C9, C10, C11, D3, D4, D5, D6, D7, E3, E4, E5, F4, F5, G6, G7 |
| NOAMP | No amplification | 4 | D8, D9, E9, G8 |
| NOISE | Noise higher than others in plate | 0 | |
| NOSIGNAL | No signal in well | 0 | |
| OFFSCALE | Fluorescence is offscale | 0 | |
| OUTLIERRG | Outlier in replicate group | 0 | |
| SPIKE | Noise spikes | 0 | |
| THOLDFAIL | Thresholding algorithm failed | 0 | |
