# Supplementary material for: Ecological impact of algal-preying protists aplanochytrids (Labyrinthulea) as a source of docosahexaenoic acid for copepods in marine environments
Source: ISME Commun. 2026 Jun 26;6(1):ycag178. doi: 10.1093/ismeco/ycag178 (PMC13685785; doi:10.1093/ismeco/ycag178)
Supplement: Supplementary file 2 [file supplementary_materials_ycag178.zip › Morimoto_ISME_C_3rdrev_supplemental_data.zip!/Morimoto_ISME_C_3rdrev_supplemental_data/Supplemental_Data_2/Standard Curve/Aplanochytrid/2110/Aplano2110AlineN=3.pdf]

Experiment Results Report
230724Aplano2110AlineN=3

Experiment Summary

Experiment Name	:	230724Aplano2110AlineN=3
Experiment Type	:	Quantitation - Standard Curve
File Name	:	230724Aplano2110AlineN=3.eds
Run Started	:	2023 Jul 24 2:41:09 PM
Run Finished	:	2023 Jul 24 4:15:42 PM
Run Duration	:	94 minutes 32 seconds
Date Modified	:	2023 Jul 24 4:26:32 PM
User	:	
Number of wells used	:	71
Number of wells with results	:	71
Instrument Name	:	
Instrument Type	:	Applied Biosystems StepOnePlus™ Instrument
Comments	:	

Results Summary

Sample	Target	Quantity (Mean)	Quantity (Std Dev)	C (Mean)	C (Std Dev)
A01 BTM saki	Aplano	272.14	75.19	29.75	0.45
A01 SCM saki	Aplano	294.31	34.51	29.59	0.18
A04 1000m saki	Aplano	9.22		34.77	
A04 200m saki	Aplano	22.24	5.63	33.48	0.40
A04 3000m saki	Aplano	7.71	8.07	35.63	2.01
A04 SCM saki	Aplano	12471.92	2730.11	24.00	0.32
A07 1000m saki	Aplano	6.65	5.39	35.55	1.38
A07 200m saki	Aplano	7.66		35.05	
A07 3000m saki	Aplano	15.51	22.29	35.60	2.97
A07 SCM saki	Aplano	1036.53	103.01	27.71	0.15
A11 1000m saki	Aplano				
A11 200m saki	Aplano	0.37		39.60	
A11 3000m saki	Aplano				
A11 SCM saki	Aplano	284.65	34.64	29.64	0.19
A21 1000m saki	Aplano	6.99	5.00	35.40	1.18
A21 10m saki	Aplano	286.20	85.69	29.67	0.43
A21 200m saki	Aplano	62.18	55.34	32.31	1.34
A21 3000m saki	Aplano				
A21 SCM saki	Aplano	674.52	93.95	28.36	0.21

Plate Layout

	1	2	3	4	5	6	7	8	9	10	11	12
A	⚠1<br>S Aplano<br>2E6<br>FAM-NFQ-MGB<br>CT: 17.1	⚠1<br>S Aplano<br>2E6<br>FAM-NFQ-MGB<br>CT: 16.34	A01 SCM saki<br>U Aplano<br>324.39<br>FAM-NFQ-MGB<br>CT: 29.44	A01 SCM saki<br>U Aplano<br>256.63<br>FAM-NFQ-MGB<br>CT: 29.79	A01 SCM saki<br>U Aplano<br>301.9<br>FAM-NFQ-MGB<br>CT: 29.55	A07 SCM saki<br>U Aplano<br>919.71<br>FAM-NFQ-MGB<br>CT: 27.88	A07 SCM saki<br>U Aplano<br>1.11E3<br>FAM-NFQ-MGB<br>CT: 27.59	A07 SCM saki<br>U Aplano<br>1.08E3<br>FAM-NFQ-MGB<br>CT: 27.65	A21 10m saki<br>U Aplano<br>383.69<br>FAM-NFQ-MGB<br>CT: 29.19	A21 10m saki<br>U Aplano<br>252.05<br>FAM-NFQ-MGB<br>CT: 29.82	A21 10m saki<br>U Aplano<br>222.85<br>FAM-NFQ-MGB<br>CT: 30	
B	S Aplano<br>2E5<br>FAM-NFQ-MGB<br>CT: 19.41	S Aplano<br>2E5<br>FAM-NFQ-MGB<br>CT: 19.72	A01 BTM saki<br>U Aplano<br>188.42<br>FAM-NFQ-MGB<br>CT: 30.25	A01 BTM saki<br>U Aplano<br>294.07<br>FAM-NFQ-MGB<br>CT: 29.59	A01 BTM saki<br>U Aplano<br>333.92<br>FAM-NFQ-MGB<br>CT: 29.4	⚠1<br>A07 200m saki<br>U Aplano<br>FAM-NFQ-MGB<br>CT: Undetermined	A07 200m saki<br>U Aplano<br>7.66<br>FAM-NFQ-MGB<br>CT: 35.05	⚠1<br>A07 200m saki<br>U Aplano<br>FAM-NFQ-MGB<br>CT: Undetermined	A21 SCM saki<br>U Aplano<br>572.74<br>FAM-NFQ-MGB<br>CT: 28.59	A21 SCM saki<br>U Aplano<br>757.94<br>FAM-NFQ-MGB<br>CT: 28.17	A21 SCM saki<br>U Aplano<br>692.87<br>FAM-NFQ-MGB<br>CT: 28.31	
C	S Aplano<br>2E4<br>FAM-NFQ-MGB<br>CT: 23.31	S Aplano<br>2E4<br>FAM-NFQ-MGB<br>CT: 22.81	A04 SCM saki<br>U Aplano<br>1.02E4<br>FAM-NFQ-MGB<br>CT: 24.29	A04 SCM saki<br>U Aplano<br>1.18E4<br>FAM-NFQ-MGB<br>CT: 24.07	A04 SCM saki<br>U Aplano<br>1.55E4<br>FAM-NFQ-MGB<br>CT: 23.66	⚠1<br>A07 1000m saki<br>U Aplano<br>10.46<br>FAM-NFQ-MGB<br>CT: 34.58	⚠1<br>A07 1000m saki<br>U Aplano<br>2.84<br>FAM-NFQ-MGB<br>CT: 36.53	⚠2<br>A07 1000m saki<br>U Aplano<br>FAM-NFQ-MGB<br>CT: Undetermined	⚠1<br>A21 200m saki<br>U Aplano<br>39.79<br>FAM-NFQ-MGB<br>CT: 32.58	⚠1<br>A21 200m saki<br>U Aplano<br>125.21<br>FAM-NFQ-MGB<br>CT: 30.86	⚠1<br>A21 200m saki<br>U Aplano<br>21.55<br>FAM-NFQ-MGB<br>CT: 33.5	
D	S Aplano<br>2E3<br>FAM-NFQ-MGB<br>CT: 26.58	S Aplano<br>2E3<br>FAM-NFQ-MGB<br>CT: 26.78	A04 200m saki<br>U Aplano<br>27.35<br>FAM-NFQ-MGB<br>CT: 33.14	A04 200m saki<br>U Aplano<br>23.17<br>FAM-NFQ-MGB<br>CT: 33.39	A04 200m saki<br>U Aplano<br>16.21<br>FAM-NFQ-MGB<br>CT: 33.92	⚠1<br>A07 3000m saki<br>U Aplano<br>0.78<br>FAM-NFQ-MGB<br>CT: 38.46	⚠1<br>A07 3000m saki<br>U Aplano<br>41.16<br>FAM-NFQ-MGB<br>CT: 32.53	⚠1<br>A07 3000m saki<br>U Aplano<br>4.58<br>FAM-NFQ-MGB<br>CT: 35.81	⚠1<br>A21 1000m saki<br>U Aplano<br>3.45<br>FAM-NFQ-MGB<br>CT: 36.24	⚠1<br>A21 1000m saki<br>U Aplano<br>10.52<br>FAM-NFQ-MGB<br>CT: 34.57	⚠2<br>A21 1000m saki<br>U Aplano<br>FAM-NFQ-MGB<br>CT: Undetermined	
E	S Aplano<br>200<br>FAM-NFQ-MGB<br>CT: 29.95	S Aplano<br>200<br>FAM-NFQ-MGB<br>CT: 30.64	A04 1000m saki<br>U Aplano<br>9.22<br>FAM-NFQ-MGB<br>CT: 34.77	⚠1<br>A04 1000m saki<br>U Aplano<br>FAM-NFQ-MGB<br>CT: Undetermined	⚠2<br>A04 1000m saki<br>U Aplano<br>FAM-NFQ-MGB<br>CT: Undetermined	A11 SCM saki<br>U Aplano<br>246.02<br>FAM-NFQ-MGB<br>CT: 29.85	A11 SCM saki<br>U Aplano<br>294.98<br>FAM-NFQ-MGB<br>CT: 29.58	A11 SCM saki<br>U Aplano<br>312.96<br>FAM-NFQ-MGB<br>CT: 29.49	⚠1<br>A21 3000m saki<br>U Aplano<br>FAM-NFQ-MGB<br>CT: Undetermined	⚠2<br>A21 3000m saki<br>U Aplano<br>FAM-NFQ-MGB<br>CT: Undetermined	⚠2<br>A21 3000m saki<br>U Aplano<br>FAM-NFQ-MGB<br>CT: Undetermined	
F	S Aplano<br>20<br>FAM-NFQ-MGB<br>CT: 33.58	S Aplano<br>20<br>FAM-NFQ-MGB<br>CT: 33.75	⚠2<br>A04 3000m saki<br>U Aplano<br>FAM-NFQ-MGB<br>CT: Undetermined	⚠1<br>A04 3000m saki<br>U Aplano<br>13.42<br>FAM-NFQ-MGB<br>CT: 34.21	⚠1<br>A04 3000m saki<br>U Aplano<br>2<br>FAM-NFQ-MGB<br>CT: 37.05	A11 200m saki<br>U Aplano<br>0.37<br>FAM-NFQ-MGB<br>CT: 39.6	⚠1<br>A11 200m saki<br>U Aplano<br>FAM-NFQ-MGB<br>CT: Undetermined	⚠2<br>A11 200m saki<br>U Aplano<br>FAM-NFQ-MGB<br>CT: Undetermined				
G						⚠2<br>A11 1000m saki<br>U Aplano<br>FAM-NFQ-MGB<br>CT: Undetermined	⚠2<br>A11 1000m saki<br>U Aplano<br>FAM-NFQ-MGB<br>CT: Undetermined	⚠2<br>A11 1000m saki<br>U Aplano<br>FAM-NFQ-MGB<br>CT: Undetermined				
H	N Aplano<br>FAM-NFQ-MGB<br>CT: Undetermined	N Aplano<br>FAM-NFQ-MGB<br>CT: Undetermined				⚠1<br>A11 3000m saki<br>U Aplano<br>FAM-NFQ-MGB<br>CT: Undetermined	⚠1<br>A11 3000m saki<br>U Aplano<br>FAM-NFQ-MGB<br>CT: Undetermined	⚠1<br>A11 3000m saki<br>U Aplano<br>FAM-NFQ-MGB<br>CT: Undetermined				

Standard Curves

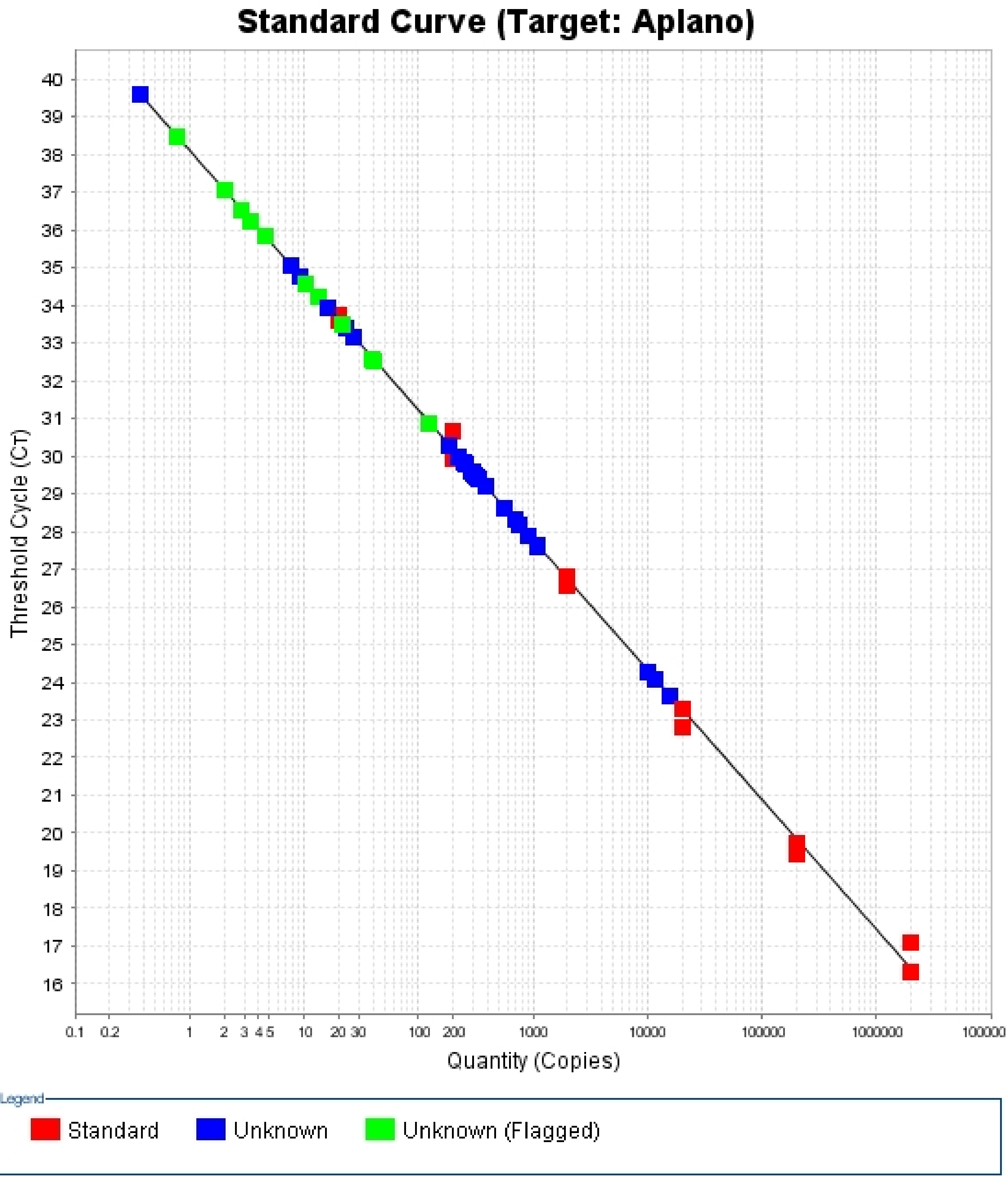

slope:-3.444 Y-Intercept:38.09 R^2:0.997 Eff%:95.127

Results Table

Well		Sample	Target	Task	Quantity	C
A1			Aplano	STANDARD	2000000	17.0959
A2			Aplano	STANDARD	2000000	16.3365
A3		A01 SCM saki	Aplano	UNKNOWN	324.3912	29.4408
A4		A01 SCM saki	Aplano	UNKNOWN	256.6321	29.7913
A5		A01 SCM saki	Aplano	UNKNOWN	301.8962	29.5483
A6		A07 SCM saki	Aplano	UNKNOWN	919.7064	27.8819
A7		A07 SCM saki	Aplano	UNKNOWN	1114.3372	27.5948
A8		A07 SCM saki	Aplano	UNKNOWN	1075.5455	27.6478
A9		A21 10m saki	Aplano	UNKNOWN	383.6932	29.1897
A10		A21 10m saki	Aplano	UNKNOWN	252.0513	29.8183
A11		A21 10m saki	Aplano	UNKNOWN	222.8469	30.0025
B1			Aplano	STANDARD	200000	19.4144
B2			Aplano	STANDARD	200000	19.7171
B3		A01 BTM saki	Aplano	UNKNOWN	188.4162	30.2536
B4		A01 BTM saki	Aplano	UNKNOWN	294.0703	29.5876
B5		A01 BTM saki	Aplano	UNKNOWN	333.919	29.3975
B6		A07 200m saki	Aplano	UNKNOWN		Undetermined
B7		A07 200m saki	Aplano	UNKNOWN	7.6569	35.0451
B8		A07 200m saki	Aplano	UNKNOWN		Undetermined
B9		A21 SCM saki	Aplano	UNKNOWN	572.7397	28.5904
B10		A21 SCM saki	Aplano	UNKNOWN	757.9375	28.1713
B11		A21 SCM saki	Aplano	UNKNOWN	692.8694	28.3056
C1			Aplano	STANDARD	20000	23.3088
C2			Aplano	STANDARD	20000	22.807
C3		A04 SCM saki	Aplano	UNKNOWN	10155.5098	24.2891
C4		A04 SCM saki	Aplano	UNKNOWN	11778.3291	24.0674
C5		A04 SCM saki	Aplano	UNKNOWN	15481.9238	23.6583
C6		A07 1000m saki	Aplano	UNKNOWN	10.4604	34.5784
C7		A07 1000m saki	Aplano	UNKNOWN	2.8415	36.5279
C8		A07 1000m saki	Aplano	UNKNOWN		Undetermined

Well		Sample	Target	Task	Quantity	C
C9		A21 200m saki	Aplano	UNKNOWN	39.7907	32.5798
C10		A21 200m saki	Aplano	UNKNOWN	125.2147	30.8648
C11		A21 200m saki	Aplano	UNKNOWN	21.5475	33.4973
D1			Aplano	STANDARD	2000	26.5825
D2			Aplano	STANDARD	2000	26.7832
D3		A04 200m saki	Aplano	UNKNOWN	27.353	33.1404
D4		A04 200m saki	Aplano	UNKNOWN	23.1664	33.3889
D5		A04 200m saki	Aplano	UNKNOWN	16.2128	33.9228
D6		A07 3000m saki	Aplano	UNKNOWN	0.7811	38.4597
D7		A07 3000m saki	Aplano	UNKNOWN	41.1557	32.5293
D8		A07 3000m saki	Aplano	UNKNOWN	4.5807	35.8136
D9		A21 1000m saki	Aplano	UNKNOWN	3.448	36.2385
D10		A21 1000m saki	Aplano	UNKNOWN	10.5228	34.5695
D11		A21 1000m saki	Aplano	UNKNOWN		Undetermined
E1			Aplano	STANDARD	200	29.9505
E2			Aplano	STANDARD	200	30.6433
E3		A04 1000m saki	Aplano	UNKNOWN	9.2181	34.7675
E4		A04 1000m saki	Aplano	UNKNOWN		Undetermined
E5		A04 1000m saki	Aplano	UNKNOWN		Undetermined
E6		A11 SCM saki	Aplano	UNKNOWN	246.0179	29.8545
E7		A11 SCM saki	Aplano	UNKNOWN	294.9769	29.583
E8		A11 SCM saki	Aplano	UNKNOWN	312.955	29.4945
E9		A21 3000m saki	Aplano	UNKNOWN		Undetermined
E10		A21 3000m saki	Aplano	UNKNOWN		Undetermined
E11		A21 3000m saki	Aplano	UNKNOWN		Undetermined
F1			Aplano	STANDARD	20	33.5779
F2			Aplano	STANDARD	20	33.7511
F3		A04 3000m saki	Aplano	UNKNOWN		Undetermined
F4		A04 3000m saki	Aplano	UNKNOWN	13.416	34.2061
F5		A04 3000m saki	Aplano	UNKNOWN	1.9988	37.0542
F6		A11 200m saki	Aplano	UNKNOWN	0.365	39.5977
F7		A11 200m saki	Aplano	UNKNOWN		Undetermined

Well		Sample	Target	Task	Quantity	C
F8		A11 200m saki	Aplano	UNKNOWN		Undetermined
G6		A11 1000m saki	Aplano	UNKNOWN		Undetermined
G7		A11 1000m saki	Aplano	UNKNOWN		Undetermined
G8		A11 1000m saki	Aplano	UNKNOWN		Undetermined
H1			Aplano	NTC		Undetermined
H2			Aplano	NTC		Undetermined
H6		A11 3000m saki	Aplano	UNKNOWN		Undetermined
H7		A11 3000m saki	Aplano	UNKNOWN		Undetermined
H8		A11 3000m saki	Aplano	UNKNOWN		Undetermined

QC Summary

Total Wells	96	Processed Wells	71	Targets Used	1
Well Setup	71	Flagged Wells	32	Samples Used	19

Flag	Name	Frequency	Locations
AMPNC	Amplification in negative control	0	
BADROX	Bad passive reference signal	0	
BLFAIL	Baseline algorithm failed	0	
CTFAIL	C algorithm failed	0	
EXPFAIL	Exponential algorithm failed	18	B6, B8, C8, D11, E4, E5, E9, E10, E11, F3, F7, F8, G6, G7, G8, H6, H7, H8
HIGHSD	High standard deviation in replicate group	14	A1, A2, C6, C7, C9, C10, C11, D6, D7, D8, D9, D10, F4, F5
NOAMP	No amplification	10	C8, D11, E5, E10, E11, F3, F8, G6, G7, G8
NOISE	Noise higher than others in plate	0	
NOSIGNAL	No signal in well	0	
OFFSCALE	Fluorescence is offscale	0	
OUTLIERRG	Outlier in replicate group	0	
SPIKE	Noise spikes	0	
THOLDFAIL	Thresholding algorithm failed	0	
