# Supplementary material for: Ecological impact of algal-preying protists aplanochytrids (Labyrinthulea) as a source of docosahexaenoic acid for copepods in marine environments
Source: ISME Commun. 2026 Jun 26;6(1):ycag178. doi: 10.1093/ismeco/ycag178 (PMC13685785; doi:10.1093/ismeco/ycag178)
Supplement: Supplementary file 2 [file supplementary_materials_ycag178.zip › Morimoto_ISME_C_3rdrev_supplemental_data.zip!/Morimoto_ISME_C_3rdrev_supplemental_data/Supplemental_Data_2/Standard Curve/Aplanochytrid/2110/Aplano2210AlineN=1.pdf]

Experiment Results Report

230721Aplano2110AlineN=1

Experiment Summary

Experiment Name	:	230721Aplano2110AlineN=1
Experiment Type	:	Quantitation - Standard Curve
File Name	:	230721Aplano2110AlineN=1.eds
Run Started	:	2023 Jul 21 5:54:13 PM
Run Finished	:	2023 Jul 21 7:28:41 PM
Run Duration	:	94 minutes 27 seconds
Date Modified	:	2023 Jul 24 11:23:51 AM
User	:	
Number of wells used	:	71
Number of wells with results	:	69
Instrument Name	:	
Instrument Type	:	Applied Biosystems StepOnePlus™ Instrument
Comments	:	

Results Summary

Sample	Target	Quantity (Mean)	Quantity (Std Dev)	C (Mean)	C (Std Dev)
A01 BTM saki	Aplano	33.52	20.79	33.75	1.06
A01 SCM saki	Aplano	624.41	123.35	29.06	0.30
A04 1000m saki	Aplano	12.54	1.25	35.13	0.15
A04 200m saki	Aplano	251.78	82.65	30.52	0.58
A04 3000m saki	Aplano	67.08	26.75	32.60	0.61
A04 SCM saki	Aplano	12580.51	2043.26	24.38	0.25
A07 1000m saki	Aplano	4.90	5.62	37.43	2.49
A07 200m saki	Aplano	10.19	0.80	35.45	0.12
A07 3000m saki	Aplano	14.47	2.44	34.92	0.25
A07 SCM saki	Aplano	2720.44	835.17	26.80	0.45
A11 1000m saki	Aplano			35.65	1.05
A11 200m saki	Aplano	27.66	10.07	33.97	0.61
A11 3000m saki	Aplano	4.55	0.69	36.72	0.24
A11 SCM saki	Aplano	285.55	19.76	30.26	0.11
A21 1000m saki	Aplano	28.59	27.21	34.40	1.67
A21 10m saki	Aplano	556.06	64.03	29.23	0.18
A21 200m saki	Aplano	53.47	11.27	32.89	0.34
A21 3000m saki	Aplano	31.88	11.67	33.75	0.59
A21 SCM saki	Aplano	1417.53	268.72	27.78	0.28

Plate Layout

	1	2	3	4	5	6
A	S Aplano<br>2E6<br>FAM-NFQ-MGB<br>CT: 16.96	S Aplano<br>2E6<br>FAM-NFQ-MGB<br>CT: 16.72	A01 SCM saki<br>U Aplano<br>765.56<br>FAM-NFQ-MGB<br>CT: 28.73	A01 SCM saki<br>U Aplano<br>537.31<br>FAM-NFQ-MGB<br>CT: 29.28	A01 SCM saki<br>U Aplano<br>570.37<br>FAM-NFQ-MGB<br>CT: 29.18	A07 SCM saki<br>U Aplano<br>2.1E3<br>FAM-NFQ-MGB<br>CT: 27.15
B	S Aplano<br>2E5<br>FAM-NFQ-MGB<br>CT: 20	S Aplano<br>2E5<br>FAM-NFQ-MGB<br>CT: 20.21	A01 BTM saki<br>U Aplano<br>54.66<br>FAM-NFQ-MGB<br>CT: 32.8	A01 BTM saki<br>U Aplano<br>785.32<br>FAM-NFQ-MGB<br>CT: 28.69	A01 BTM saki<br>U Aplano<br>801.79<br>FAM-NFQ-MGB<br>CT: 28.65	A07 200m saki<br>U Aplano<br>9.39<br>FAM-NFQ-MGB<br>CT: 35.58
C	S Aplano<br>2E4<br>FAM-NFQ-MGB<br>CT: 23.52	S Aplano<br>2E4<br>FAM-NFQ-MGB<br>CT: 23.18	A04 SCM saki<br>U Aplano<br>1.25E4<br>FAM-NFQ-MGB<br>CT: 24.37	A04 SCM saki<br>U Aplano<br>1.06E4<br>FAM-NFQ-MGB<br>CT: 24.64	A04 SCM saki<br>U Aplano<br>1.47E4<br>FAM-NFQ-MGB<br>CT: 24.13	⚠ 2<br>A07 1000m saki<br>U Aplano<br>FAM-NFQ-MGB<br>CT: Undetermined
D	S Aplano<br>2E3<br>FAM-NFQ-MGB<br>CT: 26.62	S Aplano<br>2E3<br>FAM-NFQ-MGB<br>CT: 26.82	⚠ 2<br>A04 200m saki<br>U Aplano<br>156.35<br>FAM-NFQ-MGB<br>CT: 31.2	⚠ 1<br>A04 200m saki<br>U Aplano<br>300.42<br>FAM-NFQ-MGB<br>CT: 30.18	⚠ 1<br>A04 200m saki<br>U Aplano<br>298.58<br>FAM-NFQ-MGB<br>CT: 30.19	A07 3000m saki<br>U Aplano<br>12.65<br>FAM-NFQ-MGB<br>CT: 35.11
E	S Aplano<br>200<br>FAM-NFQ-MGB<br>CT: 30.54	S Aplano<br>200<br>FAM-NFQ-MGB<br>CT: 30.91	A04 1000m saki<br>U Aplano<br>12.34<br>FAM-NFQ-MGB<br>CT: 35.15	A04 1000m saki<br>U Aplano<br>11.4<br>FAM-NFQ-MGB<br>CT: 35.28	A04 1000m saki<br>U Aplano<br>13.87<br>FAM-NFQ-MGB<br>CT: 34.97	A11 SCM saki<br>U Aplano<br>263.99<br>FAM-NFQ-MGB<br>CT: 30.38
F	⚠ 1<br>S Aplano<br>20<br>FAM-NFQ-MGB<br>CT: 32.7	⚠ 1<br>S Aplano<br>20<br>FAM-NFQ-MGB<br>CT: 37.08	⚠ 1<br>A04 3000m saki<br>U Aplano<br>44.59<br>FAM-NFQ-MGB<br>CT: 33.15	⚠ 1<br>A04 3000m saki<br>U Aplano<br>96.67<br>FAM-NFQ-MGB<br>CT: 31.95	⚠ 1<br>A04 3000m saki<br>U Aplano<br>60<br>FAM-NFQ-MGB<br>CT: 32.69	⚠ 1<br>A11 200m saki<br>U Aplano<br>17.25<br>FAM-NFQ-MGB<br>CT: 34.63
G						⚠ 1<br>A11 1000m saki<br>U Aplano<br>FAM-NFQ-MGB<br>CT: 36.69
H	N Aplano<br>FAM-NFQ-MGB<br>CT: Undetermined	N Aplano<br>FAM-NFQ-MGB<br>CT: Undetermined				A11 3000m saki<br>U Aplano<br>5.18<br>FAM-NFQ-MGB<br>CT: 36.5

	7	8	9	10	11	12
A	A07 SCM saki<br>U Aplano<br>3.67E3<br>FAM-NFQ-MGB<br>CT: 26.28	A07 SCM saki<br>U Aplano<br>2.39E3<br>FAM-NFQ-MGB<br>CT: 26.96	A21 10m saki<br>U Aplano<br>611.67<br>FAM-NFQ-MGB<br>CT: 29.07	A21 10m saki<br>U Aplano<br>570.46<br>FAM-NFQ-MGB<br>CT: 29.18	A21 10m saki<br>U Aplano<br>486.06<br>FAM-NFQ-MGB<br>CT: 29.43	
B	A07 200m saki<br>U Aplano<br>10.99<br>FAM-NFQ-MGB<br>CT: 35.33	A07 200m saki<br>U Aplano<br>4.66<br>FAM-NFQ-MGB<br>CT: 36.67	A21 SCM saki<br>U Aplano<br>1.26E3<br>FAM-NFQ-MGB<br>CT: 27.95	⚠ 1<br>A21 SCM saki<br>U Aplano<br>1.73E3<br>FAM-NFQ-MGB<br>CT: 27.46	A21 SCM saki<br>U Aplano<br>1.27E3<br>FAM-NFQ-MGB<br>CT: 27.94	
C	⚠ 1<br>A07 1000m saki<br>U Aplano<br>0.93<br>FAM-NFQ-MGB<br>CT: 39.19	⚠ 1<br>A07 1000m saki<br>U Aplano<br>8.88<br>FAM-NFQ-MGB<br>CT: 35.67	A21 200m saki<br>U Aplano<br>54.82<br>FAM-NFQ-MGB<br>CT: 32.83	A21 200m saki<br>U Aplano<br>64<br>FAM-NFQ-MGB<br>CT: 32.59	A21 200m saki<br>U Aplano<br>41.58<br>FAM-NFQ-MGB<br>CT: 33.26	
D	A07 3000m saki<br>U Aplano<br>13.51<br>FAM-NFQ-MGB<br>CT: 35.01	A07 3000m saki<br>U Aplano<br>17.25<br>FAM-NFQ-MGB<br>CT: 34.63	⚠ 1<br>A21 1000m saki<br>U Aplano<br>59.12<br>FAM-NFQ-MGB<br>CT: 32.71	⚠ 1<br>A21 1000m saki<br>U Aplano<br>19.78<br>FAM-NFQ-MGB<br>CT: 34.42	⚠ 1<br>A21 1000m saki<br>U Aplano<br>6.88<br>FAM-NFQ-MGB<br>CT: 36.06	
E	A11 SCM saki<br>U Aplano<br>302.82<br>FAM-NFQ-MGB<br>CT: 30.17	A11 SCM saki<br>U Aplano<br>289.84<br>FAM-NFQ-MGB<br>CT: 30.24	⚠ 1<br>A21 3000m saki<br>U Aplano<br>31.21<br>FAM-NFQ-MGB<br>CT: 33.71	⚠ 1<br>A21 3000m saki<br>U Aplano<br>43.86<br>FAM-NFQ-MGB<br>CT: 33.18	⚠ 1<br>A21 3000m saki<br>U Aplano<br>20.55<br>FAM-NFQ-MGB<br>CT: 34.36	
F	⚠ 1<br>A11 200m saki<br>U Aplano<br>FAM-NFQ-MGB<br>CT: 33.86	⚠ 1<br>A11 200m saki<br>U Aplano<br>FAM-NFQ-MGB<br>CT: 33.43				
G	⚠ 1<br>A11 1000m saki<br>U Aplano<br>8.8<br>FAM-NFQ-MGB<br>CT: 35.68	⚠ 1<br>A11 1000m saki<br>U Aplano<br>17.66<br>FAM-NFQ-MGB<br>CT: 34.59				
H	A11 3000m saki<br>U Aplano<br>FAM-NFQ-MGB<br>CT: Undetermined	A11 3000m saki<br>U Aplano<br>FAM-NFQ-MGB<br>CT: Undetermined				

Standard Curves

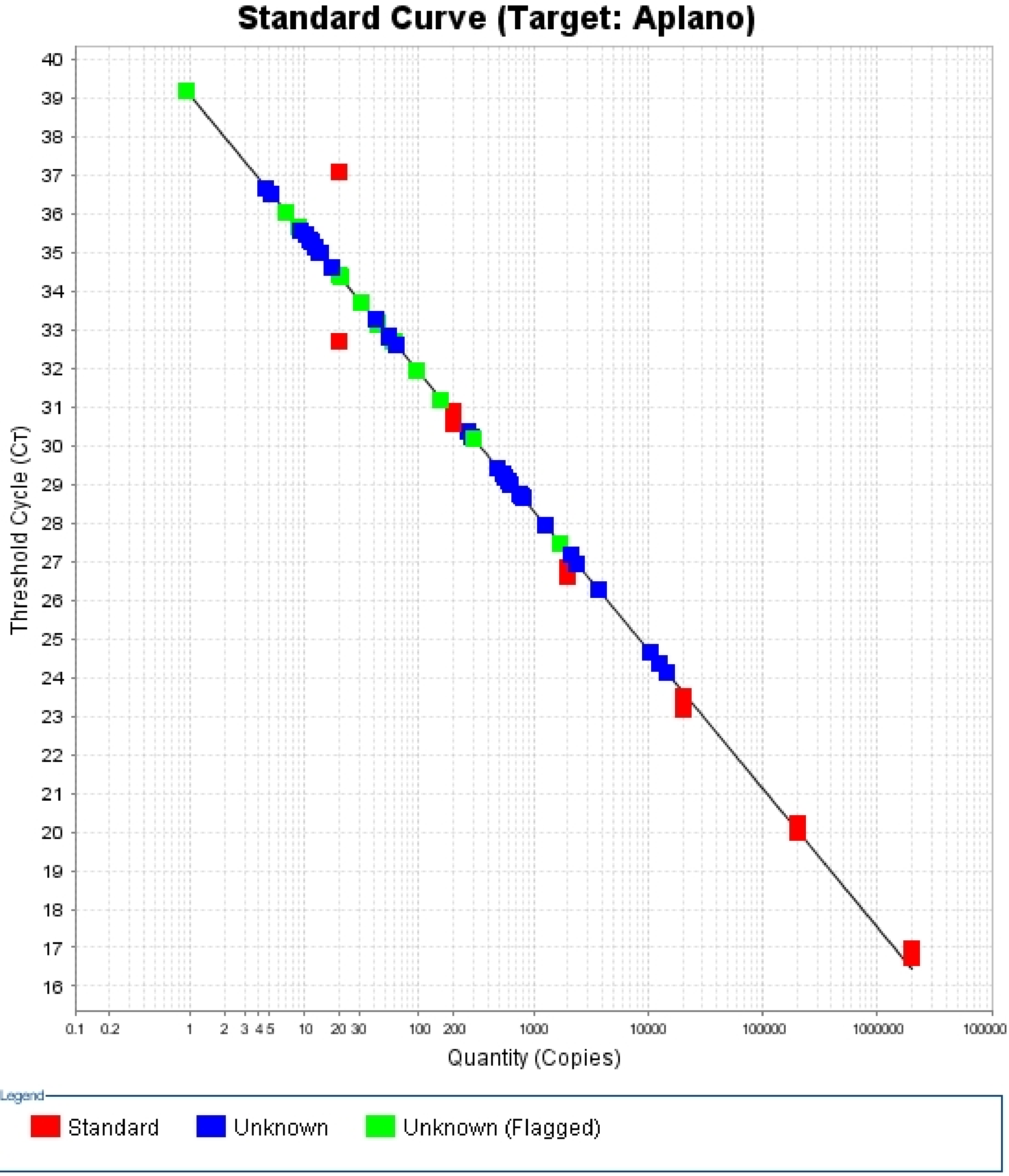

slope:-3.585 Y-Intercept:39.065 R^2:0.975 Eff%:90.078

Results Table

Well		Sample	Target	Task	Quantity	C
A1			Aplano	STANDARD	2000000	16.9572
A2			Aplano	STANDARD	2000000	16.7232
A3		A01 SCM saki	Aplano	UNKNOWN	765.5559	28.7255
A4		A01 SCM saki	Aplano	UNKNOWN	537.312	29.2767
A5		A01 SCM saki	Aplano	UNKNOWN	570.3715	29.1837
A6		A07 SCM saki	Aplano	UNKNOWN	2104.782	27.1508
A7		A07 SCM saki	Aplano	UNKNOWN	3671.1003	26.2846
A8		A07 SCM saki	Aplano	UNKNOWN	2385.4487	26.9559
A9		A21 10m saki	Aplano	UNKNOWN	611.6653	29.0749
A10		A21 10m saki	Aplano	UNKNOWN	570.4561	29.1835
A11		A21 10m saki	Aplano	UNKNOWN	486.0645	29.4327
B1			Aplano	STANDARD	200000	19.995
B2			Aplano	STANDARD	200000	20.2109
B3		A01 BTM saki	Aplano	UNKNOWN	54.6558	32.8042
B4		A01 BTM saki	Aplano	UNKNOWN	785.3249	28.6858
B5		A01 BTM saki	Aplano	UNKNOWN	801.7862	28.6535
B6		A07 200m saki	Aplano	UNKNOWN	9.3879	35.5781
B7		A07 200m saki	Aplano	UNKNOWN	10.9899	35.3327
B8		A07 200m saki	Aplano	UNKNOWN	4.6596	36.6687
B9		A21 SCM saki	Aplano	UNKNOWN	1258.8647	27.9511
B10		A21 SCM saki	Aplano	UNKNOWN	1727.7926	27.4581
B11		A21 SCM saki	Aplano	UNKNOWN	1265.9336	27.9423
C1			Aplano	STANDARD	20000	23.5207
C2			Aplano	STANDARD	20000	23.1758
C3		A04 SCM saki	Aplano	UNKNOWN	12522.4033	24.3742
C4		A04 SCM saki	Aplano	UNKNOWN	10566.9219	24.6385
C5		A04 SCM saki	Aplano	UNKNOWN	14652.2021	24.1296
C6		A07 1000m saki	Aplano	UNKNOWN		Undetermined
C7		A07 1000m saki	Aplano	UNKNOWN	0.9256	39.1852
C8		A07 1000m saki	Aplano	UNKNOWN	8.8756	35.6654

Well		Sample	Target	Task	Quantity	C
C9		A21 200m saki	Aplano	UNKNOWN	54.8195	32.8305
C10		A21 200m saki	Aplano	UNKNOWN	63.9966	32.5895
C11		A21 200m saki	Aplano	UNKNOWN	41.5809	33.2609
D1			Aplano	STANDARD	2000	26.6173
D2			Aplano	STANDARD	2000	26.8228
D3		A04 200m saki	Aplano	UNKNOWN	156.3548	31.1987
D4		A04 200m saki	Aplano	UNKNOWN	300.4185	30.1819
D5		A04 200m saki	Aplano	UNKNOWN	298.5811	30.1915
D6		A07 3000m saki	Aplano	UNKNOWN	12.6496	35.1137
D7		A07 3000m saki	Aplano	UNKNOWN	13.5104	35.0112
D8		A07 3000m saki	Aplano	UNKNOWN	17.2462	34.6311
D9		A21 1000m saki	Aplano	UNKNOWN	59.1187	32.713
D10		A21 1000m saki	Aplano	UNKNOWN	19.7779	34.4179
D11		A21 1000m saki	Aplano	UNKNOWN	6.8849	36.0608
E1			Aplano	STANDARD	200	30.5408
E2			Aplano	STANDARD	200	30.9073
E3		A04 1000m saki	Aplano	UNKNOWN	12.3419	35.1521
E4		A04 1000m saki	Aplano	UNKNOWN	11.3953	35.2763
E5		A04 1000m saki	Aplano	UNKNOWN	13.8693	34.9704
E6		A11 SCM saki	Aplano	UNKNOWN	263.9919	30.3832
E7		A11 SCM saki	Aplano	UNKNOWN	302.8158	30.1695
E8		A11 SCM saki	Aplano	UNKNOWN	289.8396	30.2377
E9		A21 3000m saki	Aplano	UNKNOWN	31.2111	33.7076
E10		A21 3000m saki	Aplano	UNKNOWN	43.8632	33.1777
E11		A21 3000m saki	Aplano	UNKNOWN	20.5512	34.3581
F1			Aplano	STANDARD	20	32.6965
F2			Aplano	STANDARD	20	37.0823
F3		A04 3000m saki	Aplano	UNKNOWN	44.5864	33.1522
F4		A04 3000m saki	Aplano	UNKNOWN	96.6656	31.9474
F5		A04 3000m saki	Aplano	UNKNOWN	59.9994	32.69
F6		A11 200m saki	Aplano	UNKNOWN	17.2488	34.6309
F7		A11 200m saki	Aplano	UNKNOWN		33.8554

Well		Sample	Target	Task	Quantity	C
F8		A11 200m saki	Aplano	UNKNOWN		33.4277
G6		A11 1000m saki	Aplano	UNKNOWN		36.691
G7		A11 1000m saki	Aplano	UNKNOWN	8.8048	35.6779
G8		A11 1000m saki	Aplano	UNKNOWN	17.6615	34.5941
H1			Aplano	NTC		Undetermined
H2			Aplano	NTC		Undetermined
H6		A11 3000m saki	Aplano	UNKNOWN	5.1769	36.5048
H7		A11 3000m saki	Aplano	UNKNOWN		
H8		A11 3000m saki	Aplano	UNKNOWN		

QC Summary

Total Wells	96	Processed Wells	71	Targets Used	1
Well Setup	71	Flagged Wells	24	Samples Used	19

Flag	Name	Frequency	Locations
AMPNC	Amplification in negative control	0	
BADROX	Bad passive reference signal	0	
BLFAIL	Baseline algorithm failed	0	
CTFAIL	C algorithm failed	0	
EXPFAIL	Exponential algorithm failed	1	C6
HIGHSD	High standard deviation in replicate group	22	C7, C8, D3, D4, D5, D9, D10, D11, E9, E10, E11, F1, F2, F3, F4, F5, F6, F7, F8, G6, G7, G8
NOAMP	No amplification	1	C6
NOISE	Noise higher than others in plate	0	
NOSIGNAL	No signal in well	0	
OFFSCALE	Fluorescence is offscale	0	
OUTLIERRG	Outlier in replicate group	2	B10, D3
SPIKE	Noise spikes	0	
THOLDFAIL	Thresholding algorithm failed	0	
