# Supplementary material for: Ecological impact of algal-preying protists aplanochytrids (Labyrinthulea) as a source of docosahexaenoic acid for copepods in marine environments
Source: ISME Commun. 2026 Jun 26;6(1):ycag178. doi: 10.1093/ismeco/ycag178 (PMC13685785; doi:10.1093/ismeco/ycag178)
Supplement: Supplementary file 2 [file supplementary_materials_ycag178.zip › Morimoto_ISME_C_3rdrev_supplemental_data.zip!/Morimoto_ISME_C_3rdrev_supplemental_data/Supplemental_Data_2/Standard Curve/Aplanochytrid/2110/Aplano2110A15N=1,2,3.pdf]

Experiment Results Report

230724Aplano2110AlineA15N=1,2,3

Experiment Summary

Experiment Name	:	230724Aplano2110AlineA15N=1,2,3
Experiment Type	:	Quantitation - Standard Curve
File Name	:	230724Aplano2110AlineA15N=1,2,3.eds
Run Started	:	2023 Jul 24 5:00:54 PM
Run Finished	:	2023 Jul 24 6:35:23 PM
Run Duration	:	94 minutes 28 seconds
Date Modified	:	2023 Jul 24 6:45:16 PM
User	:	
Number of wells used	:	50
Number of wells with results	:	50
Instrument Name	:	
Instrument Type	:	Applied Biosystems StepOnePlus™ Instrument
Comments	:	

Results Summary

Sample	Target	Quantity (Mean)	Quantity (Std Dev)	C (Mean)	C (Std Dev)
A15 1000m saki1	Aplano	15.51	10.16	35.27	1.53
A15 1000m saki2	Aplano	12.83	1.66	35.17	0.20
A15 1000m saki3	Aplano	4930964.50	6973396.00	24.21	13.76
A15 200m saki1	Aplano	46.24	20.18	33.30	0.65
A15 200m saki2	Aplano	48.41	32.70	33.42	1.21
A15 200m saki3	Aplano	26.31	10.73	34.15	0.61
A15 3000 saki1	Aplano	1642643.75		17.21	
A15 3000m saki2	Aplano				
A15 3000m saki3	Aplano				
A15 SCM saki1	Aplano	7729.94	2143.67	25.43	0.43
A15 SCM saki2	Aplano	4692.15	778.69	26.17	0.24
A15 SCM saki3	Aplano	7429.91	501.28	25.46	0.10

Plate Layout

	1	2	3	4	5	6	7	8	9	10	11	12
A	S Aplano<br>2E6<br>FAM-NFQ-MGB<br>CT: 17.34	S Aplano<br>2E6<br>FAM-NFQ-MGB<br>CT: 16.94	A15 SCM saki1<br>U Aplano<br>5.64E3<br>FAM-NFQ-MGB<br>CT: 25.87	A15 SCM saki1<br>U Aplano<br>9.93E3<br>FAM-NFQ-MGB<br>CT: 25.01	A15 SCM saki1<br>U Aplano<br>7.62E3<br>FAM-NFQ-MGB<br>CT: 25.42	A15 SCM saki2<br>U Aplano<br>4.26E3<br>FAM-NFQ-MGB<br>CT: 26.3	A15 SCM saki2<br>U Aplano<br>5.59E3<br>FAM-NFQ-MGB<br>CT: 25.89	A15 SCM saki2<br>U Aplano<br>4.22E3<br>FAM-NFQ-MGB<br>CT: 26.32	A15 SCM saki3<br>U Aplano<br>7.93E3<br>FAM-NFQ-MGB<br>CT: 25.35	A15 SCM saki3<br>U Aplano<br>6.93E3<br>FAM-NFQ-MGB<br>CT: 25.56	A15 SCM saki3<br>U Aplano<br>7.43E3<br>FAM-NFQ-MGB<br>CT: 25.45	
B	S Aplano<br>2E5<br>FAM-NFQ-MGB<br>CT: 20.45	S Aplano<br>2E5<br>FAM-NFQ-MGB<br>CT: 20.33	⚠ 1<br>A15 200m saki1<br>U Aplano<br>29.33<br>FAM-NFQ-MGB<br>CT: 33.9	⚠ 1<br>A15 200m saki1<br>U Aplano<br>68.58<br>FAM-NFQ-MGB<br>CT: 32.61	⚠ 1<br>A15 200m saki1<br>U Aplano<br>40.81<br>FAM-NFQ-MGB<br>CT: 33.4	⚠ 1<br>A15 200m saki2<br>U Aplano<br>45.56<br>FAM-NFQ-MGB<br>CT: 33.23	⚠ 1<br>A15 200m saki2<br>U Aplano<br>17.23<br>FAM-NFQ-MGB<br>CT: 34.72	⚠ 1<br>A15 200m saki2<br>U Aplano<br>82.44<br>FAM-NFQ-MGB<br>CT: 32.33	⚠ 1<br>A15 200m saki3<br>U Aplano<br>23.45<br>FAM-NFQ-MGB<br>CT: 34.24	⚠ 1<br>A15 200m saki3<br>U Aplano<br>17.3<br>FAM-NFQ-MGB<br>CT: 34.71	⚠ 1<br>A15 200m saki3<br>U Aplano<br>38.18<br>FAM-NFQ-MGB<br>CT: 33.5	
C	S Aplano<br>2E4<br>FAM-NFQ-MGB<br>CT: 23.8	S Aplano<br>2E4<br>FAM-NFQ-MGB<br>CT: 23.67	⚠ 1<br>A15 1000m saki1<br>U Aplano<br>21.59<br>FAM-NFQ-MGB<br>CT: 34.37	⚠ 2<br>A15 1000m saki1<br>U Aplano<br>3.78<br>FAM-NFQ-MGB<br>CT: 37.03	⚠ 1<br>A15 1000m saki1<br>U Aplano<br>21.16<br>FAM-NFQ-MGB<br>CT: 34.4	A15 1000m saki2<br>U Aplano<br>11.66<br>FAM-NFQ-MGB<br>CT: 35.31	A15 1000m saki2<br>U Aplano<br>14<br>FAM-NFQ-MGB<br>CT: 35.03	⚠ 2<br>A15 1000m saki2<br>U Aplano<br>FAM-NFQ-MGB<br>CT: Undetermined	⚠ 2<br>A15 1000m saki3<br>U Aplano<br>9.86E6<br>FAM-NFQ-MGB<br>CT: 14.48	⚠ 2<br>A15 1000m saki3<br>U Aplano<br>FAM-NFQ-MGB<br>CT: Undetermined	⚠ 1<br>A15 1000m saki3<br>U Aplano<br>28.57<br>FAM-NFQ-MGB<br>CT: 33.94	
D	S Aplano<br>2E3<br>FAM-NFQ-MGB<br>CT: 27.48	S Aplano<br>2E3<br>FAM-NFQ-MGB<br>CT: 27.38	⚠ 2<br>A15 3000 saki1<br>U Aplano<br>FAM-NFQ-MGB<br>CT: Undetermined	⚠ 1<br>A15 3000 saki1<br>U Aplano<br>1.64E6<br>FAM-NFQ-MGB<br>CT: 17.21	A15 3000 saki1<br>U Aplano<br>FAM-NFQ-MGB<br>CT: Undetermined	⚠ 1<br>A15 3000m saki2<br>U Aplano<br>FAM-NFQ-MGB<br>CT: Undetermined	⚠ 2<br>A15 3000m saki2<br>U Aplano<br>FAM-NFQ-MGB<br>CT: Undetermined	⚠ 2<br>A15 3000m saki2<br>U Aplano<br>FAM-NFQ-MGB<br>CT: Undetermined	⚠ 2<br>A15 3000m saki3<br>U Aplano<br>FAM-NFQ-MGB<br>CT: Undetermined	⚠ 2<br>A15 3000m saki3<br>U Aplano<br>FAM-NFQ-MGB<br>CT: Undetermined	⚠ 2<br>A15 3000m saki3<br>U Aplano<br>FAM-NFQ-MGB<br>CT: Undetermined	
E	S Aplano<br>200<br>FAM-NFQ-MGB<br>CT: 30.71	S Aplano<br>200<br>FAM-NFQ-MGB<br>CT: 30.58										
F	S Aplano<br>20<br>FAM-NFQ-MGB<br>CT: 34.63	S Aplano<br>20<br>FAM-NFQ-MGB<br>CT: 35.08										
G												
H	N Aplano<br>FAM-NFQ-MGB<br>CT: Undetermined	N Aplano<br>FAM-NFQ-MGB<br>CT: Undetermined										

Standard Curves

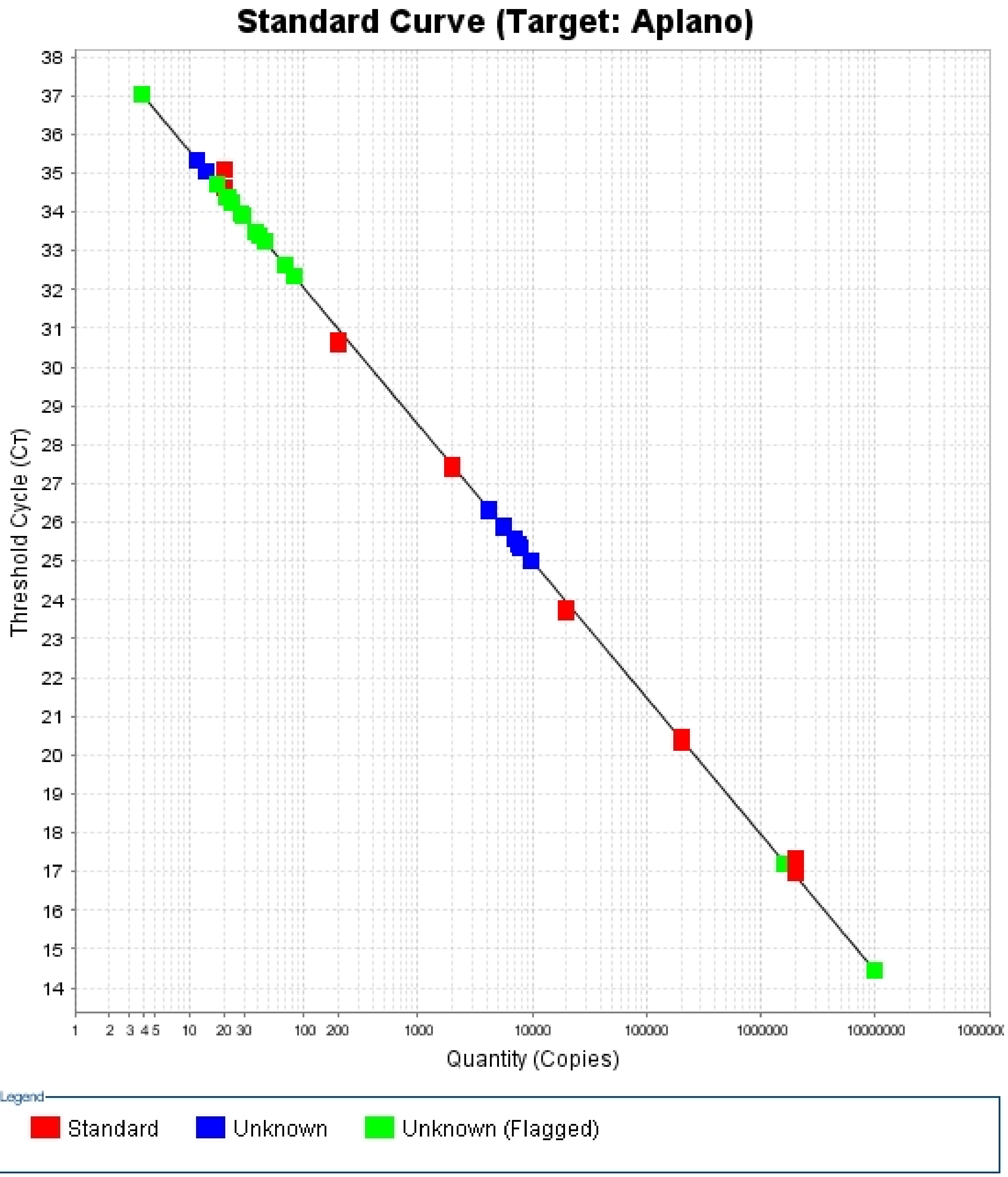

slope:-3.515 Y-Intercept:39.061 R^2:0.998 Eff%:92.528

Results Table

Well		Sample	Target	Task	Quantity	C
A1			Aplano	STANDARD	2000000	17.3422
A2			Aplano	STANDARD	2000000	16.9417
A3		A15 SCM saki1	Aplano	UNKNOWN	5644.501	25.8736
A4		A15 SCM saki1	Aplano	UNKNOWN	9927.4414	25.0117
A5		A15 SCM saki1	Aplano	UNKNOWN	7617.8628	25.416
A6		A15 SCM saki2	Aplano	UNKNOWN	4261.2266	26.3028
A7		A15 SCM saki2	Aplano	UNKNOWN	5591.0571	25.8882
A8		A15 SCM saki2	Aplano	UNKNOWN	4224.1772	26.3161
A9		A15 SCM saki3	Aplano	UNKNOWN	7930.4878	25.3546
A10		A15 SCM saki3	Aplano	UNKNOWN	6927.9297	25.5609
A11		A15 SCM saki3	Aplano	UNKNOWN	7431.3159	25.4538
B1			Aplano	STANDARD	200000	20.4541
B2			Aplano	STANDARD	200000	20.3271
B3		A15 200m saki1	Aplano	UNKNOWN	29.3341	33.9028
B4		A15 200m saki1	Aplano	UNKNOWN	68.5778	32.6064
B5		A15 200m saki1	Aplano	UNKNOWN	40.8141	33.3986
B6		A15 200m saki2	Aplano	UNKNOWN	45.5637	33.2306
B7		A15 200m saki2	Aplano	UNKNOWN	17.2269	34.7153
B8		A15 200m saki2	Aplano	UNKNOWN	82.4378	32.3254
B9		A15 200m saki3	Aplano	UNKNOWN	23.4464	34.2448
B10		A15 200m saki3	Aplano	UNKNOWN	17.295	34.7093
B11		A15 200m saki3	Aplano	UNKNOWN	38.1815	33.5004
C1			Aplano	STANDARD	20000	23.7991
C2			Aplano	STANDARD	20000	23.6709
C3		A15 1000m	Aplano	UNKNOWN	21.5945	34.3704
C4		A15 1000m	Aplano	UNKNOWN	3.7781	37.0315
C5		A15 1000m	Aplano	UNKNOWN	21.1566	34.4017
C6		A15 1000m	Aplano	UNKNOWN	11.6554	35.3118
C7		A15 1000m	Aplano	UNKNOWN	13.9963	35.0324
C8		A15 1000m	Aplano	UNKNOWN		Undetermined

Well		Sample	Target	Task	Quantity	C
C9		A15 1000m	Aplano	UNKNOWN	9861900	14.4768
C10		A15 1000m	Aplano	UNKNOWN		Undetermined
C11		A15 1000m	Aplano	UNKNOWN	28.5699	33.9431
D1			Aplano	STANDARD	2000	27.4754
D2			Aplano	STANDARD	2000	27.3804
D3		A15 3000 saki1	Aplano	UNKNOWN		Undetermined
D4		A15 3000 saki1	Aplano	UNKNOWN	1642643.75	17.213
D5		A15 3000 saki1	Aplano	UNKNOWN		Undetermined
D6		A15 3000m	Aplano	UNKNOWN		Undetermined
D7		A15 3000m	Aplano	UNKNOWN		Undetermined
D8		A15 3000m	Aplano	UNKNOWN		Undetermined
D9		A15 3000m	Aplano	UNKNOWN		Undetermined
D10		A15 3000m	Aplano	UNKNOWN		Undetermined
D11		A15 3000m	Aplano	UNKNOWN		Undetermined
E1			Aplano	STANDARD	200	30.7115
E2			Aplano	STANDARD	200	30.5842
F1			Aplano	STANDARD	20	34.6295
F2			Aplano	STANDARD	20	35.0799
H1			Aplano	NTC		Undetermined
H2			Aplano	NTC		Undetermined

QC Summary

Total Wells	96	Processed Wells	50	Targets Used	1
Well Setup	50	Flagged Wells	24	Samples Used	12

Flag	Name	Frequency	Locations
AMPNC	Amplification in negative control	0	
BADROX	Bad passive reference signal	0	
BLFAIL	Baseline algorithm failed	0	
CTFAIL	C algorithm failed	0	
EXPFAIL	Exponential algorithm failed	9	C8, C10, D3, D6, D7, D8, D9, D10, D11
HIGHSD	High standard deviation in replicate group	14	B3, B4, B5, B6, B7, B8, B9, B10, B11, C3, C4, C5, C9, C11
NOAMP	No amplification	10	C8, C9, C10, D3, D4, D7, D8, D9, D10, D11
NOISE	Noise higher than others in plate	0	
NOSIGNAL	No signal in well	0	
OFFSCALE	Fluorescence is offscale	0	
OUTLIERRG	Outlier in replicate group	1	C4
SPIKE	Noise spikes	0	
THOLDFAIL	Thresholding algorithm failed	0	
