# Supplementary material for: Ecological impact of algal-preying protists aplanochytrids (Labyrinthulea) as a source of docosahexaenoic acid for copepods in marine environments
Source: ISME Commun. 2026 Jun 26;6(1):ycag178. doi: 10.1093/ismeco/ycag178 (PMC13685785; doi:10.1093/ismeco/ycag178)
Supplement: Supplementary file 2 [file supplementary_materials_ycag178.zip › Morimoto_ISME_C_3rdrev_supplemental_data.zip!/Morimoto_ISME_C_3rdrev_supplemental_data/Supplemental_Data_2/Standard Curve/Aplanochytrid/2205/Aplano2205Aline_A04-A07_N=1_data.pdf]

Experiment Results Report
230309AplanoEmmAline2205A04-A07

Experiment Summary

Experiment Name	:	230309AplanoEmmAline2205A04-A07
Experiment Type	:	Quantitation - Standard Curve
File Name	:	230309AplanoEmmAline2205A04-A07.eds
Run Started	:	2023 Mar 10 3:17:17 PM
Run Finished	:	2023 Mar 10 4:52:04 PM
Run Duration	:	94 minutes 47 seconds
Date Modified	:	2023 Mar 10 5:08:56 PM
User	:	
Number of wells used	:	46
Number of wells with results	:	46
Instrument Name	:	
Instrument Type	:	Applied Biosystems StepOnePlus™ Instrument
Comments	:	

Results Summary

Sample	Target	Quantity (Mean)	Quantity (Std Dev)	C (Mean)	C (Std Dev)
2205 A04 1000m	Aplano				
2205 A04 10m saki	Aplano	3.53	1.26	34.82	0.58
2205 A04 200m saki	Aplano	0.75		37.05	
2205 A04 3000m	Aplano	1.53	1.06	36.19	1.12
2205 A04 SCM saki	Aplano	32.01	13.56	31.55	0.59
2205 A07 1000m	Aplano	18.84	8.21	32.38	0.78
2205 A07 10m saki	Aplano	1278.26	156.16	25.99	0.18
2205 A07 200m saki	Aplano	111.35	25.01	29.63	0.32
2205 A07 3000m	Aplano	7.29	2.03	33.71	0.43
2205 A07 SCM saki	Aplano	7815.67	933.36	23.29	0.17
Comf2E4	Aplano	20826.07	4830.08	21.85	0.35

Plate Layout

	1	2	3	4	5	6	7	8	9	10	11	12
A	S Aplano<br>2E6<br>FAM-NFQ-MGB<br>CT: 15.73	S Aplano<br>2E6<br>FAM-NFQ-MGB<br>CT: 15.2		⚠ 1<br>2205 A04 10m saki<br>U Aplano<br>4.66<br>FAM-NFQ-MGB<br>CT: 34.33	⚠ 1<br>2205 A04 10m saki<br>U Aplano<br>3.75<br>FAM-NFQ-MGB<br>CT: 34.66	⚠ 1<br>2205 A04 10m saki<br>U Aplano<br>2.17<br>FAM-NFQ-MGB<br>CT: 35.47		2205 A07 10m saki<br>U Aplano<br>1.43E3<br>FAM-NFQ-MGB<br>CT: 25.82	2205 A07 10m saki<br>U Aplano<br>1.29E3<br>FAM-NFQ-MGB<br>CT: 25.96	2205 A07 10m saki<br>U Aplano<br>1.12E3<br>FAM-NFQ-MGB<br>CT: 26.18		
B	S Aplano<br>2E5<br>FAM-NFQ-MGB<br>CT: 18.34	S Aplano<br>2E5<br>FAM-NFQ-MGB<br>CT: 18.24		⚠ 1<br>2205 A04 SCM saki<br>U Aplano<br>22.72<br>FAM-NFQ-MGB<br>CT: 31.98	⚠ 1<br>2205 A04 SCM saki<br>U Aplano<br>25.74<br>FAM-NFQ-MGB<br>CT: 31.79	⚠ 1<br>2205 A04 SCM saki<br>U Aplano<br>47.57<br>FAM-NFQ-MGB<br>CT: 30.88		2205 A07 SCM saki<br>U Aplano<br>7.27E3<br>FAM-NFQ-MGB<br>CT: 23.39	2205 A07 SCM saki<br>U Aplano<br>7.29E3<br>FAM-NFQ-MGB<br>CT: 23.39	2205 A07 SCM saki<br>U Aplano<br>8.89E3<br>FAM-NFQ-MGB<br>CT: 23.09		
C	S Aplano<br>2E4<br>FAM-NFQ-MGB<br>CT: 21.49	S Aplano<br>2E4<br>FAM-NFQ-MGB<br>CT: 21.55		⚠ 2<br>2205 A04 200m saki<br>U Aplano<br>FAM-NFQ-MGB<br>CT: Undetermined	⚠ 2<br>2205 A04 200m saki<br>U Aplano<br>FAM-NFQ-MGB<br>CT: Undetermined	2205 A04 200m saki<br>U Aplano<br>0.75<br>FAM-NFQ-MGB<br>CT: 37.05		2205 A07 200m saki<br>U Aplano<br>98.5<br>FAM-NFQ-MGB<br>CT: 29.79	2205 A07 200m saki<br>U Aplano<br>95.38<br>FAM-NFQ-MGB<br>CT: 29.84	2205 A07 200m saki<br>U Aplano<br>140.18<br>FAM-NFQ-MGB<br>CT: 29.27		
D	S Aplano<br>2E3<br>FAM-NFQ-MGB<br>CT: 25.15	S Aplano<br>2E3<br>FAM-NFQ-MGB<br>CT: 25.09		⚠ 1<br>2205 A04 1000m saki<br>U Aplano<br>FAM-NFQ-MGB<br>CT: Undetermined	⚠ 1<br>2205 A04 1000m saki<br>U Aplano<br>FAM-NFQ-MGB<br>CT: Undetermined	⚠ 2<br>2205 A04 1000m saki<br>U Aplano<br>FAM-NFQ-MGB<br>CT: Undetermined		⚠ 1<br>2205 A07 1000m saki<br>U Aplano<br>21.73<br>FAM-NFQ-MGB<br>CT: 32.04	⚠ 1<br>2205 A07 1000m saki<br>U Aplano<br>25.21<br>FAM-NFQ-MGB<br>CT: 31.82	⚠ 1<br>2205 A07 1000m saki<br>U Aplano<br>9.57<br>FAM-NFQ-MGB<br>CT: 33.26		
E	S Aplano<br>200<br>FAM-NFQ-MGB<br>CT: 28.61	S Aplano<br>200<br>FAM-NFQ-MGB<br>CT: 28.89		⚠ 1<br>2205 A04 3000m saki<br>U Aplano<br>0.79<br>FAM-NFQ-MGB<br>CT: 36.98	⚠ 1<br>2205 A04 3000m saki<br>U Aplano<br>2.28<br>FAM-NFQ-MGB<br>CT: 35.4	⚠ 1<br>2205 A04 3000m saki<br>U Aplano<br>FAM-NFQ-MGB<br>CT: Undetermined		2205 A07 3000m saki<br>U Aplano<br>5.28<br>FAM-NFQ-MGB<br>CT: 34.15	2205 A07 3000m saki<br>U Aplano<br>9.34<br>FAM-NFQ-MGB<br>CT: 33.3	2205 A07 3000m saki<br>U Aplano<br>7.26<br>FAM-NFQ-MGB<br>CT: 33.67		
F	S Aplano<br>20<br>FAM-NFQ-MGB<br>CT: 32.15	S Aplano<br>20<br>FAM-NFQ-MGB<br>CT: 32.75										
G												
H	Comf2E4<br>U Aplano<br>1.74E4<br>FAM-NFQ-MGB<br>CT: 22.09	Comf2E4<br>U Aplano<br>2.42E4<br>FAM-NFQ-MGB<br>CT: 21.6									N Aplano<br>FAM-NFQ-MGB<br>CT: Undetermined	N Aplano<br>FAM-NFQ-MGB<br>CT: Undetermined

Standard Curves

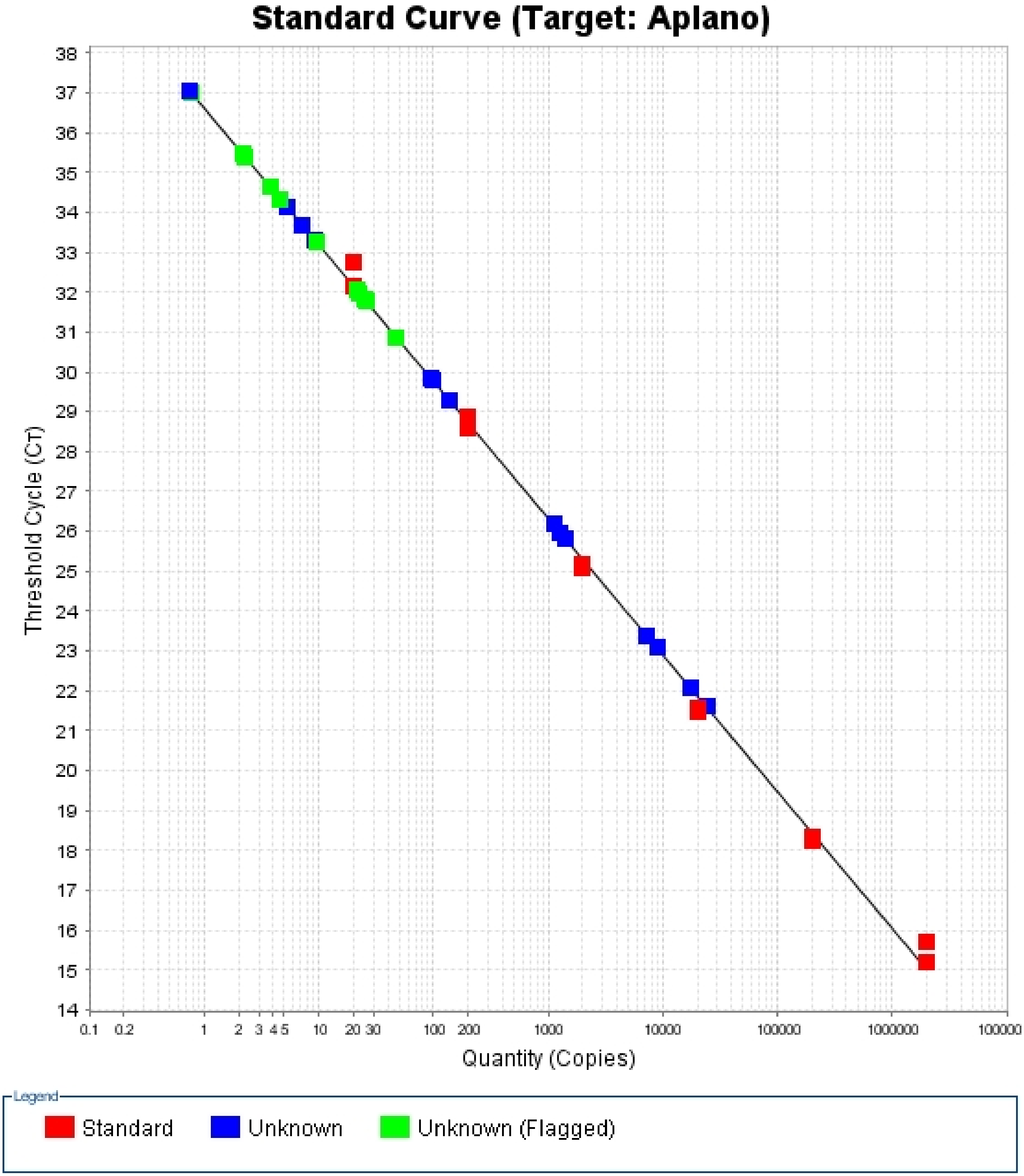

slope:-3.427 Y-Intercept:36.624 R^2:0.997 Eff%:95.814

Results Table

Well		Sample	Target	Task	Quantity	C
A1			Aplano	STANDARD	2000000	15.7253
A2			Aplano	STANDARD	2000000	15.2039
A4		2205 A04 10m	Aplano	UNKNOWN	4.6595	34.3336
A5		2205 A04 10m	Aplano	UNKNOWN	3.7486	34.6573
A6		2205 A04 10m	Aplano	UNKNOWN	2.1725	35.4691
A8		2205 A07 10m	Aplano	UNKNOWN	1426.9551	25.8151
A9		2205 A07 10m	Aplano	UNKNOWN	1292.2532	25.9627
A10		2205 A07 10m	Aplano	UNKNOWN	1115.5702	26.1815
B1			Aplano	STANDARD	200000	18.3364
B2			Aplano	STANDARD	200000	18.2388
B4		2205 A04 SCM	Aplano	UNKNOWN	22.7165	31.9762
B5		2205 A04 SCM	Aplano	UNKNOWN	25.7355	31.7905
B6		2205 A04 SCM	Aplano	UNKNOWN	47.5665	30.8764
B8		2205 A07 SCM	Aplano	UNKNOWN	7268.1904	23.3925
B9		2205 A07 SCM	Aplano	UNKNOWN	7285.4355	23.389
B10		2205 A07 SCM	Aplano	UNKNOWN	8893.3701	23.0922
C1			Aplano	STANDARD	20000	21.4871
C2			Aplano	STANDARD	20000	21.5534
C4		2205 A04 200m	Aplano	UNKNOWN		Undetermined
C5		2205 A04 200m	Aplano	UNKNOWN		Undetermined
C6		2205 A04 200m	Aplano	UNKNOWN	0.7515	37.0489
C8		2205 A07 200m	Aplano	UNKNOWN	98.5048	29.7931
C9		2205 A07 200m	Aplano	UNKNOWN	95.381	29.8411
C10		2205 A07 200m	Aplano	UNKNOWN	140.1786	29.2681
D1			Aplano	STANDARD	2000	25.1524
D2			Aplano	STANDARD	2000	25.0865
D4		2205 A04	Aplano	UNKNOWN		Undetermined
D5		2205 A04	Aplano	UNKNOWN		Undetermined
D6		2205 A04	Aplano	UNKNOWN		Undetermined
D8		2205 A07	Aplano	UNKNOWN	21.7304	32.0422

Well		Sample	Target	Task	Quantity	C
D9		2205 A07	Aplano	UNKNOWN	25.2078	31.8213
D10		2205 A07	Aplano	UNKNOWN	9.5708	33.2625
E1			Aplano	STANDARD	200	28.6106
E2			Aplano	STANDARD	200	28.8889
E4		2205 A04	Aplano	UNKNOWN	0.7858	36.9824
E5		2205 A04	Aplano	UNKNOWN	2.2803	35.3971
E6		2205 A04	Aplano	UNKNOWN		Undetermined
E8		2205 A07	Aplano	UNKNOWN	5.2757	34.1488
E9		2205 A07	Aplano	UNKNOWN	9.3449	33.298
E10		2205 A07	Aplano	UNKNOWN	7.2554	33.6746
F1			Aplano	STANDARD	20	32.1528
F2			Aplano	STANDARD	20	32.7542
H1		Comf2E4	Aplano	UNKNOWN	17410.6836	22.0925
H2		Comf2E4	Aplano	UNKNOWN	24241.4512	21.6
H11			Aplano	NTC		Undetermined
H12			Aplano	NTC		Undetermined

QC Summary

Total Wells	96	Processed Wells	46	Targets Used	1
Well Setup	46	Flagged Wells	17	Samples Used	11

Flag	Name	Frequency	Locations
AMPNC	Amplification in negative control	0	
BADROX	Bad passive reference signal	0	
BLFAIL	Baseline algorithm failed	0	
CTFAIL	C algorithm failed	0	
EXPFAIL	Exponential algorithm failed	6	C4, C5, D4, D5, D6, E6
HIGHSD	High standard deviation in replicate group	11	A4, A5, A6, B4, B5, B6, D8, D9, D10, E4, E5
NOAMP	No amplification	3	C4, C5, D6
NOISE	Noise higher than others in plate	0	
NOSIGNAL	No signal in well	0	
OFFSCALE	Fluorescence is offscale	0	
OUTLIERRG	Outlier in replicate group	0	
SPIKE	Noise spikes	0	
THOLDFAIL	Thresholding algorithm failed	0	
