# Supplementary material for: Ecological impact of algal-preying protists aplanochytrids (Labyrinthulea) as a source of docosahexaenoic acid for copepods in marine environments
Source: ISME Commun. 2026 Jun 26;6(1):ycag178. doi: 10.1093/ismeco/ycag178 (PMC13685785; doi:10.1093/ismeco/ycag178)
Supplement: Supplementary file 2 [file supplementary_materials_ycag178.zip › Morimoto_ISME_C_3rdrev_supplemental_data.zip!/Morimoto_ISME_C_3rdrev_supplemental_data/Supplemental_Data_2/Standard Curve/Aplanochytrid/2205/Aplano2205Aline_A04-A07_N=2,3_data.pdf]

Experiment Results Report

230324AplanoAline2205A0407DnaExtN=3EmmByKurabo

Experiment Summary

Experiment Name	: 230324AplanoAline2205A0407DnaExtN=3EmmByKurabo
Experiment Type	: Quantitation - Standard Curve
File Name	: 230324AplanoAline2205A0407DnaExtN=3EmmByKurabo.eds
Run Started	: 2023 Mar 24 2:34:30 PM
Run Finished	: 2023 Mar 24 4:09:11 PM
Run Duration	: 94 minutes 40 seconds
Date Modified	: 2023 Mar 24 4:44:18 PM
User	:
Number of wells used	: 76
Number of wells with results	: 76
Instrument Name	:
Instrument Type	: Applied Biosystems StepOnePlus™ Instrument
Comments	:

Results Summary

Sample	Target	Quantity (Mean)	Quantity (Std Dev)	C (Mean)	C (Std Dev)
2205 A04 1000m	Aplano				
2205 A04 1000m	Aplano				
2205 A04 10m saki2	Aplano	23.21	1.93	36.44	0.12
2205 A04 10m saki3	Aplano	72.98	13.86	34.76	0.27
2205 A04 200m	Aplano				
2205 A04 200m	Aplano	5.35	2.22	38.69	0.57
2205 A04 3000m	Aplano				
2205 A04 3000m	Aplano				
2205 A04 SCM saki2	Aplano	76.13	42.04	34.84	0.85
2205 A04 SCM saki3	Aplano	111.89	7.99	34.12	0.11
2205 A07 1000m	Aplano	50.82	15.76	35.32	0.43
2205 A07 1000m	Aplano	46.42	30.01	35.60	0.88
2205 A07 10m saki2	Aplano	1379.11	94.37	30.40	0.10
2205 A07 10m saki3	Aplano	1398.38	37.59	30.38	0.04
2205 A07 200m	Aplano	584.79	58.63	31.67	0.15
2205 A07 200m	Aplano	244.21	12.12	32.96	0.07
2205 A07 3000m	Aplano	3.01		39.46	
2205 A07 3000m	Aplano	23.92		36.40	
2205 A07 SCM saki2	Aplano	12595.76	1317.76	27.13	0.15
2205 A07 SCM saki3	Aplano	11229.37	565.51	27.29	0.08
Comf2E4	Aplano	19945.12	901.07	26.44	0.07

Plate Layout

	1	2	3	4	5	6	7	8	9	10	11	12
A	S Aplano<br>2E6<br>FAM-NFQ-MGB<br>CT: 19.8	S Aplano<br>2E6<br>FAM-NFQ-MGB<br>CT: 19.65	2205 A04 10m saki2<br>U Aplano<br>21.64<br>FAM-NFQ-MGB<br>CT: 36.54	2205 A04 10m saki2<br>U Aplano<br>25.36<br>FAM-NFQ-MGB<br>CT: 36.31	2205 A04 10m saki2<br>U Aplano<br>22.61<br>FAM-NFQ-MGB<br>CT: 36.48	⚠1<br>2205 A04 1000m saki3<br>U Aplano<br>FAM-NFQ-MGB<br>CT: Undetermined	2205 A07 10m saki2<br>U Aplano<br>1.42E3<br>FAM-NFQ-MGB<br>CT: 30.36	2205 A07 10m saki2<br>U Aplano<br>1.27E3<br>FAM-NFQ-MGB<br>CT: 30.52	2205 A07 10m saki2<br>U Aplano<br>1.45E3<br>FAM-NFQ-MGB<br>CT: 30.32			
B	S Aplano<br>2E5<br>FAM-NFQ-MGB<br>CT: 23.01	S Aplano<br>2E5<br>FAM-NFQ-MGB<br>CT: 22.86	⚠1<br>2205 A04 SCM saki2<br>U Aplano<br>121.63<br>FAM-NFQ-MGB<br>CT: 33.99	⚠1<br>2205 A04 SCM saki2<br>U Aplano<br>38.74<br>FAM-NFQ-MGB<br>CT: 35.68	⚠1<br>2205 A04 SCM saki2<br>U Aplano<br>68.01<br>FAM-NFQ-MGB<br>CT: 34.85	⚠1<br>2205 A04 1000m saki3<br>U Aplano<br>FAM-NFQ-MGB<br>CT: Undetermined	2205 A07 SCM saki2<br>U Aplano<br>1.16E4<br>FAM-NFQ-MGB<br>CT: 27.24	2205 A07 SCM saki2<br>U Aplano<br>1.2E4<br>FAM-NFQ-MGB<br>CT: 27.19	2205 A07 SCM saki2<br>U Aplano<br>1.41E4<br>FAM-NFQ-MGB<br>CT: 26.95			
C	S Aplano<br>2E4<br>FAM-NFQ-MGB<br>CT: 26.42	S Aplano<br>2E4<br>FAM-NFQ-MGB<br>CT: 26.24	⚠2<br>2205 A04 200m saki2<br>U Aplano<br>FAM-NFQ-MGB<br>CT: Undetermined	⚠2<br>2205 A04 200m saki2<br>U Aplano<br>FAM-NFQ-MGB<br>CT: Undetermined	⚠2<br>2205 A04 200m saki2<br>U Aplano<br>FAM-NFQ-MGB<br>CT: Undetermined	⚠2<br>2205 A04 1000m saki3<br>U Aplano<br>FAM-NFQ-MGB<br>CT: Undetermined	2205 A07 200m saki2<br>U Aplano<br>628.75<br>FAM-NFQ-MGB<br>CT: 31.56	2205 A07 200m saki2<br>U Aplano<br>518.22<br>FAM-NFQ-MGB<br>CT: 31.84	2205 A07 200m saki2<br>U Aplano<br>607.4<br>FAM-NFQ-MGB<br>CT: 31.61	⚠1<br>2205 A07 1000m saki3<br>U Aplano<br>31.38<br>FAM-NFQ-MGB<br>CT: 35.99		
D	S Aplano<br>2E3<br>FAM-NFQ-MGB<br>CT: 29.97	S Aplano<br>2E3<br>FAM-NFQ-MGB<br>CT: 29.88	⚠2<br>2205 A04 1000m saki2<br>U Aplano<br>FAM-NFQ-MGB<br>CT: Undetermined	⚠2<br>2205 A04 1000m saki2<br>U Aplano<br>FAM-NFQ-MGB<br>CT: Undetermined	2205 A04 1000m saki2<br>U Aplano<br>FAM-NFQ-MGB<br>CT: Undetermined	⚠2<br>2205 A04 3000m saki3<br>U Aplano<br>FAM-NFQ-MGB<br>CT: Undetermined	2205 A07 1000m saki2<br>U Aplano<br>68.96<br>FAM-NFQ-MGB<br>CT: 34.83	2205 A07 1000m saki2<br>U Aplano<br>43.07<br>FAM-NFQ-MGB<br>CT: 35.53	2205 A07 1000m saki2<br>U Aplano<br>40.44<br>FAM-NFQ-MGB<br>CT: 35.62	⚠1<br>2205 A07 1000m saki3<br>U Aplano<br>80.98<br>FAM-NFQ-MGB<br>CT: 34.59		
E	S Aplano<br>200<br>FAM-NFQ-MGB<br>CT: 33.37	S Aplano<br>200<br>FAM-NFQ-MGB<br>CT: 33.24	⚠1<br>2205 A04 3000m saki2<br>U Aplano<br>FAM-NFQ-MGB<br>CT: Undetermined	2205 A04 3000m saki2<br>U Aplano<br>FAM-NFQ-MGB<br>CT: Undetermined	⚠2<br>2205 A04 3000m saki2<br>U Aplano<br>FAM-NFQ-MGB<br>CT: Undetermined	⚠2<br>2205 A04 3000m saki3<br>U Aplano<br>FAM-NFQ-MGB<br>CT: Undetermined	⚠2<br>2205 A07 3000m saki2<br>U Aplano<br>FAM-NFQ-MGB<br>CT: Undetermined	⚠2<br>2205 A07 3000m saki2<br>U Aplano<br>FAM-NFQ-MGB<br>CT: Undetermined	2205 A07 3000m saki2<br>U Aplano<br>3.01<br>FAM-NFQ-MGB<br>CT: 39.46	⚠1<br>2205 A07 1000m saki3<br>U Aplano<br>26.89<br>FAM-NFQ-MGB<br>CT: 36.22		
F	S Aplano<br>20<br>FAM-NFQ-MGB<br>CT: 36.83	S Aplano<br>20<br>FAM-NFQ-MGB<br>CT: 36.44	2205 A04 10m saki3<br>U Aplano<br>67.4<br>FAM-NFQ-MGB<br>CT: 34.86	2205 A04 10m saki3<br>U Aplano<br>62.78<br>FAM-NFQ-MGB<br>CT: 34.97	2205 A04 10m saki3<br>U Aplano<br>88.76<br>FAM-NFQ-MGB<br>CT: 34.46	⚠2<br>2205 A04 3000m saki3<br>U Aplano<br>FAM-NFQ-MGB<br>CT: Undetermined	2205 A07 10m saki3<br>U Aplano<br>1.43E3<br>FAM-NFQ-MGB<br>CT: 30.34	2205 A07 10m saki3<br>U Aplano<br>1.4E3<br>FAM-NFQ-MGB<br>CT: 30.37	2205 A07 10m saki3<br>U Aplano<br>1.36E3<br>FAM-NFQ-MGB<br>CT: 30.42	⚠1<br>2205 A07 3000m saki3<br>U Aplano<br>FAM-NFQ-MGB<br>CT: Undetermined		
G			2205 A04 SCM saki3<br>U Aplano<br>113.94<br>FAM-NFQ-MGB<br>CT: 34.09	2205 A04 SCM saki3<br>U Aplano<br>118.65<br>FAM-NFQ-MGB<br>CT: 34.03	2205 A04 SCM saki3<br>U Aplano<br>103.07<br>FAM-NFQ-MGB<br>CT: 34.23		2205 A07 SCM saki3<br>U Aplano<br>1.06E4<br>FAM-NFQ-MGB<br>CT: 27.37	2205 A07 SCM saki3<br>U Aplano<br>1.17E4<br>FAM-NFQ-MGB<br>CT: 27.23	2205 A07 SCM saki3<br>U Aplano<br>1.13E4<br>FAM-NFQ-MGB<br>CT: 27.28	⚠2<br>2205 A07 3000m saki3<br>U Aplano<br>FAM-NFQ-MGB<br>CT: Undetermined		N Aplano<br>FAM-NFQ-MGB<br>CT: Undetermined
H	Comf2E4<br>U Aplano<br>1.93E4<br>FAM-NFQ-MGB<br>CT: 26.49	Comf2E4<br>U Aplano<br>2.06E4<br>FAM-NFQ-MGB<br>CT: 26.39	⚠1<br>2205 A04 200m saki3<br>U Aplano<br>4.13<br>FAM-NFQ-MGB<br>CT: 38.99	⚠1<br>2205 A04 200m saki3<br>U Aplano<br>7.91<br>FAM-NFQ-MGB<br>CT: 38.03	⚠1<br>2205 A04 200m saki3<br>U Aplano<br>4.01<br>FAM-NFQ-MGB<br>CT: 39.04		2205 A07 200m saki3<br>U Aplano<br>258.2<br>FAM-NFQ-MGB<br>CT: 32.88	2205 A07 200m saki3<br>U Aplano<br>237.38<br>FAM-NFQ-MGB<br>CT: 33	2205 A07 200m saki3<br>U Aplano<br>237.05<br>FAM-NFQ-MGB<br>CT: 33	2205 A07 3000m saki3<br>U Aplano<br>23.92<br>FAM-NFQ-MGB<br>CT: 36.4		N Aplano<br>FAM-NFQ-MGB<br>CT: Undetermined

Standard Curves

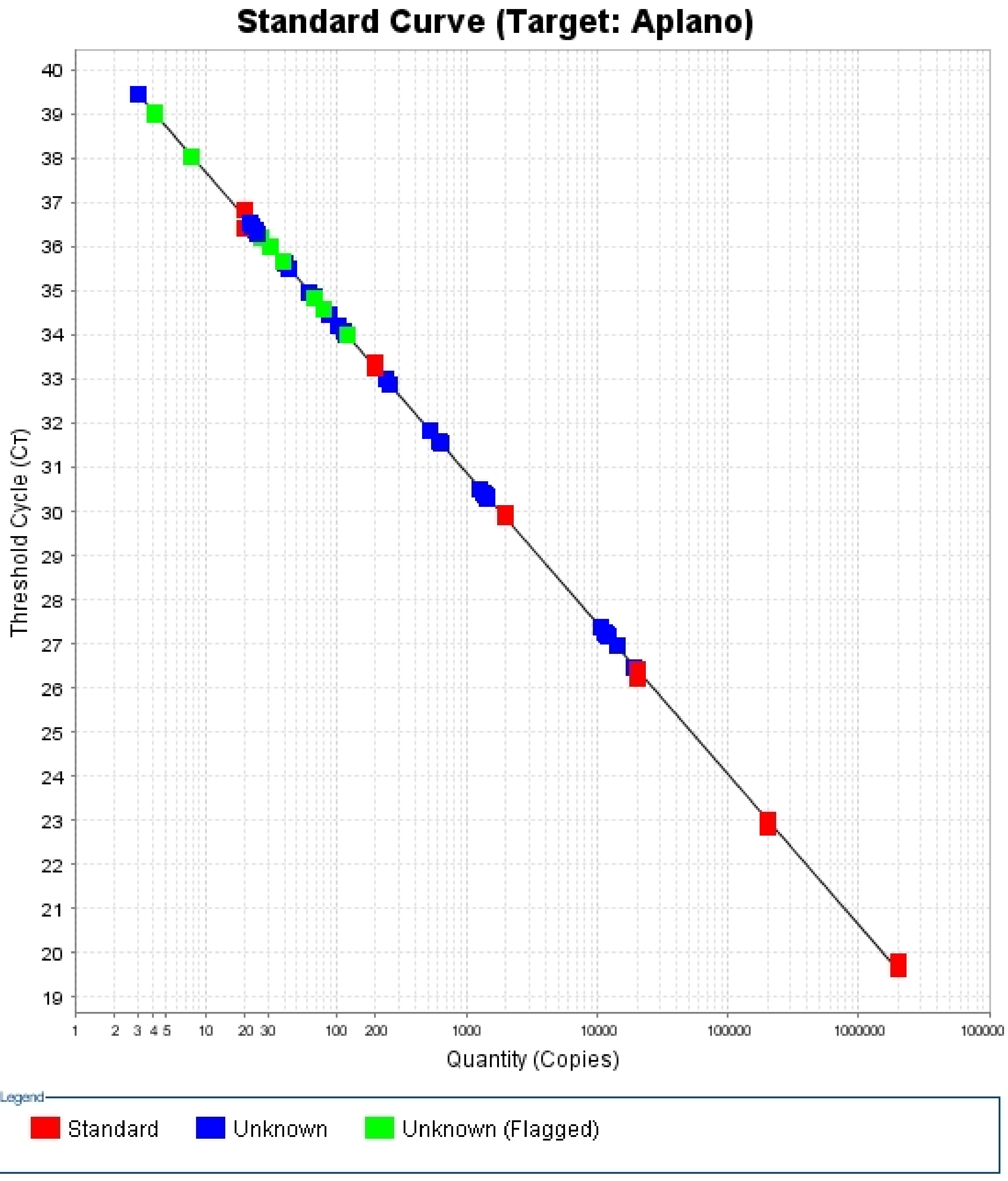

slope:-3.408 Y-Intercept:41.096 R^2:0.999 Eff%:96.521

Results Table

Well		Sample	Target	Task	Quantity	C
A1			Aplano	STANDARD	2000000	19.7965
A2			Aplano	STANDARD	2000000	19.6463
A3		2205 A04 10m	Aplano	UNKNOWN	21.6442	36.5445
A4		2205 A04 10m	Aplano	UNKNOWN	25.3621	36.3099
A5		2205 A04 10m	Aplano	UNKNOWN	22.6135	36.4796
A6		2205 A04	Aplano	UNKNOWN		Undetermined
A7		2205 A07 10m	Aplano	UNKNOWN	1416.1207	30.356
A8		2205 A07 10m	Aplano	UNKNOWN	1271.8494	30.5151
A9		2205 A07 10m	Aplano	UNKNOWN	1449.3739	30.3217
B1			Aplano	STANDARD	200000	23.0071
B2			Aplano	STANDARD	200000	22.8559
B3		2205 A04 SCM	Aplano	UNKNOWN	121.635	33.9893
B4		2205 A04 SCM	Aplano	UNKNOWN	38.7358	35.683
B5		2205 A04 SCM	Aplano	UNKNOWN	68.0056	34.8499
B6		2205 A04	Aplano	UNKNOWN		Undetermined
B7		2205 A07 SCM	Aplano	UNKNOWN	11649.3564	27.2368
B8		2205 A07 SCM	Aplano	UNKNOWN	12037.0957	27.1884
B9		2205 A07 SCM	Aplano	UNKNOWN	14100.8193	26.9541
C1			Aplano	STANDARD	20000	26.4163
C2			Aplano	STANDARD	20000	26.2366
C3		2205 A04 200m	Aplano	UNKNOWN		Undetermined
C4		2205 A04 200m	Aplano	UNKNOWN		Undetermined
C5		2205 A04 200m	Aplano	UNKNOWN		Undetermined
C6		2205 A04	Aplano	UNKNOWN		Undetermined
C7		2205 A07 200m	Aplano	UNKNOWN	628.7469	31.5578
C8		2205 A07 200m	Aplano	UNKNOWN	518.2169	31.844
C9		2205 A07 200m	Aplano	UNKNOWN	607.3977	31.609
C10		2205 A07	Aplano	UNKNOWN	31.3833	35.9946
D1			Aplano	STANDARD	2000	29.9709
D2			Aplano	STANDARD	2000	29.8815

Well		Sample	Target	Task	Quantity	C
D3		2205 A04	Aplano	UNKNOWN		Undetermined
D4		2205 A04	Aplano	UNKNOWN		Undetermined
D5		2205 A04	Aplano	UNKNOWN		Undetermined
D6		2205 A04	Aplano	UNKNOWN		Undetermined
D7		2205 A07	Aplano	UNKNOWN	68.957	34.8294
D8		2205 A07	Aplano	UNKNOWN	43.0676	35.5261
D9		2205 A07	Aplano	UNKNOWN	40.4394	35.6193
D10		2205 A07	Aplano	UNKNOWN	80.9757	34.5915
E1			Aplano	STANDARD	200	33.3685
E2			Aplano	STANDARD	200	33.2375
E3		2205 A04	Aplano	UNKNOWN		Undetermined
E4		2205 A04	Aplano	UNKNOWN		Undetermined
E5		2205 A04	Aplano	UNKNOWN		Undetermined
E6		2205 A04	Aplano	UNKNOWN		Undetermined
E7		2205 A07	Aplano	UNKNOWN		Undetermined
E8		2205 A07	Aplano	UNKNOWN		Undetermined
E9		2205 A07	Aplano	UNKNOWN	3.0127	39.4632
E10		2205 A07	Aplano	UNKNOWN	26.8934	36.2231
F1			Aplano	STANDARD	20	36.8321
F2			Aplano	STANDARD	20	36.4404
F3		2205 A04 10m	Aplano	UNKNOWN	67.3982	34.8632
F4		2205 A04 10m	Aplano	UNKNOWN	62.779	34.9683
F5		2205 A04 10m	Aplano	UNKNOWN	88.7564	34.4557
F6		2205 A04	Aplano	UNKNOWN		Undetermined
F7		2205 A07 10m	Aplano	UNKNOWN	1434.8606	30.3366
F8		2205 A07 10m	Aplano	UNKNOWN	1400.5104	30.3724
F9		2205 A07 10m	Aplano	UNKNOWN	1359.7671	30.4161
F10		2205 A07	Aplano	UNKNOWN		Undetermined
G3		2205 A04 SCM	Aplano	UNKNOWN	113.9411	34.086
G4		2205 A04 SCM	Aplano	UNKNOWN	118.6506	34.0261
G5		2205 A04 SCM	Aplano	UNKNOWN	103.0734	34.2344
G7		2205 A07 SCM	Aplano	UNKNOWN	10618.333	27.374

Well		Sample	Target	Task	Quantity	C
G8		2205 A07 SCM	Aplano	UNKNOWN	11734.3447	27.2261
G9		2205 A07 SCM	Aplano	UNKNOWN	11335.4209	27.2773
G10		2205 A07	Aplano	UNKNOWN		Undetermined
G12			Aplano	NTC		Undetermined
H1		Comf2E4	Aplano	UNKNOWN	19307.9688	26.489
H2		Comf2E4	Aplano	UNKNOWN	20582.2734	26.3944
H3		2205 A04 200m	Aplano	UNKNOWN	4.1343	38.9948
H4		2205 A04 200m	Aplano	UNKNOWN	7.9104	38.0344
H5		2205 A04 200m	Aplano	UNKNOWN	4.0124	39.0391
H7		2205 A07 200m	Aplano	UNKNOWN	258.1965	32.8752
H8		2205 A07 200m	Aplano	UNKNOWN	237.3782	32.9996
H9		2205 A07 200m	Aplano	UNKNOWN	237.045	33.0017
H10		2205 A07	Aplano	UNKNOWN	23.9228	36.3963
H12			Aplano	NTC		Undetermined

QC Summary

Total Wells	96	Processed Wells	76	Targets Used	1
Well Setup	76	Flagged Wells	26	Samples Used	21

Flag	Name	Frequency	Locations
AMPNC	Amplification in negative control	0	
BADROX	Bad passive reference signal	0	
BLFAIL	Baseline algorithm failed	0	
CTFAIL	C algorithm failed	0	
EXPFAIL	Exponential algorithm failed	17	A6, B6, C3, C4, C5, C6, D3, D4, D6, E3, E5, E6, E7, E8, F6, F10, G10
HIGHSD	High standard deviation in replicate group	9	B3, B4, B5, C10, D10, E10, H3, H4, H5
NOAMP	No amplification	13	C3, C4, C5, C6, D3, D4, D6, E5, E6, E7, E8, F6, G10
NOISE	Noise higher than others in plate	0	
NOSIGNAL	No signal in well	0	
OFFSCALE	Fluorescence is offscale	0	
OUTLIERRG	Outlier in replicate group	0	
SPIKE	Noise spikes	0	
THOLDFAIL	Thresholding algorithm failed	0	
