# Supplementary material for: Ecological impact of algal-preying protists aplanochytrids (Labyrinthulea) as a source of docosahexaenoic acid for copepods in marine environments
Source: ISME Commun. 2026 Jun 26;6(1):ycag178. doi: 10.1093/ismeco/ycag178 (PMC13685785; doi:10.1093/ismeco/ycag178)
Supplement: Supplementary file 2 [file supplementary_materials_ycag178.zip › Morimoto_ISME_C_3rdrev_supplemental_data.zip!/Morimoto_ISME_C_3rdrev_supplemental_data/Supplemental_Data_2/Standard Curve/Aplanochytrid/2205/Aplano2205Aline_A01_N=1_data.pdf]

# Experiment Results Report

## 230309AplanoEmmAline2205A01ByKurabo

## Experiment Summary

| | | |
|---|---|---|
| Experiment Name | : | 230309AplanoEmmAline2205A01ByKurabo |
| Experiment Type | : | Quantitation - Standard Curve |
| File Name | : | 230309AplanoEmmAline2205A01ByKurabo.eds |
| Run Started | : | 2023 Mar 10 11:58:26 AM |
| Run Finished | : | 2023 Mar 10 1:33:10 PM |
| Run Duration | : | 94 minutes 44 seconds |
| Date Modified | : | 2023 Mar 10 2:34:46 PM |
| User | : | |
| Number of wells used | : | 25 |
| Number of wells with results | : | 25 |
| Instrument Name | : | |
| Instrument Type | : | Applied Biosystems StepOnePlus™ Instrument |
| Comments | : | |

# Results Summary

| Sample | Target | Quantity (Mean) | Quantity (Std Dev) | C (Mean) | C (Std Dev) |
|---|---|---|---|---|---|
| 2205 A01 10m saki | Aplano | 1.00 | | 39.70 | |
| 2205 A01 Bottom | Aplano | 2.52 | 1.39 | 38.53 | 0.93 |
| 2205 A01 SCM saki | Aplano | 3.49 | 2.36 | 38.17 | 1.23 |
| Comf2E4 | Aplano | 19351.78 | 3297.24 | 25.22 | 0.25 |

# Plate Layout

| | 1 | 2 | 3 | 4 | 5 | 6 | 7 | 8 | 9 | 10 | 11 | 12 |
|---|---|---|---|---|---|---|---|---|---|---|---|---|
| A | S Aplano<br>2E6<br>FAM-NFQ-MGB<br>CT: 18.61 | S Aplano<br>2E6<br>FAM-NFQ-MGB<br>CT: 18.48 | | 2205 A01 10m saki<br>U Aplano<br>FAM-NFQ-MGB<br>CT: Undetermined | 2205 A01 10m saki<br>U Aplano<br>FAM-NFQ-MGB<br>CT: Undetermined | 2205 A01 10m saki<br>U Aplano<br>1<br>FAM-NFQ-MGB<br>CT: 39.7 | | | | | | |
| B | S Aplano<br>2E5<br>FAM-NFQ-MGB<br>CT: 21.64 | S Aplano<br>2E5<br>FAM-NFQ-MGB<br>CT: 21.61 | | ⚠ 1<br>2205 A01 SCM saki<br>U Aplano<br>1.14<br>FAM-NFQ-MGB<br>CT: 39.51 | ⚠ 1<br>2205 A01 SCM saki<br>U Aplano<br>3.46<br>FAM-NFQ-MGB<br>CT: 37.88 | ⚠ 1<br>2205 A01 SCM saki<br>U Aplano<br>5.86<br>FAM-NFQ-MGB<br>CT: 37.11 | | | | | | |
| C | S Aplano<br>2E4<br>FAM-NFQ-MGB<br>CT: 25.11 | S Aplano<br>2E4<br>FAM-NFQ-MGB<br>CT: 25 | | ⚠ 1<br>2205 A01 Bottom saki<br>U Aplano<br>1.12<br>FAM-NFQ-MGB<br>CT: 39.54 | ⚠ 1<br>2205 A01 Bottom saki<br>U Aplano<br>3.91<br>FAM-NFQ-MGB<br>CT: 37.7 | ⚠ 1<br>2205 A01 Bottom saki<br>U Aplano<br>2.54<br>FAM-NFQ-MGB<br>CT: 38.34 | | | | | | |
| D | S Aplano<br>2E3<br>FAM-NFQ-MGB<br>CT: 28.51 | S Aplano<br>2E3<br>FAM-NFQ-MGB<br>CT: 28.72 | | | | | | | | | | |
| E | S Aplano<br>200<br>FAM-NFQ-MGB<br>CT: 32.07 | S Aplano<br>200<br>FAM-NFQ-MGB<br>CT: 32.05 | | | | | | | | | | |
| F | S Aplano<br>20<br>FAM-NFQ-MGB<br>CT: 35.22 | S Aplano<br>20<br>FAM-NFQ-MGB<br>CT: 35.25 | | | | | | | | | | |
| G | | | | | | | | | | | | |
| H | Comf2E4<br>U Aplano<br>1.7E4<br>FAM-NFQ-MGB<br>CT: 25.4 | Comf2E4<br>U Aplano<br>2.17E4<br>FAM-NFQ-MGB<br>CT: 25.05 | | | | | | | | | N Aplano<br>FAM-NFQ-MGB<br>CT: Undetermined | N Aplano<br>FAM-NFQ-MGB<br>CT: Undetermined |

# Standard Curves

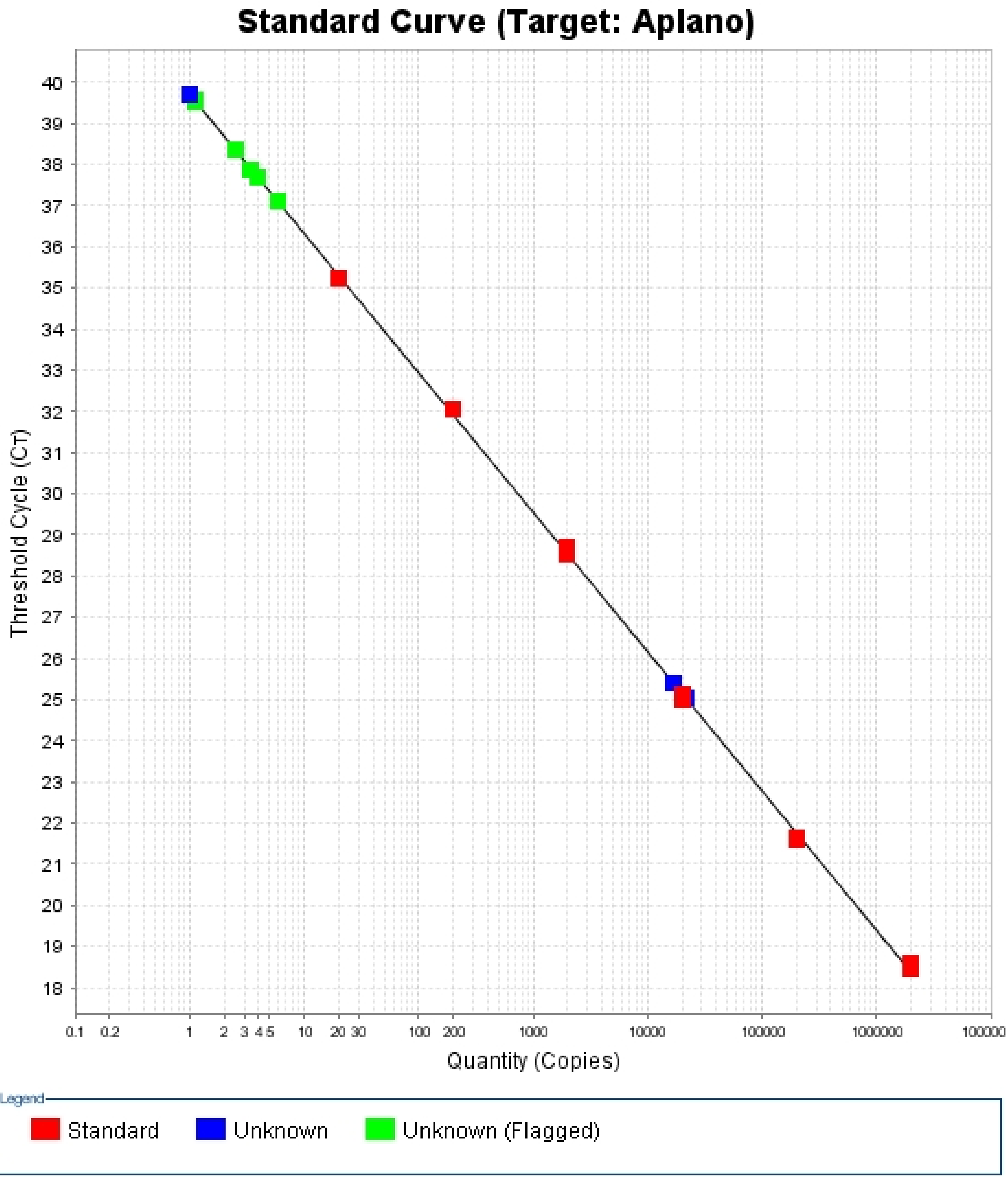


slope:-3.381 Y-Intercept:39.705 $R^2$:0.999 Eff%:97.604

# Results Table

| Well | | Sample | Target | Task | Quantity | C |
|---|---|---|---|---|---|---|
| A1 | | | Aplano | STANDARD | 2000000 | 18.6057 |
| A2 | | | Aplano | STANDARD | 2000000 | 18.4757 |
| A4 | | 2205 A01 10m | Aplano | UNKNOWN | | Undetermined |
| A5 | | 2205 A01 10m | Aplano | UNKNOWN | | Undetermined |
| A6 | | 2205 A01 10m | Aplano | UNKNOWN | 1.0038 | 39.6996 |
| B1 | | | Aplano | STANDARD | 200000 | 21.6441 |
| B2 | | | Aplano | STANDARD | 200000 | 21.6094 |
| B4 | | 2205 A01 SCM | Aplano | UNKNOWN | 1.1395 | 39.5135 |
| B5 | | 2205 A01 SCM | Aplano | UNKNOWN | 3.465 | 37.8806 |
| B6 | | 2205 A01 SCM | Aplano | UNKNOWN | 5.8554 | 37.1103 |
| C1 | | | Aplano | STANDARD | 20000 | 25.1102 |
| C2 | | | Aplano | STANDARD | 20000 | 25.0007 |
| C4 | | 2205 A01 | Aplano | UNKNOWN | 1.1176 | 39.5419 |
| C5 | | 2205 A01 | Aplano | UNKNOWN | 3.907 | 37.7044 |
| C6 | | 2205 A01 | Aplano | UNKNOWN | 2.5413 | 38.3358 |
| D1 | | | Aplano | STANDARD | 2000 | 28.5117 |
| D2 | | | Aplano | STANDARD | 2000 | 28.7164 |
| E1 | | | Aplano | STANDARD | 200 | 32.07 |
| E2 | | | Aplano | STANDARD | 200 | 32.0492 |
| F1 | | | Aplano | STANDARD | 20 | 35.217 |
| F2 | | | Aplano | STANDARD | 20 | 35.2513 |
| H1 | | Comf2E4 | Aplano | UNKNOWN | 17020.2754 | 25.4016 |
| H2 | | Comf2E4 | Aplano | UNKNOWN | 21683.2773 | 25.0461 |
| H11 | | | Aplano | NTC | | Undetermined |
| H12 | | | Aplano | NTC | | Undetermined |

# QC Summary

| | | | | | |
|---|---|---|---|---|---|
| Total Wells | 96 | Processed Wells | 25 | Targets Used | 1 |
| Well Setup | 25 | Flagged Wells | 6 | Samples Used | 4 |

| Flag | Name | Frequency | Locations |
|---|---|---|---|
| AMPNC | Amplification in negative control | 0 | |
| BADROX | Bad passive reference signal | 0 | |
| BLFAIL | Baseline algorithm failed | 0 | |
| CTFAIL | C algorithm failed | 0 | |
| EXPFAIL | Exponential algorithm failed | 0 | |
| HIGHSD | High standard deviation in replicate group | 6 | B4, B5, B6, C4, C5, C6 |
| NOAMP | No amplification | 0 | |
| NOISE | Noise higher than others in plate | 0 | |
| NOSIGNAL | No signal in well | 0 | |
| OFFSCALE | Fluorescence is offscale | 0 | |
| OUTLIERRG | Outlier in replicate group | 0 | |
| SPIKE | Noise spikes | 0 | |
| THOLDFAIL | Thresholding algorithm failed | 0 | |
