# Supplementary material for: Ecological impact of algal-preying protists aplanochytrids (Labyrinthulea) as a source of docosahexaenoic acid for copepods in marine environments
Source: ISME Commun. 2026 Jun 26;6(1):ycag178. doi: 10.1093/ismeco/ycag178 (PMC13685785; doi:10.1093/ismeco/ycag178)
Supplement: Supplementary file 2 [file supplementary_materials_ycag178.zip › Morimoto_ISME_C_3rdrev_supplemental_data.zip!/Morimoto_ISME_C_3rdrev_supplemental_data/Supplemental_Data_2/Standard Curve/Aplanochytrid/2205/Aplano2205Aline_A11-A21_N=1_data.pdf]

Experiment Results Report

230309AplanoEmmAline2205A11-A21ByKurabo

Experiment Summary

Experiment Name	:	230309AplanoEmmAline2205A11-A21ByKurabo
Experiment Type	:	Quantitation - Standard Curve
File Name	:	230309AplanoEmmAline2205A11-A21ByKurabo.eds
Run Started	:	2023 Mar 10 5:42:13 PM
Run Finished	:	2023 Mar 10 7:17:08 PM
Run Duration	:	94 minutes 55 seconds
Date Modified	:	2023 Mar 13 2:12:40 PM
User	:	
Number of wells used	:	46
Number of wells with results	:	46
Instrument Name	:	
Instrument Type	:	Applied Biosystems StepOnePlus™ Instrument
Comments	:	

Results Summary

Sample	Target	Quantity (Mean)	Quantity (Std Dev)	C (Mean)	C (Std Dev)
2205 A11 1000m	Aplano	13.71	12.90	33.09	1.37
2205 A11 10m saki	Aplano	15.36	2.27	32.50	0.21
2205 A11 200m saki	Aplano	126.22	29.80	29.41	0.37
2205 A11 3000m	Aplano	1.38	1.03	36.27	1.22
2205 A11 SCM saki	Aplano	42.42	15.64	31.05	0.52
2205 A21 1000m	Aplano	2.93		34.93	
2205 A21 10m saki	Aplano	127.35	15.78	29.38	0.18
2205 A21 200m saki	Aplano	51.42	5.29	30.71	0.16
2205 A21 3000m	Aplano	5.88	3.54	34.05	0.95
2205 A21 SCM saki	Aplano	247.57	35.07	28.40	0.20
Comf2E4	Aplano	17655.65	3158.21	22.11	0.26

Plate Layout

	1	2	3	4	5	6	7	8	9	10	11	12
A	S Aplano<br>2E6<br>FAM-NFQ-MGB<br>CT: 15.85	S Aplano<br>2E6<br>FAM-NFQ-MGB<br>CT: 15.15		2205 A11 10m saki<br>U Aplano<br>14.12<br>FAM-NFQ-MGB<br>CT: 32.61	2205 A11 10m saki<br>U Aplano<br>13.99<br>FAM-NFQ-MGB<br>CT: 32.62	2205 A11 10m saki<br>U Aplano<br>17.98<br>FAM-NFQ-MGB<br>CT: 32.25		2205 A21 10m saki<br>U Aplano<br>145.38<br>FAM-NFQ-MGB<br>CT: 29.17	2205 A21 10m saki<br>U Aplano<br>120.61<br>FAM-NFQ-MGB<br>CT: 29.45	2205 A21 10m saki<br>U Aplano<br>116.06<br>FAM-NFQ-MGB<br>CT: 29.51		
B	S Aplano<br>2E5<br>FAM-NFQ-MGB<br>CT: 18.16	S Aplano<br>2E5<br>FAM-NFQ-MGB<br>CT: 18.38		⚠1<br>2205 A11 SCM saki<br>U Aplano<br>30.71<br>FAM-NFQ-MGB<br>CT: 31.46	⚠1<br>2205 A11 SCM saki<br>U Aplano<br>36.37<br>FAM-NFQ-MGB<br>CT: 31.22	⚠1<br>2205 A11 SCM saki<br>U Aplano<br>60.18<br>FAM-NFQ-MGB<br>CT: 30.47		2205 A21 SCM saki<br>U Aplano<br>219.84<br>FAM-NFQ-MGB<br>CT: 28.56	2205 A21 SCM saki<br>U Aplano<br>286.99<br>FAM-NFQ-MGB<br>CT: 28.17	2205 A21 SCM saki<br>U Aplano<br>235.89<br>FAM-NFQ-MGB<br>CT: 28.46		
C	S Aplano<br>2E4<br>FAM-NFQ-MGB<br>CT: 21.8	S Aplano<br>2E4<br>FAM-NFQ-MGB<br>CT: 21.66		2205 A11 200m saki<br>U Aplano<br>131.41<br>FAM-NFQ-MGB<br>CT: 29.32	2205 A11 200m saki<br>U Aplano<br>153.08<br>FAM-NFQ-MGB<br>CT: 29.1	2205 A11 200m saki<br>U Aplano<br>94.17<br>FAM-NFQ-MGB<br>CT: 29.81		2205 A21 200m saki<br>U Aplano<br>45.44<br>FAM-NFQ-MGB<br>CT: 30.89	2205 A21 200m saki<br>U Aplano<br>53.33<br>FAM-NFQ-MGB<br>CT: 30.65	2205 A21 200m saki<br>U Aplano<br>55.48<br>FAM-NFQ-MGB<br>CT: 30.59		
D	S Aplano<br>2E3<br>FAM-NFQ-MGB<br>CT: 25.22	S Aplano<br>2E3<br>FAM-NFQ-MGB<br>CT: 25.28		⚠1<br>2205 A11 1000m saki<br>U Aplano<br>28.48<br>FAM-NFQ-MGB<br>CT: 31.58	⚠1<br>2205 A11 1000m saki<br>U Aplano<br>4.61<br>FAM-NFQ-MGB<br>CT: 34.26	⚠1<br>2205 A11 1000m saki<br>U Aplano<br>8.05<br>FAM-NFQ-MGB<br>CT: 33.44		2205 A21 1000m saki<br>U Aplano<br>2.93<br>FAM-NFQ-MGB<br>CT: 34.93	⚠1<br>2205 A21 1000m saki<br>U Aplano<br>FAM-NFQ-MGB<br>CT: Undetermined	⚠2<br>2205 A21 1000m saki<br>U Aplano<br>FAM-NFQ-MGB<br>CT: Undetermined		
E	S Aplano<br>200<br>FAM-NFQ-MGB<br>CT: 28.61	S Aplano<br>200<br>FAM-NFQ-MGB<br>CT: 28.57		⚠1<br>2205 A11 3000m saki<br>U Aplano<br>0.66<br>FAM-NFQ-MGB<br>CT: 37.13	⚠1<br>2205 A11 3000m saki<br>U Aplano<br>FAM-NFQ-MGB<br>CT: Undetermined	⚠1<br>2205 A11 3000m saki<br>U Aplano<br>2.11<br>FAM-NFQ-MGB<br>CT: 35.41		⚠1<br>2205 A21 3000m saki<br>U Aplano<br>3.38<br>FAM-NFQ-MGB<br>CT: 34.72	⚠1<br>2205 A21 3000m saki<br>U Aplano<br>8.38<br>FAM-NFQ-MGB<br>CT: 33.38	⚠2<br>2205 A21 3000m saki<br>U Aplano<br>FAM-NFQ-MGB<br>CT: Undetermined		
F	⚠1<br>S Aplano<br>20<br>FAM-NFQ-MGB<br>CT: 31.99	⚠1<br>S Aplano<br>20<br>FAM-NFQ-MGB<br>CT: 32.71										
G												
H	Comf2E4<br>U Aplano<br>1.54E4<br>FAM-NFQ-MGB<br>CT: 22.3	Comf2E4<br>U Aplano<br>1.99E4<br>FAM-NFQ-MGB<br>CT: 21.93									N Aplano<br>FAM-NFQ-MGB<br>CT: Undetermined	N Aplano<br>FAM-NFQ-MGB<br>CT: Undetermined

Standard Curves

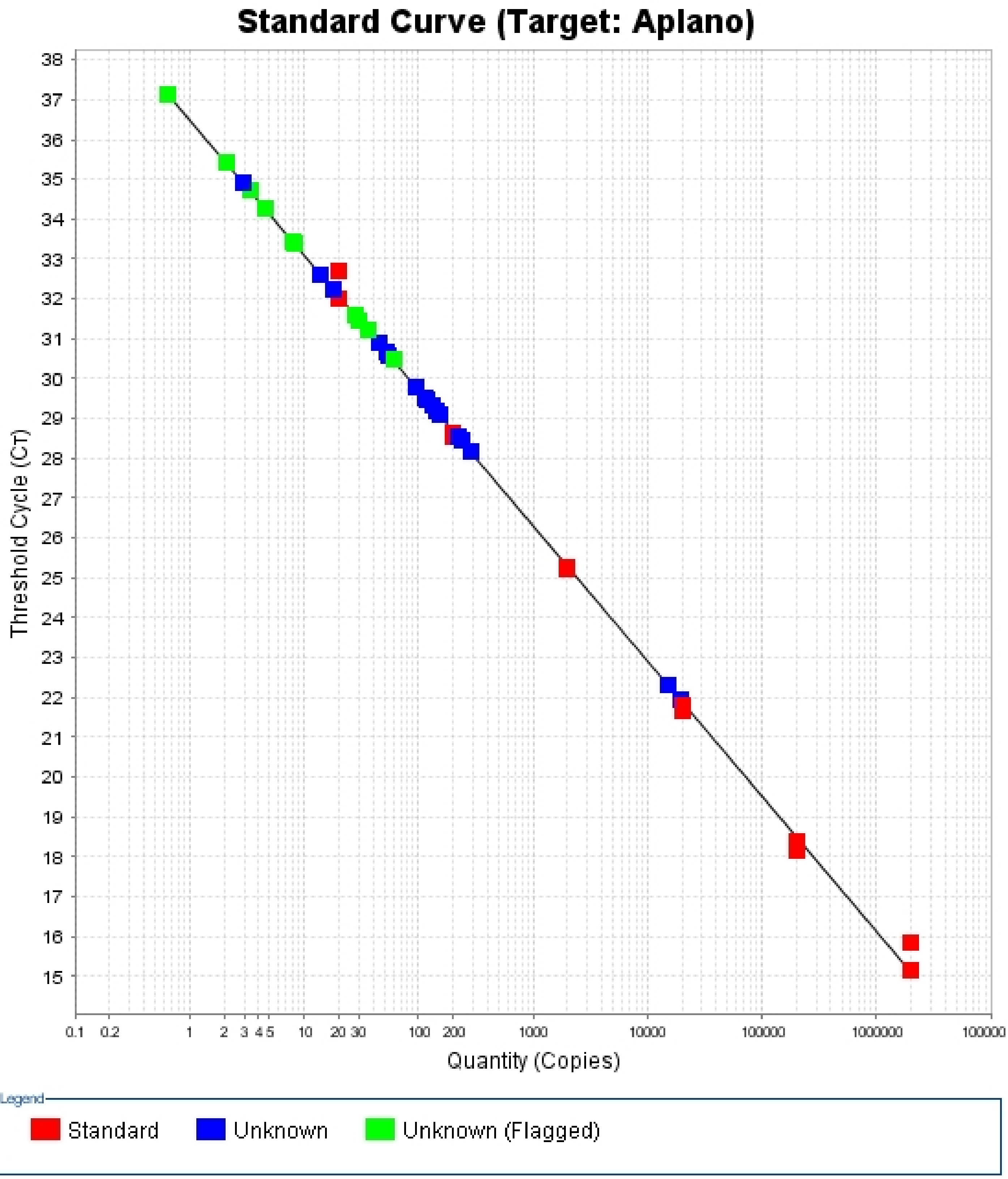

slope:-3.392 Y-Intercept:36.51 R^2:0.997 Eff%:97.141

Results Table

Well		Sample	Target	Task	Quantity	C
A1			Aplano	STANDARD	2000000	15.8534
A2			Aplano	STANDARD	2000000	15.1466
A4		2205 A11 10m	Aplano	UNKNOWN	14.1155	32.6097
A5		2205 A11 10m	Aplano	UNKNOWN	13.9859	32.6233
A6		2205 A11 10m	Aplano	UNKNOWN	17.9765	32.2534
A8		2205 A21 10m	Aplano	UNKNOWN	145.3793	29.1738
A9		2205 A21 10m	Aplano	UNKNOWN	120.6144	29.449
A10		2205 A21 10m	Aplano	UNKNOWN	116.0615	29.5057
B1			Aplano	STANDARD	200000	18.16
B2			Aplano	STANDARD	200000	18.3772
B4		2205 A11 SCM	Aplano	UNKNOWN	30.709	31.4645
B5		2205 A11 SCM	Aplano	UNKNOWN	36.3702	31.2152
B6		2205 A11 SCM	Aplano	UNKNOWN	60.1811	30.4733
B8		2205 A21 SCM	Aplano	UNKNOWN	219.8369	28.5646
B9		2205 A21 SCM	Aplano	UNKNOWN	286.9877	28.1718
B10		2205 A21 SCM	Aplano	UNKNOWN	235.8937	28.4607
C1			Aplano	STANDARD	20000	21.8033
C2			Aplano	STANDARD	20000	21.6572
C4		2205 A11 200m	Aplano	UNKNOWN	131.4063	29.3227
C5		2205 A11 200m	Aplano	UNKNOWN	153.0787	29.0978
C6		2205 A11 200m	Aplano	UNKNOWN	94.1662	29.8137
C8		2205 A21 200m	Aplano	UNKNOWN	45.4396	30.8872
C9		2205 A21 200m	Aplano	UNKNOWN	53.3335	30.6512
C10		2205 A21 200m	Aplano	UNKNOWN	55.484	30.593
D1			Aplano	STANDARD	2000	25.2219
D2			Aplano	STANDARD	2000	25.2802
D4		2205 A11	Aplano	UNKNOWN	28.4786	31.5756
D5		2205 A11	Aplano	UNKNOWN	4.61	34.2584
D6		2205 A11	Aplano	UNKNOWN	8.0544	33.4363
D8		2205 A21	Aplano	UNKNOWN	2.9256	34.9283

Well		Sample	Target	Task	Quantity	C
D9		2205 A21	Aplano	UNKNOWN		Undetermined
D10		2205 A21	Aplano	UNKNOWN		Undetermined
E1			Aplano	STANDARD	200	28.6147
E2			Aplano	STANDARD	200	28.5676
E4		2205 A11	Aplano	UNKNOWN	0.6552	37.1328
E5		2205 A11	Aplano	UNKNOWN		Undetermined
E6		2205 A11	Aplano	UNKNOWN	2.1135	35.4074
E8		2205 A21	Aplano	UNKNOWN	3.3769	34.717
E9		2205 A21	Aplano	UNKNOWN	8.3847	33.3771
E10		2205 A21	Aplano	UNKNOWN		Undetermined
F1			Aplano	STANDARD	20	31.9942
F2			Aplano	STANDARD	20	32.705
H1		Comf2E4	Aplano	UNKNOWN	15422.459	22.302
H2		Comf2E4	Aplano	UNKNOWN	19888.8359	21.9273
H11			Aplano	NTC		Undetermined
H12			Aplano	NTC		Undetermined

QC Summary

Total Wells	96	Processed Wells	46	Targets Used	1
Well Setup	46	Flagged Wells	16	Samples Used	11

Flag	Name	Frequency	Locations
AMPNC	Amplification in negative control	0	
BADROX	Bad passive reference signal	0	
BLFAIL	Baseline algorithm failed	0	
CTFAIL	C algorithm failed	0	
EXPFAIL	Exponential algorithm failed	4	D9, D10, E5, E10
HIGHSD	High standard deviation in replicate group	12	B4, B5, B6, D4, D5, D6, E4, E6, E8, E9, F1, F2
NOAMP	No amplification	2	D10, E10
NOISE	Noise higher than others in plate	0	
NOSIGNAL	No signal in well	0	
OFFSCALE	Fluorescence is offscale	0	
OUTLIERRG	Outlier in replicate group	0	
SPIKE	Noise spikes	0	
THOLDFAIL	Thresholding algorithm failed	0	
