# Supplementary material for: Ecological impact of algal-preying protists aplanochytrids (Labyrinthulea) as a source of docosahexaenoic acid for copepods in marine environments
Source: ISME Commun. 2026 Jun 26;6(1):ycag178. doi: 10.1093/ismeco/ycag178 (PMC13685785; doi:10.1093/ismeco/ycag178)
Supplement: Supplementary file 2 [file supplementary_materials_ycag178.zip › Morimoto_ISME_C_3rdrev_supplemental_data.zip!/Morimoto_ISME_C_3rdrev_supplemental_data/Supplemental_Data_2/Standard Curve/Aplanochytrid/2205/Aplano2205Aline_A11-A21_N=2,3_data.pdf]

Experiment Results Report
230329AplanoAline2205A11A21ByKurabo

Experiment Summary

Experiment Name	:	230329AplanoAline2205A11A21ByKurabo
Experiment Type	:	Quantitation - Standard Curve
File Name	:	230329AplanoAline2205A11A21ByKurabo.eds
Run Started	:	2023 Mar 29 1:00:53 PM
Run Finished	:	2023 Mar 29 2:35:31 PM
Run Duration	:	94 minutes 37 seconds
Date Modified	:	2023 Mar 29 2:53:09 PM
User	:	
Number of wells used	:	76
Number of wells with results	:	76
Instrument Name	:	
Instrument Type	:	Applied Biosystems StepOnePlus™ Instrument
Comments	:	

Results Summary

Sample	Target	Quantity (Mean)	Quantity (Std Dev)	C (Mean)	C (Std Dev)
2205 A11 1000m	Aplano	1.00	0.03	39.96	0.05
2205 A11 1000m	Aplano	10.04	6.19	36.67	0.86
2205 A11 10m saki2	Aplano	25.03	7.90	35.16	0.44
2205 A11 10m saki3	Aplano	29.55	11.20	34.94	0.55
2205 A11 200m	Aplano	176.59	21.21	32.19	0.18
2205 A11 200m	Aplano	177.95	28.49	32.19	0.25
2205 A11 3000m	Aplano	1.90	1.29	39.19	1.11
2205 A11 3000m	Aplano	15.56	4.97	35.89	0.50
2205 A11 SCM saki2	Aplano	116.94	20.28	32.82	0.28
2205 A11 SCM saki3	Aplano	89.46	12.58	33.22	0.20
2205 A21 1000m	Aplano	6.43		37.16	
2205 A21 1000m	Aplano	3.86	3.71	38.36	1.35
2205 A21 10m saki2	Aplano	277.41	52.96	31.53	0.30
2205 A21 10m saki3	Aplano	223.42	25.04	31.84	0.17
2205 A21 200m	Aplano	51.40	12.66	34.07	0.38
2205 A21 200m	Aplano	94.64	20.07	33.14	0.31
2205 A21 3000m	Aplano	10.09	4.96	36.65	0.93
2205 A21 3000m	Aplano				
2205 A21 SCM saki2	Aplano	585.03	20.32	30.39	0.05
2205 A21 SCM saki3	Aplano	173.14	27.19	32.23	0.25
Comf2E4	Aplano	21471.91	1911.05	24.98	0.13

Plate Layout

	1	2	3	4	5	6	7	8	9	10	11	12
A	S Aplano<br>2E6<br>FAM-NFQ-MGB<br>CT: 18.35	S Aplano<br>2E6<br>FAM-NFQ-MGB<br>CT: 18.05	2205 A11 10m saki2<br>U Aplano<br>20.38<br>FAM-NFQ-MGB<br>CT: 35.43	⚠1<br>2205 A11 10m saki2<br>U Aplano<br>34.15<br>FAM-NFQ-MGB<br>CT: 34.65	2205 A11 10m saki2<br>U Aplano<br>20.58<br>FAM-NFQ-MGB<br>CT: 35.41	⚠1<br>2205 A11 1000m saki3<br>U Aplano<br>5.76<br>FAM-NFQ-MGB<br>CT: 37.32	2205 A21 10m saki2<br>U Aplano<br>296.66<br>FAM-NFQ-MGB<br>CT: 31.41	2205 A21 10m saki2<br>U Aplano<br>217.52<br>FAM-NFQ-MGB<br>CT: 31.87	2205 A21 10m saki2<br>U Aplano<br>318.06<br>FAM-NFQ-MGB<br>CT: 31.3	⚠1<br>2205 A21 1000m saki3<br>U Aplano<br>8.14<br>FAM-NFQ-MGB<br>CT: 36.81		
B	S Aplano<br>2E5<br>FAM-NFQ-MGB<br>CT: 21.4	S Aplano<br>2E5<br>FAM-NFQ-MGB<br>CT: 21.39	2205 A11 SCM saki2<br>U Aplano<br>131.37<br>FAM-NFQ-MGB<br>CT: 32.63	2205 A11 SCM saki2<br>U Aplano<br>125.69<br>FAM-NFQ-MGB<br>CT: 32.7	2205 A11 SCM saki2<br>U Aplano<br>93.75<br>FAM-NFQ-MGB<br>CT: 33.14	⚠1<br>2205 A11 1000m saki3<br>U Aplano<br>17.13<br>FAM-NFQ-MGB<br>CT: 35.69	2205 A21 SCM saki2<br>U Aplano<br>596.66<br>FAM-NFQ-MGB<br>CT: 30.36	2205 A21 SCM saki2<br>U Aplano<br>596.85<br>FAM-NFQ-MGB<br>CT: 30.36	2205 A21 SCM saki2<br>U Aplano<br>561.57<br>FAM-NFQ-MGB<br>CT: 30.45	⚠1<br>2205 A21 1000m saki3<br>U Aplano<br>1.85<br>FAM-NFQ-MGB<br>CT: 39.03		
C	S Aplano<br>2E4<br>FAM-NFQ-MGB<br>CT: 25.48	S Aplano<br>2E4<br>FAM-NFQ-MGB<br>CT: 24.93	2205 A11 200m saki2<br>U Aplano<br>200.96<br>FAM-NFQ-MGB<br>CT: 31.99	2205 A11 200m saki2<br>U Aplano<br>162.36<br>FAM-NFQ-MGB<br>CT: 32.31	2205 A11 200m saki2<br>U Aplano<br>166.45<br>FAM-NFQ-MGB<br>CT: 32.27	⚠1<br>2205 A11 1000m saki3<br>U Aplano<br>7.22<br>FAM-NFQ-MGB<br>CT: 36.99	2205 A21 200m saki2<br>U Aplano<br>38.27<br>FAM-NFQ-MGB<br>CT: 34.48	2205 A21 200m saki2<br>U Aplano<br>52.4<br>FAM-NFQ-MGB<br>CT: 34.01	2205 A21 200m saki2<br>U Aplano<br>63.53<br>FAM-NFQ-MGB<br>CT: 33.72	⚠1<br>2205 A21 1000m saki3<br>U Aplano<br>1.59<br>FAM-NFQ-MGB<br>CT: 39.25		
D	S Aplano<br>2E3<br>FAM-NFQ-MGB<br>CT: 28.69	S Aplano<br>2E3<br>FAM-NFQ-MGB<br>CT: 28.66	⚠2<br>2205 A11 1000m saki2<br>U Aplano<br>FAM-NFQ-MGB<br>CT: Undetermined	2205 A11 1000m saki2<br>U Aplano<br>0.97<br>FAM-NFQ-MGB<br>CT: 39.99	2205 A11 1000m saki2<br>U Aplano<br>1.02<br>FAM-NFQ-MGB<br>CT: 39.93	2205 A11 3000m saki3<br>U Aplano<br>15.42<br>FAM-NFQ-MGB<br>CT: 35.85	2205 A21 1000m saki2<br>U Aplano<br>6.43<br>FAM-NFQ-MGB<br>CT: 37.16	⚠2<br>2205 A21 1000m saki2<br>U Aplano<br>FAM-NFQ-MGB<br>CT: Undetermined	⚠2<br>2205 A21 1000m saki2<br>U Aplano<br>FAM-NFQ-MGB<br>CT: Undetermined	⚠2<br>2205 A21 3000m saki3<br>U Aplano<br>FAM-NFQ-MGB<br>CT: Undetermined		
E	S Aplano<br>200<br>FAM-NFQ-MGB<br>CT: 32.15	S Aplano<br>200<br>FAM-NFQ-MGB<br>CT: 32.12	⚠1<br>2205 A11 3000m saki2<br>U Aplano<br>2.81<br>FAM-NFQ-MGB<br>CT: 38.4	⚠1<br>2205 A11 3000m saki2<br>U Aplano<br>FAM-NFQ-MGB<br>CT: Undetermined	⚠1<br>2205 A11 3000m saki2<br>U Aplano<br>0.99<br>FAM-NFQ-MGB<br>CT: 39.97	2205 A11 3000m saki3<br>U Aplano<br>10.66<br>FAM-NFQ-MGB<br>CT: 36.4	⚠1<br>2205 A21 3000m saki2<br>U Aplano<br>4.41<br>FAM-NFQ-MGB<br>CT: 37.73	⚠1<br>2205 A21 3000m saki2<br>U Aplano<br>13.52<br>FAM-NFQ-MGB<br>CT: 36.04	⚠1<br>2205 A21 3000m saki2<br>U Aplano<br>12.34<br>FAM-NFQ-MGB<br>CT: 36.18	⚠1<br>2205 A21 3000m saki3<br>U Aplano<br>FAM-NFQ-MGB<br>CT: Undetermined		
F	S Aplano<br>20<br>FAM-NFQ-MGB<br>CT: 35.05	S Aplano<br>20<br>FAM-NFQ-MGB<br>CT: 35.48	⚠1<br>2205 A11 10m saki3<br>U Aplano<br>25.97<br>FAM-NFQ-MGB<br>CT: 35.06	⚠1<br>2205 A11 10m saki3<br>U Aplano<br>42.11<br>FAM-NFQ-MGB<br>CT: 34.34	⚠1<br>2205 A11 10m saki3<br>U Aplano<br>20.58<br>FAM-NFQ-MGB<br>CT: 35.41	2205 A11 3000m saki3<br>U Aplano<br>20.59<br>FAM-NFQ-MGB<br>CT: 35.41	2205 A21 10m saki3<br>U Aplano<br>202.07<br>FAM-NFQ-MGB<br>CT: 31.98	2205 A21 10m saki3<br>U Aplano<br>250.98<br>FAM-NFQ-MGB<br>CT: 31.66	2205 A21 10m saki3<br>U Aplano<br>217.21<br>FAM-NFQ-MGB<br>CT: 31.87	⚠2<br>2205 A21 3000m saki3<br>U Aplano<br>FAM-NFQ-MGB<br>CT: Undetermined		
G			2205 A11 SCM saki3<br>U Aplano<br>103.87<br>FAM-NFQ-MGB<br>CT: 32.98	2205 A11 SCM saki3<br>U Aplano<br>83.85<br>FAM-NFQ-MGB<br>CT: 33.3	2205 A11 SCM saki3<br>U Aplano<br>80.67<br>FAM-NFQ-MGB<br>CT: 33.36		⚠1<br>2205 A21 SCM saki3<br>U Aplano<br>141.75<br>FAM-NFQ-MGB<br>CT: 32.52	2205 A21 SCM saki3<br>U Aplano<br>189.4<br>FAM-NFQ-MGB<br>CT: 32.08	2205 A21 SCM saki3<br>U Aplano<br>188.28<br>FAM-NFQ-MGB<br>CT: 32.09			N Aplano<br>FAM-NFQ-MGB<br>CT: Undetermined
H	Comf2E4<br>U Aplano<br>2.01E4<br>FAM-NFQ-MGB<br>CT: 25.07	Comf2E4<br>U Aplano<br>2.28E4<br>FAM-NFQ-MGB<br>CT: 24.89	2205 A11 200m saki3<br>U Aplano<br>200.58<br>FAM-NFQ-MGB<br>CT: 31.99	2205 A11 200m saki3<br>U Aplano<br>187.3<br>FAM-NFQ-MGB<br>CT: 32.1	2205 A11 200m saki3<br>U Aplano<br>145.96<br>FAM-NFQ-MGB<br>CT: 32.47		2205 A21 200m saki3<br>U Aplano<br>88.09<br>FAM-NFQ-MGB<br>CT: 33.23	2205 A21 200m saki3<br>U Aplano<br>78.67<br>FAM-NFQ-MGB<br>CT: 33.4	2205 A21 200m saki3<br>U Aplano<br>117.17<br>FAM-NFQ-MGB<br>CT: 32.8			N Aplano<br>FAM-NFQ-MGB<br>CT: Undetermined

Standard Curves

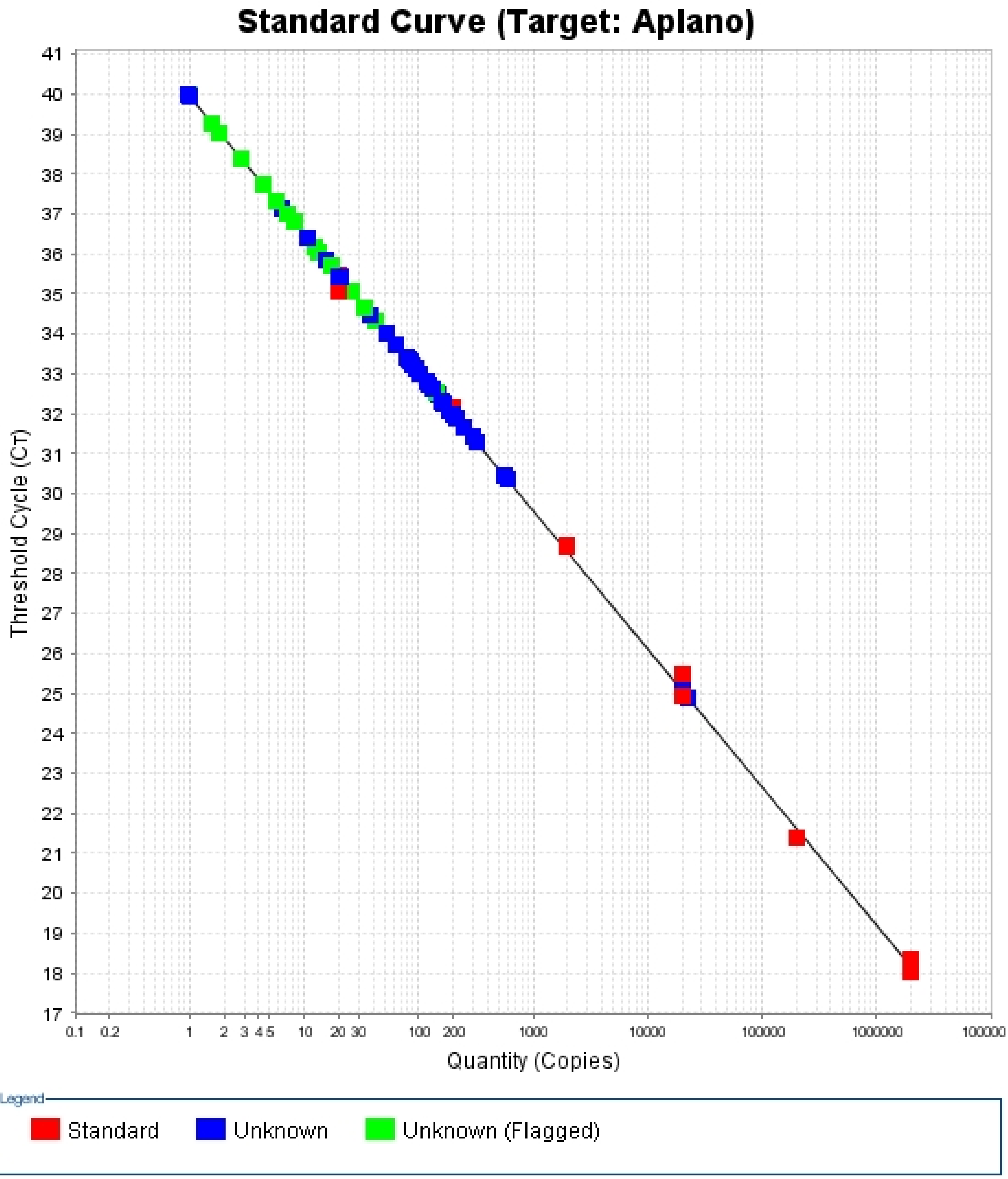

slope:-3.457 Y-Intercept:39.954 R^2:0.999 Eff%:94.641

Results Table

Well		Sample	Target	Task	Quantity	C
A1			Aplano	STANDARD	2000000	18.3548
A2			Aplano	STANDARD	2000000	18.0518
A3		2205 A11 10m	Aplano	UNKNOWN	20.377	35.4276
A4		2205 A11 10m	Aplano	UNKNOWN	34.1502	34.6522
A5		2205 A11 10m	Aplano	UNKNOWN	20.576	35.413
A6		2205 A11	Aplano	UNKNOWN	5.7606	37.3246
A7		2205 A21 10m	Aplano	UNKNOWN	296.6615	31.4062
A8		2205 A21 10m	Aplano	UNKNOWN	217.5182	31.8721
A9		2205 A21 10m	Aplano	UNKNOWN	318.0626	31.3016
A10		2205 A21	Aplano	UNKNOWN	8.1377	36.8058
B1			Aplano	STANDARD	200000	21.3953
B2			Aplano	STANDARD	200000	21.3864
B3		2205 A11 SCM	Aplano	UNKNOWN	131.3711	32.6293
B4		2205 A11 SCM	Aplano	UNKNOWN	125.6861	32.6957
B5		2205 A11 SCM	Aplano	UNKNOWN	93.7507	33.1359
B6		2205 A11	Aplano	UNKNOWN	17.1291	35.6883
B7		2205 A21 SCM	Aplano	UNKNOWN	596.6605	30.357
B8		2205 A21 SCM	Aplano	UNKNOWN	596.8524	30.3565
B9		2205 A21 SCM	Aplano	UNKNOWN	561.5684	30.448
B10		2205 A21	Aplano	UNKNOWN	1.8488	39.0311
C1			Aplano	STANDARD	20000	25.4776
C2			Aplano	STANDARD	20000	24.9268
C3		2205 A11 200m	Aplano	UNKNOWN	200.9645	31.991
C4		2205 A11 200m	Aplano	UNKNOWN	162.3574	32.3113
C5		2205 A11 200m	Aplano	UNKNOWN	166.45	32.2739
C6		2205 A11	Aplano	UNKNOWN	7.2157	36.9864
C7		2205 A21 200m	Aplano	UNKNOWN	38.2675	34.4813
C8		2205 A21 200m	Aplano	UNKNOWN	52.3999	34.0094
C9		2205 A21 200m	Aplano	UNKNOWN	63.5257	33.7203
C10		2205 A21	Aplano	UNKNOWN	1.5941	39.2537

Well		Sample	Target	Task	Quantity	C
D1			Aplano	STANDARD	2000	28.6925
D2			Aplano	STANDARD	2000	28.6632
D3		2205 A11	Aplano	UNKNOWN		Undetermined
D4		2205 A11	Aplano	UNKNOWN	0.9741	39.9932
D5		2205 A11	Aplano	UNKNOWN	1.017	39.9285
D6		2205 A11	Aplano	UNKNOWN	15.4216	35.846
D7		2205 A21	Aplano	UNKNOWN	6.4299	37.1595
D8		2205 A21	Aplano	UNKNOWN		Undetermined
D9		2205 A21	Aplano	UNKNOWN		Undetermined
D10		2205 A21	Aplano	UNKNOWN		Undetermined
E1			Aplano	STANDARD	200	32.147
E2			Aplano	STANDARD	200	32.1178
E3		2205 A11	Aplano	UNKNOWN	2.8086	38.4032
E4		2205 A11	Aplano	UNKNOWN		Undetermined
E5		2205 A11	Aplano	UNKNOWN	0.9891	39.9703
E6		2205 A11	Aplano	UNKNOWN	10.6557	36.401
E7		2205 A21	Aplano	UNKNOWN	4.4095	37.7259
E8		2205 A21	Aplano	UNKNOWN	13.5186	36.0437
E9		2205 A21	Aplano	UNKNOWN	12.3444	36.1801
E10		2205 A21	Aplano	UNKNOWN		Undetermined
F1			Aplano	STANDARD	20	35.0519
F2			Aplano	STANDARD	20	35.4786
F3		2205 A11 10m	Aplano	UNKNOWN	25.9697	35.0634
F4		2205 A11 10m	Aplano	UNKNOWN	42.1105	34.3376
F5		2205 A11 10m	Aplano	UNKNOWN	20.5771	35.4129
F6		2205 A11	Aplano	UNKNOWN	20.5886	35.4121
F7		2205 A21 10m	Aplano	UNKNOWN	202.0726	31.9827
F8		2205 A21 10m	Aplano	UNKNOWN	250.9845	31.6573
F9		2205 A21 10m	Aplano	UNKNOWN	217.2147	31.8742
F10		2205 A21	Aplano	UNKNOWN		Undetermined
G3		2205 A11 SCM	Aplano	UNKNOWN	103.8671	32.982
G4		2205 A11 SCM	Aplano	UNKNOWN	83.8484	33.3035

Well		Sample	Target	Task	Quantity	C
G5		2205 A11 SCM	Aplano	UNKNOWN	80.6698	33.3615
G7		2205 A21 SCM	Aplano	UNKNOWN	141.747	32.5152
G8		2205 A21 SCM	Aplano	UNKNOWN	189.3957	32.08
G9		2205 A21 SCM	Aplano	UNKNOWN	188.2783	32.0889
G12			Aplano	NTC		Undetermined
H1		Comf2E4	Aplano	UNKNOWN	20120.5938	25.0744
H2		Comf2E4	Aplano	UNKNOWN	22823.2285	24.8851
H3		2205 A11 200m	Aplano	UNKNOWN	200.585	31.9938
H4		2205 A11 200m	Aplano	UNKNOWN	187.2975	32.0967
H5		2205 A11 200m	Aplano	UNKNOWN	145.9602	32.4712
H7		2205 A21 200m	Aplano	UNKNOWN	88.0875	33.2295
H8		2205 A21 200m	Aplano	UNKNOWN	78.6679	33.3993
H9		2205 A21 200m	Aplano	UNKNOWN	117.1658	32.8011
H12			Aplano	NTC		Undetermined

QC Summary

Total Wells	96	Processed Wells	76	Targets Used	1
Well Setup	76	Flagged Wells	23	Samples Used	21

Flag	Name	Frequency	Locations
AMPNC	Amplification in negative control	0	
BADROX	Bad passive reference signal	0	
BLFAIL	Baseline algorithm failed	0	
CTFAIL	C algorithm failed	0	
EXPFAIL	Exponential algorithm failed	7	D3, D8, D9, D10, E4, E10, F10
HIGHSD	High standard deviation in replicate group	14	A6, A10, B6, B10, C6, C10, E3, E5, E7, E8, E9, F3, F4, F5
NOAMP	No amplification	5	D3, D8, D9, D10, F10
NOISE	Noise higher than others in plate	0	
NOSIGNAL	No signal in well	0	
OFFSCALE	Fluorescence is offscale	0	
OUTLIERRG	Outlier in replicate group	2	A4, G7
SPIKE	Noise spikes	0	
THOLDFAIL	Thresholding algorithm failed	0	
