# Supplementary material for: Ecological impact of algal-preying protists aplanochytrids (Labyrinthulea) as a source of docosahexaenoic acid for copepods in marine environments
Source: ISME Commun. 2026 Jun 26;6(1):ycag178. doi: 10.1093/ismeco/ycag178 (PMC13685785; doi:10.1093/ismeco/ycag178)
Supplement: Supplementary file 2 [file supplementary_materials_ycag178.zip › Morimoto_ISME_C_3rdrev_supplemental_data.zip!/Morimoto_ISME_C_3rdrev_supplemental_data/Supplemental_Data_2/Standard Curve/Aplanochytrid/2203/Aplano2203Aline(N=2).pdf]

Experiment Results Report

230524Aplano2203AlineByKuraboEmm

Experiment Summary

Experiment Name	: 230524Aplano2203AlineByKuraboEmm
Experiment Type	: Quantitation - Standard Curve
File Name	: 230524Aplano2203AlineByKuraboEmm.eds
Run Started	: 2023 May 24 12:51:51 PM
Run Finished	: 2023 May 24 2:26:25 PM
Run Duration	: 94 minutes 34 seconds
Date Modified	: 2023 May 24 2:49:54 PM
User	:
Number of wells used	: 79
Number of wells with results	: 79
Instrument Name	:
Instrument Type	: Applied Biosystems StepOnePlus™ Instrument
Comments	:

Results Summary

Sample	Target	Quantity (Mean)	Quantity (Std Dev)	C (Mean)	C (Std Dev)
2203 A01 10 saki	Aplano	4.14	2.45	35.30	0.89
2203 A01 BTM saki	Aplano	6.65	1.66	34.46	0.40
2203 A01 SCM saki	Aplano	5.47	0.30	34.72	0.08
2203 A04 10 saki	Aplano	2.38		35.93	
2203 A04 1000 saki	Aplano				
2203 A04 200 saki	Aplano	2.29	0.37	35.99	0.24
2203 A04 3000 saki	Aplano				
2203 A04 SCM saki	Aplano	9.11	3.36	34.05	0.60
2203 A07 10 saki	Aplano	16.95	4.39	33.10	0.36
2203 A07 1000 saki	Aplano				
2203 A07 200 saki	Aplano	7.56	5.16	34.49	1.07
2203 A07 3000 saki	Aplano	7.21		34.31	
2203 A07 SCM saki	Aplano	18.82	3.73	32.93	0.28
2203 A11 1000 saki	Aplano	1.72	0.82	36.49	0.73
2203 A11 200 saki	Aplano	119.21	32.46	30.26	0.41
2203 A11 3000 saki	Aplano				
2203 A11 SCM saki	Aplano	112.04	8.92	30.32	0.11
2203 A21 1000 saki	Aplano				
2203 A21 200 saki	Aplano	45.57	13.69	31.67	0.44
2203 A21 3000 saki	Aplano				
2203 A21 SCM saki	Aplano	146.88	43.99	29.97	0.49
Comf2E4	Aplano	17063.62	1181.60	22.99	0.10

Plate Layout

	1	2	3	4	5	6	7	8	9	10	11	12
A	S Aplano<br>2E6<br>FAM-NFQ-MGB<br>CT: 16.49	S Aplano<br>2E6<br>FAM-NFQ-MGB<br>CT: 15.92	⚠1<br>2203 A01 10 saki<br>U Aplano<br>3.61<br>FAM-NFQ-MGB<br>CT: 35.32	⚠1<br>2203 A01 10 saki<br>U Aplano<br>2<br>FAM-NFQ-MGB<br>CT: 36.18	⚠1<br>2203 A01 10 saki<br>U Aplano<br>6.82<br>FAM-NFQ-MGB<br>CT: 34.39	2203 A07 10 saki<br>U Aplano<br>13.67<br>FAM-NFQ-MGB<br>CT: 33.38	2203 A07 10 saki<br>U Aplano<br>15.24<br>FAM-NFQ-MGB<br>CT: 33.22	2203 A07 10 saki<br>U Aplano<br>21.94<br>FAM-NFQ-MGB<br>CT: 32.69	2203 A11 SCM saki<br>U Aplano<br>109.99<br>FAM-NFQ-MGB<br>CT: 30.34	2203 A11 SCM saki<br>U Aplano<br>104.32<br>FAM-NFQ-MGB<br>CT: 30.42	2203 A11 SCM saki<br>U Aplano<br>121.8<br>FAM-NFQ-MGB<br>CT: 30.19	
B	S Aplano<br>2E5<br>FAM-NFQ-MGB<br>CT: 19.42	S Aplano<br>2E5<br>FAM-NFQ-MGB<br>CT: 19.08	2203 A01 SCM saki<br>U Aplano<br>5.51<br>FAM-NFQ-MGB<br>CT: 34.7	2203 A01 SCM saki<br>U Aplano<br>5.15<br>FAM-NFQ-MGB<br>CT: 34.8	2203 A01 SCM saki<br>U Aplano<br>5.74<br>FAM-NFQ-MGB<br>CT: 34.64	2203 A07 SCM saki<br>U Aplano<br>15.68<br>FAM-NFQ-MGB<br>CT: 33.18	2203 A07 SCM saki<br>U Aplano<br>17.84<br>FAM-NFQ-MGB<br>CT: 32.99	2203 A07 SCM saki<br>U Aplano<br>22.94<br>FAM-NFQ-MGB<br>CT: 32.62	2203 A11 200 saki<br>U Aplano<br>119.27<br>FAM-NFQ-MGB<br>CT: 30.22	2203 A11 200 saki<br>U Aplano<br>86.72<br>FAM-NFQ-MGB<br>CT: 30.69	2203 A11 200 saki<br>U Aplano<br>151.63<br>FAM-NFQ-MGB<br>CT: 29.87	
C	S Aplano<br>2E4<br>FAM-NFQ-MGB<br>CT: 22.54	S Aplano<br>2E4<br>FAM-NFQ-MGB<br>CT: 22.71	2203 A01 BTM saki<br>U Aplano<br>4.74<br>FAM-NFQ-MGB<br>CT: 34.92	2203 A01 BTM saki<br>U Aplano<br>7.48<br>FAM-NFQ-MGB<br>CT: 34.26	2203 A01 BTM saki<br>U Aplano<br>7.73<br>FAM-NFQ-MGB<br>CT: 34.21	⚠1<br>2203 A07 200 saki<br>U Aplano<br>3.04<br>FAM-NFQ-MGB<br>CT: 35.57	⚠1<br>2203 A07 200 saki<br>U Aplano<br>6.47<br>FAM-NFQ-MGB<br>CT: 34.47	⚠1<br>2203 A07 200 saki<br>U Aplano<br>13.18<br>FAM-NFQ-MGB<br>CT: 33.43	⚠1<br>2203 A11 1000 saki<br>U Aplano<br>1.13<br>FAM-NFQ-MGB<br>CT: 37.01	⚠2<br>2203 A11 1000 saki<br>U Aplano<br>FAM-NFQ-MGB<br>CT: Undetermined	⚠1<br>2203 A11 1000 saki<br>U Aplano<br>2.3<br>FAM-NFQ-MGB<br>CT: 35.98	
D	S Aplano<br>2E3<br>FAM-NFQ-MGB<br>CT: 25.87	S Aplano<br>2E3<br>FAM-NFQ-MGB<br>CT: 26.17	2203 A04 10 saki<br>U Aplano<br>2.38<br>FAM-NFQ-MGB<br>CT: 35.93	⚠2<br>2203 A04 10 saki<br>U Aplano<br>FAM-NFQ-MGB<br>CT: Undetermined	⚠2<br>2203 A04 10 saki<br>U Aplano<br>FAM-NFQ-MGB<br>CT: Undetermined	⚠2<br>2203 A07 1000 saki<br>U Aplano<br>FAM-NFQ-MGB<br>CT: Undetermined	2203 A07 1000 saki<br>U Aplano<br>FAM-NFQ-MGB<br>CT: Undetermined	⚠2<br>2203 A07 1000 saki<br>U Aplano<br>FAM-NFQ-MGB<br>CT: Undetermined	2203 A11 3000 saki<br>U Aplano<br>FAM-NFQ-MGB<br>CT: Undetermined	⚠2<br>2203 A11 3000 saki<br>U Aplano<br>FAM-NFQ-MGB<br>CT: Undetermined	⚠1<br>2203 A11 3000 saki<br>U Aplano<br>FAM-NFQ-MGB<br>CT: Undetermined	
E	S Aplano<br>200<br>FAM-NFQ-MGB<br>CT: 29.76	S Aplano<br>200<br>FAM-NFQ-MGB<br>CT: 29.88	⚠1<br>2203 A04 SCM saki<br>U Aplano<br>9.71<br>FAM-NFQ-MGB<br>CT: 33.88	⚠1<br>2203 A04 SCM saki<br>U Aplano<br>5.49<br>FAM-NFQ-MGB<br>CT: 34.71	⚠1<br>2203 A04 SCM saki<br>U Aplano<br>12.14<br>FAM-NFQ-MGB<br>CT: 33.55	⚠2<br>2203 A07 3000 saki<br>U Aplano<br>FAM-NFQ-MGB<br>CT: Undetermined	2203 A07 3000 saki<br>U Aplano<br>7.21<br>FAM-NFQ-MGB<br>CT: 34.31	⚠2<br>2203 A07 3000 saki<br>U Aplano<br>FAM-NFQ-MGB<br>CT: Undetermined	2203 A21 SCM saki<br>U Aplano<br>174.69<br>FAM-NFQ-MGB<br>CT: 29.67	2203 A21 SCM saki<br>U Aplano<br>169.79<br>FAM-NFQ-MGB<br>CT: 29.71	2203 A21 SCM saki<br>U Aplano<br>96.17<br>FAM-NFQ-MGB<br>CT: 30.54	
F	S Aplano<br>20<br>FAM-NFQ-MGB<br>CT: 32.79	S Aplano<br>20<br>FAM-NFQ-MGB<br>CT: 32.57	2203 A04 200 saki<br>U Aplano<br>2.29<br>FAM-NFQ-MGB<br>CT: 35.98	2203 A04 200 saki<br>U Aplano<br>1.93<br>FAM-NFQ-MGB<br>CT: 36.24	2203 A04 200 saki<br>U Aplano<br>2.66<br>FAM-NFQ-MGB<br>CT: 35.77				2203 A21 200 saki<br>U Aplano<br>33.05<br>FAM-NFQ-MGB<br>CT: 32.09	2203 A21 200 saki<br>U Aplano<br>60.19<br>FAM-NFQ-MGB<br>CT: 31.22	2203 A21 200 saki<br>U Aplano<br>43.47<br>FAM-NFQ-MGB<br>CT: 31.69	
G			⚠2<br>2203 A04 1000 saki<br>U Aplano<br>FAM-NFQ-MGB<br>CT: Undetermined	⚠2<br>2203 A04 1000 saki<br>U Aplano<br>FAM-NFQ-MGB<br>CT: Undetermined	⚠2<br>2203 A04 1000 saki<br>U Aplano<br>FAM-NFQ-MGB<br>CT: Undetermined				⚠1<br>2203 A21 1000 saki<br>U Aplano<br>FAM-NFQ-MGB<br>CT: Undetermined	⚠2<br>2203 A21 1000 saki<br>U Aplano<br>FAM-NFQ-MGB<br>CT: Undetermined	⚠1<br>2203 A21 1000 saki<br>U Aplano<br>FAM-NFQ-MGB<br>CT: Undetermined	N Aplano<br>FAM-NFQ-MGB<br>CT: Undetermined
H	Comf2E4<br>U Aplano<br>1.62E4<br>FAM-NFQ-MGB<br>CT: 23.06	Comf2E4<br>U Aplano<br>1.79E4<br>FAM-NFQ-MGB<br>CT: 22.92	⚠1<br>2203 A04 3000 saki<br>U Aplano<br>FAM-NFQ-MGB<br>CT: Undetermined	⚠1<br>2203 A04 3000 saki<br>U Aplano<br>FAM-NFQ-MGB<br>CT: Undetermined	⚠1<br>2203 A04 3000 saki<br>U Aplano<br>FAM-NFQ-MGB<br>CT: Undetermined				⚠1<br>2203 A21 3000 saki<br>U Aplano<br>FAM-NFQ-MGB<br>CT: Undetermined	⚠1<br>2203 A21 3000 saki<br>U Aplano<br>FAM-NFQ-MGB<br>CT: Undetermined	⚠1<br>2203 A21 3000 saki<br>U Aplano<br>FAM-NFQ-MGB<br>CT: Undetermined	N Aplano<br>FAM-NFQ-MGB<br>CT: Undetermined

Standard Curves

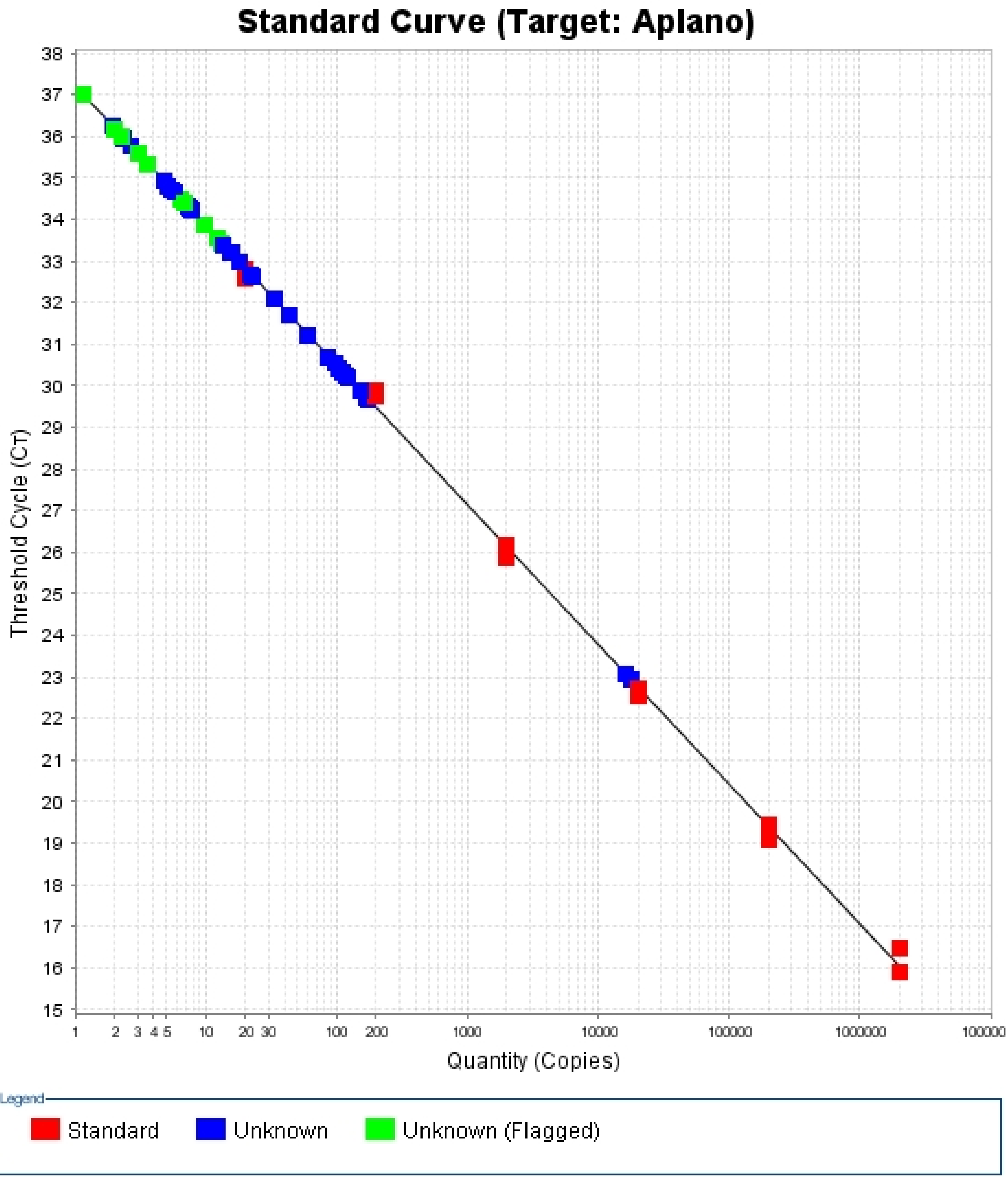

slope:-3.356 Y-Intercept:37.191 R^2:0.998 Eff%:98.585

Results Table

Well		Sample	Target	Task	Quantity	C
A1			Aplano	STANDARD	2000000	16.4913
A2			Aplano	STANDARD	2000000	15.9226
A3		2203 A01 10	Aplano	UNKNOWN	3.6145	35.3182
A4		2203 A01 10	Aplano	UNKNOWN	1.9993	36.1813
A5		2203 A01 10	Aplano	UNKNOWN	6.8168	34.3935
A6		2203 A07 10	Aplano	UNKNOWN	13.6709	33.3791
A7		2203 A07 10	Aplano	UNKNOWN	15.2408	33.2207
A8		2203 A07 10	Aplano	UNKNOWN	21.9378	32.6898
A9		2203 A11 SCM	Aplano	UNKNOWN	109.9893	30.3398
A10		2203 A11 SCM	Aplano	UNKNOWN	104.3207	30.4169
A11		2203 A11 SCM	Aplano	UNKNOWN	121.7982	30.1912
B1			Aplano	STANDARD	200000	19.4232
B2			Aplano	STANDARD	200000	19.0764
B3		2203 A01 SCM	Aplano	UNKNOWN	5.5081	34.7042
B4		2203 A01 SCM	Aplano	UNKNOWN	5.1509	34.8019
B5		2203 A01 SCM	Aplano	UNKNOWN	5.7366	34.6449
B6		2203 A07 SCM	Aplano	UNKNOWN	15.6834	33.1789
B7		2203 A07 SCM	Aplano	UNKNOWN	17.8402	32.9911
B8		2203 A07 SCM	Aplano	UNKNOWN	22.9429	32.6245
B9		2203 A11 200	Aplano	UNKNOWN	119.2703	30.2217
B10		2203 A11 200	Aplano	UNKNOWN	86.7171	30.6863
B11		2203 A11 200	Aplano	UNKNOWN	151.6329	29.8718
C1			Aplano	STANDARD	20000	22.537
C2			Aplano	STANDARD	20000	22.7095
C3		2203 A01 BTM	Aplano	UNKNOWN	4.74	34.9231
C4		2203 A01 BTM	Aplano	UNKNOWN	7.4825	34.2576
C5		2203 A01 BTM	Aplano	UNKNOWN	7.7266	34.2108
C6		2203 A07 200	Aplano	UNKNOWN	3.0418	35.5697
C7		2203 A07 200	Aplano	UNKNOWN	6.4678	34.47
C8		2203 A07 200	Aplano	UNKNOWN	13.1813	33.4323

Well		Sample	Target	Task	Quantity	C
C9		2203 A11 1000	Aplano	UNKNOWN	1.1348	37.0068
C10		2203 A11 1000	Aplano	UNKNOWN		Undetermined
C11		2203 A11 1000	Aplano	UNKNOWN	2.2999	35.9772
D1			Aplano	STANDARD	2000	25.8732
D2			Aplano	STANDARD	2000	26.1682
D3		2203 A04 10	Aplano	UNKNOWN	2.3826	35.9257
D4		2203 A04 10	Aplano	UNKNOWN		Undetermined
D5		2203 A04 10	Aplano	UNKNOWN		Undetermined
D6		2203 A07 1000	Aplano	UNKNOWN		Undetermined
D7		2203 A07 1000	Aplano	UNKNOWN		Undetermined
D8		2203 A07 1000	Aplano	UNKNOWN		Undetermined
D9		2203 A11 3000	Aplano	UNKNOWN		Undetermined
D10		2203 A11 3000	Aplano	UNKNOWN		Undetermined
D11		2203 A11 3000	Aplano	UNKNOWN		Undetermined
E1			Aplano	STANDARD	200	29.7616
E2			Aplano	STANDARD	200	29.8827
E3		2203 A04 SCM	Aplano	UNKNOWN	9.7136	33.8773
E4		2203 A04 SCM	Aplano	UNKNOWN	5.4926	34.7083
E5		2203 A04 SCM	Aplano	UNKNOWN	12.1368	33.5526
E6		2203 A07 3000	Aplano	UNKNOWN		Undetermined
E7		2203 A07 3000	Aplano	UNKNOWN	7.212	34.3113
E8		2203 A07 3000	Aplano	UNKNOWN		Undetermined
E9		2203 A21 SCM	Aplano	UNKNOWN	174.6907	29.6655
E10		2203 A21 SCM	Aplano	UNKNOWN	169.7903	29.7069
E11		2203 A21 SCM	Aplano	UNKNOWN	96.1728	30.5355
F1			Aplano	STANDARD	20	32.7894
F2			Aplano	STANDARD	20	32.5679
F3		2203 A04 200	Aplano	UNKNOWN	2.2904	35.9832
F4		2203 A04 200	Aplano	UNKNOWN	1.9264	36.2355
F5		2203 A04 200	Aplano	UNKNOWN	2.6602	35.7651
F9		2203 A21 200	Aplano	UNKNOWN	33.05	32.0924
F10		2203 A21 200	Aplano	UNKNOWN	60.1872	31.2187

Well		Sample	Target	Task	Quantity	C
F11		2203 A21 200	Aplano	UNKNOWN	43.4683	31.693
G3		2203 A04 1000	Aplano	UNKNOWN		Undetermined
G4		2203 A04 1000	Aplano	UNKNOWN		Undetermined
G5		2203 A04 1000	Aplano	UNKNOWN		Undetermined
G9		2203 A21 1000	Aplano	UNKNOWN		Undetermined
G10		2203 A21 1000	Aplano	UNKNOWN		Undetermined
G11		2203 A21 1000	Aplano	UNKNOWN		Undetermined
G12			Aplano	NTC		Undetermined
H1		Comf2E4	Aplano	UNKNOWN	16228.1074	23.0603
H2		Comf2E4	Aplano	UNKNOWN	17899.1387	22.9174
H3		2203 A04 3000	Aplano	UNKNOWN		Undetermined
H4		2203 A04 3000	Aplano	UNKNOWN		Undetermined
H5		2203 A04 3000	Aplano	UNKNOWN		Undetermined
H9		2203 A21 3000	Aplano	UNKNOWN		Undetermined
H10		2203 A21 3000	Aplano	UNKNOWN		Undetermined
H11		2203 A21 3000	Aplano	UNKNOWN		Undetermined
H12			Aplano	NTC		Undetermined

QC Summary

Total Wells	96	Processed Wells	79	Targets Used	1
Well Setup	79	Flagged Wells	32	Samples Used	22

Flag	Name	Frequency	Locations
AMPNC	Amplification in negative control	0	
BADROX	Bad passive reference signal	0	
BLFAIL	Baseline algorithm failed	0	
CTFAIL	C algorithm failed	0	
EXPFAIL	Exponential algorithm failed	21	C10, D4, D5, D6, D8, D10, D11, E6, E8, G3, G4, G5, G9, G10, G11, H3, H4, H5, H9, H10, H11
HIGHSD	High standard deviation in replicate group	11	A3, A4, A5, C6, C7, C8, C9, C11, E3, E4, E5
NOAMP	No amplification	12	C10, D4, D5, D6, D8, D10, E6, E8, G3, G4, G5, G10
NOISE	Noise higher than others in plate	0	
NOSIGNAL	No signal in well	0	
OFFSCALE	Fluorescence is offscale	0	
OUTLIERRG	Outlier in replicate group	0	
SPIKE	Noise spikes	0	
THOLDFAIL	Thresholding algorithm failed	0	
