# Supplementary material for: Ecological impact of algal-preying protists aplanochytrids (Labyrinthulea) as a source of docosahexaenoic acid for copepods in marine environments
Source: ISME Commun. 2026 Jun 26;6(1):ycag178. doi: 10.1093/ismeco/ycag178 (PMC13685785; doi:10.1093/ismeco/ycag178)
Supplement: Supplementary file 2 [file supplementary_materials_ycag178.zip › Morimoto_ISME_C_3rdrev_supplemental_data.zip!/Morimoto_ISME_C_3rdrev_supplemental_data/Supplemental_Data_2/Standard Curve/Aplanochytrid/2203/Aplano2203AlineN=3.pdf]

Experiment Results Report
230728Aplano2203AlineN=3

Experiment Summary

Experiment Name	: 230728Aplano2203AlineN=3
Experiment Type	: Quantitation - Standard Curve
File Name	: 230728Aplano2203AlineN=3.eds
Run Started	: 2023 Jul 28 6:32:28 PM
Run Finished	: 2023 Jul 28 8:10:24 PM
Run Duration	: 97 minutes 55 seconds
Date Modified	: 2023 Jul 28 10:39:43 PM
User	:
Number of wells used	: 77
Number of wells with results	: 77
Instrument Name	:
Instrument Type	: Applied Biosystems StepOnePlus™ Instrument
Comments	:

Results Summary

Sample	Target	Quantity (Mean)	Quantity (Std Dev)	C (Mean)	C (Std Dev)
2203 A01 10m saki3	Aplano	2.90	2.69	36.40	1.61
2203 A01 BTM saki3	Aplano	7.04	1.54	34.73	0.32
2203 A01 SCM saki3	Aplano	9.02		34.35	
2203 A04 1000m	Aplano				
2203 A04 10m saki3	Aplano	1611991040.00	2792050432.00	25.66	17.74
2203 A04 200m	Aplano	8464592384.00		4.37	
2203 A04 3000m	Aplano				
2203 A04 SCM saki3	Aplano	60.06	22.07	31.66	0.50
2203 A07 1000m	Aplano	4.60	3.01	35.59	1.14
2203 A07 10m saki3	Aplano	78.55	37.97	31.34	0.77
2203 A07 200m	Aplano	24.52	3.60	32.91	0.22
2203 A07 3000m	Aplano	2313438976.00		6.25	
2203 A07 SCM saki3	Aplano	18.71	5.89	33.34	0.43
2203 A11 1000m	Aplano	7.30		34.66	
2203 A11 200m	Aplano	244.36	45.56	29.58	0.28
2203 A11 3000m	Aplano				
2203 A11 SCM saki3	Aplano	162.27	26.00	30.17	0.24
2203 A21 1000m	Aplano	26.81		32.77	
2203 A21 200m	Aplano	336.93	54.31	29.11	0.24
2203 A21 3000m	Aplano	3.92		35.56	
2203 A21 SCM saki3	Aplano	376.40	73.45	28.95	0.29

Plate Layout

	1	2	3	4	5	6	7	8	9	10	11	12
A	S Aplano<br>2E6<br>FAM-NFQ-MGB<br>CT: 16.9	S Aplano<br>2E6<br>FAM-NFQ-MGB<br>CT: 16.75	⚠1<br>2203 A01 10m saki3<br>U Aplano<br>FAM-NFQ-MGB<br>CT: Undetermined	⚠1<br>2203 A01 10m saki3<br>U Aplano<br>4.8<br>FAM-NFQ-MGB<br>CT: 35.26	⚠1<br>2203 A01 10m saki3<br>U Aplano<br>1<br>FAM-NFQ-MGB<br>CT: 37.54	⚠1<br>2203 A07 10m saki3<br>U Aplano<br>41.02<br>FAM-NFQ-MGB<br>CT: 32.15	⚠1<br>2203 A07 10m saki3<br>U Aplano<br>116.95<br>FAM-NFQ-MGB<br>CT: 30.63	⚠1<br>2203 A07 10m saki3<br>U Aplano<br>77.68<br>FAM-NFQ-MGB<br>CT: 31.23	2203 A11 SCM saki3<br>U Aplano<br>184.55<br>FAM-NFQ-MGB<br>CT: 29.97	2203 A11 SCM saki3<br>U Aplano<br>133.71<br>FAM-NFQ-MGB<br>CT: 30.44	2203 A11 SCM saki3<br>U Aplano<br>168.55<br>FAM-NFQ-MGB<br>CT: 30.1	
B	⚠1<br>S Aplano<br>2E5<br>FAM-NFQ-MGB<br>CT: 18.95	⚠1<br>S Aplano<br>2E5<br>FAM-NFQ-MGB<br>CT: 19.89	⚠1<br>2203 A01 SCM saki3<br>U Aplano<br>FAM-NFQ-MGB<br>CT: Undetermined	⚠1<br>2203 A01 SCM saki3<br>U Aplano<br>FAM-NFQ-MGB<br>CT: Undetermined	2203 A01 SCM saki3<br>U Aplano<br>9.02<br>FAM-NFQ-MGB<br>CT: 34.35	2203 A07 SCM saki3<br>U Aplano<br>15.59<br>FAM-NFQ-MGB<br>CT: 33.56	2203 A07 SCM saki3<br>U Aplano<br>25.5<br>FAM-NFQ-MGB<br>CT: 32.84	2203 A07 SCM saki3<br>U Aplano<br>15.04<br>FAM-NFQ-MGB<br>CT: 33.61	2203 A11 200m saki3<br>U Aplano<br>285.77<br>FAM-NFQ-MGB<br>CT: 29.34	2203 A11 200m saki3<br>U Aplano<br>251.75<br>FAM-NFQ-MGB<br>CT: 29.52	2203 A11 200m saki3<br>U Aplano<br>195.55<br>FAM-NFQ-MGB<br>CT: 29.89	
C	S Aplano<br>2E4<br>FAM-NFQ-MGB<br>CT: 23.14	S Aplano<br>2E4<br>FAM-NFQ-MGB<br>CT: 22.88	2203 A01 BTM saki3<br>U Aplano<br>8.13<br>FAM-NFQ-MGB<br>CT: 34.5	2203 A01 BTM saki3<br>U Aplano<br>5.96<br>FAM-NFQ-MGB<br>CT: 34.95	⚠1<br>2203 A01 BTM saki3<br>U Aplano<br>FAM-NFQ-MGB<br>CT: Undetermined	2203 A07 200m saki3<br>U Aplano<br>25.6<br>FAM-NFQ-MGB<br>CT: 32.84	2203 A07 200m saki3<br>U Aplano<br>20.5<br>FAM-NFQ-MGB<br>CT: 33.16	2203 A07 200m saki3<br>U Aplano<br>27.46<br>FAM-NFQ-MGB<br>CT: 32.73	⚠1<br>2203 A11 1000m saki3<br>U Aplano<br>FAM-NFQ-MGB<br>CT: Undetermined	2203 A11 1000m saki3<br>U Aplano<br>7.3<br>FAM-NFQ-MGB<br>CT: 34.66	⚠1<br>2203 A11 1000m saki3<br>U Aplano<br>FAM-NFQ-MGB<br>CT: Undetermined	
D	S Aplano<br>2E3<br>FAM-NFQ-MGB<br>CT: 26.53	S Aplano<br>2E3<br>FAM-NFQ-MGB<br>CT: 26.54	⚠1<br>2203 A04 10m saki3<br>U Aplano<br>1.97<br>FAM-NFQ-MGB<br>CT: 36.55	⚠1<br>2203 A04 10m saki3<br>U Aplano<br>4.89<br>FAM-NFQ-MGB<br>CT: 35.24	⚠1<br>2203 A04 10m saki3<br>U Aplano<br>4.84E9<br>FAM-NFQ-MGB<br>CT: 5.18	⚠1<br>2203 A07 1000m saki3<br>U Aplano<br>7.65<br>FAM-NFQ-MGB<br>CT: 34.59	⚠1<br>2203 A07 1000m saki3<br>U Aplano<br>1.62<br>FAM-NFQ-MGB<br>CT: 36.84	⚠1<br>2203 A07 1000m saki3<br>U Aplano<br>4.52<br>FAM-NFQ-MGB<br>CT: 35.35	⚠1<br>2203 A11 3000m saki3<br>U Aplano<br>FAM-NFQ-MGB<br>CT: Undetermined	⚠2<br>2203 A11 3000m saki3<br>U Aplano<br>FAM-NFQ-MGB<br>CT: Undetermined	⚠1<br>2203 A11 3000m saki3<br>U Aplano<br>FAM-NFQ-MGB<br>CT: Undetermined	
E	S Aplano<br>200<br>FAM-NFQ-MGB<br>CT: 30.43	S Aplano<br>200<br>FAM-NFQ-MGB<br>CT: 30.02	⚠1<br>2203 A04 SCM saki3<br>U Aplano<br>50.25<br>FAM-NFQ-MGB<br>CT: 31.86	⚠1<br>2203 A04 SCM saki3<br>U Aplano<br>44.59<br>FAM-NFQ-MGB<br>CT: 32.03	⚠1<br>2203 A04 SCM saki3<br>U Aplano<br>85.33<br>FAM-NFQ-MGB<br>CT: 31.09	⚠1<br>2203 A07 3000m saki3<br>U Aplano<br>FAM-NFQ-MGB<br>CT: Undetermined	⚠1<br>2203 A07 3000m saki3<br>U Aplano<br>FAM-NFQ-MGB<br>CT: Undetermined	2203 A07 3000m saki3<br>U Aplano<br>2.31E9<br>FAM-NFQ-MGB<br>CT: 6.25	2203 A21 SCM saki3<br>U Aplano<br>301.53<br>FAM-NFQ-MGB<br>CT: 29.26	2203 A21 SCM saki3<br>U Aplano<br>448.34<br>FAM-NFQ-MGB<br>CT: 28.68	2203 A21 SCM saki3<br>U Aplano<br>379.33<br>FAM-NFQ-MGB<br>CT: 28.92	
F	S Aplano<br>20<br>FAM-NFQ-MGB<br>CT: 32.69	S Aplano<br>20<br>FAM-NFQ-MGB<br>CT: 33.37	⚠2<br>2203 A04 200m saki3<br>U Aplano<br>FAM-NFQ-MGB<br>CT: Undetermined	⚠1<br>2203 A04 200m saki3<br>U Aplano<br>FAM-NFQ-MGB<br>CT: Undetermined	2203 A04 200m saki3<br>U Aplano<br>8.46E9<br>FAM-NFQ-MGB<br>CT: 4.37				2203 A21 200m saki3<br>U Aplano<br>340.69<br>FAM-NFQ-MGB<br>CT: 29.08	2203 A21 200m saki3<br>U Aplano<br>389.26<br>FAM-NFQ-MGB<br>CT: 28.89	2203 A21 200m saki3<br>U Aplano<br>280.83<br>FAM-NFQ-MGB<br>CT: 29.36	
G			⚠1<br>2203 A04 1000m saki3<br>U Aplano<br>FAM-NFQ-MGB<br>CT: Undetermined	⚠1<br>2203 A04 1000m saki3<br>U Aplano<br>FAM-NFQ-MGB<br>CT: Undetermined	⚠2<br>2203 A04 1000m saki3<br>U Aplano<br>FAM-NFQ-MGB<br>CT: Undetermined				2203 A21 1000m saki3<br>U Aplano<br>26.81<br>FAM-NFQ-MGB<br>CT: 32.77	⚠2<br>2203 A21 1000m saki3<br>U Aplano<br>FAM-NFQ-MGB<br>CT: Undetermined	⚠1<br>2203 A21 1000m saki3<br>U Aplano<br>FAM-NFQ-MGB<br>CT: Undetermined	
H	N Aplano<br>FAM-NFQ-MGB<br>CT: Undetermined	N Aplano<br>FAM-NFQ-MGB<br>CT: Undetermined	⚠1<br>2203 A04 3000m saki3<br>U Aplano<br>FAM-NFQ-MGB<br>CT: Undetermined	⚠1<br>2203 A04 3000m saki3<br>U Aplano<br>FAM-NFQ-MGB<br>CT: Undetermined	⚠1<br>2203 A04 3000m saki3<br>U Aplano<br>FAM-NFQ-MGB<br>CT: Undetermined				2203 A21 3000m saki3<br>U Aplano<br>3.92<br>FAM-NFQ-MGB<br>CT: 35.56	⚠1<br>2203 A21 3000m saki3<br>U Aplano<br>FAM-NFQ-MGB<br>CT: Undetermined	⚠1<br>2203 A21 3000m saki3<br>U Aplano<br>FAM-NFQ-MGB<br>CT: Undetermined	

Standard Curves

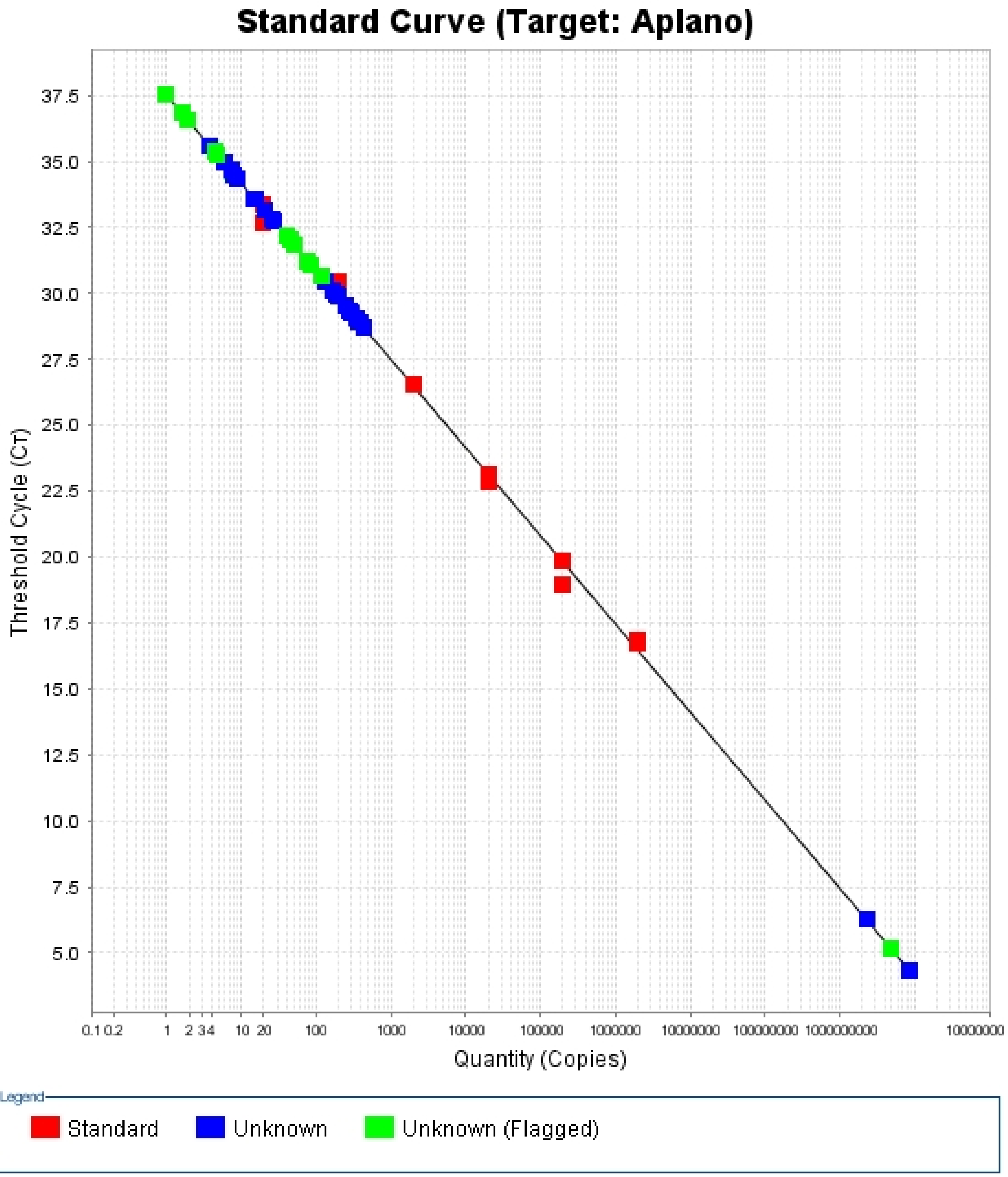

slope:-3.341 Y-Intercept:37.542 R^2:0.996 Eff%:99.198

Results Table

Well		Sample	Target	Task	Quantity	C
A1			Aplano	STANDARD	2000000	16.9013
A2			Aplano	STANDARD	2000000	16.749
A3		2203 A01 10m	Aplano	UNKNOWN		Undetermined
A4		2203 A01 10m	Aplano	UNKNOWN	4.8012	35.2649
A5		2203 A01 10m	Aplano	UNKNOWN	0.998	37.5444
A6		2203 A07 10m	Aplano	UNKNOWN	41.017	32.1521
A7		2203 A07 10m	Aplano	UNKNOWN	116.9512	30.6317
A8		2203 A07 10m	Aplano	UNKNOWN	77.6776	31.2255
A9		2203 A11 SCM	Aplano	UNKNOWN	184.554	29.9697
A10		2203 A11 SCM	Aplano	UNKNOWN	133.7089	30.4374
A11		2203 A11 SCM	Aplano	UNKNOWN	168.5473	30.1014
B1			Aplano	STANDARD	200000	18.9535
B2			Aplano	STANDARD	200000	19.8867
B3		2203 A01 SCM	Aplano	UNKNOWN		Undetermined
B4		2203 A01 SCM	Aplano	UNKNOWN		Undetermined
B5		2203 A01 SCM	Aplano	UNKNOWN	9.0154	34.3506
B6		2203 A07 SCM	Aplano	UNKNOWN	15.5873	33.5561
B7		2203 A07 SCM	Aplano	UNKNOWN	25.501	32.8418
B8		2203 A07 SCM	Aplano	UNKNOWN	15.0414	33.6078
B9		2203 A11 200m	Aplano	UNKNOWN	285.772	29.3352
B10		2203 A11 200m	Aplano	UNKNOWN	251.7531	29.5191
B11		2203 A11 200m	Aplano	UNKNOWN	195.5522	29.8857
C1			Aplano	STANDARD	20000	23.139
C2			Aplano	STANDARD	20000	22.883
C3		2203 A01 BTM	Aplano	UNKNOWN	8.131	34.5004
C4		2203 A01 BTM	Aplano	UNKNOWN	5.9576	34.9518
C5		2203 A01 BTM	Aplano	UNKNOWN		Undetermined
C6		2203 A07 200m	Aplano	UNKNOWN	25.6027	32.836
C7		2203 A07 200m	Aplano	UNKNOWN	20.5049	33.1582
C8		2203 A07 200m	Aplano	UNKNOWN	27.4572	32.7345

Well		Sample	Target	Task	Quantity	C
C9		2203 A11	Aplano	UNKNOWN		Undetermined
C10		2203 A11	Aplano	UNKNOWN	7.2979	34.6573
C11		2203 A11	Aplano	UNKNOWN		Undetermined
D1			Aplano	STANDARD	2000	26.5346
D2			Aplano	STANDARD	2000	26.5423
D3		2203 A04 10m	Aplano	UNKNOWN	1.9742	36.5545
D4		2203 A04 10m	Aplano	UNKNOWN	4.891	35.238
D5		2203 A04 10m	Aplano	UNKNOWN	4835973120	5.1827
D6		2203 A07	Aplano	UNKNOWN	7.6485	34.5892
D7		2203 A07	Aplano	UNKNOWN	1.6215	36.8401
D8		2203 A07	Aplano	UNKNOWN	4.5203	35.3524
D9		2203 A11	Aplano	UNKNOWN		Undetermined
D10		2203 A11	Aplano	UNKNOWN		Undetermined
D11		2203 A11	Aplano	UNKNOWN		Undetermined
E1			Aplano	STANDARD	200	30.4334
E2			Aplano	STANDARD	200	30.0155
E3		2203 A04 SCM	Aplano	UNKNOWN	50.2479	31.8576
E4		2203 A04 SCM	Aplano	UNKNOWN	44.5902	32.0309
E5		2203 A04 SCM	Aplano	UNKNOWN	85.3324	31.0891
E6		2203 A07	Aplano	UNKNOWN		Undetermined
E7		2203 A07	Aplano	UNKNOWN		Undetermined
E8		2203 A07	Aplano	UNKNOWN	2313438976	6.2527
E9		2203 A21 SCM	Aplano	UNKNOWN	301.5296	29.2573
E10		2203 A21 SCM	Aplano	UNKNOWN	448.338	28.6817
E11		2203 A21 SCM	Aplano	UNKNOWN	379.3308	28.9242
F1			Aplano	STANDARD	20	32.6858
F2			Aplano	STANDARD	20	33.3672
F3		2203 A04 200m	Aplano	UNKNOWN		Undetermined
F4		2203 A04 200m	Aplano	UNKNOWN		Undetermined
F5		2203 A04 200m	Aplano	UNKNOWN	8464592384	4.3704
F9		2203 A21 200m	Aplano	UNKNOWN	340.69	29.0801
F10		2203 A21 200m	Aplano	UNKNOWN	389.264	28.8867

Well		Sample	Target	Task	Quantity	C
F11		2203 A21 200m	Aplano	UNKNOWN	280.8319	29.3605
G3		2203 A04	Aplano	UNKNOWN		Undetermined
G4		2203 A04	Aplano	UNKNOWN		Undetermined
G5		2203 A04	Aplano	UNKNOWN		Undetermined
G9		2203 A21	Aplano	UNKNOWN	26.8102	32.7691
G10		2203 A21	Aplano	UNKNOWN		Undetermined
G11		2203 A21	Aplano	UNKNOWN		Undetermined
H1			Aplano	NTC		Undetermined
H2			Aplano	NTC		Undetermined
H3		2203 A04	Aplano	UNKNOWN		Undetermined
H4		2203 A04	Aplano	UNKNOWN		Undetermined
H5		2203 A04	Aplano	UNKNOWN		Undetermined
H9		2203 A21	Aplano	UNKNOWN	3.9171	35.5602
H10		2203 A21	Aplano	UNKNOWN		Undetermined
H11		2203 A21	Aplano	UNKNOWN		Undetermined

QC Summary

Total Wells	96	Processed Wells	77	Targets Used	1
Well Setup	77	Flagged Wells	39	Samples Used	21

Flag	Name	Frequency	Locations
AMPNC	Amplification in negative control	0	
BADROX	Bad passive reference signal	0	
BLFAIL	Baseline algorithm failed	0	
CTFAIL	C algorithm failed	0	
EXPFAIL	Exponential algorithm failed	22	A3, B3, B4, C5, C9, D9, D10, D11, E6, E7, F3, F4, G3, G4, G5, G10, G11, H3, H4, H5, H10, H11
HIGHSD	High standard deviation in replicate group	16	A4, A5, A6, A7, A8, B1, B2, D3, D4, D5, D6, D7, D8, E3, E4, E5
NOAMP	No amplification	5	C11, D10, F3, G5, G10
NOISE	Noise higher than others in plate	0	
NOSIGNAL	No signal in well	0	
OFFSCALE	Fluorescence is offscale	0	
OUTLIERRG	Outlier in replicate group	0	
SPIKE	Noise spikes	0	
THOLDFAIL	Thresholding algorithm failed	0	
