# Supplementary material for: Ecological impact of algal-preying protists aplanochytrids (Labyrinthulea) as a source of docosahexaenoic acid for copepods in marine environments
Source: ISME Commun. 2026 Jun 26;6(1):ycag178. doi: 10.1093/ismeco/ycag178 (PMC13685785; doi:10.1093/ismeco/ycag178)
Supplement: Supplementary file 2 [file supplementary_materials_ycag178.zip › Morimoto_ISME_C_3rdrev_supplemental_data.zip!/Morimoto_ISME_C_3rdrev_supplemental_data/Supplemental_Data_2/Standard Curve/Aplanochytrid/2203/Aplano2203AlineN=1.pdf]

Experiment Results Report

230612Aplano2203AlineN=1

Experiment Summary

Experiment Name	:	230612Aplano2203AlineN=1
Experiment Type	:	Quantitation - Standard Curve
File Name	:	230612Aplano2203AlineN=1.eds
Run Started	:	2023 Jun 12 4:47:36 PM
Run Finished	:	2023 Jun 12 6:23:04 PM
Run Duration	:	95 minutes 28 seconds
Date Modified	:	2023 Jun 13 3:02:49 PM
User	:	
Number of wells used	:	79
Number of wells with results	:	79
Instrument Name	:	
Instrument Type	:	Applied Biosystems StepOnePlus™ Instrument
Comments	:	zennkai keisoku sita 2203Aline no sannpuru ha N=2 bannme ni naru sannpurudesu . konkainomonoga honnrai no N=1 bannme

Results Summary

Sample	Target	Quantity (Mean)	Quantity (Std Dev)	C (Mean)	C (Std Dev)
2203 A01 10m saki1	Aplano	2.66	1.39	36.12	0.79
2203 A01 BTM saki1	Aplano	4.13		35.34	
2203 A01 SCM saki1	Aplano	1.50	1.50	37.30	1.39
2203 A04 1000m	Aplano	1.25		37.08	
2203 A04 10m saki1	Aplano	26.06	18.33	32.84	1.13
2203 A04 200m	Aplano	2.13		36.31	
2203 A04 3000m	Aplano	2.77	0.09	35.92	0.05
2203 A04 SCM saki1	Aplano	31.01	17.37	32.61	1.12
2203 A07 1000m	Aplano	3.44	1.29	35.66	0.56
2203 A07 10m saki1	Aplano	18.98	14.51	33.37	1.06
2203 A07 200m	Aplano	11.15	4.14	33.96	0.60
2203 A07 3000m	Aplano				
2203 A07 SCM saki1	Aplano	17.16	7.86	33.34	0.63
2203 A11 1000m	Aplano				
2203 A11 200m	Aplano	86.29	23.85	30.92	0.42
2203 A11 3000m	Aplano	3.54	1.13	35.61	0.45
2203 A11 SCM saki1	Aplano	154.57	38.44	30.05	0.36
2203 A21 1000m	Aplano	21.84	14.45	33.09	0.92
2203 A21 200m	Aplano	51.23	6.16	31.65	0.17
2203 A21 3000m	Aplano	4.85	4.84	35.61	1.82
2203 A21 SCM saki1	Aplano	267.40	11.81	29.22	0.07
Comf2E4	Aplano	16452.00	5033.20	23.21	0.46

Plate Layout

	1	2	3	4	5	6	7	8	9	10	11	12
A	S Aplano<br>2E6<br>FAM-NFQ-MGB<br>CT: 16.52	S Aplano<br>2E6<br>FAM-NFQ-MGB<br>CT: 15.88	⚠1<br>2203 A01 10m saki1<br>U Aplano<br>2.4<br>FAM-NFQ-MGB<br>CT: 36.14	⚠1<br>2203 A01 10m saki1<br>U Aplano<br>1.41<br>FAM-NFQ-MGB<br>CT: 36.91	⚠1<br>2203 A01 10m saki1<br>U Aplano<br>4.16<br>FAM-NFQ-MGB<br>CT: 35.33	⚠1<br>2203 A07 10m saki1<br>U Aplano<br>35.6<br>FAM-NFQ-MGB<br>CT: 32.18	⚠1<br>2203 A07 10m saki1<br>U Aplano<br>8.83<br>FAM-NFQ-MGB<br>CT: 34.22	⚠1<br>2203 A07 10m saki1<br>U Aplano<br>12.5<br>FAM-NFQ-MGB<br>CT: 33.71	2203 A11 SCM saki1<br>U Aplano<br>144.58<br>FAM-NFQ-MGB<br>CT: 30.12	2203 A11 SCM saki1<br>U Aplano<br>197.02<br>FAM-NFQ-MGB<br>CT: 29.67	2203 A11 SCM saki1<br>U Aplano<br>122.1<br>FAM-NFQ-MGB<br>CT: 30.37	
B	S Aplano<br>2E5<br>FAM-NFQ-MGB<br>CT: 19.81	S Aplano<br>2E5<br>FAM-NFQ-MGB<br>CT: 19.15	⚠1<br>2203 A01 SCM saki1<br>U Aplano<br>3.23<br>FAM-NFQ-MGB<br>CT: 35.7	⚠1<br>2203 A01 SCM saki1<br>U Aplano<br>0.6<br>FAM-NFQ-MGB<br>CT: 38.17	⚠1<br>2203 A01 SCM saki1<br>U Aplano<br>0.66<br>FAM-NFQ-MGB<br>CT: 38.04	⚠1<br>2203 A07 SCM saki1<br>U Aplano<br>26.12<br>FAM-NFQ-MGB<br>CT: 32.63	⚠1<br>2203 A07 SCM saki1<br>U Aplano<br>13.9<br>FAM-NFQ-MGB<br>CT: 33.56	⚠1<br>2203 A07 SCM saki1<br>U Aplano<br>11.46<br>FAM-NFQ-MGB<br>CT: 33.84	2203 A11 200m saki1<br>U Aplano<br>87.47<br>FAM-NFQ-MGB<br>CT: 30.86	2203 A11 200m saki1<br>U Aplano<br>61.87<br>FAM-NFQ-MGB<br>CT: 31.37	2203 A11 200m saki1<br>U Aplano<br>109.53<br>FAM-NFQ-MGB<br>CT: 30.53	
C	S Aplano<br>2E4<br>FAM-NFQ-MGB<br>CT: 22.79	S Aplano<br>2E4<br>FAM-NFQ-MGB<br>CT: 22.67	2203 A01 BTM saki1<br>U Aplano<br>4.13<br>FAM-NFQ-MGB<br>CT: 35.34	⚠2<br>2203 A01 BTM saki1<br>U Aplano<br>FAM-NFQ-MGB<br>CT: Undetermined	⚠2<br>2203 A01 BTM saki1<br>U Aplano<br>FAM-NFQ-MGB<br>CT: Undetermined	⚠1<br>2203 A07 200m saki1<br>U Aplano<br>11.7<br>FAM-NFQ-MGB<br>CT: 33.81	⚠1<br>2203 A07 200m saki1<br>U Aplano<br>14.99<br>FAM-NFQ-MGB<br>CT: 33.45	⚠1<br>2203 A07 200m saki1<br>U Aplano<br>6.77<br>FAM-NFQ-MGB<br>CT: 34.61	⚠2<br>2203 A11 1000m saki1<br>U Aplano<br>FAM-NFQ-MGB<br>CT: Undetermined	⚠2<br>2203 A11 1000m saki1<br>U Aplano<br>FAM-NFQ-MGB<br>CT: Undetermined	2203 A11 1000m saki1<br>U Aplano<br>FAM-NFQ-MGB<br>CT: Undetermined	
D	S Aplano<br>2E3<br>FAM-NFQ-MGB<br>CT: 26.37	S Aplano<br>2E3<br>FAM-NFQ-MGB<br>CT: 26.41	⚠1<br>2203 A04 10m saki1<br>U Aplano<br>13.1<br>FAM-NFQ-MGB<br>CT: 33.64	⚠1<br>2203 A04 10m saki1<br>U Aplano<br>FAM-NFQ-MGB<br>CT: Undetermined	⚠1<br>2203 A04 10m saki1<br>U Aplano<br>39.02<br>FAM-NFQ-MGB<br>CT: 32.04	⚠2<br>2203 A07 1000m saki1<br>U Aplano<br>FAM-NFQ-MGB<br>CT: Undetermined	⚠1<br>2203 A07 1000m saki1<br>U Aplano<br>2.53<br>FAM-NFQ-MGB<br>CT: 36.06	⚠1<br>2203 A07 1000m saki1<br>U Aplano<br>4.35<br>FAM-NFQ-MGB<br>CT: 35.26	2203 A11 3000m saki1<br>U Aplano<br>4.83<br>FAM-NFQ-MGB<br>CT: 35.11	2203 A11 3000m saki1<br>U Aplano<br>3.13<br>FAM-NFQ-MGB<br>CT: 35.74	2203 A11 3000m saki1<br>U Aplano<br>2.67<br>FAM-NFQ-MGB<br>CT: 35.98	
E	S Aplano<br>200<br>FAM-NFQ-MGB<br>CT: 29.65	S Aplano<br>200<br>FAM-NFQ-MGB<br>CT: 29.6	⚠1<br>2203 A04 SCM saki1<br>U Aplano<br>40.33<br>FAM-NFQ-MGB<br>CT: 31.99	⚠2<br>2203 A04 SCM saki1<br>U Aplano<br>10.97<br>FAM-NFQ-MGB<br>CT: 33.9	⚠1<br>2203 A04 SCM saki1<br>U Aplano<br>41.72<br>FAM-NFQ-MGB<br>CT: 31.94	⚠1<br>2203 A07 3000m saki1<br>U Aplano<br>FAM-NFQ-MGB<br>CT: Undetermined	⚠2<br>2203 A07 3000m saki1<br>U Aplano<br>FAM-NFQ-MGB<br>CT: Undetermined	⚠1<br>2203 A07 3000m saki1<br>U Aplano<br>FAM-NFQ-MGB<br>CT: Undetermined	2203 A21 SCM saki1<br>U Aplano<br>269.34<br>FAM-NFQ-MGB<br>CT: 29.21	2203 A21 SCM saki1<br>U Aplano<br>278.13<br>FAM-NFQ-MGB<br>CT: 29.16	2203 A21 SCM saki1<br>U Aplano<br>254.74<br>FAM-NFQ-MGB<br>CT: 29.29	
F	⚠1<br>S Aplano<br>20<br>FAM-NFQ-MGB<br>CT: 32.43	⚠1<br>S Aplano<br>20<br>FAM-NFQ-MGB<br>CT: 33.63	2203 A04 200m saki1<br>U Aplano<br>2.13<br>FAM-NFQ-MGB<br>CT: 36.31	⚠1<br>2203 A04 200m saki1<br>U Aplano<br>FAM-NFQ-MGB<br>CT: Undetermined	⚠1<br>2203 A04 200m saki1<br>U Aplano<br>FAM-NFQ-MGB<br>CT: Undetermined				2203 A21 200m saki1<br>U Aplano<br>58.3<br>FAM-NFQ-MGB<br>CT: 31.45	2203 A21 200m saki1<br>U Aplano<br>47.05<br>FAM-NFQ-MGB<br>CT: 31.77	2203 A21 200m saki1<br>U Aplano<br>48.34<br>FAM-NFQ-MGB<br>CT: 31.73	
G			⚠1<br>2203 A04 1000m saki1<br>U Aplano<br>FAM-NFQ-MGB<br>CT: Undetermined	2203 A04 1000m saki1<br>U Aplano<br>1.25<br>FAM-NFQ-MGB<br>CT: 37.08	⚠2<br>2203 A04 1000m saki1<br>U Aplano<br>FAM-NFQ-MGB<br>CT: Undetermined				⚠1<br>2203 A21 1000m saki1<br>U Aplano<br>15.86<br>FAM-NFQ-MGB<br>CT: 33.36	⚠1<br>2203 A21 1000m saki1<br>U Aplano<br>11.34<br>FAM-NFQ-MGB<br>CT: 33.85	⚠1<br>2203 A21 1000m saki1<br>U Aplano<br>38.33<br>FAM-NFQ-MGB<br>CT: 32.07	N Aplano<br>FAM-NFQ-MGB<br>CT: Undetermined
H	Comf2E4<br>U Aplano<br>1.29E4<br>FAM-NFQ-MGB<br>CT: 23.53	Comf2E4<br>U Aplano<br>2E4<br>FAM-NFQ-MGB<br>CT: 22.89	2203 A04 3000m saki1<br>U Aplano<br>2.83<br>FAM-NFQ-MGB<br>CT: 35.89	2203 A04 3000m saki1<br>U Aplano<br>2.71<br>FAM-NFQ-MGB<br>CT: 35.96	⚠1<br>2203 A04 3000m saki1<br>U Aplano<br>FAM-NFQ-MGB<br>CT: Undetermined				⚠1<br>2203 A21 3000m saki1<br>U Aplano<br>8.28<br>FAM-NFQ-MGB<br>CT: 34.32	⚠1<br>2203 A21 3000m saki1<br>U Aplano<br>1.43<br>FAM-NFQ-MGB<br>CT: 36.89	⚠1<br>2203 A21 3000m saki1<br>U Aplano<br>FAM-NFQ-MGB<br>CT: Undetermined	N Aplano<br>FAM-NFQ-MGB<br>CT: Undetermined

Standard Curves

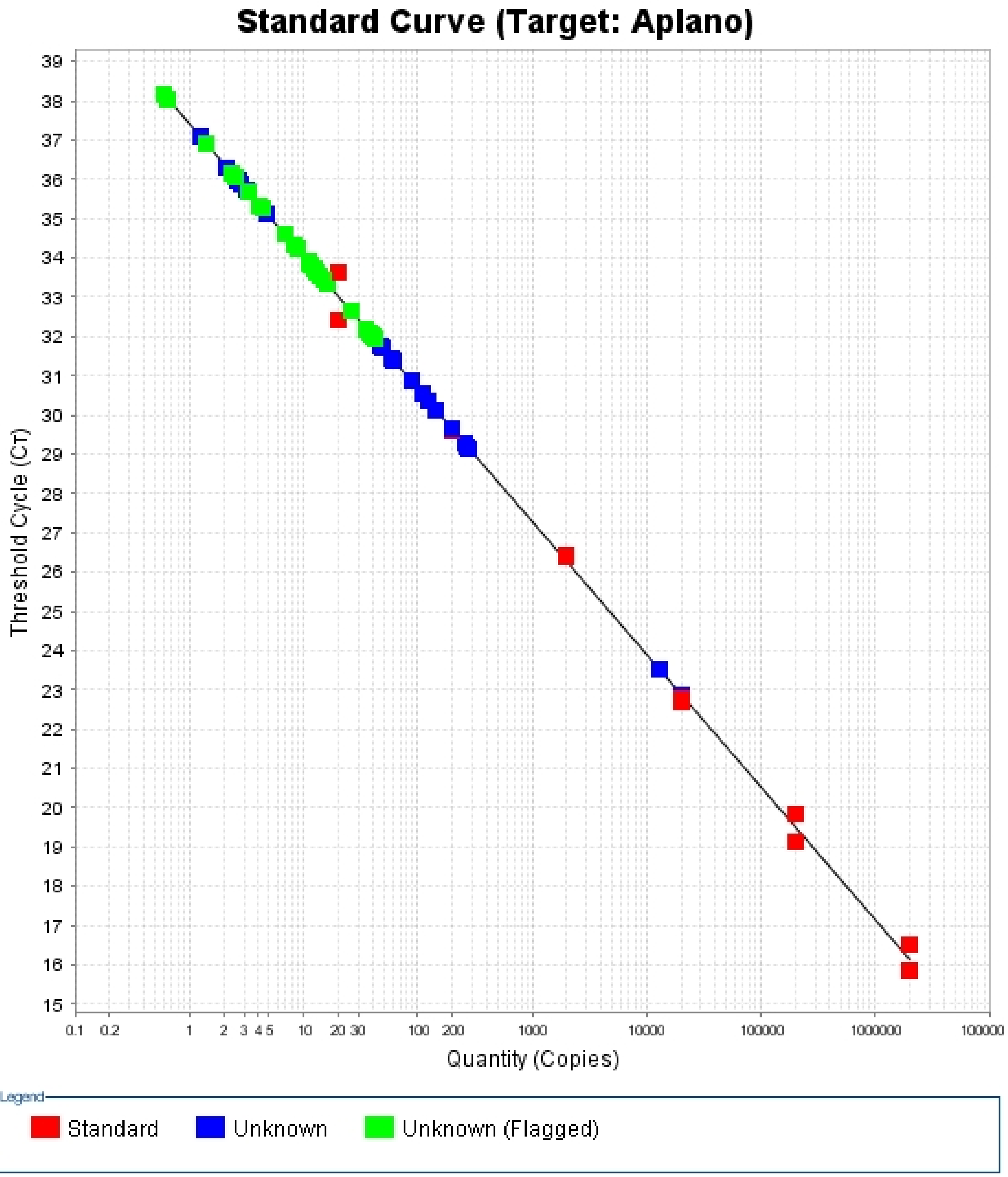

slope:-3.378 Y-Intercept:37.418 R^2:0.997 Eff%:97.696

Results Table

Well		Sample	Target	Task	Quantity	C
A1			Aplano	STANDARD	2000000	16.5194
A2			Aplano	STANDARD	2000000	15.8778
A3		2203 A01 10m	Aplano	UNKNOWN	2.3957	36.1357
A4		2203 A01 10m	Aplano	UNKNOWN	1.4132	36.9101
A5		2203 A01 10m	Aplano	UNKNOWN	4.1621	35.3253
A6		2203 A07 10m	Aplano	UNKNOWN	35.6004	32.1762
A7		2203 A07 10m	Aplano	UNKNOWN	8.8284	34.222
A8		2203 A07 10m	Aplano	UNKNOWN	12.4979	33.712
A9		2203 A11 SCM	Aplano	UNKNOWN	144.5796	30.1199
A10		2203 A11 SCM	Aplano	UNKNOWN	197.0174	29.6658
A11		2203 A11 SCM	Aplano	UNKNOWN	122.1002	30.3678
B1			Aplano	STANDARD	200000	19.8124
B2			Aplano	STANDARD	200000	19.152
B3		2203 A01 SCM	Aplano	UNKNOWN	3.2327	35.6961
B4		2203 A01 SCM	Aplano	UNKNOWN	0.5989	38.1698
B5		2203 A01 SCM	Aplano	UNKNOWN	0.655	38.0384
B6		2203 A07 SCM	Aplano	UNKNOWN	26.1201	32.6305
B7		2203 A07 SCM	Aplano	UNKNOWN	13.8961	33.5564
B8		2203 A07 SCM	Aplano	UNKNOWN	11.4584	33.8395
B9		2203 A11 200m	Aplano	UNKNOWN	87.467	30.8573
B10		2203 A11 200m	Aplano	UNKNOWN	61.8654	31.3654
B11		2203 A11 200m	Aplano	UNKNOWN	109.5251	30.5273
C1			Aplano	STANDARD	20000	22.7904
C2			Aplano	STANDARD	20000	22.6737
C3		2203 A01 BTM	Aplano	UNKNOWN	4.126	35.3381
C4		2203 A01 BTM	Aplano	UNKNOWN		Undetermined
C5		2203 A01 BTM	Aplano	UNKNOWN		Undetermined
C6		2203 A07 200m	Aplano	UNKNOWN	11.6995	33.8089
C7		2203 A07 200m	Aplano	UNKNOWN	14.9886	33.4454
C8		2203 A07 200m	Aplano	UNKNOWN	6.7656	34.6125

Well		Sample	Target	Task	Quantity	C
C9		2203 A11	Aplano	UNKNOWN		Undetermined
C10		2203 A11	Aplano	UNKNOWN		Undetermined
C11		2203 A11	Aplano	UNKNOWN		Undetermined
D1			Aplano	STANDARD	2000	26.3683
D2			Aplano	STANDARD	2000	26.4077
D3		2203 A04 10m	Aplano	UNKNOWN	13.0993	33.6431
D4		2203 A04 10m	Aplano	UNKNOWN		Undetermined
D5		2203 A04 10m	Aplano	UNKNOWN	39.0159	32.0417
D6		2203 A07	Aplano	UNKNOWN		Undetermined
D7		2203 A07	Aplano	UNKNOWN	2.5276	36.0571
D8		2203 A07	Aplano	UNKNOWN	4.3488	35.2609
D9		2203 A11	Aplano	UNKNOWN	4.8261	35.1082
D10		2203 A11	Aplano	UNKNOWN	3.1346	35.7413
D11		2203 A11	Aplano	UNKNOWN	2.6724	35.9754
E1			Aplano	STANDARD	200	29.6514
E2			Aplano	STANDARD	200	29.5985
E3		2203 A04 SCM	Aplano	UNKNOWN	40.3305	31.9931
E4		2203 A04 SCM	Aplano	UNKNOWN	10.9658	33.9039
E5		2203 A04 SCM	Aplano	UNKNOWN	41.7215	31.9434
E6		2203 A07	Aplano	UNKNOWN		Undetermined
E7		2203 A07	Aplano	UNKNOWN		Undetermined
E8		2203 A07	Aplano	UNKNOWN		Undetermined
E9		2203 A21 SCM	Aplano	UNKNOWN	269.3425	29.2071
E10		2203 A21 SCM	Aplano	UNKNOWN	278.1273	29.16
E11		2203 A21 SCM	Aplano	UNKNOWN	254.7417	29.2888
F1			Aplano	STANDARD	20	32.427
F2			Aplano	STANDARD	20	33.6349
F3		2203 A04 200m	Aplano	UNKNOWN	2.1324	36.3066
F4		2203 A04 200m	Aplano	UNKNOWN		Undetermined
F5		2203 A04 200m	Aplano	UNKNOWN		Undetermined
F9		2203 A21 200m	Aplano	UNKNOWN	58.3015	31.4524
F10		2203 A21 200m	Aplano	UNKNOWN	47.05	31.767

Well		Sample	Target	Task	Quantity	C
F11		2203 A21 200m	Aplano	UNKNOWN	48.3353	31.7275
G3		2203 A04	Aplano	UNKNOWN		Undetermined
G4		2203 A04	Aplano	UNKNOWN	1.2545	37.0849
G5		2203 A04	Aplano	UNKNOWN		Undetermined
G9		2203 A21	Aplano	UNKNOWN	15.8642	33.3621
G10		2203 A21	Aplano	UNKNOWN	11.343	33.8543
G11		2203 A21	Aplano	UNKNOWN	38.3262	32.0679
G12			Aplano	NTC		Undetermined
H1		Comf2E4	Aplano	UNKNOWN	12892.9893	23.5312
H2		Comf2E4	Aplano	UNKNOWN	20011.0156	22.8862
H3		2203 A04	Aplano	UNKNOWN	2.8342	35.8891
H4		2203 A04	Aplano	UNKNOWN	2.7091	35.9553
H5		2203 A04	Aplano	UNKNOWN		Undetermined
H9		2203 A21	Aplano	UNKNOWN	8.2756	34.3169
H10		2203 A21	Aplano	UNKNOWN	1.4297	36.8931
H11		2203 A21	Aplano	UNKNOWN		Undetermined
H12			Aplano	NTC		Undetermined

QC Summary

Total Wells	96	Processed Wells	79	Targets Used	1
Well Setup	79	Flagged Wells	44	Samples Used	22

Flag	Name	Frequency	Locations
AMPNC	Amplification in negative control	0	
BADROX	Bad passive reference signal	0	
BLFAIL	Baseline algorithm failed	0	
CTFAIL	C algorithm failed	0	
EXPFAIL	Exponential algorithm failed	15	C4, C5, C9, C10, D4, D6, E6, E7, E8, F4, F5, G3, G5, H5, H11
HIGHSD	High standard deviation in replicate group	29	A3, A4, A5, A6, A7, A8, B3, B4, B5, B6, B7, B8, C6, C7, C8, D3, D5, D7, D8, E3, E4, E5, F1, F2, G9, G10, G11, H9, H10
NOAMP	No amplification	7	C4, C5, C9, C10, D6, E7, G5
NOISE	Noise higher than others in plate	0	
NOSIGNAL	No signal in well	0	
OFFSCALE	Fluorescence is offscale	0	
OUTLIERRG	Outlier in replicate group	1	E4
SPIKE	Noise spikes	0	
THOLDFAIL	Thresholding algorithm failed	0	
