# Supplementary material for: Ecological impact of algal-preying protists aplanochytrids (Labyrinthulea) as a source of docosahexaenoic acid for copepods in marine environments
Source: ISME Commun. 2026 Jun 26;6(1):ycag178. doi: 10.1093/ismeco/ycag178 (PMC13685785; doi:10.1093/ismeco/ycag178)
Supplement: Supplementary file 2 [file supplementary_materials_ycag178.zip › Morimoto_ISME_C_3rdrev_supplemental_data.zip!/Morimoto_ISME_C_3rdrev_supplemental_data/Supplemental_Data_2/Standard Curve/Aplanochytrid/2107/Aplano2107AlineN=3-2kaime.pdf]

Experiment Results Report
230729APlano2107AlineN=3-2kaime

Experiment Summary

Experiment Name	:	230729APlano2107AlineN=3-2kaime
Experiment Type	:	Quantitation - Standard Curve
File Name	:	230729APlano2107AlineN=3-2kaime.eds
Run Started	:	2023 Jul 29 9:43:36 PM
Run Finished	:	2023 Jul 29 11:18:16 PM
Run Duration	:	94 minutes 40 seconds
Date Modified	:	2023 Jul 30 2:37:31 PM
User	:	
Number of wells used	:	80
Number of wells with results	:	80
Instrument Name	:	Local Instrument
Instrument Type	:	Applied Biosystems StepOnePlus™ Instrument
Comments	:	

Results Summary

Sample	Target	Quantity (Mean)	Quantity (Std Dev)	C (Mean)	C (Std Dev)
A01 10m saki	Aplano	58.99	3.70	33.67	0.09
A01 BTM saki	Aplano	100.55	22.90	32.90	0.37
A01 SCM saki	Aplano	74.18	15.33	33.35	0.32
A04 1000m saki	Aplano	3.00		38.13	
A04 10m saki	Aplano	2926.82	97.85	27.82	0.05
A04 200m saki	Aplano	23.94	16.71	35.46	1.61
A04 3000m saki	Aplano	8.03		36.66	
A04 SCM saki	Aplano	4908.96	112.82	27.04	0.03
A07 1000m saki	Aplano				
A07 200m saki	Aplano	111.18	52.67	32.84	0.73
A07 3000m saki	Aplano				
A07 SCM saki	Aplano	7937.40	174.88	26.32	0.03
A11 1000m saki	Aplano				
A11 10m saki	Aplano	174.37	33.44	32.06	0.28
A11 200m saki	Aplano	34.73	16.47	34.58	0.73
A11 3000m saki	Aplano	7.16	7.24	37.32	1.43
A11 SCM saki	Aplano	174.21	22.89	32.06	0.20
A21 1000m saki	Aplano				
A21 10m saki	Aplano	382.10	23.15	30.87	0.09
A21 200m saki	Aplano	25.31	0.97	34.94	0.06
A21 3000m saki	Aplano	1.67		39.01	
A21 SCM saki	Aplano	228.91	23.98	31.64	0.16

Plate Layout

	1	2	3	4	5	6	7	8	9	10	11	12
A	S Aplano<br>2E6<br>FAM-NFQ-MGB<br>CT: 18.41	S Aplano<br>2E6<br>FAM-NFQ-MGB<br>CT: 17.97	A01 10m saki<br>U Aplano<br>62.71<br>FAM-NFQ-MGB<br>CT: 33.58	A01 10m saki<br>U Aplano<br>58.95<br>FAM-NFQ-MGB<br>CT: 33.67	A01 10m saki<br>U Aplano<br>55.3<br>FAM-NFQ-MGB<br>CT: 33.77	A07 SCM saki<br>U Aplano<br>7.74E3<br>FAM-NFQ-MGB<br>CT: 26.36	A07 SCM saki<br>U Aplano<br>8E3<br>FAM-NFQ-MGB<br>CT: 26.31	A07 SCM saki<br>U Aplano<br>8.07E3<br>FAM-NFQ-MGB<br>CT: 26.3	⚠1<br>A11 3000m saki<br>U Aplano<br>3.08<br>FAM-NFQ-MGB<br>CT: 38.09	⚠1<br>A11 3000m saki<br>U Aplano<br>15.53<br>FAM-NFQ-MGB<br>CT: 35.67	⚠1<br>A11 3000m saki<br>U Aplano<br>2.88<br>FAM-NFQ-MGB<br>CT: 38.2	
B	S Aplano<br>2E5<br>FAM-NFQ-MGB<br>CT: 21.37	S Aplano<br>2E5<br>FAM-NFQ-MGB<br>CT: 21.41	A01 SCM saki<br>U Aplano<br>75.86<br>FAM-NFQ-MGB<br>CT: 33.29	A01 SCM saki<br>U Aplano<br>88.6<br>FAM-NFQ-MGB<br>CT: 33.06	A01 SCM saki<br>U Aplano<br>58.08<br>FAM-NFQ-MGB<br>CT: 33.69	⚠1<br>A07 200m saki<br>U Aplano<br>102.83<br>FAM-NFQ-MGB<br>CT: 32.84	⚠1<br>A07 200m saki<br>U Aplano<br>63.18<br>FAM-NFQ-MGB<br>CT: 33.57	⚠1<br>A07 200m saki<br>U Aplano<br>167.53<br>FAM-NFQ-MGB<br>CT: 32.11	A21 10m saki<br>U Aplano<br>407.7<br>FAM-NFQ-MGB<br>CT: 30.77	A21 10m saki<br>U Aplano<br>362.65<br>FAM-NFQ-MGB<br>CT: 30.95	A21 10m saki<br>U Aplano<br>375.96<br>FAM-NFQ-MGB<br>CT: 30.89	
C	S Aplano<br>2E4<br>FAM-NFQ-MGB<br>CT: 24.99	S Aplano<br>2E4<br>FAM-NFQ-MGB<br>CT: 24.82	A01 BTM saki<br>U Aplano<br>114.89<br>FAM-NFQ-MGB<br>CT: 32.67	A01 BTM saki<br>U Aplano<br>74.14<br>FAM-NFQ-MGB<br>CT: 33.33	A01 BTM saki<br>U Aplano<br>112.63<br>FAM-NFQ-MGB<br>CT: 32.7	⚠1<br>A07 1000m saki<br>U Aplano<br>FAM-NFQ-MGB<br>CT: Undetermined	⚠1<br>A07 1000m saki<br>U Aplano<br>FAM-NFQ-MGB<br>CT: Undetermined	⚠2<br>A07 1000m saki<br>U Aplano<br>FAM-NFQ-MGB<br>CT: Undetermined	A21 SCM saki<br>U Aplano<br>204.01<br>FAM-NFQ-MGB<br>CT: 31.81	A21 SCM saki<br>U Aplano<br>251.86<br>FAM-NFQ-MGB<br>CT: 31.5	A21 SCM saki<br>U Aplano<br>230.84<br>FAM-NFQ-MGB<br>CT: 31.63	
D	S Aplano<br>2E3<br>FAM-NFQ-MGB<br>CT: 28.35	S Aplano<br>2E3<br>FAM-NFQ-MGB<br>CT: 28.23	A04 10m saki<br>U Aplano<br>2.87E3<br>FAM-NFQ-MGB<br>CT: 27.85	A04 10m saki<br>U Aplano<br>3.04E3<br>FAM-NFQ-MGB<br>CT: 27.76	A04 10m saki<br>U Aplano<br>2.87E3<br>FAM-NFQ-MGB<br>CT: 27.85	⚠1<br>A07 3000m saki<br>U Aplano<br>FAM-NFQ-MGB<br>CT: Undetermined	⚠2<br>A07 3000m saki<br>U Aplano<br>FAM-NFQ-MGB<br>CT: Undetermined	⚠2<br>A07 3000m saki<br>U Aplano<br>FAM-NFQ-MGB<br>CT: Undetermined	A21 200m saki<br>U Aplano<br>26.35<br>FAM-NFQ-MGB<br>CT: 34.88	A21 200m saki<br>U Aplano<br>24.43<br>FAM-NFQ-MGB<br>CT: 34.99	A21 200m saki<br>U Aplano<br>25.13<br>FAM-NFQ-MGB<br>CT: 34.95	
E	S Aplano<br>200<br>FAM-NFQ-MGB<br>CT: 31.85	S Aplano<br>200<br>FAM-NFQ-MGB<br>CT: 31.63	A04 SCM saki<br>U Aplano<br>4.99E3<br>FAM-NFQ-MGB<br>CT: 27.02	A04 SCM saki<br>U Aplano<br>4.78E3<br>FAM-NFQ-MGB<br>CT: 27.08	A04 SCM saki<br>U Aplano<br>4.96E3<br>FAM-NFQ-MGB<br>CT: 27.03	A11 10m saki<br>U Aplano<br>159.91<br>FAM-NFQ-MGB<br>CT: 32.18	A11 10m saki<br>U Aplano<br>212.61<br>FAM-NFQ-MGB<br>CT: 31.75	A11 10m saki<br>U Aplano<br>150.59<br>FAM-NFQ-MGB<br>CT: 32.27	⚠1<br>A21 1000m saki<br>U Aplano<br>FAM-NFQ-MGB<br>CT: Undetermined	⚠2<br>A21 1000m saki<br>U Aplano<br>FAM-NFQ-MGB<br>CT: Undetermined	⚠1<br>A21 1000m saki<br>U Aplano<br>FAM-NFQ-MGB<br>CT: Undetermined	
F	⚠1<br>S Aplano<br>20<br>FAM-NFQ-MGB<br>CT: 34.87	⚠1<br>S Aplano<br>20<br>FAM-NFQ-MGB<br>CT: 36.07	⚠1<br>A04 200m saki<br>U Aplano<br>29.22<br>FAM-NFQ-MGB<br>CT: 34.72	⚠1<br>A04 200m saki<br>U Aplano<br>37.38<br>FAM-NFQ-MGB<br>CT: 34.35	⚠1<br>A04 200m saki<br>U Aplano<br>5.23<br>FAM-NFQ-MGB<br>CT: 37.3	A11 SCM saki<br>U Aplano<br>150.67<br>FAM-NFQ-MGB<br>CT: 32.27	A11 SCM saki<br>U Aplano<br>196.39<br>FAM-NFQ-MGB<br>CT: 31.87	A11 SCM saki<br>U Aplano<br>175.56<br>FAM-NFQ-MGB<br>CT: 32.04	A21 3000m saki<br>U Aplano<br>1.67<br>FAM-NFQ-MGB<br>CT: 39.01	⚠2<br>A21 3000m saki<br>U Aplano<br>FAM-NFQ-MGB<br>CT: Undetermined	⚠2<br>A21 3000m saki<br>U Aplano<br>FAM-NFQ-MGB<br>CT: Undetermined	
G			⚠2<br>A04 1000m saki<br>U Aplano<br>FAM-NFQ-MGB<br>CT: Undetermined	⚠1<br>A04 1000m saki<br>U Aplano<br>FAM-NFQ-MGB<br>CT: Undetermined	A04 1000m saki<br>U Aplano<br>3<br>FAM-NFQ-MGB<br>CT: 38.13	⚠1<br>A11 200m saki<br>U Aplano<br>52.42<br>FAM-NFQ-MGB<br>CT: 33.85	⚠1<br>A11 200m saki<br>U Aplano<br>19.84<br>FAM-NFQ-MGB<br>CT: 35.3	⚠1<br>A11 200m saki<br>U Aplano<br>31.93<br>FAM-NFQ-MGB<br>CT: 34.59				
H	N Aplano<br>FAM-NFQ-MGB<br>CT: Undetermined	N Aplano<br>FAM-NFQ-MGB<br>CT: Undetermined	A04 3000m saki<br>U Aplano<br>8.03<br>FAM-NFQ-MGB<br>CT: 36.66	⚠1<br>A04 3000m saki<br>U Aplano<br>FAM-NFQ-MGB<br>CT: Undetermined	⚠1<br>A04 3000m saki<br>U Aplano<br>FAM-NFQ-MGB<br>CT: Undetermined	⚠1<br>A11 1000m saki<br>U Aplano<br>FAM-NFQ-MGB<br>CT: Undetermined	⚠1<br>A11 1000m saki<br>U Aplano<br>FAM-NFQ-MGB<br>CT: Undetermined	⚠1<br>A11 1000m saki<br>U Aplano<br>FAM-NFQ-MGB<br>CT: Undetermined				

Standard Curves

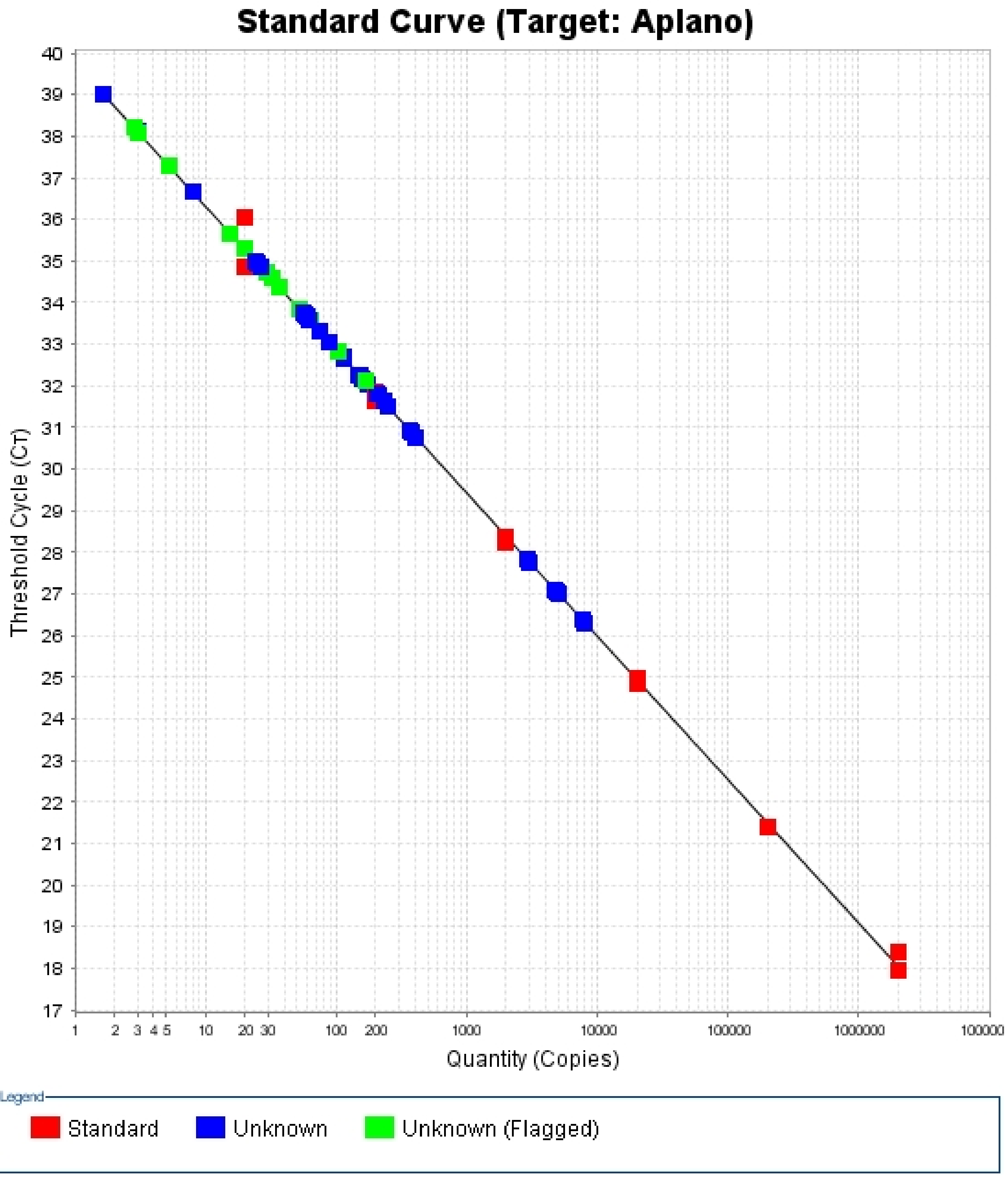

slope:-3.451 Y-Intercept:39.782 R^2:0.998 Eff%:94.874

Results Table

Well		Sample	Target	Task	Quantity	C
A1			Aplano	STANDARD	2000000	18.4132
A2			Aplano	STANDARD	2000000	17.9722
A3		A01 10m saki	Aplano	UNKNOWN	62.7104	33.5789
A4		A01 10m saki	Aplano	UNKNOWN	58.9531	33.6715
A5		A01 10m saki	Aplano	UNKNOWN	55.3013	33.7674
A6		A07 SCM saki	Aplano	UNKNOWN	7739.4526	26.3612
A7		A07 SCM saki	Aplano	UNKNOWN	8001.792	26.3112
A8		A07 SCM saki	Aplano	UNKNOWN	8070.9546	26.2983
A9		A11 3000m saki	Aplano	UNKNOWN	3.083	38.0943
A10		A11 3000m saki	Aplano	UNKNOWN	15.5274	35.6712
A11		A11 3000m saki	Aplano	UNKNOWN	2.8826	38.1951
B1			Aplano	STANDARD	200000	21.3737
B2			Aplano	STANDARD	200000	21.4112
B3		A01 SCM saki	Aplano	UNKNOWN	75.8587	33.2936
B4		A01 SCM saki	Aplano	UNKNOWN	88.6007	33.0609
B5		A01 SCM saki	Aplano	UNKNOWN	58.0829	33.6938
B6		A07 200m saki	Aplano	UNKNOWN	102.8325	32.8376
B7		A07 200m saki	Aplano	UNKNOWN	63.1847	33.5676
B8		A07 200m saki	Aplano	UNKNOWN	167.5268	32.1061
B9		A21 10m saki	Aplano	UNKNOWN	407.7005	30.7731
B10		A21 10m saki	Aplano	UNKNOWN	362.6498	30.9486
B11		A21 10m saki	Aplano	UNKNOWN	375.958	30.8946
C1			Aplano	STANDARD	20000	24.9851
C2			Aplano	STANDARD	20000	24.821
C3		A01 BTM saki	Aplano	UNKNOWN	114.8878	32.6715
C4		A01 BTM saki	Aplano	UNKNOWN	74.1367	33.328
C5		A01 BTM saki	Aplano	UNKNOWN	112.629	32.7012
C6		A07 1000m saki	Aplano	UNKNOWN		Undetermined
C7		A07 1000m saki	Aplano	UNKNOWN		Undetermined
C8		A07 1000m saki	Aplano	UNKNOWN		Undetermined

Well	Sample	Target	Task	Quantity	C
C9	A21 SCM saki	Aplano	UNKNOWN	204.0128	31.8108
C10	A21 SCM saki	Aplano	UNKNOWN	251.8628	31.495
C11	A21 SCM saki	Aplano	UNKNOWN	230.8409	31.6256
D1		Aplano	STANDARD	2000	28.3489
D2		Aplano	STANDARD	2000	28.2302
D3	A04 10m saki	Aplano	UNKNOWN	2873.9165	27.846
D4	A04 10m saki	Aplano	UNKNOWN	3039.7334	27.7619
D5	A04 10m saki	Aplano	UNKNOWN	2866.7974	27.8497
D6	A07 3000m saki	Aplano	UNKNOWN		Undetermined
D7	A07 3000m saki	Aplano	UNKNOWN		Undetermined
D8	A07 3000m saki	Aplano	UNKNOWN		Undetermined
D9	A21 200m saki	Aplano	UNKNOWN	26.3517	34.8784
D10	A21 200m saki	Aplano	UNKNOWN	24.4344	34.9916
D11	A21 200m saki	Aplano	UNKNOWN	25.1325	34.9494
E1		Aplano	STANDARD	200	31.8512
E2		Aplano	STANDARD	200	31.6272
E3	A04 SCM saki	Aplano	UNKNOWN	4985.833	27.0203
E4	A04 SCM saki	Aplano	UNKNOWN	4779.4419	27.0836
E5	A04 SCM saki	Aplano	UNKNOWN	4961.5918	27.0276
E6	A11 10m saki	Aplano	UNKNOWN	159.9145	32.1758
E7	A11 10m saki	Aplano	UNKNOWN	212.6119	31.7489
E8	A11 10m saki	Aplano	UNKNOWN	150.5871	32.2659
E9	A21 1000m saki	Aplano	UNKNOWN		Undetermined
E10	A21 1000m saki	Aplano	UNKNOWN		Undetermined
E11	A21 1000m saki	Aplano	UNKNOWN		Undetermined
F1		Aplano	STANDARD	20	34.8665
F2		Aplano	STANDARD	20	36.0652
F3	A04 200m saki	Aplano	UNKNOWN	29.2199	34.7235
F4	A04 200m saki	Aplano	UNKNOWN	37.3836	34.3543
F5	A04 200m saki	Aplano	UNKNOWN	5.2304	37.3021
F6	A11 SCM saki	Aplano	UNKNOWN	150.6676	32.2651
F7	A11 SCM saki	Aplano	UNKNOWN	196.3949	31.8678

Well		Sample	Target	Task	Quantity	C
F8		A11 SCM saki	Aplano	UNKNOWN	175.564	32.0359
F9		A21 3000m saki	Aplano	UNKNOWN	1.6731	39.0105
F10		A21 3000m saki	Aplano	UNKNOWN		Undetermined
F11		A21 3000m saki	Aplano	UNKNOWN		Undetermined
G3		A04 1000m saki	Aplano	UNKNOWN		Undetermined
G4		A04 1000m saki	Aplano	UNKNOWN		Undetermined
G5		A04 1000m saki	Aplano	UNKNOWN	3.0045	38.133
G6		A11 200m saki	Aplano	UNKNOWN	52.4233	33.8475
G7		A11 200m saki	Aplano	UNKNOWN	19.8368	35.3041
G8		A11 200m saki	Aplano	UNKNOWN	31.9309	34.5906
H1			Aplano	NTC		Undetermined
H2			Aplano	NTC		Undetermined
H3		A04 3000m saki	Aplano	UNKNOWN	8.0274	36.66
H4		A04 3000m saki	Aplano	UNKNOWN		Undetermined
H5		A04 3000m saki	Aplano	UNKNOWN		Undetermined
H6		A11 1000m saki	Aplano	UNKNOWN		Undetermined
H7		A11 1000m saki	Aplano	UNKNOWN		Undetermined
H8		A11 1000m saki	Aplano	UNKNOWN		Undetermined

QC Summary

Total Wells	96	Processed Wells	80	Targets Used	1
Well Setup	80	Flagged Wells	32	Samples Used	22

Flag	Name	Frequency	Locations
AMPNC	Amplification in negative control	0	
BADROX	Bad passive reference signal	0	
BLFAIL	Baseline algorithm failed	0	
CTFAIL	C algorithm failed	0	
EXPFAIL	Exponential algorithm failed	16	C6, C7, C8, D6, D7, D8, E10, F10, F11, G3, G4, H4, H5, H6, H7, H8
HIGHSD	High standard deviation in replicate group	14	A9, A10, A11, B6, B7, B8, F1, F2, F3, F4, F5, G6, G7, G8
NOAMP	No amplification	9	C8, D7, D8, E9, E10, E11, F10, F11, G3
NOISE	Noise higher than others in plate	0	
NOSIGNAL	No signal in well	0	
OFFSCALE	Fluorescence is offscale	0	
OUTLIERRG	Outlier in replicate group	0	
SPIKE	Noise spikes	0	
THOLDFAIL	Thresholding algorithm failed	0	
