# Supplementary material for: Ecological impact of algal-preying protists aplanochytrids (Labyrinthulea) as a source of docosahexaenoic acid for copepods in marine environments
Source: ISME Commun. 2026 Jun 26;6(1):ycag178. doi: 10.1093/ismeco/ycag178 (PMC13685785; doi:10.1093/ismeco/ycag178)
Supplement: Supplementary file 2 [file supplementary_materials_ycag178.zip › Morimoto_ISME_C_3rdrev_supplemental_data.zip!/Morimoto_ISME_C_3rdrev_supplemental_data/Supplemental_Data_2/Standard Curve/Aplanochytrid/2107/Aplano2107AlineN=1.pdf]

Experiment Results Report
230725Aplano2107AlineN=1

Experiment Summary

Experiment Name	:	230725Aplano2107AlineN=1
Experiment Type	:	Quantitation - Standard Curve
File Name	:	230725Aplano2107AlineN=1.eds
Run Started	:	2023 Jul 25 12:44:25 PM
Run Finished	:	2023 Jul 25 2:23:31 PM
Run Duration	:	99 minutes 6 seconds
Date Modified	:	2023 Jul 25 6:03:51 PM
User	:	
Number of wells used	:	77
Number of wells with results	:	77
Instrument Name	:	Local Instrument
Instrument Type	:	Applied Biosystems StepOnePlus™ Instrument
Comments	:	

Results Summary

Sample	Target	Quantity (Mean)	Quantity (Std Dev)	C (Mean)	C (Std Dev)
A01 10m saki	Aplano	55.26	9.06	33.49	0.27
A01 BTM saki	Aplano	406.40	52.32	30.38	0.20
A01 SCM saki	Aplano	114.88	36.54	32.39	0.50
A04 1000m saki	Aplano	6.02	0.46	36.91	0.12
A04 10m saki	Aplano	6054.90	797.60	26.19	0.20
A04 200m saki	Aplano	193.27	60.36	31.58	0.48
A04 3000m saki	Aplano	17.43	8.53	35.36	0.79
A07 1000m saki	Aplano	28.61	13.66	34.62	0.79
A07 200m saki	Aplano	284.74	41.41	30.94	0.22
A07 3000m saki	Aplano	13.20	7.11	35.85	0.88
A07 SCM saki	Aplano	17117.09	4334.65	24.60	0.38
A11 1000m saki	Aplano				
A11 10m saki	Aplano	937.30	134.43	29.09	0.22
A11 200m saki	Aplano	156.38	51.73	31.92	0.58
A11 3000m saki	Aplano	10.92	5.12	36.10	0.73
A11 SCM saki	Aplano	603.67	141.33	29.79	0.35
A21 1000m saki	Aplano	16126.67	22797.41	30.19	9.35
A21 10m saki	Aplano	631.85	59.65	29.69	0.15
A21 200m saki	Aplano	63.45	25.17	33.33	0.57
A21 3000m saki	Aplano	13.85	2.80	35.63	0.32
A21 SCM saki	Aplano	458.60	19.41	30.19	0.07

Plate Layout

	1	2	3	4	5	6
A	A01 10m saki<br>U Aplano<br>60.19<br>FAM-NFQ-MGB<br>CT: 33.34	A01 10m saki<br>U Aplano<br>60.78<br>FAM-NFQ-MGB<br>CT: 33.32	A01 10m saki<br>U Aplano<br>44.8<br>FAM-NFQ-MGB<br>CT: 33.8	S Aplano<br>2E6<br>FAM-NFQ-MGB<br>CT: 17.46	S Aplano<br>2E6<br>FAM-NFQ-MGB<br>CT: 17.23	A07 SCM saki<br>U Aplano<br>2.19E4<br>FAM-NFQ-MGB<br>CT: 24.18
B	1<br>A01 SCM saki<br>U Aplano<br>80.15<br>FAM-NFQ-MGB<br>CT: 32.89	1<br>A01 SCM saki<br>U Aplano<br>111.5<br>FAM-NFQ-MGB<br>CT: 32.38	1<br>A01 SCM saki<br>U Aplano<br>152.99<br>FAM-NFQ-MGB<br>CT: 31.89	S Aplano<br>2E5<br>FAM-NFQ-MGB<br>CT: 20.92	S Aplano<br>2E5<br>FAM-NFQ-MGB<br>CT: 20.62	A07 200m saki<br>U Aplano<br>332.16<br>FAM-NFQ-MGB<br>CT: 30.69
C	A01 BTM saki<br>U Aplano<br>416.4<br>FAM-NFQ-MGB<br>CT: 30.34	A01 BTM saki<br>U Aplano<br>452.99<br>FAM-NFQ-MGB<br>CT: 30.2	A01 BTM saki<br>U Aplano<br>349.8<br>FAM-NFQ-MGB<br>CT: 30.61	S Aplano<br>2E4<br>FAM-NFQ-MGB<br>CT: 24.08	S Aplano<br>2E4<br>FAM-NFQ-MGB<br>CT: 24.17	1<br>A07 1000m saki<br>U Aplano<br>15.51<br>FAM-NFQ-MGB<br>CT: 35.44
D	A04 10m saki<br>U Aplano<br>6.95E3<br>FAM-NFQ-MGB<br>CT: 25.97	A04 10m saki<br>U Aplano<br>5.42E3<br>FAM-NFQ-MGB<br>CT: 26.35	A04 10m saki<br>U Aplano<br>5.79E3<br>FAM-NFQ-MGB<br>CT: 26.25	S Aplano<br>2E3<br>FAM-NFQ-MGB<br>CT: 27.98	S Aplano<br>2E3<br>FAM-NFQ-MGB<br>CT: 27.71	1<br>A07 3000m saki<br>U Aplano<br>20.8<br>FAM-NFQ-MGB<br>CT: 34.99
E	A04 200m saki<br>U Aplano<br>140.65<br>FAM-NFQ-MGB<br>CT: 32.02	A04 200m saki<br>U Aplano<br>259.17<br>FAM-NFQ-MGB<br>CT: 31.07				A11 10m saki<br>U Aplano<br>817.19<br>FAM-NFQ-MGB<br>CT: 29.29
F	A04 1000m saki<br>U Aplano<br>6.35<br>FAM-NFQ-MGB<br>CT: 36.83	A04 1000m saki<br>U Aplano<br>5.7<br>FAM-NFQ-MGB<br>CT: 37	A04 200m saki<br>U Aplano<br>179.98<br>FAM-NFQ-MGB<br>CT: 31.64	S Aplano<br>200<br>FAM-NFQ-MGB<br>CT: 31.05	S Aplano<br>200<br>FAM-NFQ-MGB<br>CT: 31.56	A11 SCM saki<br>U Aplano<br>491.73<br>FAM-NFQ-MGB<br>CT: 30.08
G			2<br>A04 1000m saki<br>U Aplano<br>FAM-NFQ-MGB<br>CT: Undetermined	S Aplano<br>20<br>FAM-NFQ-MGB<br>CT: 35.18	S Aplano<br>20<br>FAM-NFQ-MGB<br>CT: 35.41	1<br>A11 200m saki<br>U Aplano<br>193.05<br>FAM-NFQ-MGB<br>CT: 31.53
H	1<br>A04 3000m saki<br>U Aplano<br>11.4<br>FAM-NFQ-MGB<br>CT: 35.92	1<br>A04 3000m saki<br>U Aplano<br>FAM-NFQ-MGB<br>CT: Undetermined	1<br>A04 3000m saki<br>U Aplano<br>23.46<br>FAM-NFQ-MGB<br>CT: 34.8	N Aplano<br>FAM-NFQ-MGB<br>CT: Undetermined	N Aplano<br>FAM-NFQ-MGB<br>CT: Undetermined	1<br>A11 1000m saki<br>U Aplano<br>FAM-NFQ-MGB<br>CT: Undetermined

	7	8	9	10	11	12
A	A07 SCM saki<br>U Aplano<br>1.6E4<br>FAM-NFQ-MGB<br>CT: 24.67	A07 SCM saki<br>U Aplano<br>1.34E4<br>FAM-NFQ-MGB<br>CT: 24.94	1<br>A11 3000m saki<br>U Aplano<br>6.47<br>FAM-NFQ-MGB<br>CT: 36.8	1<br>A11 3000m saki<br>U Aplano<br>16.52<br>FAM-NFQ-MGB<br>CT: 35.34	1<br>A11 3000m saki<br>U Aplano<br>9.77<br>FAM-NFQ-MGB<br>CT: 36.16	
B	A07 200m saki<br>U Aplano<br>266.41<br>FAM-NFQ-MGB<br>CT: 31.03	A07 200m saki<br>U Aplano<br>255.66<br>FAM-NFQ-MGB<br>CT: 31.09	A21 10m saki<br>U Aplano<br>570.84<br>FAM-NFQ-MGB<br>CT: 29.85	A21 10m saki<br>U Aplano<br>634.67<br>FAM-NFQ-MGB<br>CT: 29.68	A21 10m saki<br>U Aplano<br>690.04<br>FAM-NFQ-MGB<br>CT: 29.55	
C	1<br>A07 1000m saki<br>U Aplano<br>42.77<br>FAM-NFQ-MGB<br>CT: 33.87	1<br>A07 1000m saki<br>U Aplano<br>27.55<br>FAM-NFQ-MGB<br>CT: 34.55	A21 SCM saki<br>U Aplano<br>437.39<br>FAM-NFQ-MGB<br>CT: 30.26	A21 SCM saki<br>U Aplano<br>475.46<br>FAM-NFQ-MGB<br>CT: 30.13	A21 SCM saki<br>U Aplano<br>462.94<br>FAM-NFQ-MGB<br>CT: 30.17	
D	1<br>A07 3000m saki<br>U Aplano<br>6.73<br>FAM-NFQ-MGB<br>CT: 36.74	1<br>A07 3000m saki<br>U Aplano<br>12.05<br>FAM-NFQ-MGB<br>CT: 35.83	1<br>A21 200m saki<br>U Aplano<br>92.44<br>FAM-NFQ-MGB<br>CT: 32.67	1<br>A21 200m saki<br>U Aplano<br>50.79<br>FAM-NFQ-MGB<br>CT: 33.6	1<br>A21 200m saki<br>U Aplano<br>47.13<br>FAM-NFQ-MGB<br>CT: 33.72	
E	A11 10m saki<br>U Aplano<br>1.08E3<br>FAM-NFQ-MGB<br>CT: 28.85	A11 10m saki<br>U Aplano<br>912.2<br>FAM-NFQ-MGB<br>CT: 29.12	1<br>A21 1000m saki<br>U Aplano<br>3.22E4<br>FAM-NFQ-MGB<br>CT: 23.58	2<br>A21 1000m saki<br>U Aplano<br>FAM-NFQ-MGB<br>CT: Undetermined	1<br>A21 1000m saki<br>U Aplano<br>6.47<br>FAM-NFQ-MGB<br>CT: 36.8	
F	A11 SCM saki<br>U Aplano<br>762.48<br>FAM-NFQ-MGB<br>CT: 29.4	A11 SCM saki<br>U Aplano<br>556.8<br>FAM-NFQ-MGB<br>CT: 29.88	A21 3000m saki<br>U Aplano<br>11.87<br>FAM-NFQ-MGB<br>CT: 35.86	A21 3000m saki<br>U Aplano<br>15.83<br>FAM-NFQ-MGB<br>CT: 35.41	1<br>A21 3000m saki<br>U Aplano<br>FAM-NFQ-MGB<br>CT: Undetermined	
G	1<br>A11 200m saki<br>U Aplano<br>97.21<br>FAM-NFQ-MGB<br>CT: 32.59	1<br>A11 200m saki<br>U Aplano<br>178.87<br>FAM-NFQ-MGB<br>CT: 31.65				
H	1<br>A11 1000m saki<br>U Aplano<br>FAM-NFQ-MGB<br>CT: Undetermined	1<br>A11 1000m saki<br>U Aplano<br>FAM-NFQ-MGB<br>CT: Undetermined				

Standard Curves

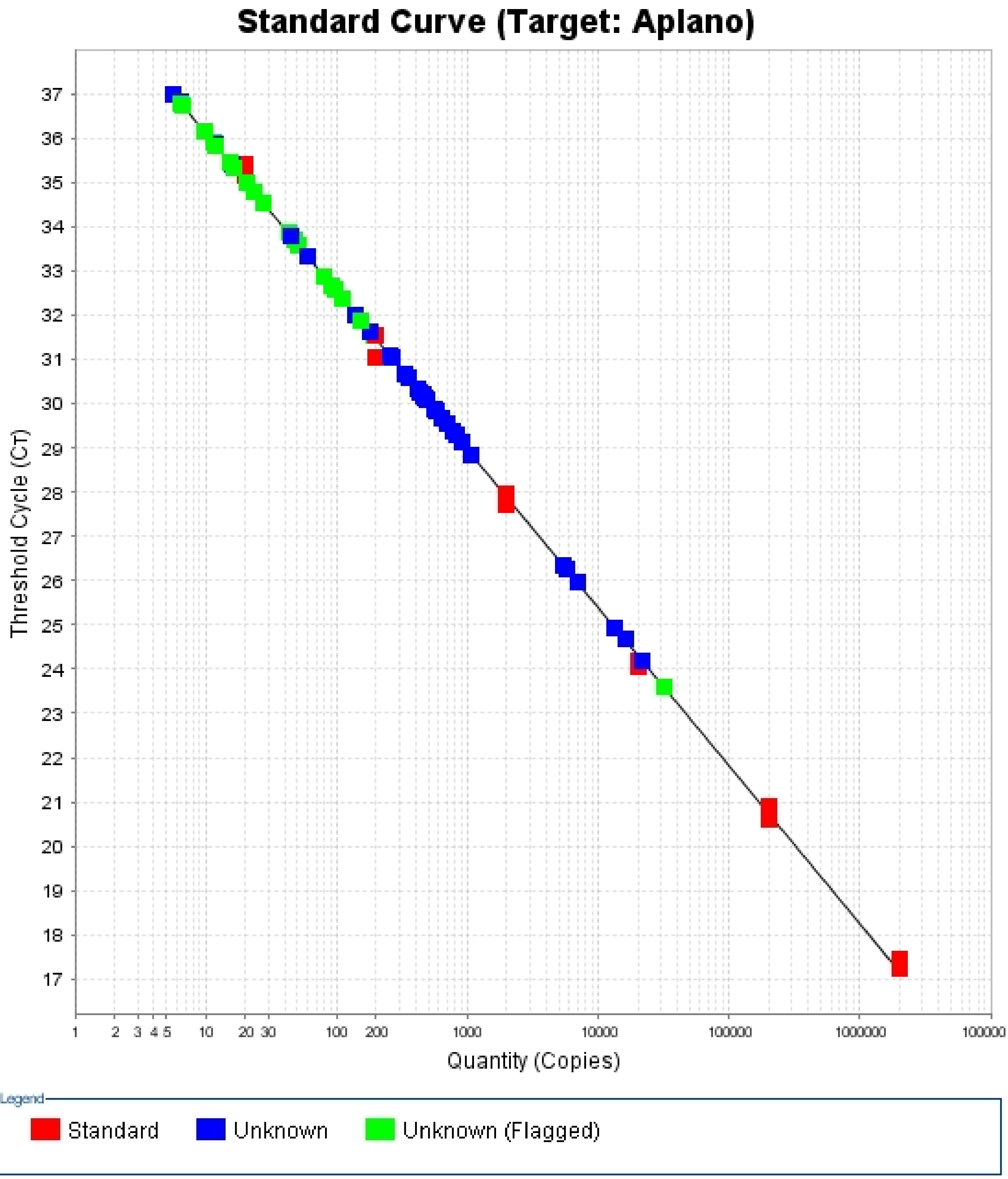

slope:-3.574 Y-Intercept:39.698 R^2:0.999 Eff%:90.447

Results Table

Well		Sample	Target	Task	Quantity	C
A1		A01 10m saki	Aplano	UNKNOWN	60.1921	33.3377
A2		A01 10m saki	Aplano	UNKNOWN	60.7753	33.3227
A3		A01 10m saki	Aplano	UNKNOWN	44.8019	33.7961
A4			Aplano	STANDARD	2000000	17.4559
A5			Aplano	STANDARD	2000000	17.2253
A6		A07 SCM saki	Aplano	UNKNOWN	21892.7988	24.1848
A7		A07 SCM saki	Aplano	UNKNOWN	16026.7539	24.6689
A8		A07 SCM saki	Aplano	UNKNOWN	13431.7041	24.9431
A9		A11 3000m saki	Aplano	UNKNOWN	6.4748	36.7987
A10		A11 3000m saki	Aplano	UNKNOWN	16.5218	35.3446
A11		A11 3000m saki	Aplano	UNKNOWN	9.7651	36.1609
B1		A01 SCM saki	Aplano	UNKNOWN	80.149	32.8932
B2		A01 SCM saki	Aplano	UNKNOWN	111.4997	32.3807
B3		A01 SCM saki	Aplano	UNKNOWN	152.9871	31.8897
B4			Aplano	STANDARD	200000	20.9179
B5			Aplano	STANDARD	200000	20.6186
B6		A07 200m saki	Aplano	UNKNOWN	332.1591	30.6863
B7		A07 200m saki	Aplano	UNKNOWN	266.4085	31.0287
B8		A07 200m saki	Aplano	UNKNOWN	255.6635	31.0926
B9		A21 10m saki	Aplano	UNKNOWN	570.8445	29.8457
B10		A21 10m saki	Aplano	UNKNOWN	634.6718	29.6811
B11		A21 10m saki	Aplano	UNKNOWN	690.0358	29.5513
C1		A01 BTM saki	Aplano	UNKNOWN	416.3991	30.3354
C2		A01 BTM saki	Aplano	UNKNOWN	452.9926	30.2046
C3		A01 BTM saki	Aplano	UNKNOWN	349.7962	30.6059
C4			Aplano	STANDARD	20000	24.0755
C5			Aplano	STANDARD	20000	24.1681
C6		A07 1000m saki	Aplano	UNKNOWN	15.5131	35.4424
C7		A07 1000m saki	Aplano	UNKNOWN	42.7733	33.868
C8		A07 1000m saki	Aplano	UNKNOWN	27.5491	34.5509

Well		Sample	Target	Task	Quantity	C
C9		A21 SCM saki	Aplano	UNKNOWN	437.3878	30.2591
C10		A21 SCM saki	Aplano	UNKNOWN	475.464	30.1295
C11		A21 SCM saki	Aplano	UNKNOWN	462.9381	30.1709
D1		A04 10m saki	Aplano	UNKNOWN	6950.2773	25.9658
D2		A04 10m saki	Aplano	UNKNOWN	5420.4019	26.3518
D3		A04 10m saki	Aplano	UNKNOWN	5794.0312	26.2483
D4			Aplano	STANDARD	2000	27.9798
D5			Aplano	STANDARD	2000	27.7066
D6		A07 3000m saki	Aplano	UNKNOWN	20.8035	34.9869
D7		A07 3000m saki	Aplano	UNKNOWN	6.7319	36.7383
D8		A07 3000m saki	Aplano	UNKNOWN	12.0544	35.834
D9		A21 200m saki	Aplano	UNKNOWN	92.4387	32.6717
D10		A21 200m saki	Aplano	UNKNOWN	50.7915	33.6013
D11		A21 200m saki	Aplano	UNKNOWN	47.1316	33.7174
E1		A04 200m saki	Aplano	UNKNOWN	140.6532	32.0202
E2		A04 200m saki	Aplano	UNKNOWN	259.1671	31.0714
E6		A11 10m saki	Aplano	UNKNOWN	817.1868	29.2888
E7		A11 10m saki	Aplano	UNKNOWN	1082.5132	28.8523
E8		A11 10m saki	Aplano	UNKNOWN	912.2003	29.118
E9		A21 1000m saki	Aplano	UNKNOWN	32246.8789	23.5836
E10		A21 1000m saki	Aplano	UNKNOWN		Undetermined
E11		A21 1000m saki	Aplano	UNKNOWN	6.4673	36.8005
F1		A04 1000m saki	Aplano	UNKNOWN	6.3465	36.8298
F2		A04 1000m saki	Aplano	UNKNOWN	5.6992	36.9968
F3		A04 200m saki	Aplano	UNKNOWN	179.9757	31.6375
F4			Aplano	STANDARD	200	31.0507
F5			Aplano	STANDARD	200	31.5583
F6		A11 SCM saki	Aplano	UNKNOWN	491.7319	30.0773
F7		A11 SCM saki	Aplano	UNKNOWN	762.4791	29.3964
F8		A11 SCM saki	Aplano	UNKNOWN	556.7984	29.8844
F9		A21 3000m saki	Aplano	UNKNOWN	11.8679	35.8582
F10		A21 3000m saki	Aplano	UNKNOWN	15.8292	35.4111

Well		Sample	Target	Task	Quantity	C
F11		A21 3000m saki	Aplano	UNKNOWN		Undetermined
G3		A04 1000m saki	Aplano	UNKNOWN		Undetermined
G4			Aplano	STANDARD	20	35.1773
G5			Aplano	STANDARD	20	35.4126
G6		A11 200m saki	Aplano	UNKNOWN	193.048	31.5286
G7		A11 200m saki	Aplano	UNKNOWN	97.2079	32.5937
G8		A11 200m saki	Aplano	UNKNOWN	178.8731	31.647
H1		A04 3000m saki	Aplano	UNKNOWN	11.3978	35.9209
H2		A04 3000m saki	Aplano	UNKNOWN		Undetermined
H3		A04 3000m saki	Aplano	UNKNOWN	23.4566	34.8006
H4			Aplano	NTC		Undetermined
H5			Aplano	NTC		Undetermined
H6		A11 1000m saki	Aplano	UNKNOWN		Undetermined
H7		A11 1000m saki	Aplano	UNKNOWN		Undetermined
H8		A11 1000m saki	Aplano	UNKNOWN		Undetermined

QC Summary

Total Wells	96	Processed Wells	77	Targets Used	1
Well Setup	77	Flagged Wells	29	Samples Used	21

Flag	Name	Frequency	Locations
AMPNC	Amplification in negative control	0	
BADROX	Bad passive reference signal	0	
BLFAIL	Baseline algorithm failed	0	
CTFAIL	C algorithm failed	0	
EXPFAIL	Exponential algorithm failed	7	E10, F11, G3, H2, H6, H7, H8
HIGHSD	High standard deviation in replicate group	22	A9, A10, A11, B1, B2, B3, C6, C7, C8, D6, D7, D8, D9, D10, D11, E9, E11, G6, G7, G8, H1, H3
NOAMP	No amplification	2	E10, G3
NOISE	Noise higher than others in plate	0	
NOSIGNAL	No signal in well	0	
OFFSCALE	Fluorescence is offscale	0	
OUTLIERRG	Outlier in replicate group	0	
SPIKE	Noise spikes	0	
THOLDFAIL	Thresholding algorithm failed	0	
