# Supplementary material for: Ecological impact of algal-preying protists aplanochytrids (Labyrinthulea) as a source of docosahexaenoic acid for copepods in marine environments
Source: ISME Commun. 2026 Jun 26;6(1):ycag178. doi: 10.1093/ismeco/ycag178 (PMC13685785; doi:10.1093/ismeco/ycag178)
Supplement: Supplementary file 2 [file supplementary_materials_ycag178.zip › Morimoto_ISME_C_3rdrev_supplemental_data.zip!/Morimoto_ISME_C_3rdrev_supplemental_data/Supplemental_Data_2/Standard Curve/Aplanochytrid/2107/Aplano2107AlineN=2.pdf]

Experiment Results Report

230728Aplano2107AlineN=2

Experiment Summary

Experiment Name	:	230728Aplano2107AlineN=2
Experiment Type	:	Quantitation - Standard Curve
File Name	:	230728Aplano2110AlineN=2.eds
Run Started	:	2023 Jul 28 12:10:08 PM
Run Finished	:	2023 Jul 28 1:48:48 PM
Run Duration	:	98 minutes 39 seconds
Date Modified	:	2023 Jul 30 2:39:53 PM
User	:	
Number of wells used	:	77
Number of wells with results	:	77
Instrument Name	:	
Instrument Type	:	Applied Biosystems StepOnePlus™ Instrument
Comments	:	

Results Summary

Sample	Target	Quantity (Mean)	Quantity (Std Dev)	C (Mean)	C (Std Dev)
A01 10m saki	Aplano	144.31	18.59	32.06	0.20
A01 BTM saki	Aplano	116.93	32.12	32.41	0.46
A01 SCM saki	Aplano	216.64	51.80	31.48	0.39
A04 1000m saki	Aplano				
A04 10m saki	Aplano	2463.10	202.36	27.82	0.13
A04 200m saki	Aplano	18.11	16.83	35.57	1.34
A04 3000m saki	Aplano				
A04 SCM saki	Aplano	4196.48	546.26	27.02	0.19
A07 1000m saki	Aplano				
A07 200m saki	Aplano	167.74	15.74	31.83	0.14
A07 3000m saki	Aplano	5.10		37.05	
A07 SCM saki	Aplano	5341.51	658.92	26.66	0.19
A11 1000m saki	Aplano				
A11 10m saki	Aplano	356.79	36.34	30.70	0.16
A11 200m saki	Aplano	57.91	25.45	33.51	0.66
A11 3000m saki	Aplano	4.11	3.20	37.64	1.31
A11 SCM saki	Aplano	447.72	90.90	30.38	0.29
A21 1000m saki	Aplano	21330764.00		14.26	
A21 200m saki	Aplano	10.10	1.76	36.04	0.25
A21 3000m saki	Aplano				
A21 SCM saki	Aplano	351.35	93.64	30.76	0.44

Plate Layout

	1	2	3	4	5	6	7	8	9	10	11	12
A	S Aplano<br>2E6<br>FAM-NFQ-MGB<br>CT: 18.23	S Aplano<br>2E6<br>FAM-NFQ-MGB<br>CT: 17.7	A01 10m saki<br>U Aplano<br>155.65<br>FAM-NFQ-MGB<br>CT: 31.94	A01 10m saki<br>U Aplano<br>154.43<br>FAM-NFQ-MGB<br>CT: 31.95	A01 10m saki<br>U Aplano<br>122.85<br>FAM-NFQ-MGB<br>CT: 32.29	A07 SCM saki<br>U Aplano<br>5.73E3<br>FAM-NFQ-MGB<br>CT: 26.55	A07 SCM saki<br>U Aplano<br>4.58E3<br>FAM-NFQ-MGB<br>CT: 26.88	A07 SCM saki<br>U Aplano<br>5.72E3<br>FAM-NFQ-MGB<br>CT: 26.55	1<br>A11 3000m saki<br>U Aplano<br>6.38<br>FAM-NFQ-MGB<br>CT: 36.71	1<br>A11 3000m saki<br>U Aplano<br>1.85<br>FAM-NFQ-MGB<br>CT: 38.56	1<br>A11 3000m saki<br>U Aplano<br>FAM-NFQ-MGB<br>CT: Undetermined	
B	S Aplano<br>2E5<br>FAM-NFQ-MGB<br>CT: 21.25	S Aplano<br>2E5<br>FAM-NFQ-MGB<br>CT: 20.96	A01 SCM saki<br>U Aplano<br>244.38<br>FAM-NFQ-MGB<br>CT: 31.26	A01 SCM saki<br>U Aplano<br>156.87<br>FAM-NFQ-MGB<br>CT: 31.93	A01 SCM saki<br>U Aplano<br>248.67<br>FAM-NFQ-MGB<br>CT: 31.24	A07 200m saki<br>U Aplano<br>176.79<br>FAM-NFQ-MGB<br>CT: 31.75	A07 200m saki<br>U Aplano<br>149.57<br>FAM-NFQ-MGB<br>CT: 32	A07 200m saki<br>U Aplano<br>176.87<br>FAM-NFQ-MGB<br>CT: 31.75	A21 SCM saki<br>U Aplano<br>412.07<br>FAM-NFQ-MGB<br>CT: 30.48	A21 SCM saki<br>U Aplano<br>243.5<br>FAM-NFQ-MGB<br>CT: 31.27	A21 SCM saki<br>U Aplano<br>398.47<br>FAM-NFQ-MGB<br>CT: 30.53	
C	S Aplano<br>2E4<br>FAM-NFQ-MGB<br>CT: 24.79	S Aplano<br>2E4<br>FAM-NFQ-MGB<br>CT: 24.24	A01 BTM saki<br>U Aplano<br>135.21<br>FAM-NFQ-MGB<br>CT: 32.15	1<br>A01 BTM saki<br>U Aplano<br>79.84<br>FAM-NFQ-MGB<br>CT: 32.94	A01 BTM saki<br>U Aplano<br>135.73<br>FAM-NFQ-MGB<br>CT: 32.14	1<br>A07 1000m saki<br>U Aplano<br>FAM-NFQ-MGB<br>CT: Undetermined	1<br>A07 1000m saki<br>U Aplano<br>FAM-NFQ-MGB<br>CT: Undetermined	1<br>A07 1000m saki<br>U Aplano<br>FAM-NFQ-MGB<br>CT: Undetermined	A21 200m saki<br>U Aplano<br>12.09<br>FAM-NFQ-MGB<br>CT: 35.76	A21 200m saki<br>U Aplano<br>8.75<br>FAM-NFQ-MGB<br>CT: 36.24	A21 200m saki<br>U Aplano<br>9.48<br>FAM-NFQ-MGB<br>CT: 36.12	
D	S Aplano<br>2E3<br>FAM-NFQ-MGB<br>CT: 28.25	S Aplano<br>2E3<br>FAM-NFQ-MGB<br>CT: 28.03	A04 10m saki<br>U Aplano<br>2.61E3<br>FAM-NFQ-MGB<br>CT: 27.73	A04 10m saki<br>U Aplano<br>2.55E3<br>FAM-NFQ-MGB<br>CT: 27.76	A04 10m saki<br>U Aplano<br>2.23E3<br>FAM-NFQ-MGB<br>CT: 27.96	1<br>A07 3000m saki<br>U Aplano<br>FAM-NFQ-MGB<br>CT: Undetermined	A07 3000m saki<br>U Aplano<br>5.1<br>FAM-NFQ-MGB<br>CT: 37.05	1<br>A07 3000m saki<br>U Aplano<br>FAM-NFQ-MGB<br>CT: Undetermined	1<br>A21 1000m saki<br>U Aplano<br>FAM-NFQ-MGB<br>CT: Undetermined	1<br>A21 1000m saki<br>U Aplano<br>FAM-NFQ-MGB<br>CT: Undetermined	1<br>A21 3000m saki<br>U Aplano<br>FAM-NFQ-MGB<br>CT: Undetermined	
E	S Aplano<br>200<br>FAM-NFQ-MGB<br>CT: 31.79	S Aplano<br>200<br>FAM-NFQ-MGB<br>CT: 31.7	A04 SCM saki<br>U Aplano<br>3.78E3<br>FAM-NFQ-MGB<br>CT: 27.17	A04 SCM saki<br>U Aplano<br>4.82E3<br>FAM-NFQ-MGB<br>CT: 26.81	A04 SCM saki<br>U Aplano<br>3.99E3<br>FAM-NFQ-MGB<br>CT: 27.09	A11 10m saki<br>U Aplano<br>373.49<br>FAM-NFQ-MGB<br>CT: 30.63	A11 10m saki<br>U Aplano<br>315.09<br>FAM-NFQ-MGB<br>CT: 30.88	A11 10m saki<br>U Aplano<br>381.78<br>FAM-NFQ-MGB<br>CT: 30.6	A21 1000m saki<br>U Aplano<br>2.13E7<br>FAM-NFQ-MGB<br>CT: 14.26	2<br>A21 3000m saki<br>U Aplano<br>FAM-NFQ-MGB<br>CT: Undetermined	1<br>A21 3000m saki<br>U Aplano<br>FAM-NFQ-MGB<br>CT: Undetermined	
F	S Aplano<br>20<br>FAM-NFQ-MGB<br>CT: 34.85	S Aplano<br>20<br>FAM-NFQ-MGB<br>CT: 35.03	1<br>A04 200m saki<br>U Aplano<br>6.78<br>FAM-NFQ-MGB<br>CT: 36.62	1<br>A04 200m saki<br>U Aplano<br>37.45<br>FAM-NFQ-MGB<br>CT: 34.07	1<br>A04 200m saki<br>U Aplano<br>10.08<br>FAM-NFQ-MGB<br>CT: 36.03	A11 SCM saki<br>U Aplano<br>373.64<br>FAM-NFQ-MGB<br>CT: 30.63	A11 SCM saki<br>U Aplano<br>420.37<br>FAM-NFQ-MGB<br>CT: 30.45	A11 SCM saki<br>U Aplano<br>549.15<br>FAM-NFQ-MGB<br>CT: 30.05				
G			1<br>A04 1000m saki<br>U Aplano<br>FAM-NFQ-MGB<br>CT: Undetermined	1<br>A04 1000m saki<br>U Aplano<br>FAM-NFQ-MGB<br>CT: Undetermined	2<br>A04 1000m saki<br>U Aplano<br>FAM-NFQ-MGB<br>CT: Undetermined	1<br>A11 200m saki<br>U Aplano<br>85.54<br>FAM-NFQ-MGB<br>CT: 32.83	1<br>A11 200m saki<br>U Aplano<br>35.44<br>FAM-NFQ-MGB<br>CT: 34.15	1<br>A11 200m saki<br>U Aplano<br>52.75<br>FAM-NFQ-MGB<br>CT: 33.56				
H	N Aplano<br>FAM-NFQ-MGB<br>CT: Undetermined	N Aplano<br>FAM-NFQ-MGB<br>CT: Undetermined	1<br>A04 3000m saki<br>U Aplano<br>FAM-NFQ-MGB<br>CT: Undetermined	1<br>A04 3000m saki<br>U Aplano<br>FAM-NFQ-MGB<br>CT: Undetermined	1<br>A04 3000m saki<br>U Aplano<br>FAM-NFQ-MGB<br>CT: Undetermined	1<br>A11 1000m saki<br>U Aplano<br>FAM-NFQ-MGB<br>CT: Undetermined	1<br>A11 1000m saki<br>U Aplano<br>FAM-NFQ-MGB<br>CT: Undetermined	1<br>A11 1000m saki<br>U Aplano<br>FAM-NFQ-MGB<br>CT: Undetermined				

Standard Curves

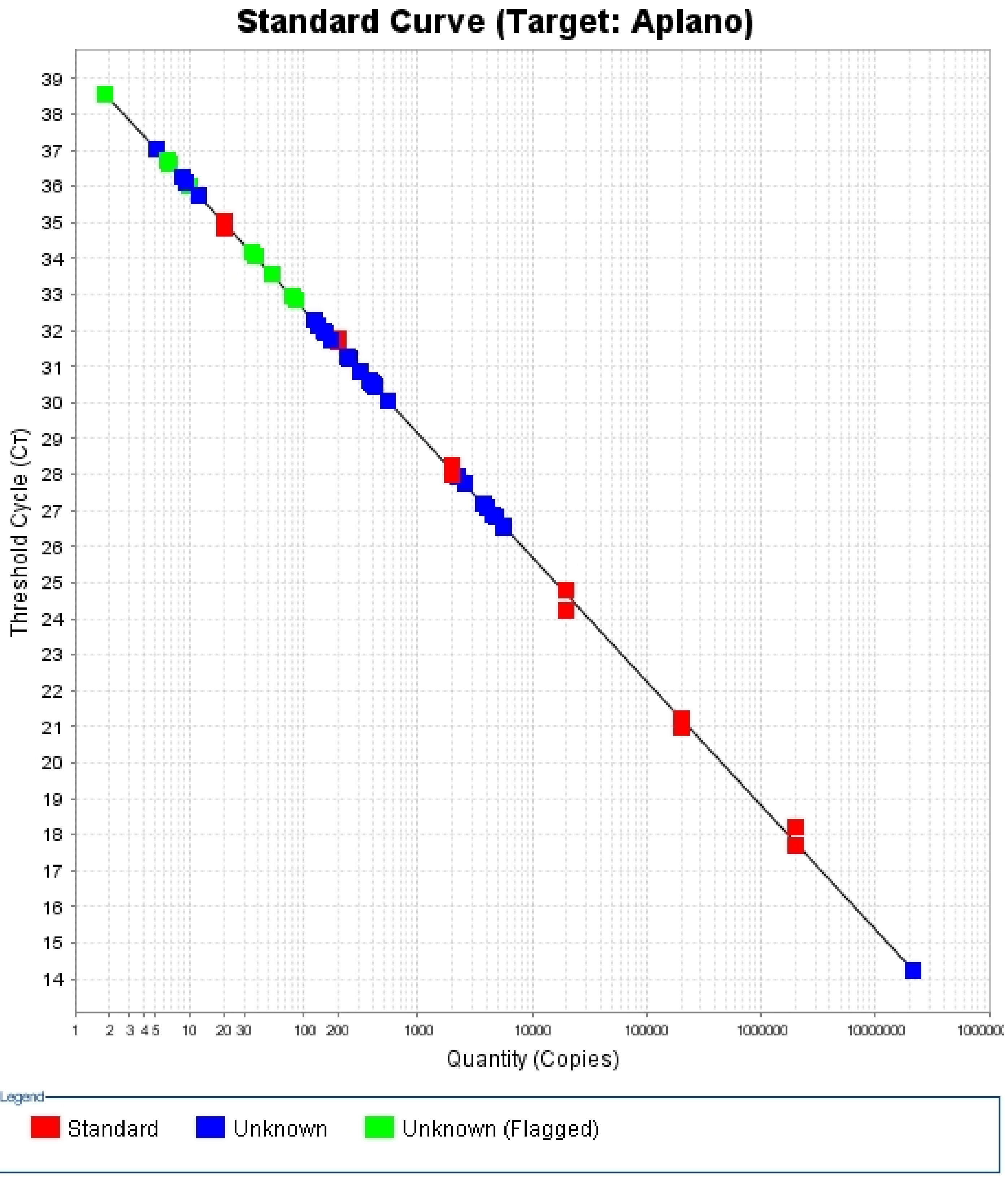

slope:-3.441 | Y-Intercept:39.482 | R^2:0.998 | Eff%:95.264

Results Table

Well		Sample	Target	Task	Quantity	C
A1			Aplano	STANDARD	2000000	18.2278
A2			Aplano	STANDARD	2000000	17.7026
A3		A01 10m saki	Aplano	UNKNOWN	155.6482	31.9388
A4		A01 10m saki	Aplano	UNKNOWN	154.4269	31.9505
A5		A01 10m saki	Aplano	UNKNOWN	122.8531	32.2923
A6		A07 SCM saki	Aplano	UNKNOWN	5726.6641	26.5511
A7		A07 SCM saki	Aplano	UNKNOWN	4580.6753	26.8848
A8		A07 SCM saki	Aplano	UNKNOWN	5717.1992	26.5536
A9		A11 3000m saki	Aplano	UNKNOWN	6.3785	36.7127
A10		A11 3000m saki	Aplano	UNKNOWN	1.8515	38.5612
A11		A11 3000m saki	Aplano	UNKNOWN		Undetermined
B1			Aplano	STANDARD	200000	21.2463
B2			Aplano	STANDARD	200000	20.9613
B3		A01 SCM saki	Aplano	UNKNOWN	244.3835	31.2646
B4		A01 SCM saki	Aplano	UNKNOWN	156.874	31.927
B5		A01 SCM saki	Aplano	UNKNOWN	248.668	31.2386
B6		A07 200m saki	Aplano	UNKNOWN	176.7914	31.7484
B7		A07 200m saki	Aplano	UNKNOWN	149.5683	31.9983
B8		A07 200m saki	Aplano	UNKNOWN	176.8686	31.7478
B9		A21 SCM saki	Aplano	UNKNOWN	412.0694	30.4838
B10		A21 SCM saki	Aplano	UNKNOWN	243.5026	31.27
B11		A21 SCM saki	Aplano	UNKNOWN	398.4706	30.534
C1			Aplano	STANDARD	20000	24.7896
C2			Aplano	STANDARD	20000	24.2445
C3		A01 BTM saki	Aplano	UNKNOWN	135.2148	32.1491
C4		A01 BTM saki	Aplano	UNKNOWN	79.8376	32.9364
C5		A01 BTM saki	Aplano	UNKNOWN	135.7304	32.1434
C6		A07 1000m saki	Aplano	UNKNOWN		Undetermined
C7		A07 1000m saki	Aplano	UNKNOWN		Undetermined
C8		A07 1000m saki	Aplano	UNKNOWN		Undetermined

Well	Sample	Target	Task	Quantity	C
C9	A21 200m saki	Aplano	UNKNOWN	12.0872	35.7575
C10	A21 200m saki	Aplano	UNKNOWN	8.7475	36.2408
C11	A21 200m saki	Aplano	UNKNOWN	9.4768	36.1211
D1		Aplano	STANDARD	2000	28.2535
D2		Aplano	STANDARD	2000	28.0289
D3	A04 10m saki	Aplano	UNKNOWN	2606.5974	27.7273
D4	A04 10m saki	Aplano	UNKNOWN	2551.0681	27.7595
D5	A04 10m saki	Aplano	UNKNOWN	2231.6428	27.9594
D6	A07 3000m saki	Aplano	UNKNOWN		Undetermined
D7	A07 3000m saki	Aplano	UNKNOWN	5.1035	37.046
D8	A07 3000m saki	Aplano	UNKNOWN		Undetermined
D9	A21 1000m saki	Aplano	UNKNOWN		Undetermined
D10	A21 1000m saki	Aplano	UNKNOWN		Undetermined
D11	A21 3000m saki	Aplano	UNKNOWN		Undetermined
E1		Aplano	STANDARD	200	31.7904
E2		Aplano	STANDARD	200	31.6973
E3	A04 SCM saki	Aplano	UNKNOWN	3782.1599	27.1711
E4	A04 SCM saki	Aplano	UNKNOWN	4815.5327	26.8101
E5	A04 SCM saki	Aplano	UNKNOWN	3991.7375	27.0905
E6	A11 10m saki	Aplano	UNKNOWN	373.4896	30.6307
E7	A11 10m saki	Aplano	UNKNOWN	315.0945	30.8848
E8	A11 10m saki	Aplano	UNKNOWN	381.7772	30.5979
E9	A21 1000m saki	Aplano	UNKNOWN	21330764	14.2633
E10	A21 3000m saki	Aplano	UNKNOWN		Undetermined
E11	A21 3000m saki	Aplano	UNKNOWN		Undetermined
F1		Aplano	STANDARD	20	34.8518
F2		Aplano	STANDARD	20	35.0349
F3	A04 200m saki	Aplano	UNKNOWN	6.7837	36.6207
F4	A04 200m saki	Aplano	UNKNOWN	37.4507	34.0676
F5	A04 200m saki	Aplano	UNKNOWN	10.0844	36.0282
F6	A11 SCM saki	Aplano	UNKNOWN	373.6407	30.6301
F7	A11 SCM saki	Aplano	UNKNOWN	420.3741	30.454

Well		Sample	Target	Task	Quantity	C
F8		A11 SCM saki	Aplano	UNKNOWN	549.1543	30.0547
G3		A04 1000m saki	Aplano	UNKNOWN		Undetermined
G4		A04 1000m saki	Aplano	UNKNOWN		Undetermined
G5		A04 1000m saki	Aplano	UNKNOWN		Undetermined
G6		A11 200m saki	Aplano	UNKNOWN	85.5396	32.8333
G7		A11 200m saki	Aplano	UNKNOWN	35.4352	34.1502
G8		A11 200m saki	Aplano	UNKNOWN	52.7543	33.5556
H1			Aplano	NTC		Undetermined
H2			Aplano	NTC		Undetermined
H3		A04 3000m saki	Aplano	UNKNOWN		Undetermined
H4		A04 3000m saki	Aplano	UNKNOWN		Undetermined
H5		A04 3000m saki	Aplano	UNKNOWN		Undetermined
H6		A11 1000m saki	Aplano	UNKNOWN		Undetermined
H7		A11 1000m saki	Aplano	UNKNOWN		Undetermined
H8		A11 1000m saki	Aplano	UNKNOWN		Undetermined

QC Summary

Total Wells	96	Processed Wells	77	Targets Used	1
Well Setup	77	Flagged Wells	29	Samples Used	21

Flag	Name	Frequency	Locations
AMPNC	Amplification in negative control	0	
BADROX	Bad passive reference signal	0	
BLFAIL	Baseline algorithm failed	0	
CTFAIL	C algorithm failed	0	
EXPFAIL	Exponential algorithm failed	20	A11, C6, C7, C8, D6, D8, D9, D10, D11, E10, E11, G3, G4, G5, H3, H4, H5, H6, H7, H8
HIGHSD	High standard deviation in replicate group	8	A9, A10, F3, F4, F5, G6, G7, G8
NOAMP	No amplification	2	E10, G5
NOISE	Noise higher than others in plate	0	
NOSIGNAL	No signal in well	0	
OFFSCALE	Fluorescence is offscale	0	
OUTLIERRG	Outlier in replicate group	1	C4
SPIKE	Noise spikes	0	
THOLDFAIL	Thresholding algorithm failed	0	
