# Supplementary material for: Ecological impact of algal-preying protists aplanochytrids (Labyrinthulea) as a source of docosahexaenoic acid for copepods in marine environments
Source: ISME Commun. 2026 Jun 26;6(1):ycag178. doi: 10.1093/ismeco/ycag178 (PMC13685785; doi:10.1093/ismeco/ycag178)
Supplement: Supplementary file 2 [file supplementary_materials_ycag178.zip › Morimoto_ISME_C_3rdrev_supplemental_data.zip!/Morimoto_ISME_C_3rdrev_supplemental_data/Supplemental_Data_2/Standard Curve/Aplanochytrid/2201/Aplano2201AlineN=2.pdf]

Experiment Results Report

230720Aplano2201AlineN=2

Experiment Summary

Experiment Name	:	230720Aplano2201AlineN=2
Experiment Type	:	Quantitation - Standard Curve
File Name	:	230720Aplano2201AlineN=2.eds
Run Started	:	2023 Jul 20 5:49:53 PM
Run Finished	:	2023 Jul 20 7:24:32 PM
Run Duration	:	94 minutes 38 seconds
Date Modified	:	2023 Jul 21 11:01:31 AM
User	:	
Number of wells used	:	71
Number of wells with results	:	71
Instrument Name	:	
Instrument Type	:	Applied Biosystems StepOnePlus™ Instrument
Comments	:	

Results Summary

Sample	Target	Quantity (Mean)	Quantity (Std Dev)	C (Mean)	C (Std Dev)
2201 A01 10m saki1	Aplano	67.51	24.23	32.78	0.56
2201 A01 SCM saki1	Aplano	50.09	22.68	33.32	0.87
2201 A04 1000m	Aplano	15.61	4.85	35.03	0.49
2201 A04 10m saki1	Aplano	85.51	21.32	32.38	0.39
2201 A04 200m	Aplano	32.68	14.78	33.94	0.67
2201 A04 3000m	Aplano				
2201 A04 SCM saki1	Aplano	60.69	13.74	32.91	0.34
2201 A07 1000m	Aplano				
2201 A07 200m	Aplano	109.14	45.87	32.08	0.78
2201 A07 3000m	Aplano				
2201 A07 SCM saki1	Aplano	97.92	56.15	32.39	1.19
2201 A11 1000m	Aplano	25097.25		23.51	
2201 A11 200m	Aplano	43.07	41.19	34.31	2.42
2201 A11 3000m	Aplano				
2201 A11 SCM saki1	Aplano	852.94	104.83	28.78	0.19
2201 A21 1000m	Aplano	133178.30		20.92	
2201 A21 200m	Aplano	43.54	7.68	33.41	0.28
2201 A21 3000m	Aplano				
2201 A21 SCM saki1	Aplano	446.03	37.55	29.78	0.13

Plate Layout

	1	2	3	4	5	6	7	8	9	10	11	12
A	S Aplano<br>2E6<br>FAM-NFQ-MGB<br>CT: 17.62	S Aplano<br>2E6<br>FAM-NFQ-MGB<br>CT: 16.96	⚠1<br>2201 A01 10m saki1<br>U Aplano<br>45.93<br>FAM-NFQ-MGB<br>CT: 33.31	⚠1<br>2201 A01 10m saki1<br>U Aplano<br>93.72<br>FAM-NFQ-MGB<br>CT: 32.21	⚠1<br>2201 A01 10m saki1<br>U Aplano<br>62.88<br>FAM-NFQ-MGB<br>CT: 32.83	⚠1<br>2201 A07 SCM saki1<br>U Aplano<br>34.66<br>FAM-NFQ-MGB<br>CT: 33.75	⚠1<br>2201 A07 SCM saki1<br>U Aplano<br>117.22<br>FAM-NFQ-MGB<br>CT: 31.86	⚠1<br>2201 A07 SCM saki1<br>U Aplano<br>141.87<br>FAM-NFQ-MGB<br>CT: 31.56	2201 A11 SCM saki1<br>U Aplano<br>828.72<br>FAM-NFQ-MGB<br>CT: 28.82	2201 A11 SCM saki1<br>U Aplano<br>762.34<br>FAM-NFQ-MGB<br>CT: 28.95	2201 A11 SCM saki1<br>U Aplano<br>967.75<br>FAM-NFQ-MGB<br>CT: 28.58	
B	S Aplano<br>2E5<br>FAM-NFQ-MGB<br>CT: 19.83	S Aplano<br>2E5<br>FAM-NFQ-MGB<br>CT: 20.21	⚠1<br>2201 A01 SCM saki1<br>U Aplano<br>64.07<br>FAM-NFQ-MGB<br>CT: 32.8	⚠1<br>2201 A01 SCM saki1<br>U Aplano<br>62.28<br>FAM-NFQ-MGB<br>CT: 32.84	⚠2<br>2201 A01 SCM saki1<br>U Aplano<br>23.92<br>FAM-NFQ-MGB<br>CT: 34.33	⚠1<br>2201 A07 200m saki1<br>U Aplano<br>57.24<br>FAM-NFQ-MGB<br>CT: 32.97	⚠1<br>2201 A07 200m saki1<br>U Aplano<br>144.25<br>FAM-NFQ-MGB<br>CT: 31.54	⚠1<br>2201 A07 200m saki1<br>U Aplano<br>125.94<br>FAM-NFQ-MGB<br>CT: 31.75	⚠1<br>2201 A11 200m saki1<br>U Aplano<br>38.66<br>FAM-NFQ-MGB<br>CT: 33.58	⚠1<br>2201 A11 200m saki1<br>U Aplano<br>4.26<br>FAM-NFQ-MGB<br>CT: 37.01	⚠1<br>2201 A11 200m saki1<br>U Aplano<br>86.28<br>FAM-NFQ-MGB<br>CT: 32.33	
C	S Aplano<br>2E4<br>FAM-NFQ-MGB<br>CT: 23.76	S Aplano<br>2E4<br>FAM-NFQ-MGB<br>CT: 23.2	2201 A04 10m saki1<br>U Aplano<br>107.17<br>FAM-NFQ-MGB<br>CT: 32	2201 A04 10m saki1<br>U Aplano<br>64.54<br>FAM-NFQ-MGB<br>CT: 32.79	2201 A04 10m saki1<br>U Aplano<br>84.8<br>FAM-NFQ-MGB<br>CT: 32.36	⚠1<br>2201 A07 1000m saki1<br>U Aplano<br>FAM-NFQ-MGB<br>CT: Undetermined	⚠2<br>2201 A07 1000m saki1<br>U Aplano<br>FAM-NFQ-MGB<br>CT: Undetermined	⚠2<br>2201 A07 1000m saki1<br>U Aplano<br>FAM-NFQ-MGB<br>CT: Undetermined	⚠2<br>2201 A11 1000m saki1<br>U Aplano<br>FAM-NFQ-MGB<br>CT: Undetermined	⚠1<br>2201 A11 1000m saki1<br>U Aplano<br>FAM-NFQ-MGB<br>CT: Undetermined	⚠1<br>2201 A11 1000m saki1<br>U Aplano<br>2.51E4<br>FAM-NFQ-MGB<br>CT: 23.51	
D	S Aplano<br>2E3<br>FAM-NFQ-MGB<br>CT: 27.05	S Aplano<br>2E3<br>FAM-NFQ-MGB<br>CT: 27.26	2201 A04 SCM saki1<br>U Aplano<br>49.31<br>FAM-NFQ-MGB<br>CT: 33.2	2201 A04 SCM saki1<br>U Aplano<br>75.96<br>FAM-NFQ-MGB<br>CT: 32.53	2201 A04 SCM saki1<br>U Aplano<br>56.81<br>FAM-NFQ-MGB<br>CT: 32.98	⚠1<br>2201 A07 3000m saki1<br>U Aplano<br>FAM-NFQ-MGB<br>CT: Undetermined	⚠1<br>2201 A07 3000m saki1<br>U Aplano<br>FAM-NFQ-MGB<br>CT: Undetermined	⚠2<br>2201 A07 3000m saki1<br>U Aplano<br>FAM-NFQ-MGB<br>CT: Undetermined	⚠1<br>2201 A11 3000m saki1<br>U Aplano<br>FAM-NFQ-MGB<br>CT: Undetermined	⚠2<br>2201 A11 3000m saki1<br>U Aplano<br>FAM-NFQ-MGB<br>CT: Undetermined	⚠1<br>2201 A11 3000m saki1<br>U Aplano<br>FAM-NFQ-MGB<br>CT: Undetermined	
E	S Aplano<br>200<br>FAM-NFQ-MGB<br>CT: 30.87	S Aplano<br>200<br>FAM-NFQ-MGB<br>CT: 30.92	⚠1<br>2201 A04 200m saki1<br>U Aplano<br>21.41<br>FAM-NFQ-MGB<br>CT: 34.5	⚠1<br>2201 A04 200m saki1<br>U Aplano<br>27.22<br>FAM-NFQ-MGB<br>CT: 34.13	⚠1<br>2201 A04 200m saki1<br>U Aplano<br>49.41<br>FAM-NFQ-MGB<br>CT: 33.2				2201 A21 SCM saki1<br>U Aplano<br>455.53<br>FAM-NFQ-MGB<br>CT: 29.75	2201 A21 SCM saki1<br>U Aplano<br>404.65<br>FAM-NFQ-MGB<br>CT: 29.93	2201 A21 SCM saki1<br>U Aplano<br>477.91<br>FAM-NFQ-MGB<br>CT: 29.67	
F	S Aplano<br>20<br>FAM-NFQ-MGB<br>CT: 34.93	S Aplano<br>20<br>FAM-NFQ-MGB<br>CT: 35.26	2201 A04 1000m saki1<br>U Aplano<br>19.04<br>FAM-NFQ-MGB<br>CT: 34.68	2201 A04 1000m saki1<br>U Aplano<br>12.18<br>FAM-NFQ-MGB<br>CT: 35.38	⚠1<br>2201 A04 1000m saki1<br>U Aplano<br>FAM-NFQ-MGB<br>CT: Undetermined				2201 A21 200m saki1<br>U Aplano<br>50.62<br>FAM-NFQ-MGB<br>CT: 33.16	2201 A21 200m saki1<br>U Aplano<br>35.38<br>FAM-NFQ-MGB<br>CT: 33.72	2201 A21 200m saki1<br>U Aplano<br>44.62<br>FAM-NFQ-MGB<br>CT: 33.36	
G			⚠2<br>2201 A04 3000m saki1<br>U Aplano<br>FAM-NFQ-MGB<br>CT: Undetermined	⚠1<br>2201 A04 3000m saki1<br>U Aplano<br>FAM-NFQ-MGB<br>CT: Undetermined	⚠2<br>2201 A04 3000m saki1<br>U Aplano<br>FAM-NFQ-MGB<br>CT: Undetermined				⚠2<br>2201 A21 1000m saki1<br>U Aplano<br>FAM-NFQ-MGB<br>CT: Undetermined	2201 A21 1000m saki1<br>U Aplano<br>1.33E5<br>FAM-NFQ-MGB<br>CT: 20.92	2201 A21 1000m saki1<br>U Aplano<br>FAM-NFQ-MGB<br>CT: Undetermined	
H	N Aplano<br>FAM-NFQ-MGB<br>CT: Undetermined	N Aplano<br>FAM-NFQ-MGB<br>CT: Undetermined							⚠1<br>2201 A21 3000m saki1<br>U Aplano<br>FAM-NFQ-MGB<br>CT: Undetermined	⚠1<br>2201 A21 3000m saki1<br>U Aplano<br>FAM-NFQ-MGB<br>CT: Undetermined	2201 A21 3000m saki1<br>U Aplano<br>FAM-NFQ-MGB<br>CT: Undetermined	

Standard Curves

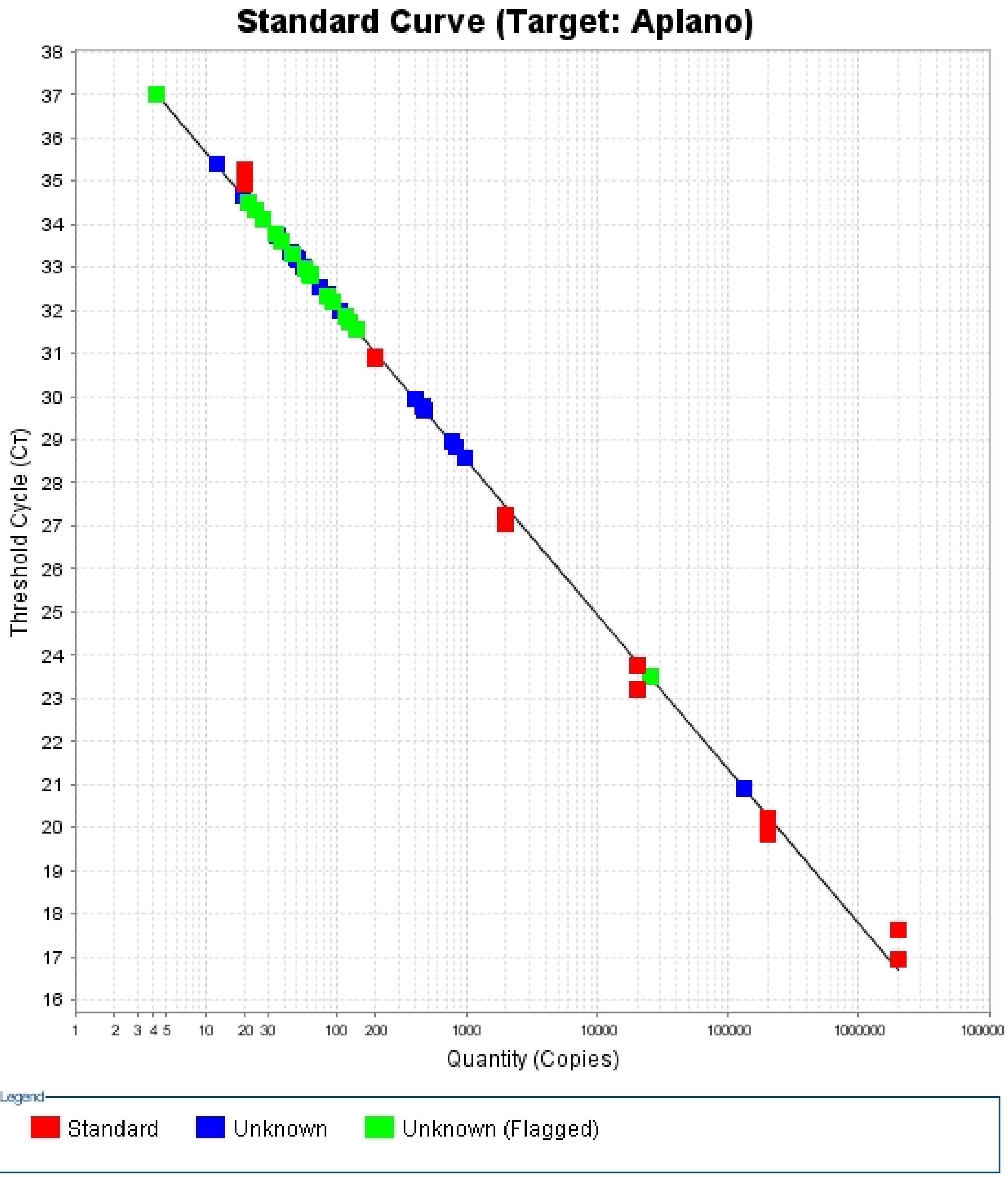

slope:-3.58 Y-Intercept:39.265 R^2:0.995 Eff%:90.245

Results Table

Well		Sample	Target	Task	Quantity	C
A1			Aplano	STANDARD	2000000	17.622
A2			Aplano	STANDARD	2000000	16.9605
A3		2201 A01 10m	Aplano	UNKNOWN	45.9261	33.3149
A4		2201 A01 10m	Aplano	UNKNOWN	93.7178	32.2059
A5		2201 A01 10m	Aplano	UNKNOWN	62.8766	32.8265
A6		2201 A07 SCM	Aplano	UNKNOWN	34.6631	33.7524
A7		2201 A07 SCM	Aplano	UNKNOWN	117.2154	31.858
A8		2201 A07 SCM	Aplano	UNKNOWN	141.8733	31.5612
A9		2201 A11 SCM	Aplano	UNKNOWN	828.7164	28.8169
A10		2201 A11 SCM	Aplano	UNKNOWN	762.3398	28.9467
A11		2201 A11 SCM	Aplano	UNKNOWN	967.7543	28.5758
B1			Aplano	STANDARD	200000	19.8292
B2			Aplano	STANDARD	200000	20.2137
B3		2201 A01 SCM	Aplano	UNKNOWN	64.0705	32.7972
B4		2201 A01 SCM	Aplano	UNKNOWN	62.2792	32.8413
B5		2201 A01 SCM	Aplano	UNKNOWN	23.9218	34.329
B6		2201 A07 200m	Aplano	UNKNOWN	57.2411	32.9725
B7		2201 A07 200m	Aplano	UNKNOWN	144.245	31.5354
B8		2201 A07 200m	Aplano	UNKNOWN	125.9351	31.7465
B9		2201 A11 200m	Aplano	UNKNOWN	38.6584	33.5827
B10		2201 A11 200m	Aplano	UNKNOWN	4.2598	37.0121
B11		2201 A11 200m	Aplano	UNKNOWN	86.2792	32.3345
C1			Aplano	STANDARD	20000	23.7583
C2			Aplano	STANDARD	20000	23.2003
C3		2201 A04 10m	Aplano	UNKNOWN	107.1716	31.9973
C4		2201 A04 10m	Aplano	UNKNOWN	64.543	32.7858
C5		2201 A04 10m	Aplano	UNKNOWN	84.8018	32.3613
C6		2201 A07	Aplano	UNKNOWN		Undetermined
C7		2201 A07	Aplano	UNKNOWN		Undetermined
C8		2201 A07	Aplano	UNKNOWN		Undetermined

Well		Sample	Target	Task	Quantity	C
C9		2201 A11	Aplano	UNKNOWN		Undetermined
C10		2201 A11	Aplano	UNKNOWN		Undetermined
C11		2201 A11	Aplano	UNKNOWN	25097.2461	23.5139
D1			Aplano	STANDARD	2000	27.0533
D2			Aplano	STANDARD	2000	27.2644
D3		2201 A04 SCM	Aplano	UNKNOWN	49.3106	33.2043
D4		2201 A04 SCM	Aplano	UNKNOWN	75.9564	32.5326
D5		2201 A04 SCM	Aplano	UNKNOWN	56.8142	32.9841
D6		2201 A07	Aplano	UNKNOWN		Undetermined
D7		2201 A07	Aplano	UNKNOWN		Undetermined
D8		2201 A07	Aplano	UNKNOWN		Undetermined
D9		2201 A11	Aplano	UNKNOWN		Undetermined
D10		2201 A11	Aplano	UNKNOWN		Undetermined
D11		2201 A11	Aplano	UNKNOWN		Undetermined
E1			Aplano	STANDARD	200	30.8744
E2			Aplano	STANDARD	200	30.9224
E3		2201 A04 200m	Aplano	UNKNOWN	21.4081	34.5017
E4		2201 A04 200m	Aplano	UNKNOWN	27.219	34.1283
E5		2201 A04 200m	Aplano	UNKNOWN	49.4125	33.2011
E9		2201 A21 SCM	Aplano	UNKNOWN	455.5345	29.7474
E10		2201 A21 SCM	Aplano	UNKNOWN	404.6451	29.9316
E11		2201 A21 SCM	Aplano	UNKNOWN	477.9086	29.6728
F1			Aplano	STANDARD	20	34.9258
F2			Aplano	STANDARD	20	35.2562
F3		2201 A04	Aplano	UNKNOWN	19.0357	34.6843
F4		2201 A04	Aplano	UNKNOWN	12.1805	35.3785
F5		2201 A04	Aplano	UNKNOWN		Undetermined
F9		2201 A21 200m	Aplano	UNKNOWN	50.6199	33.1636
F10		2201 A21 200m	Aplano	UNKNOWN	35.3786	33.7206
F11		2201 A21 200m	Aplano	UNKNOWN	44.6182	33.3598
G3		2201 A04	Aplano	UNKNOWN		Undetermined
G4		2201 A04	Aplano	UNKNOWN		Undetermined

Well		Sample	Target	Task	Quantity	C
G5		2201 A04	Aplano	UNKNOWN		Undetermined
G9		2201 A21	Aplano	UNKNOWN		Undetermined
G10		2201 A21	Aplano	UNKNOWN	133178.2969	20.9189
G11		2201 A21	Aplano	UNKNOWN		Undetermined
H1			Aplano	NTC		Undetermined
H2			Aplano	NTC		Undetermined
H9		2201 A21	Aplano	UNKNOWN		Undetermined
H10		2201 A21	Aplano	UNKNOWN		Undetermined
H11		2201 A21	Aplano	UNKNOWN		Undetermined

QC Summary

Total Wells	96	Processed Wells	71	Targets Used	1
Well Setup	71	Flagged Wells	37	Samples Used	19

Flag	Name	Frequency	Locations
AMPNC	Amplification in negative control	0	
BADROX	Bad passive reference signal	0	
BLFAIL	Baseline algorithm failed	0	
CTFAIL	C algorithm failed	0	
EXPFAIL	Exponential algorithm failed	18	C6, C7, C8, C9, C10, D6, D7, D8, D9, D10, D11, F5, G3, G4, G5, G9, H9, H10
HIGHSD	High standard deviation in replicate group	18	A3, A4, A5, A6, A7, A8, B3, B4, B5, B6, B7, B8, B9, B10, B11, E3, E4, E5
NOAMP	No amplification	9	C7, C8, C9, C11, D8, D10, G3, G5, G9
NOISE	Noise higher than others in plate	0	
NOSIGNAL	No signal in well	0	
OFFSCALE	Fluorescence is offscale	0	
OUTLIERRG	Outlier in replicate group	1	B5
SPIKE	Noise spikes	0	
THOLDFAIL	Thresholding algorithm failed	0	
