# Supplementary material for: Ecological impact of algal-preying protists aplanochytrids (Labyrinthulea) as a source of docosahexaenoic acid for copepods in marine environments
Source: ISME Commun. 2026 Jun 26;6(1):ycag178. doi: 10.1093/ismeco/ycag178 (PMC13685785; doi:10.1093/ismeco/ycag178)
Supplement: Supplementary file 2 [file supplementary_materials_ycag178.zip › Morimoto_ISME_C_3rdrev_supplemental_data.zip!/Morimoto_ISME_C_3rdrev_supplemental_data/Supplemental_Data_2/Standard Curve/Aplanochytrid/2201/Aplano2201AlineN=3.pdf]

Experiment Results Report

230721Aplano2201AlineN=3

Experiment Summary

Experiment Name	:	230721Aplano2201AlineN=3
Experiment Type	:	Quantitation - Standard Curve
File Name	:	230721Aplano2201AlineN=3.eds
Run Started	:	2023 Jul 21 12:10:05 PM
Run Finished	:	2023 Jul 21 1:48:28 PM
Run Duration	:	98 minutes 22 seconds
Date Modified	:	2023 Jul 21 2:00:20 PM
User	:	
Number of wells used	:	71
Number of wells with results	:	71
Instrument Name	:	
Instrument Type	:	Applied Biosystems StepOnePlus™ Instrument
Comments	:	

Results Summary

Sample	Target	Quantity (Mean)	Quantity (Std Dev)	C (Mean)	C (Std Dev)
2201 A01 10m saki1	Aplano	25.80	13.21	34.02	0.70
2201 A01 SCM saki1	Aplano	33.52	20.79	33.75	1.06
2201 A04 1000m	Aplano				
2201 A04 10m saki1	Aplano	30.96	23.32	33.99	1.33
2201 A04 200m	Aplano	22.45	19.90	34.61	1.61
2201 A04 3000m	Aplano				
2201 A04 SCM saki1	Aplano	26.25	10.98	33.95	0.56
2201 A07 1000m	Aplano	0.86		38.86	
2201 A07 200m	Aplano	81.92	25.37	32.27	0.48
2201 A07 3000m	Aplano				
2201 A07 SCM saki1	Aplano	92.44	20.47	32.06	0.35
2201 A11 1000m	Aplano				
2201 A11 200m	Aplano	3.69	0.51	36.74	0.21
2201 A11 3000m	Aplano	5.99	2.96	36.12	0.75
2201 A11 SCM saki1	Aplano	376.76	33.02	29.99	0.13
2201 A21 1000m	Aplano				
2201 A21 200m	Aplano	14.58	11.59	35.03	1.13
2201 A21 3000m	Aplano				
2201 A21 SCM saki1	Aplano	198.95	33.00	30.94	0.25

Plate Layout

	1	2	3	4	5	6	7	8	9	10	11	12
A	S Aplano<br>2E6<br>FAM-NFQ-MGB<br>CT: 17.76	S Aplano<br>2E6<br>FAM-NFQ-MGB<br>CT: 17.63	⚠1<br>2201 A01 10m saki1<br>U Aplano<br>40.94<br>FAM-NFQ-MGB<br>CT: 33.23	⚠1<br>2201 A01 10m saki1<br>U Aplano<br>19.82<br>FAM-NFQ-MGB<br>CT: 34.28	⚠1<br>2201 A01 10m saki1<br>U Aplano<br>16.64<br>FAM-NFQ-MGB<br>CT: 34.54	2201 A07 SCM saki1<br>U Aplano<br>108.32<br>FAM-NFQ-MGB<br>CT: 31.81	2201 A07 SCM saki1<br>U Aplano<br>69.34<br>FAM-NFQ-MGB<br>CT: 32.46	2201 A07 SCM saki1<br>U Aplano<br>99.66<br>FAM-NFQ-MGB<br>CT: 31.93	2201 A11 SCM saki1<br>U Aplano<br>397.02<br>FAM-NFQ-MGB<br>CT: 29.91	2201 A11 SCM saki1<br>U Aplano<br>338.66<br>FAM-NFQ-MGB<br>CT: 30.15	2201 A11 SCM saki1<br>U Aplano<br>394.6<br>FAM-NFQ-MGB<br>CT: 29.92	
B	S Aplano<br>2E5<br>FAM-NFQ-MGB<br>CT: 20.65	S Aplano<br>2E5<br>FAM-NFQ-MGB<br>CT: 20.82	⚠1<br>2201 A01 SCM saki1<br>U Aplano<br>54.66<br>FAM-NFQ-MGB<br>CT: 32.8	⚠1<br>2201 A01 SCM saki1<br>U Aplano<br>32.82<br>FAM-NFQ-MGB<br>CT: 33.55	⚠1<br>2201 A01 SCM saki1<br>U Aplano<br>13.09<br>FAM-NFQ-MGB<br>CT: 34.89	2201 A07 200m saki1<br>U Aplano<br>105.4<br>FAM-NFQ-MGB<br>CT: 31.85	2201 A07 200m saki1<br>U Aplano<br>55.01<br>FAM-NFQ-MGB<br>CT: 32.79	2201 A07 200m saki1<br>U Aplano<br>4.66<br>FAM-NFQ-MGB<br>CT: 36.67	2201 A11 200m saki1<br>U Aplano<br>3.89<br>FAM-NFQ-MGB<br>CT: 36.66	2201 A11 200m saki1<br>U Aplano<br>3.11<br>FAM-NFQ-MGB<br>CT: 36.98	2201 A11 200m saki1<br>U Aplano<br>4.07<br>FAM-NFQ-MGB<br>CT: 36.59	
C	S Aplano<br>2E4<br>FAM-NFQ-MGB<br>CT: 24.14	S Aplano<br>2E4<br>FAM-NFQ-MGB<br>CT: 23.88	⚠1<br>2201 A04 10m saki1<br>U Aplano<br>9.11<br>FAM-NFQ-MGB<br>CT: 35.42	⚠1<br>2201 A04 10m saki1<br>U Aplano<br>28.25<br>FAM-NFQ-MGB<br>CT: 33.77	⚠1<br>2201 A04 10m saki1<br>U Aplano<br>55.51<br>FAM-NFQ-MGB<br>CT: 32.78	⚠1<br>2201 A07 1000m saki1<br>U Aplano<br>FAM-NFQ-MGB<br>CT: Undetermined	⚠1<br>2201 A07 1000m saki1<br>U Aplano<br>FAM-NFQ-MGB<br>CT: Undetermined	2201 A07 1000m saki1<br>U Aplano<br>0.86<br>FAM-NFQ-MGB<br>CT: 38.86	⚠1<br>2201 A11 1000m saki1<br>U Aplano<br>FAM-NFQ-MGB<br>CT: Undetermined	⚠2<br>2201 A11 1000m saki1<br>U Aplano<br>FAM-NFQ-MGB<br>CT: Undetermined	⚠1<br>2201 A11 1000m saki1<br>U Aplano<br>FAM-NFQ-MGB<br>CT: Undetermined	
D	S Aplano<br>2E3<br>FAM-NFQ-MGB<br>CT: 27.61	S Aplano<br>2E3<br>FAM-NFQ-MGB<br>CT: 27.36	⚠1<br>2201 A04 SCM saki1<br>U Aplano<br>20.41<br>FAM-NFQ-MGB<br>CT: 34.24	⚠1<br>2201 A04 SCM saki1<br>U Aplano<br>38.91<br>FAM-NFQ-MGB<br>CT: 33.3	⚠1<br>2201 A04 SCM saki1<br>U Aplano<br>19.42<br>FAM-NFQ-MGB<br>CT: 34.31	⚠1<br>2201 A07 3000m saki1<br>U Aplano<br>FAM-NFQ-MGB<br>CT: Undetermined	⚠2<br>2201 A07 3000m saki1<br>U Aplano<br>FAM-NFQ-MGB<br>CT: Undetermined	⚠1<br>2201 A07 3000m saki1<br>U Aplano<br>FAM-NFQ-MGB<br>CT: Undetermined	⚠2<br>2201 A11 3000m saki1<br>U Aplano<br>FAM-NFQ-MGB<br>CT: Undetermined	⚠1<br>2201 A11 3000m saki1<br>U Aplano<br>3.9<br>FAM-NFQ-MGB<br>CT: 36.65	⚠1<br>2201 A11 3000m saki1<br>U Aplano<br>8.09<br>FAM-NFQ-MGB<br>CT: 35.59	
E	S Aplano<br>200<br>FAM-NFQ-MGB<br>CT: 31.21	S Aplano<br>200<br>FAM-NFQ-MGB<br>CT: 30.77	⚠1<br>2201 A04 200m saki1<br>U Aplano<br>18.39<br>FAM-NFQ-MGB<br>CT: 34.39	⚠1<br>2201 A04 200m saki1<br>U Aplano<br>44.07<br>FAM-NFQ-MGB<br>CT: 33.12	⚠1<br>2201 A04 200m saki1<br>U Aplano<br>4.9<br>FAM-NFQ-MGB<br>CT: 36.32				2201 A21 SCM saki1<br>U Aplano<br>163.3<br>FAM-NFQ-MGB<br>CT: 31.21	2201 A21 SCM saki1<br>U Aplano<br>228.41<br>FAM-NFQ-MGB<br>CT: 30.72	2201 A21 SCM saki1<br>U Aplano<br>205.13<br>FAM-NFQ-MGB<br>CT: 30.88	
F	S Aplano<br>20<br>FAM-NFQ-MGB<br>CT: 34.03	S Aplano<br>20<br>FAM-NFQ-MGB<br>CT: 34.66	⚠1<br>2201 A04 1000m saki1<br>U Aplano<br>FAM-NFQ-MGB<br>CT: Undetermined	⚠1<br>2201 A04 1000m saki1<br>U Aplano<br>FAM-NFQ-MGB<br>CT: Undetermined	⚠1<br>2201 A04 1000m saki1<br>U Aplano<br>FAM-NFQ-MGB<br>CT: Undetermined				⚠1<br>2201 A21 200m saki1<br>U Aplano<br>9.9<br>FAM-NFQ-MGB<br>CT: 35.29	⚠1<br>2201 A21 200m saki1<br>U Aplano<br>27.78<br>FAM-NFQ-MGB<br>CT: 33.79	⚠1<br>2201 A21 200m saki1<br>U Aplano<br>6.06<br>FAM-NFQ-MGB<br>CT: 36.01	
G			⚠1<br>2201 A04 3000m saki1<br>U Aplano<br>FAM-NFQ-MGB<br>CT: Undetermined	⚠1<br>2201 A04 3000m saki1<br>U Aplano<br>FAM-NFQ-MGB<br>CT: Undetermined	⚠2<br>2201 A04 3000m saki1<br>U Aplano<br>FAM-NFQ-MGB<br>CT: Undetermined				⚠1<br>2201 A21 1000m saki1<br>U Aplano<br>FAM-NFQ-MGB<br>CT: Undetermined	⚠1<br>2201 A21 1000m saki1<br>U Aplano<br>FAM-NFQ-MGB<br>CT: Undetermined	⚠1<br>2201 A21 1000m saki1<br>U Aplano<br>FAM-NFQ-MGB<br>CT: Undetermined	
H	N Aplano<br>FAM-NFQ-MGB<br>CT: Undetermined	N Aplano<br>FAM-NFQ-MGB<br>CT: Undetermined							⚠1<br>2201 A21 3000m saki1<br>U Aplano<br>FAM-NFQ-MGB<br>CT: Undetermined	⚠1<br>2201 A21 3000m saki1<br>U Aplano<br>FAM-NFQ-MGB<br>CT: Undetermined	⚠1<br>2201 A21 3000m saki1<br>U Aplano<br>FAM-NFQ-MGB<br>CT: Undetermined	

Standard Curves

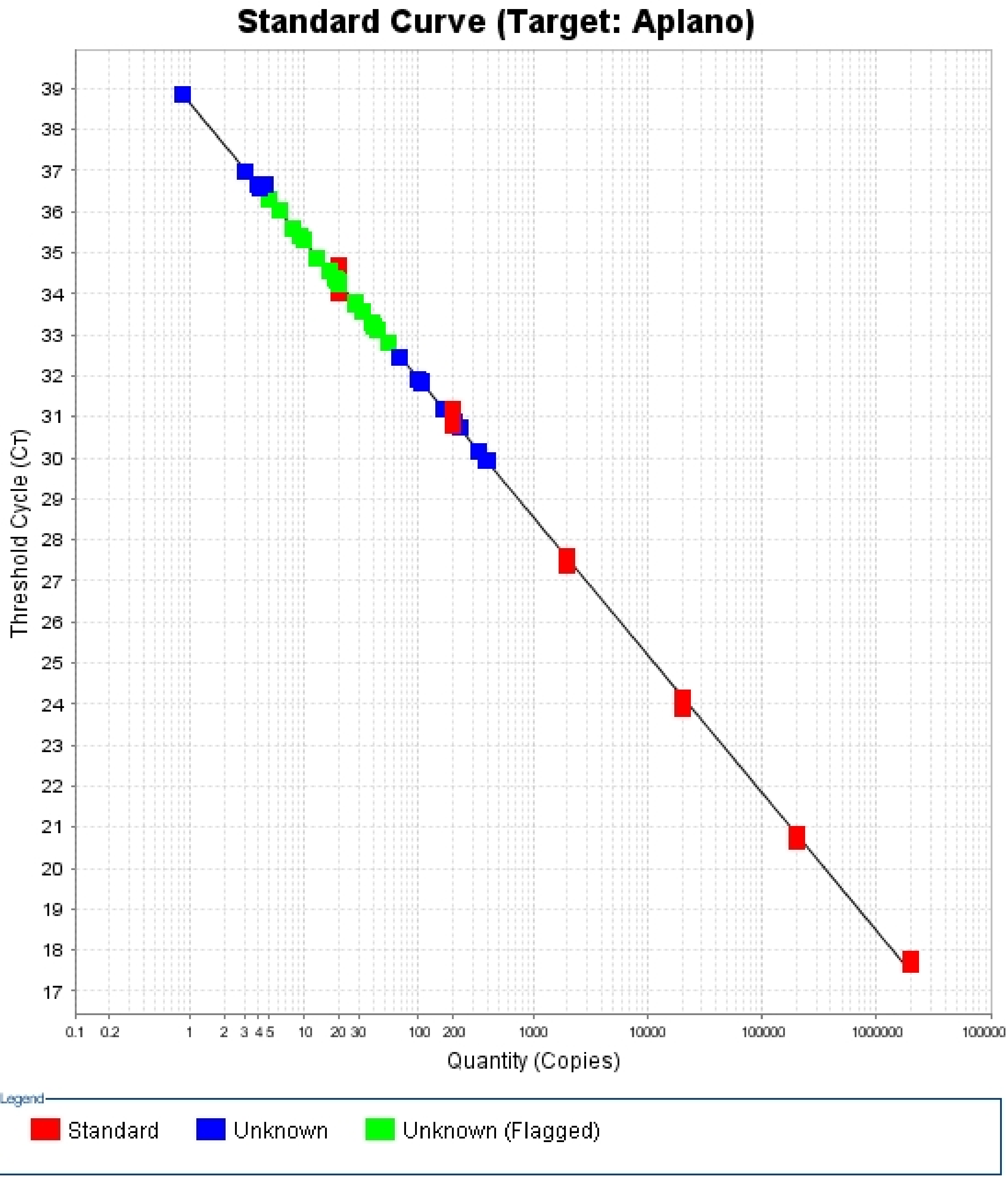

slope:-3.356 Y-Intercept:38.636 R^2:0.998 Eff%:98.589

Results Table

Well		Sample	Target	Task	Quantity	C
A1			Aplano	STANDARD	2000000	17.7647
A2			Aplano	STANDARD	2000000	17.6336
A3		2201 A01 10m	Aplano	UNKNOWN	40.9379	33.2255
A4		2201 A01 10m	Aplano	UNKNOWN	19.8186	34.2829
A5		2201 A01 10m	Aplano	UNKNOWN	16.6437	34.5373
A6		2201 A07 SCM	Aplano	UNKNOWN	108.3169	31.8073
A7		2201 A07 SCM	Aplano	UNKNOWN	69.3404	32.4574
A8		2201 A07 SCM	Aplano	UNKNOWN	99.6642	31.9286
A9		2201 A11 SCM	Aplano	UNKNOWN	397.0181	29.914
A10		2201 A11 SCM	Aplano	UNKNOWN	338.6636	30.1457
A11		2201 A11 SCM	Aplano	UNKNOWN	394.6009	29.9229
B1			Aplano	STANDARD	200000	20.6537
B2			Aplano	STANDARD	200000	20.8209
B3		2201 A01 SCM	Aplano	UNKNOWN	54.6558	32.8042
B4		2201 A01 SCM	Aplano	UNKNOWN	32.8218	33.5476
B5		2201 A01 SCM	Aplano	UNKNOWN	13.0894	34.8875
B6		2201 A07 200m	Aplano	UNKNOWN	105.4025	31.847
B7		2201 A07 200m	Aplano	UNKNOWN	55.0142	32.7947
B8		2201 A07 200m	Aplano	UNKNOWN	4.6596	36.6687
B9		2201 A11 200m	Aplano	UNKNOWN	3.8852	36.6579
B10		2201 A11 200m	Aplano	UNKNOWN	3.1086	36.983
B11		2201 A11 200m	Aplano	UNKNOWN	4.0694	36.5904
C1			Aplano	STANDARD	20000	24.1447
C2			Aplano	STANDARD	20000	23.8838
C3		2201 A04 10m	Aplano	UNKNOWN	9.1104	35.4157
C4		2201 A04 10m	Aplano	UNKNOWN	28.2463	33.7664
C5		2201 A04 10m	Aplano	UNKNOWN	55.5117	32.7816
C6		2201 A07	Aplano	UNKNOWN		Undetermined
C7		2201 A07	Aplano	UNKNOWN		Undetermined
C8		2201 A07	Aplano	UNKNOWN	0.8586	38.8583

Well		Sample	Target	Task	Quantity	C
C9		2201 A11	Aplano	UNKNOWN		Undetermined
C10		2201 A11	Aplano	UNKNOWN		Undetermined
C11		2201 A11	Aplano	UNKNOWN		Undetermined
D1			Aplano	STANDARD	2000	27.61
D2			Aplano	STANDARD	2000	27.3626
D3		2201 A04 SCM	Aplano	UNKNOWN	20.414	34.2397
D4		2201 A04 SCM	Aplano	UNKNOWN	38.9074	33.2996
D5		2201 A04 SCM	Aplano	UNKNOWN	19.4208	34.3124
D6		2201 A07	Aplano	UNKNOWN		Undetermined
D7		2201 A07	Aplano	UNKNOWN		Undetermined
D8		2201 A07	Aplano	UNKNOWN		Undetermined
D9		2201 A11	Aplano	UNKNOWN		Undetermined
D10		2201 A11	Aplano	UNKNOWN	3.9011	36.6519
D11		2201 A11	Aplano	UNKNOWN	8.0885	35.5891
E1			Aplano	STANDARD	200	31.2111
E2			Aplano	STANDARD	200	30.7721
E3		2201 A04 200m	Aplano	UNKNOWN	18.3893	34.392
E4		2201 A04 200m	Aplano	UNKNOWN	44.0682	33.1181
E5		2201 A04 200m	Aplano	UNKNOWN	4.8956	36.321
E9		2201 A21 SCM	Aplano	UNKNOWN	163.297	31.2089
E10		2201 A21 SCM	Aplano	UNKNOWN	228.4139	30.7198
E11		2201 A21 SCM	Aplano	UNKNOWN	205.1293	30.8765
F1			Aplano	STANDARD	20	34.0268
F2			Aplano	STANDARD	20	34.6646
F3		2201 A04	Aplano	UNKNOWN		Undetermined
F4		2201 A04	Aplano	UNKNOWN		Undetermined
F5		2201 A04	Aplano	UNKNOWN		Undetermined
F9		2201 A21 200m	Aplano	UNKNOWN	9.9001	35.2945
F10		2201 A21 200m	Aplano	UNKNOWN	27.7791	33.7907
F11		2201 A21 200m	Aplano	UNKNOWN	6.0575	36.0106
G3		2201 A04	Aplano	UNKNOWN		Undetermined
G4		2201 A04	Aplano	UNKNOWN		Undetermined

Well		Sample	Target	Task	Quantity	C
G5		2201 A04	Aplano	UNKNOWN		Undetermined
G9		2201 A21	Aplano	UNKNOWN		Undetermined
G10		2201 A21	Aplano	UNKNOWN		Undetermined
G11		2201 A21	Aplano	UNKNOWN		Undetermined
H1			Aplano	NTC		Undetermined
H2			Aplano	NTC		Undetermined
H9		2201 A21	Aplano	UNKNOWN		Undetermined
H10		2201 A21	Aplano	UNKNOWN		Undetermined
H11		2201 A21	Aplano	UNKNOWN		Undetermined

QC Summary

Total Wells	96	Processed Wells	71	Targets Used	1
Well Setup	71	Flagged Wells	41	Samples Used	19

Flag	Name	Frequency	Locations
AMPNC	Amplification in negative control	0	
BADROX	Bad passive reference signal	0	
BLFAIL	Baseline algorithm failed	0	
CTFAIL	C algorithm failed	0	
EXPFAIL	Exponential algorithm failed	21	C6, C7, C9, C10, C11, D6, D7, D8, D9, F3, F4, F5, G3, G4, G5, G9, G10, G11, H9, H10, H11
HIGHSD	High standard deviation in replicate group	20	A3, A4, A5, B3, B4, B5, C3, C4, C5, D3, D4, D5, D10, D11, E3, E4, E5, F9, F10, F11
NOAMP	No amplification	4	C10, D7, D9, G5
NOISE	Noise higher than others in plate	0	
NOSIGNAL	No signal in well	0	
OFFSCALE	Fluorescence is offscale	0	
OUTLIERRG	Outlier in replicate group	0	
SPIKE	Noise spikes	0	
THOLDFAIL	Thresholding algorithm failed	0	
