# Supplementary material for: Ecological impact of algal-preying protists aplanochytrids (Labyrinthulea) as a source of docosahexaenoic acid for copepods in marine environments
Source: ISME Commun. 2026 Jun 26;6(1):ycag178. doi: 10.1093/ismeco/ycag178 (PMC13685785; doi:10.1093/ismeco/ycag178)
Supplement: Supplementary file 2 [file supplementary_materials_ycag178.zip › Morimoto_ISME_C_3rdrev_supplemental_data.zip!/Morimoto_ISME_C_3rdrev_supplemental_data/Supplemental_Data_2/Standard Curve/Aplanochytrid/2201/Aplano2201AlineN=1.pdf]

Experiment Results Report

230919Aplano2201AlineN=1

Experiment Summary

Experiment Name	:	230919Aplano2201AlineN=1
Experiment Type	:	Quantitation - Standard Curve
File Name	:	230919Aplano2201AlineN=1.eds
Run Started	:	2023 Sep 19 4:37:30 PM
Run Finished	:	2023 Sep 19 6:16:50 PM
Run Duration	:	99 minutes 19 seconds
Date Modified	:	2023 Sep 20 1:32:09 PM
User	:	
Number of wells used	:	68
Number of wells with results	:	68
Instrument Name	:	
Instrument Type	:	Applied Biosystems StepOnePlus™ Instrument
Comments	:	

Results Summary

Sample	Target	Quantity (Mean)	Quantity (Std Dev)	C (Mean)	C (Std Dev)
2201 A01 10m saki1	Aplano	56.92	23.35	32.81	0.63
2201 A01 SCM saki1	Aplano	67.40	21.52	32.52	0.56
2201 A04 1000m	Aplano	15.95	7.92	34.85	0.92
2201 A04 200m	Aplano	16.64	1.42	34.62	0.13
2201 A04 3000m	Aplano	14.04	7.70	35.11	1.13
2201 A04 SCM saki1	Aplano	62.71	3.26	32.57	0.08
2201 A07 1000m	Aplano	11.22	8.97	35.53	1.40
2201 A07 200m	Aplano	44.01	17.10	33.19	0.57
2201 A07 3000m	Aplano	20.35	9.53	34.46	0.88
2201 A07 SCM saki1	Aplano	180.91	22.74	30.94	0.19
2201 A11 1000m	Aplano	28.94	7.93	33.81	0.45
2201 A11 200m	Aplano	12.63	4.60	35.12	0.62
2201 A11 3000m	Aplano	12.30	1.36	35.09	0.17
2201 A11 SCM saki1	Aplano	538.87	117.34	29.28	0.33
2201 A21 1000m	Aplano	4.84	1.44	36.58	0.49
2201 A21 200m	Aplano	27.20	18.18	34.28	1.60
2201 A21 3000m	Aplano	42.18	13.29	33.24	0.55
2201 A21 SCM saki1	Aplano	196.83	20.30	30.81	0.16

Plate Layout

	1	2	3	4	5	6	7	8	9	10	11	12
A	S Aplano<br>2E6<br>FAM-NFQ-MGB<br>CT: 17	S Aplano<br>2E6<br>FAM-NFQ-MGB<br>CT: 16.5	⚠1<br>2201 A01 10m saki1<br>U Aplano<br>36.43<br>FAM-NFQ-MGB<br>CT: 33.41	⚠1<br>2201 A01 10m saki1<br>U Aplano<br>82.33<br>FAM-NFQ-MGB<br>CT: 32.15	⚠1<br>2201 A01 10m saki1<br>U Aplano<br>51.98<br>FAM-NFQ-MGB<br>CT: 32.86	2201 A07 SCM saki1<br>U Aplano<br>175.44<br>FAM-NFQ-MGB<br>CT: 30.98	2201 A07 SCM saki1<br>U Aplano<br>161.41<br>FAM-NFQ-MGB<br>CT: 31.11	2201 A07 SCM saki1<br>U Aplano<br>205.89<br>FAM-NFQ-MGB<br>CT: 30.74	2201 A21 SCM saki1<br>U Aplano<br>173.71<br>FAM-NFQ-MGB<br>CT: 31	2201 A21 SCM saki1<br>U Aplano<br>211.76<br>FAM-NFQ-MGB<br>CT: 30.69	2201 A21 SCM saki1<br>U Aplano<br>205.01<br>FAM-NFQ-MGB<br>CT: 30.74	
B	S Aplano<br>2E5<br>FAM-NFQ-MGB<br>CT: 20.14	S Aplano<br>2E5<br>FAM-NFQ-MGB<br>CT: 20.24	⚠2<br>2201 A01 SCM saki1<br>U Aplano<br>42.57<br>FAM-NFQ-MGB<br>CT: 33.17	⚠1<br>2201 A01 SCM saki1<br>U Aplano<br>80.5<br>FAM-NFQ-MGB<br>CT: 32.19	⚠1<br>2201 A01 SCM saki1<br>U Aplano<br>79.14<br>FAM-NFQ-MGB<br>CT: 32.21	⚠1<br>2201 A07 200m saki1<br>U Aplano<br>31.52<br>FAM-NFQ-MGB<br>CT: 33.63	⚠1<br>2201 A07 200m saki1<br>U Aplano<br>37.02<br>FAM-NFQ-MGB<br>CT: 33.39	⚠1<br>2201 A07 200m saki1<br>U Aplano<br>63.5<br>FAM-NFQ-MGB<br>CT: 32.55	⚠2<br>2201 A21 200m saki1<br>U Aplano<br>6.24<br>FAM-NFQ-MGB<br>CT: 36.13	⚠1<br>2201 A21 200m saki1<br>U Aplano<br>36.73<br>FAM-NFQ-MGB<br>CT: 33.4	⚠1<br>2201 A21 200m saki1<br>U Aplano<br>38.64<br>FAM-NFQ-MGB<br>CT: 33.32	
C	S Aplano<br>2E4<br>FAM-NFQ-MGB<br>CT: 23.35	S Aplano<br>2E4<br>FAM-NFQ-MGB<br>CT: 23.33	2201 A04 SCM saki1<br>U Aplano<br>66.26<br>FAM-NFQ-MGB<br>CT: 32.49	2201 A04 SCM saki1<br>U Aplano<br>59.84<br>FAM-NFQ-MGB<br>CT: 32.64	2201 A04 SCM saki1<br>U Aplano<br>62.03<br>FAM-NFQ-MGB<br>CT: 32.59	⚠1<br>2201 A07 1000m saki1<br>U Aplano<br>17.56<br>FAM-NFQ-MGB<br>CT: 34.54	⚠1<br>2201 A07 1000m saki1<br>U Aplano<br>FAM-NFQ-MGB<br>CT: Undetermined	⚠1<br>2201 A07 1000m saki1<br>U Aplano<br>4.88<br>FAM-NFQ-MGB<br>CT: 36.51	2201 A21 1000m saki1<br>U Aplano<br>5.11<br>FAM-NFQ-MGB<br>CT: 36.44	2201 A21 1000m saki1<br>U Aplano<br>6.13<br>FAM-NFQ-MGB<br>CT: 36.16	2201 A21 1000m saki1<br>U Aplano<br>3.29<br>FAM-NFQ-MGB<br>CT: 37.12	
D	S Aplano<br>2E3<br>FAM-NFQ-MGB<br>CT: 27.21	S Aplano<br>2E3<br>FAM-NFQ-MGB<br>CT: 27.23	2201 A04 200m saki1<br>U Aplano<br>15.45<br>FAM-NFQ-MGB<br>CT: 34.73	2201 A04 200m saki1<br>U Aplano<br>16.26<br>FAM-NFQ-MGB<br>CT: 34.66	2201 A04 200m saki1<br>U Aplano<br>18.22<br>FAM-NFQ-MGB<br>CT: 34.48	⚠1<br>2201 A07 3000m saki1<br>U Aplano<br>9.64<br>FAM-NFQ-MGB<br>CT: 35.46	⚠1<br>2201 A07 3000m saki1<br>U Aplano<br>23.5<br>FAM-NFQ-MGB<br>CT: 34.09	⚠1<br>2201 A07 3000m saki1<br>U Aplano<br>27.9<br>FAM-NFQ-MGB<br>CT: 33.82	⚠1<br>2201 A21 3000m saki1<br>U Aplano<br>51.62<br>FAM-NFQ-MGB<br>CT: 32.87	⚠1<br>2201 A21 3000m saki1<br>U Aplano<br>47.94<br>FAM-NFQ-MGB<br>CT: 32.99	⚠1<br>2201 A21 3000m saki1<br>U Aplano<br>26.99<br>FAM-NFQ-MGB<br>CT: 33.87	
E	S Aplano<br>200<br>FAM-NFQ-MGB<br>CT: 30.42	S Aplano<br>200<br>FAM-NFQ-MGB<br>CT: 30.71	⚠1<br>2201 A04 1000m saki1<br>U Aplano<br>7.36<br>FAM-NFQ-MGB<br>CT: 35.88	⚠1<br>2201 A04 1000m saki1<br>U Aplano<br>17.51<br>FAM-NFQ-MGB<br>CT: 34.54	⚠1<br>2201 A04 1000m saki1<br>U Aplano<br>22.97<br>FAM-NFQ-MGB<br>CT: 34.12	2201 A11 SCM saki1<br>U Aplano<br>433.03<br>FAM-NFQ-MGB<br>CT: 29.59	2201 A11 SCM saki1<br>U Aplano<br>518.52<br>FAM-NFQ-MGB<br>CT: 29.31	2201 A11 SCM saki1<br>U Aplano<br>665.06<br>FAM-NFQ-MGB<br>CT: 28.93				
F	S Aplano<br>20<br>FAM-NFQ-MGB<br>CT: 34.4	S Aplano<br>20<br>FAM-NFQ-MGB<br>CT: 34.86	⚠1<br>2201 A04 3000m saki1<br>U Aplano<br>19.38<br>FAM-NFQ-MGB<br>CT: 34.39	⚠1<br>2201 A04 3000m saki1<br>U Aplano<br>5.21<br>FAM-NFQ-MGB<br>CT: 36.41	⚠1<br>2201 A04 3000m saki1<br>U Aplano<br>17.52<br>FAM-NFQ-MGB<br>CT: 34.54	⚠1<br>2201 A11 200m saki1<br>U Aplano<br>16.88<br>FAM-NFQ-MGB<br>CT: 34.6	⚠1<br>2201 A11 200m saki1<br>U Aplano<br>13.26<br>FAM-NFQ-MGB<br>CT: 34.97	⚠1<br>2201 A11 200m saki1<br>U Aplano<br>7.74<br>FAM-NFQ-MGB<br>CT: 35.8				
G						2201 A11 1000m saki1<br>U Aplano<br>35.87<br>FAM-NFQ-MGB<br>CT: 33.43	2201 A11 1000m saki1<br>U Aplano<br>30.66<br>FAM-NFQ-MGB<br>CT: 33.68	2201 A11 1000m saki1<br>U Aplano<br>20.28<br>FAM-NFQ-MGB<br>CT: 34.31				
H	N Aplano<br>FAM-NFQ-MGB<br>CT: Undetermined	N Aplano<br>FAM-NFQ-MGB<br>CT: Undetermined				2201 A11 3000m saki1<br>U Aplano<br>12.01<br>FAM-NFQ-MGB<br>CT: 35.12	2201 A11 3000m saki1<br>U Aplano<br>13.79<br>FAM-NFQ-MGB<br>CT: 34.91	2201 A11 3000m saki1<br>U Aplano<br>11.12<br>FAM-NFQ-MGB<br>CT: 35.24				

Standard Curves

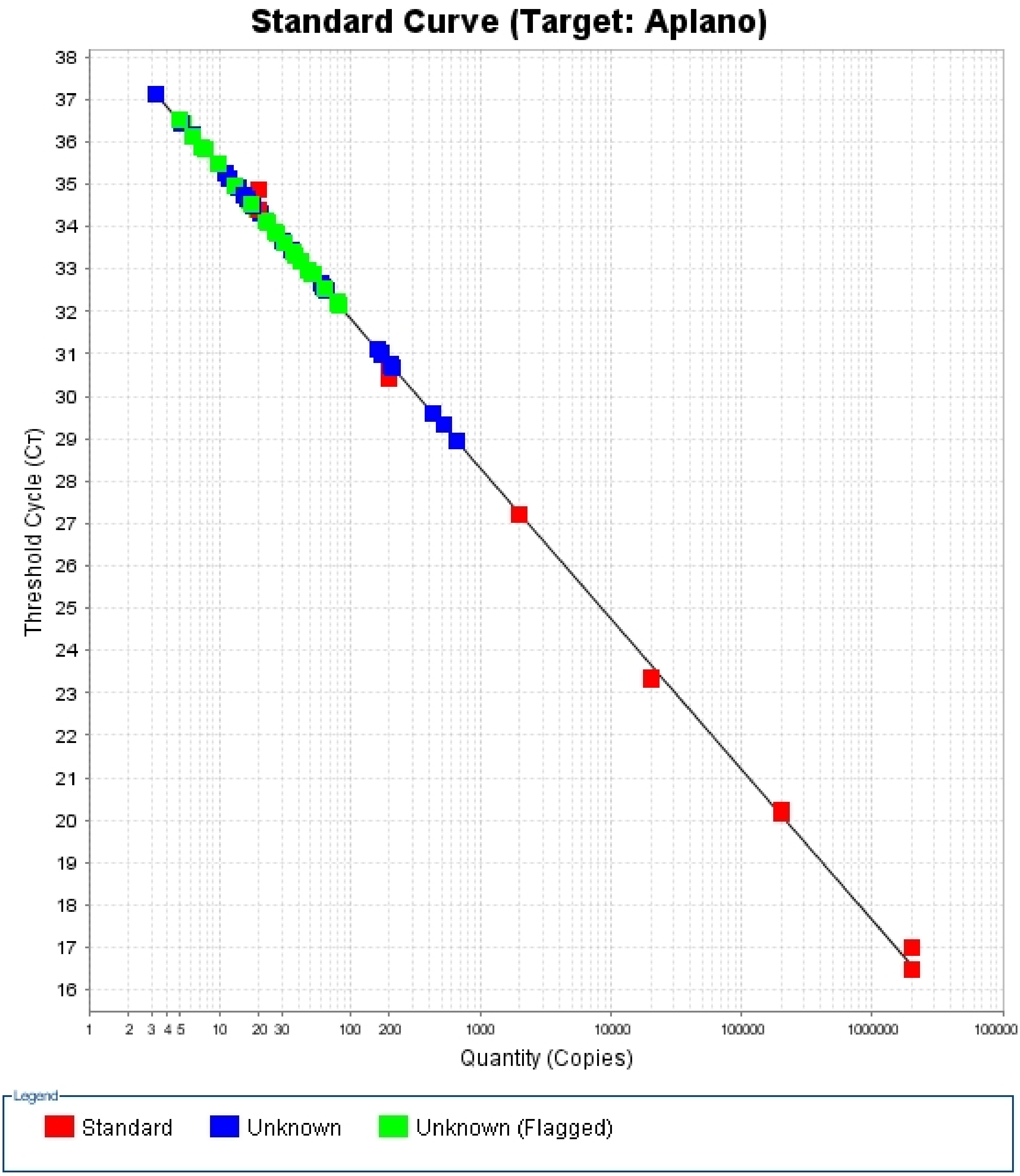

slope:-3.555 Y-Intercept:38.961 R^2:0.998 Eff%:91.129

Results Table

Well		Sample	Target	Task	Quantity	C
A1			Aplano	STANDARD	2000000	16.9961
A2			Aplano	STANDARD	2000000	16.4988
A3		2201 A01 10m	Aplano	UNKNOWN	36.4281	33.4104
A4		2201 A01 10m	Aplano	UNKNOWN	82.3343	32.1516
A5		2201 A01 10m	Aplano	UNKNOWN	51.983	32.8615
A6		2201 A07 SCM	Aplano	UNKNOWN	175.4393	30.9837
A7		2201 A07 SCM	Aplano	UNKNOWN	161.409	31.1124
A8		2201 A07 SCM	Aplano	UNKNOWN	205.8889	30.7367
A9		2201 A21 SCM	Aplano	UNKNOWN	173.7116	30.999
A10		2201 A21 SCM	Aplano	UNKNOWN	211.7632	30.6932
A11		2201 A21 SCM	Aplano	UNKNOWN	205.0053	30.7433
B1			Aplano	STANDARD	200000	20.1395
B2			Aplano	STANDARD	200000	20.2446
B3		2201 A01 SCM	Aplano	UNKNOWN	42.5702	33.1699
B4		2201 A01 SCM	Aplano	UNKNOWN	80.5019	32.1863
B5		2201 A01 SCM	Aplano	UNKNOWN	79.1419	32.2126
B6		2201 A07 200m	Aplano	UNKNOWN	31.519	33.6339
B7		2201 A07 200m	Aplano	UNKNOWN	37.0172	33.3857
B8		2201 A07 200m	Aplano	UNKNOWN	63.503	32.5525
B9		2201 A21 200m	Aplano	UNKNOWN	6.2416	36.1337
B10		2201 A21 200m	Aplano	UNKNOWN	36.7264	33.3978
B11		2201 A21 200m	Aplano	UNKNOWN	38.636	33.3196
C1			Aplano	STANDARD	20000	23.354
C2			Aplano	STANDARD	20000	23.3272
C3		2201 A04 SCM	Aplano	UNKNOWN	66.2604	32.4869
C4		2201 A04 SCM	Aplano	UNKNOWN	59.8435	32.6441
C5		2201 A04 SCM	Aplano	UNKNOWN	62.0298	32.5887
C6		2201 A07	Aplano	UNKNOWN	17.5639	34.5366
C7		2201 A07	Aplano	UNKNOWN		Undetermined
C8		2201 A07	Aplano	UNKNOWN	4.8785	36.5141

Well		Sample	Target	Task	Quantity	C
C9		2201 A21	Aplano	UNKNOWN	5.1101	36.4425
C10		2201 A21	Aplano	UNKNOWN	6.1275	36.1622
C11		2201 A21	Aplano	UNKNOWN	3.2899	37.1223
D1			Aplano	STANDARD	2000	27.2104
D2			Aplano	STANDARD	2000	27.2299
D3		2201 A04 200m	Aplano	UNKNOWN	15.4499	34.7345
D4		2201 A04 200m	Aplano	UNKNOWN	16.261	34.6556
D5		2201 A04 200m	Aplano	UNKNOWN	18.2183	34.4801
D6		2201 A07	Aplano	UNKNOWN	9.6415	35.4625
D7		2201 A07	Aplano	UNKNOWN	23.4954	34.0874
D8		2201 A07	Aplano	UNKNOWN	27.8989	33.8222
D9		2201 A21	Aplano	UNKNOWN	51.6184	32.8724
D10		2201 A21	Aplano	UNKNOWN	47.9351	32.9866
D11		2201 A21	Aplano	UNKNOWN	26.9852	33.8736
E1			Aplano	STANDARD	200	30.4231
E2			Aplano	STANDARD	200	30.7123
E3		2201 A04	Aplano	UNKNOWN	7.3588	35.8795
E4		2201 A04	Aplano	UNKNOWN	17.5088	34.5414
E5		2201 A04	Aplano	UNKNOWN	22.969	34.1224
E6		2201 A11 SCM	Aplano	UNKNOWN	433.0314	29.5889
E7		2201 A11 SCM	Aplano	UNKNOWN	518.5212	29.3108
E8		2201 A11 SCM	Aplano	UNKNOWN	665.0581	28.9266
F1			Aplano	STANDARD	20	34.3977
F2			Aplano	STANDARD	20	34.8596
F3		2201 A04	Aplano	UNKNOWN	19.3758	34.385
F4		2201 A04	Aplano	UNKNOWN	5.2118	36.4121
F5		2201 A04	Aplano	UNKNOWN	17.5216	34.5403
F6		2201 A11 200m	Aplano	UNKNOWN	16.8832	34.5976
F7		2201 A11 200m	Aplano	UNKNOWN	13.2552	34.9711
F8		2201 A11 200m	Aplano	UNKNOWN	7.7387	35.8018
G6		2201 A11	Aplano	UNKNOWN	35.8662	33.4344
G7		2201 A11	Aplano	UNKNOWN	30.6638	33.6763

Well		Sample	Target	Task	Quantity	C
G8		2201 A11	Aplano	UNKNOWN	20.281	34.3145
H1			Aplano	NTC		Undetermined
H2			Aplano	NTC		Undetermined
H6		2201 A11	Aplano	UNKNOWN	12.0064	35.1238
H7		2201 A11	Aplano	UNKNOWN	13.7854	34.9105
H8		2201 A11	Aplano	UNKNOWN	11.1196	35.2423

QC Summary

Total Wells	96	Processed Wells	68	Targets Used	1
Well Setup	68	Flagged Wells	30	Samples Used	18

Flag	Name	Frequency	Locations
AMPNC	Amplification in negative control	0	
BADROX	Bad passive reference signal	0	
BLFAIL	Baseline algorithm failed	0	
CTFAIL	C algorithm failed	0	
EXPFAIL	Exponential algorithm failed	1	C7
HIGHSD	High standard deviation in replicate group	29	A3, A4, A5, B3, B4, B5, B6, B7, B8, B9, B10, B11, C6, C8, D6, D7, D8, D9, D10, D11, E3, E4, E5, F3, F4, F5, F6, F7, F8
NOAMP	No amplification	0	
NOISE	Noise higher than others in plate	0	
NOSIGNAL	No signal in well	0	
OFFSCALE	Fluorescence is offscale	0	
OUTLIERRG	Outlier in replicate group	2	B3, B9
SPIKE	Noise spikes	0	
THOLDFAIL	Thresholding algorithm failed	0	
