# Supplementary material for: Ecological impact of algal-preying protists aplanochytrids (Labyrinthulea) as a source of docosahexaenoic acid for copepods in marine environments
Source: ISME Commun. 2026 Jun 26;6(1):ycag178. doi: 10.1093/ismeco/ycag178 (PMC13685785; doi:10.1093/ismeco/ycag178)
Supplement: Supplementary file 2 [file supplementary_materials_ycag178.zip › Morimoto_ISME_C_3rdrev_supplemental_data.zip!/Morimoto_ISME_C_3rdrev_supplemental_data/Supplemental_Data_2/Standard Curve/DNA recovery/2110/DnaRec2110AlineA01-A07-2kaimeN=1.pdf]

Experiment Results Report

230710DnaRec2110AlineA01-A07-2kaimeN=1

Experiment Summary

Experiment Name	:	230710DnaRec2110AlineA01-A07-2kaimeN=1
Experiment Type	:	Quantitation - Standard Curve
File Name	:	230707DnaRec2110AlineA01-A07-2kaimeN=1.eds
Run Started	:	2023 Jul 10 2:35:53 PM
Run Finished	:	2023 Jul 10 4:14:43 PM
Run Duration	:	98 minutes 49 seconds
Date Modified	:	2023 Jul 10 4:18:02 PM
User	:	
Number of wells used	:	76
Number of wells with results	:	76
Instrument Name	:	
Instrument Type	:	Applied Biosystems StepOnePlus™ Instrument
Comments	:	

Results Summary

Sample	Target	Quantity (Mean)	Quantity (Std Dev)	C (Mean)	C (Std Dev)
A01 10m ato1	pGEM	54413.09	3984.55	24.57	0.11
A01 10m saki1	pGEM	19668.01	2078.98	26.08	0.16
A01 BTM ato1	pGEM	47556.05	678.37	24.77	0.02
A01 BTM saki1	pGEM	26965.36	1333.65	25.61	0.07
A04 1000m ato1	pGEM	37926.06	1491.38	25.10	0.06
A04 1000m saki1	pGEM	16701.43	1918.66	26.32	0.17
A04 200m ato1	pGEM	47845.75	1334.42	24.76	0.04
A04 200m saki1	pGEM	26695.27	340.60	25.62	0.02
A04 3000m ato1	pGEM	46711.24	2171.30	24.79	0.07
A04 3000m saki1	pGEM	18467.52	848.15	26.17	0.07
A04 SCM ato1	pGEM	42972.39	2144.02	24.92	0.07
A04 SCM saki1	pGEM	20807.39	154.52	25.99	0.01
A07 1000m ato1	pGEM	45181.32	118.51	24.84	0.00
A07 1000m saki1	pGEM	25756.69	485.47	25.67	0.03
A07 200m ato1	pGEM	50434.36	1263.32	24.68	0.04
A07 200m saki1	pGEM	28823.52	1906.25	25.51	0.10
A07 3000m ato1	pGEM	47978.05	1517.42	24.75	0.05
A07 3000m saki1	pGEM	26928.62	2944.71	25.61	0.16
A07 SCM ato1	pGEM	49786.47	4282.08	24.70	0.13
A07 SCM saki1	pGEM	24593.09	2143.24	25.75	0.13
Comf2E4	pGEM	22500.12	2411.72	25.88	0.16

Plate Layout

	1	2	3	4	5	6	7	8	9	10	11	12
A	S pGEM<br>2E6<br>FAM-NFQ-MGB<br>CT: 19.46	S pGEM<br>2E6<br>FAM-NFQ-MGB<br>CT: 19.68	A01 10m saki1<br>U pGEM<br>1.78E4<br>FAM-NFQ-MGB<br>CT: 26.22	A01 10m saki1<br>U pGEM<br>1.93E4<br>FAM-NFQ-MGB<br>CT: 26.1	A01 10m saki1<br>U pGEM<br>2.19E4<br>FAM-NFQ-MGB<br>CT: 25.92	A04 1000m saki1<br>U pGEM<br>1.59E4<br>FAM-NFQ-MGB<br>CT: 26.39	A04 1000m saki1<br>U pGEM<br>1.53E4<br>FAM-NFQ-MGB<br>CT: 26.44	A04 1000m saki1<br>U pGEM<br>1.89E4<br>FAM-NFQ-MGB<br>CT: 26.13	A07 1000m saki1<br>U pGEM<br>2.58E4<br>FAM-NFQ-MGB<br>CT: 25.67	A07 1000m saki1<br>U pGEM<br>2.53E4<br>FAM-NFQ-MGB<br>CT: 25.7	A07 1000m saki1<br>U pGEM<br>2.62E4<br>FAM-NFQ-MGB<br>CT: 25.65	
B	S pGEM<br>2E5<br>FAM-NFQ-MGB<br>CT: 22.28	S pGEM<br>2E5<br>FAM-NFQ-MGB<br>CT: 22.18	A01 10m ato1<br>U pGEM<br>5.87E4<br>FAM-NFQ-MGB<br>CT: 24.45	A01 10m ato1<br>U pGEM<br>5.36E4<br>FAM-NFQ-MGB<br>CT: 24.59	A01 10m ato1<br>U pGEM<br>5.09E4<br>FAM-NFQ-MGB<br>CT: 24.66	A04 1000m ato1<br>U pGEM<br>3.74E4<br>FAM-NFQ-MGB<br>CT: 25.12	A04 1000m ato1<br>U pGEM<br>3.96E4<br>FAM-NFQ-MGB<br>CT: 25.04	A04 1000m ato1<br>U pGEM<br>3.68E4<br>FAM-NFQ-MGB<br>CT: 25.15	A07 1000m ato1<br>U pGEM<br>4.51E4<br>FAM-NFQ-MGB<br>CT: 24.84	A07 1000m ato1<br>U pGEM<br>4.51E4<br>FAM-NFQ-MGB<br>CT: 24.84	A07 1000m ato1<br>U pGEM<br>4.53E4<br>FAM-NFQ-MGB<br>CT: 24.84	
C	S pGEM<br>2E4<br>FAM-NFQ-MGB<br>CT: 26.1	S pGEM<br>2E4<br>FAM-NFQ-MGB<br>CT: 25.9	A01 BTM saki1<br>U pGEM<br>2.78E4<br>FAM-NFQ-MGB<br>CT: 25.56	A01 BTM saki1<br>U pGEM<br>2.77E4<br>FAM-NFQ-MGB<br>CT: 25.57	A01 BTM saki1<br>U pGEM<br>2.54E4<br>FAM-NFQ-MGB<br>CT: 25.69	A04 3000m saki1<br>U pGEM<br>1.75E4<br>FAM-NFQ-MGB<br>CT: 26.25	A04 3000m saki1<br>U pGEM<br>1.92E4<br>FAM-NFQ-MGB<br>CT: 26.11	A04 3000m saki1<br>U pGEM<br>1.87E4<br>FAM-NFQ-MGB<br>CT: 26.15	A07 3000m saki1<br>U pGEM<br>2.38E4<br>FAM-NFQ-MGB<br>CT: 25.79	A07 3000m saki1<br>U pGEM<br>2.97E4<br>FAM-NFQ-MGB<br>CT: 25.46	A07 3000m saki1<br>U pGEM<br>2.73E4<br>FAM-NFQ-MGB<br>CT: 25.59	
D	S pGEM<br>2E3<br>FAM-NFQ-MGB<br>CT: 29.41	S pGEM<br>2E3<br>FAM-NFQ-MGB<br>CT: 29.43	A01 BTM ato1<br>U pGEM<br>4.73E4<br>FAM-NFQ-MGB<br>CT: 24.77	A01 BTM ato1<br>U pGEM<br>4.83E4<br>FAM-NFQ-MGB<br>CT: 24.74	A01 BTM ato1<br>U pGEM<br>4.71E4<br>FAM-NFQ-MGB<br>CT: 24.78	A04 3000m ato1<br>U pGEM<br>4.42E4<br>FAM-NFQ-MGB<br>CT: 24.87	A04 3000m ato1<br>U pGEM<br>4.81E4<br>FAM-NFQ-MGB<br>CT: 24.75	A04 3000m ato1<br>U pGEM<br>4.79E4<br>FAM-NFQ-MGB<br>CT: 24.76	A07 3000m ato1<br>U pGEM<br>4.87E4<br>FAM-NFQ-MGB<br>CT: 24.73	A07 3000m ato1<br>U pGEM<br>4.9E4<br>FAM-NFQ-MGB<br>CT: 24.72	A07 3000m ato1<br>U pGEM<br>4.62E4<br>FAM-NFQ-MGB<br>CT: 24.81	
E	S pGEM<br>200<br>FAM-NFQ-MGB<br>CT: 33.05	S pGEM<br>200<br>FAM-NFQ-MGB<br>CT: 32.92	A04 SCM saki1<br>U pGEM<br>2.1E4<br>FAM-NFQ-MGB<br>CT: 25.98	A04 SCM saki1<br>U pGEM<br>2.06E4<br>FAM-NFQ-MGB<br>CT: 26	A04 SCM saki1<br>U pGEM<br>2.08E4<br>FAM-NFQ-MGB<br>CT: 25.99	A07 SCM saki1<br>U pGEM<br>2.25E4<br>FAM-NFQ-MGB<br>CT: 25.87	A07 SCM saki1<br>U pGEM<br>2.68E4<br>FAM-NFQ-MGB<br>CT: 25.62	A07 SCM saki1<br>U pGEM<br>2.44E4<br>FAM-NFQ-MGB<br>CT: 25.75				
F	1<br>S pGEM<br>20<br>FAM-NFQ-MGB<br>CT: 36.97	1<br>S pGEM<br>20<br>FAM-NFQ-MGB<br>CT: 35.71	A04 SCM ato1<br>U pGEM<br>4.49E4<br>FAM-NFQ-MGB<br>CT: 24.85	A04 SCM ato1<br>U pGEM<br>4.07E4<br>FAM-NFQ-MGB<br>CT: 25	A04 SCM ato1<br>U pGEM<br>4.33E4<br>FAM-NFQ-MGB<br>CT: 24.9	A07 SCM ato1<br>U pGEM<br>4.54E4<br>FAM-NFQ-MGB<br>CT: 24.83	A07 SCM ato1<br>U pGEM<br>5.39E4<br>FAM-NFQ-MGB<br>CT: 24.58	A07 SCM ato1<br>U pGEM<br>5E4<br>FAM-NFQ-MGB<br>CT: 24.69				
G			A04 200m saki1<br>U pGEM<br>2.65E4<br>FAM-NFQ-MGB<br>CT: 25.63	A04 200m saki1<br>U pGEM<br>2.65E4<br>FAM-NFQ-MGB<br>CT: 25.63	A04 200m saki1<br>U pGEM<br>2.71E4<br>FAM-NFQ-MGB<br>CT: 25.6	A07 200m saki1<br>U pGEM<br>2.84E4<br>FAM-NFQ-MGB<br>CT: 25.53	A07 200m saki1<br>U pGEM<br>2.72E4<br>FAM-NFQ-MGB<br>CT: 25.59	A07 200m saki1<br>U pGEM<br>3.09E4<br>FAM-NFQ-MGB<br>CT: 25.4				N pGEM<br>FAM-NFQ-MGB<br>CT: Undetermined
H	Comf2E4<br>U pGEM<br>2.08E4<br>FAM-NFQ-MGB<br>CT: 25.99	Comf2E4<br>U pGEM<br>2.42E4<br>FAM-NFQ-MGB<br>CT: 25.77	A04 200m ato1<br>U pGEM<br>4.94E4<br>FAM-NFQ-MGB<br>CT: 24.71	A04 200m ato1<br>U pGEM<br>4.69E4<br>FAM-NFQ-MGB<br>CT: 24.79	A04 200m ato1<br>U pGEM<br>4.73E4<br>FAM-NFQ-MGB<br>CT: 24.77	A07 200m ato1<br>U pGEM<br>5.15E4<br>FAM-NFQ-MGB<br>CT: 24.65	A07 200m ato1<br>U pGEM<br>4.91E4<br>FAM-NFQ-MGB<br>CT: 24.72	A07 200m ato1<br>U pGEM<br>5.07E4<br>FAM-NFQ-MGB<br>CT: 24.67				N pGEM<br>FAM-NFQ-MGB<br>CT: Undetermined

Standard Curves

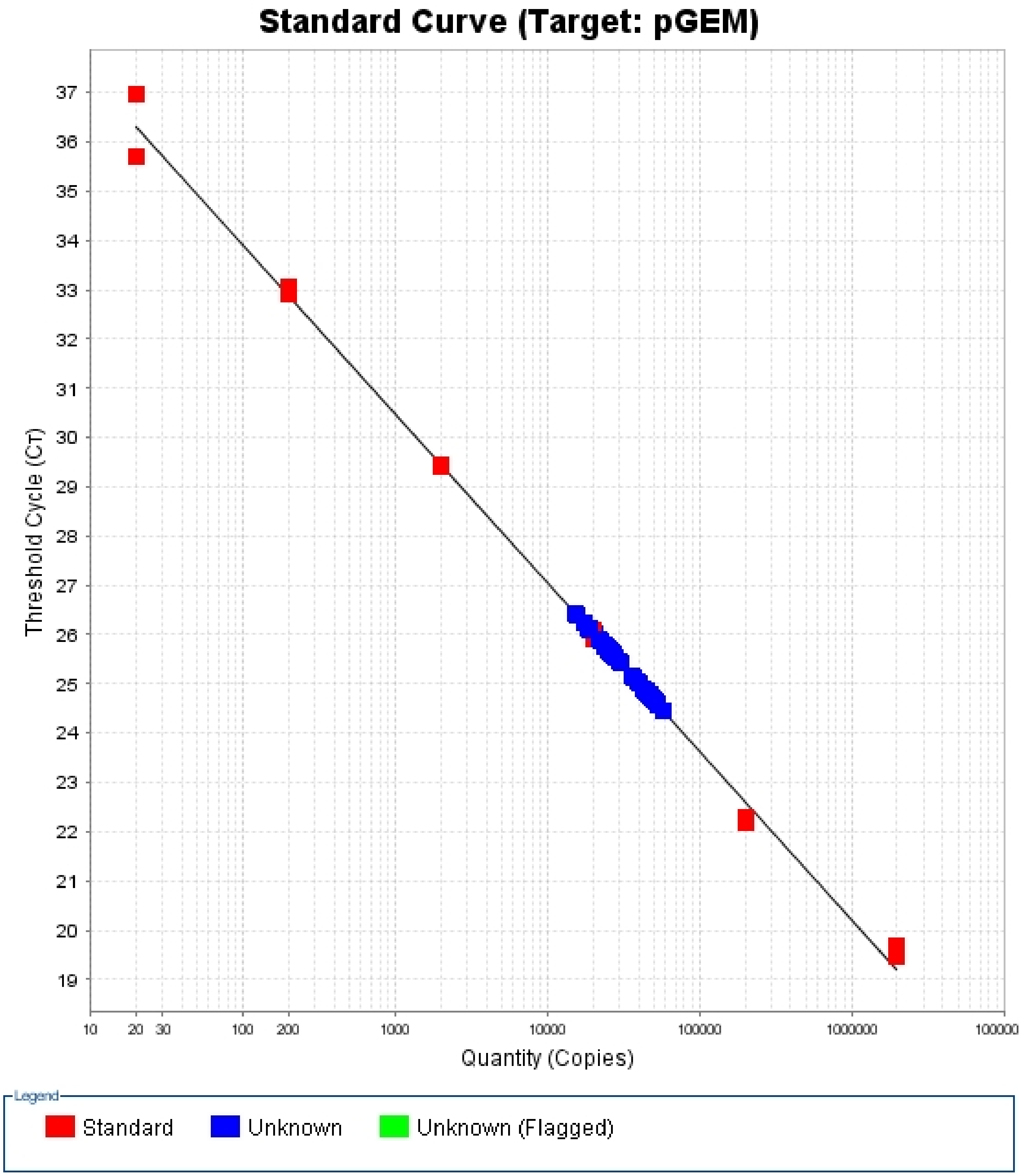

slope:-3.415 Y-Intercept:40.74 R^2:0.996 Eff%:96.242

Results Table

Well		Sample	Target	Task	Quantity	C
A1			pGEM	STANDARD	2000000	19.4627
A2			pGEM	STANDARD	2000000	19.6774
A3		A01 10m saki1	pGEM	UNKNOWN	17785.3223	26.224
A4		A01 10m saki1	pGEM	UNKNOWN	19319.4863	26.1013
A5		A01 10m saki1	pGEM	UNKNOWN	21899.2266	25.9154
A6		A04 1000m	pGEM	UNKNOWN	15887.3994	26.3914
A7		A04 1000m	pGEM	UNKNOWN	15323.9932	26.445
A8		A04 1000m	pGEM	UNKNOWN	18892.8965	26.1344
A9		A07 1000m	pGEM	UNKNOWN	25766.0078	25.6742
A10		A07 1000m	pGEM	UNKNOWN	25266.6367	25.7032
A11		A07 1000m	pGEM	UNKNOWN	26237.4336	25.6473
B1			pGEM	STANDARD	200000	22.2771
B2			pGEM	STANDARD	200000	22.1812
B3		A01 10m ato1	pGEM	UNKNOWN	58747.3164	24.4517
B4		A01 10m ato1	pGEM	UNKNOWN	53582.9531	24.5882
B5		A01 10m ato1	pGEM	UNKNOWN	50908.9961	24.6641
B6		A04 1000m ato1	pGEM	UNKNOWN	37388.6289	25.122
B7		A04 1000m ato1	pGEM	UNKNOWN	39611.668	25.0363
B8		A04 1000m ato1	pGEM	UNKNOWN	36777.8906	25.1464
B9		A07 1000m ato1	pGEM	UNKNOWN	45093.4805	24.844
B10		A07 1000m ato1	pGEM	UNKNOWN	45134.3789	24.8427
B11		A07 1000m ato1	pGEM	UNKNOWN	45316.1094	24.8367
C1			pGEM	STANDARD	20000	26.1027
C2			pGEM	STANDARD	20000	25.8997
C3		A01 BTM saki1	pGEM	UNKNOWN	27762.834	25.5635
C4		A01 BTM saki1	pGEM	UNKNOWN	27707.5195	25.5665
C5		A01 BTM saki1	pGEM	UNKNOWN	25425.7207	25.6939
C6		A04 3000m	pGEM	UNKNOWN	17519.8594	26.2463
C7		A04 3000m	pGEM	UNKNOWN	19155.3809	26.114
C8		A04 3000m	pGEM	UNKNOWN	18727.3086	26.1475

Well		Sample	Target	Task	Quantity	C
C9		A07 3000m	pGEM	UNKNOWN	23832.4062	25.7899
C10		A07 3000m	pGEM	UNKNOWN	29693.8535	25.4638
C11		A07 3000m	pGEM	UNKNOWN	27259.6191	25.5906
D1			pGEM	STANDARD	2000	29.4063
D2			pGEM	STANDARD	2000	29.43
D3		A01 BTM ato1	pGEM	UNKNOWN	47281.3672	24.7738
D4		A01 BTM ato1	pGEM	UNKNOWN	48328.6875	24.7413
D5		A01 BTM ato1	pGEM	UNKNOWN	47058.0977	24.7808
D6		A04 3000m ato1	pGEM	UNKNOWN	44207.2422	24.8735
D7		A04 3000m ato1	pGEM	UNKNOWN	48072.9531	24.7491
D8		A04 3000m ato1	pGEM	UNKNOWN	47853.5117	24.7559
D9		A07 3000m ato1	pGEM	UNKNOWN	48744.4766	24.7286
D10		A07 3000m ato1	pGEM	UNKNOWN	48959.3789	24.722
D11		A07 3000m ato1	pGEM	UNKNOWN	46230.2852	24.8071
E1			pGEM	STANDARD	200	33.0454
E2			pGEM	STANDARD	200	32.9243
E3		A04 SCM saki1	pGEM	UNKNOWN	20952.6582	25.9809
E4		A04 SCM saki1	pGEM	UNKNOWN	20645.043	26.0029
E5		A04 SCM saki1	pGEM	UNKNOWN	20824.4805	25.99
E6		A07 SCM saki1	pGEM	UNKNOWN	22527.4199	25.8734
E7		A07 SCM saki1	pGEM	UNKNOWN	26806.2715	25.6155
E8		A07 SCM saki1	pGEM	UNKNOWN	24445.5703	25.7522
F1			pGEM	STANDARD	20	36.9707
F2			pGEM	STANDARD	20	35.7121
F3		A04 SCM ato1	pGEM	UNKNOWN	44941.1758	24.8491
F4		A04 SCM ato1	pGEM	UNKNOWN	40688.1055	24.9965
F5		A04 SCM ato1	pGEM	UNKNOWN	43287.9062	24.9047
F6		A07 SCM ato1	pGEM	UNKNOWN	45388.9492	24.8344
F7		A07 SCM ato1	pGEM	UNKNOWN	53942.9258	24.5783
F8		A07 SCM ato1	pGEM	UNKNOWN	50027.5234	24.69
G3		A04 200m saki1	pGEM	UNKNOWN	26532.6758	25.6307
G4		A04 200m saki1	pGEM	UNKNOWN	26466.4316	25.6344

Well		Sample	Target	Task	Quantity	C
G5		A04 200m saki1	pGEM	UNKNOWN	27086.6934	25.6001
G6		A07 200m saki1	pGEM	UNKNOWN	28374.7383	25.5312
G7		A07 200m saki1	pGEM	UNKNOWN	27181.7031	25.5949
G8		A07 200m saki1	pGEM	UNKNOWN	30914.1152	25.404
G12			pGEM	NTC		Undetermined
H1		Comf2E4	pGEM	UNKNOWN	20794.7793	25.9922
H2		Comf2E4	pGEM	UNKNOWN	24205.4668	25.7669
H3		A04 200m ato1	pGEM	UNKNOWN	49365.6758	24.7098
H4		A04 200m ato1	pGEM	UNKNOWN	46866.6055	24.7868
H5		A04 200m ato1	pGEM	UNKNOWN	47304.9648	24.773
H6		A07 200m ato1	pGEM	UNKNOWN	51542.8633	24.6458
H7		A07 200m ato1	pGEM	UNKNOWN	49058.8867	24.719
H8		A07 200m ato1	pGEM	UNKNOWN	50701.3125	24.6702
H12			pGEM	NTC		Undetermined

QC Summary

Total Wells	96	Processed Wells	76	Targets Used	1
Well Setup	76	Flagged Wells	2	Samples Used	21

Flag	Name	Frequency	Locations
AMPNC	Amplification in negative control	0	
BADROX	Bad passive reference signal	0	
BLFAIL	Baseline algorithm failed	0	
CTFAIL	C algorithm failed	0	
EXPFAIL	Exponential algorithm failed	0	
HIGHSD	High standard deviation in replicate group	2	F1, F2
NOAMP	No amplification	0	
NOISE	Noise higher than others in plate	0	
NOSIGNAL	No signal in well	0	
OFFSCALE	Fluorescence is offscale	0	
OUTLIERRG	Outlier in replicate group	0	
SPIKE	Noise spikes	0	
THOLDFAIL	Thresholding algorithm failed	0	
