# Supplementary material for: Ecological impact of algal-preying protists aplanochytrids (Labyrinthulea) as a source of docosahexaenoic acid for copepods in marine environments
Source: ISME Commun. 2026 Jun 26;6(1):ycag178. doi: 10.1093/ismeco/ycag178 (PMC13685785; doi:10.1093/ismeco/ycag178)
Supplement: Supplementary file 2 [file supplementary_materials_ycag178.zip › Morimoto_ISME_C_3rdrev_supplemental_data.zip!/Morimoto_ISME_C_3rdrev_supplemental_data/Supplemental_Data_2/Standard Curve/DNA recovery/2110/DnaRec2110AlineA11-A21N=2.pdf]

Experiment Results Report
230711DnaRec2110AlineA11-A21N=2

Experiment Summary

Experiment Name	:	230711DnaRec2110AlineA11-A21N=2
Experiment Type	:	Quantitation - Standard Curve
File Name	:	230711DnaRec2110AlineA11-A21N=2.eds
Run Started	:	2023 Jul 11 5:05:29 PM
Run Finished	:	2023 Jul 11 6:40:04 PM
Run Duration	:	94 minutes 34 seconds
Date Modified	:	2023 Jul 11 10:13:02 PM
User	:	
Number of wells used	:	70
Number of wells with results	:	70
Instrument Name	:	
Instrument Type	:	Applied Biosystems StepOnePlus™ Instrument
Comments	:	

Results Summary

Sample	Target	Quantity (Mean)	Quantity (Std Dev)	C (Mean)	C (Std Dev)
A11 1000m ato	pGEM	22844.35	2614.46	25.49	0.17
A11 1000m saki	pGEM	10509.83	715.08	26.65	0.10
A11 200m ato	pGEM	24625.88	1308.02	25.37	0.08
A11 200m saki	pGEM	11392.51	637.11	26.53	0.08
A11 3000m ato	pGEM	26719.43	536.06	25.25	0.03
A11 3000m saki	pGEM	9287.41	130.09	26.84	0.02
A11 SCM ato	pGEM	26636.96	1479.03	25.25	0.09
A11 SCM saki	pGEM	11951.08	1329.35	26.46	0.17
A21 1000m ato	pGEM	25140.49	599.70	25.34	0.04
A21 1000m saki	pGEM	6743.47	589.35	27.32	0.13
A21 10m ato	pGEM	23730.27	1555.65	25.43	0.10
A21 10m saki	pGEM	7862.31	710.21	27.09	0.14
A21 200m ato	pGEM	25880.49	1087.16	25.29	0.06
A21 200m saki	pGEM	7312.06	803.51	27.20	0.17
A21 3000m ato	pGEM	22630.03	1844.59	25.50	0.12
A21 3000m saki	pGEM	4471.68	124.03	27.94	0.04
A21 SCM ato	pGEM	26052.74	3259.74	25.29	0.18
A21 SCM saki	pGEM	8596.56	227.85	26.95	0.04
Comf2E4	pGEM	20094.88	747.28	25.68	0.06

Plate Layout

	1	2	3	4	5	6	7	8	9	10	11	12
A	S pGEM<br>2E6<br>FAM-NFQ-MGB<br>CT: 19.01	S pGEM<br>2E6<br>FAM-NFQ-MGB<br>CT: 18.64	A11 SCM saki<br>U pGEM<br>1.2E4<br>FAM-NFQ-MGB<br>CT: 26.45	A11 SCM saki<br>U pGEM<br>1.06E4<br>FAM-NFQ-MGB<br>CT: 26.64	A11 SCM saki<br>U pGEM<br>1.33E4<br>FAM-NFQ-MGB<br>CT: 26.3	A21 10m saki<br>U pGEM<br>7.88E3<br>FAM-NFQ-MGB<br>CT: 27.08	A21 10m saki<br>U pGEM<br>7.14E3<br>FAM-NFQ-MGB<br>CT: 27.23	A21 10m saki<br>U pGEM<br>8.56E3<br>FAM-NFQ-MGB<br>CT: 26.96	A21 3000m saki<br>U pGEM<br>4.36E3<br>FAM-NFQ-MGB<br>CT: 27.98	A21 3000m saki<br>U pGEM<br>4.46E3<br>FAM-NFQ-MGB<br>CT: 27.94	A21 3000m saki<br>U pGEM<br>4.6E3<br>FAM-NFQ-MGB<br>CT: 27.9	
B	S pGEM<br>2E5<br>FAM-NFQ-MGB<br>CT: 22.2	S pGEM<br>2E5<br>FAM-NFQ-MGB<br>CT: 22.02	A11 SCM ato<br>U pGEM<br>2.75E4<br>FAM-NFQ-MGB<br>CT: 25.2	A11 SCM ato<br>U pGEM<br>2.74E4<br>FAM-NFQ-MGB<br>CT: 25.21	A11 SCM ato<br>U pGEM<br>2.49E4<br>FAM-NFQ-MGB<br>CT: 25.35	A21 10m ato<br>U pGEM<br>2.31E4<br>FAM-NFQ-MGB<br>CT: 25.46	A21 10m ato<br>U pGEM<br>2.26E4<br>FAM-NFQ-MGB<br>CT: 25.5	A21 10m ato<br>U pGEM<br>2.55E4<br>FAM-NFQ-MGB<br>CT: 25.32	A21 3000m ato<br>U pGEM<br>2.22E4<br>FAM-NFQ-MGB<br>CT: 25.53	A21 3000m ato<br>U pGEM<br>2.47E4<br>FAM-NFQ-MGB<br>CT: 25.37	A21 3000m ato<br>U pGEM<br>2.1E4<br>FAM-NFQ-MGB<br>CT: 25.61	
C	S pGEM<br>2E4<br>FAM-NFQ-MGB<br>CT: 25.5	S pGEM<br>2E4<br>FAM-NFQ-MGB<br>CT: 25.5	A11 200m saki<br>U pGEM<br>1.21E4<br>FAM-NFQ-MGB<br>CT: 26.44	A11 200m saki<br>U pGEM<br>1.1E4<br>FAM-NFQ-MGB<br>CT: 26.58	A11 200m saki<br>U pGEM<br>1.1E4<br>FAM-NFQ-MGB<br>CT: 26.58	A21 SCM saki<br>U pGEM<br>8.33E3<br>FAM-NFQ-MGB<br>CT: 27	A21 SCM saki<br>U pGEM<br>8.75E3<br>FAM-NFQ-MGB<br>CT: 26.93	A21 SCM saki<br>U pGEM<br>8.71E3<br>FAM-NFQ-MGB<br>CT: 26.94				
D	S pGEM<br>2E3<br>FAM-NFQ-MGB<br>CT: 29.34	S pGEM<br>2E3<br>FAM-NFQ-MGB<br>CT: 29.28	A11 200m ato<br>U pGEM<br>2.36E4<br>FAM-NFQ-MGB<br>CT: 25.43	A11 200m ato<br>U pGEM<br>2.61E4<br>FAM-NFQ-MGB<br>CT: 25.28	A11 200m ato<br>U pGEM<br>2.42E4<br>FAM-NFQ-MGB<br>CT: 25.4	A21 SCM ato<br>U pGEM<br>2.34E4<br>FAM-NFQ-MGB<br>CT: 25.44	A21 SCM ato<br>U pGEM<br>2.97E4<br>FAM-NFQ-MGB<br>CT: 25.09	A21 SCM ato<br>U pGEM<br>2.5E4<br>FAM-NFQ-MGB<br>CT: 25.34				
E	S pGEM<br>200<br>FAM-NFQ-MGB<br>CT: 32.86	S pGEM<br>200<br>FAM-NFQ-MGB<br>CT: 32.8	A11 1000m saki<br>U pGEM<br>1.02E4<br>FAM-NFQ-MGB<br>CT: 26.7	A11 1000m saki<br>U pGEM<br>1E4<br>FAM-NFQ-MGB<br>CT: 26.72	A11 1000m saki<br>U pGEM<br>1.13E4<br>FAM-NFQ-MGB<br>CT: 26.54	A21 200m saki<br>U pGEM<br>6.41E3<br>FAM-NFQ-MGB<br>CT: 27.4	A21 200m saki<br>U pGEM<br>7.96E3<br>FAM-NFQ-MGB<br>CT: 27.07	A21 200m saki<br>U pGEM<br>7.57E3<br>FAM-NFQ-MGB<br>CT: 27.15				
F	S pGEM<br>20<br>FAM-NFQ-MGB<br>CT: 35.96	S pGEM<br>20<br>FAM-NFQ-MGB<br>CT: 35.87	A11 1000m ato<br>U pGEM<br>2.58E4<br>FAM-NFQ-MGB<br>CT: 25.3	A11 1000m ato<br>U pGEM<br>2.1E4<br>FAM-NFQ-MGB<br>CT: 25.61	A11 1000m ato<br>U pGEM<br>2.17E4<br>FAM-NFQ-MGB<br>CT: 25.56	A21 200m ato<br>U pGEM<br>2.46E4<br>FAM-NFQ-MGB<br>CT: 25.37	A21 200m ato<br>U pGEM<br>2.66E4<br>FAM-NFQ-MGB<br>CT: 25.25	A21 200m ato<br>U pGEM<br>2.64E4<br>FAM-NFQ-MGB<br>CT: 25.26				
G			A11 3000m saki<br>U pGEM<br>9.29E3<br>FAM-NFQ-MGB<br>CT: 26.84	A11 3000m saki<br>U pGEM<br>9.15E3<br>FAM-NFQ-MGB<br>CT: 26.86	A11 3000m saki<br>U pGEM<br>9.41E3<br>FAM-NFQ-MGB<br>CT: 26.82	A21 1000m saki<br>U pGEM<br>6.88E3<br>FAM-NFQ-MGB<br>CT: 27.29	A21 1000m saki<br>U pGEM<br>6.1E3<br>FAM-NFQ-MGB<br>CT: 27.47	A21 1000m saki<br>U pGEM<br>7.26E3<br>FAM-NFQ-MGB<br>CT: 27.21				N pGEM<br>FAM-NFQ-MGB<br>CT: Undetermined
H	Comf2E4<br>U pGEM<br>1.96E4<br>FAM-NFQ-MGB<br>CT: 25.72	Comf2E4<br>U pGEM<br>2.06E4<br>FAM-NFQ-MGB<br>CT: 25.64	A11 3000m ato<br>U pGEM<br>2.73E4<br>FAM-NFQ-MGB<br>CT: 25.21	A11 3000m ato<br>U pGEM<br>2.66E4<br>FAM-NFQ-MGB<br>CT: 25.25	A11 3000m ato<br>U pGEM<br>2.63E4<br>FAM-NFQ-MGB<br>CT: 25.27	A21 1000m ato<br>U pGEM<br>2.51E4<br>FAM-NFQ-MGB<br>CT: 25.34	A21 1000m ato<br>U pGEM<br>2.58E4<br>FAM-NFQ-MGB<br>CT: 25.3	A21 1000m ato<br>U pGEM<br>2.46E4<br>FAM-NFQ-MGB<br>CT: 25.37				N pGEM<br>FAM-NFQ-MGB<br>CT: Undetermined

Standard Curves

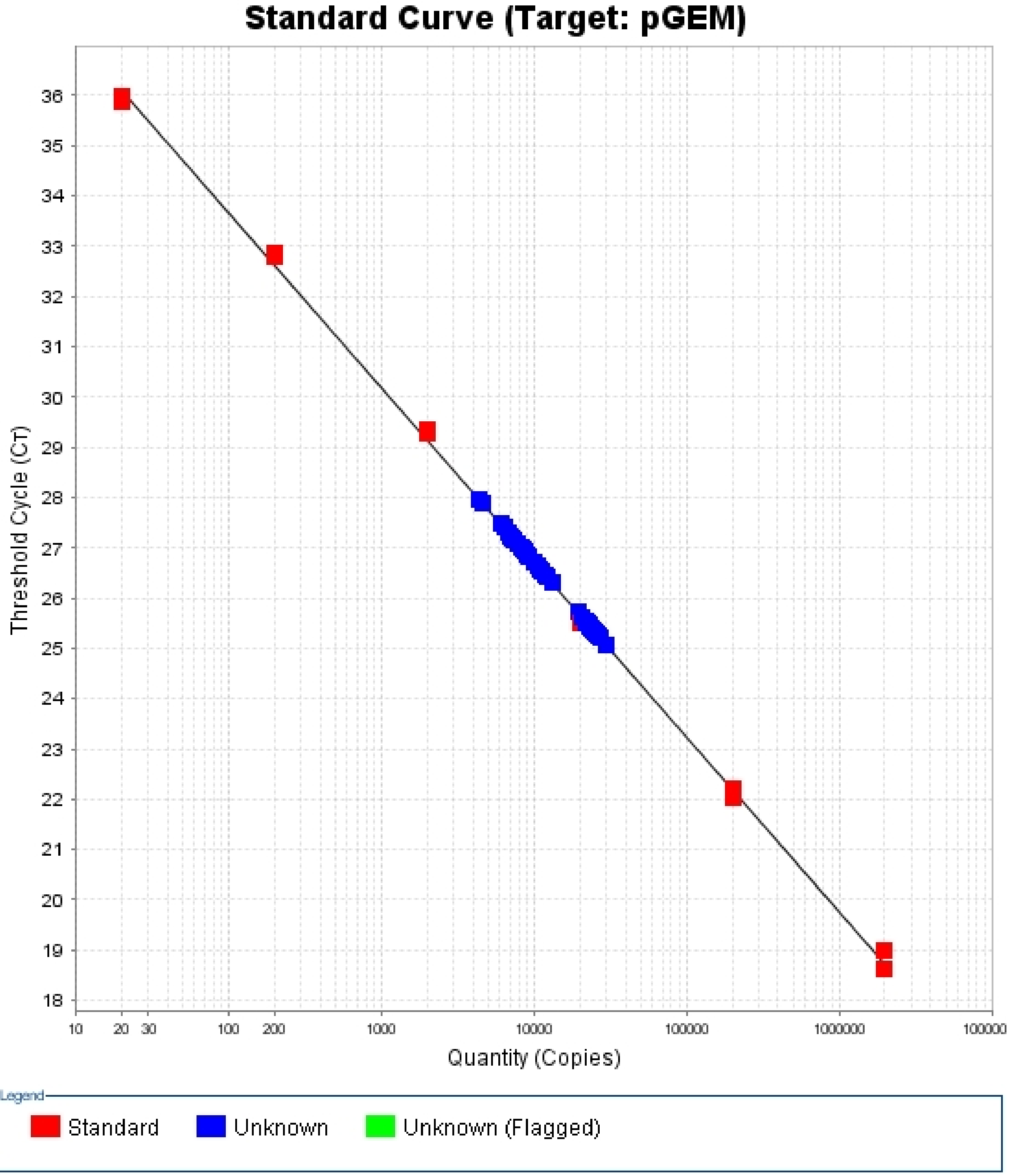

slope:-3.469 Y-Intercept:40.602 R^2:0.999 Eff%:94.212

Results Table

Well		Sample	Target	Task	Quantity	C
A1			pGEM	STANDARD	2000000	19.0095
A2			pGEM	STANDARD	2000000	18.6438
A3		A11 SCM saki	pGEM	UNKNOWN	11999.0771	26.4517
A4		A11 SCM saki	pGEM	UNKNOWN	10598.3838	26.6387
A5		A11 SCM saki	pGEM	UNKNOWN	13255.7891	26.3017
A6		A21 10m saki	pGEM	UNKNOWN	7884.3154	27.0844
A7		A21 10m saki	pGEM	UNKNOWN	7141.3545	27.2335
A8		A21 10m saki	pGEM	UNKNOWN	8561.2627	26.9603
A9		A21 3000m saki	pGEM	UNKNOWN	4356.4204	27.9781
A10		A21 3000m saki	pGEM	UNKNOWN	4455.688	27.9442
A11		A21 3000m saki	pGEM	UNKNOWN	4602.9282	27.8952
B1			pGEM	STANDARD	200000	22.1998
B2			pGEM	STANDARD	200000	22.0214
B3		A11 SCM ato	pGEM	UNKNOWN	27540.0625	25.2001
B4		A11 SCM ato	pGEM	UNKNOWN	27440.7305	25.2055
B5		A11 SCM ato	pGEM	UNKNOWN	24930.0801	25.3501
B6		A21 10m ato	pGEM	UNKNOWN	23121.3105	25.4636
B7		A21 10m ato	pGEM	UNKNOWN	22571.2168	25.4999
B8		A21 10m ato	pGEM	UNKNOWN	25498.2793	25.3162
B9		A21 3000m ato	pGEM	UNKNOWN	22186.2695	25.5258
B10		A21 3000m ato	pGEM	UNKNOWN	24656.0215	25.3668
B11		A21 3000m ato	pGEM	UNKNOWN	21047.7871	25.6051
C1			pGEM	STANDARD	20000	25.5037
C2			pGEM	STANDARD	20000	25.5047
C3		A11 200m saki	pGEM	UNKNOWN	12128.1807	26.4356
C4		A11 200m saki	pGEM	UNKNOWN	11027.3633	26.579
C5		A11 200m saki	pGEM	UNKNOWN	11021.9893	26.5797
C6		A21 SCM saki	pGEM	UNKNOWN	8334.6152	27.0007
C7		A21 SCM saki	pGEM	UNKNOWN	8748.8311	26.9277
C8		A21 SCM saki	pGEM	UNKNOWN	8706.2354	26.935

Well		Sample	Target	Task	Quantity	C
D1			pGEM	STANDARD	2000	29.3376
D2			pGEM	STANDARD	2000	29.2803
D3		A11 200m ato	pGEM	UNKNOWN	23588.2695	25.4335
D4		A11 200m ato	pGEM	UNKNOWN	26095.1816	25.2813
D5		A11 200m ato	pGEM	UNKNOWN	24194.1895	25.3952
D6		A21 SCM ato	pGEM	UNKNOWN	23413.7656	25.4446
D7		A21 SCM ato	pGEM	UNKNOWN	29696.6152	25.0865
D8		A21 SCM ato	pGEM	UNKNOWN	25047.834	25.343
E1			pGEM	STANDARD	200	32.8639
E2			pGEM	STANDARD	200	32.8015
E3		A11 1000m saki	pGEM	UNKNOWN	10181.7715	26.6992
E4		A11 1000m saki	pGEM	UNKNOWN	10017.6436	26.7236
E5		A11 1000m saki	pGEM	UNKNOWN	11330.0762	26.5382
E6		A21 200m saki	pGEM	UNKNOWN	6412.0146	27.3958
E7		A21 200m saki	pGEM	UNKNOWN	7957.1992	27.0706
E8		A21 200m saki	pGEM	UNKNOWN	7566.9531	27.1463
F1			pGEM	STANDARD	20	35.9596
F2			pGEM	STANDARD	20	35.8707
F3		A11 1000m ato	pGEM	UNKNOWN	25832.2441	25.2966
F4		A11 1000m ato	pGEM	UNKNOWN	20976.5977	25.6102
F5		A11 1000m ato	pGEM	UNKNOWN	21724.1953	25.5575
F6		A21 200m ato	pGEM	UNKNOWN	24627.6934	25.3685
F7		A21 200m ato	pGEM	UNKNOWN	26576.0996	25.2538
F8		A21 200m ato	pGEM	UNKNOWN	26437.668	25.2617
G3		A11 3000m saki	pGEM	UNKNOWN	9294.25	26.8366
G4		A11 3000m saki	pGEM	UNKNOWN	9154.0312	26.8595
G5		A11 3000m saki	pGEM	UNKNOWN	9413.9443	26.8173
G6		A21 1000m saki	pGEM	UNKNOWN	6875.1387	27.2908
G7		A21 1000m saki	pGEM	UNKNOWN	6099.4253	27.4711
G8		A21 1000m saki	pGEM	UNKNOWN	7255.8584	27.2096
G12			pGEM	NTC		Undetermined
H1		Comf2E4	pGEM	UNKNOWN	19566.4668	25.7151

Well		Sample	Target	Task	Quantity	C
H2		Comf2E4	pGEM	UNKNOWN	20623.2832	25.6358
H3		A11 3000m ato	pGEM	UNKNOWN	27312.0391	25.2126
H4		A11 3000m ato	pGEM	UNKNOWN	26577.9844	25.2537
H5		A11 3000m ato	pGEM	UNKNOWN	26268.2773	25.2713
H6		A21 1000m ato	pGEM	UNKNOWN	25085.4102	25.3407
H7		A21 1000m ato	pGEM	UNKNOWN	25765.8398	25.3004
H8		A21 1000m ato	pGEM	UNKNOWN	24570.2324	25.372
H12			pGEM	NTC		Undetermined

QC Summary

Total Wells	96	Processed Wells	70	Targets Used	1
Well Setup	70	Flagged Wells	0	Samples Used	19

Flag	Name	Frequency	Locations
AMPNC	Amplification in negative control	0	
BADROX	Bad passive reference signal	0	
BLFAIL	Baseline algorithm failed	0	
CTFAIL	C algorithm failed	0	
EXPFAIL	Exponential algorithm failed	0	
HIGHSD	High standard deviation in replicate group	0	
NOAMP	No amplification	0	
NOISE	Noise higher than others in plate	0	
NOSIGNAL	No signal in well	0	
OFFSCALE	Fluorescence is offscale	0	
OUTLIERRG	Outlier in replicate group	0	
SPIKE	Noise spikes	0	
THOLDFAIL	Thresholding algorithm failed	0	
