# Supplementary material for: Ecological impact of algal-preying protists aplanochytrids (Labyrinthulea) as a source of docosahexaenoic acid for copepods in marine environments
Source: ISME Commun. 2026 Jun 26;6(1):ycag178. doi: 10.1093/ismeco/ycag178 (PMC13685785; doi:10.1093/ismeco/ycag178)
Supplement: Supplementary file 2 [file supplementary_materials_ycag178.zip › Morimoto_ISME_C_3rdrev_supplemental_data.zip!/Morimoto_ISME_C_3rdrev_supplemental_data/Supplemental_Data_2/Standard Curve/DNA recovery/2110/DnaRec2110AlineA11-A21N=3.pdf]

Experiment Results Report

230712DnaRec2110AlineA11-A21N=3

Experiment Summary

Experiment Name	:	230712DnaRec2110AlineA11-A21N=3
Experiment Type	:	Quantitation - Standard Curve
File Name	:	230712DnaRec2110AlineA11-A21N=3.eds
Run Started	:	2023 Jul 12 2:49:12 PM
Run Finished	:	2023 Jul 12 4:23:51 PM
Run Duration	:	94 minutes 39 seconds
Date Modified	:	2023 Jul 13 10:48:04 AM
User	:	
Number of wells used	:	70
Number of wells with results	:	70
Instrument Name	:	
Instrument Type	:	Applied Biosystems StepOnePlus™ Instrument
Comments	:	

Results Summary

Sample	Target	Quantity (Mean)	Quantity (Std Dev)	C (Mean)	C (Std Dev)
A11 1000m ato	pGEM	21716.65	1292.00	25.70	0.09
A11 1000m saki	pGEM	9589.81	162.90	26.96	0.03
A11 200m ato	pGEM	23840.97	1587.22	25.56	0.10
A11 200m saki	pGEM	9239.20	745.29	27.02	0.12
A11 3000m ato	pGEM	23111.91	322.90	25.60	0.02
A11 3000m saki	pGEM	10315.50	213.00	26.84	0.03
A11 SCM ato	pGEM	23118.92	781.05	25.60	0.05
A11 SCM saki	pGEM	8062.56	318.31	27.22	0.06
A21 1000m ato	pGEM	23258.54	320.71	25.59	0.02
A21 1000m saki	pGEM	7557.35	613.11	27.33	0.13
A21 10m ato	pGEM	22678.93	1392.33	25.63	0.10
A21 10m saki	pGEM	5794.77	1032.68	27.75	0.28
A21 200m ato	pGEM	21782.39	2231.67	25.70	0.16
A21 200m saki	pGEM	9261.75	650.74	27.01	0.11
A21 3000m ato	pGEM	22100.89	907.03	25.67	0.06
A21 3000m saki	pGEM	6336.58	56.93	27.59	0.01
A21 SCM ato	pGEM	23135.51	1572.45	25.60	0.10
A21 SCM saki	pGEM	7114.21	53.98	27.42	0.01
Comf2E4	pGEM	22458.60	411.46	25.65	0.03

Plate Layout

	1	2	3	4	5	6	7	8	9	10	11	12
A	S pGEM<br>2E6<br>FAM-NFQ-MGB<br>CT: 18.97	S pGEM<br>2E6<br>FAM-NFQ-MGB<br>CT: 18.67	A11 SCM saki<br>U pGEM<br>7.75E3<br>FAM-NFQ-MGB<br>CT: 27.28	A11 SCM saki<br>U pGEM<br>8.05E3<br>FAM-NFQ-MGB<br>CT: 27.22	A11 SCM saki<br>U pGEM<br>8.39E3<br>FAM-NFQ-MGB<br>CT: 27.16	A21 10m saki<br>U pGEM<br>5.97E3<br>FAM-NFQ-MGB<br>CT: 27.69	A21 10m saki<br>U pGEM<br>6.73E3<br>FAM-NFQ-MGB<br>CT: 27.5	A21 10m saki<br>U pGEM<br>4.69E3<br>FAM-NFQ-MGB<br>CT: 28.06	A21 3000m saki<br>U pGEM<br>6.32E3<br>FAM-NFQ-MGB<br>CT: 27.6	A21 3000m saki<br>U pGEM<br>6.4E3<br>FAM-NFQ-MGB<br>CT: 27.58	A21 3000m saki<br>U pGEM<br>6.29E3<br>FAM-NFQ-MGB<br>CT: 27.61	
B	S pGEM<br>2E5<br>FAM-NFQ-MGB<br>CT: 22.25	S pGEM<br>2E5<br>FAM-NFQ-MGB<br>CT: 22.15	A11 SCM ato<br>U pGEM<br>2.26E4<br>FAM-NFQ-MGB<br>CT: 25.63	A11 SCM ato<br>U pGEM<br>2.4E4<br>FAM-NFQ-MGB<br>CT: 25.54	A11 SCM ato<br>U pGEM<br>2.27E4<br>FAM-NFQ-MGB<br>CT: 25.63	A21 10m ato<br>U pGEM<br>2.36E4<br>FAM-NFQ-MGB<br>CT: 25.57	A21 10m ato<br>U pGEM<br>2.33E4<br>FAM-NFQ-MGB<br>CT: 25.59	A21 10m ato<br>U pGEM<br>2.11E4<br>FAM-NFQ-MGB<br>CT: 25.74	A21 3000m ato<br>U pGEM<br>2.31E4<br>FAM-NFQ-MGB<br>CT: 25.6	A21 3000m ato<br>U pGEM<br>2.14E4<br>FAM-NFQ-MGB<br>CT: 25.73	A21 3000m ato<br>U pGEM<br>2.18E4<br>FAM-NFQ-MGB<br>CT: 25.69	
C	S pGEM<br>2E4<br>FAM-NFQ-MGB<br>CT: 25.79	S pGEM<br>2E4<br>FAM-NFQ-MGB<br>CT: 25.6	A11 200m saki<br>U pGEM<br>8.94E3<br>FAM-NFQ-MGB<br>CT: 27.06	A11 200m saki<br>U pGEM<br>1.01E4<br>FAM-NFQ-MGB<br>CT: 26.88	A11 200m saki<br>U pGEM<br>8.69E3<br>FAM-NFQ-MGB<br>CT: 27.11	A21 SCM saki<br>U pGEM<br>7.09E3<br>FAM-NFQ-MGB<br>CT: 27.42	A21 SCM saki<br>U pGEM<br>7.08E3<br>FAM-NFQ-MGB<br>CT: 27.42	A21 SCM saki<br>U pGEM<br>7.18E3<br>FAM-NFQ-MGB<br>CT: 27.4				
D	S pGEM<br>2E3<br>FAM-NFQ-MGB<br>CT: 29.46	S pGEM<br>2E3<br>FAM-NFQ-MGB<br>CT: 29.53	A11 200m ato<br>U pGEM<br>2.5E4<br>FAM-NFQ-MGB<br>CT: 25.48	A11 200m ato<br>U pGEM<br>2.45E4<br>FAM-NFQ-MGB<br>CT: 25.52	A11 200m ato<br>U pGEM<br>2.2E4<br>FAM-NFQ-MGB<br>CT: 25.68	A21 SCM ato<br>U pGEM<br>2.2E4<br>FAM-NFQ-MGB<br>CT: 25.68	A21 SCM ato<br>U pGEM<br>2.49E4<br>FAM-NFQ-MGB<br>CT: 25.49	A21 SCM ato<br>U pGEM<br>2.25E4<br>FAM-NFQ-MGB<br>CT: 25.65				
E	S pGEM<br>200<br>FAM-NFQ-MGB<br>CT: 33.01	S pGEM<br>200<br>FAM-NFQ-MGB<br>CT: 32.97	A11 1000m saki<br>U pGEM<br>9.44E3<br>FAM-NFQ-MGB<br>CT: 26.98	A11 1000m saki<br>U pGEM<br>9.57E3<br>FAM-NFQ-MGB<br>CT: 26.96	A11 1000m saki<br>U pGEM<br>9.76E3<br>FAM-NFQ-MGB<br>CT: 26.93	A21 200m saki<br>U pGEM<br>1E4<br>FAM-NFQ-MGB<br>CT: 26.89	A21 200m saki<br>U pGEM<br>8.82E3<br>FAM-NFQ-MGB<br>CT: 27.08	A21 200m saki<br>U pGEM<br>8.96E3<br>FAM-NFQ-MGB<br>CT: 27.06				
F	1<br>S pGEM<br>20<br>FAM-NFQ-MGB<br>CT: 36.77	1<br>S pGEM<br>20<br>FAM-NFQ-MGB<br>CT: 35.98	A11 1000m ato<br>U pGEM<br>2.28E4<br>FAM-NFQ-MGB<br>CT: 25.63	A11 1000m ato<br>U pGEM<br>2.03E4<br>FAM-NFQ-MGB<br>CT: 25.81	A11 1000m ato<br>U pGEM<br>2.21E4<br>FAM-NFQ-MGB<br>CT: 25.67	A21 200m ato<br>U pGEM<br>1.92E4<br>FAM-NFQ-MGB<br>CT: 25.89	A21 200m ato<br>U pGEM<br>2.34E4<br>FAM-NFQ-MGB<br>CT: 25.59	A21 200m ato<br>U pGEM<br>2.28E4<br>FAM-NFQ-MGB<br>CT: 25.63				
G			A11 3000m saki<br>U pGEM<br>1.03E4<br>FAM-NFQ-MGB<br>CT: 26.85	A11 3000m saki<br>U pGEM<br>1.05E4<br>FAM-NFQ-MGB<br>CT: 26.81	A11 3000m saki<br>U pGEM<br>1.01E4<br>FAM-NFQ-MGB<br>CT: 26.87	A21 1000m saki<br>U pGEM<br>7.99E3<br>FAM-NFQ-MGB<br>CT: 27.24	A21 1000m saki<br>U pGEM<br>6.86E3<br>FAM-NFQ-MGB<br>CT: 27.47	A21 1000m saki<br>U pGEM<br>7.83E3<br>FAM-NFQ-MGB<br>CT: 27.27				N pGEM<br>FAM-NFQ-MGB<br>CT: Undetermined
H	Comf2E4<br>U pGEM<br>2.22E4<br>FAM-NFQ-MGB<br>CT: 25.67	Comf2E4<br>U pGEM<br>2.27E4<br>FAM-NFQ-MGB<br>CT: 25.63	A11 3000m ato<br>U pGEM<br>2.28E4<br>FAM-NFQ-MGB<br>CT: 25.63	A11 3000m ato<br>U pGEM<br>2.34E4<br>FAM-NFQ-MGB<br>CT: 25.58	A11 3000m ato<br>U pGEM<br>2.31E4<br>FAM-NFQ-MGB<br>CT: 25.6	A21 1000m ato<br>U pGEM<br>2.33E4<br>FAM-NFQ-MGB<br>CT: 25.59	A21 1000m ato<br>U pGEM<br>2.29E4<br>FAM-NFQ-MGB<br>CT: 25.62	A21 1000m ato<br>U pGEM<br>2.35E4<br>FAM-NFQ-MGB<br>CT: 25.58				N pGEM<br>FAM-NFQ-MGB<br>CT: Undetermined

Standard Curves

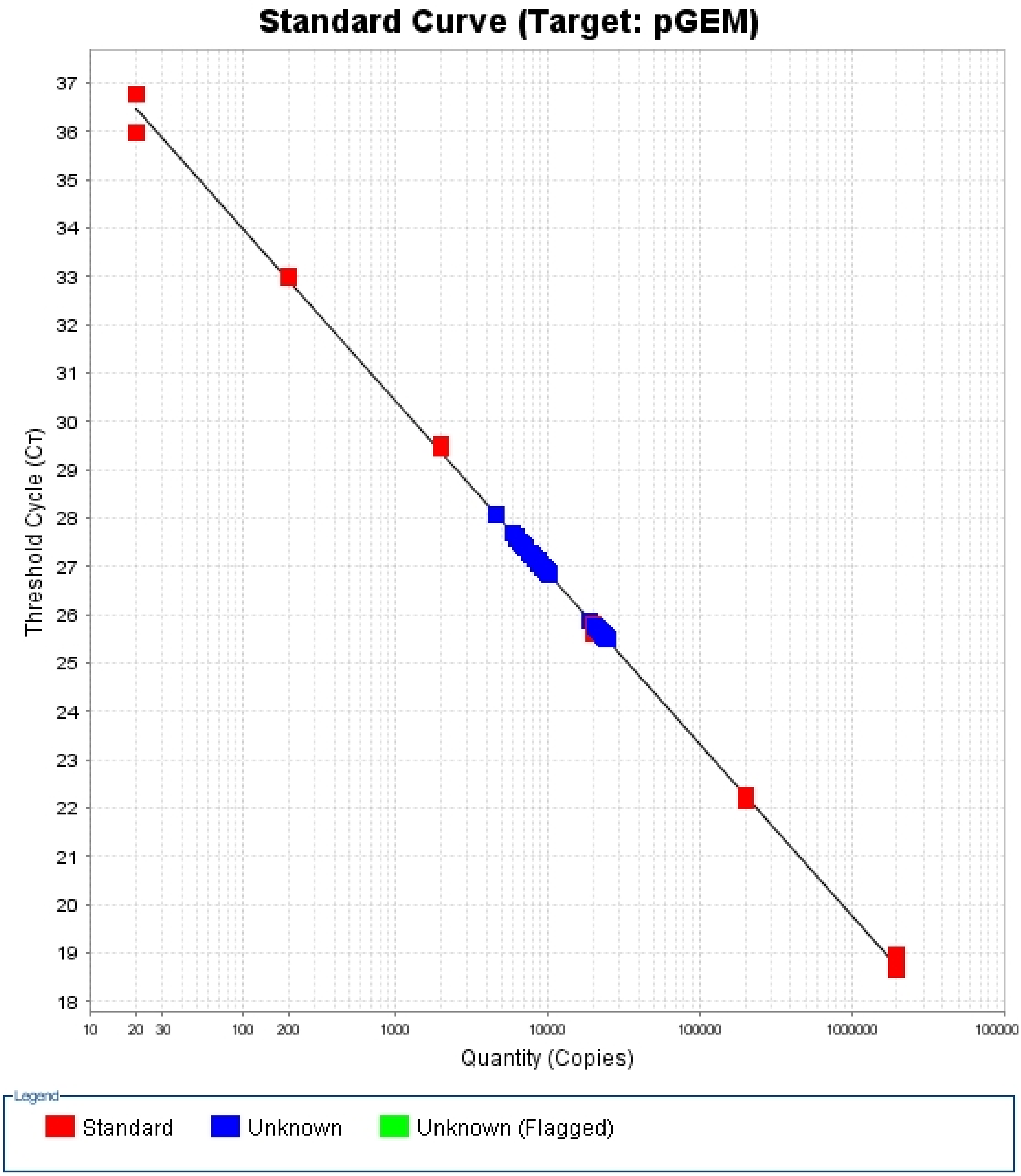

slope:-3.541 | Y-Intercept:41.056 | R^2:0.999 | Eff%:91.601

Results Table

Well		Sample	Target	Task	Quantity	C
A1			pGEM	STANDARD	2000000	18.9679
A2			pGEM	STANDARD	2000000	18.6742
A3		A11 SCM saki	pGEM	UNKNOWN	7749.1987	27.284
A4		A11 SCM saki	pGEM	UNKNOWN	8052.8789	27.2248
A5		A11 SCM saki	pGEM	UNKNOWN	8385.5918	27.1626
A6		A21 10m saki	pGEM	UNKNOWN	5969.2988	27.6853
A7		A21 10m saki	pGEM	UNKNOWN	6729.063	27.501
A8		A21 10m saki	pGEM	UNKNOWN	4685.9365	28.0575
A9		A21 3000m saki	pGEM	UNKNOWN	6324.8066	27.5963
A10		A21 3000m saki	pGEM	UNKNOWN	6398.4746	27.5785
A11		A21 3000m saki	pGEM	UNKNOWN	6286.4473	27.6057
B1			pGEM	STANDARD	200000	22.2541
B2			pGEM	STANDARD	200000	22.1501
B3		A11 SCM ato	pGEM	UNKNOWN	22646.8809	25.6347
B4		A11 SCM ato	pGEM	UNKNOWN	24020.4727	25.5441
B5		A11 SCM ato	pGEM	UNKNOWN	22689.418	25.6318
B6		A21 10m ato	pGEM	UNKNOWN	23637.875	25.5688
B7		A21 10m ato	pGEM	UNKNOWN	23317.002	25.5898
B8		A21 10m ato	pGEM	UNKNOWN	21081.9102	25.7448
B9		A21 3000m ato	pGEM	UNKNOWN	23109.2539	25.6036
B10		A21 3000m ato	pGEM	UNKNOWN	21351.5449	25.7253
B11		A21 3000m ato	pGEM	UNKNOWN	21841.8809	25.6903
C1			pGEM	STANDARD	20000	25.7895
C2			pGEM	STANDARD	20000	25.5991
C3		A11 200m saki	pGEM	UNKNOWN	8935.041	27.065
C4		A11 200m saki	pGEM	UNKNOWN	10088.4717	26.8783
C5		A11 200m saki	pGEM	UNKNOWN	8694.0996	27.107
C6		A21 SCM saki	pGEM	UNKNOWN	7088.2388	27.4211
C7		A21 SCM saki	pGEM	UNKNOWN	7078.1362	27.4233
C8		A21 SCM saki	pGEM	UNKNOWN	7176.2705	27.4021

Well		Sample	Target	Task	Quantity	C
D1			pGEM	STANDARD	2000	29.4587
D2			pGEM	STANDARD	2000	29.5284
D3		A11 200m ato	pGEM	UNKNOWN	25022.1797	25.4813
D4		A11 200m ato	pGEM	UNKNOWN	24463.9688	25.516
D5		A11 200m ato	pGEM	UNKNOWN	22036.7598	25.6767
D6		A21 SCM ato	pGEM	UNKNOWN	21986.418	25.6802
D7		A21 SCM ato	pGEM	UNKNOWN	24927.5527	25.4871
D8		A21 SCM ato	pGEM	UNKNOWN	22492.5605	25.6452
E1			pGEM	STANDARD	200	33.0098
E2			pGEM	STANDARD	200	32.9717
E3		A11 1000m saki	pGEM	UNKNOWN	9436.4053	26.981
E4		A11 1000m saki	pGEM	UNKNOWN	9572.2451	26.959
E5		A11 1000m saki	pGEM	UNKNOWN	9760.7754	26.929
E6		A21 200m saki	pGEM	UNKNOWN	10009.1572	26.8904
E7		A21 200m saki	pGEM	UNKNOWN	8820.959	27.0847
E8		A21 200m saki	pGEM	UNKNOWN	8955.1221	27.0615
F1			pGEM	STANDARD	20	36.7728
F2			pGEM	STANDARD	20	35.9792
F3		A11 1000m ato	pGEM	UNKNOWN	22751.3535	25.6276
F4		A11 1000m ato	pGEM	UNKNOWN	20268.5371	25.8053
F5		A11 1000m ato	pGEM	UNKNOWN	22130.0449	25.6702
F6		A21 200m ato	pGEM	UNKNOWN	19229.75	25.8862
F7		A21 200m ato	pGEM	UNKNOWN	23364.2734	25.5867
F8		A21 200m ato	pGEM	UNKNOWN	22753.1602	25.6275
G3		A11 3000m saki	pGEM	UNKNOWN	10271.7305	26.8506
G4		A11 3000m saki	pGEM	UNKNOWN	10546.9854	26.8099
G5		A11 3000m saki	pGEM	UNKNOWN	10127.7861	26.8723
G6		A21 1000m saki	pGEM	UNKNOWN	7989.8555	27.2369
G7		A21 1000m saki	pGEM	UNKNOWN	6855.7007	27.4724
G8		A21 1000m saki	pGEM	UNKNOWN	7826.5078	27.2687
G12			pGEM	NTC		Undetermined
H1		Comf2E4	pGEM	UNKNOWN	22167.6523	25.6676

Well		Sample	Target	Task	Quantity	C
H2		Comf2E4	pGEM	UNKNOWN	22749.5488	25.6277
H3		A11 3000m ato	pGEM	UNKNOWN	22777.0469	25.6259
H4		A11 3000m ato	pGEM	UNKNOWN	23421.3418	25.583
H5		A11 3000m ato	pGEM	UNKNOWN	23137.3301	25.6017
H6		A21 1000m ato	pGEM	UNKNOWN	23338.3535	25.5884
H7		A21 1000m ato	pGEM	UNKNOWN	22905.4609	25.6172
H8		A21 1000m ato	pGEM	UNKNOWN	23531.8105	25.5757
H12			pGEM	NTC		Undetermined

QC Summary

Total Wells	96	Processed Wells	70	Targets Used	1
Well Setup	70	Flagged Wells	2	Samples Used	19

Flag	Name	Frequency	Locations
AMPNC	Amplification in negative control	0	
BADROX	Bad passive reference signal	0	
BLFAIL	Baseline algorithm failed	0	
CTFAIL	C algorithm failed	0	
EXPFAIL	Exponential algorithm failed	0	
HIGHSD	High standard deviation in replicate group	2	F1, F2
NOAMP	No amplification	0	
NOISE	Noise higher than others in plate	0	
NOSIGNAL	No signal in well	0	
OFFSCALE	Fluorescence is offscale	0	
OUTLIERRG	Outlier in replicate group	0	
SPIKE	Noise spikes	0	
THOLDFAIL	Thresholding algorithm failed	0	
