# Supplementary material for: Ecological impact of algal-preying protists aplanochytrids (Labyrinthulea) as a source of docosahexaenoic acid for copepods in marine environments
Source: ISME Commun. 2026 Jun 26;6(1):ycag178. doi: 10.1093/ismeco/ycag178 (PMC13685785; doi:10.1093/ismeco/ycag178)
Supplement: Supplementary file 2 [file supplementary_materials_ycag178.zip › Morimoto_ISME_C_3rdrev_supplemental_data.zip!/Morimoto_ISME_C_3rdrev_supplemental_data/Supplemental_Data_2/Standard Curve/DNA recovery/2110/DnaRec2110AlineA11-A21N=1.pdf]

Experiment Results Report
230710DnaRec2210AlineA11-A21N=1

Experiment Summary

Experiment Name	:	230710DnaRec2210AlineA11-A21N=1
Experiment Type	:	Quantitation - Standard Curve
File Name	:	230710DnaRec2210AlineA11-A21N=1.eds
Run Started	:	2023 Jul 10 5:07:25 PM
Run Finished	:	2023 Jul 10 6:42:09 PM
Run Duration	:	94 minutes 43 seconds
Date Modified	:	2023 Jul 10 6:52:29 PM
User	:	
Number of wells used	:	70
Number of wells with results	:	69
Instrument Name	:	
Instrument Type	:	Applied Biosystems StepOnePlus™ Instrument
Comments	:	

Results Summary

Sample	Target	Quantity (Mean)	Quantity (Std Dev)	C (Mean)	C (Std Dev)
A11 1000m ato	pGEM	46495.91	1754.49	24.51	0.06
A11 1000m saki	pGEM	24785.51	2744.84	25.47	0.17
A11 200m ato	pGEM	46213.89	2915.48	24.52	0.09
A11 200m saki	pGEM	23869.91	1719.23	25.52	0.11
A11 3000m ato	pGEM	54116.07	1112.38	24.28	0.03
A11 3000m saki	pGEM	18351.26	948.44	25.92	0.08
A11 SCM ato	pGEM	24526.80	907.38	25.40	0.06
A11 SCM saki	pGEM			25.95	0.46
A21 1000m ato	pGEM	44393.25	969.68	24.58	0.03
A21 1000m saki	pGEM	8984.38	920.18	27.01	0.16
A21 10m ato	pGEM	39951.72	1410.92	24.74	0.05
A21 10m saki	pGEM			26.59	0.11
A21 200m ato	pGEM	60940.81	4543.61	24.10	0.11
A21 200m saki	pGEM	321.77	30.63	32.06	0.15
A21 3000m ato	pGEM	43495.00	486.23	24.61	0.02
A21 3000m saki	pGEM	10256.67	461.92	25.99	0.13
A21 SCM ato	pGEM	41208.84	902.17	24.69	0.03
A21 SCM saki	pGEM	10449.89	455.08	26.73	0.07
Comf2E4	pGEM	25290.85	2411.49	25.81	0.02

Plate Layout

	1	2	3	4	5	6	7	8	9	10	11	12
A	1<br>S pGEM<br>2E6<br>FAM-NFQ-MGB<br>CT: 19.03	1<br>S pGEM<br>2E6<br>FAM-NFQ-MGB<br>CT: 18.08	A11 SCM saki<br>U pGEM<br>FAM-NFQ-MGB<br>CT: 25.45	A11 SCM saki<br>U pGEM<br>FAM-NFQ-MGB<br>CT: 26.34	A11 SCM saki<br>U pGEM<br>FAM-NFQ-MGB<br>CT: 26.07	A21 10m saki<br>U pGEM<br>FAM-NFQ-MGB<br>CT: 26.7	A21 10m saki<br>U pGEM<br>FAM-NFQ-MGB<br>CT: 26.56	A21 10m saki<br>U pGEM<br>1.25E4<br>FAM-NFQ-MGB<br>CT: 26.5	A21 3000m saki<br>U pGEM<br>1.06E4<br>FAM-NFQ-MGB<br>CT: 25.84	A21 3000m saki<br>U pGEM<br>1.1E4<br>FAM-NFQ-MGB<br>CT: 26.65	A21 3000m saki<br>U pGEM<br>9.82E3<br>FAM-NFQ-MGB<br>CT: 26.83	
B	S pGEM<br>2E5<br>FAM-NFQ-MGB<br>CT: 22.42	S pGEM<br>2E5<br>FAM-NFQ-MGB<br>CT: 22.43	A11 SCM ato<br>U pGEM<br>2.51E4<br>FAM-NFQ-MGB<br>CT: 25.36	A11 SCM ato<br>U pGEM<br>4.14E4<br>FAM-NFQ-MGB<br>CT: 24.68	A11 SCM ato<br>U pGEM<br>4.07E4<br>FAM-NFQ-MGB<br>CT: 24.71	A21 10m ato<br>U pGEM<br>3.85E4<br>FAM-NFQ-MGB<br>CT: 24.8	A21 10m ato<br>U pGEM<br>4.13E4<br>FAM-NFQ-MGB<br>CT: 24.69	A21 10m ato<br>U pGEM<br>4.01E4<br>FAM-NFQ-MGB<br>CT: 24.73	A21 3000m ato<br>U pGEM<br>4.4E4<br>FAM-NFQ-MGB<br>CT: 24.59	A21 3000m ato<br>U pGEM<br>4.3E4<br>FAM-NFQ-MGB<br>CT: 24.63	A21 3000m ato<br>U pGEM<br>4.35E4<br>FAM-NFQ-MGB<br>CT: 24.61	
C	S pGEM<br>2E4<br>FAM-NFQ-MGB<br>CT: 25.82	S pGEM<br>2E4<br>FAM-NFQ-MGB<br>CT: 25.78	A11 200m saki<br>U pGEM<br>2.22E4<br>FAM-NFQ-MGB<br>CT: 25.63	A11 200m saki<br>U pGEM<br>2.56E4<br>FAM-NFQ-MGB<br>CT: 25.41	A11 200m saki<br>U pGEM<br>2.38E4<br>FAM-NFQ-MGB<br>CT: 25.52	A21 SCM saki<br>U pGEM<br>1.08E4<br>FAM-NFQ-MGB<br>CT: 26.68	A21 SCM saki<br>U pGEM<br>1.64E4<br>FAM-NFQ-MGB<br>CT: 26.09	A21 SCM saki<br>U pGEM<br>1.58E4<br>FAM-NFQ-MGB<br>CT: 26.15				
D	S pGEM<br>2E3<br>FAM-NFQ-MGB<br>CT: 29.46	S pGEM<br>2E3<br>FAM-NFQ-MGB<br>CT: 29.45	A11 200m ato<br>U pGEM<br>4.42E4<br>FAM-NFQ-MGB<br>CT: 24.58	A11 200m ato<br>U pGEM<br>4.96E4<br>FAM-NFQ-MGB<br>CT: 24.41	A11 200m ato<br>U pGEM<br>4.49E4<br>FAM-NFQ-MGB<br>CT: 24.56	A21 SCM ato<br>U pGEM<br>4.02E4<br>FAM-NFQ-MGB<br>CT: 24.73	A21 SCM ato<br>U pGEM<br>4.18E4<br>FAM-NFQ-MGB<br>CT: 24.67	A21 SCM ato<br>U pGEM<br>4.17E4<br>FAM-NFQ-MGB<br>CT: 24.67				
E	S pGEM<br>200<br>FAM-NFQ-MGB<br>CT: 33.25	S pGEM<br>200<br>FAM-NFQ-MGB<br>CT: 32.93	A11 1000m saki<br>U pGEM<br>2.69E4<br>FAM-NFQ-MGB<br>CT: 25.34	A11 1000m saki<br>U pGEM<br>2.17E4<br>FAM-NFQ-MGB<br>CT: 25.67	A11 1000m saki<br>U pGEM<br>2.57E4<br>FAM-NFQ-MGB<br>CT: 25.41	A21 200m saki<br>U pGEM<br>286.5<br>FAM-NFQ-MGB<br>CT: 32.24	A21 200m saki<br>U pGEM<br>337.12<br>FAM-NFQ-MGB<br>CT: 31.99	A21 200m saki<br>U pGEM<br>341.69<br>FAM-NFQ-MGB<br>CT: 31.97				
F	S pGEM<br>20<br>FAM-NFQ-MGB<br>CT: 35.97	S pGEM<br>20<br>FAM-NFQ-MGB<br>CT: 35.83	A11 1000m ato<br>U pGEM<br>4.45E4<br>FAM-NFQ-MGB<br>CT: 24.57	A11 1000m ato<br>U pGEM<br>4.79E4<br>FAM-NFQ-MGB<br>CT: 24.46	A11 1000m ato<br>U pGEM<br>4.7E4<br>FAM-NFQ-MGB<br>CT: 24.49	A21 200m ato<br>U pGEM<br>5.73E4<br>FAM-NFQ-MGB<br>CT: 24.19	A21 200m ato<br>U pGEM<br>6.6E4<br>FAM-NFQ-MGB<br>CT: 23.98	A21 200m ato<br>U pGEM<br>2.5E4<br>FAM-NFQ-MGB<br>CT: 25.36				
G			A11 3000m saki<br>U pGEM<br>1.76E4<br>FAM-NFQ-MGB<br>CT: 25.98	A11 3000m saki<br>U pGEM<br>1.94E4<br>FAM-NFQ-MGB<br>CT: 25.83	A11 3000m saki<br>U pGEM<br>1.8E4<br>FAM-NFQ-MGB<br>CT: 25.95	A21 1000m saki<br>U pGEM<br>9.04E3<br>FAM-NFQ-MGB<br>CT: 27	A21 1000m saki<br>U pGEM<br>8.04E3<br>FAM-NFQ-MGB<br>CT: 27.17	A21 1000m saki<br>U pGEM<br>9.88E3<br>FAM-NFQ-MGB<br>CT: 26.86				N pGEM<br>FAM-NFQ-MGB<br>CT: Undetermined
H	Comf2E4<br>U pGEM<br>2.78E4<br>FAM-NFQ-MGB<br>CT: 25.83	Comf2E4<br>U pGEM<br>FAM-NFQ-MGB<br>CT: Undetermined	A11 3000m ato<br>U pGEM<br>5.41E4<br>FAM-NFQ-MGB<br>CT: 24.28	A11 3000m ato<br>U pGEM<br>5.52E4<br>FAM-NFQ-MGB<br>CT: 24.25	A11 3000m ato<br>U pGEM<br>5.3E4<br>FAM-NFQ-MGB<br>CT: 24.31	A21 1000m ato<br>U pGEM<br>4.51E4<br>FAM-NFQ-MGB<br>CT: 24.55	A21 1000m ato<br>U pGEM<br>4.48E4<br>FAM-NFQ-MGB<br>CT: 24.56	A21 1000m ato<br>U pGEM<br>4.33E4<br>FAM-NFQ-MGB<br>CT: 24.62				N pGEM<br>FAM-NFQ-MGB<br>CT: Undetermined

Standard Curves

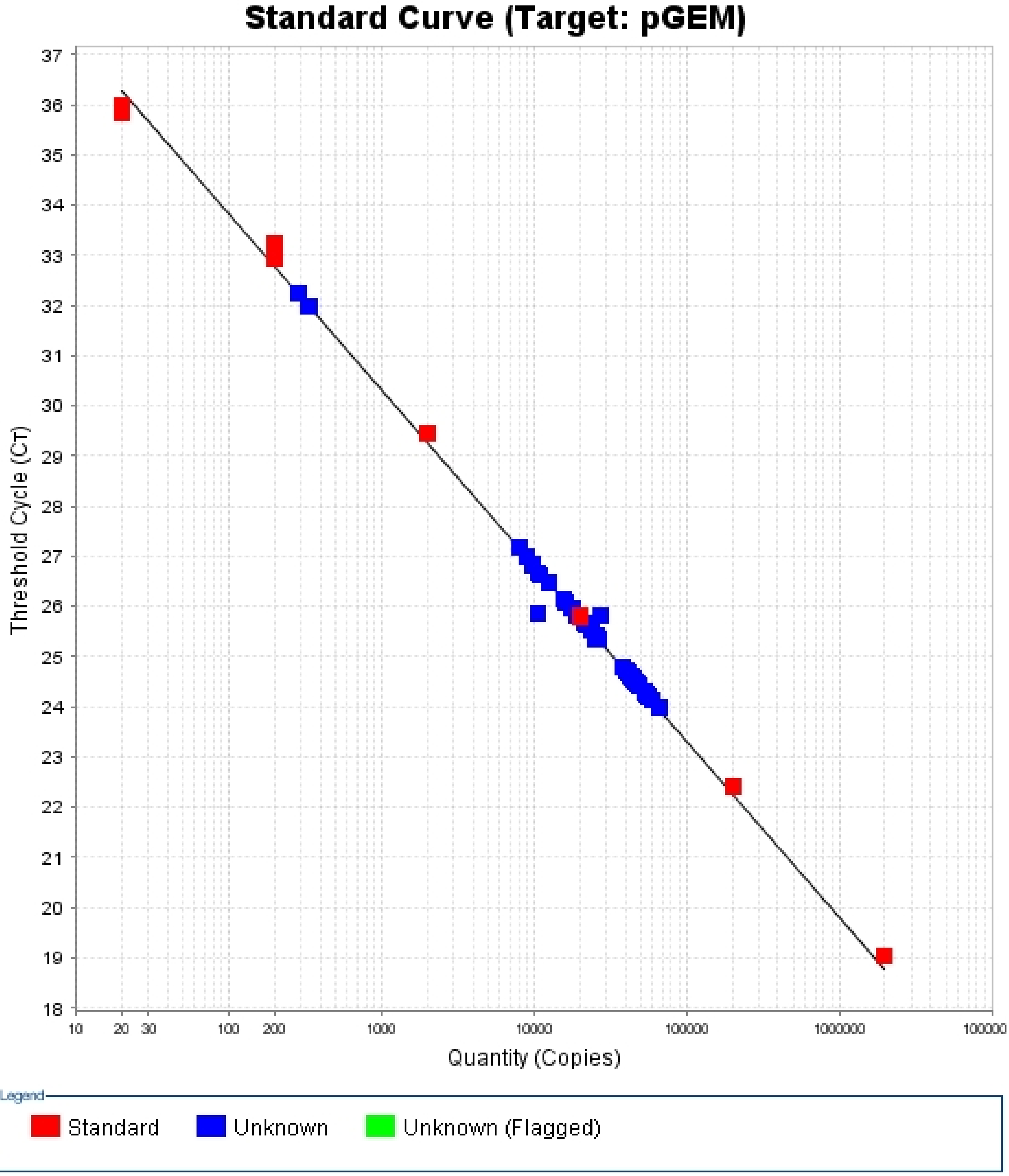

slope:-3.496 Y-Intercept:40.826 R^2:0.997 Eff%:93.212

Results Table

Well		Sample	Target	Task	Quantity	C
A1			pGEM	STANDARD	2000000	19.0327
A2			pGEM	STANDARD		18.0757
A3		A11 SCM saki	pGEM	UNKNOWN		25.4458
A4		A11 SCM saki	pGEM	UNKNOWN		26.3366
A5		A11 SCM saki	pGEM	UNKNOWN		26.0676
A6		A21 10m saki	pGEM	UNKNOWN		26.7046
A7		A21 10m saki	pGEM	UNKNOWN		26.5576
A8		A21 10m saki	pGEM	UNKNOWN	12534.2227	26.4984
A9		A21 3000m saki	pGEM	UNKNOWN	10619.3213	25.8424
A10		A21 3000m saki	pGEM	UNKNOWN	11018.3076	26.6459
A11		A21 3000m saki	pGEM	UNKNOWN	9822.2988	26.8256
B1			pGEM	STANDARD	200000	22.424
B2			pGEM	STANDARD	200000	22.4252
B3		A11 SCM ato	pGEM	UNKNOWN	25112.6719	25.3574
B4		A11 SCM ato	pGEM	UNKNOWN	41384.6602	24.6849
B5		A11 SCM ato	pGEM	UNKNOWN	40673.5117	24.7112
B6		A21 10m ato	pGEM	UNKNOWN	38486.6992	24.7951
B7		A21 10m ato	pGEM	UNKNOWN	41301.457	24.6879
B8		A21 10m ato	pGEM	UNKNOWN	40066.9922	24.734
B9		A21 3000m ato	pGEM	UNKNOWN	43955.625	24.5934
B10		A21 3000m ato	pGEM	UNKNOWN	42986.6875	24.6272
B11		A21 3000m ato	pGEM	UNKNOWN	43542.6758	24.6077
C1			pGEM	STANDARD	20000	25.8241
C2			pGEM	STANDARD	20000	25.7806
C3		A11 200m saki	pGEM	UNKNOWN	22173.4004	25.6323
C4		A11 200m saki	pGEM	UNKNOWN	25611.002	25.4135
C5		A11 200m saki	pGEM	UNKNOWN	23825.3418	25.5232
C6		A21 SCM saki	pGEM	UNKNOWN	10782.2002	26.6798
C7		A21 SCM saki	pGEM	UNKNOWN	16427.7129	26.0877
C8		A21 SCM saki	pGEM	UNKNOWN	15807.2217	26.1462

Well	Sample	Target	Task	Quantity	C
D1		pGEM	STANDARD	2000	29.46
D2		pGEM	STANDARD	2000	29.4487
D3	A11 200m ato	pGEM	UNKNOWN	44216.1953	24.5844
D4	A11 200m ato	pGEM	UNKNOWN	49559.4258	24.4112
D5	A11 200m ato	pGEM	UNKNOWN	44866.0781	24.5622
D6	A21 SCM ato	pGEM	UNKNOWN	40167.6289	24.7302
D7	A21 SCM ato	pGEM	UNKNOWN	41757.9922	24.6712
D8	A21 SCM ato	pGEM	UNKNOWN	41700.9062	24.6733
E1		pGEM	STANDARD	200	33.2454
E2		pGEM	STANDARD	200	32.9286
E3	A11 1000m saki	pGEM	UNKNOWN	26947.6484	25.3363
E4	A11 1000m saki	pGEM	UNKNOWN	21697.4531	25.6653
E5	A11 1000m saki	pGEM	UNKNOWN	25711.418	25.4076
E6	A21 200m saki	pGEM	UNKNOWN	286.5021	32.2354
E7	A21 200m saki	pGEM	UNKNOWN	337.1208	31.9884
E8	A21 200m saki	pGEM	UNKNOWN	341.6943	31.9679
F1		pGEM	STANDARD	20	35.9725
F2		pGEM	STANDARD	20	35.8259
F3	A11 1000m ato	pGEM	UNKNOWN	44546.2461	24.5731
F4	A11 1000m ato	pGEM	UNKNOWN	47947.5547	24.4614
F5	A11 1000m ato	pGEM	UNKNOWN	46993.9336	24.4919
F6	A21 200m ato	pGEM	UNKNOWN	57318.6719	24.1903
F7	A21 200m ato	pGEM	UNKNOWN	66038.8984	23.9753
F8	A21 200m ato	pGEM	UNKNOWN	25043.8301	25.3617
G3	A11 3000m saki	pGEM	UNKNOWN	17648.8008	25.9789
G4	A11 3000m saki	pGEM	UNKNOWN	19430.1191	25.8329
G5	A11 3000m saki	pGEM	UNKNOWN	17974.8457	25.9511
G6	A21 1000m saki	pGEM	UNKNOWN	9036.498	26.9952
G7	A21 1000m saki	pGEM	UNKNOWN	8039.2437	27.1728
G8	A21 1000m saki	pGEM	UNKNOWN	9877.3975	26.8601
G12		pGEM	NTC		Undetermined
H1	Comf2E4	pGEM	UNKNOWN	27816.334	25.8265

Well		Sample	Target	Task	Quantity	C
H2		Comf2E4	pGEM	UNKNOWN		
H3		A11 3000m ato	pGEM	UNKNOWN	54102.9414	24.278
H4		A11 3000m ato	pGEM	UNKNOWN	55234.9492	24.2465
H5		A11 3000m ato	pGEM	UNKNOWN	53010.3125	24.309
H6		A21 1000m ato	pGEM	UNKNOWN	45090.2773	24.5547
H7		A21 1000m ato	pGEM	UNKNOWN	44803.6172	24.5643
H8		A21 1000m ato	pGEM	UNKNOWN	43285.8594	24.6167
H12			pGEM	NTC		Undetermined

QC Summary

Total Wells	96	Processed Wells	70	Targets Used	1
Well Setup	70	Flagged Wells	2	Samples Used	19

Flag	Name	Frequency	Locations
AMPNC	Amplification in negative control	0	
BADROX	Bad passive reference signal	0	
BLFAIL	Baseline algorithm failed	0	
CTFAIL	C algorithm failed	0	
EXPFAIL	Exponential algorithm failed	0	
HIGHSD	High standard deviation in replicate group	2	A1, A2
NOAMP	No amplification	0	
NOISE	Noise higher than others in plate	0	
NOSIGNAL	No signal in well	0	
OFFSCALE	Fluorescence is offscale	0	
OUTLIERRG	Outlier in replicate group	0	
SPIKE	Noise spikes	0	
THOLDFAIL	Thresholding algorithm failed	0	
