# Supplementary material for: Ecological impact of algal-preying protists aplanochytrids (Labyrinthulea) as a source of docosahexaenoic acid for copepods in marine environments
Source: ISME Commun. 2026 Jun 26;6(1):ycag178. doi: 10.1093/ismeco/ycag178 (PMC13685785; doi:10.1093/ismeco/ycag178)
Supplement: Supplementary file 2 [file supplementary_materials_ycag178.zip › Morimoto_ISME_C_3rdrev_supplemental_data.zip!/Morimoto_ISME_C_3rdrev_supplemental_data/Supplemental_Data_2/Standard Curve/DNA recovery/2110/DnaRec2110A15N=1,2,3.pdf]

Experiment Results Report

230721DnaRec2110A15N=1,2,3

Experiment Summary

Experiment Name	: 230721DnaRec2110A15N=1,2,3
Experiment Type	: Quantitation - Standard Curve
File Name	: 230721DnaRec2110A15N=1,2,3.eds
Run Started	: 2023 Jul 21 2:42:38 PM
Run Finished	: 2023 Jul 21 4:17:13 PM
Run Duration	: 94 minutes 34 seconds
Date Modified	: 2023 Jul 21 4:46:39 PM
User	:
Number of wells used	: 86
Number of wells with results	: 86
Instrument Name	:
Instrument Type	: Applied Biosystems StepOnePlus™ Instrument
Comments	:

Results Summary

Sample	Target	Quantity (Mean)	Quantity (Std Dev)	C (Mean)	C (Std Dev)
A15 1000m ato1	pGEM	34741.58	2034.43	23.59	0.10
A15 1000m ato2	pGEM	24151.05	350.60	24.18	0.02
A15 1000m ato3	pGEM	26283.27	424.74	24.05	0.03
A15 1000m saki1	pGEM	23155.08	627.21	24.25	0.04
A15 1000m saki2	pGEM	9633.17	306.01	25.69	0.05
A15 1000m saki3	pGEM	8977.21	1137.52	25.81	0.21
A15 200m ato1	pGEM	32666.67	1373.72	23.69	0.07
A15 200m ato2	pGEM	26646.55	2075.16	24.03	0.12
A15 200m ato3	pGEM	27642.40	719.73	23.96	0.04
A15 200m saki1	pGEM	24398.48	1145.85	24.17	0.08
A15 200m saki2	pGEM	11332.43	415.09	25.42	0.06
A15 200m saki3	pGEM	8446.30	842.47	25.91	0.17
A15 3000m ato1	pGEM	34648.66	1674.98	23.60	0.08
A15 3000m ato2	pGEM	25961.12	763.82	24.07	0.05
A15 3000m ato3	pGEM	26288.37	688.23	24.05	0.04
A15 3000m saki1	pGEM	19286.34	941.11	24.55	0.08
A15 3000m saki2	pGEM	8452.42	750.62	25.91	0.15
A15 3000m saki3	pGEM	6680.38	702.44	26.29	0.17
A15 SCM ato1	pGEM	34801.18	678.56	23.59	0.03
A15 SCM ato2	pGEM	26788.33	294.83	24.02	0.02
A15 SCM ato3	pGEM	31931.42	821.00	23.73	0.04
A15 SCM saki1	pGEM	16650.35	740.75	24.79	0.07
A15 SCM saki2	pGEM	9894.40	246.18	25.64	0.04
A15 SCM saki3	pGEM	9319.52	610.95	25.74	0.11

Plate Layout

	1	2	3	4	5	6	7	8	9	10	11	12
A	S pGEM<br>2E6<br>FAM-NFQ-MGB<br>CT: 17.39	S pGEM<br>2E6<br>FAM-NFQ-MGB<br>CT: 17.27	A15 SCM saki1<br>U pGEM<br>1.69E4<br>FAM-NFQ-MGB<br>CT: 24.77	A15 SCM saki1<br>U pGEM<br>1.72E4<br>FAM-NFQ-MGB<br>CT: 24.74	A15 SCM saki1<br>U pGEM<br>1.58E4<br>FAM-NFQ-MGB<br>CT: 24.88	A15 SCM saki2<br>U pGEM<br>9.88E3<br>FAM-NFQ-MGB<br>CT: 25.65	A15 SCM saki2<br>U pGEM<br>9.66E3<br>FAM-NFQ-MGB<br>CT: 25.68	A15 SCM saki2<br>U pGEM<br>1.01E4<br>FAM-NFQ-MGB<br>CT: 25.6	A15 SCM saki3<br>U pGEM<br>9.43E3<br>FAM-NFQ-MGB<br>CT: 25.72	A15 SCM saki3<br>U pGEM<br>9.87E3<br>FAM-NFQ-MGB<br>CT: 25.65	A15 SCM saki3<br>U pGEM<br>8.66E3<br>FAM-NFQ-MGB<br>CT: 25.86	
B	S pGEM<br>2E5<br>FAM-NFQ-MGB<br>CT: 20.76	S pGEM<br>2E5<br>FAM-NFQ-MGB<br>CT: 20.77	A15 SCM ato1<br>U pGEM<br>3.53E4<br>FAM-NFQ-MGB<br>CT: 23.56	A15 SCM ato1<br>U pGEM<br>3.4E4<br>FAM-NFQ-MGB<br>CT: 23.62	A15 SCM ato1<br>U pGEM<br>3.51E4<br>FAM-NFQ-MGB<br>CT: 23.58	A15 SCM ato2<br>U pGEM<br>2.71E4<br>FAM-NFQ-MGB<br>CT: 24	A15 SCM ato2<br>U pGEM<br>2.68E4<br>FAM-NFQ-MGB<br>CT: 24.02	A15 SCM ato2<br>U pGEM<br>2.65E4<br>FAM-NFQ-MGB<br>CT: 24.03	A15 SCM ato3<br>U pGEM<br>3.23E4<br>FAM-NFQ-MGB<br>CT: 23.71	A15 SCM ato3<br>U pGEM<br>3.25E4<br>FAM-NFQ-MGB<br>CT: 23.7	A15 SCM ato3<br>U pGEM<br>3.1E4<br>FAM-NFQ-MGB<br>CT: 23.78	
C	S pGEM<br>2E4<br>FAM-NFQ-MGB<br>CT: 24.17	S pGEM<br>2E4<br>FAM-NFQ-MGB<br>CT: 24.14	A15 200m saki1<br>U pGEM<br>2.51E4<br>FAM-NFQ-MGB<br>CT: 24.12	A15 200m saki1<br>U pGEM<br>2.5E4<br>FAM-NFQ-MGB<br>CT: 24.13	A15 200m saki1<br>U pGEM<br>2.31E4<br>FAM-NFQ-MGB<br>CT: 24.26	A15 200m saki2<br>U pGEM<br>1.17E4<br>FAM-NFQ-MGB<br>CT: 25.36	A15 200m saki2<br>U pGEM<br>1.14E4<br>FAM-NFQ-MGB<br>CT: 25.42	A15 200m saki2<br>U pGEM<br>1.09E4<br>FAM-NFQ-MGB<br>CT: 25.48	A15 200m saki3<br>U pGEM<br>7.55E3<br>FAM-NFQ-MGB<br>CT: 26.09	A15 200m saki3<br>U pGEM<br>9.22E3<br>FAM-NFQ-MGB<br>CT: 25.76	A15 200m saki3<br>U pGEM<br>8.58E3<br>FAM-NFQ-MGB<br>CT: 25.88	
D	S pGEM<br>2E3<br>FAM-NFQ-MGB<br>CT: 27.9	S pGEM<br>2E3<br>FAM-NFQ-MGB<br>CT: 28.14	A15 200m ato1<br>U pGEM<br>3.12E4<br>FAM-NFQ-MGB<br>CT: 23.77	A15 200m ato1<br>U pGEM<br>3.38E4<br>FAM-NFQ-MGB<br>CT: 23.63	A15 200m ato1<br>U pGEM<br>3.3E4<br>FAM-NFQ-MGB<br>CT: 23.67	A15 200m ato2<br>U pGEM<br>2.54E4<br>FAM-NFQ-MGB<br>CT: 24.1	A15 200m ato2<br>U pGEM<br>2.9E4<br>FAM-NFQ-MGB<br>CT: 23.88	A15 200m ato2<br>U pGEM<br>2.55E4<br>FAM-NFQ-MGB<br>CT: 24.1	A15 200m ato3<br>U pGEM<br>2.81E4<br>FAM-NFQ-MGB<br>CT: 23.93	A15 200m ato3<br>U pGEM<br>2.8E4<br>FAM-NFQ-MGB<br>CT: 23.94	A15 200m ato3<br>U pGEM<br>2.68E4<br>FAM-NFQ-MGB<br>CT: 24.01	
E	S pGEM<br>200<br>FAM-NFQ-MGB<br>CT: 31.35	S pGEM<br>200<br>FAM-NFQ-MGB<br>CT: 31.66	A15 1000m saki1<br>U pGEM<br>2.25E4<br>FAM-NFQ-MGB<br>CT: 24.3	A15 1000m saki1<br>U pGEM<br>2.38E4<br>FAM-NFQ-MGB<br>CT: 24.21	A15 1000m saki1<br>U pGEM<br>2.32E4<br>FAM-NFQ-MGB<br>CT: 24.25	A15 1000m saki2<br>U pGEM<br>9.29E3<br>FAM-NFQ-MGB<br>CT: 25.75	A15 1000m saki2<br>U pGEM<br>9.86E3<br>FAM-NFQ-MGB<br>CT: 25.65	A15 1000m saki2<br>U pGEM<br>9.75E3<br>FAM-NFQ-MGB<br>CT: 25.67	A15 1000m saki3<br>U pGEM<br>9.83E3<br>FAM-NFQ-MGB<br>CT: 25.65	A15 1000m saki3<br>U pGEM<br>9.42E3<br>FAM-NFQ-MGB<br>CT: 25.72	A15 1000m saki3<br>U pGEM<br>7.69E3<br>FAM-NFQ-MGB<br>CT: 26.06	
F	1<br>S pGEM<br>20<br>FAM-NFQ-MGB<br>CT: 36.97	1<br>S pGEM<br>20<br>FAM-NFQ-MGB<br>CT: 35.97	A15 1000m ato1<br>U pGEM<br>3.51E4<br>FAM-NFQ-MGB<br>CT: 23.57	A15 1000m ato1<br>U pGEM<br>3.26E4<br>FAM-NFQ-MGB<br>CT: 23.7	A15 1000m ato1<br>U pGEM<br>3.66E4<br>FAM-NFQ-MGB<br>CT: 23.51	A15 1000m ato2<br>U pGEM<br>2.45E4<br>FAM-NFQ-MGB<br>CT: 24.16	A15 1000m ato2<br>U pGEM<br>2.39E4<br>FAM-NFQ-MGB<br>CT: 24.2	A15 1000m ato2<br>U pGEM<br>2.4E4<br>FAM-NFQ-MGB<br>CT: 24.19	A15 1000m ato3<br>U pGEM<br>2.6E4<br>FAM-NFQ-MGB<br>CT: 24.07	A15 1000m ato3<br>U pGEM<br>2.68E4<br>FAM-NFQ-MGB<br>CT: 24.02	A15 1000m ato3<br>U pGEM<br>2.61E4<br>FAM-NFQ-MGB<br>CT: 24.06	
G			A15 3000m saki1<br>U pGEM<br>1.85E4<br>FAM-NFQ-MGB<br>CT: 24.62	A15 3000m saki1<br>U pGEM<br>2.03E4<br>FAM-NFQ-MGB<br>CT: 24.47	A15 3000m saki1<br>U pGEM<br>1.9E4<br>FAM-NFQ-MGB<br>CT: 24.57	A15 3000m saki2<br>U pGEM<br>9.21E3<br>FAM-NFQ-MGB<br>CT: 25.76	A15 3000m saki2<br>U pGEM<br>7.71E3<br>FAM-NFQ-MGB<br>CT: 26.05	A15 3000m saki2<br>U pGEM<br>8.44E3<br>FAM-NFQ-MGB<br>CT: 25.9	A15 3000m saki3<br>U pGEM<br>6.4E3<br>FAM-NFQ-MGB<br>CT: 26.36	A15 3000m saki3<br>U pGEM<br>7.48E3<br>FAM-NFQ-MGB<br>CT: 26.1	A15 3000m saki3<br>U pGEM<br>6.16E3<br>FAM-NFQ-MGB<br>CT: 26.42	
H	N pGEM<br>FAM-NFQ-MGB<br>CT: Undetermined	N pGEM<br>FAM-NFQ-MGB<br>CT: Undetermined	A15 3000m ato1<br>U pGEM<br>3.31E4<br>FAM-NFQ-MGB<br>CT: 23.67	A15 3000m ato1<br>U pGEM<br>3.44E4<br>FAM-NFQ-MGB<br>CT: 23.61	A15 3000m ato1<br>U pGEM<br>3.64E4<br>FAM-NFQ-MGB<br>CT: 23.51	A15 3000m ato2<br>U pGEM<br>2.56E4<br>FAM-NFQ-MGB<br>CT: 24.09	A15 3000m ato2<br>U pGEM<br>2.54E4<br>FAM-NFQ-MGB<br>CT: 24.1	A15 3000m ato2<br>U pGEM<br>2.68E4<br>FAM-NFQ-MGB<br>CT: 24.01	A15 3000m ato3<br>U pGEM<br>2.57E4<br>FAM-NFQ-MGB<br>CT: 24.08	A15 3000m ato3<br>U pGEM<br>2.62E4<br>FAM-NFQ-MGB<br>CT: 24.05	A15 3000m ato3<br>U pGEM<br>2.7E4<br>FAM-NFQ-MGB<br>CT: 24	

Standard Curves

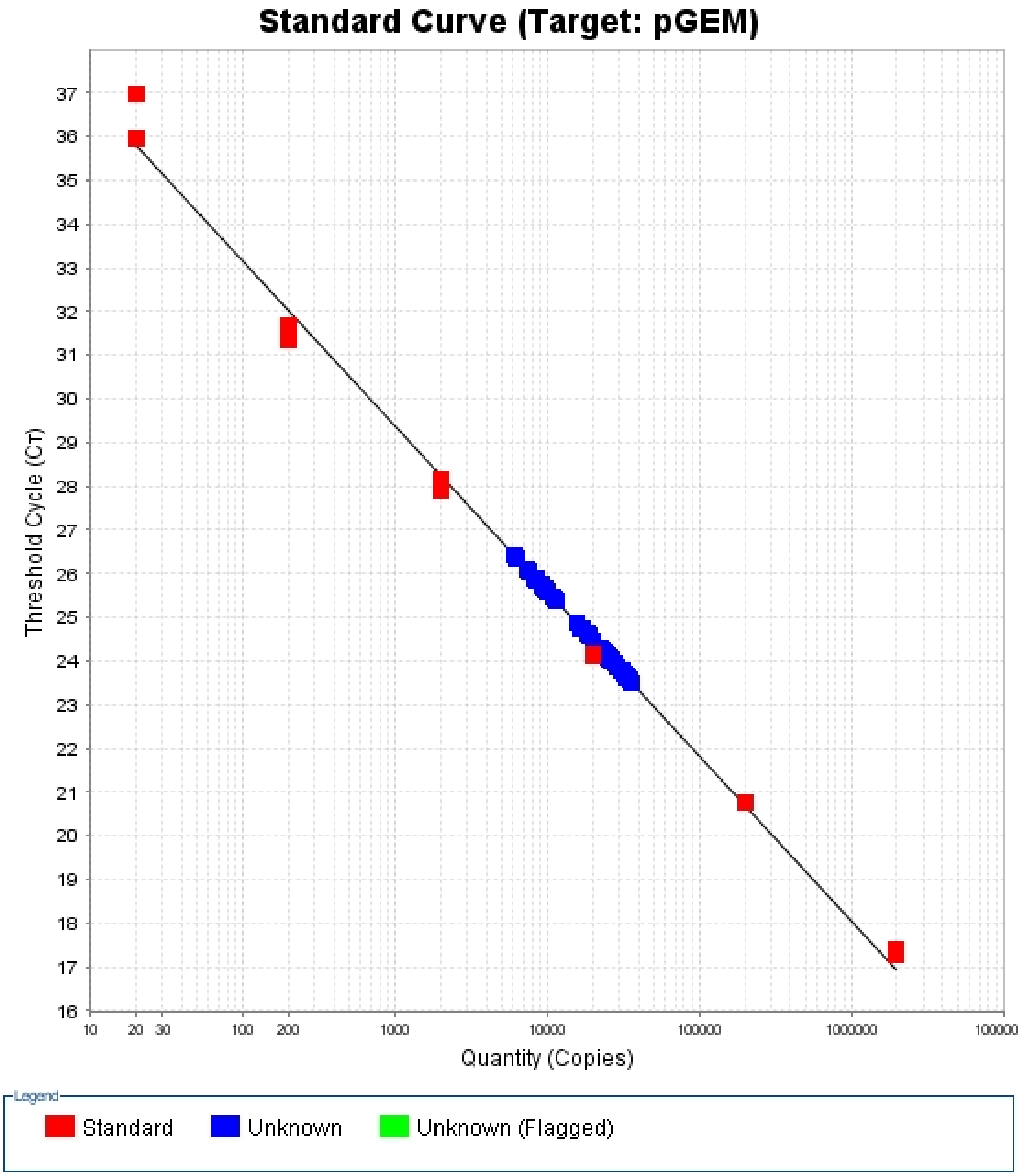

slope:-3.765 Y-Intercept:40.688 R^2:0.995 Eff%:84.325

Results Table

Well		Sample	Target	Task	Quantity	C
A1			pGEM	STANDARD	2000000	17.3929
A2			pGEM	STANDARD	2000000	17.269
A3		A15 SCM saki1	pGEM	UNKNOWN	16903.4316	24.7679
A4		A15 SCM saki1	pGEM	UNKNOWN	17231.3945	24.7365
A5		A15 SCM saki1	pGEM	UNKNOWN	15816.2314	24.8766
A6		A15 SCM saki2	pGEM	UNKNOWN	9881.0244	25.6459
A7		A15 SCM saki2	pGEM	UNKNOWN	9655.1699	25.6837
A8		A15 SCM saki2	pGEM	UNKNOWN	10146.9932	25.6024
A9		A15 SCM saki3	pGEM	UNKNOWN	9431.541	25.722
A10		A15 SCM saki3	pGEM	UNKNOWN	9866.709	25.6482
A11		A15 SCM saki3	pGEM	UNKNOWN	8660.3096	25.8615
B1			pGEM	STANDARD	200000	20.7567
B2			pGEM	STANDARD	200000	20.7726
B3		A15 SCM ato1	pGEM	UNKNOWN	35316.8711	23.563
B4		A15 SCM ato1	pGEM	UNKNOWN	34032.4609	23.6236
B5		A15 SCM ato1	pGEM	UNKNOWN	35054.2148	23.5752
B6		A15 SCM ato2	pGEM	UNKNOWN	27084.3965	23.997
B7		A15 SCM ato2	pGEM	UNKNOWN	26785.8438	24.0151
B8		A15 SCM ato2	pGEM	UNKNOWN	26494.752	24.033
B9		A15 SCM ato3	pGEM	UNKNOWN	32304.2969	23.7088
B10		A15 SCM ato3	pGEM	UNKNOWN	32499.8027	23.6989
B11		A15 SCM ato3	pGEM	UNKNOWN	30990.1543	23.7767
C1			pGEM	STANDARD	20000	24.1705
C2			pGEM	STANDARD	20000	24.1435
C3		A15 200m saki1	pGEM	UNKNOWN	25114.6152	24.1205
C4		A15 200m saki1	pGEM	UNKNOWN	25003.9238	24.1277
C5		A15 200m saki1	pGEM	UNKNOWN	23076.9102	24.2588
C6		A15 200m saki2	pGEM	UNKNOWN	11738.2295	25.3642
C7		A15 200m saki2	pGEM	UNKNOWN	11350.4121	25.4192
C8		A15 200m saki2	pGEM	UNKNOWN	10908.6348	25.4841

Well	Sample	Target	Task	Quantity	C
C9	A15 200m saki3	pGEM	UNKNOWN	7546.4932	26.0866
C10	A15 200m saki3	pGEM	UNKNOWN	9216.3906	25.7597
C11	A15 200m saki3	pGEM	UNKNOWN	8576.002	25.8775
D1		pGEM	STANDARD	2000	27.9028
D2		pGEM	STANDARD	2000	28.1411
D3	A15 200m ato1	pGEM	UNKNOWN	31157.8574	23.7679
D4	A15 200m ato1	pGEM	UNKNOWN	33845.0234	23.6326
D5	A15 200m ato1	pGEM	UNKNOWN	32997.1289	23.6741
D6	A15 200m ato2	pGEM	UNKNOWN	25399.9609	24.102
D7	A15 200m ato2	pGEM	UNKNOWN	29042.0742	23.8829
D8	A15 200m ato2	pGEM	UNKNOWN	25497.6191	24.0957
D9	A15 200m ato3	pGEM	UNKNOWN	28143.5508	23.9343
D10	A15 200m ato3	pGEM	UNKNOWN	27965.9629	23.9446
D11	A15 200m ato3	pGEM	UNKNOWN	26817.668	24.0132
E1		pGEM	STANDARD	200	31.3517
E2		pGEM	STANDARD	200	31.6624
E3	A15 1000m	pGEM	UNKNOWN	22520.4922	24.2987
E4	A15 1000m	pGEM	UNKNOWN	23774.6523	24.2101
E5	A15 1000m	pGEM	UNKNOWN	23170.0938	24.2522
E6	A15 1000m	pGEM	UNKNOWN	9285.0908	25.7476
E7	A15 1000m	pGEM	UNKNOWN	9859.8525	25.6494
E8	A15 1000m	pGEM	UNKNOWN	9754.5566	25.6669
E9	A15 1000m	pGEM	UNKNOWN	9827.4854	25.6548
E10	A15 1000m	pGEM	UNKNOWN	9419.0957	25.7242
E11	A15 1000m	pGEM	UNKNOWN	7685.0464	26.0569
F1		pGEM	STANDARD	20	36.9735
F2		pGEM	STANDARD	20	35.9668
F3	A15 1000m ato1	pGEM	UNKNOWN	35097.6641	23.5732
F4	A15 1000m ato1	pGEM	UNKNOWN	32552.6133	23.6963
F5	A15 1000m ato1	pGEM	UNKNOWN	36574.4648	23.5058
F6	A15 1000m ato2	pGEM	UNKNOWN	24542.6211	24.1581
F7	A15 1000m ato2	pGEM	UNKNOWN	23866.2285	24.2038

Well		Sample	Target	Task	Quantity	C
F8		A15 1000m ato2	pGEM	UNKNOWN	24044.2969	24.1917
F9		A15 1000m ato3	pGEM	UNKNOWN	25971.2363	24.0656
F10		A15 1000m ato3	pGEM	UNKNOWN	26766.9785	24.0163
F11		A15 1000m ato3	pGEM	UNKNOWN	26111.5977	24.0568
G3		A15 3000m	pGEM	UNKNOWN	18505.707	24.6198
G4		A15 3000m	pGEM	UNKNOWN	20331.377	24.466
G5		A15 3000m	pGEM	UNKNOWN	19021.9453	24.5748
G6		A15 3000m	pGEM	UNKNOWN	9211.3721	25.7606
G7		A15 3000m	pGEM	UNKNOWN	7710.4111	26.0515
G8		A15 3000m	pGEM	UNKNOWN	8435.4834	25.9045
G9		A15 3000m	pGEM	UNKNOWN	6401.5752	26.3557
G10		A15 3000m	pGEM	UNKNOWN	7479.4209	26.1012
G11		A15 3000m	pGEM	UNKNOWN	6160.1338	26.4186
H1			pGEM	NTC		Undetermined
H2			pGEM	NTC		Undetermined
H3		A15 3000m ato1	pGEM	UNKNOWN	33133.3867	23.6673
H4		A15 3000m ato1	pGEM	UNKNOWN	34365.3789	23.6076
H5		A15 3000m ato1	pGEM	UNKNOWN	36447.2188	23.5115
H6		A15 3000m ato2	pGEM	UNKNOWN	25601.8926	24.089
H7		A15 3000m ato2	pGEM	UNKNOWN	25443.1328	24.0992
H8		A15 3000m ato2	pGEM	UNKNOWN	26838.3203	24.0119
H9		A15 3000m ato3	pGEM	UNKNOWN	25670.0996	24.0847
H10		A15 3000m ato3	pGEM	UNKNOWN	26165.1035	24.0534
H11		A15 3000m ato3	pGEM	UNKNOWN	27029.8926	24.0003

QC Summary

Total Wells	96	Processed Wells	86	Targets Used	1
Well Setup	86	Flagged Wells	2	Samples Used	24

Flag	Name	Frequency	Locations
AMPNC	Amplification in negative control	0	
BADROX	Bad passive reference signal	0	
BLFAIL	Baseline algorithm failed	0	
CTFAIL	C algorithm failed	0	
EXPFAIL	Exponential algorithm failed	0	
HIGHSD	High standard deviation in replicate group	2	F1, F2
NOAMP	No amplification	0	
NOISE	Noise higher than others in plate	0	
NOSIGNAL	No signal in well	0	
OFFSCALE	Fluorescence is offscale	0	
OUTLIERRG	Outlier in replicate group	0	
SPIKE	Noise spikes	0	
THOLDFAIL	Thresholding algorithm failed	0	
