# Supplementary material for: Ecological impact of algal-preying protists aplanochytrids (Labyrinthulea) as a source of docosahexaenoic acid for copepods in marine environments
Source: ISME Commun. 2026 Jun 26;6(1):ycag178. doi: 10.1093/ismeco/ycag178 (PMC13685785; doi:10.1093/ismeco/ycag178)
Supplement: Supplementary file 2 [file supplementary_materials_ycag178.zip › Morimoto_ISME_C_3rdrev_supplemental_data.zip!/Morimoto_ISME_C_3rdrev_supplemental_data/Supplemental_Data_2/Standard Curve/DNA recovery/2110/DNaRec2110AlineA01-A07N=2.pdf]

Experiment Results Report

230711DnaRec2110AlineA01-A07N=2

Experiment Summary

Experiment Name	:	230711DnaRec2110AlineA01-A07N=2
Experiment Type	:	Quantitation - Standard Curve
File Name	:	230711DnaRec2110AlineA01-A07N=2.eds
Run Started	:	2023 Jul 11 2:51:04 PM
Run Finished	:	2023 Jul 11 4:30:23 PM
Run Duration	:	99 minutes 18 seconds
Date Modified	:	2023 Jul 11 4:31:41 PM
User	:	
Number of wells used	:	76
Number of wells with results	:	73
Instrument Name	:	
Instrument Type	:	Applied Biosystems StepOnePlus™ Instrument
Comments	:	

Results Summary

Sample	Target	Quantity (Mean)	Quantity (Std Dev)	C (Mean)	C (Std Dev)
A01 10m ato2	pGEM	24526.80	907.38	25.40	0.06
A01 10m saki2	pGEM	10229.00	546.88	26.76	0.08
A01 BTM ato2	pGEM	23425.06	1609.24	25.47	0.11
A01 BTM saki2	pGEM			26.46	0.04
A04 1000m ato2	pGEM	24324.29	1023.37	25.41	0.07
A04 1000m saki2	pGEM	10256.67	461.92	26.76	0.07
A04 200m ato2	pGEM	27117.62	961.01	25.24	0.06
A04 200m saki2	pGEM	10261.76	287.61	26.76	0.04
A04 3000m ato2	pGEM	23813.35	334.57	25.44	0.02
A04 3000m saki2	pGEM	10449.89	455.08	26.73	0.07
A04 SCM ato2	pGEM	26339.40	1095.32	25.28	0.06
A04 SCM saki2	pGEM	8294.39	374.54	27.09	0.07
A07 1000m ato2	pGEM	24049.88	743.16	25.43	0.05
A07 1000m saki2	pGEM				
A07 200m ato2	pGEM	23885.16	884.76	25.44	0.06
A07 200m saki2	pGEM	12804.44	527.70	26.41	0.06
A07 3000m ato2	pGEM	26112.12	954.51	25.30	0.06
A07 3000m saki2	pGEM	11618.50	1054.91	26.57	0.14
A07 SCM ato2	pGEM				
A07 SCM saki2	pGEM	9261.09	464.78	26.92	0.08
Comf2E4	pGEM				

Plate Layout

	1	2	3	4	5	6	7	8	9	10	11	12
A	1<br>S pGEM<br>2E6<br>FAM-NFQ-MGB<br>CT: 17.91	1<br>S pGEM<br>2E6<br>FAM-NFQ-MGB<br>CT: 18.84	A01 10m saki2<br>U pGEM<br>1.04E4<br>FAM-NFQ-MGB<br>CT: 26.74	A01 10m saki2<br>U pGEM<br>9.62E3<br>FAM-NFQ-MGB<br>CT: 26.86	A01 10m saki2<br>U pGEM<br>1.07E4<br>FAM-NFQ-MGB<br>CT: 26.69	A04 1000m saki2<br>U pGEM<br>1.04E4<br>FAM-NFQ-MGB<br>CT: 26.73	A04 1000m saki2<br>U pGEM<br>9.74E3<br>FAM-NFQ-MGB<br>CT: 26.84	A04 1000m saki2<br>U pGEM<br>1.06E4<br>FAM-NFQ-MGB<br>CT: 26.7	A07 1000m saki2<br>U pGEM<br>FAM-NFQ-MGB<br>CT: Undetermined	A07 1000m saki2<br>U pGEM<br>1.1E4<br>FAM-NFQ-MGB<br>CT: 26.65	A07 1000m saki2<br>U pGEM<br>9.85E3<br>FAM-NFQ-MGB<br>CT: 26.82	
B	S pGEM<br>2E5<br>FAM-NFQ-MGB<br>CT: 22.28	S pGEM<br>2E5<br>FAM-NFQ-MGB<br>CT: 22.17	A01 10m ato2<br>U pGEM<br>2.51E4<br>FAM-NFQ-MGB<br>CT: 25.36	A01 10m ato2<br>U pGEM<br>2.5E4<br>FAM-NFQ-MGB<br>CT: 25.37	A01 10m ato2<br>U pGEM<br>2.35E4<br>FAM-NFQ-MGB<br>CT: 25.46	A04 1000m ato2<br>U pGEM<br>2.32E4<br>FAM-NFQ-MGB<br>CT: 25.48	A04 1000m ato2<br>U pGEM<br>2.52E4<br>FAM-NFQ-MGB<br>CT: 25.35	A04 1000m ato2<br>U pGEM<br>2.46E4<br>FAM-NFQ-MGB<br>CT: 25.39	A07 1000m ato2<br>U pGEM<br>2.33E4<br>FAM-NFQ-MGB<br>CT: 25.47	A07 1000m ato2<br>U pGEM<br>2.48E4<br>FAM-NFQ-MGB<br>CT: 25.38	A07 1000m ato2<br>U pGEM<br>2.4E4<br>FAM-NFQ-MGB<br>CT: 25.43	
C	S pGEM<br>2E4<br>FAM-NFQ-MGB<br>CT: 25.77	S pGEM<br>2E4<br>FAM-NFQ-MGB<br>CT: 25.48	A01 BTM saki2<br>U pGEM<br>FAM-NFQ-MGB<br>CT: 26.48	A01 BTM saki2<br>U pGEM<br>FAM-NFQ-MGB<br>CT: 26.41	A01 BTM saki2<br>U pGEM<br>2.38E4<br>FAM-NFQ-MGB<br>CT: 26.49	A04 3000m saki2<br>U pGEM<br>1.08E4<br>FAM-NFQ-MGB<br>CT: 26.68	A04 3000m saki2<br>U pGEM<br>9.93E3<br>FAM-NFQ-MGB<br>CT: 26.81	A04 3000m saki2<br>U pGEM<br>1.06E4<br>FAM-NFQ-MGB<br>CT: 26.7	A07 3000m saki2<br>U pGEM<br>1.1E4<br>FAM-NFQ-MGB<br>CT: 26.64	A07 3000m saki2<br>U pGEM<br>1.28E4<br>FAM-NFQ-MGB<br>CT: 26.41	A07 3000m saki2<br>U pGEM<br>1.1E4<br>FAM-NFQ-MGB<br>CT: 26.65	
D	S pGEM<br>2E3<br>FAM-NFQ-MGB<br>CT: 29.48	S pGEM<br>2E3<br>FAM-NFQ-MGB<br>CT: 29.52	A01 BTM ato2<br>U pGEM<br>2.28E4<br>FAM-NFQ-MGB<br>CT: 25.51	A01 BTM ato2<br>U pGEM<br>2.53E4<br>FAM-NFQ-MGB<br>CT: 25.35	A01 BTM ato2<br>U pGEM<br>2.23E4<br>FAM-NFQ-MGB<br>CT: 25.55	A04 3000m ato2<br>U pGEM<br>2.36E4<br>FAM-NFQ-MGB<br>CT: 25.46	A04 3000m ato2<br>U pGEM<br>2.42E4<br>FAM-NFQ-MGB<br>CT: 25.42	A04 3000m ato2<br>U pGEM<br>2.37E4<br>FAM-NFQ-MGB<br>CT: 25.45	A07 3000m ato2<br>U pGEM<br>2.67E4<br>FAM-NFQ-MGB<br>CT: 25.26	A07 3000m ato2<br>U pGEM<br>2.67E4<br>FAM-NFQ-MGB<br>CT: 25.26	A07 3000m ato2<br>U pGEM<br>2.5E4<br>FAM-NFQ-MGB<br>CT: 25.36	
E	S pGEM<br>200<br>FAM-NFQ-MGB<br>CT: 33.11	S pGEM<br>200<br>FAM-NFQ-MGB<br>CT: 32.96	A04 SCM saki2<br>U pGEM<br>8.21E3<br>FAM-NFQ-MGB<br>CT: 27.11	A04 SCM saki2<br>U pGEM<br>7.97E3<br>FAM-NFQ-MGB<br>CT: 27.15	A04 SCM saki2<br>U pGEM<br>8.7E3<br>FAM-NFQ-MGB<br>CT: 27.01	A07 SCM saki2<br>U pGEM<br>8.87E3<br>FAM-NFQ-MGB<br>CT: 26.98	A07 SCM saki2<br>U pGEM<br>9.14E3<br>FAM-NFQ-MGB<br>CT: 26.94	A07 SCM saki2<br>U pGEM<br>5.73E4<br>FAM-NFQ-MGB<br>CT: 26.83				
F	1<br>S pGEM<br>20<br>FAM-NFQ-MGB<br>CT: Undetermined	1<br>S pGEM<br>20<br>FAM-NFQ-MGB<br>CT: 35.67	A04 SCM ato2<br>U pGEM<br>2.76E4<br>FAM-NFQ-MGB<br>CT: 25.21	A04 SCM ato2<br>U pGEM<br>2.54E4<br>FAM-NFQ-MGB<br>CT: 25.34	A04 SCM ato2<br>U pGEM<br>2.6E4<br>FAM-NFQ-MGB<br>CT: 25.3	A07 SCM ato2<br>U pGEM<br>FAM-NFQ-MGB<br>CT: Undetermined	A07 SCM ato2<br>U pGEM<br>2.78E4<br>FAM-NFQ-MGB<br>CT: 25.2	A07 SCM ato2<br>U pGEM<br>2.5E4<br>FAM-NFQ-MGB<br>CT: 25.36				
G			A04 200m saki2<br>U pGEM<br>1.06E4<br>FAM-NFQ-MGB<br>CT: 26.71	A04 200m saki2<br>U pGEM<br>1.01E4<br>FAM-NFQ-MGB<br>CT: 26.79	A04 200m saki2<br>U pGEM<br>1.01E4<br>FAM-NFQ-MGB<br>CT: 26.78	A07 200m saki2<br>U pGEM<br>1.28E4<br>FAM-NFQ-MGB<br>CT: 26.41	A07 200m saki2<br>U pGEM<br>1.23E4<br>FAM-NFQ-MGB<br>CT: 26.48	A07 200m saki2<br>U pGEM<br>1.33E4<br>FAM-NFQ-MGB<br>CT: 26.35				N pGEM<br>FAM-NFQ-MGB<br>CT: Undetermined
H	3<br>Comf2E4<br>U pGEM<br>FAM-NFQ-MGB<br>CT: Undetermined	3<br>Comf2E4<br>U pGEM<br>FAM-NFQ-MGB<br>CT: Undetermined	A04 200m ato2<br>U pGEM<br>2.6E4<br>FAM-NFQ-MGB<br>CT: 25.3	A04 200m ato2<br>U pGEM<br>2.77E4<br>FAM-NFQ-MGB<br>CT: 25.2	A04 200m ato2<br>U pGEM<br>2.76E4<br>FAM-NFQ-MGB<br>CT: 25.21	A07 200m ato2<br>U pGEM<br>2.42E4<br>FAM-NFQ-MGB<br>CT: 25.42	A07 200m ato2<br>U pGEM<br>2.29E4<br>FAM-NFQ-MGB<br>CT: 25.5	A07 200m ato2<br>U pGEM<br>2.46E4<br>FAM-NFQ-MGB<br>CT: 25.39				N pGEM<br>FAM-NFQ-MGB<br>CT: Undetermined

Standard Curves

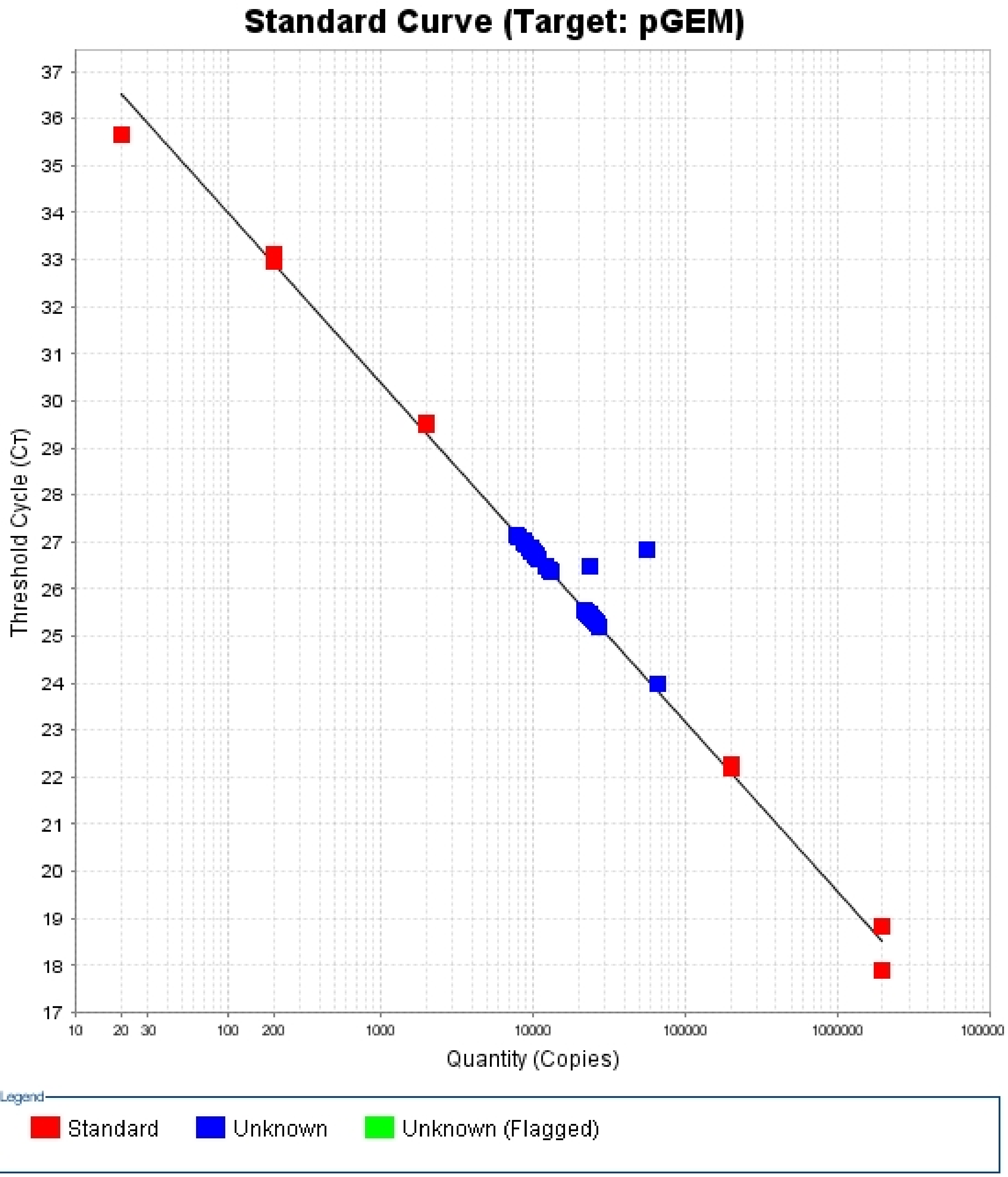

slope:-3.601 Y-Intercept:41.203 R^2:0.997 Eff%:89.526

Results Table

Well		Sample	Target	Task	Quantity	C
A1			pGEM	STANDARD	2000000	17.9127
A2			pGEM	STANDARD	2000000	18.8374
A3		A01 10m saki2	pGEM	UNKNOWN	10382.377	26.7389
A4		A01 10m saki2	pGEM	UNKNOWN	9621.8008	26.8579
A5		A01 10m saki2	pGEM	UNKNOWN	10682.8096	26.6943
A6		A04 1000m	pGEM	UNKNOWN	10414.0654	26.7341
A7		A04 1000m	pGEM	UNKNOWN	9736.624	26.8393
A8		A04 1000m	pGEM	UNKNOWN	10619.3213	26.7036
A9		A07 1000m	pGEM	UNKNOWN		
A10		A07 1000m	pGEM	UNKNOWN	11018.3076	26.6459
A11		A07 1000m	pGEM	UNKNOWN	9852.4463	26.8209
B1			pGEM	STANDARD	200000	22.2785
B2			pGEM	STANDARD	200000	22.1693
B3		A01 10m ato2	pGEM	UNKNOWN	25112.6719	25.3574
B4		A01 10m ato2	pGEM	UNKNOWN	24986.1445	25.3653
B5		A01 10m ato2	pGEM	UNKNOWN	23481.5977	25.4625
B6		A04 1000m ato2	pGEM	UNKNOWN	23205.9805	25.4809
B7		A04 1000m ato2	pGEM	UNKNOWN	25214.0879	25.3511
B8		A04 1000m ato2	pGEM	UNKNOWN	24552.793	25.3927
B9		A07 1000m ato2	pGEM	UNKNOWN	23319.084	25.4733
B10		A07 1000m ato2	pGEM	UNKNOWN	24804.8086	25.3767
B11		A07 1000m ato2	pGEM	UNKNOWN	24025.75	25.4266
C1			pGEM	STANDARD		25.7731
C2			pGEM	STANDARD		25.4784
C3		A01 BTM saki2	pGEM	UNKNOWN		26.4768
C4		A01 BTM saki2	pGEM	UNKNOWN		26.4064
C5		A01 BTM saki2	pGEM	UNKNOWN	23825.3418	26.4857
C6		A04 3000m	pGEM	UNKNOWN	10782.2002	26.6798
C7		A04 3000m	pGEM	UNKNOWN	9931.2041	26.8084
C8		A04 3000m	pGEM	UNKNOWN	10636.2598	26.7011

Well		Sample	Target	Task	Quantity	C
C9		A07 3000m	pGEM	UNKNOWN	11029.7344	26.6443
C10		A07 3000m	pGEM	UNKNOWN	12836.3799	26.4071
C11		A07 3000m	pGEM	UNKNOWN	10989.376	26.65
D1			pGEM	STANDARD	2000	29.48
D2			pGEM	STANDARD	2000	29.5189
D3		A01 BTM ato2	pGEM	UNKNOWN	22751.6992	25.5119
D4		A01 BTM ato2	pGEM	UNKNOWN	25261.6094	25.3482
D5		A01 BTM ato2	pGEM	UNKNOWN	22261.8789	25.5459
D6		A04 3000m ato2	pGEM	UNKNOWN	23581.9199	25.4558
D7		A04 3000m ato2	pGEM	UNKNOWN	24196.9648	25.4155
D8		A04 3000m ato2	pGEM	UNKNOWN	23661.166	25.4505
D9		A07 3000m ato2	pGEM	UNKNOWN	26652.082	25.2644
D10		A07 3000m ato2	pGEM	UNKNOWN	26674.2578	25.2631
D11		A07 3000m ato2	pGEM	UNKNOWN	25010.0137	25.3638
E1			pGEM	STANDARD	200	33.1078
E2			pGEM	STANDARD	200	32.963
E3		A04 SCM saki2	pGEM	UNKNOWN	8213.4736	27.1054
E4		A04 SCM saki2	pGEM	UNKNOWN	7966.916	27.1531
E5		A04 SCM saki2	pGEM	UNKNOWN	8702.7705	27.0149
E6		A07 SCM saki2	pGEM	UNKNOWN	8870.8896	26.985
E7		A07 SCM saki2	pGEM	UNKNOWN	9137.084	26.9387
E8		A07 SCM saki2	pGEM	UNKNOWN	57318.6719	26.8331
F1			pGEM	STANDARD		
F2			pGEM	STANDARD	20	35.6695
F3		A04 SCM ato2	pGEM	UNKNOWN	27556.3965	25.2122
F4		A04 SCM ato2	pGEM	UNKNOWN	25432.7266	25.3376
F5		A04 SCM ato2	pGEM	UNKNOWN	26029.0762	25.3014
F6		A07 SCM ato2	pGEM	UNKNOWN		
F7		A07 SCM ato2	pGEM	UNKNOWN	27816.334	25.1975
F8		A07 SCM ato2	pGEM	UNKNOWN	25043.8301	25.3617
G3		A04 200m saki2	pGEM	UNKNOWN	10592.5605	26.7076
G4		A04 200m saki2	pGEM	UNKNOWN	10070.8838	26.7866

Well		Sample	Target	Task	Quantity	C
G5		A04 200m saki2	pGEM	UNKNOWN	10121.832	26.7787
G6		A07 200m saki2	pGEM	UNKNOWN	12827.6328	26.4081
G7		A07 200m saki2	pGEM	UNKNOWN	12265.5176	26.4782
G8		A07 200m saki2	pGEM	UNKNOWN	13320.1602	26.3492
G12			pGEM	NTC		Undetermined
H1		Comf2E4	pGEM	UNKNOWN		Undetermined
H2		Comf2E4	pGEM	UNKNOWN		Undetermined
H3		A04 200m ato2	pGEM	UNKNOWN	26009.2773	25.3026
H4		A04 200m ato2	pGEM	UNKNOWN	27718.9141	25.203
H5		A04 200m ato2	pGEM	UNKNOWN	27624.6641	25.2083
H6		A07 200m ato2	pGEM	UNKNOWN	24166.6504	25.4175
H7		A07 200m ato2	pGEM	UNKNOWN	22893.8926	25.5021
H8		A07 200m ato2	pGEM	UNKNOWN	24594.9258	25.39
H12			pGEM	NTC		Undetermined

QC Summary

Total Wells	96	Processed Wells	76	Targets Used	1
Well Setup	76	Flagged Wells	6	Samples Used	21

Flag	Name	Frequency	Locations
AMPNC	Amplification in negative control	0	
BADROX	Bad passive reference signal	2	H1, H2
BLFAIL	Baseline algorithm failed	0	
CTFAIL	C algorithm failed	0	
EXPFAIL	Exponential algorithm failed	2	H1, H2
HIGHSD	High standard deviation in replicate group	4	A1, A2, F1, F2
NOAMP	No amplification	0	
NOISE	Noise higher than others in plate	0	
NOSIGNAL	No signal in well	2	H1, H2
OFFSCALE	Fluorescence is offscale	0	
OUTLIERRG	Outlier in replicate group	0	
SPIKE	Noise spikes	0	
THOLDFAIL	Thresholding algorithm failed	0	
