# Supplementary material for: Ecological impact of algal-preying protists aplanochytrids (Labyrinthulea) as a source of docosahexaenoic acid for copepods in marine environments
Source: ISME Commun. 2026 Jun 26;6(1):ycag178. doi: 10.1093/ismeco/ycag178 (PMC13685785; doi:10.1093/ismeco/ycag178)
Supplement: Supplementary file 2 [file supplementary_materials_ycag178.zip › Morimoto_ISME_C_3rdrev_supplemental_data.zip!/Morimoto_ISME_C_3rdrev_supplemental_data/Supplemental_Data_2/Standard Curve/DNA recovery/2110/DnaRec2110AlineA01-A07N=3.pdf]

Experiment Results Report

230712DnaRec2110AlineA01-A07N=3

Experiment Summary

Experiment Name	:230712DnaRec2110AlineA01-A07N=3
Experiment Type	:Quantitation - Standard Curve
File Name	:230712DnaRec2110AlineA01-A07N=3.eds
Run Started	:2023 Jul 12 12:15:35 PM
Run Finished	:2023 Jul 12 1:54:59 PM
Run Duration	:99 minutes 23 seconds
Date Modified	:2023 Jul 12 2:03:41 PM
User	:
Number of wells used	:76
Number of wells with results	:76
Instrument Name	:
Instrument Type	:Applied Biosystems StepOnePlus™ Instrument
Comments	:

Results Summary

Sample	Target	Quantity (Mean)	Quantity (Std Dev)	C (Mean)	C (Std Dev)
A01 10m ato1	pGEM	23140.46	736.97	25.42	0.05
A01 10m saki1	pGEM	10615.73	687.48	26.63	0.10
A01 BTM ato1	pGEM	21639.08	1322.52	25.53	0.10
A01 BTM saki1	pGEM	13111.23	670.68	26.31	0.08
A04 1000m ato1	pGEM	23182.07	599.45	25.42	0.04
A04 1000m saki1	pGEM	8508.13	616.07	26.98	0.11
A04 200m ato1	pGEM	24207.72	1365.93	25.35	0.09
A04 200m saki1	pGEM	10655.03	248.70	26.63	0.04
A04 3000m ato1	pGEM	23931.29	1742.82	25.37	0.11
A04 3000m saki1	pGEM	9546.04	190.69	26.80	0.03
A04 SCM ato1	pGEM	22607.10	1333.17	25.46	0.09
A04 SCM saki1	pGEM	7689.25	503.71	27.14	0.10
A07 1000m ato1	pGEM	22170.75	1195.40	25.49	0.08
A07 1000m saki1	pGEM	8985.94	534.04	26.89	0.09
A07 200m ato1	pGEM	22815.36	730.89	25.45	0.05
A07 200m saki1	pGEM	11867.40	672.82	26.46	0.09
A07 3000m ato1	pGEM	22253.18	192.16	25.48	0.01
A07 3000m saki1	pGEM	9961.25	1235.89	26.74	0.20
A07 SCM ato1	pGEM	22664.21	725.85	25.46	0.05
A07 SCM saki1	pGEM	9466.40	528.43	26.81	0.09
Comf2E4	pGEM	21544.57	2090.48	25.54	0.15

Plate Layout

	1	2	3	4	5	6	7	8	9	10	11	12
A	1<br>S pGEM<br>2E6<br>FAM-NFQ-MGB<br>CT: 17.93	1<br>S pGEM<br>2E6<br>FAM-NFQ-MGB<br>CT: 18.97	A01 10m saki1<br>U pGEM<br>1.09E4<br>FAM-NFQ-MGB<br>CT: 26.59	A01 10m saki1<br>U pGEM<br>1.11E4<br>FAM-NFQ-MGB<br>CT: 26.56	A01 10m saki1<br>U pGEM<br>9.84E3<br>FAM-NFQ-MGB<br>CT: 26.75	A04 1000m saki1<br>U pGEM<br>7.88E3<br>FAM-NFQ-MGB<br>CT: 27.09	A04 1000m saki1<br>U pGEM<br>8.52E3<br>FAM-NFQ-MGB<br>CT: 26.97	A04 1000m saki1<br>U pGEM<br>9.12E3<br>FAM-NFQ-MGB<br>CT: 26.87	A07 1000m saki1<br>U pGEM<br>9.54E3<br>FAM-NFQ-MGB<br>CT: 26.8	A07 1000m saki1<br>U pGEM<br>8.94E3<br>FAM-NFQ-MGB<br>CT: 26.9	A07 1000m saki1<br>U pGEM<br>8.47E3<br>FAM-NFQ-MGB<br>CT: 26.98	
B	S pGEM<br>2E5<br>FAM-NFQ-MGB<br>CT: 22.13	S pGEM<br>2E5<br>FAM-NFQ-MGB<br>CT: 22.06	A01 10m ato1<br>U pGEM<br>2.33E4<br>FAM-NFQ-MGB<br>CT: 25.42	A01 10m ato1<br>U pGEM<br>2.24E4<br>FAM-NFQ-MGB<br>CT: 25.48	A01 10m ato1<br>U pGEM<br>2.38E4<br>FAM-NFQ-MGB<br>CT: 25.38	A04 1000m ato1<br>U pGEM<br>2.27E4<br>FAM-NFQ-MGB<br>CT: 25.45	A04 1000m ato1<br>U pGEM<br>2.38E4<br>FAM-NFQ-MGB<br>CT: 25.38	A04 1000m ato1<br>U pGEM<br>2.3E4<br>FAM-NFQ-MGB<br>CT: 25.43	A07 1000m ato1<br>U pGEM<br>2.35E4<br>FAM-NFQ-MGB<br>CT: 25.4	A07 1000m ato1<br>U pGEM<br>2.2E4<br>FAM-NFQ-MGB<br>CT: 25.5	A07 1000m ato1<br>U pGEM<br>2.11E4<br>FAM-NFQ-MGB<br>CT: 25.57	
C	S pGEM<br>2E4<br>FAM-NFQ-MGB<br>CT: 25.6	S pGEM<br>2E4<br>FAM-NFQ-MGB<br>CT: 25.45	A01 BTM saki1<br>U pGEM<br>1.32E4<br>FAM-NFQ-MGB<br>CT: 26.3	A01 BTM saki1<br>U pGEM<br>1.37E4<br>FAM-NFQ-MGB<br>CT: 26.23	A01 BTM saki1<br>U pGEM<br>1.24E4<br>FAM-NFQ-MGB<br>CT: 26.39	A04 3000m saki1<br>U pGEM<br>9.56E3<br>FAM-NFQ-MGB<br>CT: 26.8	A04 3000m saki1<br>U pGEM<br>9.35E3<br>FAM-NFQ-MGB<br>CT: 26.83	A04 3000m saki1<br>U pGEM<br>9.73E3<br>FAM-NFQ-MGB<br>CT: 26.77	A07 3000m saki1<br>U pGEM<br>8.55E3<br>FAM-NFQ-MGB<br>CT: 26.97	A07 3000m saki1<br>U pGEM<br>1.08E4<br>FAM-NFQ-MGB<br>CT: 26.6	A07 3000m saki1<br>U pGEM<br>1.05E4<br>FAM-NFQ-MGB<br>CT: 26.65	
D	S pGEM<br>2E3<br>FAM-NFQ-MGB<br>CT: 29.3	S pGEM<br>2E3<br>FAM-NFQ-MGB<br>CT: 29.53	A01 BTM ato1<br>U pGEM<br>2.28E4<br>FAM-NFQ-MGB<br>CT: 25.44	A01 BTM ato1<br>U pGEM<br>2.19E4<br>FAM-NFQ-MGB<br>CT: 25.51	A01 BTM ato1<br>U pGEM<br>2.02E4<br>FAM-NFQ-MGB<br>CT: 25.63	A04 3000m ato1<br>U pGEM<br>2.32E4<br>FAM-NFQ-MGB<br>CT: 25.42	A04 3000m ato1<br>U pGEM<br>2.59E4<br>FAM-NFQ-MGB<br>CT: 25.25	A04 3000m ato1<br>U pGEM<br>2.27E4<br>FAM-NFQ-MGB<br>CT: 25.46	A07 3000m ato1<br>U pGEM<br>2.21E4<br>FAM-NFQ-MGB<br>CT: 25.49	A07 3000m ato1<br>U pGEM<br>2.25E4<br>FAM-NFQ-MGB<br>CT: 25.47	A07 3000m ato1<br>U pGEM<br>2.21E4<br>FAM-NFQ-MGB<br>CT: 25.49	
E	S pGEM<br>200<br>FAM-NFQ-MGB<br>CT: 32.87	S pGEM<br>200<br>FAM-NFQ-MGB<br>CT: 33.11	A04 SCM saki1<br>U pGEM<br>7.13E3<br>FAM-NFQ-MGB<br>CT: 27.25	A04 SCM saki1<br>U pGEM<br>7.82E3<br>FAM-NFQ-MGB<br>CT: 27.11	A04 SCM saki1<br>U pGEM<br>8.12E3<br>FAM-NFQ-MGB<br>CT: 27.05	A07 SCM saki1<br>U pGEM<br>9.04E3<br>FAM-NFQ-MGB<br>CT: 26.88	A07 SCM saki1<br>U pGEM<br>1.01E4<br>FAM-NFQ-MGB<br>CT: 26.72	A07 SCM saki1<br>U pGEM<br>9.31E3<br>FAM-NFQ-MGB<br>CT: 26.84				
F	S pGEM<br>20<br>FAM-NFQ-MGB<br>CT: 36.32	S pGEM<br>20<br>FAM-NFQ-MGB<br>CT: 35.98	A04 SCM ato1<br>U pGEM<br>2.35E4<br>FAM-NFQ-MGB<br>CT: 25.4	A04 SCM ato1<br>U pGEM<br>2.11E4<br>FAM-NFQ-MGB<br>CT: 25.57	A04 SCM ato1<br>U pGEM<br>2.32E4<br>FAM-NFQ-MGB<br>CT: 25.42	A07 SCM ato1<br>U pGEM<br>2.25E4<br>FAM-NFQ-MGB<br>CT: 25.47	A07 SCM ato1<br>U pGEM<br>2.35E4<br>FAM-NFQ-MGB<br>CT: 25.4	A07 SCM ato1<br>U pGEM<br>2.2E4<br>FAM-NFQ-MGB<br>CT: 25.5				
G			A04 200m saki1<br>U pGEM<br>1.09E4<br>FAM-NFQ-MGB<br>CT: 26.59	A04 200m saki1<br>U pGEM<br>1.06E4<br>FAM-NFQ-MGB<br>CT: 26.63	A04 200m saki1<br>U pGEM<br>1.04E4<br>FAM-NFQ-MGB<br>CT: 26.66	A07 200m saki1<br>U pGEM<br>1.22E4<br>FAM-NFQ-MGB<br>CT: 26.42	A07 200m saki1<br>U pGEM<br>1.11E4<br>FAM-NFQ-MGB<br>CT: 26.56	A07 200m saki1<br>U pGEM<br>1.23E4<br>FAM-NFQ-MGB<br>CT: 26.4				N pGEM<br>FAM-NFQ-MGB<br>CT: Undetermined
H	Comf2E4<br>U pGEM<br>2.01E4<br>FAM-NFQ-MGB<br>CT: 25.64	Comf2E4<br>U pGEM<br>2.3E4<br>FAM-NFQ-MGB<br>CT: 25.43	A04 200m ato1<br>U pGEM<br>2.57E4<br>FAM-NFQ-MGB<br>CT: 25.26	A04 200m ato1<br>U pGEM<br>2.4E4<br>FAM-NFQ-MGB<br>CT: 25.37	A04 200m ato1<br>U pGEM<br>2.3E4<br>FAM-NFQ-MGB<br>CT: 25.44	A07 200m ato1<br>U pGEM<br>2.22E4<br>FAM-NFQ-MGB<br>CT: 25.48	A07 200m ato1<br>U pGEM<br>2.36E4<br>FAM-NFQ-MGB<br>CT: 25.39	A07 200m ato1<br>U pGEM<br>2.26E4<br>FAM-NFQ-MGB<br>CT: 25.46				N pGEM<br>FAM-NFQ-MGB<br>CT: Undetermined

Standard Curves

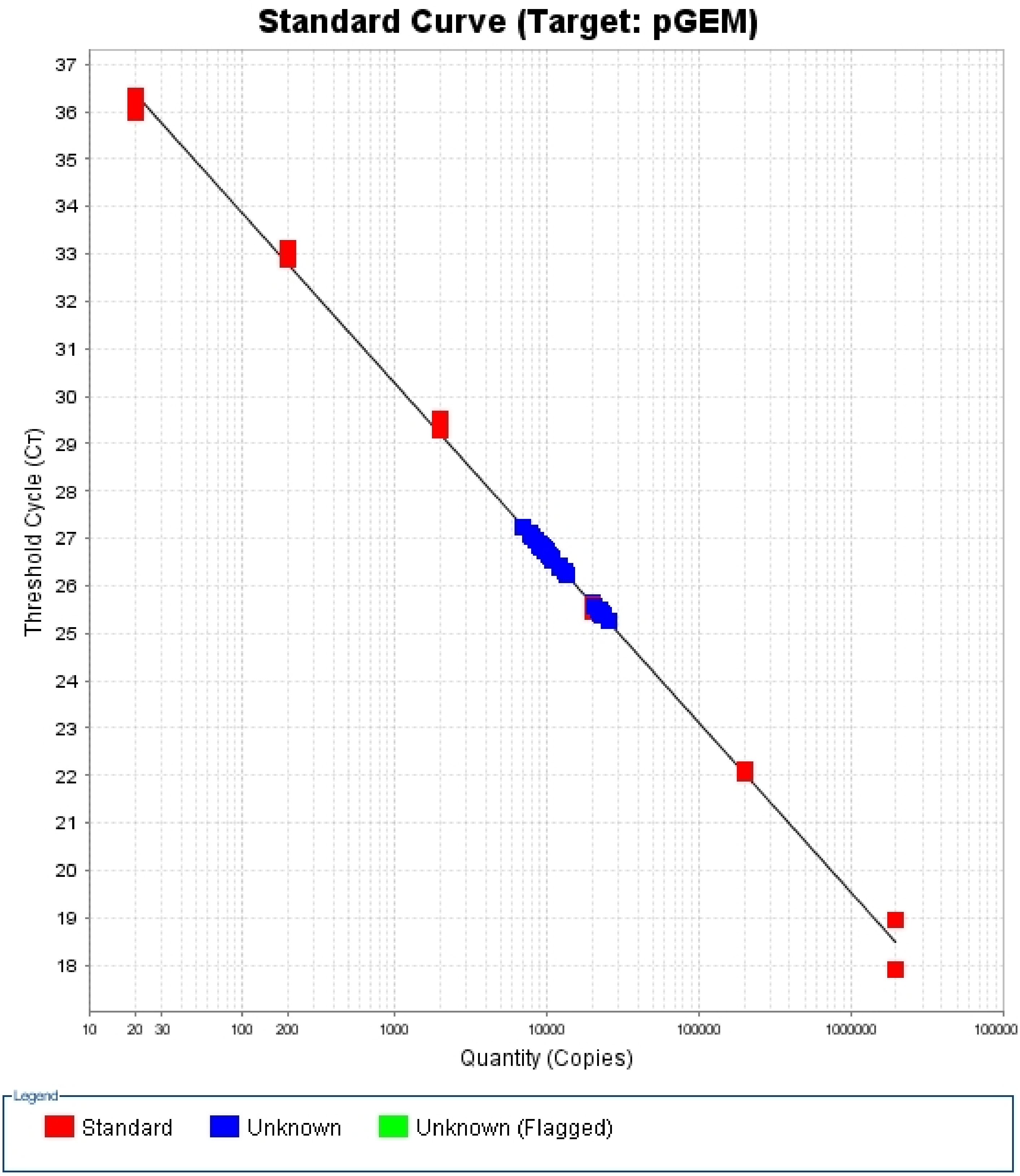

slope:-3.574 | Y-Intercept:41.021 | R^2:0.998 | Eff%:90.465

Results Table

Well		Sample	Target	Task	Quantity	C
A1			pGEM	STANDARD	2000000	17.9291
A2			pGEM	STANDARD	2000000	18.9651
A3		A01 10m saki1	pGEM	UNKNOWN	10879.5449	26.5946
A4		A01 10m saki1	pGEM	UNKNOWN	11132.2227	26.5589
A5		A01 10m saki1	pGEM	UNKNOWN	9835.4131	26.7512
A6		A04 1000m	pGEM	UNKNOWN	7884.4111	27.0943
A7		A04 1000m	pGEM	UNKNOWN	8523.7373	26.9733
A8		A04 1000m	pGEM	UNKNOWN	9116.249	26.869
A9		A07 1000m	pGEM	UNKNOWN	9540.3135	26.7984
A10		A07 1000m	pGEM	UNKNOWN	8942.6309	26.8989
A11		A07 1000m	pGEM	UNKNOWN	8474.8662	26.9822
B1			pGEM	STANDARD	200000	22.1337
B2			pGEM	STANDARD	200000	22.0563
B3		A01 10m ato1	pGEM	UNKNOWN	23250.1895	25.4159
B4		A01 10m ato1	pGEM	UNKNOWN	22354.7812	25.4768
B5		A01 10m ato1	pGEM	UNKNOWN	23816.416	25.3785
B6		A04 1000m ato1	pGEM	UNKNOWN	22688.25	25.4538
B7		A04 1000m ato1	pGEM	UNKNOWN	23849.043	25.3764
B8		A04 1000m ato1	pGEM	UNKNOWN	23008.9297	25.4321
B9		A07 1000m ato1	pGEM	UNKNOWN	23454.0547	25.4023
B10		A07 1000m ato1	pGEM	UNKNOWN	21969.3633	25.5038
B11		A07 1000m ato1	pGEM	UNKNOWN	21088.8359	25.5673
C1			pGEM	STANDARD	20000	25.5969
C2			pGEM	STANDARD	20000	25.4481
C3		A01 BTM saki1	pGEM	UNKNOWN	13187.8896	26.2959
C4		A01 BTM saki1	pGEM	UNKNOWN	13740.2822	26.2322
C5		A01 BTM saki1	pGEM	UNKNOWN	12405.5059	26.3908
C6		A04 3000m	pGEM	UNKNOWN	9559.7129	26.7953
C7		A04 3000m	pGEM	UNKNOWN	9348.8838	26.8299
C8		A04 3000m	pGEM	UNKNOWN	9729.5273	26.768

Well		Sample	Target	Task	Quantity	C
C9		A07 3000m	pGEM	UNKNOWN	8547.6113	26.969
C10		A07 3000m	pGEM	UNKNOWN	10837.3115	26.6006
C11		A07 3000m	pGEM	UNKNOWN	10498.8271	26.6498
D1			pGEM	STANDARD	2000	29.3023
D2			pGEM	STANDARD	2000	29.5289
D3		A01 BTM ato1	pGEM	UNKNOWN	22843.4375	25.4433
D4		A01 BTM ato1	pGEM	UNKNOWN	21850.0586	25.5123
D5		A01 BTM ato1	pGEM	UNKNOWN	20223.7578	25.6323
D6		A04 3000m ato1	pGEM	UNKNOWN	23202.9219	25.419
D7		A04 3000m ato1	pGEM	UNKNOWN	25920.1406	25.2471
D8		A04 3000m ato1	pGEM	UNKNOWN	22670.8027	25.455
D9		A07 3000m ato1	pGEM	UNKNOWN	22138.3418	25.4919
D10		A07 3000m ato1	pGEM	UNKNOWN	22475.0176	25.4685
D11		A07 3000m ato1	pGEM	UNKNOWN	22146.1777	25.4914
E1			pGEM	STANDARD	200	32.8692
E2			pGEM	STANDARD	200	33.1054
E3		A04 SCM saki1	pGEM	UNKNOWN	7133.4243	27.2497
E4		A04 SCM saki1	pGEM	UNKNOWN	7818.7705	27.1073
E5		A04 SCM saki1	pGEM	UNKNOWN	8115.5552	27.0495
E6		A07 SCM saki1	pGEM	UNKNOWN	9035.5098	26.8828
E7		A07 SCM saki1	pGEM	UNKNOWN	10055.9912	26.7167
E8		A07 SCM saki1	pGEM	UNKNOWN	9307.6836	26.8368
F1			pGEM	STANDARD	20	36.3216
F2			pGEM	STANDARD	20	35.9785
F3		A04 SCM ato1	pGEM	UNKNOWN	23517.002	25.3982
F4		A04 SCM ato1	pGEM	UNKNOWN	21076.7891	25.5682
F5		A04 SCM ato1	pGEM	UNKNOWN	23227.5156	25.4174
F6		A07 SCM ato1	pGEM	UNKNOWN	22486.5645	25.4677
F7		A07 SCM ato1	pGEM	UNKNOWN	23462.3867	25.4018
F8		A07 SCM ato1	pGEM	UNKNOWN	22043.6777	25.4986
G3		A04 200m saki1	pGEM	UNKNOWN	10909.8691	26.5902
G4		A04 200m saki1	pGEM	UNKNOWN	10642.2617	26.6288

Well		Sample	Target	Task	Quantity	C
G5		A04 200m saki1	pGEM	UNKNOWN	10412.9561	26.6626
G6		A07 200m saki1	pGEM	UNKNOWN	12182.0977	26.419
G7		A07 200m saki1	pGEM	UNKNOWN	11094.8975	26.5641
G8		A07 200m saki1	pGEM	UNKNOWN	12325.1973	26.4009
G12			pGEM	NTC		Undetermined
H1		Comf2E4	pGEM	UNKNOWN	20066.3828	25.6444
H2		Comf2E4	pGEM	UNKNOWN	23022.7617	25.4311
H3		A04 200m ato1	pGEM	UNKNOWN	25664.5781	25.2625
H4		A04 200m ato1	pGEM	UNKNOWN	24002.6992	25.3664
H5		A04 200m ato1	pGEM	UNKNOWN	22955.8906	25.4356
H6		A07 200m ato1	pGEM	UNKNOWN	22246.1484	25.4844
H7		A07 200m ato1	pGEM	UNKNOWN	23639.5957	25.3901
H8		A07 200m ato1	pGEM	UNKNOWN	22560.3301	25.4626
H12			pGEM	NTC		Undetermined

QC Summary

Total Wells	96	Processed Wells	76	Targets Used	1
Well Setup	76	Flagged Wells	2	Samples Used	21

Flag	Name	Frequency	Locations
AMPNC	Amplification in negative control	0	
BADROX	Bad passive reference signal	0	
BLFAIL	Baseline algorithm failed	0	
CTFAIL	C algorithm failed	0	
EXPFAIL	Exponential algorithm failed	0	
HIGHSD	High standard deviation in replicate group	2	A1, A2
NOAMP	No amplification	0	
NOISE	Noise higher than others in plate	0	
NOSIGNAL	No signal in well	0	
OFFSCALE	Fluorescence is offscale	0	
OUTLIERRG	Outlier in replicate group	0	
SPIKE	Noise spikes	0	
THOLDFAIL	Thresholding algorithm failed	0	
