# Supplementary material for: Ecological impact of algal-preying protists aplanochytrids (Labyrinthulea) as a source of docosahexaenoic acid for copepods in marine environments
Source: ISME Commun. 2026 Jun 26;6(1):ycag178. doi: 10.1093/ismeco/ycag178 (PMC13685785; doi:10.1093/ismeco/ycag178)
Supplement: Supplementary file 2 [file supplementary_materials_ycag178.zip › Morimoto_ISME_C_3rdrev_supplemental_data.zip!/Morimoto_ISME_C_3rdrev_supplemental_data/Supplemental_Data_2/Standard Curve/DNA recovery/2205/DnaRecAline2205A11_N=3.pdf]

Experiment Results Report
230320DnaRecAline2205A11DnaExtN=3EmmByKurabo

Experiment Summary

Experiment Name	:	230320DnaRecAline2205A11DnaExtN=3EmmByKurabo
Experiment Type	:	Quantitation - Standard Curve
File Name	:	230320DnaRecAline2205A11DnaExtN=3EmmByKurabo.eds
Run Started	:	2023 Mar 20 3:45:05 PM
Run Finished	:	2023 Mar 20 5:19:38 PM
Run Duration	:	94 minutes 32 seconds
Date Modified	:	2023 Mar 20 5:36:16 PM
User	:	
Number of wells used	:	76
Number of wells with results	:	76
Instrument Name	:	
Instrument Type	:	Applied Biosystems StepOnePlus™ Instrument
Comments	:	

Results Summary

Sample	Target	Quantity (Mean)	Quantity (Std Dev)	C (Mean)	C (Std Dev)
2205 A11 1000m	pGEM	25650.28	685.15	24.79	0.04
2205 A11 1000m	pGEM	27437.66	648.57	24.68	0.04
2205 A11 1000m	pGEM	16506.99	2232.18	25.48	0.22
2205 A11 1000m	pGEM	17744.08	683.43	25.36	0.06
2205 A11 10m ato2	pGEM	25605.69	1760.48	24.79	0.11
2205 A11 10m ato3	pGEM	28264.76	2292.29	24.64	0.13
2205 A11 10m saki2	pGEM	14545.21	607.63	25.67	0.06
2205 A11 10m saki3	pGEM	16345.30	947.42	25.49	0.09
2205 A11 200m ato2	pGEM	24177.40	905.25	24.88	0.06
2205 A11 200m ato3	pGEM	27014.27	1416.96	24.71	0.08
2205 A11 200m	pGEM	15973.23	877.82	25.53	0.08
2205 A11 200m	pGEM	18610.52	806.81	25.29	0.07
2205 A11 3000m	pGEM	27143.05	1291.72	24.70	0.08
2205 A11 3000m	pGEM	25856.68	1436.78	24.77	0.09
2205 A11 3000m	pGEM	13672.12	874.24	25.77	0.10
2205 A11 3000m	pGEM	18891.23	1746.07	25.27	0.14
2205 A11 SCM ato2	pGEM	27948.86	844.28	24.65	0.05
2205 A11 SCM ato3	pGEM	26208.76	1857.89	24.75	0.11
2205 A11 SCM saki2	pGEM	17503.46	589.42	25.38	0.05
2205 A11 SCM saki3	pGEM	16064.64	1097.63	25.52	0.10
Comf2E4	pGEM	23313.17	2214.49	24.94	0.15

Plate Layout

	1	2	3	4	5	6	7	8	9	10	11	12
A	S pGEM<br>2E6<br>FAM-NFQ-MGB<br>CT: 18.29	S pGEM<br>2E6<br>FAM-NFQ-MGB<br>CT: 18.18		2205 A11 10m saki2<br>U pGEM<br>1.42E4<br>FAM-NFQ-MGB<br>CT: 25.71	2205 A11 10m saki2<br>U pGEM<br>1.42E4<br>FAM-NFQ-MGB<br>CT: 25.7	2205 A11 10m saki2<br>U pGEM<br>1.52E4<br>FAM-NFQ-MGB<br>CT: 25.6	2205 A11 3000m saki2<br>U pGEM<br>1.27E4<br>FAM-NFQ-MGB<br>CT: 25.88	2205 A11 10m saki3<br>U pGEM<br>1.53E4<br>FAM-NFQ-MGB<br>CT: 25.59	2205 A11 10m saki3<br>U pGEM<br>1.71E4<br>FAM-NFQ-MGB<br>CT: 25.42	2205 A11 10m saki3<br>U pGEM<br>1.67E4<br>FAM-NFQ-MGB<br>CT: 25.46	2205 A11 3000m saki3<br>U pGEM<br>1.75E4<br>FAM-NFQ-MGB<br>CT: 25.38	
B	S pGEM<br>2E5<br>FAM-NFQ-MGB<br>CT: 21.44	S pGEM<br>2E5<br>FAM-NFQ-MGB<br>CT: 21.42		2205 A11 10m ato2<br>U pGEM<br>2.72E4<br>FAM-NFQ-MGB<br>CT: 24.7	2205 A11 10m ato2<br>U pGEM<br>2.37E4<br>FAM-NFQ-MGB<br>CT: 24.91	2205 A11 10m ato2<br>U pGEM<br>2.59E4<br>FAM-NFQ-MGB<br>CT: 24.77	2205 A11 3000m saki2<br>U pGEM<br>1.41E4<br>FAM-NFQ-MGB<br>CT: 25.72	2205 A11 10m ato3<br>U pGEM<br>2.58E4<br>FAM-NFQ-MGB<br>CT: 24.78	2205 A11 10m ato3<br>U pGEM<br>3.03E4<br>FAM-NFQ-MGB<br>CT: 24.52	2205 A11 10m ato3<br>U pGEM<br>2.87E4<br>FAM-NFQ-MGB<br>CT: 24.61	2205 A11 3000m saki3<br>U pGEM<br>1.83E4<br>FAM-NFQ-MGB<br>CT: 25.31	
C	S pGEM<br>2E4<br>FAM-NFQ-MGB<br>CT: 25	S pGEM<br>2E4<br>FAM-NFQ-MGB<br>CT: 24.92		2205 A11 SCM saki2<br>U pGEM<br>1.81E4<br>FAM-NFQ-MGB<br>CT: 25.33	2205 A11 SCM saki2<br>U pGEM<br>1.69E4<br>FAM-NFQ-MGB<br>CT: 25.44	2205 A11 SCM saki2<br>U pGEM<br>1.76E4<br>FAM-NFQ-MGB<br>CT: 25.37	2205 A11 3000m saki2<br>U pGEM<br>1.43E4<br>FAM-NFQ-MGB<br>CT: 25.7	2205 A11 SCM saki3<br>U pGEM<br>1.57E4<br>FAM-NFQ-MGB<br>CT: 25.55	2205 A11 SCM saki3<br>U pGEM<br>1.52E4<br>FAM-NFQ-MGB<br>CT: 25.6	2205 A11 SCM saki3<br>U pGEM<br>1.73E4<br>FAM-NFQ-MGB<br>CT: 25.4	2205 A11 3000m saki3<br>U pGEM<br>2.09E4<br>FAM-NFQ-MGB<br>CT: 25.11	
D	S pGEM<br>2E3<br>FAM-NFQ-MGB<br>CT: 28.43	S pGEM<br>2E3<br>FAM-NFQ-MGB<br>CT: 28.43		2205 A11 SCM ato2<br>U pGEM<br>2.87E4<br>FAM-NFQ-MGB<br>CT: 24.61	2205 A11 SCM ato2<br>U pGEM<br>2.82E4<br>FAM-NFQ-MGB<br>CT: 24.64	2205 A11 SCM ato2<br>U pGEM<br>2.7E4<br>FAM-NFQ-MGB<br>CT: 24.7	2205 A11 3000m ato2<br>U pGEM<br>2.79E4<br>FAM-NFQ-MGB<br>CT: 24.66	2205 A11 SCM ato3<br>U pGEM<br>2.49E4<br>FAM-NFQ-MGB<br>CT: 24.83	2205 A11 SCM ato3<br>U pGEM<br>2.54E4<br>FAM-NFQ-MGB<br>CT: 24.8	2205 A11 SCM ato3<br>U pGEM<br>2.83E4<br>FAM-NFQ-MGB<br>CT: 24.63	2205 A11 3000m ato3<br>U pGEM<br>2.61E4<br>FAM-NFQ-MGB<br>CT: 24.76	
E	S pGEM<br>200<br>FAM-NFQ-MGB<br>CT: 32.82	S pGEM<br>200<br>FAM-NFQ-MGB<br>CT: 33.3		2205 A11 200m saki2<br>U pGEM<br>1.57E4<br>FAM-NFQ-MGB<br>CT: 25.55	2205 A11 200m saki2<br>U pGEM<br>1.7E4<br>FAM-NFQ-MGB<br>CT: 25.43	2205 A11 200m saki2<br>U pGEM<br>1.53E4<br>FAM-NFQ-MGB<br>CT: 25.59	2205 A11 3000m ato2<br>U pGEM<br>2.57E4<br>FAM-NFQ-MGB<br>CT: 24.79	2205 A11 200m saki3<br>U pGEM<br>1.77E4<br>FAM-NFQ-MGB<br>CT: 25.37	2205 A11 200m saki3<br>U pGEM<br>1.91E4<br>FAM-NFQ-MGB<br>CT: 25.25	2205 A11 200m saki3<br>U pGEM<br>1.91E4<br>FAM-NFQ-MGB<br>CT: 25.25	2205 A11 3000m ato3<br>U pGEM<br>2.43E4<br>FAM-NFQ-MGB<br>CT: 24.87	
F	S pGEM<br>20<br>FAM-NFQ-MGB<br>CT: 35.99	S pGEM<br>20<br>FAM-NFQ-MGB<br>CT: 35.37		2205 A11 200m ato2<br>U pGEM<br>2.32E4<br>FAM-NFQ-MGB<br>CT: 24.94	2205 A11 200m ato2<br>U pGEM<br>2.44E4<br>FAM-NFQ-MGB<br>CT: 24.86	2205 A11 200m ato2<br>U pGEM<br>2.49E4<br>FAM-NFQ-MGB<br>CT: 24.83	2205 A11 3000m ato2<br>U pGEM<br>2.79E4<br>FAM-NFQ-MGB<br>CT: 24.66	2205 A11 200m ato3<br>U pGEM<br>2.55E4<br>FAM-NFQ-MGB<br>CT: 24.8	2205 A11 200m ato3<br>U pGEM<br>2.73E4<br>FAM-NFQ-MGB<br>CT: 24.69	2205 A11 200m ato3<br>U pGEM<br>2.82E4<br>FAM-NFQ-MGB<br>CT: 24.64	2205 A11 3000m ato3<br>U pGEM<br>2.72E4<br>FAM-NFQ-MGB<br>CT: 24.7	
G				2205 A11 1000m saki2<br>U pGEM<br>1.7E4<br>FAM-NFQ-MGB<br>CT: 25.43	2205 A11 1000m saki2<br>U pGEM<br>1.84E4<br>FAM-NFQ-MGB<br>CT: 25.3	2205 A11 1000m saki2<br>U pGEM<br>1.41E4<br>FAM-NFQ-MGB<br>CT: 25.72		2205 A11 1000m saki3<br>U pGEM<br>1.74E4<br>FAM-NFQ-MGB<br>CT: 25.39	2205 A11 1000m saki3<br>U pGEM<br>1.73E4<br>FAM-NFQ-MGB<br>CT: 25.4	2205 A11 1000m saki3<br>U pGEM<br>1.85E4<br>FAM-NFQ-MGB<br>CT: 25.29		N pGEM<br>FAM-NFQ-MGB<br>CT: Undetermined
H	Comf2E4<br>U pGEM<br>2.17E4<br>FAM-NFQ-MGB<br>CT: 25.04	Comf2E4<br>U pGEM<br>2.49E4<br>FAM-NFQ-MGB<br>CT: 24.83		2205 A11 1000m ato2<br>U pGEM<br>2.63E4<br>FAM-NFQ-MGB<br>CT: 24.74	2205 A11 1000m ato2<br>U pGEM<br>2.5E4<br>FAM-NFQ-MGB<br>CT: 24.83	2205 A11 1000m ato2<br>U pGEM<br>2.56E4<br>FAM-NFQ-MGB<br>CT: 24.79		2205 A11 1000m ato3<br>U pGEM<br>2.7E4<br>FAM-NFQ-MGB<br>CT: 24.71	2205 A11 1000m ato3<br>U pGEM<br>2.72E4<br>FAM-NFQ-MGB<br>CT: 24.7	2205 A11 1000m ato3<br>U pGEM<br>2.82E4<br>FAM-NFQ-MGB<br>CT: 24.64		N pGEM<br>FAM-NFQ-MGB<br>CT: Undetermined

Standard Curves

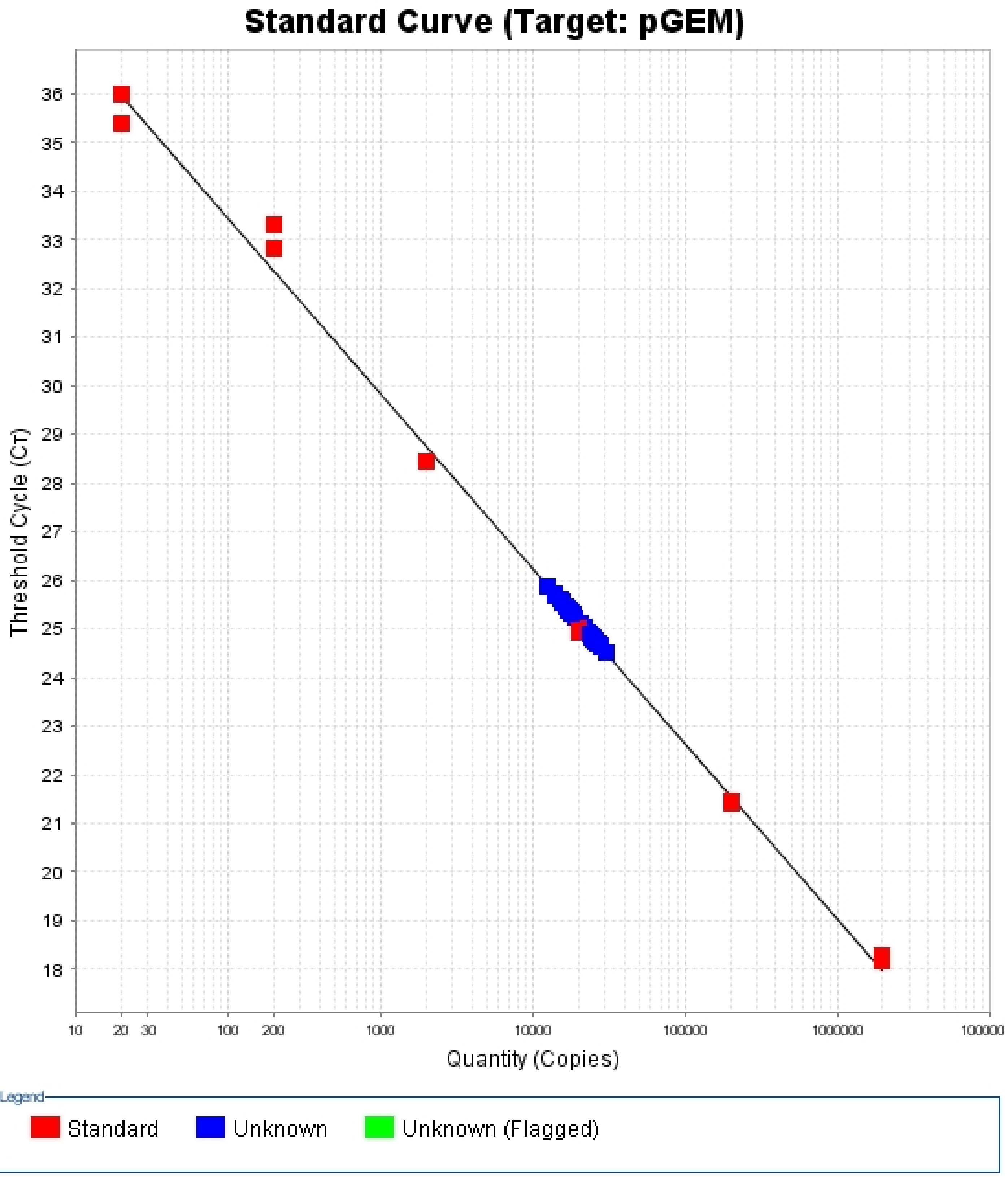

slope:-3.588 Y-Intercept:40.605 R^2:0.996 Eff%:89.983

Results Table

Well		Sample	Target	Task	Quantity	C
A1			pGEM	STANDARD	2000000	18.2948
A2			pGEM	STANDARD	2000000	18.1775
A4		2205 A11 10m	pGEM	UNKNOWN	14171.8799	25.71
A5		2205 A11 10m	pGEM	UNKNOWN	14217.4033	25.705
A6		2205 A11 10m	pGEM	UNKNOWN	15246.3516	25.5961
A7		2205 A11	pGEM	UNKNOWN	12668.6006	25.8847
A8		2205 A11 10m	pGEM	UNKNOWN	15279.1211	25.5928
A9		2205 A11 10m	pGEM	UNKNOWN	17090.6211	25.4182
A10		2205 A11 10m	pGEM	UNKNOWN	16666.1582	25.4574
A11		2205 A11	pGEM	UNKNOWN	17536.582	25.378
B1			pGEM	STANDARD	200000	21.4436
B2			pGEM	STANDARD	200000	21.424
B4		2205 A11 10m	pGEM	UNKNOWN	27182.4141	24.6951
B5		2205 A11 10m	pGEM	UNKNOWN	23706.1426	24.9083
B6		2205 A11 10m	pGEM	UNKNOWN	25928.5137	24.7687
B7		2205 A11	pGEM	UNKNOWN	14078.9619	25.7202
B8		2205 A11 10m	pGEM	UNKNOWN	25804.1758	24.7762
B9		2205 A11 10m	pGEM	UNKNOWN	30339.8906	24.5239
B10		2205 A11 10m	pGEM	UNKNOWN	28650.2188	24.6132
B11		2205 A11	pGEM	UNKNOWN	18275.3145	25.3138
C1			pGEM	STANDARD	20000	24.998
C2			pGEM	STANDARD	20000	24.9219
C4		2205 A11 SCM	pGEM	UNKNOWN	18050.7891	25.333
C5		2205 A11 SCM	pGEM	UNKNOWN	16879.4512	25.4376
C6		2205 A11 SCM	pGEM	UNKNOWN	17580.1484	25.3742
C7		2205 A11	pGEM	UNKNOWN	14268.7998	25.6994
C8		2205 A11 SCM	pGEM	UNKNOWN	15666.9551	25.5537
C9		2205 A11 SCM	pGEM	UNKNOWN	15221.29	25.5987
C10		2205 A11 SCM	pGEM	UNKNOWN	17305.6836	25.3987
C11		2205 A11	pGEM	UNKNOWN	20861.7949	25.1075

Well		Sample	Target	Task	Quantity	C
D1			pGEM	STANDARD	2000	28.4319
D2			pGEM	STANDARD	2000	28.4334
D4		2205 A11 SCM	pGEM	UNKNOWN	28663.8301	24.6124
D5		2205 A11 SCM	pGEM	UNKNOWN	28165.3203	24.6398
D6		2205 A11 SCM	pGEM	UNKNOWN	27017.418	24.7046
D7		2205 A11	pGEM	UNKNOWN	27888.1934	24.6552
D8		2205 A11 SCM	pGEM	UNKNOWN	24871.1035	24.8336
D9		2205 A11 SCM	pGEM	UNKNOWN	25425.082	24.7993
D10		2205 A11 SCM	pGEM	UNKNOWN	28330.0801	24.6307
D11		2205 A11	pGEM	UNKNOWN	26080.1875	24.7596
E1			pGEM	STANDARD	200	32.818
E2			pGEM	STANDARD	200	33.2989
E4		2205 A11 200m	pGEM	UNKNOWN	15700.7822	25.5504
E5		2205 A11 200m	pGEM	UNKNOWN	16954.9727	25.4306
E6		2205 A11 200m	pGEM	UNKNOWN	15263.9424	25.5943
E7		2205 A11	pGEM	UNKNOWN	25651.5	24.7854
E8		2205 A11 200m	pGEM	UNKNOWN	17678.9609	25.3654
E9		2205 A11 200m	pGEM	UNKNOWN	19085.7969	25.2461
E10		2205 A11 200m	pGEM	UNKNOWN	19066.8125	25.2477
E11		2205 A11	pGEM	UNKNOWN	24321.2441	24.8684
F1			pGEM	STANDARD	20	35.9937
F2			pGEM	STANDARD	20	35.3708
F4		2205 A11 200m	pGEM	UNKNOWN	23169.6426	24.944
F5		2205 A11 200m	pGEM	UNKNOWN	24440.8594	24.8608
F6		2205 A11 200m	pGEM	UNKNOWN	24921.6914	24.8304
F7		2205 A11	pGEM	UNKNOWN	27889.457	24.6551
F8		2205 A11 200m	pGEM	UNKNOWN	25464.3887	24.7969
F9		2205 A11 200m	pGEM	UNKNOWN	27335.1328	24.6864
F10		2205 A11 200m	pGEM	UNKNOWN	28243.2754	24.6355
F11		2205 A11	pGEM	UNKNOWN	27168.6094	24.6959
G4		2205 A11	pGEM	UNKNOWN	17013.2676	25.4253
G5		2205 A11	pGEM	UNKNOWN	18442.5547	25.2996

Well		Sample	Target	Task	Quantity	C
G6		2205 A11	pGEM	UNKNOWN	14065.1641	25.7218
G8		2205 A11	pGEM	UNKNOWN	17400.7383	25.3902
G9		2205 A11	pGEM	UNKNOWN	17300.3887	25.3992
G10		2205 A11	pGEM	UNKNOWN	18531.1016	25.2921
G12			pGEM	NTC		Undetermined
H1		Comf2E4	pGEM	UNKNOWN	21747.2832	25.0427
H2		Comf2E4	pGEM	UNKNOWN	24879.0508	24.8331
H4		2205 A11	pGEM	UNKNOWN	26339.3809	24.7442
H5		2205 A11	pGEM	UNKNOWN	24969.1426	24.8275
H6		2205 A11	pGEM	UNKNOWN	25642.3027	24.786
H8		2205 A11	pGEM	UNKNOWN	26973.9961	24.7071
H9		2205 A11	pGEM	UNKNOWN	27160.1641	24.6964
H10		2205 A11	pGEM	UNKNOWN	28178.8047	24.639
H12			pGEM	NTC		Undetermined

QC Summary

Total Wells	96	Processed Wells	76	Targets Used	1
Well Setup	76	Flagged Wells	0	Samples Used	21

Flag	Name	Frequency	Locations
AMPNC	Amplification in negative control	0	
BADROX	Bad passive reference signal	0	
BLFAIL	Baseline algorithm failed	0	
CTFAIL	C algorithm failed	0	
EXPFAIL	Exponential algorithm failed	0	
HIGHSD	High standard deviation in replicate group	0	
NOAMP	No amplification	0	
NOISE	Noise higher than others in plate	0	
NOSIGNAL	No signal in well	0	
OFFSCALE	Fluorescence is offscale	0	
OUTLIERRG	Outlier in replicate group	0	
SPIKE	Noise spikes	0	
THOLDFAIL	Thresholding algorithm failed	0	
