# Supplementary material for: Ecological impact of algal-preying protists aplanochytrids (Labyrinthulea) as a source of docosahexaenoic acid for copepods in marine environments
Source: ISME Commun. 2026 Jun 26;6(1):ycag178. doi: 10.1093/ismeco/ycag178 (PMC13685785; doi:10.1093/ismeco/ycag178)
Supplement: Supplementary file 2 [file supplementary_materials_ycag178.zip › Morimoto_ISME_C_3rdrev_supplemental_data.zip!/Morimoto_ISME_C_3rdrev_supplemental_data/Supplemental_Data_2/Standard Curve/DNA recovery/2205/DnaRec2205AlineA07_N=1.pdf]

Experiment Results Report
230302DnaRecEmmAline2205A07byKurabo

Experiment Summary

Experiment Name	:	230302DnaRecEmmAline2205A07byKurabo
Experiment Type	:	Quantitation - Standard Curve
File Name	:	230302DnaRecEmmAline2205A07byKurabo.eds
Run Started	:	2023 Mar 03 11:57:03 AM
Run Finished	:	2023 Mar 03 1:31:39 PM
Run Duration	:	94 minutes 35 seconds
Date Modified	:	2023 Mar 03 1:38:23 PM
User	:	
Number of wells used	:	46
Number of wells with results	:	46
Instrument Name	:	
Instrument Type	:	Applied Biosystems StepOnePlus™ Instrument
Comments	:	

Results Summary

Sample	Target	Quantity (Mean)	Quantity (Std Dev)	C (Mean)	C (Std Dev)
2205 A07 1000m ato	pGEM	19974.69	1123.87	25.25	0.08
2205 A07 1000m	pGEM	12635.14	821.87	25.93	0.10
2205 A07 10m ato	pGEM	20474.03	1792.75	25.21	0.13
2205 A07 10m saki	pGEM	9216.33	694.43	26.41	0.11
2205 A07 200m ato	pGEM	22531.72	1962.40	25.07	0.13
2205 A07 200m saki	pGEM	5603.48	139.23	27.15	0.04
2205 A07 3000m ato	pGEM	18449.15	1076.70	25.37	0.09
2205 A07 3000m	pGEM	11626.25	430.49	26.06	0.05
2205 A07 SCM ato	pGEM	19941.28	1087.03	25.25	0.08
2205 A07 SCM saki	pGEM	8168.61	636.70	26.59	0.12
Comf2E4	pGEM	19529.55	3240.36	25.29	0.25

Plate Layout

	1	2	3	4	5	6	7	8	9	10	11	12
A	S pGEM<br>2E6<br>FAM-NFQ-MGB<br>CT: 18.73	S pGEM<br>2E6<br>FAM-NFQ-MGB<br>CT: 18.41		2205 A07 10m saki<br>U pGEM<br>8.47E3<br>FAM-NFQ-MGB<br>CT: 26.53	2205 A07 10m saki<br>U pGEM<br>9.34E3<br>FAM-NFQ-MGB<br>CT: 26.38	2205 A07 10m saki<br>U pGEM<br>9.84E3<br>FAM-NFQ-MGB<br>CT: 26.31		2205 A07 3000m saki<br>U pGEM<br>1.15E4<br>FAM-NFQ-MGB<br>CT: 26.08	2205 A07 3000m saki<br>U pGEM<br>1.21E4<br>FAM-NFQ-MGB<br>CT: 26	2205 A07 3000m saki<br>U pGEM<br>1.13E4<br>FAM-NFQ-MGB<br>CT: 26.1		
B	S pGEM<br>2E5<br>FAM-NFQ-MGB<br>CT: 21.69	S pGEM<br>2E5<br>FAM-NFQ-MGB<br>CT: 21.63		2205 A07 10m ato<br>U pGEM<br>2.01E4<br>FAM-NFQ-MGB<br>CT: 25.23	2205 A07 10m ato<br>U pGEM<br>1.89E4<br>FAM-NFQ-MGB<br>CT: 25.33	2205 A07 10m ato<br>U pGEM<br>2.24E4<br>FAM-NFQ-MGB<br>CT: 25.07		2205 A07 3000m ato<br>U pGEM<br>1.92E4<br>FAM-NFQ-MGB<br>CT: 25.31	2205 A07 3000m ato<br>U pGEM<br>1.72E4<br>FAM-NFQ-MGB<br>CT: 25.47	2205 A07 3000m ato<br>U pGEM<br>1.9E4<br>FAM-NFQ-MGB<br>CT: 25.32		
C	S pGEM<br>2E4<br>FAM-NFQ-MGB<br>CT: 25.16	S pGEM<br>2E4<br>FAM-NFQ-MGB<br>CT: 25.04		2205 A07 SCM saki<br>U pGEM<br>8.63E3<br>FAM-NFQ-MGB<br>CT: 26.5	2205 A07 SCM saki<br>U pGEM<br>8.43E3<br>FAM-NFQ-MGB<br>CT: 26.54	2205 A07 SCM saki<br>U pGEM<br>7.44E3<br>FAM-NFQ-MGB<br>CT: 26.72						
D	S pGEM<br>2E3<br>FAM-NFQ-MGB<br>CT: 28.62	S pGEM<br>2E3<br>FAM-NFQ-MGB<br>CT: 28.69		2205 A07 SCM ato<br>U pGEM<br>1.87E4<br>FAM-NFQ-MGB<br>CT: 25.34	2205 A07 SCM ato<br>U pGEM<br>2.08E4<br>FAM-NFQ-MGB<br>CT: 25.18	2205 A07 SCM ato<br>U pGEM<br>2.03E4<br>FAM-NFQ-MGB<br>CT: 25.23						
E	S pGEM<br>200<br>FAM-NFQ-MGB<br>CT: 32.12	S pGEM<br>200<br>FAM-NFQ-MGB<br>CT: 32.07		2205 A07 200m saki<br>U pGEM<br>5.75E3<br>FAM-NFQ-MGB<br>CT: 27.11	2205 A07 200m saki<br>U pGEM<br>5.47E3<br>FAM-NFQ-MGB<br>CT: 27.18	2205 A07 200m saki<br>U pGEM<br>5.59E3<br>FAM-NFQ-MGB<br>CT: 27.15						
F	S pGEM<br>20<br>FAM-NFQ-MGB<br>CT: 35.97	S pGEM<br>20<br>FAM-NFQ-MGB<br>CT: 35.47		2205 A07 200m ato<br>U pGEM<br>2.03E4<br>FAM-NFQ-MGB<br>CT: 25.22	2205 A07 200m ato<br>U pGEM<br>2.35E4<br>FAM-NFQ-MGB<br>CT: 25	2205 A07 200m ato<br>U pGEM<br>2.38E4<br>FAM-NFQ-MGB<br>CT: 24.98						
G				2205 A07 1000m saki<br>U pGEM<br>1.28E4<br>FAM-NFQ-MGB<br>CT: 25.91	2205 A07 1000m saki<br>U pGEM<br>1.34E4<br>FAM-NFQ-MGB<br>CT: 25.85	2205 A07 1000m saki<br>U pGEM<br>1.17E4<br>FAM-NFQ-MGB<br>CT: 26.04						
H	Comf2E4<br>U pGEM<br>1.72E4<br>FAM-NFQ-MGB<br>CT: 25.47	Comf2E4<br>U pGEM<br>2.18E4<br>FAM-NFQ-MGB<br>CT: 25.11		2205 A07 1000m ato<br>U pGEM<br>2.1E4<br>FAM-NFQ-MGB<br>CT: 25.17	2205 A07 1000m ato<br>U pGEM<br>2.01E4<br>FAM-NFQ-MGB<br>CT: 25.23	2205 A07 1000m ato<br>U pGEM<br>1.88E4<br>FAM-NFQ-MGB<br>CT: 25.34					N pGEM<br>FAM-NFQ-MGB<br>CT: Undetermined	N pGEM<br>FAM-NFQ-MGB<br>CT: Undetermined

Standard Curves

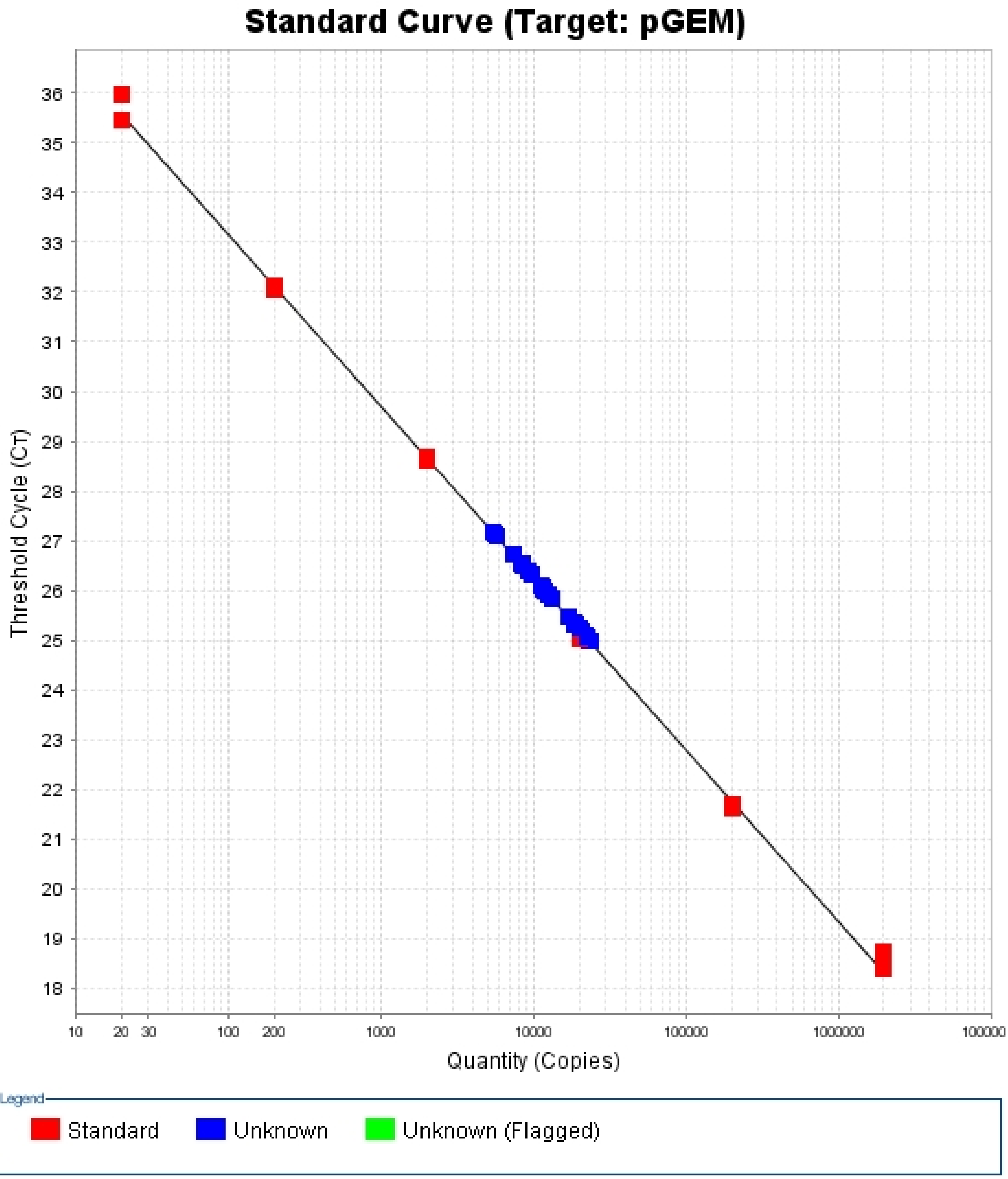

slope:-3.446 Y-Intercept:40.068 R^2:0.999 Eff%:95.055

Results Table

Well		Sample	Target	Task	Quantity	C
A1			pGEM	STANDARD	2000000	18.7253
A2			pGEM	STANDARD	2000000	18.4139
A4		2205 A07 10m	pGEM	UNKNOWN	8469.7686	26.5307
A5		2205 A07 10m	pGEM	UNKNOWN	9336.2051	26.3849
A6		2205 A07 10m	pGEM	UNKNOWN	9843.0205	26.3058
A8		2205 A07	pGEM	UNKNOWN	11477.415	26.0759
A9		2205 A07	pGEM	UNKNOWN	12111.4043	25.9954
A10		2205 A07	pGEM	UNKNOWN	11289.9229	26.1005
B1			pGEM	STANDARD	200000	21.6904
B2			pGEM	STANDARD	200000	21.6334
B4		2205 A07 10m	pGEM	UNKNOWN	20142.5078	25.234
B5		2205 A07 10m	pGEM	UNKNOWN	18870.1855	25.3317
B6		2205 A07 10m	pGEM	UNKNOWN	22409.4023	25.0744
B8		2205 A07	pGEM	UNKNOWN	19155.3242	25.3092
B9		2205 A07	pGEM	UNKNOWN	17209.9062	25.4695
B10		2205 A07	pGEM	UNKNOWN	18982.2129	25.3228
C1			pGEM	STANDARD	20000	25.1643
C2			pGEM	STANDARD	20000	25.0408
C4		2205 A07 SCM	pGEM	UNKNOWN	8634.8525	26.5018
C5		2205 A07 SCM	pGEM	UNKNOWN	8427.79	26.5381
C6		2205 A07 SCM	pGEM	UNKNOWN	7443.1987	26.7241
D1			pGEM	STANDARD	2000	28.617
D2			pGEM	STANDARD	2000	28.6875
D4		2205 A07 SCM	pGEM	UNKNOWN	18730.9902	25.3427
D5		2205 A07 SCM	pGEM	UNKNOWN	20834.5664	25.1834
D6		2205 A07 SCM	pGEM	UNKNOWN	20258.2949	25.2254
E1			pGEM	STANDARD	200	32.1249
E2			pGEM	STANDARD	200	32.0698
E4		2205 A07 200m	pGEM	UNKNOWN	5748.1265	27.1109
E5		2205 A07 200m	pGEM	UNKNOWN	5470.3901	27.185

Well		Sample	Target	Task	Quantity	C
E6		2205 A07 200m	pGEM	UNKNOWN	5591.9224	27.1521
F1			pGEM	STANDARD	20	35.9744
F2			pGEM	STANDARD	20	35.4721
F4		2205 A07 200m	pGEM	UNKNOWN	20271.0508	25.2245
F5		2205 A07 200m	pGEM	UNKNOWN	23527.7129	25.0015
F6		2205 A07 200m	pGEM	UNKNOWN	23796.4043	24.9845
G4		2205 A07	pGEM	UNKNOWN	12789.3574	25.9139
G5		2205 A07	pGEM	UNKNOWN	13368.9785	25.8475
G6		2205 A07	pGEM	UNKNOWN	11747.0928	26.0411
H1		Comf2E4	pGEM	UNKNOWN	17238.2656	25.467
H2		Comf2E4	pGEM	UNKNOWN	21820.832	25.1142
H4		2205 A07	pGEM	UNKNOWN	21001.8633	25.1715
H5		2205 A07	pGEM	UNKNOWN	20147.9512	25.2336
H6		2205 A07	pGEM	UNKNOWN	18774.2422	25.3393
H11			pGEM	NTC		Undetermined
H12			pGEM	NTC		Undetermined

QC Summary

Total Wells	96	Processed Wells	46	Targets Used	1
Well Setup	46	Flagged Wells	0	Samples Used	11

Flag	Name	Frequency	Locations
AMPNC	Amplification in negative control	0	
BADROX	Bad passive reference signal	0	
BLFAIL	Baseline algorithm failed	0	
CTFAIL	C algorithm failed	0	
EXPFAIL	Exponential algorithm failed	0	
HIGHSD	High standard deviation in replicate group	0	
NOAMP	No amplification	0	
NOISE	Noise higher than others in plate	0	
NOSIGNAL	No signal in well	0	
OFFSCALE	Fluorescence is offscale	0	
OUTLIERRG	Outlier in replicate group	0	
SPIKE	Noise spikes	0	
THOLDFAIL	Thresholding algorithm failed	0	
