# Supplementary material for: Ecological impact of algal-preying protists aplanochytrids (Labyrinthulea) as a source of docosahexaenoic acid for copepods in marine environments
Source: ISME Commun. 2026 Jun 26;6(1):ycag178. doi: 10.1093/ismeco/ycag178 (PMC13685785; doi:10.1093/ismeco/ycag178)
Supplement: Supplementary file 2 [file supplementary_materials_ycag178.zip › Morimoto_ISME_C_3rdrev_supplemental_data.zip!/Morimoto_ISME_C_3rdrev_supplemental_data/Supplemental_Data_2/Standard Curve/DNA recovery/2205/DnaRec2205AlineA21_N=1.pdf]

# Experiment Results Report
## 230302DnaRecEmmAlineA21byKurabo

## Experiment Summary

| | | |
|---|---|---|
| Experiment Name | : | 230302DnaRecEmmAlineA21byKurabo |
| Experiment Type | : | Quantitation - Standard Curve |
| File Name | : | 230302DnaRecEmmAlineA21byKurabo.eds |
| Run Started | : | 2023 Mar 03 4:31:40 PM |
| Run Finished | : | 2023 Mar 03 6:06:16 PM |
| Run Duration | : | 94 minutes 35 seconds |
| Date Modified | : | 2023 Mar 03 6:30:55 PM |
| User | : | |
| Number of wells used | : | 46 |
| Number of wells with results | : | 46 |
| Instrument Name | : | |
| Instrument Type | : | Applied Biosystems StepOnePlus™ Instrument |
| Comments | : | |

# Results Summary

| Sample | Target | Quantity (Mean) | Quantity (Std Dev) | C (Mean) | C (Std Dev) |
|---|---|---|---|---|---|
| 2205 A21 1000m ato | pGEM | 21441.72 | 1177.95 | 25.36 | 0.08 |
| 2205 A21 1000m | pGEM | 5630.33 | 402.73 | 27.37 | 0.11 |
| 2205 A21 10m ato | pGEM | 20397.86 | 1200.29 | 25.43 | 0.09 |
| 2205 A21 10m saki | pGEM | 7686.95 | 104.05 | 26.90 | 0.02 |
| 2205 A21 200m ato | pGEM | 18904.66 | 1029.32 | 25.55 | 0.08 |
| 2205 A21 200m saki | pGEM | 7247.22 | 148.83 | 26.98 | 0.03 |
| 2205 A21 3000m ato | pGEM | 18164.79 | 1260.25 | 25.61 | 0.10 |
| 2205 A21 3000m | pGEM | 3749.88 | 207.37 | 27.98 | 0.08 |
| 2205 A21 SCM ato | pGEM | 20766.40 | 1274.75 | 25.41 | 0.09 |
| 2205 A21 SCM saki | pGEM | 6700.30 | 479.46 | 27.10 | 0.11 |
| comf2E4 | pGEM | 20408.07 | 3832.02 | 25.44 | 0.28 |

# Plate Layout

| | 1 | 2 | 3 | 4 | 5 | 6 | 7 | 8 | 9 | 10 | 11 | 12 |
|---|---|---|---|---|---|---|---|---|---|---|---|---|
| A | S pGEM<br>2E6<br>FAM-NFQ-MGB<br>CT: 18.79 | S pGEM<br>2E6<br>FAM-NFQ-MGB<br>CT: 18.65 | | 2205 A21 10m saki<br>U pGEM<br>7.58E3<br>FAM-NFQ-MGB<br>CT: 26.92 | 2205 A21 10m saki<br>U pGEM<br>7.7E3<br>FAM-NFQ-MGB<br>CT: 26.89 | 2205 A21 10m saki<br>U pGEM<br>7.79E3<br>FAM-NFQ-MGB<br>CT: 26.88 | | 2205 A21 3000m saki<br>U pGEM<br>3.56E3<br>FAM-NFQ-MGB<br>CT: 28.05 | 2205 A21 3000m saki<br>U pGEM<br>3.97E3<br>FAM-NFQ-MGB<br>CT: 27.89 | 2205 A21 3000m saki<br>U pGEM<br>3.72E3<br>FAM-NFQ-MGB<br>CT: 27.99 | | |
| B | S pGEM<br>2E5<br>FAM-NFQ-MGB<br>CT: 21.81 | S pGEM<br>2E5<br>FAM-NFQ-MGB<br>CT: 21.86 | | 2205 A21 10m ato<br>U pGEM<br>1.95E4<br>FAM-NFQ-MGB<br>CT: 25.5 | 2205 A21 10m ato<br>U pGEM<br>1.99E4<br>FAM-NFQ-MGB<br>CT: 25.46 | 2205 A21 10m ato<br>U pGEM<br>2.18E4<br>FAM-NFQ-MGB<br>CT: 25.33 | | 2205 A21 3000m ato<br>U pGEM<br>1.95E4<br>FAM-NFQ-MGB<br>CT: 25.5 | 2205 A21 3000m ato<br>U pGEM<br>1.79E4<br>FAM-NFQ-MGB<br>CT: 25.63 | 2205 A21 3000m ato<br>U pGEM<br>1.71E4<br>FAM-NFQ-MGB<br>CT: 25.7 | | |
| C | S pGEM<br>2E4<br>FAM-NFQ-MGB<br>CT: 25.34 | S pGEM<br>2E4<br>FAM-NFQ-MGB<br>CT: 25.31 | | 2205 A21 SCM saki<br>U pGEM<br>6.89E3<br>FAM-NFQ-MGB<br>CT: 27.06 | 2205 A21 SCM saki<br>U pGEM<br>7.06E3<br>FAM-NFQ-MGB<br>CT: 27.02 | 2205 A21 SCM saki<br>U pGEM<br>6.16E3<br>FAM-NFQ-MGB<br>CT: 27.23 | | | | | | |
| D | S pGEM<br>2E3<br>FAM-NFQ-MGB<br>CT: 28.89 | S pGEM<br>2E3<br>FAM-NFQ-MGB<br>CT: 28.89 | | 2205 A21 SCM ato<br>U pGEM<br>1.93E4<br>FAM-NFQ-MGB<br>CT: 25.51 | 2205 A21 SCM ato<br>U pGEM<br>2.14E4<br>FAM-NFQ-MGB<br>CT: 25.36 | 2205 A21 SCM ato<br>U pGEM<br>2.16E4<br>FAM-NFQ-MGB<br>CT: 25.35 | | | | | | |
| E | S pGEM<br>200<br>FAM-NFQ-MGB<br>CT: 32.62 | S pGEM<br>200<br>FAM-NFQ-MGB<br>CT: 32.65 | | 2205 A21 200m saki<br>U pGEM<br>7.42E3<br>FAM-NFQ-MGB<br>CT: 26.95 | 2205 A21 200m saki<br>U pGEM<br>7.18E3<br>FAM-NFQ-MGB<br>CT: 27 | 2205 A21 200m saki<br>U pGEM<br>7.15E3<br>FAM-NFQ-MGB<br>CT: 27 | | | | | | |
| F | S pGEM<br>20<br>FAM-NFQ-MGB<br>CT: 35.48 | S pGEM<br>20<br>FAM-NFQ-MGB<br>CT: 35.97 | | 2205 A21 200m ato<br>U pGEM<br>1.79E4<br>FAM-NFQ-MGB<br>CT: 25.63 | 2205 A21 200m ato<br>U pGEM<br>1.89E4<br>FAM-NFQ-MGB<br>CT: 25.55 | 2205 A21 200m ato<br>U pGEM<br>1.99E4<br>FAM-NFQ-MGB<br>CT: 25.46 | | | | | | |
| G | | | | 2205 A21 1000m saki<br>U pGEM<br>5.84E3<br>FAM-NFQ-MGB<br>CT: 27.31 | 2205 A21 1000m saki<br>U pGEM<br>5.88E3<br>FAM-NFQ-MGB<br>CT: 27.3 | 2205 A21 1000m saki<br>U pGEM<br>5.17E3<br>FAM-NFQ-MGB<br>CT: 27.49 | | | | | | |
| H | comf2E4<br>U pGEM<br>1.77E4<br>FAM-NFQ-MGB<br>CT: 25.64 | comf2E4<br>U pGEM<br>2.31E4<br>FAM-NFQ-MGB<br>CT: 25.24 | | 2205 A21 1000m ato<br>U pGEM<br>2.03E4<br>FAM-NFQ-MGB<br>CT: 25.43 | 2205 A21 1000m ato<br>U pGEM<br>2.27E4<br>FAM-NFQ-MGB<br>CT: 25.27 | 2205 A21 1000m ato<br>U pGEM<br>2.13E4<br>FAM-NFQ-MGB<br>CT: 25.37 | | | | | N pGEM<br>FAM-NFQ-MGB<br>CT: Undetermined | N pGEM<br>FAM-NFQ-MGB<br>CT: Undetermined |

# Standard Curves

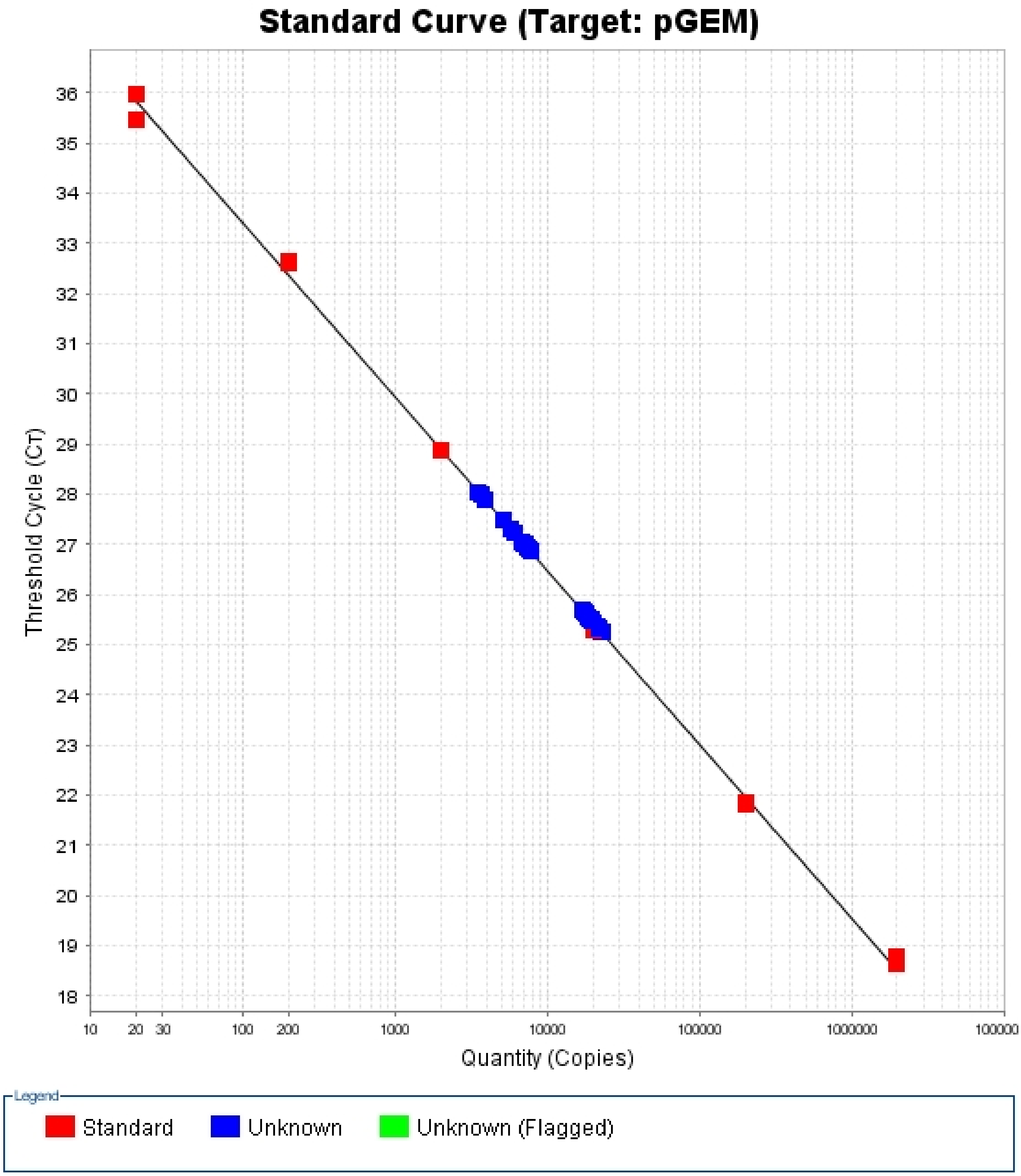


slope:-3.458 Y-Intercept:40.331 $R^2$:0.999 Eff%:94.63

# Results Table

| Well | | Sample | Target | Task | Quantity | C |
|---|---|---|---|---|---|---|
| A1 | | | pGEM | STANDARD | 2000000 | 18.7851 |
| A2 | | | pGEM | STANDARD | 2000000 | 18.6458 |
| A4 | | 2205 A21 10m | pGEM | UNKNOWN | 7577.9102 | 26.9171 |
| A5 | | 2205 A21 10m | pGEM | UNKNOWN | 7697.7671 | 26.8935 |
| A6 | | 2205 A21 10m | pGEM | UNKNOWN | 7785.1611 | 26.8766 |
| A8 | | 2205 A21 | pGEM | UNKNOWN | 3560.1208 | 28.0515 |
| A9 | | 2205 A21 | pGEM | UNKNOWN | 3971.2327 | 27.8874 |
| A10 | | 2205 A21 | pGEM | UNKNOWN | 3718.2827 | 27.9862 |
| B1 | | | pGEM | STANDARD | 200000 | 21.8109 |
| B2 | | | pGEM | STANDARD | 200000 | 21.8648 |
| B4 | | 2205 A21 10m | pGEM | UNKNOWN | 19487.3711 | 25.4987 |
| B5 | | 2205 A21 10m | pGEM | UNKNOWN | 19948.1484 | 25.4636 |
| B6 | | 2205 A21 10m | pGEM | UNKNOWN | 21758.0703 | 25.3332 |
| B8 | | 2205 A21 | pGEM | UNKNOWN | 19532.9434 | 25.4952 |
| B9 | | 2205 A21 | pGEM | UNKNOWN | 17910.0723 | 25.6255 |
| B10 | | 2205 A21 | pGEM | UNKNOWN | 17051.3535 | 25.6992 |
| C1 | | | pGEM | STANDARD | 20000 | 25.3422 |
| C2 | | | pGEM | STANDARD | 20000 | 25.3058 |
| C4 | | 2205 A21 SCM | pGEM | UNKNOWN | 6886.918 | 27.0607 |
| C5 | | 2205 A21 SCM | pGEM | UNKNOWN | 7058.3955 | 27.0237 |
| C6 | | 2205 A21 SCM | pGEM | UNKNOWN | 6155.5972 | 27.2292 |
| D1 | | | pGEM | STANDARD | 2000 | 28.8893 |
| D2 | | | pGEM | STANDARD | 2000 | 28.8885 |
| D4 | | 2205 A21 SCM | pGEM | UNKNOWN | 19297.2676 | 25.5134 |
| D5 | | 2205 A21 SCM | pGEM | UNKNOWN | 21422.0664 | 25.3566 |
| D6 | | 2205 A21 SCM | pGEM | UNKNOWN | 21579.8594 | 25.3456 |
| E1 | | | pGEM | STANDARD | 200 | 32.6229 |
| E2 | | | pGEM | STANDARD | 200 | 32.6544 |
| E4 | | 2205 A21 200m | pGEM | UNKNOWN | 7418.2412 | 26.9491 |
| E5 | | 2205 A21 200m | pGEM | UNKNOWN | 7176.4131 | 26.9988 |

| Well | | Sample | Target | Task | Quantity | C |
|---|---|---|---|---|---|---|
| E6 | | 2205 A21 200m | pGEM | UNKNOWN | 7147.0132 | 27.005 |
| F1 | | | pGEM | STANDARD | 20 | 35.4781 |
| F2 | | | pGEM | STANDARD | 20 | 35.9743 |
| F4 | | 2205 A21 200m | pGEM | UNKNOWN | 17891.5645 | 25.627 |
| F5 | | 2205 A21 200m | pGEM | UNKNOWN | 18872.9434 | 25.5468 |
| F6 | | 2205 A21 200m | pGEM | UNKNOWN | 19949.4648 | 25.4635 |
| G4 | | 2205 A21 | pGEM | UNKNOWN | 5842.3262 | 27.3077 |
| G5 | | 2205 A21 | pGEM | UNKNOWN | 5882.7896 | 27.2973 |
| G6 | | 2205 A21 | pGEM | UNKNOWN | 5165.8823 | 27.4925 |
| H1 | | comf2E4 | pGEM | UNKNOWN | 17698.4277 | 25.6433 |
| H2 | | comf2E4 | pGEM | UNKNOWN | 23117.7188 | 25.2422 |
| H4 | | 2205 A21 | pGEM | UNKNOWN | 20348.541 | 25.4338 |
| H5 | | 2205 A21 | pGEM | UNKNOWN | 22689.2188 | 25.2703 |
| H6 | | 2205 A21 | pGEM | UNKNOWN | 21287.3965 | 25.3661 |
| H11 | | | pGEM | NTC | | Undetermined |
| H12 | | | pGEM | NTC | | Undetermined |

# QC Summary

| | | | | | |
|---|---|---|---|---|---|
| Total Wells | 96 | Processed Wells | 46 | Targets Used | 1 |
| Well Setup | 46 | Flagged Wells | 0 | Samples Used | 11 |

| Flag | Name | Frequency | Locations |
|---|---|---|---|
| AMPNC | Amplification in negative control | 0 | |
| BADROX | Bad passive reference signal | 0 | |
| BLFAIL | Baseline algorithm failed | 0 | |
| CTFAIL | C algorithm failed | 0 | |
| EXPFAIL | Exponential algorithm failed | 0 | |
| HIGHSD | High standard deviation in replicate group | 0 | |
| NOAMP | No amplification | 0 | |
| NOISE | Noise higher than others in plate | 0 | |
| NOSIGNAL | No signal in well | 0 | |
| OFFSCALE | Fluorescence is offscale | 0 | |
| OUTLIERRG | Outlier in replicate group | 0 | |
| SPIKE | Noise spikes | 0 | |
| THOLDFAIL | Thresholding algorithm failed | 0 | |
