# Supplementary material for: Ecological impact of algal-preying protists aplanochytrids (Labyrinthulea) as a source of docosahexaenoic acid for copepods in marine environments
Source: ISME Commun. 2026 Jun 26;6(1):ycag178. doi: 10.1093/ismeco/ycag178 (PMC13685785; doi:10.1093/ismeco/ycag178)
Supplement: Supplementary file 2 [file supplementary_materials_ycag178.zip › Morimoto_ISME_C_3rdrev_supplemental_data.zip!/Morimoto_ISME_C_3rdrev_supplemental_data/Supplemental_Data_2/Standard Curve/DNA recovery/2205/DnaRecAline2205_A01_N=2,3.pdf]

Experiment Results Report

230322DnaRec&AplanoAlineDnaExtN=3EmmByKurabo

Experiment Summary

Experiment Name	:	230322DnaRec&AplanoAlineDnaExtN=3EmmByKurabo
Experiment Type	:	Quantitation - Standard Curve
File Name	:	230322DnaRec&AplanoAlineDnaExtN=3EmmByKurabo.eds
Run Started	:	2023 Mar 22 2:34:30 PM
Run Finished	:	2023 Mar 22 4:09:12 PM
Run Duration	:	94 minutes 41 seconds
Date Modified	:	2023 Mar 22 4:27:34 PM
User	:	
Number of wells used	:	62
Number of wells with results	:	62
Instrument Name	:	
Instrument Type	:	Applied Biosystems StepOnePlus™ Instrument
Comments	:	

Results Summary

Sample	Target	Quantity (Mean)	Quantity (Std Dev)	C (Mean)	C (Std Dev)
2205 A01 10m saki2	Aplano	21.62	5.47	32.65	0.39
2205 A01 10m saki3	Aplano	8.68	7.43	34.30	1.47
2205 A01 BTM saki2	Aplano	9.76	6.72	33.99	1.12
2205 A01 BTM saki3	Aplano	0.20		39.57	
2205 A01 SCM saki2	Aplano	2.29	1.36	36.12	0.87
2205 A01 SCM saki3	Aplano	18.79	7.64	32.90	0.61
Comf2E4	Aplano	17564.96	838.98	22.67	0.07
2205 A01 SCM ato3	pGEM	19212.95	2306.00	22.06	0.18
2205 A01 SCM saki3	pGEM	14661.02	1013.23	22.45	0.10
2205 A04 3000m	pGEM	15452.67	191.78	22.37	0.02
2205A04 3000m ato3	pGEM	4383.41	317.42	24.24	0.11
Comf2E4	pGEM	18760.51	1086.07	22.09	0.09

Plate Layout

	1	2	3	4	5	6	7	8	9	10	11	12
A	S pGEM<br>2E6<br>FAM-NFQ-MGB<br>CT: 15.65	S pGEM<br>2E6<br>FAM-NFQ-MGB<br>CT: 15.34	2205 A01 SCM saki3<br>U pGEM<br>1.58E4<br>FAM-NFQ-MGB<br>CT: 22.34	2205 A01 SCM saki3<br>U pGEM<br>1.4E4<br>FAM-NFQ-MGB<br>CT: 22.52	2205 A01 SCM saki3<br>U pGEM<br>1.42E4<br>FAM-NFQ-MGB<br>CT: 22.5		S Aplano<br>2E6<br>FAM-NFQ-MGB<br>CT: 15.89	S Aplano<br>2E6<br>FAM-NFQ-MGB<br>CT: 16.11	2205 A01 10m saki2<br>U Aplano<br>27.06<br>FAM-NFQ-MGB<br>CT: 32.28	2205 A01 10m saki2<br>U Aplano<br>21.69<br>FAM-NFQ-MGB<br>CT: 32.61	2205 A01 10m saki2<br>U Aplano<br>16.11<br>FAM-NFQ-MGB<br>CT: 33.05	
B	S pGEM<br>2E5<br>FAM-NFQ-MGB<br>CT: 18.51	S pGEM<br>2E5<br>FAM-NFQ-MGB<br>CT: 18.32	2205 A01 SCM ato3<br>U pGEM<br>1.66E4<br>FAM-NFQ-MGB<br>CT: 22.27	2205 A01 SCM ato3<br>U pGEM<br>2.06E4<br>FAM-NFQ-MGB<br>CT: 21.95	2205 A01 SCM ato3<br>U pGEM<br>2.05E4<br>FAM-NFQ-MGB<br>CT: 21.96		S Aplano<br>2E5<br>FAM-NFQ-MGB<br>CT: 19.29	S Aplano<br>2E5<br>FAM-NFQ-MGB<br>CT: 18.96	⚠ 1<br>2205 A01 SCM saki2<br>U Aplano<br>3.81<br>FAM-NFQ-MGB<br>CT: 35.19	⚠ 1<br>2205 A01 SCM saki2<br>U Aplano<br>1.87<br>FAM-NFQ-MGB<br>CT: 36.25	⚠ 1<br>2205 A01 SCM saki2<br>U Aplano<br>1.18<br>FAM-NFQ-MGB<br>CT: 36.92	
C	S pGEM<br>2E4<br>FAM-NFQ-MGB<br>CT: 21.72	S pGEM<br>2E4<br>FAM-NFQ-MGB<br>CT: 21.8	2205 A04 3000m saki3<br>U pGEM<br>1.57E4<br>FAM-NFQ-MGB<br>CT: 22.35	2205 A04 3000m saki3<br>U pGEM<br>1.53E4<br>FAM-NFQ-MGB<br>CT: 22.39	2205 A04 3000m saki3<br>U pGEM<br>1.54E4<br>FAM-NFQ-MGB<br>CT: 22.38		S Aplano<br>2E4<br>FAM-NFQ-MGB<br>CT: 22.35	S Aplano<br>2E4<br>FAM-NFQ-MGB<br>CT: 22.43	⚠ 2<br>2205 A01 BTM saki2<br>U Aplano<br>FAM-NFQ-MGB<br>CT: Undetermined	⚠ 1<br>2205 A01 BTM saki2<br>U Aplano<br>5<br>FAM-NFQ-MGB<br>CT: 34.79	⚠ 1<br>2205 A01 BTM saki2<br>U Aplano<br>14.51<br>FAM-NFQ-MGB<br>CT: 33.2	
D	S pGEM<br>2E3<br>FAM-NFQ-MGB<br>CT: 25.27	S pGEM<br>2E3<br>FAM-NFQ-MGB<br>CT: 25.34	2205A04 3000m ato3<br>U pGEM<br>4.03E3<br>FAM-NFQ-MGB<br>CT: 24.36	2205A04 3000m ato3<br>U pGEM<br>4.65E3<br>FAM-NFQ-MGB<br>CT: 24.14	2205A04 3000m ato3<br>U pGEM<br>4.46E3<br>FAM-NFQ-MGB<br>CT: 24.21		⚠ 1<br>S Aplano<br>2E3<br>FAM-NFQ-MGB<br>CT: 24.29	⚠ 1<br>S Aplano<br>2E3<br>FAM-NFQ-MGB<br>CT: 25.61	⚠ 1<br>2205 A01 10m saki3<br>U Aplano<br>13.94<br>FAM-NFQ-MGB<br>CT: 33.26	⚠ 2<br>2205 A01 10m saki3<br>U Aplano<br>FAM-NFQ-MGB<br>CT: Undetermined	⚠ 1<br>2205 A01 10m saki3<br>U Aplano<br>3.43<br>FAM-NFQ-MGB<br>CT: 35.34	
E	S pGEM<br>200<br>FAM-NFQ-MGB<br>CT: 28.86	S pGEM<br>200<br>FAM-NFQ-MGB<br>CT: 28.87					S Aplano<br>200<br>FAM-NFQ-MGB<br>CT: 29.16	S Aplano<br>200<br>FAM-NFQ-MGB<br>CT: 29.59	⚠ 1<br>2205 A01 SCM saki3<br>U Aplano<br>17.55<br>FAM-NFQ-MGB<br>CT: 32.92	⚠ 1<br>2205 A01 SCM saki3<br>U Aplano<br>11.84<br>FAM-NFQ-MGB<br>CT: 33.51	⚠ 1<br>2205 A01 SCM saki3<br>U Aplano<br>26.98<br>FAM-NFQ-MGB<br>CT: 32.28	
F	S pGEM<br>20<br>FAM-NFQ-MGB<br>CT: 32.37	S pGEM<br>20<br>FAM-NFQ-MGB<br>CT: 32.26					⚠ 1<br>S Aplano<br>20<br>FAM-NFQ-MGB<br>CT: 32.37	⚠ 1<br>S Aplano<br>20<br>FAM-NFQ-MGB<br>CT: 34.15	⚠ 1<br>2205 A01 BTM saki3<br>U Aplano<br>FAM-NFQ-MGB<br>CT: Undetermined	2205 A01 BTM saki3<br>U Aplano<br>0.2<br>FAM-NFQ-MGB<br>CT: 39.57	⚠ 2<br>2205 A01 BTM saki3<br>U Aplano<br>FAM-NFQ-MGB<br>CT: Undetermined	
G												
H	Comf2E4<br>U pGEM<br>1.8E4<br>FAM-NFQ-MGB<br>CT: 22.15	Comf2E4<br>U pGEM<br>1.95E4<br>FAM-NFQ-MGB<br>CT: 22.03		⚠ 1<br>N pGEM<br>FAM-NFQ-MGB<br>CT: 36.96	⚠ 2<br>N pGEM<br>FAM-NFQ-MGB<br>CT: 32.68		Comf2E4<br>U Aplano<br>1.82E4<br>FAM-NFQ-MGB<br>CT: 22.62	Comf2E4<br>U Aplano<br>1.7E4<br>FAM-NFQ-MGB<br>CT: 22.72		N Aplano<br>FAM-NFQ-MGB<br>CT: Undetermined	N Aplano<br>FAM-NFQ-MGB<br>CT: Undetermined	

Standard Curves

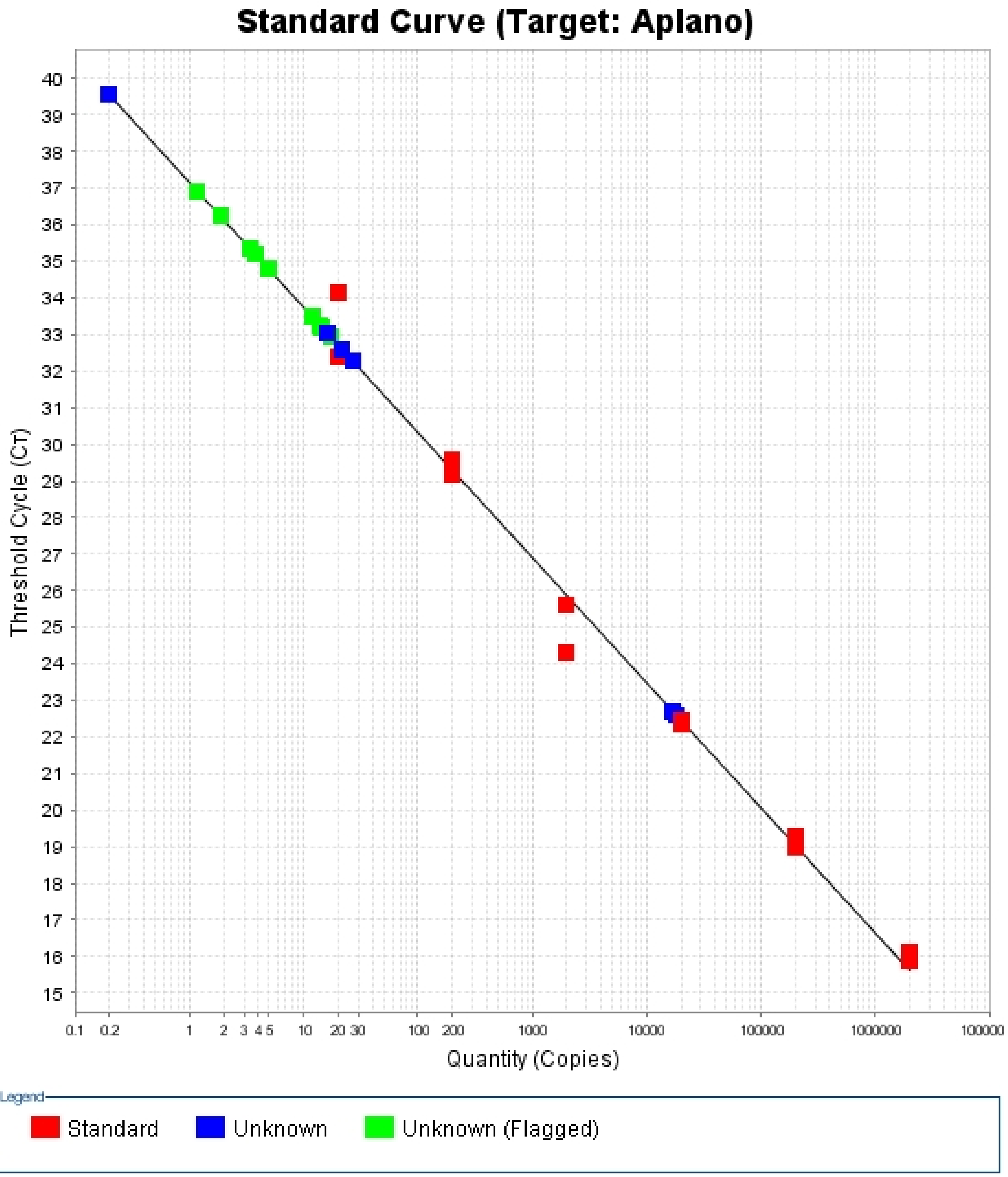

slope:-3.418 Y-Intercept:37.175 R^2:0.987 Eff%:96.16

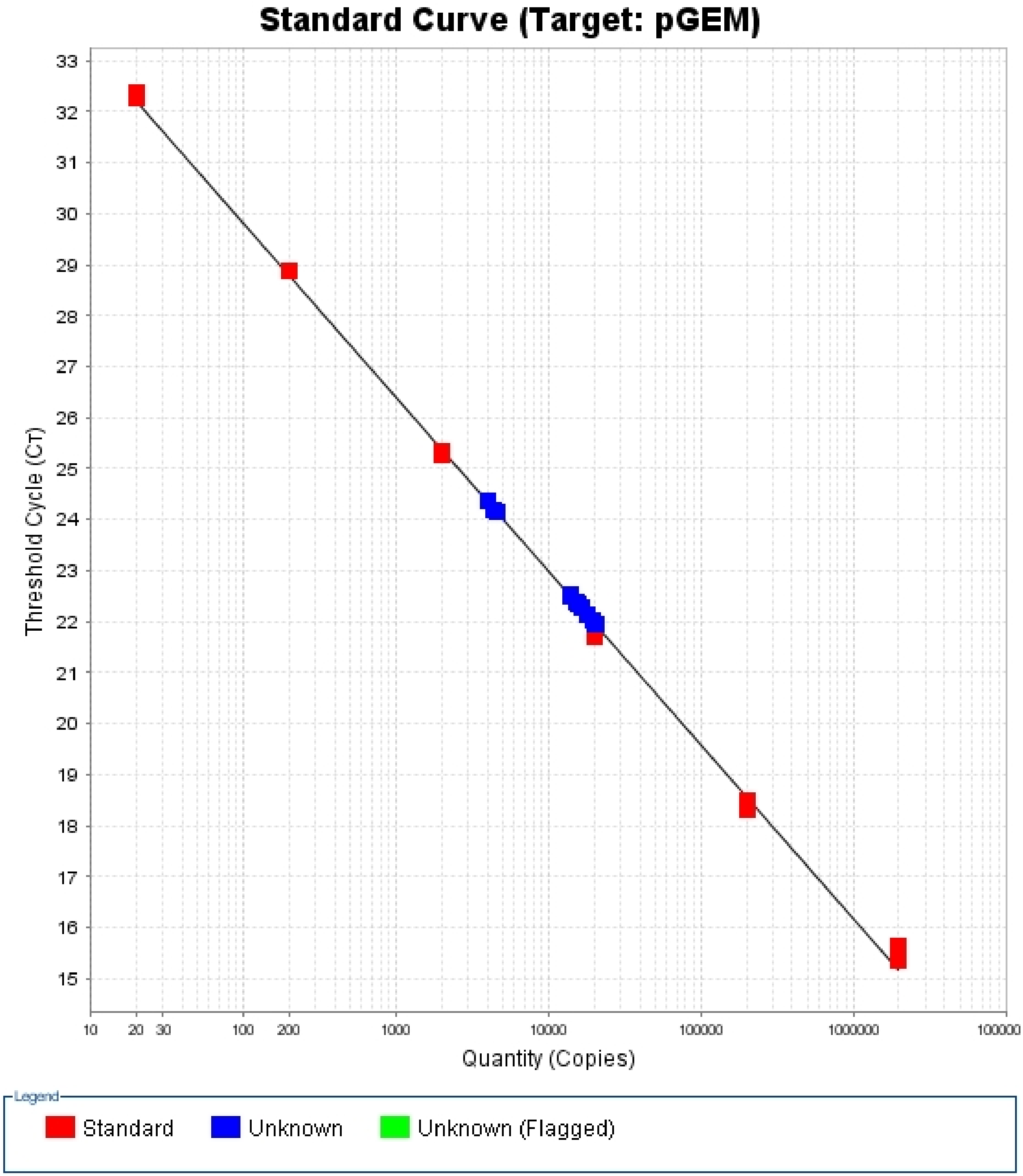

slope:-3.4 Y-Intercept:36.615 R^2:0.999 Eff%:96.85

Results Table

Well		Sample	Target	Task	Quantity	C
A1			pGEM	STANDARD	2000000	15.6529
A2			pGEM	STANDARD	2000000	15.3446
A3		2205 A01 SCM	pGEM	UNKNOWN	15825.4902	22.3376
A4		2205 A01 SCM	pGEM	UNKNOWN	13980.5645	22.5206
A5		2205 A01 SCM	pGEM	UNKNOWN	14176.9971	22.5
A7			Aplano	STANDARD	2000000	15.8864
A8			Aplano	STANDARD	2000000	16.1123
A9		2205 A01 10m	Aplano	UNKNOWN	27.0598	32.2797
A10		2205 A01 10m	Aplano	UNKNOWN	21.6864	32.6083
A11		2205 A01 10m	Aplano	UNKNOWN	16.1109	33.0494
B1			pGEM	STANDARD	200000	18.5056
B2			pGEM	STANDARD	200000	18.3194
B3		2205 A01 SCM	pGEM	UNKNOWN	16550.8164	22.2715
B4		2205 A01 SCM	pGEM	UNKNOWN	20593.3418	21.9488
B5		2205 A01 SCM	pGEM	UNKNOWN	20494.6992	21.9559
B7			Aplano	STANDARD	200000	19.2937
B8			Aplano	STANDARD	200000	18.9612
B9		2205 A01 SCM	Aplano	UNKNOWN	3.8066	35.1907
B10		2205 A01 SCM	Aplano	UNKNOWN	1.8708	36.245
B11		2205 A01 SCM	Aplano	UNKNOWN	1.1835	36.9246
C1			pGEM	STANDARD	20000	21.7168
C2			pGEM	STANDARD	20000	21.7951
C3		2205 A04	pGEM	UNKNOWN	15670.6182	22.3521
C4		2205 A04	pGEM	UNKNOWN	15309.7129	22.3865
C5		2205 A04	pGEM	UNKNOWN	15377.6787	22.38
C7			Aplano	STANDARD	20000	22.3535
C8			Aplano	STANDARD	20000	22.4253
C9		2205 A01 BTM	Aplano	UNKNOWN		Undetermined
C10		2205 A01 BTM	Aplano	UNKNOWN	5.0024	34.7853
C11		2205 A01 BTM	Aplano	UNKNOWN	14.5124	33.2045

Well		Sample	Target	Task	Quantity	C
D1			pGEM	STANDARD	2000	25.268
D2			pGEM	STANDARD	2000	25.3378
D3		2205A04 3000m	pGEM	UNKNOWN	4033.842	24.3559
D4		2205A04 3000m	pGEM	UNKNOWN	4653.6211	24.1448
D5		2205A04 3000m	pGEM	UNKNOWN	4462.7642	24.2067
D7			Aplano	STANDARD	2000	24.2899
D8			Aplano	STANDARD	2000	25.6145
D9		2205 A01 10m	Aplano	UNKNOWN	13.9355	33.2647
D10		2205 A01 10m	Aplano	UNKNOWN		Undetermined
D11		2205 A01 10m	Aplano	UNKNOWN	3.4326	35.3442
E1			pGEM	STANDARD	200	28.8579
E2			pGEM	STANDARD	200	28.8654
E7			Aplano	STANDARD	200	29.1626
E8			Aplano	STANDARD	200	29.5932
E9		2205 A01 SCM	Aplano	UNKNOWN	17.5497	32.9224
E10		2205 A01 SCM	Aplano	UNKNOWN	11.844	33.506
E11		2205 A01 SCM	Aplano	UNKNOWN	26.9789	32.2842
F1			pGEM	STANDARD	20	32.3743
F2			pGEM	STANDARD	20	32.2633
F7			Aplano	STANDARD	20	32.3725
F8			Aplano	STANDARD	20	34.1469
F9		2205 A01 BTM	Aplano	UNKNOWN		Undetermined
F10		2205 A01 BTM	Aplano	UNKNOWN	0.1987	39.5733
F11		2205 A01 BTM	Aplano	UNKNOWN		Undetermined
H1		Comf2E4	pGEM	UNKNOWN	17992.5449	22.1481
H2		Comf2E4	pGEM	UNKNOWN	19528.4805	22.0272
H4			pGEM	NTC		36.9648
H5			pGEM	NTC		32.6786
H7		Comf2E4	Aplano	UNKNOWN	18158.209	22.6193
H8		Comf2E4	Aplano	UNKNOWN	16971.7168	22.7196
H10			Aplano	NTC		Undetermined
H11			Aplano	NTC		Undetermined

QC Summary

Total Wells	96	Processed Wells	62	Targets Used	2
Well Setup	62	Flagged Wells	20	Samples Used	10

Flag	Name	Frequency	Locations
AMPNC	Amplification in negative control	1	H5
BADROX	Bad passive reference signal	0	
BLFAIL	Baseline algorithm failed	0	
CTFAIL	C algorithm failed	0	
EXPFAIL	Exponential algorithm failed	4	C9, D10, F9, F11
HIGHSD	High standard deviation in replicate group	16	B9, B10, B11, C10, C11, D7, D8, D9, D11, E9, E10, E11, F7, F8, H4, H5
NOAMP	No amplification	3	C9, D10, F11
NOISE	Noise higher than others in plate	0	
NOSIGNAL	No signal in well	0	
OFFSCALE	Fluorescence is offscale	0	
OUTLIERRG	Outlier in replicate group	0	
SPIKE	Noise spikes	0	
THOLDFAIL	Thresholding algorithm failed	0	
