# Supplementary material for: Ecological impact of algal-preying protists aplanochytrids (Labyrinthulea) as a source of docosahexaenoic acid for copepods in marine environments
Source: ISME Commun. 2026 Jun 26;6(1):ycag178. doi: 10.1093/ismeco/ycag178 (PMC13685785; doi:10.1093/ismeco/ycag178)
Supplement: Supplementary file 2 [file supplementary_materials_ycag178.zip › Morimoto_ISME_C_3rdrev_supplemental_data.zip!/Morimoto_ISME_C_3rdrev_supplemental_data/Supplemental_Data_2/Standard Curve/DNA recovery/2205/DnaRec2205AlineA04_N=1.pdf]

Experiment Results Report
230302DnaRecEmm2205AlineA04byKurabo

Experiment Summary

Experiment Name	:	230302DnaRecEmm2205AlineA04byKurabo
Experiment Type	:	Quantitation - Standard Curve
File Name	:	230302DnaRecEmm2205AlineA04byKurabo.eds
Run Started	:	2023 Mar 02 4:40:47 PM
Run Finished	:	2023 Mar 02 6:15:19 PM
Run Duration	:	94 minutes 32 seconds
Date Modified	:	2023 Mar 03 11:01:18 AM
User	:	
Number of wells used	:	46
Number of wells with results	:	46
Instrument Name	:	
Instrument Type	:	Applied Biosystems StepOnePlus™ Instrument
Comments	:	

Results Summary

Sample	Target	Quantity (Mean)	Quantity (Std Dev)	C (Mean)	C (Std Dev)
2205 A04 1000m ato	pGEM	23101.02	1698.58	25.42	0.11
2205 A04 1000m	pGEM	9673.49	1259.12	26.72	0.20
2205 A04 10m ato	pGEM	23697.58	810.13	25.38	0.05
2205 A04 10m saki	pGEM	6758.12	508.63	27.25	0.11
2205 A04 200m ato	pGEM	21041.56	972.72	25.56	0.07
2205 A04 200m saki	pGEM	11579.65	333.46	26.45	0.04
2205 A04 3000m ato	pGEM	21541.70	1848.20	25.53	0.12
2205 A04 3000m	pGEM	8845.89	137.19	26.85	0.02
2205 A04 SCM ato	pGEM	22231.92	160.63	25.48	0.01
2205 A04 SCM saki	pGEM	6321.57	223.36	27.34	0.05
Comf2E4	pGEM	21279.93	1527.48	25.55	0.11

Plate Layout

	1	2	3	4	5	6	7	8	9	10	11	12
A	S pGEM<br>2E6<br>FAM-NFQ-MGB<br>CT: 19.15	S pGEM<br>2E6<br>FAM-NFQ-MGB<br>CT: 18.72		2205 A04 10m saki<br>U pGEM<br>6.42E3<br>FAM-NFQ-MGB<br>CT: 27.32	2205 A04 10m saki<br>U pGEM<br>6.51E3<br>FAM-NFQ-MGB<br>CT: 27.3	2205 A04 10m saki<br>U pGEM<br>7.34E3<br>FAM-NFQ-MGB<br>CT: 27.12		2205 A04 3000m saki<br>U pGEM<br>8.69E3<br>FAM-NFQ-MGB<br>CT: 26.87	2205 A04 3000m saki<br>U pGEM<br>8.92E3<br>FAM-NFQ-MGB<br>CT: 26.83	2205 A04 3000m saki<br>U pGEM<br>8.93E3<br>FAM-NFQ-MGB<br>CT: 26.83		
B	S pGEM<br>2E5<br>FAM-NFQ-MGB<br>CT: 21.91	S pGEM<br>2E5<br>FAM-NFQ-MGB<br>CT: 21.91		2205 A04 10m ato<br>U pGEM<br>2.32E4<br>FAM-NFQ-MGB<br>CT: 25.42	2205 A04 10m ato<br>U pGEM<br>2.46E4<br>FAM-NFQ-MGB<br>CT: 25.33	2205 A04 10m ato<br>U pGEM<br>2.33E4<br>FAM-NFQ-MGB<br>CT: 25.41		2205 A04 3000m ato<br>U pGEM<br>2.37E4<br>FAM-NFQ-MGB<br>CT: 25.39	2205 A04 3000m ato<br>U pGEM<br>2.07E4<br>FAM-NFQ-MGB<br>CT: 25.59	2205 A04 3000m ato<br>U pGEM<br>2.03E4<br>FAM-NFQ-MGB<br>CT: 25.61		
C	⚠<br>S pGEM<br>2E4<br>FAM-NFQ-MGB<br>CT: 26.59	⚠<br>S pGEM<br>2E4<br>FAM-NFQ-MGB<br>CT: 25.38		2205 A04 SCM saki<br>U pGEM<br>6.24E3<br>FAM-NFQ-MGB<br>CT: 27.36	2205 A04 SCM saki<br>U pGEM<br>6.57E3<br>FAM-NFQ-MGB<br>CT: 27.29	2205 A04 SCM saki<br>U pGEM<br>6.15E3<br>FAM-NFQ-MGB<br>CT: 27.38						
D	S pGEM<br>2E3<br>FAM-NFQ-MGB<br>CT: 28.85	S pGEM<br>2E3<br>FAM-NFQ-MGB<br>CT: 28.89		2205 A04 SCM ato<br>U pGEM<br>2.21E4<br>FAM-NFQ-MGB<br>CT: 25.49	2205 A04 SCM ato<br>U pGEM<br>2.22E4<br>FAM-NFQ-MGB<br>CT: 25.48	2205 A04 SCM ato<br>U pGEM<br>2.24E4<br>FAM-NFQ-MGB<br>CT: 25.47						
E	S pGEM<br>200<br>FAM-NFQ-MGB<br>CT: 32.48	S pGEM<br>200<br>FAM-NFQ-MGB<br>CT: 32.31		2205 A04 200m saki<br>U pGEM<br>1.15E4<br>FAM-NFQ-MGB<br>CT: 26.46	2205 A04 200m saki<br>U pGEM<br>1.13E4<br>FAM-NFQ-MGB<br>CT: 26.48	2205 A04 200m saki<br>U pGEM<br>1.19E4<br>FAM-NFQ-MGB<br>CT: 26.4						
F	S pGEM<br>20<br>FAM-NFQ-MGB<br>CT: 35.98	S pGEM<br>20<br>FAM-NFQ-MGB<br>CT: 35.96		2205 A04 200m ato<br>U pGEM<br>2.01E4<br>FAM-NFQ-MGB<br>CT: 25.63	2205 A04 200m ato<br>U pGEM<br>2.1E4<br>FAM-NFQ-MGB<br>CT: 25.56	2205 A04 200m ato<br>U pGEM<br>2.2E4<br>FAM-NFQ-MGB<br>CT: 25.49						
G				2205 A04 1000m saki<br>U pGEM<br>1E4<br>FAM-NFQ-MGB<br>CT: 26.66	2205 A04 1000m saki<br>U pGEM<br>1.07E4<br>FAM-NFQ-MGB<br>CT: 26.56	2205 A04 1000m saki<br>U pGEM<br>8.28E3<br>FAM-NFQ-MGB<br>CT: 26.94						
H	Comf2E4<br>U pGEM<br>2.02E4<br>FAM-NFQ-MGB<br>CT: 25.62	Comf2E4<br>U pGEM<br>2.24E4<br>FAM-NFQ-MGB<br>CT: 25.47		2205 A04 1000m ato<br>U pGEM<br>2.39E4<br>FAM-NFQ-MGB<br>CT: 25.37	2205 A04 1000m ato<br>U pGEM<br>2.42E4<br>FAM-NFQ-MGB<br>CT: 25.35	2205 A04 1000m ato<br>U pGEM<br>2.11E4<br>FAM-NFQ-MGB<br>CT: 25.55					N pGEM<br>FAM-NFQ-MGB<br>CT: Undetermined	N pGEM<br>FAM-NFQ-MGB<br>CT: Undetermined

Standard Curves

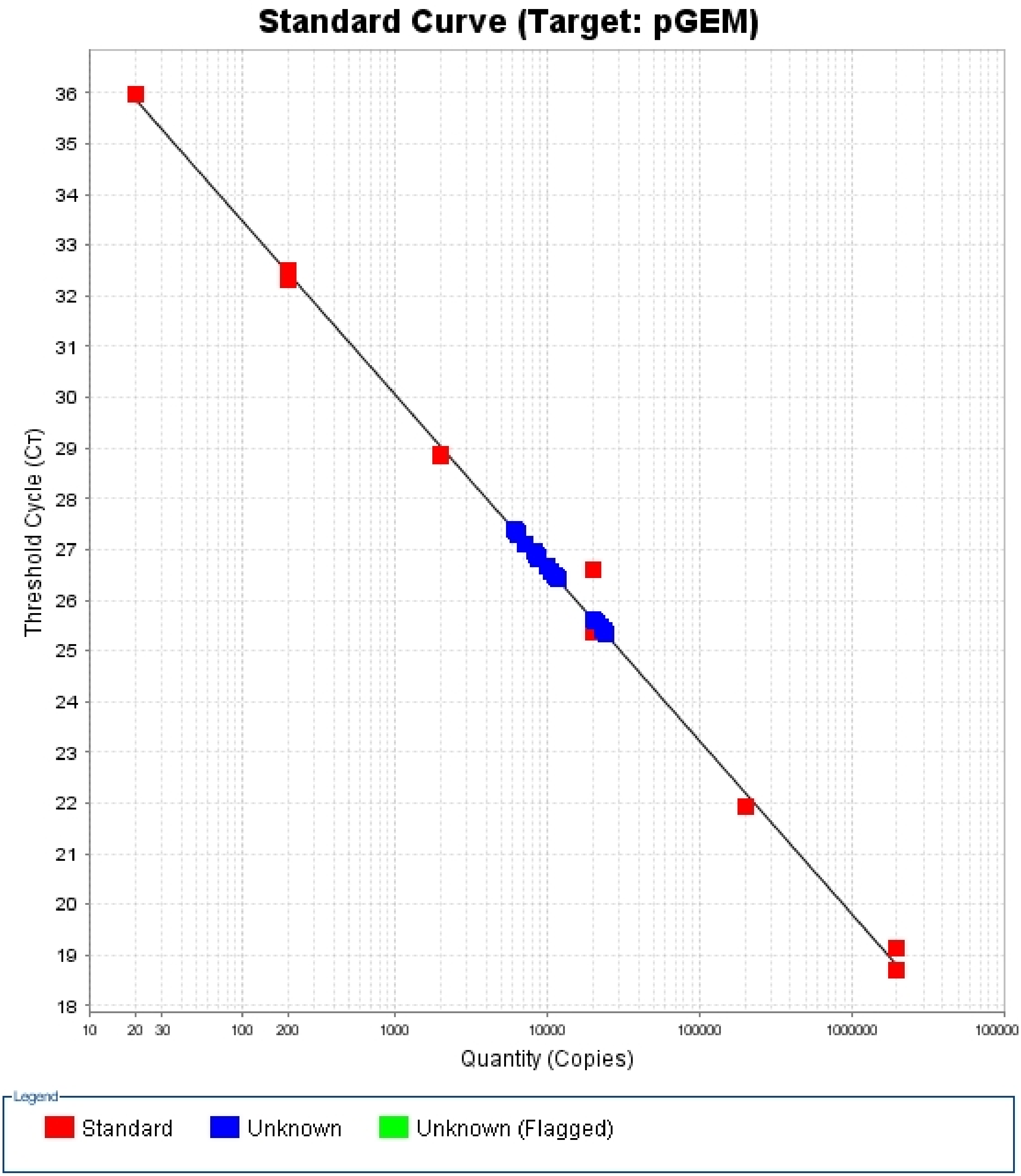

slope:-3.415 Y-Intercept:40.322 R^2:0.997 Eff%:96.273

Results Table

Well		Sample	Target	Task	Quantity	C
A1			pGEM	STANDARD	2000000	19.1453
A2			pGEM	STANDARD	2000000	18.7207
A4		2205 A04 10m	pGEM	UNKNOWN	6422.5869	27.3203
A5		2205 A04 10m	pGEM	UNKNOWN	6508.4307	27.3006
A6		2205 A04 10m	pGEM	UNKNOWN	7343.3369	27.1216
A8		2205 A04	pGEM	UNKNOWN	8687.6396	26.8723
A9		2205 A04	pGEM	UNKNOWN	8918.7773	26.8334
A10		2205 A04	pGEM	UNKNOWN	8931.2559	26.8313
B1			pGEM	STANDARD	200000	21.9117
B2			pGEM	STANDARD	200000	21.9107
B4		2205 A04 10m	pGEM	UNKNOWN	23179.2402	25.417
B5		2205 A04 10m	pGEM	UNKNOWN	24631.1445	25.3269
B6		2205 A04 10m	pGEM	UNKNOWN	23282.3535	25.4104
B8		2205 A04	pGEM	UNKNOWN	23663.9297	25.3863
B9		2205 A04	pGEM	UNKNOWN	20675.3906	25.5866
B10		2205 A04	pGEM	UNKNOWN	20285.7793	25.6148
C1			pGEM	STANDARD	20000	26.5879
C2			pGEM	STANDARD	20000	25.3796
C4		2205 A04 SCM	pGEM	UNKNOWN	6239.6421	27.3631
C5		2205 A04 SCM	pGEM	UNKNOWN	6574.333	27.2857
C6		2205 A04 SCM	pGEM	UNKNOWN	6150.7422	27.3844
D1			pGEM	STANDARD	2000	28.8473
D2			pGEM	STANDARD	2000	28.8859
D4		2205 A04 SCM	pGEM	UNKNOWN	22097.8184	25.4879
D5		2205 A04 SCM	pGEM	UNKNOWN	22187.9863	25.4818
D6		2205 A04 SCM	pGEM	UNKNOWN	22409.9453	25.4671
E1			pGEM	STANDARD	200	32.4823
E2			pGEM	STANDARD	200	32.3071
E4		2205 A04 200m	pGEM	UNKNOWN	11489.2607	26.4578
E5		2205 A04 200m	pGEM	UNKNOWN	11300.6992	26.4824

Well		Sample	Target	Task	Quantity	C
E6		2205 A04 200m	pGEM	UNKNOWN	11948.9902	26.3996
F1			pGEM	STANDARD	20	35.9779
F2			pGEM	STANDARD	20	35.9597
F4		2205 A04 200m	pGEM	UNKNOWN	20093.4883	25.6289
F5		2205 A04 200m	pGEM	UNKNOWN	20994.0059	25.5639
F6		2205 A04 200m	pGEM	UNKNOWN	22037.1934	25.492
G4		2205 A04	pGEM	UNKNOWN	10003.3564	26.6632
G5		2205 A04	pGEM	UNKNOWN	10734.835	26.5585
G6		2205 A04	pGEM	UNKNOWN	8282.2666	26.9432
H1		Comf2E4	pGEM	UNKNOWN	20199.834	25.6211
H2		Comf2E4	pGEM	UNKNOWN	22360.0215	25.4704
H4		2205 A04	pGEM	UNKNOWN	23930.8594	25.3697
H5		2205 A04	pGEM	UNKNOWN	24225.1543	25.3516
H6		2205 A04	pGEM	UNKNOWN	21147.043	25.5531
H11			pGEM	NTC		Undetermined
H12			pGEM	NTC		Undetermined

QC Summary

Total Wells	96	Processed Wells	46	Targets Used	1
Well Setup	46	Flagged Wells	2	Samples Used	11

Flag	Name	Frequency	Locations
AMPNC	Amplification in negative control	0	
BADROX	Bad passive reference signal	0	
BLFAIL	Baseline algorithm failed	0	
CTFAIL	C algorithm failed	0	
EXPFAIL	Exponential algorithm failed	0	
HIGHSD	High standard deviation in replicate group	2	C1, C2
NOAMP	No amplification	0	
NOISE	Noise higher than others in plate	0	
NOSIGNAL	No signal in well	0	
OFFSCALE	Fluorescence is offscale	0	
OUTLIERRG	Outlier in replicate group	0	
SPIKE	Noise spikes	0	
THOLDFAIL	Thresholding algorithm failed	0	
