# Supplementary material for: Ecological impact of algal-preying protists aplanochytrids (Labyrinthulea) as a source of docosahexaenoic acid for copepods in marine environments
Source: ISME Commun. 2026 Jun 26;6(1):ycag178. doi: 10.1093/ismeco/ycag178 (PMC13685785; doi:10.1093/ismeco/ycag178)
Supplement: Supplementary file 2 [file supplementary_materials_ycag178.zip › Morimoto_ISME_C_3rdrev_supplemental_data.zip!/Morimoto_ISME_C_3rdrev_supplemental_data/Supplemental_Data_2/Standard Curve/DNA recovery/2205/DnaRecAline2205amari1_N=3.pdf]

Experiment Results Report

230322DnaRecAline2205A010407EmmByKurabo

Experiment Summary

Experiment Name	:	230322DnaRecAline2205A010407EmmByKurabo
Experiment Type	:	Quantitation - Standard Curve
File Name	:	230322DnaRecAline2205A010407EmmByKurabo.eds
Run Started	:	2023 Mar 22 11:58:51 AM
Run Finished	:	2023 Mar 22 1:33:25 PM
Run Duration	:	94 minutes 33 seconds
Date Modified	:	2023 Mar 22 1:46:44 PM
User	:	
Number of wells used	:	34
Number of wells with results	:	34
Instrument Name	:	
Instrument Type	:	Applied Biosystems StepOnePlus™ Instrument
Comments	:	

Results Summary

Sample	Target	Quantity (Mean)	Quantity (Std Dev)	C (Mean)	C (Std Dev)
2205 A01 SCM ato3	pGEM				
2205 A01 SCM saki3	pGEM	15477.61	1708.67	25.58	0.17
2205 A04 3000m	pGEM	17089.33	322.53	25.42	0.03
2205 A04 3000m	pGEM				
2205 A07 3000m	pGEM	24959.23	2249.94	24.85	0.14
2205 A07 3000m	pGEM	17785.39	187.42	25.36	0.02
Comf2E4	pGEM	21793.53	1835.65	25.05	0.13

Plate Layout

	1	2	3	4	5	6	7	8	9	10	11	12
A	S pGEM<br>2E6<br>FAM-NFQ-MGB<br>CT: 18.34	S pGEM<br>2E6<br>FAM-NFQ-MGB<br>CT: 18.18		2205 A01 SCM saki3<br>U pGEM<br>1.73E4<br>FAM-NFQ-MGB<br>CT: 25.4	2205 A01 SCM saki3<br>U pGEM<br>1.52E4<br>FAM-NFQ-MGB<br>CT: 25.6	2205 A01 SCM saki3<br>U pGEM<br>1.39E4<br>FAM-NFQ-MGB<br>CT: 25.74						
B	S pGEM<br>2E5<br>FAM-NFQ-MGB<br>CT: 21.48	S pGEM<br>2E5<br>FAM-NFQ-MGB<br>CT: 21.45		⚠1<br>2205 A01 SCM ato3<br>U pGEM<br>FAM-NFQ-MGB<br>CT: Undetermined	⚠2<br>2205 A01 SCM ato3<br>U pGEM<br>FAM-NFQ-MGB<br>CT: Undetermined	⚠1<br>2205 A01 SCM ato3<br>U pGEM<br>FAM-NFQ-MGB<br>CT: Undetermined						
C	S pGEM<br>2E4<br>FAM-NFQ-MGB<br>CT: 25.11	S pGEM<br>2E4<br>FAM-NFQ-MGB<br>CT: 25.02		⚠1<br>2205 A04 3000m saki3<br>U pGEM<br>FAM-NFQ-MGB<br>CT: Undetermined	⚠2<br>2205 A04 3000m saki3<br>U pGEM<br>FAM-NFQ-MGB<br>CT: Undetermined	⚠1<br>2205 A04 3000m saki3<br>U pGEM<br>FAM-NFQ-MGB<br>CT: Undetermined						
D	⚠1<br>S pGEM<br>2E3<br>FAM-NFQ-MGB<br>CT: 29.43	⚠1<br>S pGEM<br>2E3<br>FAM-NFQ-MGB<br>CT: 28.5		2205 A04 3000m ato3<br>U pGEM<br>1.74E4<br>FAM-NFQ-MGB<br>CT: 25.39	2205 A04 3000m ato3<br>U pGEM<br>1.71E4<br>FAM-NFQ-MGB<br>CT: 25.42	2205 A04 3000m ato3<br>U pGEM<br>1.68E4<br>FAM-NFQ-MGB<br>CT: 25.45						
E	S pGEM<br>200<br>FAM-NFQ-MGB<br>CT: 32.38	S pGEM<br>200<br>FAM-NFQ-MGB<br>CT: 32.36		2205 A07 3000m saki3<br>U pGEM<br>1.79E4<br>FAM-NFQ-MGB<br>CT: 25.35	2205 A07 3000m saki3<br>U pGEM<br>1.76E4<br>FAM-NFQ-MGB<br>CT: 25.38	2205 A07 3000m saki3<br>U pGEM<br>1.79E4<br>FAM-NFQ-MGB<br>CT: 25.35						
F	⚠1<br>S pGEM<br>20<br>FAM-NFQ-MGB<br>CT: 35.02	⚠1<br>S pGEM<br>20<br>FAM-NFQ-MGB<br>CT: 35.99		2205 A07 3000m ato3<br>U pGEM<br>2.25E4<br>FAM-NFQ-MGB<br>CT: 25	2205 A07 3000m ato3<br>U pGEM<br>2.69E4<br>FAM-NFQ-MGB<br>CT: 24.73	2205 A07 3000m ato3<br>U pGEM<br>2.54E4<br>FAM-NFQ-MGB<br>CT: 24.82						
G												
H	Comf2E4<br>U pGEM<br>2.05E4<br>FAM-NFQ-MGB<br>CT: 25.15	Comf2E4<br>U pGEM<br>2.31E4<br>FAM-NFQ-MGB<br>CT: 24.96									N pGEM<br>FAM-NFQ-MGB<br>CT: Undetermined	N pGEM<br>FAM-NFQ-MGB<br>CT: Undetermined

Standard Curves

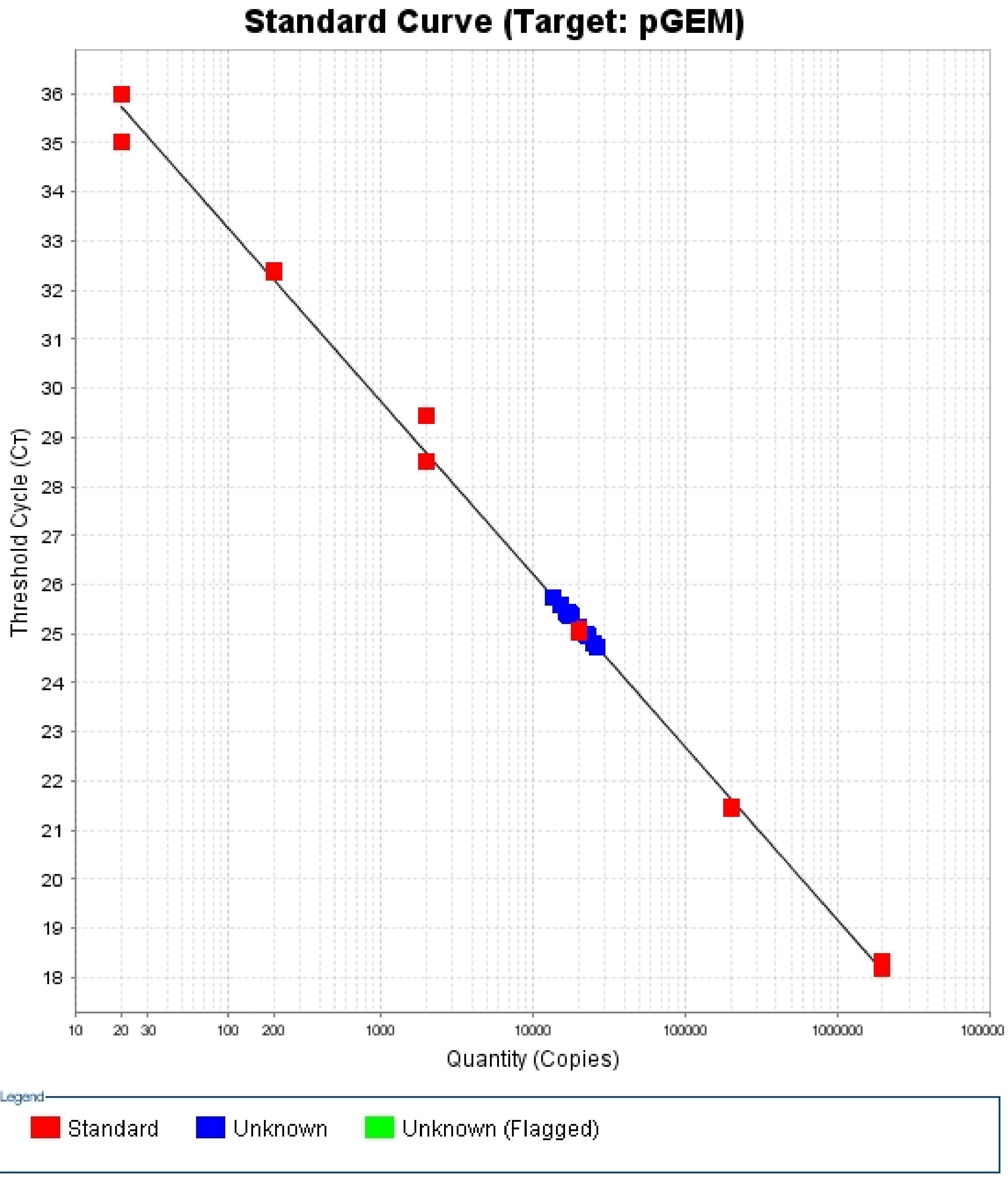

slope:-3.51 Y-Intercept:40.278 R^2:0.997 Eff%:92.719

Results Table

Well		Sample	Target	Task	Quantity	C
A1			pGEM	STANDARD	2000000	18.3392
A2			pGEM	STANDARD	2000000	18.1755
A4		2205 A01 SCM	pGEM	UNKNOWN	17306.8145	25.4036
A5		2205 A01 SCM	pGEM	UNKNOWN	15203.3594	25.6011
A6		2205 A01 SCM	pGEM	UNKNOWN	13922.6514	25.7353
B1			pGEM	STANDARD	200000	21.4809
B2			pGEM	STANDARD	200000	21.4457
B4		2205 A01 SCM	pGEM	UNKNOWN		Undetermined
B5		2205 A01 SCM	pGEM	UNKNOWN		Undetermined
B6		2205 A01 SCM	pGEM	UNKNOWN		Undetermined
C1			pGEM	STANDARD	20000	25.1149
C2			pGEM	STANDARD	20000	25.0224
C4		2205 A04	pGEM	UNKNOWN		Undetermined
C5		2205 A04	pGEM	UNKNOWN		Undetermined
C6		2205 A04	pGEM	UNKNOWN		Undetermined
D1			pGEM	STANDARD	2000	29.4308
D2			pGEM	STANDARD	2000	28.5003
D4		2205 A04	pGEM	UNKNOWN	17414.5879	25.3942
D5		2205 A04	pGEM	UNKNOWN	17083.8125	25.4234
D6		2205 A04	pGEM	UNKNOWN	16769.6016	25.4517
E1			pGEM	STANDARD	200	32.3821
E2			pGEM	STANDARD	200	32.3582
E4		2205 A07	pGEM	UNKNOWN	17895.5117	25.3526
E5		2205 A07	pGEM	UNKNOWN	17568.9863	25.3807
E6		2205 A07	pGEM	UNKNOWN	17891.6602	25.353
F1			pGEM	STANDARD	20	35.015
F2			pGEM	STANDARD	20	35.9892
F4		2205 A07	pGEM	UNKNOWN	22511.2383	25.0029
F5		2205 A07	pGEM	UNKNOWN	26936.6953	24.7293
F6		2205 A07	pGEM	UNKNOWN	25429.7637	24.8171

Well		Sample	Target	Task	Quantity	C
H1		Comf2E4	pGEM	UNKNOWN	20495.5254	25.1459
H2		Comf2E4	pGEM	UNKNOWN	23091.5254	24.9641
H11			pGEM	NTC		Undetermined
H12			pGEM	NTC		Undetermined

QC Summary

Total Wells	96	Processed Wells	34	Targets Used	1
Well Setup	34	Flagged Wells	10	Samples Used	7

Flag	Name	Frequency	Locations
AMPNC	Amplification in negative control	0	
BADROX	Bad passive reference signal	0	
BLFAIL	Baseline algorithm failed	0	
CTFAIL	C algorithm failed	0	
EXPFAIL	Exponential algorithm failed	6	B4, B5, B6, C4, C5, C6
HIGHSD	High standard deviation in replicate group	4	D1, D2, F1, F2
NOAMP	No amplification	2	B5, C5
NOISE	Noise higher than others in plate	0	
NOSIGNAL	No signal in well	0	
OFFSCALE	Fluorescence is offscale	0	
OUTLIERRG	Outlier in replicate group	0	
SPIKE	Noise spikes	0	
THOLDFAIL	Thresholding algorithm failed	0	
