# Supplementary material for: Ecological impact of algal-preying protists aplanochytrids (Labyrinthulea) as a source of docosahexaenoic acid for copepods in marine environments
Source: ISME Commun. 2026 Jun 26;6(1):ycag178. doi: 10.1093/ismeco/ycag178 (PMC13685785; doi:10.1093/ismeco/ycag178)
Supplement: Supplementary file 2 [file supplementary_materials_ycag178.zip › Morimoto_ISME_C_3rdrev_supplemental_data.zip!/Morimoto_ISME_C_3rdrev_supplemental_data/Supplemental_Data_2/Standard Curve/DNA recovery/2205/DnaRec2205A01_N=3.pdf]

Experiment Results Report

230317DnaRecAline2205A01DnaExtN=3EmmByKurabo

Experiment Summary

Experiment Name	: 230317DnaRecAline2205A01DnaExtN=3EmmByKurabo
Experiment Type	: Quantitation - Standard Curve
File Name	: 230317DnaRecAline2205A01DnaExtN=3EmmByKurabo.eds
Run Started	: 2023 Mar 17 12:28:35 PM
Run Finished	: 2023 Mar 17 2:03:17 PM
Run Duration	: 94 minutes 41 seconds
Date Modified	: 2023 Mar 17 2:30:07 PM
User	:
Number of wells used	: 46
Number of wells with results	: 46
Instrument Name	:
Instrument Type	: Applied Biosystems StepOnePlus™ Instrument
Comments	:

Results Summary

Sample	Target	Quantity (Mean)	Quantity (Std Dev)	C (Mean)	C (Std Dev)
2205 A01 10m ato3	pGEM	24621.96	536.00	25.56	0.03
2205 A01 10m saki3	pGEM	12200.24	681.65	26.64	0.09
2205 A01 BTM ato2	pGEM	26547.66	1718.12	25.45	0.10
2205 A01 BTM ato3	pGEM	25326.92	1050.82	25.52	0.06
2205 A01 BTM saki2	pGEM	16277.10	517.23	26.20	0.05
2205 A01 BTM saki3	pGEM	11901.97	1320.43	26.68	0.17
2205 A01 SCM ato2	pGEM	27394.10	1190.31	25.40	0.07
2205 A01 SCM	pGEM	11132.33	331.02	26.78	0.05
2205 A01 10m ato2	pGEM	26512.88	1152.34	25.45	0.07
2205 A01 10m saki2	pGEM	14540.92	462.81	26.37	0.05
Comf2E4	pGEM	22374.41	1099.20	25.71	0.07

Plate Layout

	1	2	3	4	5	6	7	8	9	10	11	12
A	S pGEM<br>2E6<br>FAM-NFQ-MGB<br>CT: 18.99	S pGEM<br>2E6<br>FAM-NFQ-MGB<br>CT: 18.95		2205 A01 10m saki2<br>U pGEM<br>1.41E4<br>FAM-NFQ-MGB<br>CT: 26.42	2205 A01 10m saki2<br>U pGEM<br>1.5E4<br>FAM-NFQ-MGB<br>CT: 26.32	2205 A01 10m saki2<br>U pGEM<br>1.45E4<br>FAM-NFQ-MGB<br>CT: 26.37		2205 A01 10m saki3<br>U pGEM<br>1.26E4<br>FAM-NFQ-MGB<br>CT: 26.59	2205 A01 10m saki3<br>U pGEM<br>1.14E4<br>FAM-NFQ-MGB<br>CT: 26.74	2205 A01 10m saki3<br>U pGEM<br>1.26E4<br>FAM-NFQ-MGB<br>CT: 26.58		
B	S pGEM<br>2E5<br>FAM-NFQ-MGB<br>CT: 22.41	S pGEM<br>2E5<br>FAM-NFQ-MGB<br>CT: 22.22		2205 A01 10m ato2<br>U pGEM<br>2.59E4<br>FAM-NFQ-MGB<br>CT: 25.48	2205 A01 10m ato2<br>U pGEM<br>2.58E4<br>FAM-NFQ-MGB<br>CT: 25.49	2205 A01 10m ato2<br>U pGEM<br>2.78E4<br>FAM-NFQ-MGB<br>CT: 25.38		2205 A01 10m ato3<br>U pGEM<br>2.52E4<br>FAM-NFQ-MGB<br>CT: 25.53	2205 A01 10m ato3<br>U pGEM<br>2.42E4<br>FAM-NFQ-MGB<br>CT: 25.59	2205 A01 10m ato3<br>U pGEM<br>2.45E4<br>FAM-NFQ-MGB<br>CT: 25.57		
C	S pGEM<br>2E4<br>FAM-NFQ-MGB<br>CT: 25.81	S pGEM<br>2E4<br>FAM-NFQ-MGB<br>CT: 25.72		2205 A01 SCM saki2<br>U pGEM<br>1.12E4<br>FAM-NFQ-MGB<br>CT: 26.76	2205 A01 SCM saki2<br>U pGEM<br>FAM-NFQ-MGB<br>CT: 26.83	2205 A01 SCM saki2<br>U pGEM<br>1.14E4<br>FAM-NFQ-MGB<br>CT: 26.74		2205 A01 BTM saki3<br>U pGEM<br>1.29E4<br>FAM-NFQ-MGB<br>CT: 26.55	2205 A01 BTM saki3<br>U pGEM<br>1.04E4<br>FAM-NFQ-MGB<br>CT: 26.88	2205 A01 BTM saki3<br>U pGEM<br>1.24E4<br>FAM-NFQ-MGB<br>CT: 26.61		
D	S pGEM<br>2E3<br>FAM-NFQ-MGB<br>CT: 29.35	S pGEM<br>2E3<br>FAM-NFQ-MGB<br>CT: 29.35		2205 A01 SCM ato2<br>U pGEM<br>2.74E4<br>FAM-NFQ-MGB<br>CT: 25.4	2205 A01 SCM ato2<br>U pGEM<br>2.86E4<br>FAM-NFQ-MGB<br>CT: 25.34	2205 A01 SCM ato2<br>U pGEM<br>2.62E4<br>FAM-NFQ-MGB<br>CT: 25.47		2205 A01 BTM ato3<br>U pGEM<br>2.42E4<br>FAM-NFQ-MGB<br>CT: 25.59	2205 A01 BTM ato3<br>U pGEM<br>FAM-NFQ-MGB<br>CT: 25.46	2205 A01 BTM ato3<br>U pGEM<br>FAM-NFQ-MGB<br>CT: 25.51		
E	S pGEM<br>200<br>FAM-NFQ-MGB<br>CT: 32.96	S pGEM<br>200<br>FAM-NFQ-MGB<br>CT: 32.96		2205 A01 BTM saki2<br>U pGEM<br>1.62E4<br>FAM-NFQ-MGB<br>CT: 26.2	2205 A01 BTM saki2<br>U pGEM<br>1.68E4<br>FAM-NFQ-MGB<br>CT: 26.15	2205 A01 BTM saki2<br>U pGEM<br>1.58E4<br>FAM-NFQ-MGB<br>CT: 26.24						
F	S pGEM<br>20<br>FAM-NFQ-MGB<br>CT: 36.3	S pGEM<br>20<br>FAM-NFQ-MGB<br>CT: 36.63		2205 A01 BTM ato2<br>U pGEM<br>2.48E4<br>FAM-NFQ-MGB<br>CT: 25.56	2205 A01 BTM ato2<br>U pGEM<br>2.67E4<br>FAM-NFQ-MGB<br>CT: 25.44	2205 A01 BTM ato2<br>U pGEM<br>2.82E4<br>FAM-NFQ-MGB<br>CT: 25.36						
G												
H	Comf2E4<br>U pGEM<br>2.16E4<br>FAM-NFQ-MGB<br>CT: 25.76	Comf2E4<br>U pGEM<br>2.32E4<br>FAM-NFQ-MGB<br>CT: 25.66									N pGEM<br>FAM-NFQ-MGB<br>CT: Undetermined	N pGEM<br>FAM-NFQ-MGB<br>CT: Undetermined

Standard Curves

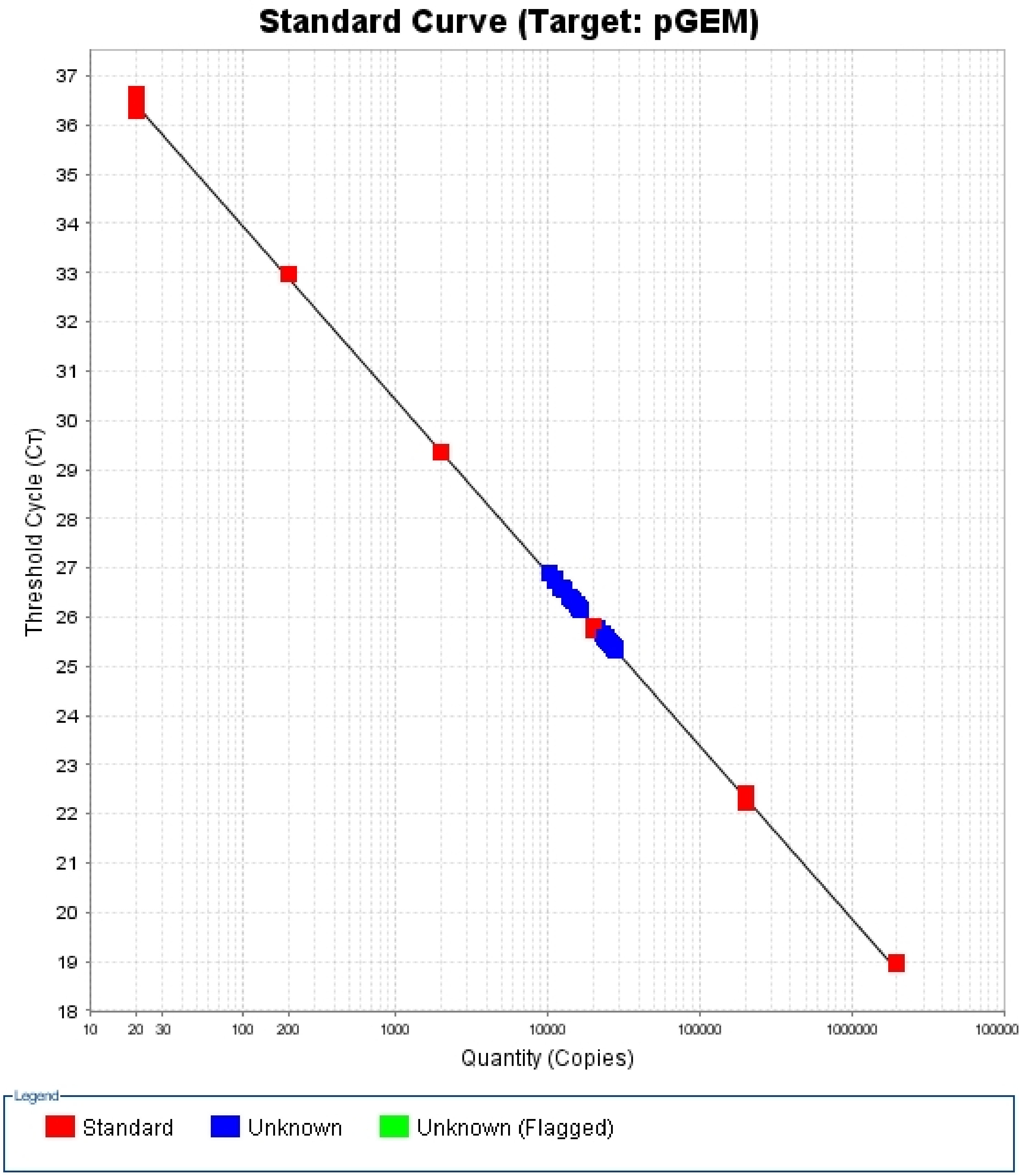

slope:-3.514 Y-Intercept:40.993 R^2:1.0 Eff%:92.579

Results Table

Well		Sample	Target	Task	Quantity	C
A1			pGEM	STANDARD	2000000	18.9916
A2			pGEM	STANDARD	2000000	18.9483
A4		2205 A01 10m	pGEM	UNKNOWN	14079.7373	26.4165
A5		2205 A01 10m	pGEM	UNKNOWN	15005.3496	26.3193
A6		2205 A01 10m	pGEM	UNKNOWN	14537.6846	26.3677
A8		2205 A01 10m	pGEM	UNKNOWN	12571.373	26.5894
A9		2205 A01 10m	pGEM	UNKNOWN	11413.5605	26.7368
A10		2205 A01 10m	pGEM	UNKNOWN	12615.7949	26.584
B1			pGEM	STANDARD	200000	22.4113
B2			pGEM	STANDARD	200000	22.222
B4		2205 A01 10m	pGEM	UNKNOWN	25928.8086	25.4847
B5		2205 A01 10m	pGEM	UNKNOWN	25769.5195	25.4941
B6		2205 A01 10m	pGEM	UNKNOWN	27840.2949	25.3762
B8		2205 A01 10m	pGEM	UNKNOWN	25210.9141	25.5276
B9		2205 A01 10m	pGEM	UNKNOWN	24162.7227	25.5924
B10		2205 A01 10m	pGEM	UNKNOWN	24492.252	25.5717
C1			pGEM	STANDARD	20000	25.8088
C2			pGEM	STANDARD	20000	25.7208
C4		2205 A01 SCM	pGEM	UNKNOWN	11221.6963	26.7627
C5		2205 A01 SCM	pGEM	UNKNOWN		26.826
C6		2205 A01 SCM	pGEM	UNKNOWN	11409.4961	26.7374
C8		2205 A01 BTM	pGEM	UNKNOWN	12871.5107	26.5534
C9		2205 A01 BTM	pGEM	UNKNOWN	10398.1143	26.879
C10		2205 A01 BTM	pGEM	UNKNOWN	12436.291	26.6059
D1			pGEM	STANDARD	2000	29.3545
D2			pGEM	STANDARD	2000	29.3501
D4		2205 A01 SCM	pGEM	UNKNOWN	27407.25	25.4001
D5		2205 A01 SCM	pGEM	UNKNOWN	28577.7852	25.3363
D6		2205 A01 SCM	pGEM	UNKNOWN	26197.2773	25.469
D8		2205 A01 BTM	pGEM	UNKNOWN	24205.8613	25.5897

Well		Sample	Target	Task	Quantity	C
D9		2205 A01 BTM	pGEM	UNKNOWN		25.4637
D10		2205 A01 BTM	pGEM	UNKNOWN		25.5111
E1			pGEM	STANDARD	200	32.9575
E2			pGEM	STANDARD	200	32.9577
E4		2205 A01 BTM	pGEM	UNKNOWN	16233.792	26.1993
E5		2205 A01 BTM	pGEM	UNKNOWN	16814.6191	26.1456
E6		2205 A01 BTM	pGEM	UNKNOWN	15782.8848	26.2423
F1			pGEM	STANDARD	20	36.297
F2			pGEM	STANDARD	20	36.6303
F4		2205 A01 BTM	pGEM	UNKNOWN	24751.3223	25.5557
F5		2205 A01 BTM	pGEM	UNKNOWN	26716.5918	25.4391
F6		2205 A01 BTM	pGEM	UNKNOWN	28175.0762	25.358
H1		Comf2E4	pGEM	UNKNOWN	21597.1562	25.7637
H2		Comf2E4	pGEM	UNKNOWN	23151.6602	25.6576
H11			pGEM	NTC		Undetermined
H12			pGEM	NTC		Undetermined

QC Summary

Total Wells	96	Processed Wells	46	Targets Used	1
Well Setup	46	Flagged Wells	0	Samples Used	11

Flag	Name	Frequency	Locations
AMPNC	Amplification in negative control	0	
BADROX	Bad passive reference signal	0	
BLFAIL	Baseline algorithm failed	0	
CTFAIL	C algorithm failed	0	
EXPFAIL	Exponential algorithm failed	0	
HIGHSD	High standard deviation in replicate group	0	
NOAMP	No amplification	0	
NOISE	Noise higher than others in plate	0	
NOSIGNAL	No signal in well	0	
OFFSCALE	Fluorescence is offscale	0	
OUTLIERRG	Outlier in replicate group	0	
SPIKE	Noise spikes	0	
THOLDFAIL	Thresholding algorithm failed	0	
