# Supplementary material for: Ecological impact of algal-preying protists aplanochytrids (Labyrinthulea) as a source of docosahexaenoic acid for copepods in marine environments
Source: ISME Commun. 2026 Jun 26;6(1):ycag178. doi: 10.1093/ismeco/ycag178 (PMC13685785; doi:10.1093/ismeco/ycag178)
Supplement: Supplementary file 2 [file supplementary_materials_ycag178.zip › Morimoto_ISME_C_3rdrev_supplemental_data.zip!/Morimoto_ISME_C_3rdrev_supplemental_data/Supplemental_Data_2/Standard Curve/DNA recovery/2205/DnaRec2205AlineA11_N=1.pdf]

Experiment Results Report

230302DnaRecEmmAline2205A11byKurabo

Experiment Summary

Experiment Name	:	230302DnaRecEmmAline2205A11byKurabo
Experiment Type	:	Quantitation - Standard Curve
File Name	:	230302DnaRecEmmAline2205A11byKurabo.eds
Run Started	:	2023 Mar 03 2:15:00 PM
Run Finished	:	2023 Mar 03 3:49:35 PM
Run Duration	:	94 minutes 34 seconds
Date Modified	:	2023 Mar 03 4:01:52 PM
User	:	
Number of wells used	:	46
Number of wells with results	:	46
Instrument Name	:	
Instrument Type	:	Applied Biosystems StepOnePlus™ Instrument
Comments	:	

Results Summary

Sample	Target	Quantity (Mean)	Quantity (Std Dev)	C (Mean)	C (Std Dev)
2205 A11 1000m ato	pGEM	20440.52	1042.85	25.50	0.07
2205 A11 1000m	pGEM	10348.88	1440.87	26.51	0.21
2205 A11 10m ato	pGEM	20603.67	1642.90	25.49	0.11
2205 A11 10m saki	pGEM	9153.24	195.81	26.68	0.03
2205 A11 200m ato	pGEM	18736.89	1644.11	25.63	0.13
2205 A11 200m saki	pGEM	6852.66	331.32	27.11	0.07
2205 A11 3000m ato	pGEM	18741.79	1499.32	25.63	0.11
2205 A11 3000m	pGEM	6937.07	457.95	27.09	0.10
2205 A11 SCM ato	pGEM	18352.76	1629.16	25.66	0.13
2205 A11 SCM saki	pGEM	7743.97	442.97	26.93	0.08
comf2E4	pGEM	19724.09	3624.89	25.57	0.27

Plate Layout

	1	2	3	4	5	6	7	8	9	10	11	12
A	S pGEM<br>2E6<br>FAM-NFQ-MGB<br>CT: 18.98	S pGEM<br>2E6<br>FAM-NFQ-MGB<br>CT: 18.71		2205 A11 10m saki<br>U pGEM<br>8.94E3<br>FAM-NFQ-MGB<br>CT: 26.71	2205 A11 10m saki<br>U pGEM<br>9.33E3<br>FAM-NFQ-MGB<br>CT: 26.65	2205 A11 10m saki<br>U pGEM<br>9.19E3<br>FAM-NFQ-MGB<br>CT: 26.67		2205 A11 3000m saki<br>U pGEM<br>6.44E3<br>FAM-NFQ-MGB<br>CT: 27.19	2205 A11 3000m saki<br>U pGEM<br>7.03E3<br>FAM-NFQ-MGB<br>CT: 27.07	2205 A11 3000m saki<br>U pGEM<br>7.34E3<br>FAM-NFQ-MGB<br>CT: 27		
B	S pGEM<br>2E5<br>FAM-NFQ-MGB<br>CT: 21.97	S pGEM<br>2E5<br>FAM-NFQ-MGB<br>CT: 21.99		2205 A11 10m ato<br>U pGEM<br>1.94E4<br>FAM-NFQ-MGB<br>CT: 25.58	2205 A11 10m ato<br>U pGEM<br>1.99E4<br>FAM-NFQ-MGB<br>CT: 25.54	2205 A11 10m ato<br>U pGEM<br>2.25E4<br>FAM-NFQ-MGB<br>CT: 25.36		2205 A11 3000m ato<br>U pGEM<br>1.79E4<br>FAM-NFQ-MGB<br>CT: 25.7	2205 A11 3000m ato<br>U pGEM<br>1.79E4<br>FAM-NFQ-MGB<br>CT: 25.7	2205 A11 3000m ato<br>U pGEM<br>2.05E4<br>FAM-NFQ-MGB<br>CT: 25.5		
C	S pGEM<br>2E4<br>FAM-NFQ-MGB<br>CT: 25.59	S pGEM<br>2E4<br>FAM-NFQ-MGB<br>CT: 25.38		2205 A11 SCM saki<br>U pGEM<br>8.19E3<br>FAM-NFQ-MGB<br>CT: 26.84	2205 A11 SCM saki<br>U pGEM<br>7.74E3<br>FAM-NFQ-MGB<br>CT: 26.93	2205 A11 SCM saki<br>U pGEM<br>7.31E3<br>FAM-NFQ-MGB<br>CT: 27.01						
D	S pGEM<br>2E3<br>FAM-NFQ-MGB<br>CT: 28.95	S pGEM<br>2E3<br>FAM-NFQ-MGB<br>CT: 29.08		2205 A11 SCM ato<br>U pGEM<br>1.65E4<br>FAM-NFQ-MGB<br>CT: 25.81	2205 A11 SCM ato<br>U pGEM<br>1.89E4<br>FAM-NFQ-MGB<br>CT: 25.62	2205 A11 SCM ato<br>U pGEM<br>1.97E4<br>FAM-NFQ-MGB<br>CT: 25.56						
E	S pGEM<br>200<br>FAM-NFQ-MGB<br>CT: 32.64	S pGEM<br>200<br>FAM-NFQ-MGB<br>CT: 32.65		2205 A11 200m saki<br>U pGEM<br>7.22E3<br>FAM-NFQ-MGB<br>CT: 27.03	2205 A11 200m saki<br>U pGEM<br>6.75E3<br>FAM-NFQ-MGB<br>CT: 27.13	2205 A11 200m saki<br>U pGEM<br>6.59E3<br>FAM-NFQ-MGB<br>CT: 27.16						
F	S pGEM<br>20<br>FAM-NFQ-MGB<br>CT: 35.01	S pGEM<br>20<br>FAM-NFQ-MGB<br>CT: 35.69		2205 A11 200m ato<br>U pGEM<br>1.68E4<br>FAM-NFQ-MGB<br>CT: 25.79	2205 A11 200m ato<br>U pGEM<br>1.96E4<br>FAM-NFQ-MGB<br>CT: 25.56	2205 A11 200m ato<br>U pGEM<br>1.97E4<br>FAM-NFQ-MGB<br>CT: 25.55						
G				2205 A11 1000m saki<br>U pGEM<br>1.08E4<br>FAM-NFQ-MGB<br>CT: 26.43	2205 A11 1000m saki<br>U pGEM<br>1.15E4<br>FAM-NFQ-MGB<br>CT: 26.35	2205 A11 1000m saki<br>U pGEM<br>8.73E3<br>FAM-NFQ-MGB<br>CT: 26.75						
H	comf2E4<br>U pGEM<br>1.72E4<br>FAM-NFQ-MGB<br>CT: 25.76	comf2E4<br>U pGEM<br>2.23E4<br>FAM-NFQ-MGB<br>CT: 25.38		2205 A11 1000m ato<br>U pGEM<br>1.94E4<br>FAM-NFQ-MGB<br>CT: 25.58	2205 A11 1000m ato<br>U pGEM<br>2.05E4<br>FAM-NFQ-MGB<br>CT: 25.5	2205 A11 1000m ato<br>U pGEM<br>2.15E4<br>FAM-NFQ-MGB<br>CT: 25.43					N pGEM<br>FAM-NFQ-MGB<br>CT: Undetermined	N pGEM<br>FAM-NFQ-MGB<br>CT: Undetermined

Standard Curves

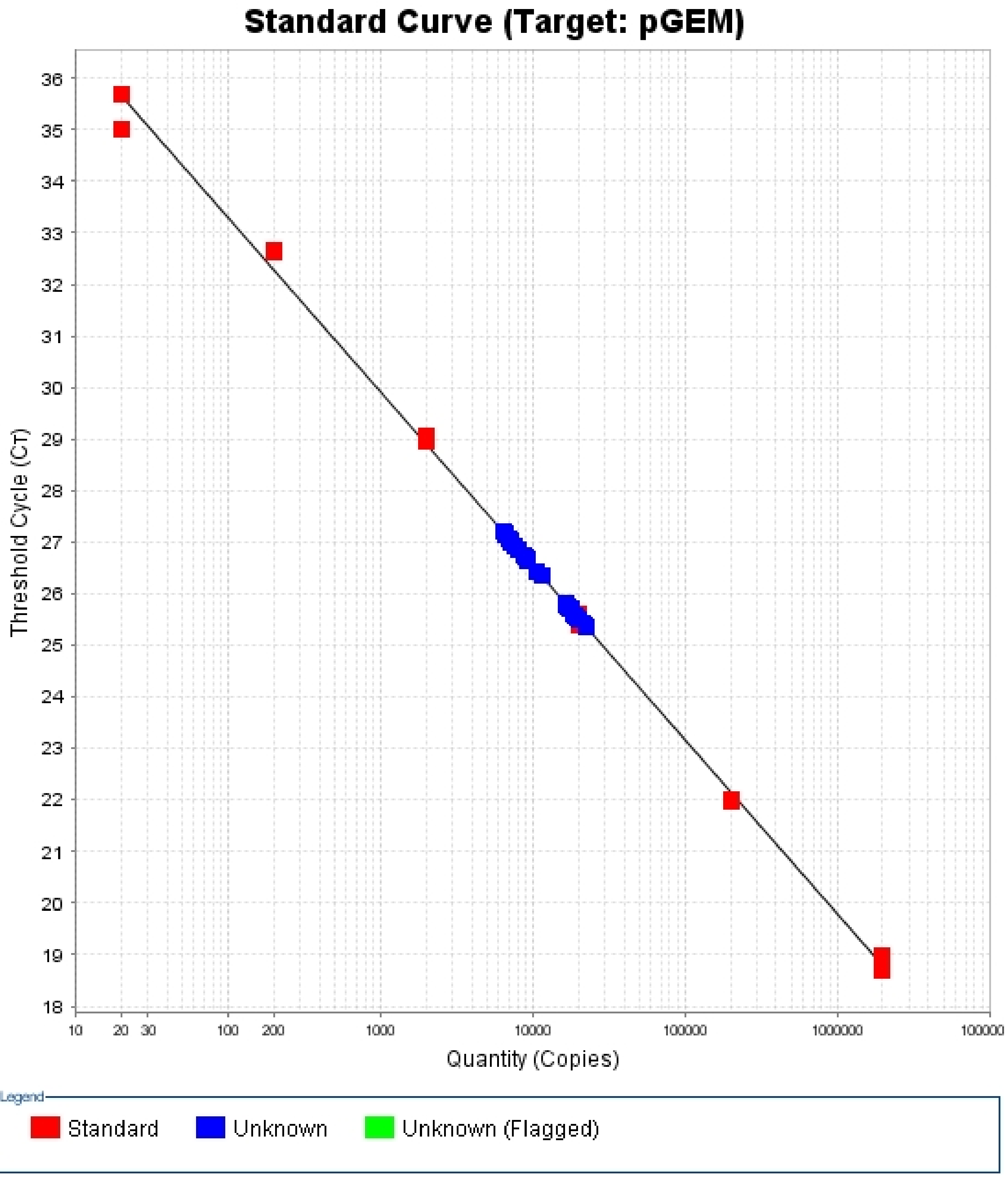

slope:-3.373

Y-Intercept:40.042

R^2:0.998

Eff%:97.912

Results Table

Well		Sample	Target	Task	Quantity	C
A1			pGEM	STANDARD	2000000	18.9761
A2			pGEM	STANDARD	2000000	18.7121
A4		2205 A11 10m	pGEM	UNKNOWN	8940.9199	26.7144
A5		2205 A11 10m	pGEM	UNKNOWN	9326.7217	26.6525
A6		2205 A11 10m	pGEM	UNKNOWN	9192.0801	26.6738
A8		2205 A11	pGEM	UNKNOWN	6441.0552	27.1948
A9		2205 A11	pGEM	UNKNOWN	7026.3389	27.0674
A10		2205 A11	pGEM	UNKNOWN	7343.8174	27.0026
B1			pGEM	STANDARD	200000	21.9747
B2			pGEM	STANDARD	200000	21.9938
B4		2205 A11 10m	pGEM	UNKNOWN	19417.6602	25.5783
B5		2205 A11 10m	pGEM	UNKNOWN	19914.4199	25.5413
B6		2205 A11 10m	pGEM	UNKNOWN	22478.9258	25.3639
B8		2205 A11	pGEM	UNKNOWN	17897.877	25.6977
B9		2205 A11	pGEM	UNKNOWN	17854.6309	25.7012
B10		2205 A11	pGEM	UNKNOWN	20472.8789	25.5008
C1			pGEM	STANDARD	20000	25.59
C2			pGEM	STANDARD	20000	25.3824
C4		2205 A11 SCM	pGEM	UNKNOWN	8191.1509	26.8427
C5		2205 A11 SCM	pGEM	UNKNOWN	7735.438	26.9265
C6		2205 A11 SCM	pGEM	UNKNOWN	7305.3359	27.0103
D1			pGEM	STANDARD	2000	28.9524
D2			pGEM	STANDARD	2000	29.0758
D4		2205 A11 SCM	pGEM	UNKNOWN	16524.8203	25.8146
D5		2205 A11 SCM	pGEM	UNKNOWN	18881.8125	25.6193
D6		2205 A11 SCM	pGEM	UNKNOWN	19651.6348	25.5608
E1			pGEM	STANDARD	200	32.6401
E2			pGEM	STANDARD	200	32.6535
E4		2205 A11 200m	pGEM	UNKNOWN	7224.4033	27.0267
E5		2205 A11 200m	pGEM	UNKNOWN	6745.0918	27.1272

Well		Sample	Target	Task	Quantity	C
E6		2205 A11 200m	pGEM	UNKNOWN	6588.4932	27.1616
F1			pGEM	STANDARD	20	35.0144
F2			pGEM	STANDARD	20	35.6904
F4		2205 A11 200m	pGEM	UNKNOWN	16839.7031	25.787
F5		2205 A11 200m	pGEM	UNKNOWN	19625.2715	25.5627
F6		2205 A11 200m	pGEM	UNKNOWN	19745.6895	25.5538
G4		2205 A11	pGEM	UNKNOWN	10842.2373	26.4319
G5		2205 A11	pGEM	UNKNOWN	11478.2734	26.3484
G6		2205 A11	pGEM	UNKNOWN	8726.1348	26.75
H1		comf2E4	pGEM	UNKNOWN	17160.9004	25.7593
H2		comf2E4	pGEM	UNKNOWN	22287.2754	25.3764
H4		2205 A11	pGEM	UNKNOWN	19376.2402	25.5814
H5		2205 A11	pGEM	UNKNOWN	20484.7715	25.4999
H6		2205 A11	pGEM	UNKNOWN	21460.541	25.4318
H11			pGEM	NTC		Undetermined
H12			pGEM	NTC		Undetermined

QC Summary

Total Wells	96	Processed Wells	46	Targets Used	1
Well Setup	46	Flagged Wells	0	Samples Used	11

Flag	Name	Frequency	Locations
AMPNC	Amplification in negative control	0	
BADROX	Bad passive reference signal	0	
BLFAIL	Baseline algorithm failed	0	
CTFAIL	C algorithm failed	0	
EXPFAIL	Exponential algorithm failed	0	
HIGHSD	High standard deviation in replicate group	0	
NOAMP	No amplification	0	
NOISE	Noise higher than others in plate	0	
NOSIGNAL	No signal in well	0	
OFFSCALE	Fluorescence is offscale	0	
OUTLIERRG	Outlier in replicate group	0	
SPIKE	Noise spikes	0	
THOLDFAIL	Thresholding algorithm failed	0	
