# Supplementary material for: Ecological impact of algal-preying protists aplanochytrids (Labyrinthulea) as a source of docosahexaenoic acid for copepods in marine environments
Source: ISME Commun. 2026 Jun 26;6(1):ycag178. doi: 10.1093/ismeco/ycag178 (PMC13685785; doi:10.1093/ismeco/ycag178)
Supplement: Supplementary file 2 [file supplementary_materials_ycag178.zip › Morimoto_ISME_C_3rdrev_supplemental_data.zip!/Morimoto_ISME_C_3rdrev_supplemental_data/Supplemental_Data_2/Standard Curve/DNA recovery/2205/DnaRec2205AlineA01_N=1.pdf]

Experiment Results Report
230301DnaRecEmm2205AlineA01byKurabo

Experiment Summary

Experiment Name	:	230301DnaRecEmm2205AlineA01byKurabo
Experiment Type	:	Quantitation - Standard Curve
File Name	:	230301DnaRecEmm2205AlineA01byKurabo.eds
Run Started	:	2023 Mar 01 5:19:33 PM
Run Finished	:	2023 Mar 01 6:54:06 PM
Run Duration	:	94 minutes 33 seconds
Date Modified	:	2023 Mar 14 12:27:13 PM
User	:	
Number of wells used	:	34
Number of wells with results	:	34
Instrument Name	:	
Instrument Type	:	Applied Biosystems StepOnePlus™ Instrument
Comments	:	

Results Summary

Sample	Target	Quantity (Mean)	Quantity (Std Dev)	C (Mean)	C (Std Dev)
2205 A01 10m ato	pGEM	19036.96	967.28	25.43	0.08
2205 A01 10m saki	pGEM	8469.16	42.58	26.64	0.01
2205 A01 BTM ato	pGEM	19335.69	1310.11	25.41	0.10
2205 A01 BTM saki	pGEM	10935.13	153.30	26.26	0.02
2205 A01 SCM ato	pGEM	18462.56	1237.57	25.48	0.10
2205 A01 SCM saki	pGEM	10437.43	276.07	26.33	0.04
comf2E4	pGEM	20733.73	2695.20	25.31	0.19

Plate Layout

	1	2	3	4	5	6	7	8	9	10	11	12
A	S pGEM<br>2E6<br>FAM-NFQ-MGB<br>CT: 18.82	S pGEM<br>2E6<br>FAM-NFQ-MGB<br>CT: 18.63		2205 A01 10m saki<br>U pGEM<br>8.43E3<br>FAM-NFQ-MGB<br>CT: 26.64	2205 A01 10m saki<br>U pGEM<br>8.47E3<br>FAM-NFQ-MGB<br>CT: 26.64	2205 A01 10m saki<br>U pGEM<br>8.51E3<br>FAM-NFQ-MGB<br>CT: 26.63						
B	S pGEM<br>2E5<br>FAM-NFQ-MGB<br>CT: 21.76	S pGEM<br>2E5<br>FAM-NFQ-MGB<br>CT: 21.88		2205 A01 10m ato<br>U pGEM<br>1.91E4<br>FAM-NFQ-MGB<br>CT: 25.43	2205 A01 10m ato<br>U pGEM<br>1.8E4<br>FAM-NFQ-MGB<br>CT: 25.51	2205 A01 10m ato<br>U pGEM<br>2E4<br>FAM-NFQ-MGB<br>CT: 25.36						
C	S pGEM<br>2E4<br>FAM-NFQ-MGB<br>CT: 25.27	S pGEM<br>2E4<br>FAM-NFQ-MGB<br>CT: 25.21		2205 A01 SCM saki<br>U pGEM<br>1.05E4<br>FAM-NFQ-MGB<br>CT: 26.32	2205 A01 SCM saki<br>U pGEM<br>1.07E4<br>FAM-NFQ-MGB<br>CT: 26.29	2205 A01 SCM saki<br>U pGEM<br>1.02E4<br>FAM-NFQ-MGB<br>CT: 26.37						
D	S pGEM<br>2E3<br>FAM-NFQ-MGB<br>CT: 28.68	S pGEM<br>2E3<br>FAM-NFQ-MGB<br>CT: 28.65		2205 A01 SCM ato<br>U pGEM<br>1.7E4<br>FAM-NFQ-MGB<br>CT: 25.6	2205 A01 SCM ato<br>U pGEM<br>1.9E4<br>FAM-NFQ-MGB<br>CT: 25.43	2205 A01 SCM ato<br>U pGEM<br>1.93E4<br>FAM-NFQ-MGB<br>CT: 25.41						
E	S pGEM<br>200<br>FAM-NFQ-MGB<br>CT: 32.29	S pGEM<br>200<br>FAM-NFQ-MGB<br>CT: 32.16		2205 A01 BTM saki<br>U pGEM<br>1.09E4<br>FAM-NFQ-MGB<br>CT: 26.27	2205 A01 BTM saki<br>U pGEM<br>1.11E4<br>FAM-NFQ-MGB<br>CT: 26.23	2205 A01 BTM saki<br>U pGEM<br>1.08E4<br>FAM-NFQ-MGB<br>CT: 26.27						
F	S pGEM<br>20<br>FAM-NFQ-MGB<br>CT: 35.98	S pGEM<br>20<br>FAM-NFQ-MGB<br>CT: 35.54		2205 A01 BTM ato<br>U pGEM<br>1.82E4<br>FAM-NFQ-MGB<br>CT: 25.5	2205 A01 BTM ato<br>U pGEM<br>2.08E4<br>FAM-NFQ-MGB<br>CT: 25.3	2205 A01 BTM ato<br>U pGEM<br>1.9E4<br>FAM-NFQ-MGB<br>CT: 25.43						
G												
H	comf2E4<br>U pGEM<br>1.88E4<br>FAM-NFQ-MGB<br>CT: 25.45	comf2E4<br>U pGEM<br>2.26E4<br>FAM-NFQ-MGB<br>CT: 25.18									N pGEM<br>FAM-NFQ-MGB<br>CT: Undetermined	N pGEM<br>FAM-NFQ-MGB<br>CT: Undetermined

Standard Curves

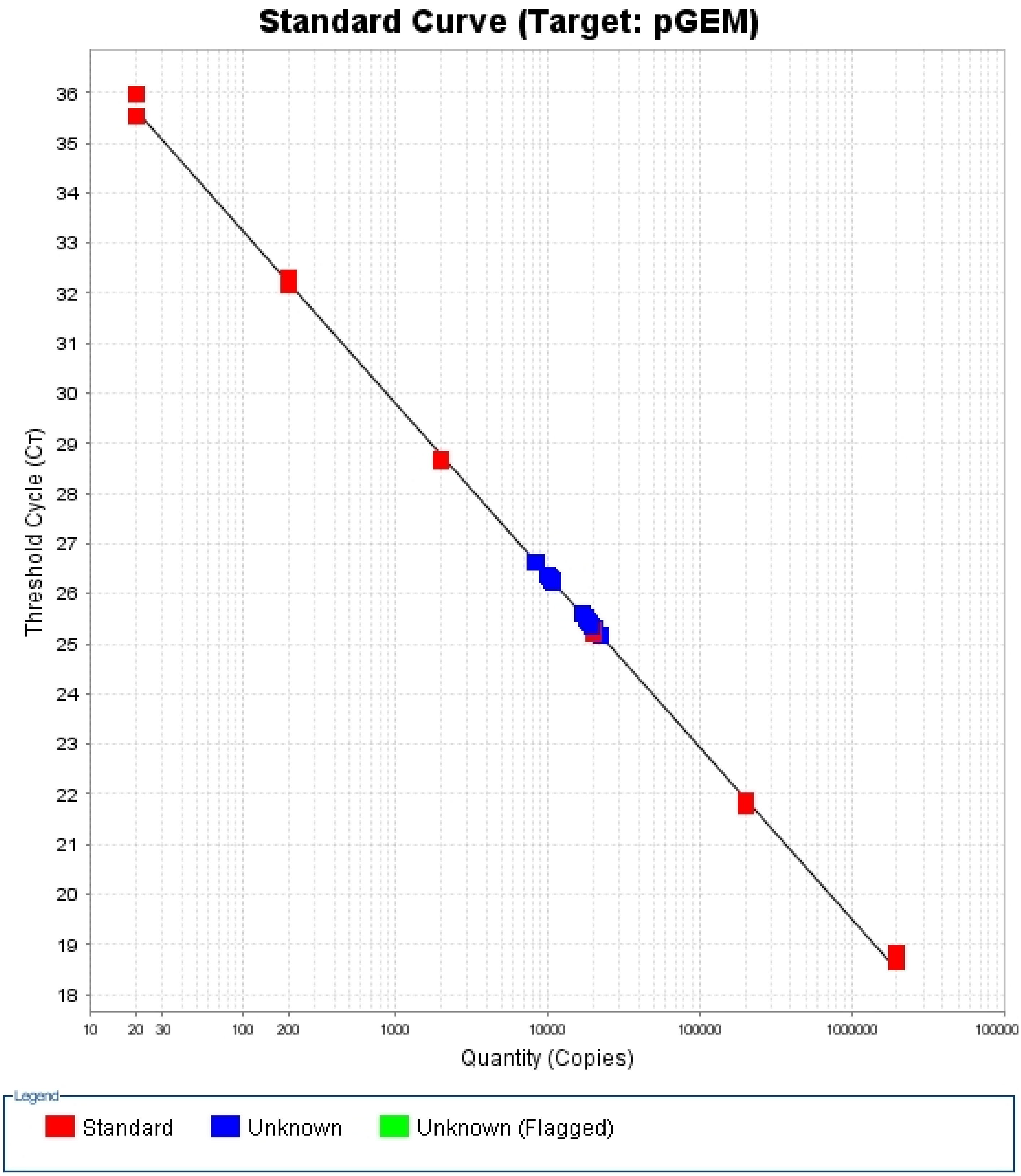

slope:-3.423 Y-Intercept:40.083 R^2:0.999 Eff%:95.944

Results Table

Well		Sample	Target	Task	Quantity	C
A1			pGEM	STANDARD	2000000	18.8178
A2			pGEM	STANDARD	2000000	18.631
A4		2205 A01 10m	pGEM	UNKNOWN	8427.917	26.6449
A5		2205 A01 10m	pGEM	UNKNOWN	8466.6191	26.6381
A6		2205 A01 10m	pGEM	UNKNOWN	8512.9551	26.6299
B1			pGEM	STANDARD	200000	21.7586
B2			pGEM	STANDARD	200000	21.8821
B4		2205 A01 10m	pGEM	UNKNOWN	19097.502	25.4288
B5		2205 A01 10m	pGEM	UNKNOWN	18040.8262	25.5134
B6		2205 A01 10m	pGEM	UNKNOWN	19972.5449	25.3622
C1			pGEM	STANDARD	20000	25.2679
C2			pGEM	STANDARD	20000	25.207
C4		2205 A01 SCM	pGEM	UNKNOWN	10459.6592	26.3238
C5		2205 A01 SCM	pGEM	UNKNOWN	10701.7178	26.2898
C6		2205 A01 SCM	pGEM	UNKNOWN	10150.9287	26.3683
D1			pGEM	STANDARD	2000	28.6769
D2			pGEM	STANDARD	2000	28.6484
D4		2205 A01 SCM	pGEM	UNKNOWN	17042.377	25.598
D5		2205 A01 SCM	pGEM	UNKNOWN	19035.1973	25.4336
D6		2205 A01 SCM	pGEM	UNKNOWN	19310.1055	25.4123
E1			pGEM	STANDARD	200	32.2931
E2			pGEM	STANDARD	200	32.1566
E4		2205 A01 BTM	pGEM	UNKNOWN	10875.6367	26.2658
E5		2205 A01 BTM	pGEM	UNKNOWN	11109.2637	26.2342
E6		2205 A01 BTM	pGEM	UNKNOWN	10820.4941	26.2734
F1			pGEM	STANDARD	20	35.9756
F2			pGEM	STANDARD	20	35.5423
F4		2205 A01 BTM	pGEM	UNKNOWN	18202.4355	25.5002
F5		2205 A01 BTM	pGEM	UNKNOWN	20770.1797	25.304
F6		2205 A01 BTM	pGEM	UNKNOWN	19034.4395	25.4337

Well		Sample	Target	Task	Quantity	C
H1		comf2E4	pGEM	UNKNOWN	18827.9395	25.4499
H2		comf2E4	pGEM	UNKNOWN	22639.5254	25.1759
H11			pGEM	NTC		Undetermined
H12			pGEM	NTC		Undetermined

QC Summary

Total Wells	96	Processed Wells	34	Targets Used	1
Well Setup	34	Flagged Wells	0	Samples Used	7

Flag	Name	Frequency	Locations
AMPNC	Amplification in negative control	0	
BADROX	Bad passive reference signal	0	
BLFAIL	Baseline algorithm failed	0	
CTFAIL	C algorithm failed	0	
EXPFAIL	Exponential algorithm failed	0	
HIGHSD	High standard deviation in replicate group	0	
NOAMP	No amplification	0	
NOISE	Noise higher than others in plate	0	
NOSIGNAL	No signal in well	0	
OFFSCALE	Fluorescence is offscale	0	
OUTLIERRG	Outlier in replicate group	0	
SPIKE	Noise spikes	0	
THOLDFAIL	Thresholding algorithm failed	0	
