# Supplementary material for: Ecological impact of algal-preying protists aplanochytrids (Labyrinthulea) as a source of docosahexaenoic acid for copepods in marine environments
Source: ISME Commun. 2026 Jun 26;6(1):ycag178. doi: 10.1093/ismeco/ycag178 (PMC13685785; doi:10.1093/ismeco/ycag178)
Supplement: Supplementary file 2 [file supplementary_materials_ycag178.zip › Morimoto_ISME_C_3rdrev_supplemental_data.zip!/Morimoto_ISME_C_3rdrev_supplemental_data/Supplemental_Data_2/Standard Curve/DNA recovery/2205/DnaRecAline2205A07_N=3.pdf]

Experiment Results Report

230320DnaRecAline2205A07DnaExtN=3EmmByKurabo

Experiment Summary

Experiment Name	:	230320DnaRecAline2205A07DnaExtN=3EmmByKurabo
Experiment Type	:	Quantitation - Standard Curve
File Name	:	230320DnaRecAline2205A07DnaExtN=3EmmByKurabo.eds
Run Started	:	2023 Mar 20 12:51:05 PM
Run Finished	:	2023 Mar 20 2:25:39 PM
Run Duration	:	94 minutes 34 seconds
Date Modified	:	2023 Mar 20 3:05:27 PM
User	:	
Number of wells used	:	70
Number of wells with results	:	70
Instrument Name	:	
Instrument Type	:	Applied Biosystems StepOnePlus™ Instrument
Comments	:	

Results Summary

Sample	Target	Quantity (Mean)	Quantity (Std Dev)	C (Mean)	C (Std Dev)
2205 A07 1000m	pGEM	27216.92	1119.00	24.82	0.07
2205 A07 1000m	pGEM	27912.08	482.58	24.78	0.03
2205 A07 1000m	pGEM	18801.34	1599.63	25.41	0.14
2205 A07 1000m	pGEM	18581.11	1485.30	25.43	0.13
2205 A07 10m ato2	pGEM	28018.82	1234.34	24.78	0.07
2205 A07 10m ato3	pGEM	28270.74	1746.12	24.76	0.10
2205 A07 10m saki2	pGEM	14814.75	660.25	25.79	0.07
2205 A07 10m saki3	pGEM	17687.13	1694.86	25.51	0.15
2205 A07 200m ato2	pGEM	26620.70	1356.22	24.86	0.08
2205 A07 200m ato3	pGEM	27899.73	247.96	24.78	0.01
2205 A07 200m	pGEM	19579.02	304.34	25.35	0.02
2205 A07 200m	pGEM	19323.08	1494.42	25.37	0.12
2205 A07 3000m	pGEM	28811.62	1164.21	24.73	0.06
2205 A07 3000m	pGEM	14995.68	880.59	25.77	0.09
2205 A07 SCM ato2	pGEM	29808.34	634.10	24.68	0.03
2205 A07 SCM ato3	pGEM	27800.55	2097.79	24.79	0.12
2205 A07 SCM saki2	pGEM	12630.73	662.49	26.05	0.08
2205 A07 SCM saki3	pGEM	17535.54	1650.06	25.53	0.15
Comf2E4	pGEM	24901.01	2245.91	24.97	0.14

Plate Layout

	1	2	3	4	5	6	7	8	9	10	11	12
A	S pGEM<br>2E6<br>FAM-NFQ-MGB<br>CT: 18.37	S pGEM<br>2E6<br>FAM-NFQ-MGB<br>CT: 18.32		2205 A07 10m saki2<br>U pGEM<br>1.47E4<br>FAM-NFQ-MGB<br>CT: 25.8	2205 A07 10m saki2<br>U pGEM<br>1.42E4<br>FAM-NFQ-MGB<br>CT: 25.86	2205 A07 10m saki2<br>U pGEM<br>1.55E4<br>FAM-NFQ-MGB<br>CT: 25.72	2205 A07 3000m saki2<br>U pGEM<br>1.4E4<br>FAM-NFQ-MGB<br>CT: 25.88	2205 A07 10m saki3<br>U pGEM<br>1.62E4<br>FAM-NFQ-MGB<br>CT: 25.65	2205 A07 10m saki3<br>U pGEM<br>1.73E4<br>FAM-NFQ-MGB<br>CT: 25.54	2205 A07 10m saki3<br>U pGEM<br>1.95E4<br>FAM-NFQ-MGB<br>CT: 25.35		
B	S pGEM<br>2E5<br>FAM-NFQ-MGB<br>CT: 21.65	S pGEM<br>2E5<br>FAM-NFQ-MGB<br>CT: 21.42		2205 A07 10m ato2<br>U pGEM<br>2.79E4<br>FAM-NFQ-MGB<br>CT: 24.78	2205 A07 10m ato2<br>U pGEM<br>2.68E4<br>FAM-NFQ-MGB<br>CT: 24.84	2205 A07 10m ato2<br>U pGEM<br>2.93E4<br>FAM-NFQ-MGB<br>CT: 24.7	2205 A07 3000m saki2<br>U pGEM<br>1.52E4<br>FAM-NFQ-MGB<br>CT: 25.75	2205 A07 10m ato3<br>U pGEM<br>2.64E4<br>FAM-NFQ-MGB<br>CT: 24.87	2205 A07 10m ato3<br>U pGEM<br>2.86E4<br>FAM-NFQ-MGB<br>CT: 24.74	2205 A07 10m ato3<br>U pGEM<br>2.98E4<br>FAM-NFQ-MGB<br>CT: 24.68		
C	S pGEM<br>2E4<br>FAM-NFQ-MGB<br>CT: 25.03	S pGEM<br>2E4<br>FAM-NFQ-MGB<br>CT: 24.97		2205 A07 SCM saki2<br>U pGEM<br>1.23E4<br>FAM-NFQ-MGB<br>CT: 26.09	2205 A07 SCM saki2<br>U pGEM<br>1.34E4<br>FAM-NFQ-MGB<br>CT: 25.95	2205 A07 SCM saki2<br>U pGEM<br>1.22E4<br>FAM-NFQ-MGB<br>CT: 26.09	2205 A07 3000m saki2<br>U pGEM<br>1.57E4<br>FAM-NFQ-MGB<br>CT: 25.69	2205 A07 SCM saki3<br>U pGEM<br>1.76E4<br>FAM-NFQ-MGB<br>CT: 25.52	2205 A07 SCM saki3<br>U pGEM<br>1.59E4<br>FAM-NFQ-MGB<br>CT: 25.68	2205 A07 SCM saki3<br>U pGEM<br>1.91E4<br>FAM-NFQ-MGB<br>CT: 25.38		
D	S pGEM<br>2E3<br>FAM-NFQ-MGB<br>CT: 28.38	S pGEM<br>2E3<br>FAM-NFQ-MGB<br>CT: 28.53		2205 A07 SCM ato2<br>U pGEM<br>3.05E4<br>FAM-NFQ-MGB<br>CT: 24.64	2205 A07 SCM ato2<br>U pGEM<br>2.95E4<br>FAM-NFQ-MGB<br>CT: 24.69	2205 A07 SCM ato2<br>U pGEM<br>2.94E4<br>FAM-NFQ-MGB<br>CT: 24.7	2205 A07 3000m ato2<br>U pGEM<br>2.84E4<br>FAM-NFQ-MGB<br>CT: 24.76	2205 A07 SCM ato3<br>U pGEM<br>2.54E4<br>FAM-NFQ-MGB<br>CT: 24.93	2205 A07 SCM ato3<br>U pGEM<br>2.86E4<br>FAM-NFQ-MGB<br>CT: 24.74	2205 A07 SCM ato3<br>U pGEM<br>2.94E4<br>FAM-NFQ-MGB<br>CT: 24.7		
E	S pGEM<br>200<br>FAM-NFQ-MGB<br>CT: 33.5	S pGEM<br>200<br>FAM-NFQ-MGB<br>CT: 33.08		2205 A07 200m saki2<br>U pGEM<br>1.99E4<br>FAM-NFQ-MGB<br>CT: 25.32	2205 A07 200m saki2<br>U pGEM<br>1.93E4<br>FAM-NFQ-MGB<br>CT: 25.37	2205 A07 200m saki2<br>U pGEM<br>1.96E4<br>FAM-NFQ-MGB<br>CT: 25.35	2205 A07 3000m ato2<br>U pGEM<br>2.79E4<br>FAM-NFQ-MGB<br>CT: 24.78	2205 A07 200m saki3<br>U pGEM<br>1.81E4<br>FAM-NFQ-MGB<br>CT: 25.47	2205 A07 200m saki3<br>U pGEM<br>2.1E4<br>FAM-NFQ-MGB<br>CT: 25.24	2205 A07 200m saki3<br>U pGEM<br>1.89E4<br>FAM-NFQ-MGB<br>CT: 25.4		
F	1<br>S pGEM<br>20<br>FAM-NFQ-MGB<br>CT: 36.97	1<br>S pGEM<br>20<br>FAM-NFQ-MGB<br>CT: 35.5		2205 A07 200m ato2<br>U pGEM<br>2.54E4<br>FAM-NFQ-MGB<br>CT: 24.93	2205 A07 200m ato2<br>U pGEM<br>2.81E4<br>FAM-NFQ-MGB<br>CT: 24.77	2205 A07 200m ato2<br>U pGEM<br>2.64E4<br>FAM-NFQ-MGB<br>CT: 24.87	2205 A07 3000m ato2<br>U pGEM<br>3.01E4<br>FAM-NFQ-MGB<br>CT: 24.66	2205 A07 200m ato3<br>U pGEM<br>2.76E4<br>FAM-NFQ-MGB<br>CT: 24.8	2205 A07 200m ato3<br>U pGEM<br>2.81E4<br>FAM-NFQ-MGB<br>CT: 24.77	2205 A07 200m ato3<br>U pGEM<br>2.8E4<br>FAM-NFQ-MGB<br>CT: 24.78		
G				2205 A07 1000m saki2<br>U pGEM<br>1.97E4<br>FAM-NFQ-MGB<br>CT: 25.34	2205 A07 1000m saki2<br>U pGEM<br>1.97E4<br>FAM-NFQ-MGB<br>CT: 25.33	2205 A07 1000m saki2<br>U pGEM<br>1.7E4<br>FAM-NFQ-MGB<br>CT: 25.58		2205 A07 1000m saki3<br>U pGEM<br>1.71E4<br>FAM-NFQ-MGB<br>CT: 25.56	2205 A07 1000m saki3<br>U pGEM<br>1.85E4<br>FAM-NFQ-MGB<br>CT: 25.43	2205 A07 1000m saki3<br>U pGEM<br>2.01E4<br>FAM-NFQ-MGB<br>CT: 25.31		N pGEM<br>FAM-NFQ-MGB<br>CT: Undetermined
H	Comf2E4<br>U pGEM<br>2.33E4<br>FAM-NFQ-MGB<br>CT: 25.07	Comf2E4<br>U pGEM<br>2.65E4<br>FAM-NFQ-MGB<br>CT: 24.87		2205 A07 1000m ato2<br>U pGEM<br>2.74E4<br>FAM-NFQ-MGB<br>CT: 24.81	2205 A07 1000m ato2<br>U pGEM<br>2.6E4<br>FAM-NFQ-MGB<br>CT: 24.89	2205 A07 1000m ato2<br>U pGEM<br>2.83E4<br>FAM-NFQ-MGB<br>CT: 24.76		2205 A07 1000m ato3<br>U pGEM<br>2.8E4<br>FAM-NFQ-MGB<br>CT: 24.78	2205 A07 1000m ato3<br>U pGEM<br>2.74E4<br>FAM-NFQ-MGB<br>CT: 24.81	2205 A07 1000m ato3<br>U pGEM<br>2.84E4<br>FAM-NFQ-MGB<br>CT: 24.76		N pGEM<br>FAM-NFQ-MGB<br>CT: Undetermined

Standard Curves

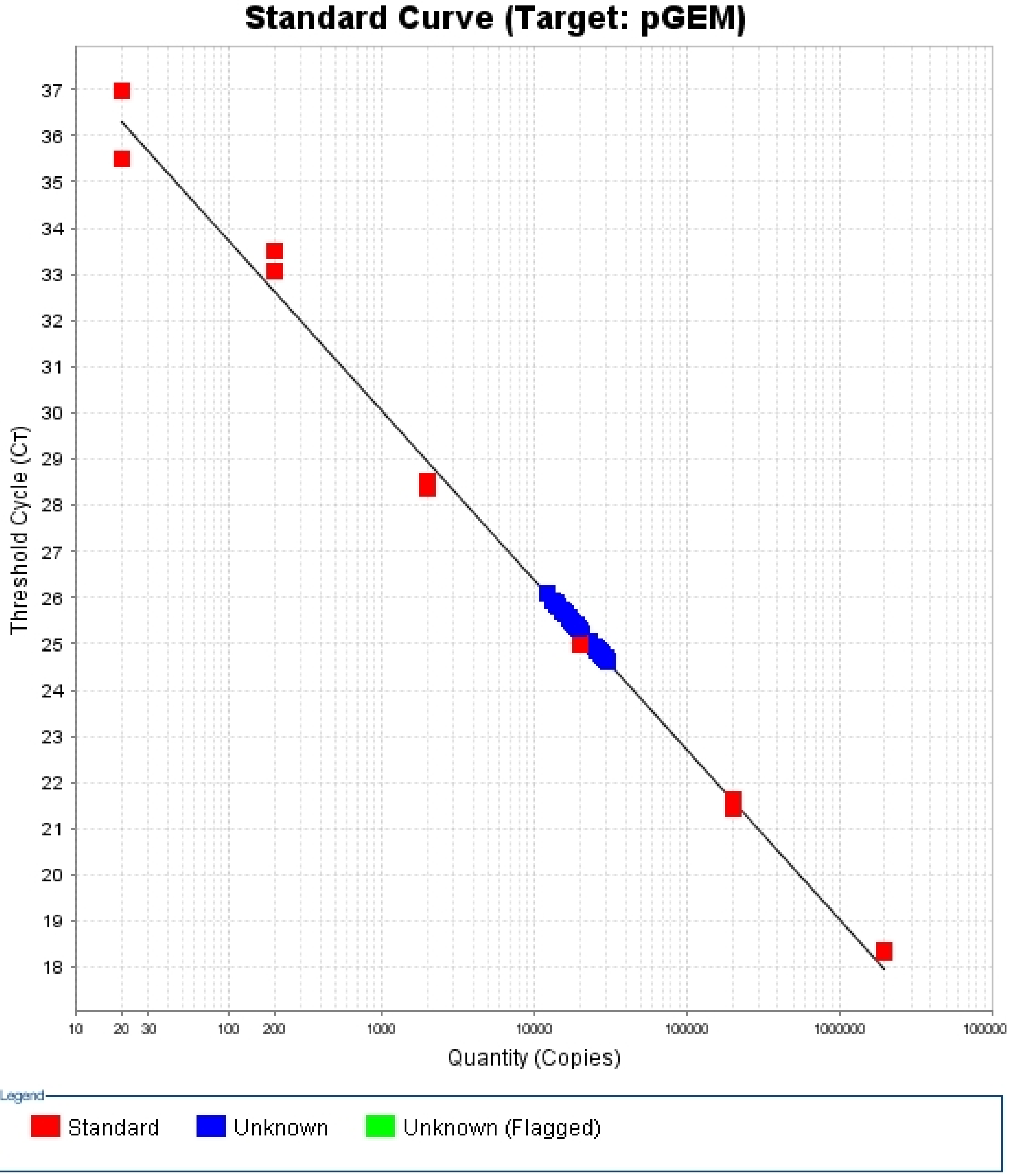

slope:-3.663 Y-Intercept:41.065 R^2:0.993 Eff%:87.512

Results Table

Well		Sample	Target	Task	Quantity	C
A1			pGEM	STANDARD	2000000	18.3653
A2			pGEM	STANDARD	2000000	18.3238
A4		2205 A07 10m	pGEM	UNKNOWN	14699.8701	25.8023
A5		2205 A07 10m	pGEM	UNKNOWN	14219.4785	25.8551
A6		2205 A07 10m	pGEM	UNKNOWN	15524.9092	25.7154
A7		2205 A07	pGEM	UNKNOWN	14019.7148	25.8777
A8		2205 A07 10m	pGEM	UNKNOWN	16225.5107	25.6452
A9		2205 A07 10m	pGEM	UNKNOWN	17290.8789	25.5441
A10		2205 A07 10m	pGEM	UNKNOWN	19545.0137	25.3492
B1			pGEM	STANDARD	200000	21.6506
B2			pGEM	STANDARD	200000	21.4184
B4		2205 A07 10m	pGEM	UNKNOWN	27908.1387	24.7826
B5		2205 A07 10m	pGEM	UNKNOWN	26843.5488	24.8444
B6		2205 A07 10m	pGEM	UNKNOWN	29304.7793	24.7049
B7		2205 A07	pGEM	UNKNOWN	15236.5605	25.7453
B8		2205 A07 10m	pGEM	UNKNOWN	26375.1289	24.8724
B9		2205 A07 10m	pGEM	UNKNOWN	28623.6309	24.7423
B10		2205 A07 10m	pGEM	UNKNOWN	29813.4648	24.6775
C1			pGEM	STANDARD	20000	25.0278
C2			pGEM	STANDARD	20000	24.9656
C4		2205 A07 SCM	pGEM	UNKNOWN	12263.2568	26.0906
C5		2205 A07 SCM	pGEM	UNKNOWN	13395.5186	25.9501
C6		2205 A07 SCM	pGEM	UNKNOWN	12233.4268	26.0944
C7		2205 A07	pGEM	UNKNOWN	15730.7578	25.6945
C8		2205 A07 SCM	pGEM	UNKNOWN	17607.7988	25.5152
C9		2205 A07 SCM	pGEM	UNKNOWN	15850.5381	25.6824
C10		2205 A07 SCM	pGEM	UNKNOWN	19148.2852	25.3818
D1			pGEM	STANDARD	2000	28.3815
D2			pGEM	STANDARD	2000	28.5345
D4		2205 A07 SCM	pGEM	UNKNOWN	30537.8281	24.6393

Well		Sample	Target	Task	Quantity	C
D5		2205 A07 SCM	pGEM	UNKNOWN	29498.0488	24.6944
D6		2205 A07 SCM	pGEM	UNKNOWN	29389.1289	24.7003
D7		2205 A07	pGEM	UNKNOWN	28368.1465	24.7566
D8		2205 A07 SCM	pGEM	UNKNOWN	25423.8613	24.9309
D9		2205 A07 SCM	pGEM	UNKNOWN	28583.6719	24.7445
D10		2205 A07 SCM	pGEM	UNKNOWN	29394.1348	24.7
E1			pGEM	STANDARD	200	33.4967
E2			pGEM	STANDARD	200	33.0838
E4		2205 A07 200m	pGEM	UNKNOWN	19876.918	25.3224
E5		2205 A07 200m	pGEM	UNKNOWN	19268.6309	25.3718
E6		2205 A07 200m	pGEM	UNKNOWN	19591.5195	25.3454
E7		2205 A07	pGEM	UNKNOWN	27934.3203	24.7811
E8		2205 A07 200m	pGEM	UNKNOWN	18071.3691	25.4738
E9		2205 A07 200m	pGEM	UNKNOWN	20977.627	25.2366
E10		2205 A07 200m	pGEM	UNKNOWN	18920.252	25.4008
F1			pGEM	STANDARD	20	36.9712
F2			pGEM	STANDARD	20	35.5039
F4		2205 A07 200m	pGEM	UNKNOWN	25416.0586	24.9314
F5		2205 A07 200m	pGEM	UNKNOWN	28089.6035	24.7723
F6		2205 A07 200m	pGEM	UNKNOWN	26356.4434	24.8736
F7		2205 A07	pGEM	UNKNOWN	30132.3984	24.6606
F8		2205 A07 200m	pGEM	UNKNOWN	27614.4043	24.7994
F9		2205 A07 200m	pGEM	UNKNOWN	28063.0742	24.7738
F10		2205 A07 200m	pGEM	UNKNOWN	28021.7148	24.7761
G4		2205 A07	pGEM	UNKNOWN	19717.5371	25.3352
G5		2205 A07	pGEM	UNKNOWN	19732.2246	25.334
G6		2205 A07	pGEM	UNKNOWN	16954.2715	25.5753
G8		2205 A07	pGEM	UNKNOWN	17116.5859	25.5602
G9		2205 A07	pGEM	UNKNOWN	18540.3848	25.4331
G10		2205 A07	pGEM	UNKNOWN	20086.3516	25.3057
G12			pGEM	NTC		Undetermined
H1		Comf2E4	pGEM	UNKNOWN	23312.9141	25.0687

Well		Sample	Target	Task	Quantity	C
H2		Comf2E4	pGEM	UNKNOWN	26489.1035	24.8656
H4		2205 A07	pGEM	UNKNOWN	27366.0566	24.8138
H5		2205 A07	pGEM	UNKNOWN	26030.832	24.8933
H6		2205 A07	pGEM	UNKNOWN	28253.8789	24.763
H8		2205 A07	pGEM	UNKNOWN	27984.7441	24.7782
H9		2205 A07	pGEM	UNKNOWN	27397.2812	24.812
H10		2205 A07	pGEM	UNKNOWN	28354.2031	24.7573
H12			pGEM	NTC		Undetermined

QC Summary

Total Wells	96	Processed Wells	70	Targets Used	1
Well Setup	70	Flagged Wells	2	Samples Used	19

Flag	Name	Frequency	Locations
AMPNC	Amplification in negative control	0	
BADROX	Bad passive reference signal	0	
BLFAIL	Baseline algorithm failed	0	
CTFAIL	C algorithm failed	0	
EXPFAIL	Exponential algorithm failed	0	
HIGHSD	High standard deviation in replicate group	2	F1, F2
NOAMP	No amplification	0	
NOISE	Noise higher than others in plate	0	
NOSIGNAL	No signal in well	0	
OFFSCALE	Fluorescence is offscale	0	
OUTLIERRG	Outlier in replicate group	0	
SPIKE	Noise spikes	0	
THOLDFAIL	Thresholding algorithm failed	0	
