# Supplementary material for: Ecological impact of algal-preying protists aplanochytrids (Labyrinthulea) as a source of docosahexaenoic acid for copepods in marine environments
Source: ISME Commun. 2026 Jun 26;6(1):ycag178. doi: 10.1093/ismeco/ycag178 (PMC13685785; doi:10.1093/ismeco/ycag178)
Supplement: Supplementary file 2 [file supplementary_materials_ycag178.zip › Morimoto_ISME_C_3rdrev_supplemental_data.zip!/Morimoto_ISME_C_3rdrev_supplemental_data/Supplemental_Data_2/Standard Curve/DNA recovery/2205/DnaRecAline2205A21_N=3.pdf]

Experiment Results Report
230320DnaRecAline2205A21EmmByKurabo

Experiment Summary

Experiment Name	:	230320DnaRecAline2205A21EmmByKurabo
Experiment Type	:	Quantitation - Standard Curve
File Name	:	230320DnaRecAline2205A21EmmByKurabo.eds
Run Started	:	2023 Mar 20 6:21:48 PM
Run Finished	:	2023 Mar 20 7:56:27 PM
Run Duration	:	94 minutes 39 seconds
Date Modified	:	2023 Mar 22 11:12:45 AM
User	:	
Number of wells used	:	76
Number of wells with results	:	76
Instrument Name	:	
Instrument Type	:	Applied Biosystems StepOnePlus™ Instrument
Comments	:	

Results Summary

Sample	Target	Quantity (Mean)	Quantity (Std Dev)	C (Mean)	C (Std Dev)
2205 A21 1000m	pGEM	29236.90	1100.50	24.38	0.06
2205 A21 1000m	pGEM	29841.01	464.40	24.35	0.03
2205 A21 1000m	pGEM	18440.63	399.45	25.14	0.04
2205 A21 1000m	pGEM	22718.45	1197.74	24.80	0.09
2205 A21 10m ato2	pGEM	31363.99	1206.38	24.27	0.06
2205 A21 10m ato3	pGEM	31000.70	473.12	24.28	0.03
2205 A21 10m saki2	pGEM	15531.77	602.97	25.42	0.06
2205 A21 10m saki3	pGEM	16852.72	410.13	25.29	0.04
2205 A21 200m ato2	pGEM	27123.95	1638.77	24.51	0.10
2205 A21 200m ato3	pGEM	32842.88	1320.67	24.19	0.07
2205 A21 200m	pGEM	17542.15	659.20	25.22	0.06
2205 A21 200m	pGEM	20392.63	1503.86	24.98	0.12
2205 A21 3000m	pGEM	28934.54	1209.96	24.40	0.07
2205 A21 3000m	pGEM	28899.45	774.96	24.40	0.04
2205 A21 3000m	pGEM	5035.17	917.76	27.29	0.29
2205 A21 3000m	pGEM	20468.66	1444.58	24.97	0.11
2205 A21 SCM ato2	pGEM	28876.94	253.71	24.40	0.01
2205 A21 SCM ato3	pGEM	32139.53	2145.64	24.23	0.11
2205 A21 SCM saki2	pGEM	9925.50	439.16	26.16	0.07
2205 A21 SCM saki3	pGEM	20381.63	1452.75	24.98	0.12
Comf2E4	pGEM	24837.22	3180.31	24.66	0.21

Plate Layout

	1	2	3	4	5	6	7	8	9	10	11	12
A	S pGEM<br>2E6<br>FAM-NFQ-MGB<br>CT: 18.02	S pGEM<br>2E6<br>FAM-NFQ-MGB<br>CT: 17.83		2205 A21 10m saki2<br>U pGEM<br>1.49E4<br>FAM-NFQ-MGB<br>CT: 25.49	2205 A21 10m saki2<br>U pGEM<br>1.55E4<br>FAM-NFQ-MGB<br>CT: 25.42	2205 A21 10m saki2<br>U pGEM<br>1.61E4<br>FAM-NFQ-MGB<br>CT: 25.36	2205 A21 3000m saki2<br>U pGEM<br>4.36E3<br>FAM-NFQ-MGB<br>CT: 27.51	2205 A21 10m saki3<br>U pGEM<br>1.68E4<br>FAM-NFQ-MGB<br>CT: 25.3	2205 A21 10m saki3<br>U pGEM<br>1.73E4<br>FAM-NFQ-MGB<br>CT: 25.24	2205 A21 10m saki3<br>U pGEM<br>1.65E4<br>FAM-NFQ-MGB<br>CT: 25.32	2205 A21 3000m saki3<br>U pGEM<br>1.95E4<br>FAM-NFQ-MGB<br>CT: 25.05	
B	S pGEM<br>2E5<br>FAM-NFQ-MGB<br>CT: 21.13	S pGEM<br>2E5<br>FAM-NFQ-MGB<br>CT: 21.11		2205 A21 10m ato2<br>U pGEM<br>3.12E4<br>FAM-NFQ-MGB<br>CT: 24.27	2205 A21 10m ato2<br>U pGEM<br>3.02E4<br>FAM-NFQ-MGB<br>CT: 24.33	2205 A21 10m ato2<br>U pGEM<br>3.26E4<br>FAM-NFQ-MGB<br>CT: 24.2	2205 A21 3000m saki2<br>U pGEM<br>4.67E3<br>FAM-NFQ-MGB<br>CT: 27.4	2205 A21 10m ato3<br>U pGEM<br>3.05E4<br>FAM-NFQ-MGB<br>CT: 24.31	2205 A21 10m ato3<br>U pGEM<br>3.1E4<br>FAM-NFQ-MGB<br>CT: 24.28	2205 A21 10m ato3<br>U pGEM<br>3.15E4<br>FAM-NFQ-MGB<br>CT: 24.26	2205 A21 3000m saki3<br>U pGEM<br>1.98E4<br>FAM-NFQ-MGB<br>CT: 25.02	
C	S pGEM<br>2E4<br>FAM-NFQ-MGB<br>CT: 24.68	S pGEM<br>2E4<br>FAM-NFQ-MGB<br>CT: 24.58		2205 A21 SCM saki2<br>U pGEM<br>9.54E3<br>FAM-NFQ-MGB<br>CT: 26.22	2205 A21 SCM saki2<br>U pGEM<br>1.04E4<br>FAM-NFQ-MGB<br>CT: 26.08	2205 A21 SCM saki2<br>U pGEM<br>9.83E3<br>FAM-NFQ-MGB<br>CT: 26.17	2205 A21 3000m saki2<br>U pGEM<br>6.08E3<br>FAM-NFQ-MGB<br>CT: 26.96	2205 A21 SCM saki3<br>U pGEM<br>2.03E4<br>FAM-NFQ-MGB<br>CT: 24.98	2205 A21 SCM saki3<br>U pGEM<br>1.9E4<br>FAM-NFQ-MGB<br>CT: 25.09	2205 A21 SCM saki3<br>U pGEM<br>2.19E4<br>FAM-NFQ-MGB<br>CT: 24.86	2205 A21 3000m saki3<br>U pGEM<br>2.21E4<br>FAM-NFQ-MGB<br>CT: 24.84	
D	S pGEM<br>2E3<br>FAM-NFQ-MGB<br>CT: 28.17	S pGEM<br>2E3<br>FAM-NFQ-MGB<br>CT: 28.13		2205 A21 SCM ato2<br>U pGEM<br>2.86E4<br>FAM-NFQ-MGB<br>CT: 24.41	2205 A21 SCM ato2<br>U pGEM<br>2.91E4<br>FAM-NFQ-MGB<br>CT: 24.39	2205 A21 SCM ato2<br>U pGEM<br>2.89E4<br>FAM-NFQ-MGB<br>CT: 24.4	2205 A21 3000m ato2<br>U pGEM<br>2.88E4<br>FAM-NFQ-MGB<br>CT: 24.4	2205 A21 SCM ato3<br>U pGEM<br>2.97E4<br>FAM-NFQ-MGB<br>CT: 24.35	2205 A21 SCM ato3<br>U pGEM<br>3.28E4<br>FAM-NFQ-MGB<br>CT: 24.19	2205 A21 SCM ato3<br>U pGEM<br>3.39E4<br>FAM-NFQ-MGB<br>CT: 24.14	2205 A21 3000m ato3<br>U pGEM<br>2.83E4<br>FAM-NFQ-MGB<br>CT: 24.44	
E	S pGEM<br>200<br>FAM-NFQ-MGB<br>CT: 33.16	S pGEM<br>200<br>FAM-NFQ-MGB<br>CT: 32.67		2205 A21 200m saki2<br>U pGEM<br>1.74E4<br>FAM-NFQ-MGB<br>CT: 25.23	2205 A21 200m saki2<br>U pGEM<br>1.82E4<br>FAM-NFQ-MGB<br>CT: 25.16	2205 A21 200m saki2<br>U pGEM<br>1.69E4<br>FAM-NFQ-MGB<br>CT: 25.28	2205 A21 3000m ato2<br>U pGEM<br>3.02E4<br>FAM-NFQ-MGB<br>CT: 24.33	2205 A21 200m saki3<br>U pGEM<br>1.87E4<br>FAM-NFQ-MGB<br>CT: 25.12	2205 A21 200m saki3<br>U pGEM<br>2.15E4<br>FAM-NFQ-MGB<br>CT: 24.88	2205 A21 200m saki3<br>U pGEM<br>2.1E4<br>FAM-NFQ-MGB<br>CT: 24.93	2205 A21 3000m ato3<br>U pGEM<br>2.87E4<br>FAM-NFQ-MGB<br>CT: 24.41	
F	S pGEM<br>20<br>FAM-NFQ-MGB<br>CT: 36.97	S pGEM<br>20<br>FAM-NFQ-MGB<br>CT: 36.34		2205 A21 200m ato2<br>U pGEM<br>2.53E4<br>FAM-NFQ-MGB<br>CT: 24.62	2205 A21 200m ato2<br>U pGEM<br>2.78E4<br>FAM-NFQ-MGB<br>CT: 24.47	2205 A21 200m ato2<br>U pGEM<br>2.84E4<br>FAM-NFQ-MGB<br>CT: 24.43	2205 A21 3000m ato2<br>U pGEM<br>2.78E4<br>FAM-NFQ-MGB<br>CT: 24.46	2205 A21 200m ato3<br>U pGEM<br>3.17E4<br>FAM-NFQ-MGB<br>CT: 24.24	2205 A21 200m ato3<br>U pGEM<br>3.25E4<br>FAM-NFQ-MGB<br>CT: 24.21	2205 A21 200m ato3<br>U pGEM<br>3.43E4<br>FAM-NFQ-MGB<br>CT: 24.12	2205 A21 3000m ato3<br>U pGEM<br>2.98E4<br>FAM-NFQ-MGB<br>CT: 24.35	
G				2205 A21 1000m saki2<br>U pGEM<br>1.82E4<br>FAM-NFQ-MGB<br>CT: 25.16	2205 A21 1000m saki2<br>U pGEM<br>1.82E4<br>FAM-NFQ-MGB<br>CT: 25.16	2205 A21 1000m saki2<br>U pGEM<br>1.89E4<br>FAM-NFQ-MGB<br>CT: 25.1		2205 A21 1000m saki3<br>U pGEM<br>2.17E4<br>FAM-NFQ-MGB<br>CT: 24.87	2205 A21 1000m saki3<br>U pGEM<br>2.25E4<br>FAM-NFQ-MGB<br>CT: 24.81	2205 A21 1000m saki3<br>U pGEM<br>2.4E4<br>FAM-NFQ-MGB<br>CT: 24.7		N pGEM<br>FAM-NFQ-MGB<br>CT: Undetermined
H	Comf2E4<br>U pGEM<br>2.26E4<br>FAM-NFQ-MGB<br>CT: 24.8	Comf2E4<br>U pGEM<br>2.71E4<br>FAM-NFQ-MGB<br>CT: 24.51		2205 A21 1000m ato2<br>U pGEM<br>3.05E4<br>FAM-NFQ-MGB<br>CT: 24.31	2205 A21 1000m ato2<br>U pGEM<br>2.84E4<br>FAM-NFQ-MGB<br>CT: 24.43	2205 A21 1000m ato2<br>U pGEM<br>2.88E4<br>FAM-NFQ-MGB<br>CT: 24.4		2205 A21 1000m ato3<br>U pGEM<br>2.95E4<br>FAM-NFQ-MGB<br>CT: 24.36	2205 A21 1000m ato3<br>U pGEM<br>2.96E4<br>FAM-NFQ-MGB<br>CT: 24.36	2205 A21 1000m ato3<br>U pGEM<br>3.04E4<br>FAM-NFQ-MGB<br>CT: 24.32		N pGEM<br>FAM-NFQ-MGB<br>CT: Undetermined

Standard Curves

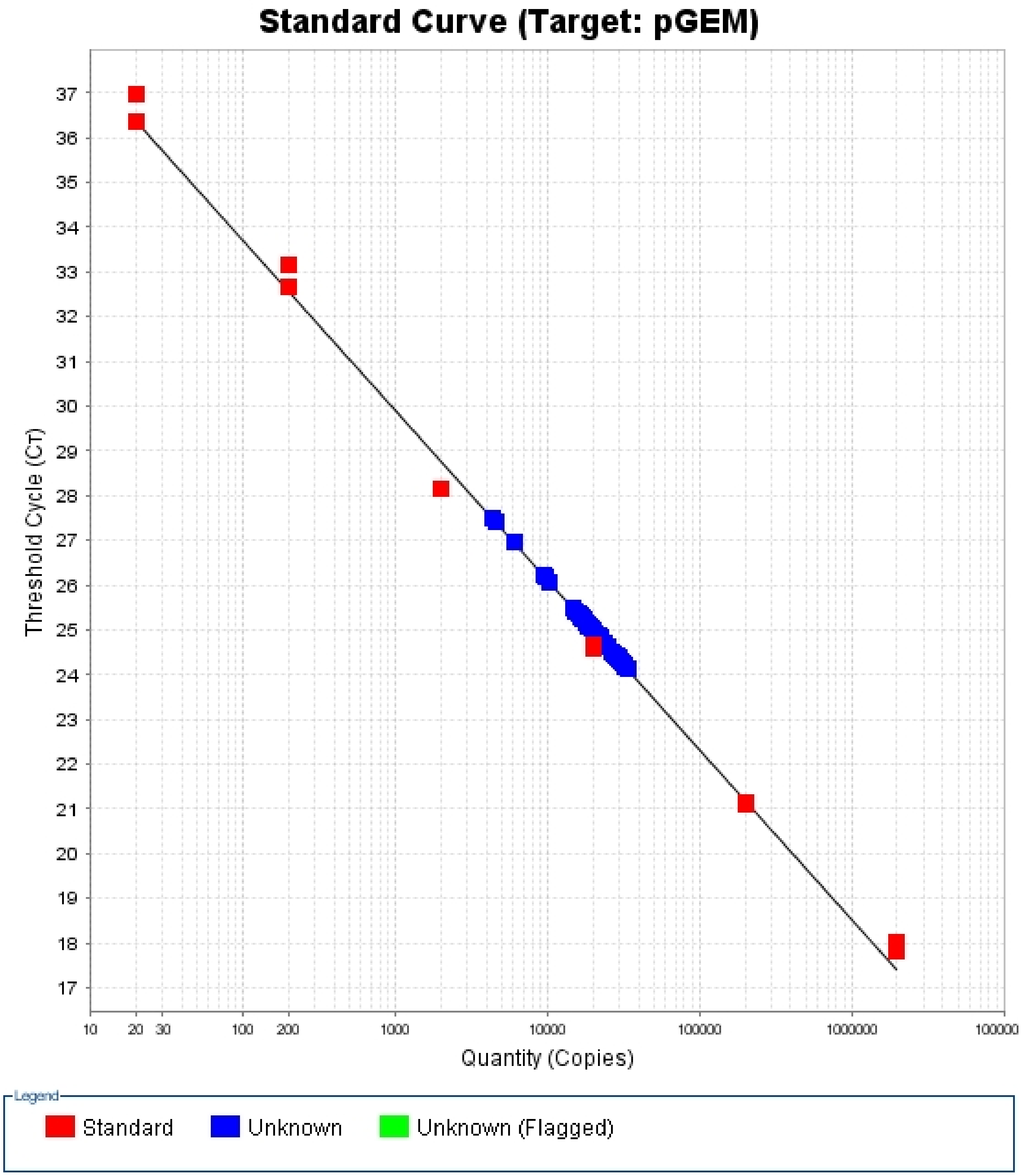

slope:-3.788 Y-Intercept:41.297 R^2:0.995 Eff%:83.653

Results Table

Well	Sample	Target	Task	Quantity	C
A1		pGEM	STANDARD	2000000	18.0189
A2		pGEM	STANDARD	2000000	17.826
A4	2205 A21 10m	pGEM	UNKNOWN	14928.9619	25.4859
A5	2205 A21 10m	pGEM	UNKNOWN	15531.4434	25.4208
A6	2205 A21 10m	pGEM	UNKNOWN	16134.8936	25.3581
A7	2205 A21	pGEM	UNKNOWN	4357.4844	27.5116
A8	2205 A21 10m	pGEM	UNKNOWN	16753.377	25.2962
A9	2205 A21 10m	pGEM	UNKNOWN	17303.3984	25.2431
A10	2205 A21 10m	pGEM	UNKNOWN	16501.3926	25.3212
A11	2205 A21	pGEM	UNKNOWN	19483.9727	25.0478
B1		pGEM	STANDARD	200000	21.1298
B2		pGEM	STANDARD	200000	21.1073
B4	2205 A21 10m	pGEM	UNKNOWN	31227.8887	24.2718
B5	2205 A21 10m	pGEM	UNKNOWN	30231.4297	24.3252
B6	2205 A21 10m	pGEM	UNKNOWN	32632.6523	24.1994
B7	2205 A21	pGEM	UNKNOWN	4668.438	27.3982
B8	2205 A21 10m	pGEM	UNKNOWN	30535.7617	24.3087
B9	2205 A21 10m	pGEM	UNKNOWN	30984.7344	24.2847
B10	2205 A21 10m	pGEM	UNKNOWN	31481.6016	24.2585
B11	2205 A21	pGEM	UNKNOWN	19794.9922	25.0218
C1		pGEM	STANDARD	20000	24.6784
C2		pGEM	STANDARD	20000	24.5773
C4	2205 A21 SCM	pGEM	UNKNOWN	9543.8623	26.2219
C5	2205 A21 SCM	pGEM	UNKNOWN	10405.498	26.0797
C6	2205 A21 SCM	pGEM	UNKNOWN	9827.1455	26.1738
C7	2205 A21	pGEM	UNKNOWN	6079.584	26.9638
C8	2205 A21 SCM	pGEM	UNKNOWN	20305.1621	24.9799
C9	2205 A21 SCM	pGEM	UNKNOWN	18968.6191	25.0919
C10	2205 A21 SCM	pGEM	UNKNOWN	21871.0977	24.8577
C11	2205 A21	pGEM	UNKNOWN	22127.0273	24.8386

Well		Sample	Target	Task	Quantity	C
D1			pGEM	STANDARD	2000	28.1692
D2			pGEM	STANDARD	2000	28.1338
D4		2205 A21 SCM	pGEM	UNKNOWN	28629.4219	24.4147
D5		2205 A21 SCM	pGEM	UNKNOWN	29136.416	24.3859
D6		2205 A21 SCM	pGEM	UNKNOWN	28864.9688	24.4013
D7		2205 A21	pGEM	UNKNOWN	28804.3887	24.4047
D8		2205 A21 SCM	pGEM	UNKNOWN	29740.1543	24.3521
D9		2205 A21 SCM	pGEM	UNKNOWN	32804.4219	24.1908
D10		2205 A21 SCM	pGEM	UNKNOWN	33874.0234	24.138
D11		2205 A21	pGEM	UNKNOWN	28254.7461	24.4364
E1			pGEM	STANDARD	200	33.1638
E2			pGEM	STANDARD	200	32.6678
E4		2205 A21 200m	pGEM	UNKNOWN	17447.6016	25.2294
E5		2205 A21 200m	pGEM	UNKNOWN	18243.5273	25.156
E6		2205 A21 200m	pGEM	UNKNOWN	16935.3359	25.2785
E7		2205 A21	pGEM	UNKNOWN	30204.3125	24.3266
E8		2205 A21 200m	pGEM	UNKNOWN	18685.0723	25.1167
E9		2205 A21 200m	pGEM	UNKNOWN	21519.873	24.8843
E10		2205 A21 200m	pGEM	UNKNOWN	20972.957	24.9267
E11		2205 A21	pGEM	UNKNOWN	28684.377	24.4116
F1			pGEM	STANDARD	20	36.9708
F2			pGEM	STANDARD	20	36.3395
F4		2205 A21 200m	pGEM	UNKNOWN	25263.207	24.6205
F5		2205 A21 200m	pGEM	UNKNOWN	27756.334	24.4657
F6		2205 A21 200m	pGEM	UNKNOWN	28352.3066	24.4307
F7		2205 A21	pGEM	UNKNOWN	27794.9141	24.4634
F8		2205 A21 200m	pGEM	UNKNOWN	31746.502	24.2447
F9		2205 A21 200m	pGEM	UNKNOWN	32473.1133	24.2075
F10		2205 A21 200m	pGEM	UNKNOWN	34309.0117	24.117
F11		2205 A21	pGEM	UNKNOWN	29759.2285	24.3511
G4		2205 A21	pGEM	UNKNOWN	18238.6641	25.1565
G5		2205 A21	pGEM	UNKNOWN	18182.5	25.1616

Well		Sample	Target	Task	Quantity	C
G6		2205 A21	pGEM	UNKNOWN	18900.7383	25.0978
G8		2205 A21	pGEM	UNKNOWN	21666.2812	24.8732
G9		2205 A21	pGEM	UNKNOWN	22467.1582	24.8135
G10		2205 A21	pGEM	UNKNOWN	24021.8945	24.7034
G12			pGEM	NTC		Undetermined
H1		Comf2E4	pGEM	UNKNOWN	22588.4043	24.8046
H2		Comf2E4	pGEM	UNKNOWN	27086.041	24.5059
H4		2205 A21	pGEM	UNKNOWN	30487.1191	24.3113
H5		2205 A21	pGEM	UNKNOWN	28414.7695	24.4271
H6		2205 A21	pGEM	UNKNOWN	28808.7988	24.4045
H8		2205 A21	pGEM	UNKNOWN	29512.9297	24.3647
H9		2205 A21	pGEM	UNKNOWN	29637.7129	24.3578
H10		2205 A21	pGEM	UNKNOWN	30372.3848	24.3175
H12			pGEM	NTC		Undetermined

QC Summary

Total Wells	96	Processed Wells	76	Targets Used	1
Well Setup	76	Flagged Wells	0	Samples Used	21

Flag	Name	Frequency	Locations
AMPNC	Amplification in negative control	0	
BADROX	Bad passive reference signal	0	
BLFAIL	Baseline algorithm failed	0	
CTFAIL	C algorithm failed	0	
EXPFAIL	Exponential algorithm failed	0	
HIGHSD	High standard deviation in replicate group	0	
NOAMP	No amplification	0	
NOISE	Noise higher than others in plate	0	
NOSIGNAL	No signal in well	0	
OFFSCALE	Fluorescence is offscale	0	
OUTLIERRG	Outlier in replicate group	0	
SPIKE	Noise spikes	0	
THOLDFAIL	Thresholding algorithm failed	0	
