# Supplementary material for: Ecological impact of algal-preying protists aplanochytrids (Labyrinthulea) as a source of docosahexaenoic acid for copepods in marine environments
Source: ISME Commun. 2026 Jun 26;6(1):ycag178. doi: 10.1093/ismeco/ycag178 (PMC13685785; doi:10.1093/ismeco/ycag178)
Supplement: Supplementary file 2 [file supplementary_materials_ycag178.zip › Morimoto_ISME_C_3rdrev_supplemental_data.zip!/Morimoto_ISME_C_3rdrev_supplemental_data/Supplemental_Data_2/Standard Curve/DNA recovery/2205/DnaRecAline2205_N=3_Amari.pdf]

Experiment Results Report

230322DnaRecAline2205DnaExt=3Amari

Experiment Summary

Experiment Name	: 230322DnaRecAline2205DnaExt=3Amari
Experiment Type	: Quantitation - Standard Curve
File Name	: 230322DnaRecAline2205DnaExt=3Amari.eds
Run Started	: 2023 Mar 22 5:05:09 PM
Run Finished	: 2023 Mar 22 6:39:45 PM
Run Duration	: 94 minutes 36 seconds
Date Modified	: 2023 Mar 24 1:46:02 PM
User	:
Number of wells used	: 28
Number of wells with results	: 28
Instrument Name	:
Instrument Type	: Applied Biosystems StepOnePlus™ Instrument
Comments	:

Results Summary

Sample	Target	Quantity (Mean)	Quantity (Std Dev)	C (Mean)	C (Std Dev)
2205 A01 SCM ato3	pGEM	21733.90	465.51	23.15	0.03
2205 A01 SCM saki3	pGEM	15207.00	375.84	23.70	0.04
2205 A04 3000m	pGEM	5673.16	701.10	25.21	0.18
2205 A04 3000m	pGEM	16540.04	37.34	23.57	0.00
Comf2E4	pGEM	20980.96	992.42	23.21	0.07

Plate Layout

	1	2	3	4	5	6	7	8	9	10	11	12
A	S pGEM<br>2E6<br>FAM-NFQ-MGB<br>CT: 16.57	S pGEM<br>2E6<br>FAM-NFQ-MGB<br>CT: 16.42		2205 A01 SCM saki3<br>U pGEM<br>1.56E4<br>FAM-NFQ-MGB<br>CT: 23.66	2205 A01 SCM saki3<br>U pGEM<br>1.5E4<br>FAM-NFQ-MGB<br>CT: 23.72	2205 A01 SCM saki3<br>U pGEM<br>1.5E4<br>FAM-NFQ-MGB<br>CT: 23.72						
B	S pGEM<br>2E5<br>FAM-NFQ-MGB<br>CT: 19.82	S pGEM<br>2E5<br>FAM-NFQ-MGB<br>CT: 19.49		2205 A01 SCM ato3<br>U pGEM<br>2.15E4<br>FAM-NFQ-MGB<br>CT: 23.17	2205 A01 SCM ato3<br>U pGEM<br>2.23E4<br>FAM-NFQ-MGB<br>CT: 23.12	2205 A01 SCM ato3<br>U pGEM<br>2.15E4<br>FAM-NFQ-MGB<br>CT: 23.17						
C	S pGEM<br>2E4<br>FAM-NFQ-MGB<br>CT: 23.1	S pGEM<br>2E4<br>FAM-NFQ-MGB<br>CT: 23.07		2205 A04 3000m saki3<br>U pGEM<br>1.65E4<br>FAM-NFQ-MGB<br>CT: 23.57	2205 A04 3000m saki3<br>U pGEM<br>1.66E4<br>FAM-NFQ-MGB<br>CT: 23.57	2205 A04 3000m saki3<br>U pGEM<br>1.65E4<br>FAM-NFQ-MGB<br>CT: 23.57						
D	S pGEM<br>2E3<br>FAM-NFQ-MGB<br>CT: 26.75	S pGEM<br>2E3<br>FAM-NFQ-MGB<br>CT: 26.62		2205 A04 3000m ato3<br>U pGEM<br>6.47E3<br>FAM-NFQ-MGB<br>CT: 25	2205 A04 3000m ato3<br>U pGEM<br>5.14E3<br>FAM-NFQ-MGB<br>CT: 25.35	2205 A04 3000m ato3<br>U pGEM<br>5.41E3<br>FAM-NFQ-MGB<br>CT: 25.28						
E	S pGEM<br>200<br>FAM-NFQ-MGB<br>CT: 30.16	S pGEM<br>200<br>FAM-NFQ-MGB<br>CT: 30.51										
F	S pGEM<br>20<br>FAM-NFQ-MGB<br>CT: 34.17	S pGEM<br>20<br>FAM-NFQ-MGB<br>CT: 33.77										
G												
H	Comf2E4<br>U pGEM<br>2.03E4<br>FAM-NFQ-MGB<br>CT: 23.26	Comf2E4<br>U pGEM<br>2.17E4<br>FAM-NFQ-MGB<br>CT: 23.16									N pGEM<br>FAM-NFQ-MGB<br>CT: Undetermined	N pGEM<br>FAM-NFQ-MGB<br>CT: 36.98

Standard Curves

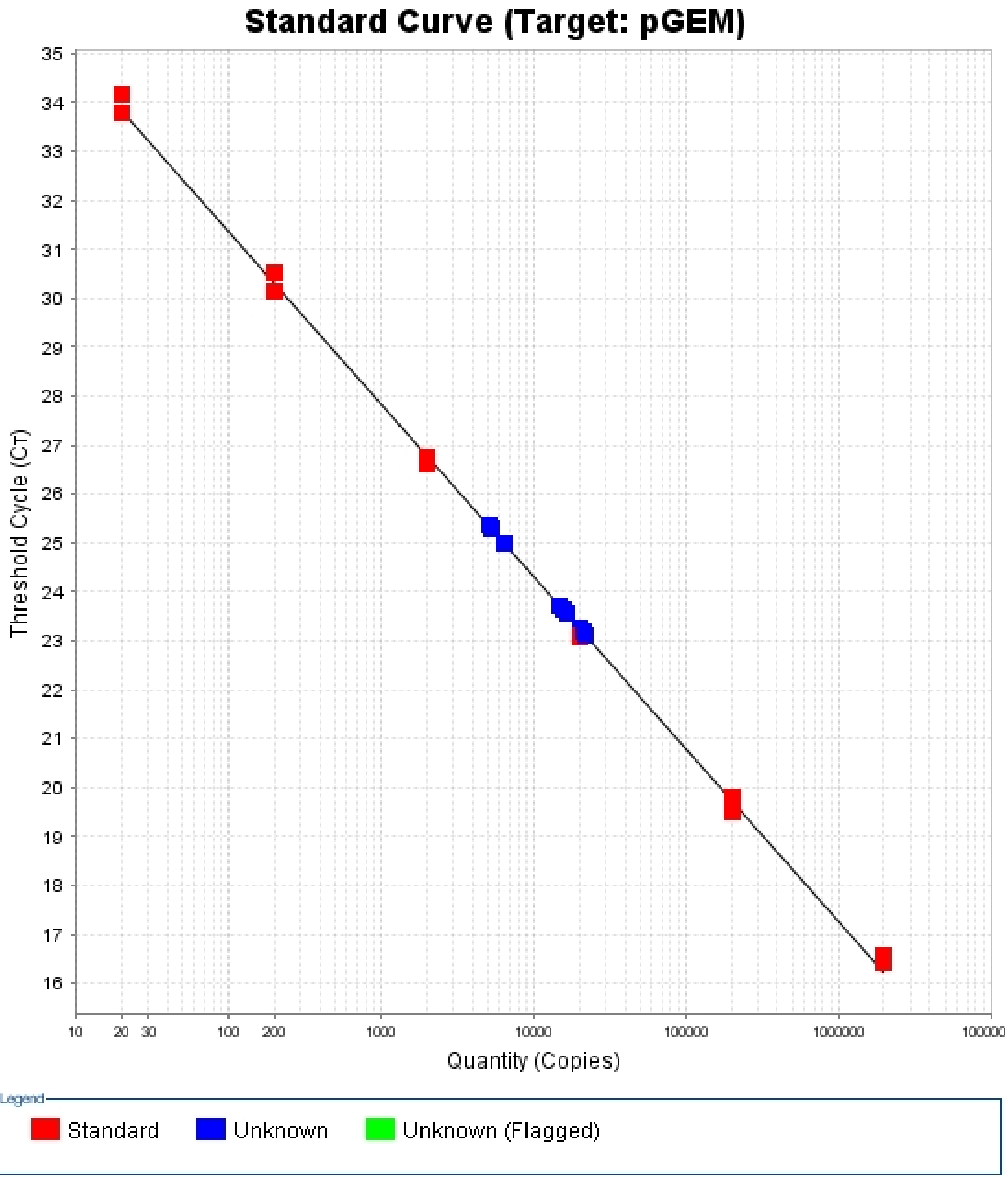

slope:-3.515 Y-Intercept:38.397 R^2:0.999 Eff%:92.536

Results Table

Well	Sample	Target	Task	Quantity	C
A1		pGEM	STANDARD	2000000	16.5705
A2		pGEM	STANDARD	2000000	16.4194
A4	2205 A01 SCM	pGEM	UNKNOWN	15639.6406	23.6554
A5	2205 A01 SCM	pGEM	UNKNOWN	15020.2471	23.7171
A6	2205 A01 SCM	pGEM	UNKNOWN	14961.1123	23.7231
B1		pGEM	STANDARD	200000	19.8169
B2		pGEM	STANDARD	200000	19.487
B4	2205 A01 SCM	pGEM	UNKNOWN	21453.627	23.1729
B5	2205 A01 SCM	pGEM	UNKNOWN	22271.2598	23.1158
B6	2205 A01 SCM	pGEM	UNKNOWN	21476.8281	23.1713
C1		pGEM	STANDARD	20000	23.1047
C2		pGEM	STANDARD	20000	23.068
C4	2205 A04	pGEM	UNKNOWN	16532.1406	23.5707
C5	2205 A04	pGEM	UNKNOWN	16580.6953	23.5662
C6	2205 A04	pGEM	UNKNOWN	16507.2891	23.573
D1		pGEM	STANDARD	2000	26.7508
D2		pGEM	STANDARD	2000	26.6226
D4	2205 A04	pGEM	UNKNOWN	6468.4175	25.0031
D5	2205 A04	pGEM	UNKNOWN	5144.3018	25.3527
D6	2205 A04	pGEM	UNKNOWN	5406.7642	25.2768
E1		pGEM	STANDARD	200	30.1585
E2		pGEM	STANDARD	200	30.5064
F1		pGEM	STANDARD	20	34.1686
F2		pGEM	STANDARD	20	33.773
H1	Comf2E4	pGEM	UNKNOWN	20279.2129	23.2589
H2	Comf2E4	pGEM	UNKNOWN	21682.7012	23.1567
H11		pGEM	NTC		Undetermined
H12		pGEM	NTC		36.9831

QC Summary

Total Wells	96	Processed Wells	28	Targets Used	1
Well Setup	28	Flagged Wells	0	Samples Used	5

Flag	Name	Frequency	Locations
AMPNC	Amplification in negative control	0	
BADROX	Bad passive reference signal	0	
BLFAIL	Baseline algorithm failed	0	
CTFAIL	C algorithm failed	0	
EXPFAIL	Exponential algorithm failed	0	
HIGHSD	High standard deviation in replicate group	0	
NOAMP	No amplification	0	
NOISE	Noise higher than others in plate	0	
NOSIGNAL	No signal in well	0	
OFFSCALE	Fluorescence is offscale	0	
OUTLIERRG	Outlier in replicate group	0	
SPIKE	Noise spikes	0	
THOLDFAIL	Thresholding algorithm failed	0	
