# Supplementary material for: Ecological impact of algal-preying protists aplanochytrids (Labyrinthulea) as a source of docosahexaenoic acid for copepods in marine environments
Source: ISME Commun. 2026 Jun 26;6(1):ycag178. doi: 10.1093/ismeco/ycag178 (PMC13685785; doi:10.1093/ismeco/ycag178)
Supplement: Supplementary file 2 [file supplementary_materials_ycag178.zip › Morimoto_ISME_C_3rdrev_supplemental_data.zip!/Morimoto_ISME_C_3rdrev_supplemental_data/Supplemental_Data_2/Standard Curve/DNA recovery/2205/DnaRecAline2205A04_N=3.pdf]

Experiment Results Report

230317DnaRecAline2205A04DnaExtN=3EmmByKurabo

Experiment Summary

Experiment Name	: 230317DnaRecAline2205A04DnaExtN=3EmmByKurabo
Experiment Type	: Quantitation - Standard Curve
File Name	: 230317DnaRecAline2205A04DnaExtN=3EmmByKurabo.eds
Run Started	: 2023 Mar 17 3:27:44 PM
Run Finished	: 2023 Mar 17 5:02:27 PM
Run Duration	: 94 minutes 42 seconds
Date Modified	: 2023 Mar 20 11:36:21 AM
User	:
Number of wells used	: 70
Number of wells with results	: 70
Instrument Name	:
Instrument Type	: Applied Biosystems StepOnePlus™ Instrument
Comments	:

Results Summary

Sample	Target	Quantity (Mean)	Quantity (Std Dev)	C (Mean)	C (Std Dev)
2205 A04 1000m	pGEM	29329.18	2048.96	24.68	0.11
2205 A04 1000m	pGEM	25720.77	703.47	24.88	0.04
2205 A04 1000m	pGEM	14254.43	1874.87	25.81	0.21
2205 A04 1000m	pGEM	15018.58	481.23	25.72	0.05
2205 A04 10m ato2	pGEM	25053.72	729.38	24.92	0.04
2205 A04 10m ato3	pGEM	26894.34	2008.31	24.81	0.12
2205 A04 10m saki2	pGEM	11696.59	378.66	26.11	0.05
2205 A04 10m saki3	pGEM	11556.40	315.17	26.13	0.04
2205 A04 200m ato2	pGEM	25163.75	1647.14	24.92	0.10
2205 A04 200m ato3	pGEM	25699.82	456.95	24.88	0.03
2205 A04 200m	pGEM	15204.03	540.02	25.70	0.05
2205 A04 200m	pGEM	16364.64	1334.55	25.59	0.13
2205 A04 3000m	pGEM	26369.35	2519.45	24.85	0.15
2205 A04 3000m	pGEM	13947.31	479.23	25.83	0.05
2205 A04 SCM ato2	pGEM	26109.56	1165.33	24.86	0.07
2205 A04 SCM ato3	pGEM	26286.57	1036.27	24.85	0.06
2205 A04 SCM saki2	pGEM	14244.91	665.91	25.80	0.07
2205 A04 SCM saki3	pGEM	13142.05	898.59	25.93	0.11
Comf2E4	pGEM	22014.23	3003.39	25.13	0.21

Plate Layout

	1	2	3	4	5	6	7	8	9	10	11	12
A	S pGEM<br>2E6<br>FAM-NFQ-MGB<br>CT: 18.45	S pGEM<br>2E6<br>FAM-NFQ-MGB<br>CT: 18.34		2205 A04 10m saki2<br>U pGEM<br>1.17E4<br>FAM-NFQ-MGB<br>CT: 26.11	2205 A04 10m saki2<br>U pGEM<br>1.13E4<br>FAM-NFQ-MGB<br>CT: 26.16	2205 A04 10m saki2<br>U pGEM<br>1.21E4<br>FAM-NFQ-MGB<br>CT: 26.06	2205 A04 3000m saki2<br>U pGEM<br>1.34E4<br>FAM-NFQ-MGB<br>CT: 25.89	2205 A04 10m saki3<br>U pGEM<br>1.16E4<br>FAM-NFQ-MGB<br>CT: 26.11	2205 A04 10m saki3<br>U pGEM<br>1.18E4<br>FAM-NFQ-MGB<br>CT: 26.09	2205 A04 10m saki3<br>U pGEM<br>1.12E4<br>FAM-NFQ-MGB<br>CT: 26.17		
B	S pGEM<br>2E5<br>FAM-NFQ-MGB<br>CT: 21.61	S pGEM<br>2E5<br>FAM-NFQ-MGB<br>CT: 21.59		2205 A04 10m ato2<br>U pGEM<br>2.59E4<br>FAM-NFQ-MGB<br>CT: 24.87	2205 A04 10m ato2<br>U pGEM<br>2.47E4<br>FAM-NFQ-MGB<br>CT: 24.95	2205 A04 10m ato2<br>U pGEM<br>2.46E4<br>FAM-NFQ-MGB<br>CT: 24.95	2205 A04 3000m saki2<br>U pGEM<br>1.4E4<br>FAM-NFQ-MGB<br>CT: 25.82	2205 A04 10m ato3<br>U pGEM<br>2.51E4<br>FAM-NFQ-MGB<br>CT: 24.92	2205 A04 10m ato3<br>U pGEM<br>2.66E4<br>FAM-NFQ-MGB<br>CT: 24.83	2205 A04 10m ato3<br>U pGEM<br>2.91E4<br>FAM-NFQ-MGB<br>CT: 24.69		
C	S pGEM<br>2E4<br>FAM-NFQ-MGB<br>CT: 25.17	S pGEM<br>2E4<br>FAM-NFQ-MGB<br>CT: 25.11		2205 A04 SCM saki2<br>U pGEM<br>1.44E4<br>FAM-NFQ-MGB<br>CT: 25.78	2205 A04 SCM saki2<br>U pGEM<br>1.35E4<br>FAM-NFQ-MGB<br>CT: 25.88	2205 A04 SCM saki2<br>U pGEM<br>1.48E4<br>FAM-NFQ-MGB<br>CT: 25.74	2205 A04 3000m saki2<br>U pGEM<br>1.44E4<br>FAM-NFQ-MGB<br>CT: 25.78	2205 A04 SCM saki3<br>U pGEM<br>1.3E4<br>FAM-NFQ-MGB<br>CT: 25.94	2205 A04 SCM saki3<br>U pGEM<br>1.23E4<br>FAM-NFQ-MGB<br>CT: 26.03	2205 A04 SCM saki3<br>U pGEM<br>1.41E4<br>FAM-NFQ-MGB<br>CT: 25.82		
D	S pGEM<br>2E3<br>FAM-NFQ-MGB<br>CT: 28.54	S pGEM<br>2E3<br>FAM-NFQ-MGB<br>CT: 28.65		2205 A04 SCM ato2<br>U pGEM<br>2.7E4<br>FAM-NFQ-MGB<br>CT: 24.8	2205 A04 SCM ato2<br>U pGEM<br>2.48E4<br>FAM-NFQ-MGB<br>CT: 24.94	2205 A04 SCM ato2<br>U pGEM<br>2.65E4<br>FAM-NFQ-MGB<br>CT: 24.83	2205 A04 3000m ato2<br>U pGEM<br>2.92E4<br>FAM-NFQ-MGB<br>CT: 24.68	2205 A04 SCM ato3<br>U pGEM<br>2.51E4<br>FAM-NFQ-MGB<br>CT: 24.92	2205 A04 SCM ato3<br>U pGEM<br>2.68E4<br>FAM-NFQ-MGB<br>CT: 24.82	2205 A04 SCM ato3<br>U pGEM<br>2.7E4<br>FAM-NFQ-MGB<br>CT: 24.81		
E	S pGEM<br>200<br>FAM-NFQ-MGB<br>CT: 32.28	S pGEM<br>200<br>FAM-NFQ-MGB<br>CT: 32.24		2205 A04 200m saki2<br>U pGEM<br>1.58E4<br>FAM-NFQ-MGB<br>CT: 25.64	2205 A04 200m saki2<br>U pGEM<br>1.49E4<br>FAM-NFQ-MGB<br>CT: 25.73	2205 A04 200m saki2<br>U pGEM<br>1.48E4<br>FAM-NFQ-MGB<br>CT: 25.74	2205 A04 3000m ato2<br>U pGEM<br>2.54E4<br>FAM-NFQ-MGB<br>CT: 24.9	2205 A04 200m saki3<br>U pGEM<br>1.48E4<br>FAM-NFQ-MGB<br>CT: 25.74	2205 A04 200m saki3<br>U pGEM<br>1.73E4<br>FAM-NFQ-MGB<br>CT: 25.5	2205 A04 200m saki3<br>U pGEM<br>1.7E4<br>FAM-NFQ-MGB<br>CT: 25.53		
F	⚠<br>S pGEM<br>20<br>FAM-NFQ-MGB<br>CT: 35.96	⚠<br>S pGEM<br>20<br>FAM-NFQ-MGB<br>CT: 36.81		2205 A04 200m ato2<br>U pGEM<br>2.33E4<br>FAM-NFQ-MGB<br>CT: 25.04	2205 A04 200m ato2<br>U pGEM<br>2.62E4<br>FAM-NFQ-MGB<br>CT: 24.85	2205 A04 200m ato2<br>U pGEM<br>2.6E4<br>FAM-NFQ-MGB<br>CT: 24.86	2205 A04 3000m ato2<br>U pGEM<br>2.44E4<br>FAM-NFQ-MGB<br>CT: 24.96	2205 A04 200m ato3<br>U pGEM<br>2.52E4<br>FAM-NFQ-MGB<br>CT: 24.91	2205 A04 200m ato3<br>U pGEM<br>2.57E4<br>FAM-NFQ-MGB<br>CT: 24.88	2205 A04 200m ato3<br>U pGEM<br>2.62E4<br>FAM-NFQ-MGB<br>CT: 24.85		
G				2205 A04 1000m saki2<br>U pGEM<br>1.53E4<br>FAM-NFQ-MGB<br>CT: 25.69	2205 A04 1000m saki2<br>U pGEM<br>1.54E4<br>FAM-NFQ-MGB<br>CT: 25.68	2205 A04 1000m saki2<br>U pGEM<br>1.21E4<br>FAM-NFQ-MGB<br>CT: 26.06		2205 A04 1000m saki3<br>U pGEM<br>1.45E4<br>FAM-NFQ-MGB<br>CT: 25.78	2205 A04 1000m saki3<br>U pGEM<br>1.52E4<br>FAM-NFQ-MGB<br>CT: 25.69	2205 A04 1000m saki3<br>U pGEM<br>1.53E4<br>FAM-NFQ-MGB<br>CT: 25.68		N pGEM<br>FAM-NFQ-MGB<br>CT: Undetermined
H	Comf2E4<br>U pGEM<br>1.99E4<br>FAM-NFQ-MGB<br>CT: 25.28	Comf2E4<br>U pGEM<br>2.41E4<br>FAM-NFQ-MGB<br>CT: 24.98		2205 A04 1000m ato2<br>U pGEM<br>2.97E4<br>FAM-NFQ-MGB<br>CT: 24.66	2205 A04 1000m ato2<br>U pGEM<br>2.71E4<br>FAM-NFQ-MGB<br>CT: 24.8	2205 A04 1000m ato2<br>U pGEM<br>3.12E4<br>FAM-NFQ-MGB<br>CT: 24.58		2205 A04 1000m ato3<br>U pGEM<br>2.56E4<br>FAM-NFQ-MGB<br>CT: 24.89	2205 A04 1000m ato3<br>U pGEM<br>2.65E4<br>FAM-NFQ-MGB<br>CT: 24.84	2205 A04 1000m ato3<br>U pGEM<br>2.51E4<br>FAM-NFQ-MGB<br>CT: 24.92		N pGEM<br>FAM-NFQ-MGB<br>CT: Undetermined

Standard Curves

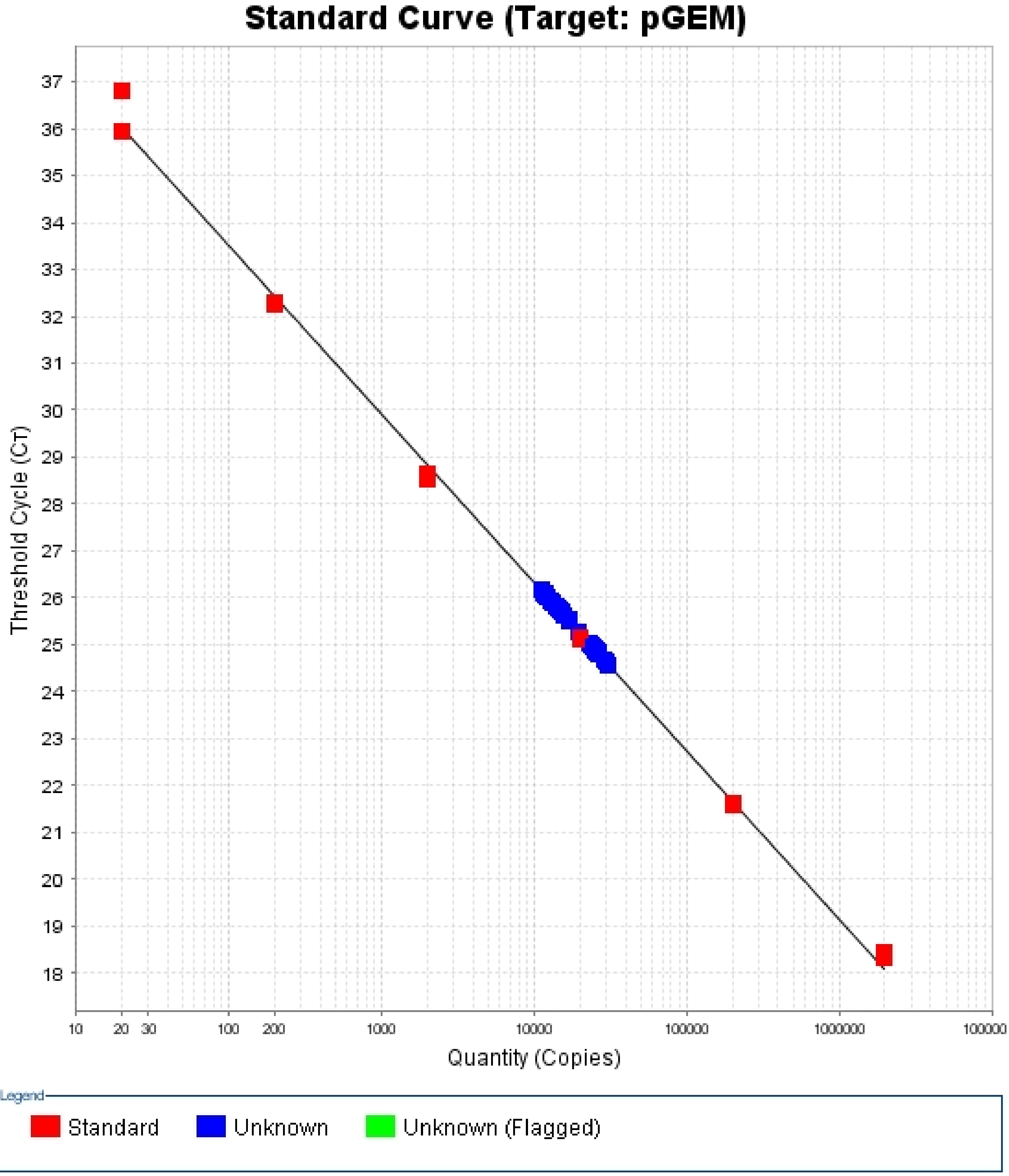

slope:-3.582 Y-Intercept:40.68 R^2:0.998 Eff%:90.167

Results Table

Well		Sample	Target	Task	Quantity	C
A1			pGEM	STANDARD	2000000	18.4531
A2			pGEM	STANDARD	2000000	18.3396
A4		2205 A04 10m	pGEM	UNKNOWN	11680.5469	26.1087
A5		2205 A04 10m	pGEM	UNKNOWN	11326.2021	26.1566
A6		2205 A04 10m	pGEM	UNKNOWN	12083.0117	26.056
A7		2205 A04	pGEM	UNKNOWN	13435.6191	25.8909
A8		2205 A04 10m	pGEM	UNKNOWN	11647.916	26.1131
A9		2205 A04 10m	pGEM	UNKNOWN	11815.6807	26.0908
A10		2205 A04 10m	pGEM	UNKNOWN	11205.6055	26.1733
B1			pGEM	STANDARD	200000	21.6072
B2			pGEM	STANDARD	200000	21.5919
B4		2205 A04 10m	pGEM	UNKNOWN	25895.3457	24.87
B5		2205 A04 10m	pGEM	UNKNOWN	24660.1484	24.9461
B6		2205 A04 10m	pGEM	UNKNOWN	24605.6719	24.9495
B7		2205 A04	pGEM	UNKNOWN	14020.6758	25.8246
B8		2205 A04 10m	pGEM	UNKNOWN	25079.627	24.9198
B9		2205 A04 10m	pGEM	UNKNOWN	26551.3457	24.8311
B10		2205 A04 10m	pGEM	UNKNOWN	29052.0605	24.6911
C1			pGEM	STANDARD	20000	25.1681
C2			pGEM	STANDARD	20000	25.1075
C4		2205 A04 SCM	pGEM	UNKNOWN	14434.1162	25.7794
C5		2205 A04 SCM	pGEM	UNKNOWN	13504.876	25.8829
C6		2205 A04 SCM	pGEM	UNKNOWN	14795.7451	25.7409
C7		2205 A04	pGEM	UNKNOWN	14385.625	25.7846
C8		2205 A04 SCM	pGEM	UNKNOWN	12998.0254	25.9424
C9		2205 A04 SCM	pGEM	UNKNOWN	12324.1826	26.0253
C10		2205 A04 SCM	pGEM	UNKNOWN	14103.958	25.8154
D1			pGEM	STANDARD	2000	28.5429
D2			pGEM	STANDARD	2000	28.654
D4		2205 A04 SCM	pGEM	UNKNOWN	27030.7148	24.8033

Well		Sample	Target	Task	Quantity	C
D5		2205 A04 SCM	pGEM	UNKNOWN	24799.5137	24.9373
D6		2205 A04 SCM	pGEM	UNKNOWN	26498.4395	24.8342
D7		2205 A04	pGEM	UNKNOWN	29220.1504	24.6821
D8		2205 A04 SCM	pGEM	UNKNOWN	25093.5898	24.919
D9		2205 A04 SCM	pGEM	UNKNOWN	26802.8359	24.8164
D10		2205 A04 SCM	pGEM	UNKNOWN	26963.2988	24.8072
E1			pGEM	STANDARD	200	32.2791
E2			pGEM	STANDARD	200	32.2425
E4		2205 A04 200m	pGEM	UNKNOWN	15824.9775	25.6363
E5		2205 A04 200m	pGEM	UNKNOWN	14942.8838	25.7255
E6		2205 A04 200m	pGEM	UNKNOWN	14844.2168	25.7358
E7		2205 A04	pGEM	UNKNOWN	25446.2773	24.8973
E8		2205 A04 200m	pGEM	UNKNOWN	14836.249	25.7366
E9		2205 A04 200m	pGEM	UNKNOWN	17299.207	25.4977
E10		2205 A04 200m	pGEM	UNKNOWN	16958.4766	25.5286
F1			pGEM	STANDARD	20	35.9609
F2			pGEM	STANDARD	20	36.8097
F4		2205 A04 200m	pGEM	UNKNOWN	23265.291	25.0367
F5		2205 A04 200m	pGEM	UNKNOWN	26212.8242	24.8511
F6		2205 A04 200m	pGEM	UNKNOWN	26013.1328	24.863
F7		2205 A04	pGEM	UNKNOWN	24441.6133	24.9599
F8		2205 A04 200m	pGEM	UNKNOWN	25236.457	24.9101
F9		2205 A04 200m	pGEM	UNKNOWN	25712.9141	24.881
F10		2205 A04 200m	pGEM	UNKNOWN	26150.0762	24.8548
G4		2205 A04	pGEM	UNKNOWN	15310.9697	25.6876
G5		2205 A04	pGEM	UNKNOWN	15362.5996	25.6824
G6		2205 A04	pGEM	UNKNOWN	12089.7236	26.0551
G8		2205 A04	pGEM	UNKNOWN	14466.1445	25.7759
G9		2205 A04	pGEM	UNKNOWN	15242.8369	25.6946
G10		2205 A04	pGEM	UNKNOWN	15346.75	25.684
G12			pGEM	NTC		Undetermined
H1		Comf2E4	pGEM	UNKNOWN	19890.5176	25.2805

Well		Sample	Target	Task	Quantity	C
H2		Comf2E4	pGEM	UNKNOWN	24137.9512	24.9794
H4		2205 A04	pGEM	UNKNOWN	29725.3574	24.6554
H5		2205 A04	pGEM	UNKNOWN	27111.0605	24.7987
H6		2205 A04	pGEM	UNKNOWN	31151.1133	24.5825
H8		2205 A04	pGEM	UNKNOWN	25620.25	24.8867
H9		2205 A04	pGEM	UNKNOWN	26469.0898	24.8359
H10		2205 A04	pGEM	UNKNOWN	25072.957	24.9202
H12			pGEM	NTC		Undetermined

QC Summary

Total Wells	96	Processed Wells	70	Targets Used	1
Well Setup	70	Flagged Wells	2	Samples Used	19

Flag	Name	Frequency	Locations
AMPNC	Amplification in negative control	0	
BADROX	Bad passive reference signal	0	
BLFAIL	Baseline algorithm failed	0	
CTFAIL	C algorithm failed	0	
EXPFAIL	Exponential algorithm failed	0	
HIGHSD	High standard deviation in replicate group	2	F1, F2
NOAMP	No amplification	0	
NOISE	Noise higher than others in plate	0	
NOSIGNAL	No signal in well	0	
OFFSCALE	Fluorescence is offscale	0	
OUTLIERRG	Outlier in replicate group	0	
SPIKE	Noise spikes	0	
THOLDFAIL	Thresholding algorithm failed	0	
