# Supplementary material for: Ecological impact of algal-preying protists aplanochytrids (Labyrinthulea) as a source of docosahexaenoic acid for copepods in marine environments
Source: ISME Commun. 2026 Jun 26;6(1):ycag178. doi: 10.1093/ismeco/ycag178 (PMC13685785; doi:10.1093/ismeco/ycag178)
Supplement: Supplementary file 2 [file supplementary_materials_ycag178.zip › Morimoto_ISME_C_3rdrev_supplemental_data.zip!/Morimoto_ISME_C_3rdrev_supplemental_data/Supplemental_Data_2/Standard Curve/DNA recovery/2203/DnaRec2203AlineA21N=3.pdf]

Experiment Results Report

230621DnaRec2203AlineA21N=3

Experiment Summary

Experiment Name	: 230621DnaRec2203AlineA21N=3
Experiment Type	: Quantitation - Standard Curve
File Name	: 230621DnaRec2203AlineA21N=3.eds
Run Started	: 2023 Jun 21 5:50:02 PM
Run Finished	: 2023 Jun 21 7:24:40 PM
Run Duration	: 94 minutes 37 seconds
Date Modified	: 2023 Jun 22 1:21:12 PM
User	:
Number of wells used	: 40
Number of wells with results	: 40
Instrument Name	:
Instrument Type	: Applied Biosystems StepOnePlus™ Instrument
Comments	:

Results Summary

Sample	Target	Quantity (Mean)	Quantity (Std Dev)	C (Mean)	C (Std Dev)
2203 A21 1000 ato3	pGEM	22855.31	1425.07	25.83	0.09
2203 A21 1000 saki3	pGEM	11430.04	747.05	26.85	0.10
2203 A21 200 ato3	pGEM	22953.94	977.99	25.83	0.06
2203 A21 200 saki3	pGEM	15561.41	799.64	26.40	0.07
2203 A21 3000 ato3	pGEM	22671.78	1857.14	25.85	0.12
2203 A21 3000 saki3	pGEM	6875.39	288.68	27.60	0.06
2203 A21 SCM ato3	pGEM	22774.14	1395.72	25.84	0.09
2203 A21 SCM saki3	pGEM	8099.55	2302.44	27.40	0.46
Comf2E4	pGEM	29201.96	3381.64	25.48	0.17

Plate Layout

	1	2	3	4	5	6	7	8	9	10	11	12
A	S pGEM<br>2E6<br>FAM-NFQ-MGB<br>CT: 19.52	S pGEM<br>2E6<br>FAM-NFQ-MGB<br>CT: 19.38	2203 A21 SCM saki3<br>U pGEM<br>9.18E3<br>FAM-NFQ-MGB<br>CT: 27.17	2203 A21 SCM saki3<br>U pGEM<br>5.46E3<br>FAM-NFQ-MGB<br>CT: 27.93	2203 A21 SCM saki3<br>U pGEM<br>9.66E3<br>FAM-NFQ-MGB<br>CT: 27.09							
B	S pGEM<br>2E5<br>FAM-NFQ-MGB<br>CT: 23.25	S pGEM<br>2E5<br>FAM-NFQ-MGB<br>CT: 22.61	2203 A21 SCM ato3<br>U pGEM<br>2.31E4<br>FAM-NFQ-MGB<br>CT: 25.81	2203 A21 SCM ato3<br>U pGEM<br>2.4E4<br>FAM-NFQ-MGB<br>CT: 25.76	2203 A21 SCM ato3<br>U pGEM<br>2.12E4<br>FAM-NFQ-MGB<br>CT: 25.94							
C	S pGEM<br>2E4<br>FAM-NFQ-MGB<br>CT: 25.49	S pGEM<br>2E4<br>FAM-NFQ-MGB<br>CT: 25.34	2203 A21 200 saki3<br>U pGEM<br>1.49E4<br>FAM-NFQ-MGB<br>CT: 26.46	2203 A21 200 saki3<br>U pGEM<br>1.65E4<br>FAM-NFQ-MGB<br>CT: 26.31	2203 A21 200 saki3<br>U pGEM<br>1.53E4<br>FAM-NFQ-MGB<br>CT: 26.42							
D	S pGEM<br>2E3<br>FAM-NFQ-MGB<br>CT: 29.34	S pGEM<br>2E3<br>FAM-NFQ-MGB<br>CT: 29.31	2203 A21 200 ato3<br>U pGEM<br>2.35E4<br>FAM-NFQ-MGB<br>CT: 25.79	2203 A21 200 ato3<br>U pGEM<br>2.35E4<br>FAM-NFQ-MGB<br>CT: 25.79	2203 A21 200 ato3<br>U pGEM<br>2.18E4<br>FAM-NFQ-MGB<br>CT: 25.9							
E	S pGEM<br>200<br>FAM-NFQ-MGB<br>CT: 32.65	S pGEM<br>200<br>FAM-NFQ-MGB<br>CT: 32.92	2203 A21 1000 saki3<br>U pGEM<br>1.2E4<br>FAM-NFQ-MGB<br>CT: 26.78	2203 A21 1000 saki3<br>U pGEM<br>1.17E4<br>FAM-NFQ-MGB<br>CT: 26.81	2203 A21 1000 saki3<br>U pGEM<br>1.06E4<br>FAM-NFQ-MGB<br>CT: 26.96							
F	1<br>S pGEM<br>20<br>FAM-NFQ-MGB<br>CT: 35.95	1<br>S pGEM<br>20<br>FAM-NFQ-MGB<br>CT: 36.87	2203 A21 1000 ato3<br>U pGEM<br>2.26E4<br>FAM-NFQ-MGB<br>CT: 25.85	2203 A21 1000 ato3<br>U pGEM<br>2.16E4<br>FAM-NFQ-MGB<br>CT: 25.92	2203 A21 1000 ato3<br>U pGEM<br>2.44E4<br>FAM-NFQ-MGB<br>CT: 25.74							
G			2203 A21 3000 saki3<br>U pGEM<br>7.02E3<br>FAM-NFQ-MGB<br>CT: 27.56	2203 A21 3000 saki3<br>U pGEM<br>7.06E3<br>FAM-NFQ-MGB<br>CT: 27.56	2203 A21 3000 saki3<br>U pGEM<br>6.54E3<br>FAM-NFQ-MGB<br>CT: 27.67							N pGEM<br>FAM-NFQ-MGB<br>CT: Undetermined
H	Comf2E4<br>U pGEM<br>2.68E4<br>FAM-NFQ-MGB<br>CT: 25.6	Comf2E4<br>U pGEM<br>3.16E4<br>FAM-NFQ-MGB<br>CT: 25.36	2203 A21 3000 ato3<br>U pGEM<br>2.44E4<br>FAM-NFQ-MGB<br>CT: 25.74	2203 A21 3000 ato3<br>U pGEM<br>2.3E4<br>FAM-NFQ-MGB<br>CT: 25.82	2203 A21 3000 ato3<br>U pGEM<br>2.07E4<br>FAM-NFQ-MGB<br>CT: 25.98							N pGEM<br>FAM-NFQ-MGB<br>CT: Undetermined

Standard Curves

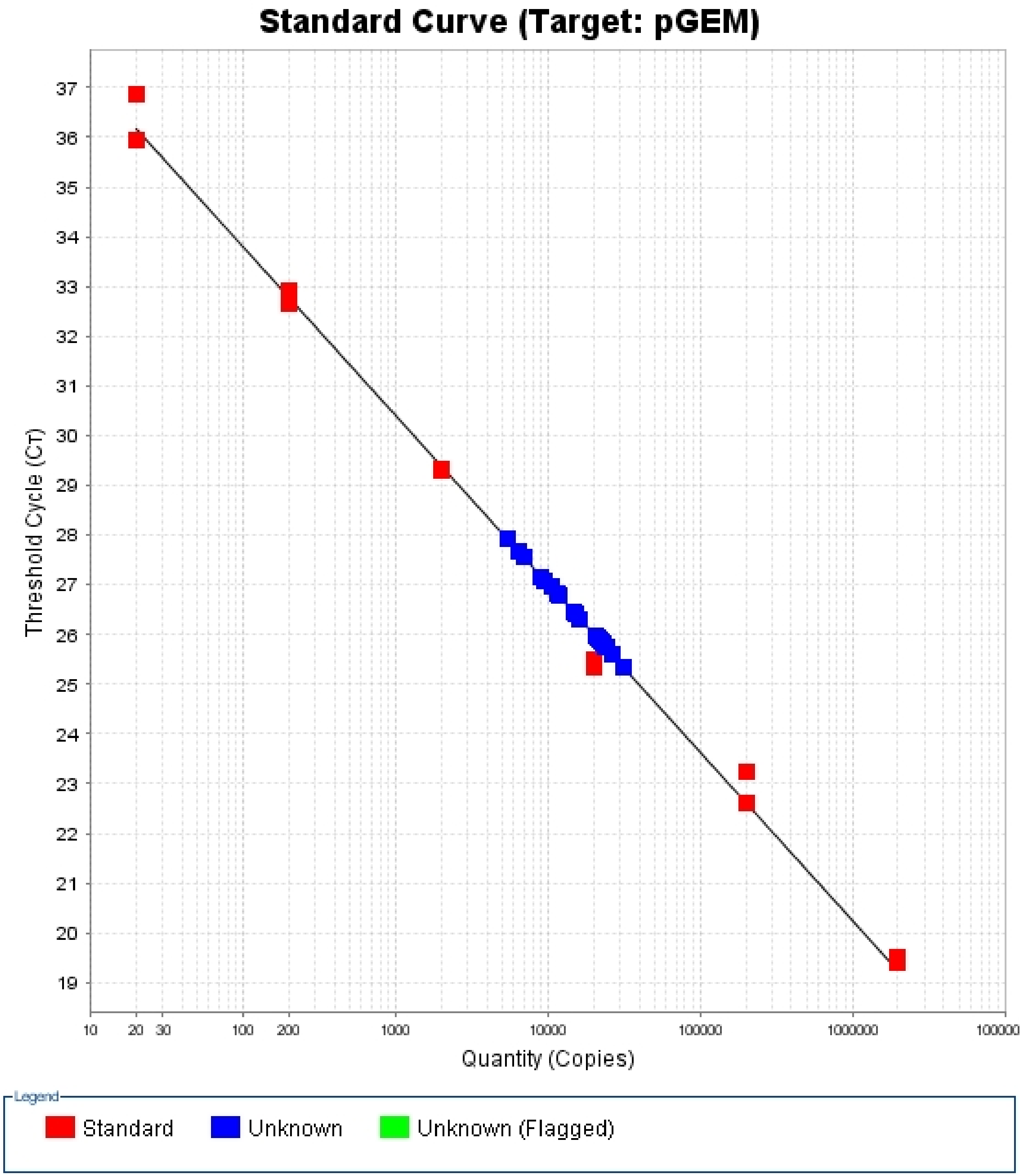

slope:-3.379 Y-Intercept:40.562 R^2:0.996 Eff%:97.664

Results Table

Well	Sample	Target	Task	Quantity	C
A1		pGEM	STANDARD	2000000	19.516
A2		pGEM	STANDARD	2000000	19.3815
A3	2203 A21 SCM	pGEM	UNKNOWN	9178.751	27.1705
A4	2203 A21 SCM	pGEM	UNKNOWN	5455.7412	27.9339
A5	2203 A21 SCM	pGEM	UNKNOWN	9664.1729	27.0948
B1		pGEM	STANDARD	200000	23.2464
B2		pGEM	STANDARD	200000	22.6084
B3	2203 A21 SCM	pGEM	UNKNOWN	23135.959	25.8137
B4	2203 A21 SCM	pGEM	UNKNOWN	23953.3301	25.7627
B5	2203 A21 SCM	pGEM	UNKNOWN	21233.1426	25.9397
C1		pGEM	STANDARD	20000	25.4882
C2		pGEM	STANDARD	20000	25.3374
C3	2203 A21 200	pGEM	UNKNOWN	14912.0127	26.4583
C4	2203 A21 200	pGEM	UNKNOWN	16454.5586	26.3138
C5	2203 A21 200	pGEM	UNKNOWN	15317.6611	26.4189
D1		pGEM	STANDARD	2000	29.3363
D2		pGEM	STANDARD	2000	29.3075
D3	2203 A21 200	pGEM	UNKNOWN	23507.6797	25.7903
D4	2203 A21 200	pGEM	UNKNOWN	23529.4121	25.789
D5	2203 A21 200	pGEM	UNKNOWN	21824.7227	25.8993
E1		pGEM	STANDARD	200	32.6511
E2		pGEM	STANDARD	200	32.9169
E3	2203 A21 1000	pGEM	UNKNOWN	11982.8457	26.7792
E4	2203 A21 1000	pGEM	UNKNOWN	11727.1367	26.8109
E5	2203 A21 1000	pGEM	UNKNOWN	10580.1523	26.9619
F1		pGEM	STANDARD	20	35.9481
F2		pGEM	STANDARD	20	36.8669
F3	2203 A21 1000	pGEM	UNKNOWN	22614.8945	25.8471
F4	2203 A21 1000	pGEM	UNKNOWN	21565.7402	25.9168
F5	2203 A21 1000	pGEM	UNKNOWN	24385.2969	25.7365

Well		Sample	Target	Task	Quantity	C
G3		2203 A21 3000	pGEM	UNKNOWN	7022.7764	27.5634
G4		2203 A21 3000	pGEM	UNKNOWN	7060.6191	27.5555
G5		2203 A21 3000	pGEM	UNKNOWN	6542.7612	27.6673
G12			pGEM	NTC		Undetermined
H1		Comf2E4	pGEM	UNKNOWN	26810.7793	25.5974
H2		Comf2E4	pGEM	UNKNOWN	31593.1445	25.3565
H3		2203 A21 3000	pGEM	UNKNOWN	24353.5918	25.7384
H4		2203 A21 3000	pGEM	UNKNOWN	22983.084	25.8234
H5		2203 A21 3000	pGEM	UNKNOWN	20678.6562	25.9785
H12			pGEM	NTC		Undetermined

QC Summary

Total Wells	96	Processed Wells	40	Targets Used	1
Well Setup	40	Flagged Wells	2	Samples Used	9

Flag	Name	Frequency	Locations
AMPNC	Amplification in negative control	0	
BADROX	Bad passive reference signal	0	
BLFAIL	Baseline algorithm failed	0	
CTFAIL	C algorithm failed	0	
EXPFAIL	Exponential algorithm failed	0	
HIGHSD	High standard deviation in replicate group	2	F1, F2
NOAMP	No amplification	0	
NOISE	Noise higher than others in plate	0	
NOSIGNAL	No signal in well	0	
OFFSCALE	Fluorescence is offscale	0	
OUTLIERRG	Outlier in replicate group	0	
SPIKE	Noise spikes	0	
THOLDFAIL	Thresholding algorithm failed	0	
