# Supplementary material for: Ecological impact of algal-preying protists aplanochytrids (Labyrinthulea) as a source of docosahexaenoic acid for copepods in marine environments
Source: ISME Commun. 2026 Jun 26;6(1):ycag178. doi: 10.1093/ismeco/ycag178 (PMC13685785; doi:10.1093/ismeco/ycag178)
Supplement: Supplementary file 2 [file supplementary_materials_ycag178.zip › Morimoto_ISME_C_3rdrev_supplemental_data.zip!/Morimoto_ISME_C_3rdrev_supplemental_data/Supplemental_Data_2/Standard Curve/DNA recovery/2203/DnaRec2203AlinemajinoN=1_A01-A04.pdf]

Experiment Results Report

230619DnaRec2203AlinemajinoN=1

Experiment Summary

Experiment Name	:	230619DnaRec2203AlinemajinoN=1
Experiment Type	:	Quantitation - Standard Curve
File Name	:	230619DnaRec2203AlinemajiN=1.eds
Run Started	:	2023 Jun 19 1:54:09 PM
Run Finished	:	2023 Jun 19 3:28:45 PM
Run Duration	:	94 minutes 36 seconds
Date Modified	:	2023 Jun 19 3:42:42 PM
User	:	
Number of wells used	:	64
Number of wells with results	:	64
Instrument Name	:	
Instrument Type	:	Applied Biosystems StepOnePlus™ Instrument
Comments	:	

Results Summary

Sample	Target	Quantity (Mean)	Quantity (Std Dev)	C (Mean)	C (Std Dev)
2203 A01 10 ato1	pGEM	44048.89	1250.75	23.40	0.04
2203 A01 10 saki1	pGEM	20554.10	687.19	24.51	0.05
2203 A01 BTM ato1	pGEM	34572.33	1583.44	23.75	0.07
2203 A01 BTM saki1	pGEM	21038.46	2226.14	24.48	0.15
2203 A01 SCM ato1	pGEM	38338.77	674.29	23.60	0.03
2203 A01 SCM saki1	pGEM	21800.57	1384.31	24.42	0.09
2203 A04 10 ato1	pGEM	34254.83	821.89	23.77	0.03
2203 A04 10 saki1	pGEM	20804.47	712.03	24.49	0.05
2203 A04 1000 ato1	pGEM	37506.06	4198.16	23.64	0.16
2203 A04 1000 saki1	pGEM	22011.98	1290.25	24.41	0.09
2203 A04 200 ato1	pGEM	35916.93	1194.62	23.70	0.05
2203 A04 200 saki1	pGEM	20624.64	181.63	24.50	0.01
2203 A04 3000 ato1	pGEM	32593.97	1379.82	23.84	0.06
2203 A04 3000 saki1	pGEM	19409.90	785.96	24.59	0.06
2203 A04 SCM ato1	pGEM	39044.78	956.87	23.58	0.04
2203 A04 SCM saki1	pGEM	19823.21	1547.91	24.56	0.11
Comf2E4	pGEM	18003.74	3031.73	24.71	0.24

Plate Layout

	1	2	3	4	5	6	7	8	9	10	11	12
A	S pGEM<br>2E6<br>FAM-NFQ-MGB<br>CT: 17.94	S pGEM<br>2E6<br>FAM-NFQ-MGB<br>CT: 17.59	2203 A01 10 saki1<br>U pGEM<br>2.06E4<br>FAM-NFQ-MGB<br>CT: 24.5	2203 A01 10 saki1<br>U pGEM<br>2.12E4<br>FAM-NFQ-MGB<br>CT: 24.46	2203 A01 10 saki1<br>U pGEM<br>1.99E4<br>FAM-NFQ-MGB<br>CT: 24.56	2203 A04 SCM saki1<br>U pGEM<br>1.84E4<br>FAM-NFQ-MGB<br>CT: 24.67	2203 A04 SCM saki1<br>U pGEM<br>1.96E4<br>FAM-NFQ-MGB<br>CT: 24.57	2203 A04 SCM saki1<br>U pGEM<br>2.15E4<br>FAM-NFQ-MGB<br>CT: 24.44				
B	S pGEM<br>2E5<br>FAM-NFQ-MGB<br>CT: 21.12	S pGEM<br>2E5<br>FAM-NFQ-MGB<br>CT: 21.12	2203 A01 10 ato1<br>U pGEM<br>4.27E4<br>FAM-NFQ-MGB<br>CT: 23.45	2203 A01 10 ato1<br>U pGEM<br>4.43E4<br>FAM-NFQ-MGB<br>CT: 23.4	2203 A01 10 ato1<br>U pGEM<br>4.52E4<br>FAM-NFQ-MGB<br>CT: 23.37	2203 A04 SCM ato1<br>U pGEM<br>3.8E4<br>FAM-NFQ-MGB<br>CT: 23.62	2203 A04 SCM ato1<br>U pGEM<br>3.98E4<br>FAM-NFQ-MGB<br>CT: 23.55	2203 A04 SCM ato1<br>U pGEM<br>3.94E4<br>FAM-NFQ-MGB<br>CT: 23.56				
C	S pGEM<br>2E4<br>FAM-NFQ-MGB<br>CT: 24.62	S pGEM<br>2E4<br>FAM-NFQ-MGB<br>CT: 24.49	2203 A01 SCM saki1<br>U pGEM<br>2.05E4<br>FAM-NFQ-MGB<br>CT: 24.51	2203 A01 SCM saki1<br>U pGEM<br>2.33E4<br>FAM-NFQ-MGB<br>CT: 24.33	2203 A01 SCM saki1<br>U pGEM<br>2.16E4<br>FAM-NFQ-MGB<br>CT: 24.43	2203 A04 200 saki1<br>U pGEM<br>2.06E4<br>FAM-NFQ-MGB<br>CT: 24.5	2203 A04 200 saki1<br>U pGEM<br>2.04E4<br>FAM-NFQ-MGB<br>CT: 24.51	2203 A04 200 saki1<br>U pGEM<br>2.08E4<br>FAM-NFQ-MGB<br>CT: 24.49				
D	S pGEM<br>2E3<br>FAM-NFQ-MGB<br>CT: 28.21	S pGEM<br>2E3<br>FAM-NFQ-MGB<br>CT: 28	2203 A01 SCM ato1<br>U pGEM<br>3.76E4<br>FAM-NFQ-MGB<br>CT: 23.63	2203 A01 SCM ato1<br>U pGEM<br>3.86E4<br>FAM-NFQ-MGB<br>CT: 23.59	2203 A01 SCM ato1<br>U pGEM<br>3.89E4<br>FAM-NFQ-MGB<br>CT: 23.58	2203 A04 200 ato1<br>U pGEM<br>3.46E4<br>FAM-NFQ-MGB<br>CT: 23.75	2203 A04 200 ato1<br>U pGEM<br>3.62E4<br>FAM-NFQ-MGB<br>CT: 23.68	2203 A04 200 ato1<br>U pGEM<br>3.69E4<br>FAM-NFQ-MGB<br>CT: 23.66				
E	S pGEM<br>200<br>FAM-NFQ-MGB<br>CT: 31.8	S pGEM<br>200<br>FAM-NFQ-MGB<br>CT: 31.53	2203 A01 BTM saki1<br>U pGEM<br>2.04E4<br>FAM-NFQ-MGB<br>CT: 24.52	2203 A01 BTM saki1<br>U pGEM<br>2.35E4<br>FAM-NFQ-MGB<br>CT: 24.31	2203 A01 BTM saki1<br>U pGEM<br>1.92E4<br>FAM-NFQ-MGB<br>CT: 24.6	2203 A04 1000 saki1<br>U pGEM<br>2.21E4<br>FAM-NFQ-MGB<br>CT: 24.4	2203 A04 1000 saki1<br>U pGEM<br>2.32E4<br>FAM-NFQ-MGB<br>CT: 24.33	2203 A04 1000 saki1<br>U pGEM<br>2.07E4<br>FAM-NFQ-MGB<br>CT: 24.5				
F	S pGEM<br>20<br>FAM-NFQ-MGB<br>CT: 34.12	S pGEM<br>20<br>FAM-NFQ-MGB<br>CT: 33.98	2203 A01 BTM ato1<br>U pGEM<br>3.48E4<br>FAM-NFQ-MGB<br>CT: 23.74	2203 A01 BTM ato1<br>U pGEM<br>3.29E4<br>FAM-NFQ-MGB<br>CT: 23.83	2203 A01 BTM ato1<br>U pGEM<br>3.6E4<br>FAM-NFQ-MGB<br>CT: 23.69	2203 A04 1000 ato1<br>U pGEM<br>3.64E4<br>FAM-NFQ-MGB<br>CT: 23.68	2203 A04 1000 ato1<br>U pGEM<br>4.22E4<br>FAM-NFQ-MGB<br>CT: 23.47	2203 A04 1000 ato1<br>U pGEM<br>3.4E4<br>FAM-NFQ-MGB<br>CT: 23.78				
G			2203 A04 10 saki1<br>U pGEM<br>2.04E4<br>FAM-NFQ-MGB<br>CT: 24.52	2203 A04 10 saki1<br>U pGEM<br>2.04E4<br>FAM-NFQ-MGB<br>CT: 24.52	2203 A04 10 saki1<br>U pGEM<br>2.16E4<br>FAM-NFQ-MGB<br>CT: 24.43	2203 A04 3000 saki1<br>U pGEM<br>2.01E4<br>FAM-NFQ-MGB<br>CT: 24.54	2203 A04 3000 saki1<br>U pGEM<br>1.86E4<br>FAM-NFQ-MGB<br>CT: 24.65	2203 A04 3000 saki1<br>U pGEM<br>1.95E4<br>FAM-NFQ-MGB<br>CT: 24.58				N pGEM<br>FAM-NFQ-MGB<br>CT: Undetermined
H	Comf2E4<br>U pGEM<br>1.59E4<br>FAM-NFQ-MGB<br>CT: 24.88	Comf2E4<br>U pGEM<br>2.01E4<br>FAM-NFQ-MGB<br>CT: 24.53	2203 A04 10 ato1<br>U pGEM<br>3.42E4<br>FAM-NFQ-MGB<br>CT: 23.77	2203 A04 10 ato1<br>U pGEM<br>3.35E4<br>FAM-NFQ-MGB<br>CT: 23.8	2203 A04 10 ato1<br>U pGEM<br>3.51E4<br>FAM-NFQ-MGB<br>CT: 23.73	2203 A04 3000 ato1<br>U pGEM<br>3.19E4<br>FAM-NFQ-MGB<br>CT: 23.87	2203 A04 3000 ato1<br>U pGEM<br>3.17E4<br>FAM-NFQ-MGB<br>CT: 23.88	2203 A04 3000 ato1<br>U pGEM<br>3.42E4<br>FAM-NFQ-MGB<br>CT: 23.77				N pGEM<br>FAM-NFQ-MGB<br>CT: 36.98

Standard Curves

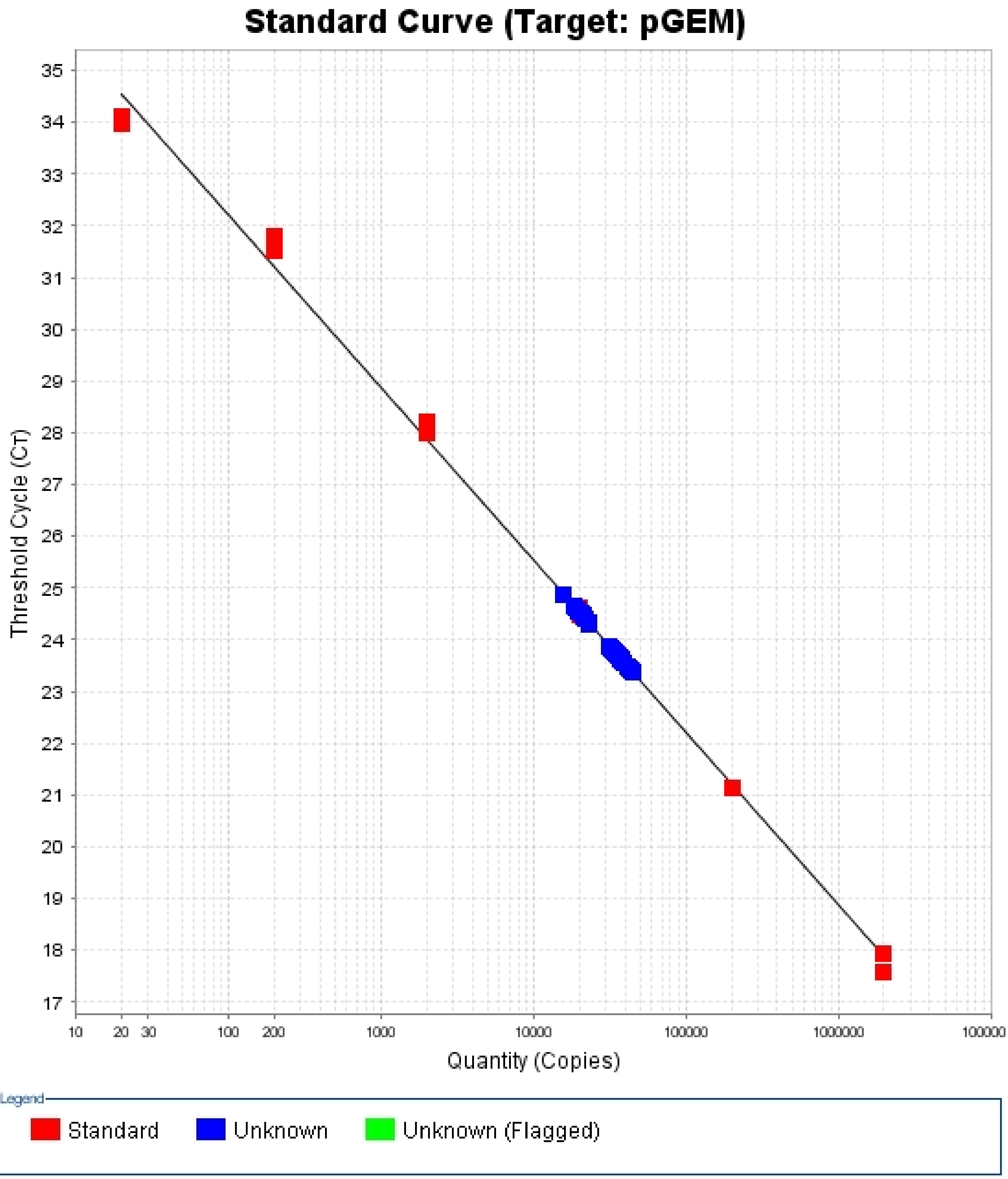

slope:-3.332 Y-Intercept:38.876 R^2:0.997 Eff%:99.577

Results Table

Well		Sample	Target	Task	Quantity	C
A1			pGEM	STANDARD	2000000	17.9396
A2			pGEM	STANDARD	2000000	17.5946
A3		2203 A01 10	pGEM	UNKNOWN	20578.3301	24.5038
A4		2203 A01 10	pGEM	UNKNOWN	21228.8574	24.4587
A5		2203 A01 10	pGEM	UNKNOWN	19855.1172	24.5556
A6		2203 A04 SCM	pGEM	UNKNOWN	18396.9883	24.6659
A7		2203 A04 SCM	pGEM	UNKNOWN	19603.3516	24.574
A8		2203 A04 SCM	pGEM	UNKNOWN	21469.3008	24.4424
B1			pGEM	STANDARD	200000	21.1248
B2			pGEM	STANDARD	200000	21.1182
B3		2203 A01 10	pGEM	UNKNOWN	42708.3477	23.4472
B4		2203 A01 10	pGEM	UNKNOWN	44253.7695	23.3957
B5		2203 A01 10	pGEM	UNKNOWN	45184.543	23.3656
B6		2203 A04 SCM	pGEM	UNKNOWN	37964.5508	23.6176
B7		2203 A04 SCM	pGEM	UNKNOWN	39785.9688	23.5497
B8		2203 A04 SCM	pGEM	UNKNOWN	39383.8125	23.5644
C1			pGEM	STANDARD	20000	24.6153
C2			pGEM	STANDARD	20000	24.4861
C3		2203 A01 SCM	pGEM	UNKNOWN	20522.5332	24.5077
C4		2203 A01 SCM	pGEM	UNKNOWN	23271.0215	24.3258
C5		2203 A01 SCM	pGEM	UNKNOWN	21608.1504	24.4331
C6		2203 A04 200	pGEM	UNKNOWN	20636.5098	24.4997
C7		2203 A04 200	pGEM	UNKNOWN	20437.3691	24.5137
C8		2203 A04 200	pGEM	UNKNOWN	20800.0527	24.4883
D1			pGEM	STANDARD	2000	28.2099
D2			pGEM	STANDARD	2000	28.0027
D3		2203 A01 SCM	pGEM	UNKNOWN	37575.6055	23.6325
D4		2203 A01 SCM	pGEM	UNKNOWN	38586.7695	23.594
D5		2203 A01 SCM	pGEM	UNKNOWN	38853.9375	23.584
D6		2203 A04 200	pGEM	UNKNOWN	34592.3438	23.7522

Well	Sample	Target	Task	Quantity	C
D7	2203 A04 200	pGEM	UNKNOWN	36245.7148	23.6846
D8	2203 A04 200	pGEM	UNKNOWN	36912.7188	23.6582
E1		pGEM	STANDARD	200	31.8029
E2		pGEM	STANDARD	200	31.5343
E3	2203 A01 BTM	pGEM	UNKNOWN	20387.3008	24.5173
E4	2203 A01 BTM	pGEM	UNKNOWN	23517.5723	24.3106
E5	2203 A01 BTM	pGEM	UNKNOWN	19210.5215	24.6033
E6	2203 A04 1000	pGEM	UNKNOWN	22122.6387	24.3991
E7	2203 A04 1000	pGEM	UNKNOWN	23243.3398	24.3276
E8	2203 A04 1000	pGEM	UNKNOWN	20669.9648	24.4974
F1		pGEM	STANDARD	20	34.124
F2		pGEM	STANDARD	20	33.9809
F3	2203 A01 BTM	pGEM	UNKNOWN	34831.1172	23.7422
F4	2203 A01 BTM	pGEM	UNKNOWN	32875.4375	23.8258
F5	2203 A01 BTM	pGEM	UNKNOWN	36010.4336	23.694
F6	2203 A04 1000	pGEM	UNKNOWN	36353.0273	23.6803
F7	2203 A04 1000	pGEM	UNKNOWN	42160.2422	23.4659
F8	2203 A04 1000	pGEM	UNKNOWN	34004.8945	23.777
G3	2203 A04 10	pGEM	UNKNOWN	20380.8789	24.5177
G4	2203 A04 10	pGEM	UNKNOWN	20406.0117	24.516
G5	2203 A04 10	pGEM	UNKNOWN	21626.5273	24.4319
G6	2203 A04 3000	pGEM	UNKNOWN	20133.2168	24.5354
G7	2203 A04 3000	pGEM	UNKNOWN	18573.5312	24.6521
G8	2203 A04 3000	pGEM	UNKNOWN	19522.9551	24.58
G12		pGEM	NTC		Undetermined
H1	Comf2E4	pGEM	UNKNOWN	15859.9912	24.8807
H2	Comf2E4	pGEM	UNKNOWN	20147.498	24.5344
H3	2203 A04 10	pGEM	UNKNOWN	34217.6875	23.7679
H4	2203 A04 10	pGEM	UNKNOWN	33452.1484	23.8007
H5	2203 A04 10	pGEM	UNKNOWN	35094.6602	23.7313
H6	2203 A04 3000	pGEM	UNKNOWN	31854.623	23.8715
H7	2203 A04 3000	pGEM	UNKNOWN	31741.3789	23.8766

Well		Sample	Target	Task	Quantity	C
H8		2203 A04 3000	pGEM	UNKNOWN	34185.9062	23.7693
H12			pGEM	NTC		36.9756

QC Summary

Total Wells	96	Processed Wells	64	Targets Used	1
Well Setup	64	Flagged Wells	0	Samples Used	17

Flag	Name	Frequency	Locations
AMPNC	Amplification in negative control	0	
BADROX	Bad passive reference signal	0	
BLFAIL	Baseline algorithm failed	0	
CTFAIL	C algorithm failed	0	
EXPFAIL	Exponential algorithm failed	0	
HIGHSD	High standard deviation in replicate group	0	
NOAMP	No amplification	0	
NOISE	Noise higher than others in plate	0	
NOSIGNAL	No signal in well	0	
OFFSCALE	Fluorescence is offscale	0	
OUTLIERRG	Outlier in replicate group	0	
SPIKE	Noise spikes	0	
THOLDFAIL	Thresholding algorithm failed	0	
