# Supplementary material for: Ecological impact of algal-preying protists aplanochytrids (Labyrinthulea) as a source of docosahexaenoic acid for copepods in marine environments
Source: ISME Commun. 2026 Jun 26;6(1):ycag178. doi: 10.1093/ismeco/ycag178 (PMC13685785; doi:10.1093/ismeco/ycag178)
Supplement: Supplementary file 2 [file supplementary_materials_ycag178.zip › Morimoto_ISME_C_3rdrev_supplemental_data.zip!/Morimoto_ISME_C_3rdrev_supplemental_data/Supplemental_Data_2/Standard Curve/DNA recovery/2203/DnaRec2203AlineA11-3000saki1.pdf]

Experiment Results Report

230607DnaRec2203AlineA113000saki1

Experiment Summary

Experiment Name	:	230607DnaRec2203AlineA113000saki1
Experiment Type	:	Quantitation - Standard Curve
File Name	:	230607DnaRec2203AlineA113000saki1.eds
Run Started	:	2023 Jun 08 4:12:32 PM
Run Finished	:	2023 Jun 08 5:47:07 PM
Run Duration	:	94 minutes 34 seconds
Date Modified	:	2023 Jun 09 6:57:50 PM
User	:	
Number of wells used	:	19
Number of wells with results	:	19
Instrument Name	:	
Instrument Type	:	Applied Biosystems StepOnePlus™ Instrument
Comments	:	

Results Summary

Sample	Target	Quantity (Mean)	Quantity (Std Dev)	C (Mean)	C (Std Dev)
2203 A11 3000m	pGEM	17697.08	937.12	23.04	0.08
Comf2E4	pGEM	18954.76	3182.42	22.95	0.24

Plate Layout

	1	2	3	4	5	6	7	8	9	10	11	12
A	S pGEM<br>2E6<br>FAM-NFQ-MGB<br>CT: 16.65	S pGEM<br>2E6<br>FAM-NFQ-MGB<br>CT: 15.98	2203 A11 3000m saki1<br>U pGEM<br>1.81E4<br>FAM-NFQ-MGB<br>CT: 23	2203 A11 3000m saki1<br>U pGEM<br>1.66E4<br>FAM-NFQ-MGB<br>CT: 23.13	2203 A11 3000m saki1<br>U pGEM<br>1.84E4<br>FAM-NFQ-MGB<br>CT: 22.98							
B	S pGEM<br>2E5<br>FAM-NFQ-MGB<br>CT: 19.58	S pGEM<br>2E5<br>FAM-NFQ-MGB<br>CT: 19.26										
C	S pGEM<br>2E4<br>FAM-NFQ-MGB<br>CT: 22.9	S pGEM<br>2E4<br>FAM-NFQ-MGB<br>CT: 22.72										
D	S pGEM<br>2E3<br>FAM-NFQ-MGB<br>CT: 26.25	S pGEM<br>2E3<br>FAM-NFQ-MGB<br>CT: 26.17										
E	S pGEM<br>200<br>FAM-NFQ-MGB<br>CT: 29.7	S pGEM<br>200<br>FAM-NFQ-MGB<br>CT: 29.82										
F	S pGEM<br>20<br>FAM-NFQ-MGB<br>CT: 32.48	S pGEM<br>20<br>FAM-NFQ-MGB<br>CT: 32.62										
G												N pGEM<br>FAM-NFQ-MGB<br>CT: 37.12
H	Comf2E4<br>U pGEM<br>1.67E4<br>FAM-NFQ-MGB<br>CT: 23.12	Comf2E4<br>U pGEM<br>2.12E4<br>FAM-NFQ-MGB<br>CT: 22.78										N pGEM<br>FAM-NFQ-MGB<br>CT: Undetermined

Standard Curves

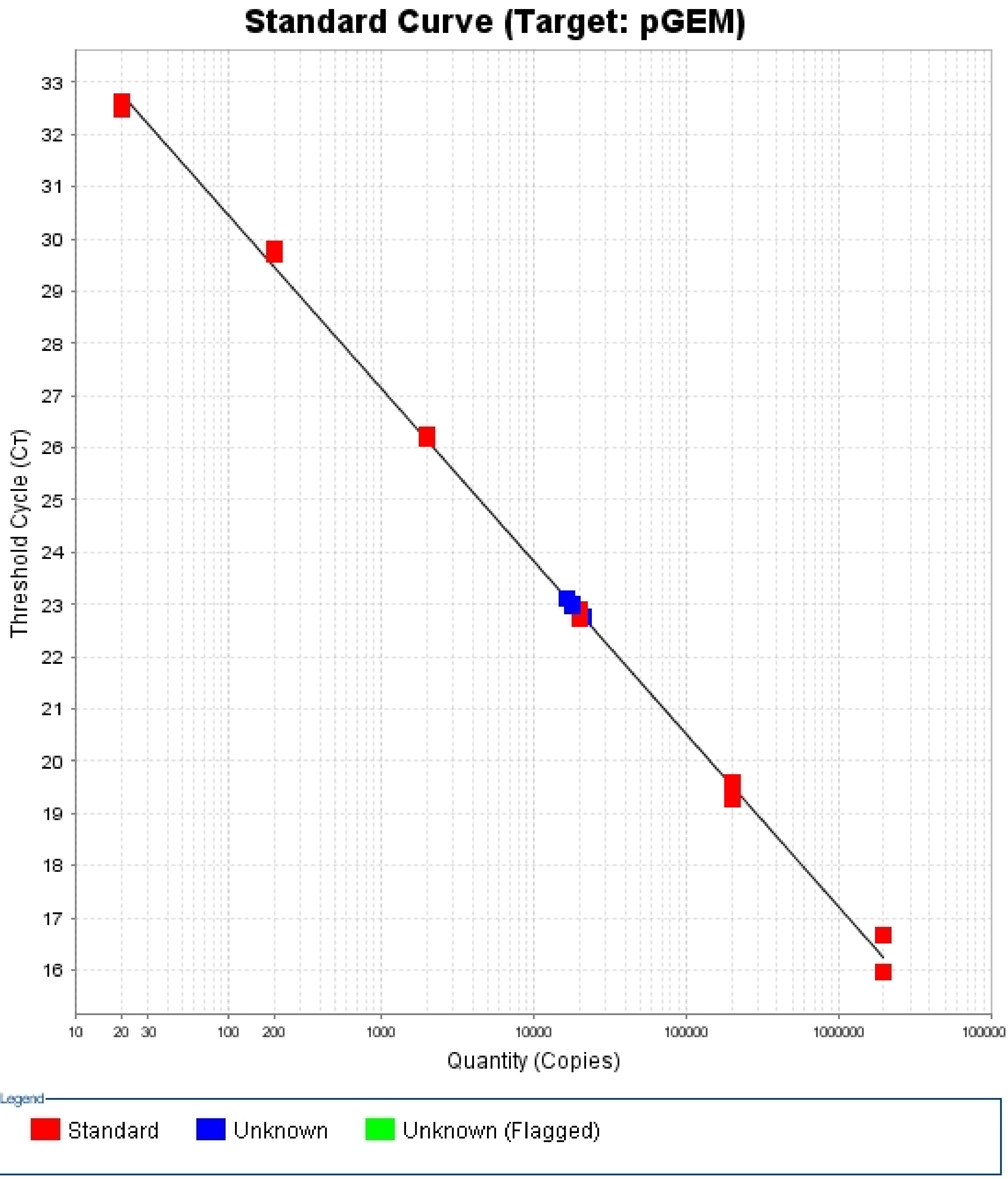

slope:-3.303 Y-Intercept:37.066 R^2:0.998 Eff%:100.804

Results Table

Well		Sample	Target	Task	Quantity	C
A1			pGEM	STANDARD	2000000	16.6534
A2			pGEM	STANDARD	2000000	15.979
A3		2203 A11	pGEM	UNKNOWN	18086.0254	23.0045
A4		2203 A11	pGEM	UNKNOWN	16628.1211	23.1251
A5		2203 A11	pGEM	UNKNOWN	18377.1055	22.9816
B1			pGEM	STANDARD	200000	19.5778
B2			pGEM	STANDARD	200000	19.2576
C1			pGEM	STANDARD	20000	22.9008
C2			pGEM	STANDARD	20000	22.7247
D1			pGEM	STANDARD	2000	26.2518
D2			pGEM	STANDARD	2000	26.17
E1			pGEM	STANDARD	200	29.7031
E2			pGEM	STANDARD	200	29.8212
F1			pGEM	STANDARD	20	32.4777
F2			pGEM	STANDARD	20	32.6218
G12			pGEM	NTC		37.1223
H1		Comf2E4	pGEM	UNKNOWN	16704.4453	23.1185
H2		Comf2E4	pGEM	UNKNOWN	21205.0723	22.7763
H12			pGEM	NTC		Undetermined

QC Summary

Total Wells	96	Processed Wells	19	Targets Used	1
Well Setup	19	Flagged Wells	0	Samples Used	2

Flag	Name	Frequency	Locations
AMPNC	Amplification in negative control	0	
BADROX	Bad passive reference signal	0	
BLFAIL	Baseline algorithm failed	0	
CTFAIL	C algorithm failed	0	
EXPFAIL	Exponential algorithm failed	0	
HIGHSD	High standard deviation in replicate group	0	
NOAMP	No amplification	0	
NOISE	Noise higher than others in plate	0	
NOSIGNAL	No signal in well	0	
OFFSCALE	Fluorescence is offscale	0	
OUTLIERRG	Outlier in replicate group	0	
SPIKE	Noise spikes	0	
THOLDFAIL	Thresholding algorithm failed	0	
