# Supplementary material for: Ecological impact of algal-preying protists aplanochytrids (Labyrinthulea) as a source of docosahexaenoic acid for copepods in marine environments
Source: ISME Commun. 2026 Jun 26;6(1):ycag178. doi: 10.1093/ismeco/ycag178 (PMC13685785; doi:10.1093/ismeco/ycag178)
Supplement: Supplementary file 2 [file supplementary_materials_ycag178.zip › Morimoto_ISME_C_3rdrev_supplemental_data.zip!/Morimoto_ISME_C_3rdrev_supplemental_data/Supplemental_Data_2/Standard Curve/DNA recovery/2203/DnaRec2203AlineA07-A11N=3.pdf]

Experiment Results Report

230621DnaRec2203AlineA07-A11N=3

Experiment Summary

Experiment Name	:	230621DnaRec2203AlineA07-A11N=3
Experiment Type	:	Quantitation - Standard Curve
File Name	:	230621DnaRec2203AlineA07-A11N=3.eds
Run Started	:	2023 Jun 21 2:48:36 PM
Run Finished	:	2023 Jun 21 4:23:17 PM
Run Duration	:	94 minutes 40 seconds
Date Modified	:	2023 Jun 21 5:23:18 PM
User	:	
Number of wells used	:	70
Number of wells with results	:	70
Instrument Name	:	
Instrument Type	:	Applied Biosystems StepOnePlus™ Instrument
Comments	:	

Results Summary

Sample	Target	Quantity (Mean)	Quantity (Std Dev)	C (Mean)	C (Std Dev)
2203 A07 10 ato3	pGEM	22694.02	1286.09	25.49	0.09
2203 A07 10 saki3	pGEM	11907.10	901.62	26.44	0.11
2203 A07 1000 ato3	pGEM	21791.93	1405.94	25.55	0.10
2203 A07 1000 saki3	pGEM	10739.64	570.30	26.60	0.08
2203 A07 200 ato3	pGEM	21190.21	810.05	25.59	0.06
2203 A07 200 saki3	pGEM	12947.15	337.18	26.32	0.04
2203 A07 3000 ato3	pGEM	20566.51	841.21	25.63	0.06
2203 A07 3000 saki3	pGEM	9293.15	285.56	26.81	0.05
2203 A07 SCM ato3	pGEM	21605.81	1412.95	25.56	0.10
2203 A07 SCM saki3	pGEM	11881.92	856.97	26.45	0.11
2203 A11 1000 ato3	pGEM	19117.05	2407.12	25.75	0.18
2203 A11 1000 saki3	pGEM	13367.42	933.96	26.27	0.10
2203 A11 200 ato3	pGEM	19264.10	1751.47	25.73	0.13
2203 A11 200 saki3	pGEM	11904.29	563.36	26.44	0.07
2203 A11 3000 ato3	pGEM	20291.61	788.53	25.65	0.06
2203 A11 3000 saki3	pGEM	11795.49	440.11	26.46	0.06
2203 A11 SCM ato3	pGEM	20653.40	1600.14	25.63	0.12
2203 A11 SCM saki3	pGEM	9995.59	324.06	26.70	0.05
Comf2E4	pGEM	25640.35	3632.10	25.31	0.21

Plate Layout

	1	2	3	4	5	6	7	8	9	10	11	12
A	2203 A11 SCM saki3<br>U pGEM<br>1.03E4<br>FAM-NFQ-MGB<br>CT: 26.66	2203 A11 SCM saki3<br>U pGEM<br>9.63E3<br>FAM-NFQ-MGB<br>CT: 26.75	2203 A11 SCM saki3<br>U pGEM<br>1.01E4<br>FAM-NFQ-MGB<br>CT: 26.69	S pGEM<br>2E6<br>FAM-NFQ-MGB<br>CT: 19.11	S pGEM<br>2E6<br>FAM-NFQ-MGB<br>CT: 19.13	2203 A07 3000 saki3<br>U pGEM<br>8.97E3<br>FAM-NFQ-MGB<br>CT: 26.86	2203 A07 10 saki3<br>U pGEM<br>1.09E4<br>FAM-NFQ-MGB<br>CT: 26.57	2203 A07 10 saki3<br>U pGEM<br>1.27E4<br>FAM-NFQ-MGB<br>CT: 26.34	2203 A07 10 saki3<br>U pGEM<br>1.21E4<br>FAM-NFQ-MGB<br>CT: 26.42			
B	2203 A11 SCM ato3<br>U pGEM<br>1.88E4<br>FAM-NFQ-MGB<br>CT: 25.76	2203 A11 SCM ato3<br>U pGEM<br>2.18E4<br>FAM-NFQ-MGB<br>CT: 25.54	2203 A11 SCM ato3<br>U pGEM<br>2.13E4<br>FAM-NFQ-MGB<br>CT: 25.58	S pGEM<br>2E5<br>FAM-NFQ-MGB<br>CT: 22.45	S pGEM<br>2E5<br>FAM-NFQ-MGB<br>CT: 22.47	2203 A07 3000 saki3<br>U pGEM<br>9.53E3<br>FAM-NFQ-MGB<br>CT: 26.77	2203 A07 10 ato3<br>U pGEM<br>2.12E4<br>FAM-NFQ-MGB<br>CT: 25.58	2203 A07 10 ato3<br>U pGEM<br>2.36E4<br>FAM-NFQ-MGB<br>CT: 25.43	2203 A07 10 ato3<br>U pGEM<br>2.33E4<br>FAM-NFQ-MGB<br>CT: 25.45			
C	2203 A11 200 saki3<br>U pGEM<br>1.25E4<br>FAM-NFQ-MGB<br>CT: 26.37	2203 A11 200 saki3<br>U pGEM<br>1.13E4<br>FAM-NFQ-MGB<br>CT: 26.51	2203 A11 200 saki3<br>U pGEM<br>1.19E4<br>FAM-NFQ-MGB<br>CT: 26.44	S pGEM<br>2E4<br>FAM-NFQ-MGB<br>CT: 25.08	S pGEM<br>2E4<br>FAM-NFQ-MGB<br>CT: 25.09	2203 A07 3000 saki3<br>U pGEM<br>9.38E3<br>FAM-NFQ-MGB<br>CT: 26.79	2203 A07 SCM saki3<br>U pGEM<br>1.22E4<br>FAM-NFQ-MGB<br>CT: 26.4	2203 A07 SCM saki3<br>U pGEM<br>1.25E4<br>FAM-NFQ-MGB<br>CT: 26.36	2203 A07 SCM saki3<br>U pGEM<br>1.09E4<br>FAM-NFQ-MGB<br>CT: 26.57			
D	2203 A11 200 ato3<br>U pGEM<br>1.86E4<br>FAM-NFQ-MGB<br>CT: 25.78	2203 A11 200 ato3<br>U pGEM<br>1.79E4<br>FAM-NFQ-MGB<br>CT: 25.83	2203 A11 200 ato3<br>U pGEM<br>2.12E4<br>FAM-NFQ-MGB<br>CT: 25.58	S pGEM<br>2E3<br>FAM-NFQ-MGB<br>CT: 28.79	S pGEM<br>2E3<br>FAM-NFQ-MGB<br>CT: 29.07	2203 A07 3000 ato3<br>U pGEM<br>2.1E4<br>FAM-NFQ-MGB<br>CT: 25.6	2203 A07 SCM ato3<br>U pGEM<br>2.3E4<br>FAM-NFQ-MGB<br>CT: 25.47	2203 A07 SCM ato3<br>U pGEM<br>2.01E4<br>FAM-NFQ-MGB<br>CT: 25.66	2203 A07 SCM ato3<br>U pGEM<br>2.17E4<br>FAM-NFQ-MGB<br>CT: 25.55			
E	2203 A11 1000 saki3<br>U pGEM<br>1.28E4<br>FAM-NFQ-MGB<br>CT: 26.33	2203 A11 1000 saki3<br>U pGEM<br>1.28E4<br>FAM-NFQ-MGB<br>CT: 26.33	2203 A11 1000 saki3<br>U pGEM<br>1.44E4<br>FAM-NFQ-MGB<br>CT: 26.15	S pGEM<br>200<br>FAM-NFQ-MGB<br>CT: 32.39	S pGEM<br>200<br>FAM-NFQ-MGB<br>CT: 32.42	2203 A07 3000 ato3<br>U pGEM<br>1.96E4<br>FAM-NFQ-MGB<br>CT: 25.7	2203 A07 200 saki3<br>U pGEM<br>1.32E4<br>FAM-NFQ-MGB<br>CT: 26.29	2203 A07 200 saki3<br>U pGEM<br>1.26E4<br>FAM-NFQ-MGB<br>CT: 26.36	2203 A07 200 ato3<br>U pGEM<br>2.19E4<br>FAM-NFQ-MGB<br>CT: 25.54			
F	2203 A11 1000 ato3<br>U pGEM<br>1.83E4<br>FAM-NFQ-MGB<br>CT: 25.8	2203 A11 1000 ato3<br>U pGEM<br>2.18E4<br>FAM-NFQ-MGB<br>CT: 25.54	2203 A11 1000 ato3<br>U pGEM<br>1.72E4<br>FAM-NFQ-MGB<br>CT: 25.89	S pGEM<br>20<br>FAM-NFQ-MGB<br>CT: 36.6	S pGEM<br>20<br>FAM-NFQ-MGB<br>CT: 35.95	2203 A07 3000 ato3<br>U pGEM<br>2.11E4<br>FAM-NFQ-MGB<br>CT: 25.59	2203 A07 200 saki3<br>U pGEM<br>1.31E4<br>FAM-NFQ-MGB<br>CT: 26.3	2203 A07 200 ato3<br>U pGEM<br>2.03E4<br>FAM-NFQ-MGB<br>CT: 25.65	2203 A07 200 ato3<br>U pGEM<br>2.14E4<br>FAM-NFQ-MGB<br>CT: 25.57			
G			2203 A11 3000 saki3<br>U pGEM<br>1.21E4<br>FAM-NFQ-MGB<br>CT: 26.42	2203 A11 3000 saki3<br>U pGEM<br>1.2E4<br>FAM-NFQ-MGB<br>CT: 26.43	2203 A11 3000 saki3<br>U pGEM<br>1.13E4<br>FAM-NFQ-MGB<br>CT: 26.52		2203 A07 1000 saki3<br>U pGEM<br>1.02E4<br>FAM-NFQ-MGB<br>CT: 26.67	2203 A07 1000 saki3<br>U pGEM<br>1.06E4<br>FAM-NFQ-MGB<br>CT: 26.61	2203 A07 1000 saki3<br>U pGEM<br>1.14E4<br>FAM-NFQ-MGB<br>CT: 26.51			N pGEM<br>FAM-NFQ-MGB<br>CT: Undetermined
H	Comf2E4<br>U pGEM<br>2.31E4<br>FAM-NFQ-MGB<br>CT: 25.46	Comf2E4<br>U pGEM<br>2.82E4<br>FAM-NFQ-MGB<br>CT: 25.16	2203 A11 3000 ato3<br>U pGEM<br>1.94E4<br>FAM-NFQ-MGB<br>CT: 25.71	2203 A11 3000 ato3<br>U pGEM<br>2.04E4<br>FAM-NFQ-MGB<br>CT: 25.64	2203 A11 3000 ato3<br>U pGEM<br>2.1E4<br>FAM-NFQ-MGB<br>CT: 25.6		2203 A07 1000 ato3<br>U pGEM<br>2.03E4<br>FAM-NFQ-MGB<br>CT: 25.65	2203 A07 1000 ato3<br>U pGEM<br>2.19E4<br>FAM-NFQ-MGB<br>CT: 25.54	2203 A07 1000 ato3<br>U pGEM<br>2.31E4<br>FAM-NFQ-MGB<br>CT: 25.46			N pGEM<br>FAM-NFQ-MGB<br>CT: Undetermined

Standard Curves

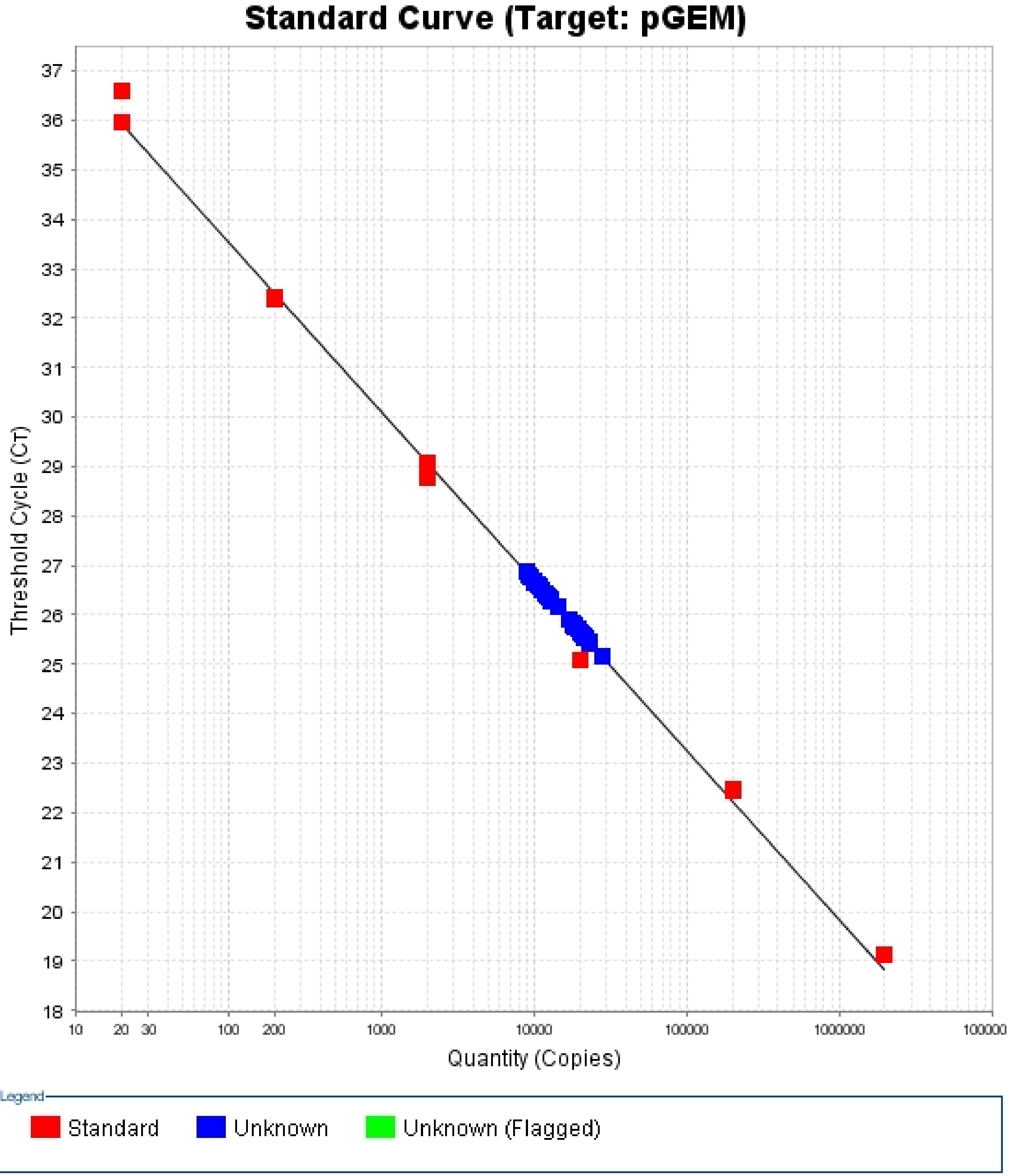

slope:-3.412 Y-Intercept:40.349 R^2:0.996 Eff%:96.362

Results Table

Well		Sample	Target	Task	Quantity	C
A1		2203 A11 SCM	pGEM	UNKNOWN	10258.8389	26.6615
A2		2203 A11 SCM	pGEM	UNKNOWN	9633.6582	26.7547
A3		2203 A11 SCM	pGEM	UNKNOWN	10094.2812	26.6855
A4			pGEM	STANDARD	2000000	19.1134
A5			pGEM	STANDARD	2000000	19.1306
A6		2203 A07 3000	pGEM	UNKNOWN	8974.1992	26.8598
A7		2203 A07 10	pGEM	UNKNOWN	10936.5645	26.5667
A8		2203 A07 10	pGEM	UNKNOWN	12718.6523	26.343
A9		2203 A07 10	pGEM	UNKNOWN	12066.0928	26.4211
B1		2203 A11 SCM	pGEM	UNKNOWN	18830.5117	25.7615
B2		2203 A11 SCM	pGEM	UNKNOWN	21826.1074	25.5427
B3		2203 A11 SCM	pGEM	UNKNOWN	21303.584	25.5786
B4			pGEM	STANDARD	200000	22.4485
B5			pGEM	STANDARD	200000	22.4672
B6		2203 A07 3000	pGEM	UNKNOWN	9525.0752	26.7715
B7		2203 A07 10	pGEM	UNKNOWN	21218.209	25.5846
B8		2203 A07 10	pGEM	UNKNOWN	23575.168	25.4285
B9		2203 A07 10	pGEM	UNKNOWN	23288.668	25.4466
C1		2203 A11 200	pGEM	UNKNOWN	12473.8887	26.3718
C2		2203 A11 200	pGEM	UNKNOWN	11347.3838	26.5121
C3		2203 A11 200	pGEM	UNKNOWN	11891.585	26.4427
C4			pGEM	STANDARD	20000	25.075
C5			pGEM	STANDARD	20000	25.0936
C6		2203 A07 3000	pGEM	UNKNOWN	9380.168	26.7942
C7		2203 A07 SCM	pGEM	UNKNOWN	12200.4844	26.4047
C8		2203 A07 SCM	pGEM	UNKNOWN	12533.9814	26.3647
C9		2203 A07 SCM	pGEM	UNKNOWN	10911.2852	26.5702
D1		2203 A11 200	pGEM	UNKNOWN	18603.6113	25.7795
D2		2203 A11 200	pGEM	UNKNOWN	17938.9062	25.8334
D3		2203 A11 200	pGEM	UNKNOWN	21249.7734	25.5824

Well		Sample	Target	Task	Quantity	C
D4			pGEM	STANDARD	2000	28.7914
D5			pGEM	STANDARD	2000	29.0684
D6		2203 A07 3000	pGEM	UNKNOWN	20990.4805	25.6006
D7		2203 A07 SCM	pGEM	UNKNOWN	22962.0762	25.4675
D8		2203 A07 SCM	pGEM	UNKNOWN	20142.2852	25.6617
D9		2203 A07 SCM	pGEM	UNKNOWN	21713.0801	25.5504
E1		2203 A11 1000	pGEM	UNKNOWN	12825.1055	26.3307
E2		2203 A11 1000	pGEM	UNKNOWN	12831.2969	26.3299
E3		2203 A11 1000	pGEM	UNKNOWN	14445.8525	26.1543
E4			pGEM	STANDARD	200	32.3862
E5			pGEM	STANDARD	200	32.4194
E6		2203 A07 3000	pGEM	UNKNOWN	19597.6738	25.7023
E7		2203 A07 200	pGEM	UNKNOWN	13166.1484	26.2918
E8		2203 A07 200	pGEM	UNKNOWN	12558.8652	26.3618
E9		2203 A07 200	pGEM	UNKNOWN	21885.6582	25.5387
F1		2203 A11 1000	pGEM	UNKNOWN	18316.3457	25.8025
F2		2203 A11 1000	pGEM	UNKNOWN	21822.4824	25.543
F3		2203 A11 1000	pGEM	UNKNOWN	17212.3359	25.8946
F4			pGEM	STANDARD	20	36.5972
F5			pGEM	STANDARD	20	35.9475
F6		2203 A07 3000	pGEM	UNKNOWN	21111.373	25.5921
F7		2203 A07 200	pGEM	UNKNOWN	13116.4219	26.2974
F8		2203 A07 200	pGEM	UNKNOWN	20300.7852	25.6501
F9		2203 A07 200	pGEM	UNKNOWN	21384.1836	25.573
G3		2203 A11 3000	pGEM	UNKNOWN	12093.2227	26.4177
G4		2203 A11 3000	pGEM	UNKNOWN	12003.2988	26.4288
G5		2203 A11 3000	pGEM	UNKNOWN	11289.958	26.5196
G7		2203 A07 1000	pGEM	UNKNOWN	10220.791	26.667
G8		2203 A07 1000	pGEM	UNKNOWN	10647.8594	26.6064
G9		2203 A07 1000	pGEM	UNKNOWN	11350.2617	26.5117
G12			pGEM	NTC		Undetermined
H1		Comf2E4	pGEM	UNKNOWN	23072.0703	25.4604

Well		Sample	Target	Task	Quantity	C
H2		Comf2E4	pGEM	UNKNOWN	28208.6328	25.1626
H3		2203 A11 3000	pGEM	UNKNOWN	19438.582	25.7144
H4		2203 A11 3000	pGEM	UNKNOWN	20442.3691	25.6398
H5		2203 A11 3000	pGEM	UNKNOWN	20993.8848	25.6003
H7		2203 A07 1000	pGEM	UNKNOWN	20326.7207	25.6482
H8		2203 A07 1000	pGEM	UNKNOWN	21919.1191	25.5364
H9		2203 A07 1000	pGEM	UNKNOWN	23129.959	25.4567
H12			pGEM	NTC		Undetermined

QC Summary

Total Wells	96	Processed Wells	70	Targets Used	1
Well Setup	70	Flagged Wells	0	Samples Used	19

Flag	Name	Frequency	Locations
AMPNC	Amplification in negative control	0	
BADROX	Bad passive reference signal	0	
BLFAIL	Baseline algorithm failed	0	
CTFAIL	C algorithm failed	0	
EXPFAIL	Exponential algorithm failed	0	
HIGHSD	High standard deviation in replicate group	0	
NOAMP	No amplification	0	
NOISE	Noise higher than others in plate	0	
NOSIGNAL	No signal in well	0	
OFFSCALE	Fluorescence is offscale	0	
OUTLIERRG	Outlier in replicate group	0	
SPIKE	Noise spikes	0	
THOLDFAIL	Thresholding algorithm failed	0	
