# Supplementary material for: Ecological impact of algal-preying protists aplanochytrids (Labyrinthulea) as a source of docosahexaenoic acid for copepods in marine environments
Source: ISME Commun. 2026 Jun 26;6(1):ycag178. doi: 10.1093/ismeco/ycag178 (PMC13685785; doi:10.1093/ismeco/ycag178)
Supplement: Supplementary file 2 [file supplementary_materials_ycag178.zip › Morimoto_ISME_C_3rdrev_supplemental_data.zip!/Morimoto_ISME_C_3rdrev_supplemental_data/Supplemental_Data_2/Standard Curve/DNA recovery/2203/DnaRec2203AlineA01-A-4N=3.pdf]

# Experiment Results Report

## 230621DnaRec2203AlineA01-A04N=3

## Experiment Summary

| | | |
|---|---|---|
| Experiment Name | : | 230621DnaRec2203AlineA01-A04N=3 |
| Experiment Type | : | Quantitation - Standard Curve |
| File Name | : | 230621DnaRec2203AlineA01-A04N=3.eds |
| Run Started | : | 2023 Jun 21 12:16:21 PM |
| Run Finished | : | 2023 Jun 21 1:51:00 PM |
| Run Duration | : | 94 minutes 39 seconds |
| Date Modified | : | 2023 Jun 21 2:02:20 PM |
| User | : | |
| Number of wells used | : | 64 |
| Number of wells with results | : | 64 |
| Instrument Name | : | |
| Instrument Type | : | Applied Biosystems StepOnePlus™ Instrument |
| Comments | : | |

# Results Summary

| Sample | Target | Quantity (Mean) | Quantity (Std Dev) | C (Mean) | C (Std Dev) |
|---|---|---|---|---|---|
| 2203 A01 10 ato3 | pGEM | 30686.10 | 1425.87 | 25.20 | 0.07 |
| 2203 A01 10 saki3 | pGEM | 19353.22 | 322.56 | 25.89 | 0.02 |
| 2203 A01 BTM ato3 | pGEM | 29481.69 | 2601.82 | 25.27 | 0.13 |
| 2203 A01 BTM saki3 | pGEM | 20063.15 | 415.74 | 25.83 | 0.03 |
| 2203 A01 SCM ato3 | pGEM | 32641.74 | 785.75 | 25.11 | 0.04 |
| 2203 A01 SCM saki3 | pGEM | 18200.84 | 852.55 | 25.98 | 0.07 |
| 2203 A04 10 ato3 | pGEM | 30804.31 | 558.22 | 25.20 | 0.03 |
| 2203 A04 10 saki3 | pGEM | 17801.12 | 214.65 | 26.01 | 0.02 |
| 2203 A04 1000 ato3 | pGEM | 32113.32 | 1704.33 | 25.14 | 0.08 |
| 2203 A04 1000 saki3 | pGEM | 19979.45 | 1291.90 | 25.84 | 0.10 |
| 2203 A04 200 ato3 | pGEM | 32498.29 | 1292.75 | 25.12 | 0.06 |
| 2203 A04 200 saki3 | pGEM | 11597.10 | 573.75 | 26.65 | 0.07 |
| 2203 A04 3000 ato3 | pGEM | 31395.71 | 1470.32 | 25.17 | 0.07 |
| 2203 A04 3000 saki3 | pGEM | 14595.13 | 566.39 | 26.31 | 0.06 |
| 2203 A04 SCM ato3 | pGEM | 32896.89 | 1177.86 | 25.10 | 0.05 |
| 2203 A04 SCM saki3 | pGEM | 17591.80 | 985.82 | 26.03 | 0.08 |
| Comf2E4 | pGEM | 35434.62 | 2059.53 | 24.99 | 0.09 |

# Plate Layout

| | 1 | 2 | 3 | 4 | 5 | 6 | 7 | 8 | 9 | 10 | 11 | 12 |
|---|---|---|---|---|---|---|---|---|---|---|---|---|
| A | S pGEM<br>2E6<br>FAM-NFQ-MGB<br>CT: 18.81 | S pGEM<br>2E6<br>FAM-NFQ-MGB<br>CT: 18.7 | 2203 A01 10 saki3<br>U pGEM<br>1.91E4<br>FAM-NFQ-MGB<br>CT: 25.91 | 2203 A01 10 saki3<br>U pGEM<br>1.97E4<br>FAM-NFQ-MGB<br>CT: 25.86 | 2203 A01 10 saki3<br>U pGEM<br>1.92E4<br>FAM-NFQ-MGB<br>CT: 25.9 | 2203 A04 SCM saki3<br>U pGEM<br>1.71E4<br>FAM-NFQ-MGB<br>CT: 26.07 | 2203 A04 SCM saki3<br>U pGEM<br>1.87E4<br>FAM-NFQ-MGB<br>CT: 25.93 | 2203 A04 SCM saki3<br>U pGEM<br>1.7E4<br>FAM-NFQ-MGB<br>CT: 26.08 | | | | |
| B | 1<br>S pGEM<br>2E5<br>FAM-NFQ-MGB<br>CT: 23.14 | 1<br>S pGEM<br>2E5<br>FAM-NFQ-MGB<br>CT: 22.43 | 2203 A01 10 ato3<br>U pGEM<br>3.17E4<br>FAM-NFQ-MGB<br>CT: 25.15 | 2203 A01 10 ato3<br>U pGEM<br>3.13E4<br>FAM-NFQ-MGB<br>CT: 25.17 | 2203 A01 10 ato3<br>U pGEM<br>2.91E4<br>FAM-NFQ-MGB<br>CT: 25.28 | 2203 A04 SCM ato3<br>U pGEM<br>3.27E4<br>FAM-NFQ-MGB<br>CT: 25.11 | 2203 A04 SCM ato3<br>U pGEM<br>3.18E4<br>FAM-NFQ-MGB<br>CT: 25.15 | 2203 A04 SCM ato3<br>U pGEM<br>3.42E4<br>FAM-NFQ-MGB<br>CT: 25.04 | | | | |
| C | S pGEM<br>2E4<br>FAM-NFQ-MGB<br>CT: 25.64 | S pGEM<br>2E4<br>FAM-NFQ-MGB<br>CT: 25.35 | 2203 A01 SCM saki3<br>U pGEM<br>1.74E4<br>FAM-NFQ-MGB<br>CT: 26.04 | 2203 A01 SCM saki3<br>U pGEM<br>1.91E4<br>FAM-NFQ-MGB<br>CT: 25.91 | 2203 A01 SCM saki3<br>U pGEM<br>1.81E4<br>FAM-NFQ-MGB<br>CT: 25.99 | 2203 A04 200 saki3<br>U pGEM<br>1.22E4<br>FAM-NFQ-MGB<br>CT: 26.57 | 2203 A04 200 saki3<br>U pGEM<br>1.11E4<br>FAM-NFQ-MGB<br>CT: 26.71 | 2203 A04 200 saki3<br>U pGEM<br>1.14E4<br>FAM-NFQ-MGB<br>CT: 26.67 | | | | |
| D | 1<br>S pGEM<br>2E3<br>FAM-NFQ-MGB<br>CT: 29.15 | 1<br>S pGEM<br>2E3<br>FAM-NFQ-MGB<br>CT: 29.87 | 2203 A01 SCM ato3<br>U pGEM<br>3.21E4<br>FAM-NFQ-MGB<br>CT: 25.14 | 2203 A01 SCM ato3<br>U pGEM<br>3.23E4<br>FAM-NFQ-MGB<br>CT: 25.13 | 2203 A01 SCM ato3<br>U pGEM<br>3.35E4<br>FAM-NFQ-MGB<br>CT: 25.07 | 2203 A04 200 ato3<br>U pGEM<br>3.23E4<br>FAM-NFQ-MGB<br>CT: 25.13 | 2203 A04 200 ato3<br>U pGEM<br>3.39E4<br>FAM-NFQ-MGB<br>CT: 25.06 | 2203 A04 200 ato3<br>U pGEM<br>3.13E4<br>FAM-NFQ-MGB<br>CT: 25.17 | | | | |
| E | S pGEM<br>200<br>FAM-NFQ-MGB<br>CT: 33.03 | S pGEM<br>200<br>FAM-NFQ-MGB<br>CT: 32.95 | 2203 A01 BTM saki3<br>U pGEM<br>1.98E4<br>FAM-NFQ-MGB<br>CT: 25.85 | 2203 A01 BTM saki3<br>U pGEM<br>2.05E4<br>FAM-NFQ-MGB<br>CT: 25.8 | 2203 A01 BTM saki3<br>U pGEM<br>1.98E4<br>FAM-NFQ-MGB<br>CT: 25.85 | 2203 A04 1000 saki3<br>U pGEM<br>1.86E4<br>FAM-NFQ-MGB<br>CT: 25.94 | 2203 A04 1000 saki3<br>U pGEM<br>2.12E4<br>FAM-NFQ-MGB<br>CT: 25.75 | 2203 A04 1000 saki3<br>U pGEM<br>2.02E4<br>FAM-NFQ-MGB<br>CT: 25.83 | | | | |
| F | S pGEM<br>20<br>FAM-NFQ-MGB<br>CT: 35.48 | S pGEM<br>20<br>FAM-NFQ-MGB<br>CT: 36 | 2203 A01 BTM ato3<br>U pGEM<br>3.08E4<br>FAM-NFQ-MGB<br>CT: 25.2 | 2203 A01 BTM ato3<br>U pGEM<br>2.65E4<br>FAM-NFQ-MGB<br>CT: 25.42 | 2203 A01 BTM ato3<br>U pGEM<br>3.11E4<br>FAM-NFQ-MGB<br>CT: 25.18 | 2203 A04 1000 ato3<br>U pGEM<br>3.02E4<br>FAM-NFQ-MGB<br>CT: 25.23 | 2203 A04 1000 ato3<br>U pGEM<br>3.35E4<br>FAM-NFQ-MGB<br>CT: 25.07 | 2203 A04 1000 ato3<br>U pGEM<br>3.27E4<br>FAM-NFQ-MGB<br>CT: 25.11 | | | | |
| G | | | 2203 A04 10 saki3<br>U pGEM<br>1.8E4<br>FAM-NFQ-MGB<br>CT: 25.99 | 2203 A04 10 saki3<br>U pGEM<br>1.76E4<br>FAM-NFQ-MGB<br>CT: 26.03 | 2203 A04 10 saki3<br>U pGEM<br>1.78E4<br>FAM-NFQ-MGB<br>CT: 26.01 | 2203 A04 3000 saki3<br>U pGEM<br>1.52E4<br>FAM-NFQ-MGB<br>CT: 26.24 | 2203 A04 3000 saki3<br>U pGEM<br>1.41E4<br>FAM-NFQ-MGB<br>CT: 26.36 | 2203 A04 3000 saki3<br>U pGEM<br>1.45E4<br>FAM-NFQ-MGB<br>CT: 26.31 | | | | N pGEM<br>FAM-NFQ-MGB<br>CT: Undetermined |
| H | Comf2E4<br>U pGEM<br>3.4E4<br>FAM-NFQ-MGB<br>CT: 25.05 | Comf2E4<br>U pGEM<br>3.69E4<br>FAM-NFQ-MGB<br>CT: 24.93 | 2203 A04 10 ato3<br>U pGEM<br>3.03E4<br>FAM-NFQ-MGB<br>CT: 25.22 | 2203 A04 10 ato3<br>U pGEM<br>3.14E4<br>FAM-NFQ-MGB<br>CT: 25.17 | 2203 A04 10 ato3<br>U pGEM<br>3.07E4<br>FAM-NFQ-MGB<br>CT: 25.2 | 2203 A04 3000 ato3<br>U pGEM<br>2.99E4<br>FAM-NFQ-MGB<br>CT: 25.24 | 2203 A04 3000 ato3<br>U pGEM<br>3.14E4<br>FAM-NFQ-MGB<br>CT: 25.17 | 2203 A04 3000 ato3<br>U pGEM<br>3.29E4<br>FAM-NFQ-MGB<br>CT: 25.1 | | | | N pGEM<br>FAM-NFQ-MGB<br>CT: Undetermined |

# Standard Curves

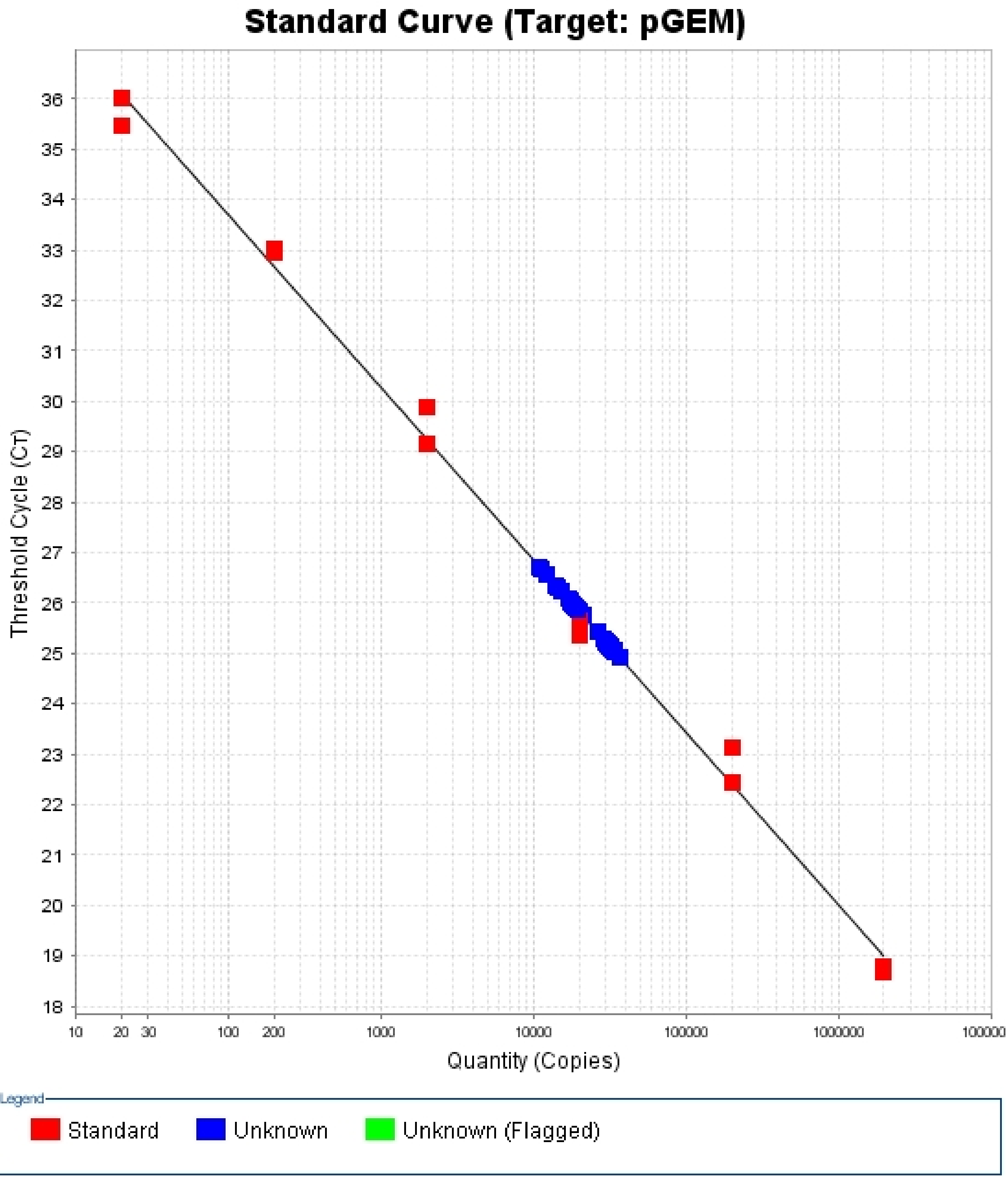


slope:-3.416 Y-Intercept:40.531 $R^2$:0.995 Eff%:96.203

# Results Table

| Well | Sample | Target | Task | Quantity | C |
|---|---|---|---|---|---|
| A1 | | pGEM | STANDARD | 2000000 | 18.805 |
| A2 | | pGEM | STANDARD | 2000000 | 18.6974 |
| A3 | 2203 A01 10 | pGEM | UNKNOWN | 19107.8555 | 25.9051 |
| A4 | 2203 A01 10 | pGEM | UNKNOWN | 19718.5781 | 25.8584 |
| A5 | 2203 A01 10 | pGEM | UNKNOWN | 19233.2441 | 25.8954 |
| A6 | 2203 A04 SCM | pGEM | UNKNOWN | 17085.7656 | 26.071 |
| A7 | 2203 A04 SCM | pGEM | UNKNOWN | 18727.8809 | 25.9349 |
| A8 | 2203 A04 SCM | pGEM | UNKNOWN | 16961.7695 | 26.0818 |
| B1 | | pGEM | STANDARD | 200000 | 23.1407 |
| B2 | | pGEM | STANDARD | 200000 | 22.4288 |
| B3 | 2203 A01 10 | pGEM | UNKNOWN | 31726.1094 | 25.1528 |
| B4 | 2203 A01 10 | pGEM | UNKNOWN | 31271.4727 | 25.1742 |
| B5 | 2203 A01 10 | pGEM | UNKNOWN | 29060.7051 | 25.283 |
| B6 | 2203 A04 SCM | pGEM | UNKNOWN | 32701.3945 | 25.1078 |
| B7 | 2203 A04 SCM | pGEM | UNKNOWN | 31829.0117 | 25.148 |
| B8 | 2203 A04 SCM | pGEM | UNKNOWN | 34160.2773 | 25.0431 |
| C1 | | pGEM | STANDARD | 20000 | 25.6395 |
| C2 | | pGEM | STANDARD | 20000 | 25.3515 |
| C3 | 2203 A01 SCM | pGEM | UNKNOWN | 17413.3398 | 26.0429 |
| C4 | 2203 A01 SCM | pGEM | UNKNOWN | 19106.1855 | 25.9052 |
| C5 | 2203 A01 SCM | pGEM | UNKNOWN | 18082.9863 | 25.9869 |
| C6 | 2203 A04 200 | pGEM | UNKNOWN | 12237.6631 | 26.5662 |
| C7 | 2203 A04 200 | pGEM | UNKNOWN | 11130.3496 | 26.7069 |
| C8 | 2203 A04 200 | pGEM | UNKNOWN | 11423.2939 | 26.6684 |
| D1 | | pGEM | STANDARD | 2000 | 29.1538 |
| D2 | | pGEM | STANDARD | 2000 | 29.8707 |
| D3 | 2203 A01 SCM | pGEM | UNKNOWN | 32101.2012 | 25.1353 |
| D4 | 2203 A01 SCM | pGEM | UNKNOWN | 32280.9238 | 25.127 |
| D5 | 2203 A01 SCM | pGEM | UNKNOWN | 33543.0977 | 25.0701 |
| D6 | 2203 A04 200 | pGEM | UNKNOWN | 32316.8809 | 25.1254 |

| Well | Sample | Target | Task | Quantity | C |
|---|---|---|---|---|---|
| D7 | 2203 A04 200 | pGEM | UNKNOWN | 33872.1562 | 25.0556 |
| D8 | 2203 A04 200 | pGEM | UNKNOWN | 31305.8223 | 25.1725 |
| E1 | | pGEM | STANDARD | 200 | 33.0339 |
| E2 | | pGEM | STANDARD | 200 | 32.9478 |
| E3 | 2203 A01 BTM | pGEM | UNKNOWN | 19827.5977 | 25.8502 |
| E4 | 2203 A01 BTM | pGEM | UNKNOWN | 20543.1855 | 25.7976 |
| E5 | 2203 A01 BTM | pGEM | UNKNOWN | 19818.6797 | 25.8509 |
| E6 | 2203 A04 1000 | pGEM | UNKNOWN | 18608.1562 | 25.9444 |
| E7 | 2203 A04 1000 | pGEM | UNKNOWN | 21173.6934 | 25.7527 |
| E8 | 2203 A04 1000 | pGEM | UNKNOWN | 20156.5 | 25.8258 |
| F1 | | pGEM | STANDARD | 20 | 35.4826 |
| F2 | | pGEM | STANDARD | 20 | 35.9956 |
| F3 | 2203 A01 BTM | pGEM | UNKNOWN | 30813.2852 | 25.1961 |
| F4 | 2203 A01 BTM | pGEM | UNKNOWN | 26483.5879 | 25.4207 |
| F5 | 2203 A01 BTM | pGEM | UNKNOWN | 31148.1816 | 25.18 |
| F6 | 2203 A04 1000 | pGEM | UNKNOWN | 30203.1406 | 25.2257 |
| F7 | 2203 A04 1000 | pGEM | UNKNOWN | 33478.4805 | 25.073 |
| F8 | 2203 A04 1000 | pGEM | UNKNOWN | 32658.334 | 25.1098 |
| G3 | 2203 A04 10 | pGEM | UNKNOWN | 18031.7324 | 25.9911 |
| G4 | 2203 A04 10 | pGEM | UNKNOWN | 17607.1465 | 26.0264 |
| G5 | 2203 A04 10 | pGEM | UNKNOWN | 17764.4746 | 26.0132 |
| G6 | 2203 A04 3000 | pGEM | UNKNOWN | 15198.6992 | 26.2447 |
| G7 | 2203 A04 3000 | pGEM | UNKNOWN | 14075.2373 | 26.3586 |
| G8 | 2203 A04 3000 | pGEM | UNKNOWN | 14511.4648 | 26.3133 |
| G12 | | pGEM | NTC | | Undetermined |
| H1 | Comf2E4 | pGEM | UNKNOWN | 33978.3047 | 25.051 |
| H2 | Comf2E4 | pGEM | UNKNOWN | 36890.9258 | 24.929 |
| H3 | 2203 A04 10 | pGEM | UNKNOWN | 30285.1758 | 25.2217 |
| H4 | 2203 A04 10 | pGEM | UNKNOWN | 31394.7656 | 25.1683 |
| H5 | 2203 A04 10 | pGEM | UNKNOWN | 30732.9805 | 25.1999 |
| H6 | 2203 A04 3000 | pGEM | UNKNOWN | 29945.6211 | 25.2385 |
| H7 | 2203 A04 3000 | pGEM | UNKNOWN | 31356.0469 | 25.1702 |

| Well | | Sample | Target | Task | Quantity | C |
|---|---|---|---|---|---|---|
| H8 | | 2203 A04 3000 | pGEM | UNKNOWN | 32885.4492 | 25.0995 |
| H12 | | | pGEM | NTC | | Undetermined |

# QC Summary

| | | | | | |
|---|---|---|---|---|---|
| Total Wells | 96 | Processed Wells | 64 | Targets Used | 1 |
| Well Setup | 64 | Flagged Wells | 4 | Samples Used | 17 |

| Flag | Name | Frequency | Locations |
|---|---|---|---|
| AMPNC | Amplification in negative control | 0 | |
| BADROX | Bad passive reference signal | 0 | |
| BLFAIL | Baseline algorithm failed | 0 | |
| CTFAIL | C algorithm failed | 0 | |
| EXPFAIL | Exponential algorithm failed | 0 | |
| HIGHSD | High standard deviation in replicate group | 4 | B1, B2, D1, D2 |
| NOAMP | No amplification | 0 | |
| NOISE | Noise higher than others in plate | 0 | |
| NOSIGNAL | No signal in well | 0 | |
| OFFSCALE | Fluorescence is offscale | 0 | |
| OUTLIERRG | Outlier in replicate group | 0 | |
| SPIKE | Noise spikes | 0 | |
| THOLDFAIL | Thresholding algorithm failed | 0 | |
