# Supplementary material for: Ecological impact of algal-preying protists aplanochytrids (Labyrinthulea) as a source of docosahexaenoic acid for copepods in marine environments
Source: ISME Commun. 2026 Jun 26;6(1):ycag178. doi: 10.1093/ismeco/ycag178 (PMC13685785; doi:10.1093/ismeco/ycag178)
Supplement: Supplementary file 2 [file supplementary_materials_ycag178.zip › Morimoto_ISME_C_3rdrev_supplemental_data.zip!/Morimoto_ISME_C_3rdrev_supplemental_data/Supplemental_Data_2/Standard Curve/DNA recovery/2203/DnaRec2203AlineA07-A21_N=2.pdf]

Experiment Results Report
230523DnaRec2203AlineA07-A21

Experiment Summary

Experiment Name	:	230523DnaRec2203AlineA07-A21
Experiment Type	:	Quantitation - Standard Curve
File Name	:	230523DnaRec2203AlineA07-A21.eds
Run Started	:	2023 May 23 6:05:54 PM
Run Finished	:	2023 May 23 7:40:32 PM
Run Duration	:	94 minutes 37 seconds
Date Modified	:	2023 May 24 11:38:12 AM
User	:	
Number of wells used	:	94
Number of wells with results	:	93
Instrument Name	:	
Instrument Type	:	Applied Biosystems StepOnePlus™ Instrument
Comments	:	

Results Summary

Sample	Target	Quantity (Mean)	Quantity (Std Dev)	C (Mean)	C (Std Dev)
2203 A07 10 ato	pGEM	25099.32	490.79	25.42	0.03
2203 A07 10 saki	pGEM	18179.79	690.14	25.90	0.06
2203 A07 1000 ato	pGEM	26619.14	1337.76	24.79	0.04
2203 A07 1000 saki	pGEM	20059.42	1028.43	25.75	0.08
2203 A07 200 ato	pGEM	24829.79	1469.16	25.44	0.09
2203 A07 200 saki	pGEM	18956.77	831.21	25.84	0.07
2203 A07 3000 ato	pGEM	24548.09	1744.45	25.45	0.10
2203 A07 3000 saki	pGEM	6279.91	663.83	27.49	0.16
2203 A07 SCM ato	pGEM	27103.26	445.49	25.42	0.04
2203 A07 SCM saki	pGEM	18632.62	1354.70	25.87	0.11
2203 A11 1000 ato	pGEM	26766.05	1316.43	25.32	0.07
2203 A11 1000 saki	pGEM	17907.46	531.79	25.92	0.04
2203 A11 200 ato	pGEM	25768.03	1812.45	25.38	0.10
2203 A11 200 saki	pGEM	17736.72	1520.28	25.94	0.13
2203 A11 3000 ato	pGEM	25436.66	628.83	25.40	0.04
2203 A11 3000 saki	pGEM			25.95	0.15
2203 A11 SCM ato	pGEM	25540.17	2139.71	25.40	0.13
2203 A11 SCM saki	pGEM	17879.50	364.78	25.92	0.03
2203 A21 1000 ato	pGEM	26071.31	1259.25	25.36	0.07
2203 A21 1000 saki	pGEM	10277.70	363.36	26.75	0.05
2203 A21 200 ato	pGEM	26141.68	1795.81	25.36	0.10
2203 A21 200 saki	pGEM	16188.78	698.02	26.49	0.09
2203 A21 3000 ato	pGEM	24900.97	460.43	25.43	0.03
2203 A21 3000 saki	pGEM	7774.67	331.34	26.75	0.05
2203 A21 SCM ato	pGEM	24789.30	1329.25	25.44	0.08
2203 A21 SCM saki	pGEM	11195.77	279.53	26.62	0.04
Comf2E4	pGEM	21912.73	421.87	25.62	0.03

Plate Layout

	1	2	3	4	5	6
A	S pGEM<br>2E6<br>FAM-NFQ-MGB<br>CT: 19.1	S pGEM<br>2E6<br>FAM-NFQ-MGB<br>CT: 19.06	2203 A07 10 saki<br>U pGEM<br>1.89E4<br>FAM-NFQ-MGB<br>CT: 25.84	2203 A07 10 saki<br>U pGEM<br>1.82E4<br>FAM-NFQ-MGB<br>CT: 25.9	2203 A07 10 saki<br>U pGEM<br>1.75E4<br>FAM-NFQ-MGB<br>CT: 25.96	2203 A07 3000 saki<br>U pGEM<br>5.63E3<br>FAM-NFQ-MGB<br>CT: 27.64
B	S pGEM<br>2E5<br>FAM-NFQ-MGB<br>CT: 22.15	S pGEM<br>2E5<br>FAM-NFQ-MGB<br>CT: 22.06	2203 A07 10 ato<br>U pGEM<br>2.56E4<br>FAM-NFQ-MGB<br>CT: 25.39	2203 A07 10 ato<br>U pGEM<br>2.46E4<br>FAM-NFQ-MGB<br>CT: 25.45	2203 A07 10 ato<br>U pGEM<br>2.51E4<br>FAM-NFQ-MGB<br>CT: 25.42	2203 A07 3000 saki<br>U pGEM<br>6.96E3<br>FAM-NFQ-MGB<br>CT: 27.33
C	S pGEM<br>2E4<br>FAM-NFQ-MGB<br>CT: 25.8	S pGEM<br>2E4<br>FAM-NFQ-MGB<br>CT: 25.57	2203 A07 SCM saki<br>U pGEM<br>1.87E4<br>FAM-NFQ-MGB<br>CT: 25.85	2203 A07 SCM saki<br>U pGEM<br>1.99E4<br>FAM-NFQ-MGB<br>CT: 25.76	2203 A07 SCM saki<br>U pGEM<br>1.72E4<br>FAM-NFQ-MGB<br>CT: 25.98	2203 A07 3000 saki<br>U pGEM<br>6.24E3<br>FAM-NFQ-MGB<br>CT: 27.49
D	S pGEM<br>2E3<br>FAM-NFQ-MGB<br>CT: 29.33	S pGEM<br>2E3<br>FAM-NFQ-MGB<br>CT: 29.17	2203 A07 SCM ato<br>U pGEM<br>2.75E4<br>FAM-NFQ-MGB<br>CT: 25.44	2203 A07 SCM ato<br>U pGEM<br>2.72E4<br>FAM-NFQ-MGB<br>CT: 25.37	2203 A07 SCM ato<br>U pGEM<br>2.44E4<br>FAM-NFQ-MGB<br>CT: 25.46	2203 A07 3000 ato<br>U pGEM<br>2.37E4<br>FAM-NFQ-MGB<br>CT: 25.5
E	S pGEM<br>200<br>FAM-NFQ-MGB<br>CT: 32.37	S pGEM<br>200<br>FAM-NFQ-MGB<br>CT: 33.07	2203 A07 200 saki<br>U pGEM<br>1.92E4<br>FAM-NFQ-MGB<br>CT: 25.81	2203 A07 200 saki<br>U pGEM<br>1.96E4<br>FAM-NFQ-MGB<br>CT: 25.79	2203 A07 200 saki<br>U pGEM<br>1.8E4<br>FAM-NFQ-MGB<br>CT: 25.91	2203 A07 3000 ato<br>U pGEM<br>2.34E4<br>FAM-NFQ-MGB<br>CT: 25.53
F	S pGEM<br>20<br>FAM-NFQ-MGB<br>CT: 35.99	S pGEM<br>20<br>FAM-NFQ-MGB<br>CT: 35.94	2203 A07 200 ato<br>U pGEM<br>2.57E4<br>FAM-NFQ-MGB<br>CT: 25.38	2203 A07 200 ato<br>U pGEM<br>2.31E4<br>FAM-NFQ-MGB<br>CT: 25.54	2203 A07 200 ato<br>U pGEM<br>2.56E4<br>FAM-NFQ-MGB<br>CT: 25.39	2203 A07 3000 ato<br>U pGEM<br>2.66E4<br>FAM-NFQ-MGB<br>CT: 25.34
G	Comf2E4<br>U pGEM<br>2.16E4<br>FAM-NFQ-MGB<br>CT: 25.64	Comf2E4<br>U pGEM<br>2.22E4<br>FAM-NFQ-MGB<br>CT: 25.6	2203 A07 1000 saki<br>U pGEM<br>2.12E4<br>FAM-NFQ-MGB<br>CT: 25.67	2203 A07 1000 saki<br>U pGEM<br>1.93E4<br>FAM-NFQ-MGB<br>CT: 25.81	2203 A07 1000 saki<br>U pGEM<br>2.08E4<br>FAM-NFQ-MGB<br>CT: 25.78	
H	N pGEM<br>FAM-NFQ-MGB<br>CT: Undetermined	N pGEM<br>FAM-NFQ-MGB<br>CT: Undetermined	2203 A07 1000 ato<br>U pGEM<br>2.51E4<br>FAM-NFQ-MGB<br>CT: 24.88	2203 A07 1000 ato<br>U pGEM<br>2.78E4<br>FAM-NFQ-MGB<br>CT: 25.26	2203 A07 1000 ato<br>U pGEM<br>FAM-NFQ-MGB<br>CT: Undetermined	

	7	8	9	10	11	12
A	2203 A11 SCM saki<br>U pGEM<br>1.77E4<br>FAM-NFQ-MGB<br>CT: 25.94	2203 A11 SCM saki<br>U pGEM<br>1.77E4<br>FAM-NFQ-MGB<br>CT: 25.94	2203 A11 SCM saki<br>U pGEM<br>1.83E4<br>FAM-NFQ-MGB<br>CT: 25.89	2203 A21 SCM saki<br>U pGEM<br>1.09E4<br>FAM-NFQ-MGB<br>CT: 26.66	2203 A21 SCM saki<br>U pGEM<br>1.15E4<br>FAM-NFQ-MGB<br>CT: 26.58	2203 A21 SCM saki<br>U pGEM<br>1.12E4<br>FAM-NFQ-MGB<br>CT: 26.62
B	2203 A11 SCM ato<br>U pGEM<br>2.32E4<br>FAM-NFQ-MGB<br>CT: 25.54	2203 A11 SCM ato<br>U pGEM<br>2.73E4<br>FAM-NFQ-MGB<br>CT: 25.3	2203 A11 SCM ato<br>U pGEM<br>2.62E4<br>FAM-NFQ-MGB<br>CT: 25.36	2203 A21 SCM ato<br>U pGEM<br>2.57E4<br>FAM-NFQ-MGB<br>CT: 25.38	2203 A21 SCM ato<br>U pGEM<br>2.33E4<br>FAM-NFQ-MGB<br>CT: 25.53	2203 A21 SCM ato<br>U pGEM<br>2.54E4<br>FAM-NFQ-MGB<br>CT: 25.4
C	2203 A11 200 saki<br>U pGEM<br>1.91E4<br>FAM-NFQ-MGB<br>CT: 25.82	2203 A11 200 saki<br>U pGEM<br>1.8E4<br>FAM-NFQ-MGB<br>CT: 25.91	2203 A11 200 saki<br>U pGEM<br>1.68E4<br>FAM-NFQ-MGB<br>CT: 26.08	2203 A21 200 saki<br>U pGEM<br>1.55E4<br>FAM-NFQ-MGB<br>CT: 26.57	2203 A21 200 saki<br>U pGEM<br>1.3E4<br>FAM-NFQ-MGB<br>CT: 26.39	2203 A21 200 saki<br>U pGEM<br>1.22E4<br>FAM-NFQ-MGB<br>CT: 26.5
D	2203 A11 200 ato<br>U pGEM<br>2.79E4<br>FAM-NFQ-MGB<br>CT: 25.26	2203 A11 200 ato<br>U pGEM<br>2.48E4<br>FAM-NFQ-MGB<br>CT: 25.44	2203 A11 200 ato<br>U pGEM<br>2.46E4<br>FAM-NFQ-MGB<br>CT: 25.45	2203 A21 200 ato<br>U pGEM<br>2.68E4<br>FAM-NFQ-MGB<br>CT: 25.32	2203 A21 200 ato<br>U pGEM<br>2.75E4<br>FAM-NFQ-MGB<br>CT: 25.28	2203 A21 200 ato<br>U pGEM<br>2.41E4<br>FAM-NFQ-MGB<br>CT: 25.48
E	2203 A11 1000 saki<br>U pGEM<br>1.84E4<br>FAM-NFQ-MGB<br>CT: 25.88	2203 A11 1000 saki<br>U pGEM<br>1.73E4<br>FAM-NFQ-MGB<br>CT: 25.97	2203 A11 1000 saki<br>U pGEM<br>1.8E4<br>FAM-NFQ-MGB<br>CT: 25.91	2203 A21 1000 saki<br>U pGEM<br>9.88E3<br>FAM-NFQ-MGB<br>CT: 26.81	2203 A21 1000 saki<br>U pGEM<br>1.06E4<br>FAM-NFQ-MGB<br>CT: 26.7	2203 A21 1000 saki<br>U pGEM<br>1.04E4<br>FAM-NFQ-MGB<br>CT: 26.74
F	2203 A11 1000 ato<br>U pGEM<br>2.6E4<br>FAM-NFQ-MGB<br>CT: 25.37	2203 A11 1000 ato<br>U pGEM<br>2.83E4<br>FAM-NFQ-MGB<br>CT: 25.24	2203 A11 1000 ato<br>U pGEM<br>FAM-NFQ-MGB<br>CT: 25.37	2203 A21 1000 ato<br>U pGEM<br>2.66E4<br>FAM-NFQ-MGB<br>CT: 25.33	2203 A21 1000 ato<br>U pGEM<br>2.7E4<br>FAM-NFQ-MGB<br>CT: 25.31	2203 A21 1000 ato<br>U pGEM<br>2.46E4<br>FAM-NFQ-MGB<br>CT: 25.45
G	2203 A11 3000 saki<br>U pGEM<br>FAM-NFQ-MGB<br>CT: 26.12	2203 A11 3000 saki<br>U pGEM<br>1.84E4<br>FAM-NFQ-MGB<br>CT: 25.88	2203 A11 3000 saki<br>U pGEM<br>7.95E3<br>FAM-NFQ-MGB<br>CT: 25.84	2203 A21 3000 saki<br>U pGEM<br>7.39E3<br>FAM-NFQ-MGB<br>CT: 26.76	2203 A21 3000 saki<br>U pGEM<br>7.98E3<br>FAM-NFQ-MGB<br>CT: 26.7	2203 A21 3000 saki<br>U pGEM<br>2.04E4<br>FAM-NFQ-MGB<br>CT: 26.8
H	2203 A11 3000 ato<br>U pGEM<br>2.59E4<br>FAM-NFQ-MGB<br>CT: 25.37	2203 A11 3000 ato<br>U pGEM<br>2.56E4<br>FAM-NFQ-MGB<br>CT: 25.39	2203 A11 3000 ato<br>U pGEM<br>2.47E4<br>FAM-NFQ-MGB<br>CT: 25.44	2203 A21 3000 ato<br>U pGEM<br>2.53E4<br>FAM-NFQ-MGB<br>CT: 25.41	2203 A21 3000 ato<br>U pGEM<br>2.44E4<br>FAM-NFQ-MGB<br>CT: 25.46	2203 A21 3000 ato<br>U pGEM<br>2.5E4<br>FAM-NFQ-MGB<br>CT: 25.43

Standard Curves

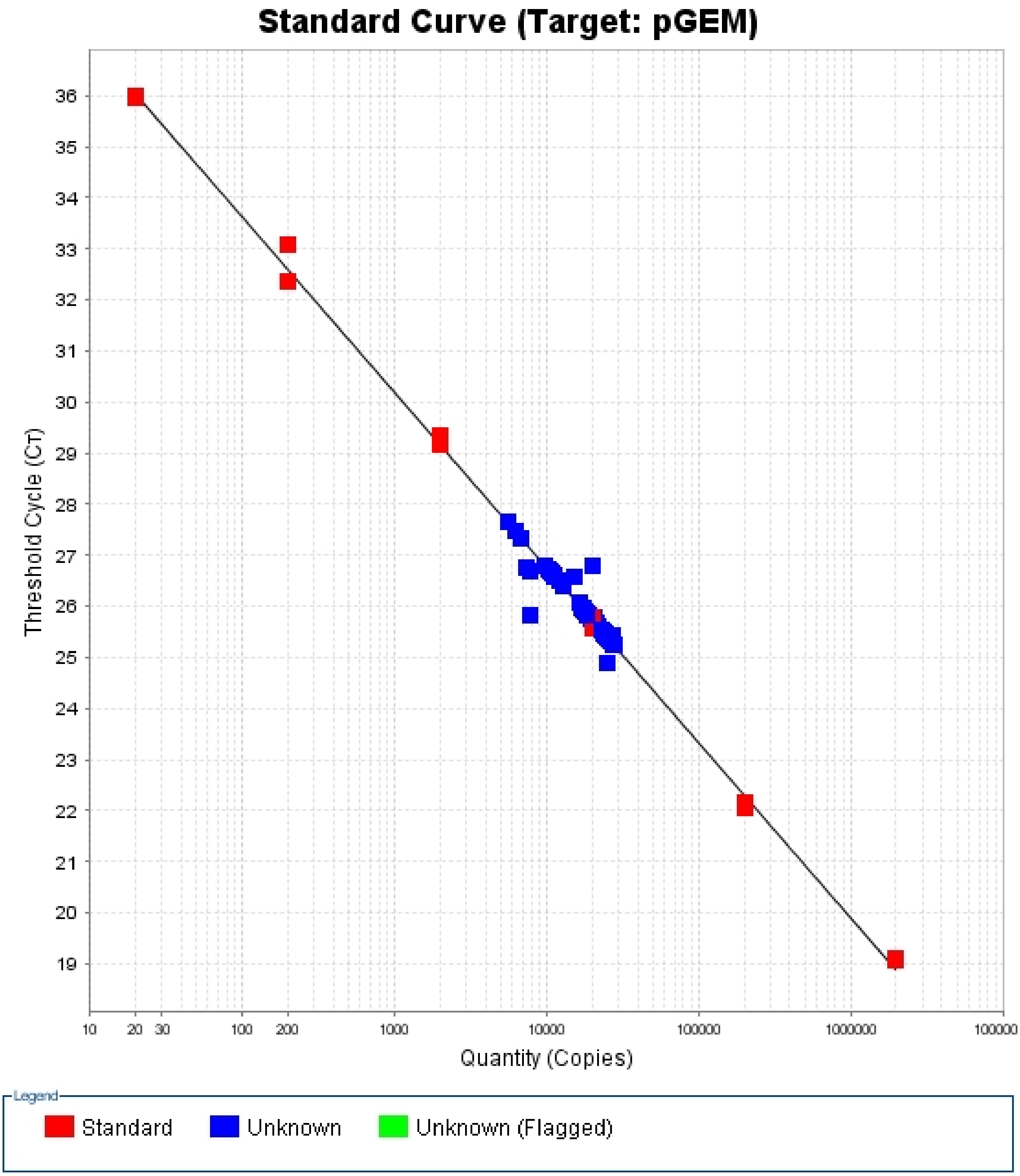

slope:-3.424 Y-Intercept:40.484 R^2:0.999 Eff%:95.91

Results Table

Well		Sample	Target	Task	Quantity	C
A1			pGEM	STANDARD	2000000	19.1031
A2			pGEM	STANDARD	2000000	19.0591
A3		2203 A07 10	pGEM	UNKNOWN	18882.5859	25.8426
A4		2203 A07 10	pGEM	UNKNOWN	18153.7617	25.9011
A5		2203 A07 10	pGEM	UNKNOWN	17503.0391	25.9554
A6		2203 A07 3000	pGEM	UNKNOWN	5634.8979	27.6408
A7		2203 A11 SCM	pGEM	UNKNOWN	17674.791	25.9409
A8		2203 A11 SCM	pGEM	UNKNOWN	17663.0527	25.9418
A9		2203 A11 SCM	pGEM	UNKNOWN	18300.6543	25.8891
A10		2203 A21 SCM	pGEM	UNKNOWN	10912.6807	26.6579
A11		2203 A21 SCM	pGEM	UNKNOWN	11471.5908	26.5836
A12		2203 A21 SCM	pGEM	UNKNOWN	11203.042	26.6189
B1			pGEM	STANDARD	200000	22.1456
B2			pGEM	STANDARD	200000	22.0638
B3		2203 A07 10 ato	pGEM	UNKNOWN	25564.8945	25.392
B4		2203 A07 10 ato	pGEM	UNKNOWN	24586.6934	25.45
B5		2203 A07 10 ato	pGEM	UNKNOWN	25146.3691	25.4166
B6		2203 A07 3000	pGEM	UNKNOWN	6961.0713	27.3265
B7		2203 A11 SCM	pGEM	UNKNOWN	23152.1953	25.5394
B8		2203 A11 SCM	pGEM	UNKNOWN	27283.2812	25.2953
B9		2203 A11 SCM	pGEM	UNKNOWN	26185.0312	25.3564
B10		2203 A21 SCM	pGEM	UNKNOWN	25742.1191	25.3818
B11		2203 A21 SCM	pGEM	UNKNOWN	23270.7793	25.5318
B12		2203 A21 SCM	pGEM	UNKNOWN	25355.0137	25.4043
C1			pGEM	STANDARD	20000	25.7973
C2			pGEM	STANDARD	20000	25.572
C3		2203 A07 SCM	pGEM	UNKNOWN	18748.7617	25.8531
C4		2203 A07 SCM	pGEM	UNKNOWN	19925.5	25.7626
C5		2203 A07 SCM	pGEM	UNKNOWN	17223.5859	25.9793
C6		2203 A07 3000	pGEM	UNKNOWN	6243.7466	27.4882

Well		Sample	Target	Task	Quantity	C
C7		2203 A11 200	pGEM	UNKNOWN	19114.2793	25.8244
C8		2203 A11 200	pGEM	UNKNOWN	17990.2715	25.9146
C9		2203 A11 200	pGEM	UNKNOWN	16843.8516	26.0791
C10		2203 A21 200	pGEM	UNKNOWN	15454.5596	26.5664
C11		2203 A21 200	pGEM	UNKNOWN	13033.3467	26.3938
C12		2203 A21 200	pGEM	UNKNOWN	12170.9785	26.4956
D1			pGEM	STANDARD	2000	29.3349
D2			pGEM	STANDARD	2000	29.1745
D3		2203 A07 SCM	pGEM	UNKNOWN	27473.0605	25.4411
D4		2203 A07 SCM	pGEM	UNKNOWN	27228.0254	25.3743
D5		2203 A07 SCM	pGEM	UNKNOWN	24447.6973	25.4585
D6		2203 A07 3000	pGEM	UNKNOWN	23723.9082	25.5032
D7		2203 A11 200	pGEM	UNKNOWN	27857.4219	25.2643
D8		2203 A11 200	pGEM	UNKNOWN	24827.3203	25.4356
D9		2203 A11 200	pGEM	UNKNOWN	24619.3555	25.4481
D10		2203 A21 200	pGEM	UNKNOWN	26848.0566	25.3192
D11		2203 A21 200	pGEM	UNKNOWN	27476.8945	25.2848
D12		2203 A21 200	pGEM	UNKNOWN	24100.0801	25.4798
E1			pGEM	STANDARD	200	32.3742
E2			pGEM	STANDARD	200	33.0726
E3		2203 A07 200	pGEM	UNKNOWN	19240.6387	25.8146
E4		2203 A07 200	pGEM	UNKNOWN	19608.8535	25.7864
E5		2203 A07 200	pGEM	UNKNOWN	18020.8047	25.912
E6		2203 A07 3000	pGEM	UNKNOWN	23368.4395	25.5256
E7		2203 A11 1000	pGEM	UNKNOWN	18366.7324	25.8838
E8		2203 A11 1000	pGEM	UNKNOWN	17324.8418	25.9706
E9		2203 A11 1000	pGEM	UNKNOWN	18030.8145	25.9112
E10		2203 A21 1000	pGEM	UNKNOWN	9879.9268	26.8058
E11		2203 A21 1000	pGEM	UNKNOWN	10592.1846	26.7022
E12		2203 A21 1000	pGEM	UNKNOWN	10360.9941	26.7351
F1			pGEM	STANDARD	20	35.9875
F2			pGEM	STANDARD	20	35.9413

Well		Sample	Target	Task	Quantity	C
F3		2203 A07 200	pGEM	UNKNOWN	25708.9238	25.3837
F4		2203 A07 200	pGEM	UNKNOWN	23133.7324	25.5406
F5		2203 A07 200	pGEM	UNKNOWN	25646.709	25.3873
F6		2203 A07 3000	pGEM	UNKNOWN	26551.9238	25.3357
F7		2203 A11 1000	pGEM	UNKNOWN	26029.9199	25.3652
F8		2203 A11 1000	pGEM	UNKNOWN	28285.8906	25.2416
F9		2203 A11 1000	pGEM	UNKNOWN		25.3679
F10		2203 A21 1000	pGEM	UNKNOWN	26618.3848	25.332
F11		2203 A21 1000	pGEM	UNKNOWN	26964.498	25.3128
F12		2203 A21 1000	pGEM	UNKNOWN	24631.043	25.4474
G1		Comf2E4	pGEM	UNKNOWN	21614.4219	25.6416
G2		Comf2E4	pGEM	UNKNOWN	22211.0332	25.6011
G3		2203 A07 1000	pGEM	UNKNOWN	21229.8594	25.6683
G4		2203 A07 1000	pGEM	UNKNOWN	19300.332	25.81
G5		2203 A07 1000	pGEM	UNKNOWN	20820.9727	25.7835
G7		2203 A11 3000	pGEM	UNKNOWN		26.1215
G8		2203 A11 3000	pGEM	UNKNOWN	18431.3262	25.8785
G9		2203 A11 3000	pGEM	UNKNOWN	7948.3687	25.8429
G10		2203 A21 3000	pGEM	UNKNOWN	7392.5933	26.7612
G11		2203 A21 3000	pGEM	UNKNOWN	7983.0479	26.6983
G12		2203 A21 3000	pGEM	UNKNOWN	20378.207	26.7975
H1			pGEM	NTC		Undetermined
H2			pGEM	NTC		Undetermined
H3		2203 A07 1000	pGEM	UNKNOWN	25075.0762	24.8761
H4		2203 A07 1000	pGEM	UNKNOWN	27845.5977	25.265
H5		2203 A07 1000	pGEM	UNKNOWN		
H7		2203 A11 3000	pGEM	UNKNOWN	25945.5859	25.37
H8		2203 A11 3000	pGEM	UNKNOWN	25630.7266	25.3882
H9		2203 A11 3000	pGEM	UNKNOWN	24733.6816	25.4412
H10		2203 A21 3000	pGEM	UNKNOWN	25321.6016	25.4062
H11		2203 A21 3000	pGEM	UNKNOWN	24409.0645	25.4608
H12		2203 A21 3000	pGEM	UNKNOWN	24972.2539	25.4269

QC Summary

Total Wells	96	Processed Wells	94	Targets Used	1
Well Setup	94	Flagged Wells	0	Samples Used	27

Flag	Name	Frequency	Locations
AMPNC	Amplification in negative control	0	
BADROX	Bad passive reference signal	0	
BLFAIL	Baseline algorithm failed	0	
CTFAIL	C algorithm failed	0	
EXPFAIL	Exponential algorithm failed	0	
HIGHSD	High standard deviation in replicate group	0	
NOAMP	No amplification	0	
NOISE	Noise higher than others in plate	0	
NOSIGNAL	No signal in well	0	
OFFSCALE	Fluorescence is offscale	0	
OUTLIERRG	Outlier in replicate group	0	
SPIKE	Noise spikes	0	
THOLDFAIL	Thresholding algorithm failed	0	
