# Supplementary material for: Ecological impact of algal-preying protists aplanochytrids (Labyrinthulea) as a source of docosahexaenoic acid for copepods in marine environments
Source: ISME Commun. 2026 Jun 26;6(1):ycag178. doi: 10.1093/ismeco/ycag178 (PMC13685785; doi:10.1093/ismeco/ycag178)
Supplement: Supplementary file 2 [file supplementary_materials_ycag178.zip › Morimoto_ISME_C_3rdrev_supplemental_data.zip!/Morimoto_ISME_C_3rdrev_supplemental_data/Supplemental_Data_2/Standard Curve/DNA recovery/2203/DnaRec2203AlineA01-A04_N=2.pdf]

Experiment Results Report

230523DnaRec2203AlineN=1A01-04

Experiment Summary

Experiment Name	:	230523DnaRec2203AlineN=1A01-04
Experiment Type	:	Quantitation - Standard Curve
File Name	:	230523DnaRec2203AlineN=1A01-04.eds
Run Started	:	2023 May 23 3:37:06 PM
Run Finished	:	2023 May 23 5:11:45 PM
Run Duration	:	94 minutes 39 seconds
Date Modified	:	2023 May 23 5:17:31 PM
User	:	
Number of wells used	:	64
Number of wells with results	:	64
Instrument Name	:	
Instrument Type	:	Applied Biosystems StepOnePlus™ Instrument
Comments	:	

Results Summary

Sample	Target	Quantity (Mean)	Quantity (Std Dev)	C (Mean)	C (Std Dev)
2203 A01 10 ato	pGEM	27103.26	445.49	24.76	0.03
2203 A01 10 saki	pGEM	15109.01	659.19	25.65	0.07
2203 A01 BTM ato	pGEM	25968.69	1783.60	24.83	0.10
2203 A01 BTM saki	pGEM	20821.49	557.17	25.16	0.04
2203 A01 SCM ato	pGEM	27027.44	236.15	24.76	0.01
2203 A01 SCM saki	pGEM	22128.45	1289.60	25.07	0.09
2203 A04 10 ato	pGEM	26619.14	1337.76	24.79	0.04
2203 A04 10 saki	pGEM	20153.93	595.89	25.21	0.04
2203 A04 1000 ato	pGEM	27980.57	1082.18	24.71	0.06
2203 A04 1000 saki	pGEM	20774.56	877.21	25.16	0.06
2203 A04 200 ato	pGEM	26503.28	2297.20	24.80	0.13
2203 A04 200 saki	pGEM	20840.44	534.73	25.16	0.04
2203 A04 3000 ato	pGEM	29684.80	584.40	24.62	0.03
2203 A04 3000 saki	pGEM	7774.67	331.34	26.66	0.07
2203 A04 SCM ato	pGEM	26927.71	1001.91	24.77	0.06
2203 A04 SCM saki	pGEM	16188.78	698.02	25.54	0.07
Comf2E4	pGEM	21869.74	2109.35	25.09	0.15

Plate Layout

	1	2	3	4	5	6	7	8	9	10	11	12
A	S pGEM<br>2E6<br>FAM-NFQ-MGB<br>CT: 18.49	S pGEM<br>2E6<br>FAM-NFQ-MGB<br>CT: 18.35	2203 A01 10 saki<br>U pGEM<br>1.58E4<br>FAM-NFQ-MGB<br>CT: 25.58	2203 A01 10 saki<br>U pGEM<br>1.45E4<br>FAM-NFQ-MGB<br>CT: 25.71	2203 A01 10 saki<br>U pGEM<br>1.51E4<br>FAM-NFQ-MGB<br>CT: 25.65	2203 A04 SCM saki<br>U pGEM<br>1.68E4<br>FAM-NFQ-MGB<br>CT: 25.48	2203 A04 SCM saki<br>U pGEM<br>1.55E4<br>FAM-NFQ-MGB<br>CT: 25.61	2203 A04 SCM saki<br>U pGEM<br>1.22E4<br>FAM-NFQ-MGB<br>CT: 25.53				
B	S pGEM<br>2E5<br>FAM-NFQ-MGB<br>CT: 21.58	S pGEM<br>2E5<br>FAM-NFQ-MGB<br>CT: 29.17	2203 A01 10 ato<br>U pGEM<br>2.66E4<br>FAM-NFQ-MGB<br>CT: 24.79	2203 A01 10 ato<br>U pGEM<br>2.75E4<br>FAM-NFQ-MGB<br>CT: 24.74	2203 A01 10 ato<br>U pGEM<br>2.72E4<br>FAM-NFQ-MGB<br>CT: 24.75	2203 A04 SCM ato<br>U pGEM<br>2.58E4<br>FAM-NFQ-MGB<br>CT: 24.83	2203 A04 SCM ato<br>U pGEM<br>2.73E4<br>FAM-NFQ-MGB<br>CT: 24.75	2203 A04 SCM ato<br>U pGEM<br>2.77E4<br>FAM-NFQ-MGB<br>CT: 24.72				
C	S pGEM<br>2E4<br>FAM-NFQ-MGB<br>CT: 24.97	S pGEM<br>2E4<br>FAM-NFQ-MGB<br>CT: 24.96	2203 A01 SCM saki<br>U pGEM<br>2.08E4<br>FAM-NFQ-MGB<br>CT: 25.16	2203 A01 SCM saki<br>U pGEM<br>2.33E4<br>FAM-NFQ-MGB<br>CT: 24.99	2203 A01 SCM saki<br>U pGEM<br>2.23E4<br>FAM-NFQ-MGB<br>CT: 25.05	2203 A04 200 saki<br>U pGEM<br>2.06E4<br>FAM-NFQ-MGB<br>CT: 25.17	2203 A04 200 saki<br>U pGEM<br>2.14E4<br>FAM-NFQ-MGB<br>CT: 25.11	2203 A04 200 saki<br>U pGEM<br>2.04E4<br>FAM-NFQ-MGB<br>CT: 25.19				
D	S pGEM<br>2E3<br>FAM-NFQ-MGB<br>CT: 28.83	S pGEM<br>2E3<br>FAM-NFQ-MGB<br>CT: 28.8	2203 A01 SCM ato<br>U pGEM<br>2.68E4<br>FAM-NFQ-MGB<br>CT: 24.78	2203 A01 SCM ato<br>U pGEM<br>2.72E4<br>FAM-NFQ-MGB<br>CT: 24.75	2203 A01 SCM ato<br>U pGEM<br>2.72E4<br>FAM-NFQ-MGB<br>CT: 24.75	2203 A04 200 ato<br>U pGEM<br>2.56E4<br>FAM-NFQ-MGB<br>CT: 24.84	2203 A04 200 ato<br>U pGEM<br>FAM-NFQ-MGB<br>CT: 24.65	2203 A04 200 ato<br>U pGEM<br>2.48E4<br>FAM-NFQ-MGB<br>CT: 24.89				
E	S pGEM<br>200<br>FAM-NFQ-MGB<br>CT: 32.34	S pGEM<br>200<br>FAM-NFQ-MGB<br>CT: 32.45	2203 A01 BTM saki<br>U pGEM<br>2.02E4<br>FAM-NFQ-MGB<br>CT: 25.2	2203 A01 BTM saki<br>U pGEM<br>2.09E4<br>FAM-NFQ-MGB<br>CT: 25.16	2203 A01 BTM saki<br>U pGEM<br>2.14E4<br>FAM-NFQ-MGB<br>CT: 25.12	2203 A04 1000 saki<br>U pGEM<br>1.98E4<br>FAM-NFQ-MGB<br>CT: 25.24	2203 A04 1000 saki<br>U pGEM<br>2.11E4<br>FAM-NFQ-MGB<br>CT: 25.14	2203 A04 1000 saki<br>U pGEM<br>2.14E4<br>FAM-NFQ-MGB<br>CT: 25.11				
F	S pGEM<br>20<br>FAM-NFQ-MGB<br>CT: 35.97	S pGEM<br>20<br>FAM-NFQ-MGB<br>CT: 35.38	2203 A01 BTM ato<br>U pGEM<br>2.79E4<br>FAM-NFQ-MGB<br>CT: 24.71	2203 A01 BTM ato<br>U pGEM<br>2.44E4<br>FAM-NFQ-MGB<br>CT: 24.92	2203 A01 BTM ato<br>U pGEM<br>2.56E4<br>FAM-NFQ-MGB<br>CT: 24.85	2203 A04 1000 ato<br>U pGEM<br>2.68E4<br>FAM-NFQ-MGB<br>CT: 24.78	2203 A04 1000 ato<br>U pGEM<br>2.83E4<br>FAM-NFQ-MGB<br>CT: 24.69	2203 A04 1000 ato<br>U pGEM<br>2.89E4<br>FAM-NFQ-MGB<br>CT: 24.66				
G			2203 A04 10 saki<br>U pGEM<br>2E4<br>FAM-NFQ-MGB<br>CT: 25.22	2203 A04 10 saki<br>U pGEM<br>2.08E4<br>FAM-NFQ-MGB<br>CT: 25.16	2203 A04 10 saki<br>U pGEM<br>1.97E4<br>FAM-NFQ-MGB<br>CT: 25.25	2203 A04 3000 saki<br>U pGEM<br>7.95E3<br>FAM-NFQ-MGB<br>CT: 26.62	2203 A04 3000 saki<br>U pGEM<br>7.39E3<br>FAM-NFQ-MGB<br>CT: 26.73	2203 A04 3000 saki<br>U pGEM<br>7.98E3<br>FAM-NFQ-MGB<br>CT: 26.62				N pGEM<br>FAM-NFQ-MGB<br>CT: Undetermined
H	Comf2E4<br>U pGEM<br>2.04E4<br>FAM-NFQ-MGB<br>CT: 25.19	Comf2E4<br>U pGEM<br>2.34E4<br>FAM-NFQ-MGB<br>CT: 24.98	2203 A04 10 ato<br>U pGEM<br>2.51E4<br>FAM-NFQ-MGB<br>CT: 24.88	2203 A04 10 ato<br>U pGEM<br>FAM-NFQ-MGB<br>CT: 24.74	2203 A04 10 ato<br>U pGEM<br>2.74E4<br>FAM-NFQ-MGB<br>CT: 24.74	2203 A04 3000 ato<br>U pGEM<br>3.03E4<br>FAM-NFQ-MGB<br>CT: 24.59	2203 A04 3000 ato<br>U pGEM<br>2.91E4<br>FAM-NFQ-MGB<br>CT: 24.65	2203 A04 3000 ato<br>U pGEM<br>2.97E4<br>FAM-NFQ-MGB<br>CT: 24.62				N pGEM<br>FAM-NFQ-MGB<br>CT: Undetermined

Standard Curves

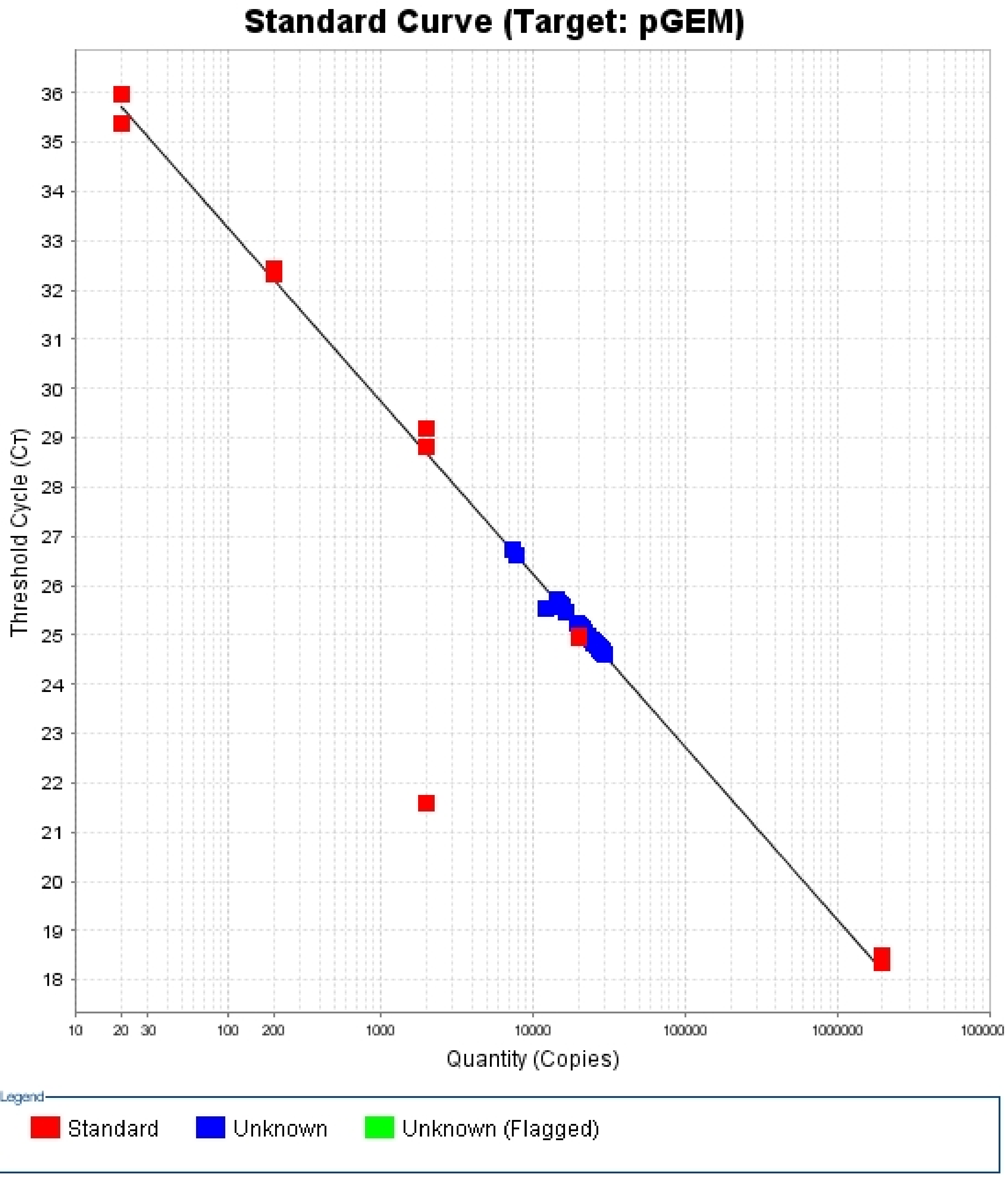

slope:-3.503 Y-Intercept:40.289 R^2:0.999 Eff%:92.943

Results Table

Well		Sample	Target	Task	Quantity	C
A1			pGEM	STANDARD	2000000	18.4927
A2			pGEM	STANDARD	2000000	18.3541
A3		2203 A01 10	pGEM	UNKNOWN	15783.4199	25.5804
A4		2203 A01 10	pGEM	UNKNOWN	14466.1699	25.713
A5		2203 A01 10	pGEM	UNKNOWN	15077.4502	25.65
A6		2203 A04 SCM	pGEM	UNKNOWN	16843.8516	25.4815
A7		2203 A04 SCM	pGEM	UNKNOWN	15454.5596	25.6124
A8		2203 A04 SCM	pGEM	UNKNOWN	12170.9785	25.5344
B1			pGEM	STANDARD	2000	21.5825
B2			pGEM	STANDARD	2000	29.1745
B3		2203 A01 10 ato	pGEM	UNKNOWN	26608.6875	24.7857
B4		2203 A01 10 ato	pGEM	UNKNOWN	27473.0605	24.7371
B5		2203 A01 10 ato	pGEM	UNKNOWN	27228.0254	24.7507
B6		2203 A04 SCM	pGEM	UNKNOWN	25799.5	24.8327
B7		2203 A04 SCM	pGEM	UNKNOWN	27270.0742	24.7484
B8		2203 A04 SCM	pGEM	UNKNOWN	27713.5625	24.7238
C1			pGEM	STANDARD	20000	24.9706
C2			pGEM	STANDARD	20000	24.9589
C3		2203 A01 SCM	pGEM	UNKNOWN	20761.0273	25.1633
C4		2203 A01 SCM	pGEM	UNKNOWN	23322.7402	24.9863
C5		2203 A01 SCM	pGEM	UNKNOWN	22301.5938	25.0544
C6		2203 A04 200	pGEM	UNKNOWN	20627.2754	25.1732
C7		2203 A04 200	pGEM	UNKNOWN	21448.8828	25.1137
C8		2203 A04 200	pGEM	UNKNOWN	20445.168	25.1866
D1			pGEM	STANDARD	2000	28.8326
D2			pGEM	STANDARD	2000	28.8042
D3		2203 A01 SCM	pGEM	UNKNOWN	26754.75	24.7774
D4		2203 A01 SCM	pGEM	UNKNOWN	27163.4551	24.7543
D5		2203 A01 SCM	pGEM	UNKNOWN	27164.1016	24.7543
D6		2203 A04 200	pGEM	UNKNOWN	25617.2266	24.8435

Well		Sample	Target	Task	Quantity	C
D7		2203 A04 200	pGEM	UNKNOWN		24.6489
D8		2203 A04 200	pGEM	UNKNOWN	24781.0605	24.894
E1			pGEM	STANDARD	200	32.3392
E2			pGEM	STANDARD	200	32.4469
E3		2203 A01 BTM	pGEM	UNKNOWN	20244.0527	25.2017
E4		2203 A01 BTM	pGEM	UNKNOWN	20864.5273	25.1558
E5		2203 A01 BTM	pGEM	UNKNOWN	21355.8926	25.1203
E6		2203 A04 1000	pGEM	UNKNOWN	19781.9629	25.2368
E7		2203 A04 1000	pGEM	UNKNOWN	21096.043	25.139
E8		2203 A04 1000	pGEM	UNKNOWN	21445.6836	25.114
F1			pGEM	STANDARD	20	35.9746
F2			pGEM	STANDARD	20	35.3758
F3		2203 A01 BTM	pGEM	UNKNOWN	27916.5605	24.7127
F4		2203 A01 BTM	pGEM	UNKNOWN	24415.4824	24.9166
F5		2203 A01 BTM	pGEM	UNKNOWN	25574.0078	24.8461
F6		2203 A04 1000	pGEM	UNKNOWN	26771.3223	24.7765
F7		2203 A04 1000	pGEM	UNKNOWN	28312.4043	24.6913
F8		2203 A04 1000	pGEM	UNKNOWN	28857.9824	24.6623
G3		2203 A04 10	pGEM	UNKNOWN	19966.6211	25.2227
G4		2203 A04 10	pGEM	UNKNOWN	20820.9727	25.1589
G5		2203 A04 10	pGEM	UNKNOWN	19674.1895	25.2451
G6		2203 A04 3000	pGEM	UNKNOWN	7948.3687	26.6242
G7		2203 A04 3000	pGEM	UNKNOWN	7392.5933	26.7345
G8		2203 A04 3000	pGEM	UNKNOWN	7983.0479	26.6176
G12			pGEM	NTC		Undetermined
H1		Comf2E4	pGEM	UNKNOWN	20378.207	25.1916
H2		Comf2E4	pGEM	UNKNOWN	23361.2754	24.9838
H3		2203 A04 10 ato	pGEM	UNKNOWN	25075.0762	24.8761
H4		2203 A04 10 ato	pGEM	UNKNOWN		24.7438
H5		2203 A04 10 ato	pGEM	UNKNOWN	27429.668	24.7395
H6		2203 A04 3000	pGEM	UNKNOWN	30282.2988	24.589
H7		2203 A04 3000	pGEM	UNKNOWN	29114.4395	24.6488

Well		Sample	Target	Task	Quantity	C
H8		2203 A04 3000	pGEM	UNKNOWN	29657.6602	24.6207
H12			pGEM	NTC		Undetermined

QC Summary

Total Wells	96	Processed Wells	64	Targets Used	1
Well Setup	64	Flagged Wells	0	Samples Used	17

Flag	Name	Frequency	Locations
AMPNC	Amplification in negative control	0	
BADROX	Bad passive reference signal	0	
BLFAIL	Baseline algorithm failed	0	
CTFAIL	C algorithm failed	0	
EXPFAIL	Exponential algorithm failed	0	
HIGHSD	High standard deviation in replicate group	0	
NOAMP	No amplification	0	
NOISE	Noise higher than others in plate	0	
NOSIGNAL	No signal in well	0	
OFFSCALE	Fluorescence is offscale	0	
OUTLIERRG	Outlier in replicate group	0	
SPIKE	Noise spikes	0	
THOLDFAIL	Thresholding algorithm failed	0	
