# Supplementary material for: Ecological impact of algal-preying protists aplanochytrids (Labyrinthulea) as a source of docosahexaenoic acid for copepods in marine environments
Source: ISME Commun. 2026 Jun 26;6(1):ycag178. doi: 10.1093/ismeco/ycag178 (PMC13685785; doi:10.1093/ismeco/ycag178)
Supplement: Supplementary file 2 [file supplementary_materials_ycag178.zip › Morimoto_ISME_C_3rdrev_supplemental_data.zip!/Morimoto_ISME_C_3rdrev_supplemental_data/Supplemental_Data_2/Standard Curve/DNA recovery/2203/DnaRec2203AlinemajiN=1_A21_2kaime.pdf]

Experiment Results Report
230620DnaRec2203AlineN=1maji2kaime

Experiment Summary

Experiment Name	:	230620DnaRec2203AlineN=1maji2kaime
Experiment Type	:	Quantitation - Standard Curve
File Name	:	230620DnaRec2203AlineN=1maji2kaime.eds
Run Started	:	2023 Jun 20 5:47:26 PM
Run Finished	:	2023 Jun 20 7:22:03 PM
Run Duration	:	94 minutes 37 seconds
Date Modified	:	2023 Jun 21 11:28:52 AM
User	:	
Number of wells used	:	40
Number of wells with results	:	40
Instrument Name	:	
Instrument Type	:	Applied Biosystems StepOnePlus™ Instrument
Comments	:	

Results Summary

Sample	Target	Quantity (Mean)	Quantity (Std Dev)	C (Mean)	C (Std Dev)
2203 A21 1000 ato1	pGEM	32742.91	1118.21	25.32	0.05
2203 A21 1000 saki1	pGEM	13131.85	143.77	26.63	0.02
2203 A21 200 ato1	pGEM	34726.62	975.66	25.24	0.04
2203 A21 200 saki1	pGEM	14377.53	974.28	26.51	0.10
2203 A21 3000 ato1	pGEM	34898.57	1685.98	25.23	0.07
2203 A21 3000 saki1	pGEM	12644.00	500.61	26.69	0.06
2203 A21 SCM ato1	pGEM	33908.28	1679.26	25.27	0.07
2203 A21 SCM saki1	pGEM	10405.72	189.35	26.97	0.03
Comf2E4	pGEM	29197.10	2259.61	25.49	0.11

Plate Layout

	1	2	3	4	5	6	7	8	9	10	11	12
A	S pGEM<br>2E6<br>FAM-NFQ-MGB<br>CT: 19.55	S pGEM<br>2E6<br>FAM-NFQ-MGB<br>CT: 19.35	2203 A21 SCM saki1<br>U pGEM<br>1.06E4<br>FAM-NFQ-MGB<br>CT: 26.95	2203 A21 SCM saki1<br>U pGEM<br>1.05E4<br>FAM-NFQ-MGB<br>CT: 26.96	2203 A21 SCM saki1<br>U pGEM<br>1.02E4<br>FAM-NFQ-MGB<br>CT: 27							
B	S pGEM<br>2E5<br>FAM-NFQ-MGB<br>CT: 22.86	S pGEM<br>2E5<br>FAM-NFQ-MGB<br>CT: 22.84	2203 A21 SCM ato1<br>U pGEM<br>3.23E4<br>FAM-NFQ-MGB<br>CT: 25.34	2203 A21 SCM ato1<br>U pGEM<br>3.57E4<br>FAM-NFQ-MGB<br>CT: 25.2	2203 A21 SCM ato1<br>U pGEM<br>3.38E4<br>FAM-NFQ-MGB<br>CT: 25.28							
C	S pGEM<br>2E4<br>FAM-NFQ-MGB<br>CT: 25.83	S pGEM<br>2E4<br>FAM-NFQ-MGB<br>CT: 25.63	2203 A21 200 saki1<br>U pGEM<br>1.37E4<br>FAM-NFQ-MGB<br>CT: 26.58	2203 A21 200 saki1<br>U pGEM<br>1.55E4<br>FAM-NFQ-MGB<br>CT: 26.4	2203 A21 200 saki1<br>U pGEM<br>1.4E4<br>FAM-NFQ-MGB<br>CT: 26.54							
D	S pGEM<br>2E3<br>FAM-NFQ-MGB<br>CT: 29.22	S pGEM<br>2E3<br>FAM-NFQ-MGB<br>CT: 29.49	2203 A21 200 ato1<br>U pGEM<br>3.37E4<br>FAM-NFQ-MGB<br>CT: 25.28	2203 A21 200 ato1<br>U pGEM<br>3.57E4<br>FAM-NFQ-MGB<br>CT: 25.2	2203 A21 200 ato1<br>U pGEM<br>3.47E4<br>FAM-NFQ-MGB<br>CT: 25.23							
E	S pGEM<br>200<br>FAM-NFQ-MGB<br>CT: 32.68	S pGEM<br>200<br>FAM-NFQ-MGB<br>CT: 32.85	2203 A21 1000 saki1<br>U pGEM<br>1.31E4<br>FAM-NFQ-MGB<br>CT: 26.64	2203 A21 1000 saki1<br>U pGEM<br>1.33E4<br>FAM-NFQ-MGB<br>CT: 26.62	2203 A21 1000 saki1<br>U pGEM<br>1.3E4<br>FAM-NFQ-MGB<br>CT: 26.65							
F	S pGEM<br>20<br>FAM-NFQ-MGB<br>CT: 35.94	S pGEM<br>20<br>FAM-NFQ-MGB<br>CT: 35.96	2203 A21 1000 ato1<br>U pGEM<br>3.24E4<br>FAM-NFQ-MGB<br>CT: 25.33	2203 A21 1000 ato1<br>U pGEM<br>3.18E4<br>FAM-NFQ-MGB<br>CT: 25.36	2203 A21 1000 ato1<br>U pGEM<br>3.4E4<br>FAM-NFQ-MGB<br>CT: 25.27							
G			2203 A21 3000 saki1<br>U pGEM<br>1.25E4<br>FAM-NFQ-MGB<br>CT: 26.7	2203 A21 3000 saki1<br>U pGEM<br>1.22E4<br>FAM-NFQ-MGB<br>CT: 26.74	2203 A21 3000 saki1<br>U pGEM<br>1.32E4<br>FAM-NFQ-MGB<br>CT: 26.63							N pGEM<br>FAM-NFQ-MGB<br>CT: Undetermined
H	Comf2E4<br>U pGEM<br>2.76E4<br>FAM-NFQ-MGB<br>CT: 25.57	Comf2E4<br>U pGEM<br>3.08E4<br>FAM-NFQ-MGB<br>CT: 25.41	2203 A21 3000 ato1<br>U pGEM<br>3.62E4<br>FAM-NFQ-MGB<br>CT: 25.18	2203 A21 3000 ato1<br>U pGEM<br>3.55E4<br>FAM-NFQ-MGB<br>CT: 25.2	2203 A21 3000 ato1<br>U pGEM<br>3.3E4<br>FAM-NFQ-MGB<br>CT: 25.31							N pGEM<br>FAM-NFQ-MGB<br>CT: Undetermined

Standard Curves

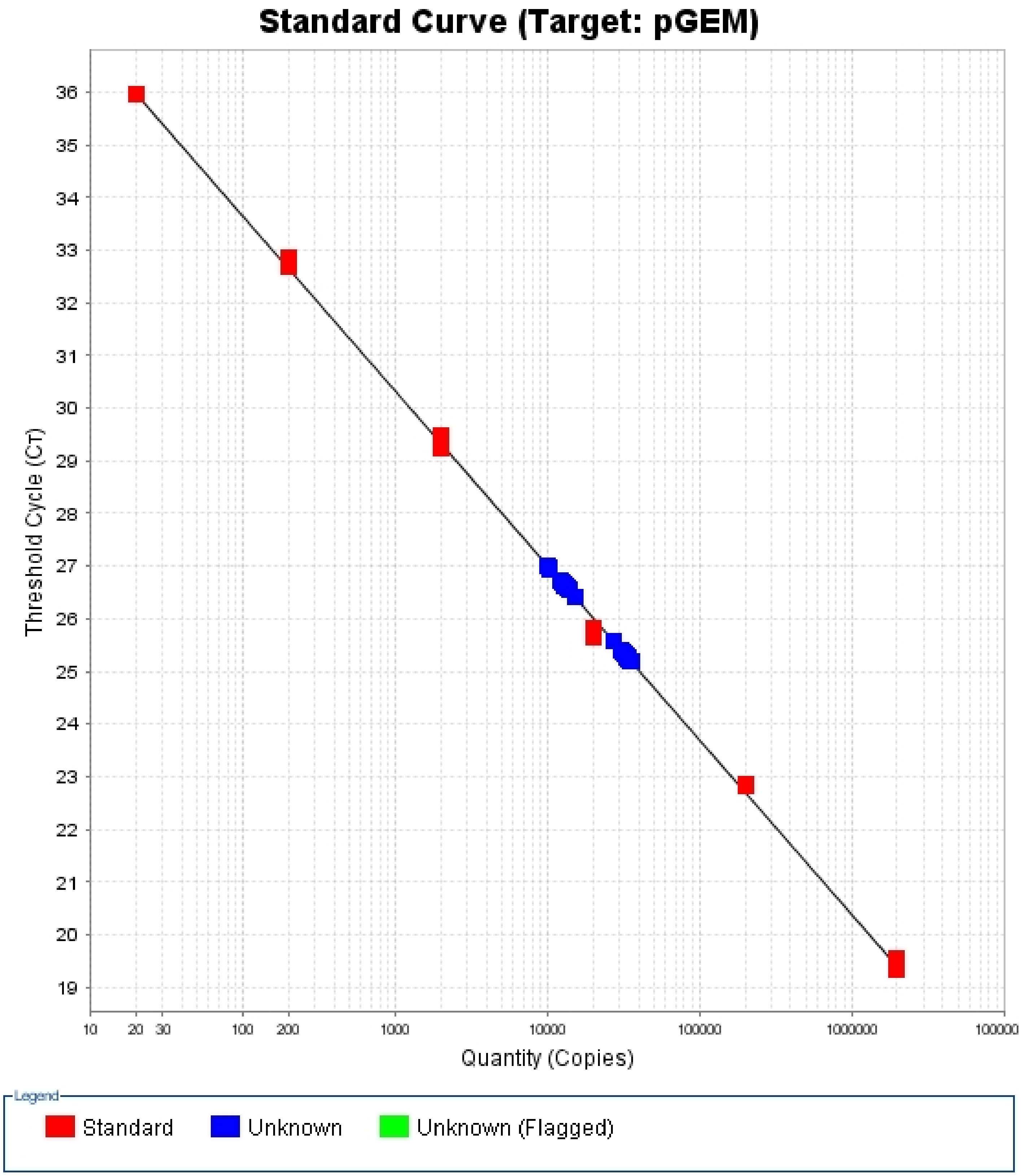

slope:-3.311 Y-Intercept:40.268 R^2:0.999 Eff%:100.471

Results Table

Well		Sample	Target	Task	Quantity	C
A1			pGEM	STANDARD	2000000	19.5498
A2			pGEM	STANDARD	2000000	19.354
A3		2203 A21 SCM	pGEM	UNKNOWN	10551.9326	26.9484
A4		2203 A21 SCM	pGEM	UNKNOWN	10473.4053	26.9591
A5		2203 A21 SCM	pGEM	UNKNOWN	10191.8301	26.9983
B1			pGEM	STANDARD	200000	22.8594
B2			pGEM	STANDARD	200000	22.8351
B3		2203 A21 SCM	pGEM	UNKNOWN	32302.7969	25.3397
B4		2203 A21 SCM	pGEM	UNKNOWN	35652.6836	25.1978
B5		2203 A21 SCM	pGEM	UNKNOWN	33769.3516	25.2758
C1			pGEM	STANDARD	20000	25.8338
C2			pGEM	STANDARD	20000	25.6316
C3		2203 A21 200	pGEM	UNKNOWN	13670.9277	26.576
C4		2203 A21 200	pGEM	UNKNOWN	15488.9531	26.3965
C5		2203 A21 200	pGEM	UNKNOWN	13972.6992	26.5446
D1			pGEM	STANDARD	2000	29.2244
D2			pGEM	STANDARD	2000	29.4881
D3		2203 A21 200	pGEM	UNKNOWN	33739.7539	25.2771
D4		2203 A21 200	pGEM	UNKNOWN	35690.6836	25.1963
D5		2203 A21 200	pGEM	UNKNOWN	34749.4414	25.2347
E1			pGEM	STANDARD	200	32.6814
E2			pGEM	STANDARD	200	32.8513
E3		2203 A21 1000	pGEM	UNKNOWN	13099.3379	26.6374
E4		2203 A21 1000	pGEM	UNKNOWN	13289.084	26.6167
E5		2203 A21 1000	pGEM	UNKNOWN	13007.1143	26.6476
F1			pGEM	STANDARD	20	35.9432
F2			pGEM	STANDARD	20	35.9585
F3		2203 A21 1000	pGEM	UNKNOWN	32425.3262	25.3342
F4		2203 A21 1000	pGEM	UNKNOWN	31817.8379	25.3614
F5		2203 A21 1000	pGEM	UNKNOWN	33985.5586	25.2666

Well		Sample	Target	Task	Quantity	C
G3		2203 A21 3000	pGEM	UNKNOWN	12506.8203	26.704
G4		2203 A21 3000	pGEM	UNKNOWN	12226.292	26.7366
G5		2203 A21 3000	pGEM	UNKNOWN	13198.9023	26.6265
G12			pGEM	NTC		Undetermined
H1		Comf2E4	pGEM	UNKNOWN	27599.3145	25.5659
H2		Comf2E4	pGEM	UNKNOWN	30794.8867	25.4084
H3		2203 A21 3000	pGEM	UNKNOWN	36180.3477	25.1767
H4		2203 A21 3000	pGEM	UNKNOWN	35526.6758	25.2029
H5		2203 A21 3000	pGEM	UNKNOWN	32988.7031	25.3094
H12			pGEM	NTC		Undetermined

QC Summary

Total Wells	96	Processed Wells	40	Targets Used	1
Well Setup	40	Flagged Wells	0	Samples Used	9

Flag	Name	Frequency	Locations
AMPNC	Amplification in negative control	0	
BADROX	Bad passive reference signal	0	
BLFAIL	Baseline algorithm failed	0	
CTFAIL	C algorithm failed	0	
EXPFAIL	Exponential algorithm failed	0	
HIGHSD	High standard deviation in replicate group	0	
NOAMP	No amplification	0	
NOISE	Noise higher than others in plate	0	
NOSIGNAL	No signal in well	0	
OFFSCALE	Fluorescence is offscale	0	
OUTLIERRG	Outlier in replicate group	0	
SPIKE	Noise spikes	0	
THOLDFAIL	Thresholding algorithm failed	0	
