# Supplementary material for: Ecological impact of algal-preying protists aplanochytrids (Labyrinthulea) as a source of docosahexaenoic acid for copepods in marine environments
Source: ISME Commun. 2026 Jun 26;6(1):ycag178. doi: 10.1093/ismeco/ycag178 (PMC13685785; doi:10.1093/ismeco/ycag178)
Supplement: Supplementary file 2 [file supplementary_materials_ycag178.zip › Morimoto_ISME_C_3rdrev_supplemental_data.zip!/Morimoto_ISME_C_3rdrev_supplemental_data/Supplemental_Data_2/Standard Curve/DNA recovery/2203/DnaRec2203AlinemajiN=1A07-A11.pdf]

Experiment Results Report
230619DnaRec2203AlinemajiN=1A07-A11

Experiment Summary

Experiment Name	:	230619DnaRec2203AlinemajiN=1A07-A11
Experiment Type	:	Quantitation - Standard Curve
File Name	:	230619DnaRec2203AlinemajiN=1A07-A11.eds
Run Started	:	2023 Jun 19 4:25:27 PM
Run Finished	:	2023 Jun 19 6:00:02 PM
Run Duration	:	94 minutes 35 seconds
Date Modified	:	2023 Jun 20 11:37:34 AM
User	:	
Number of wells used	:	70
Number of wells with results	:	70
Instrument Name	:	
Instrument Type	:	Applied Biosystems StepOnePlus™ Instrument
Comments	:	

Results Summary

Sample	Target	Quantity (Mean)	Quantity (Std Dev)	C (Mean)	C (Std Dev)
2203 A07 10 ato1	pGEM	38198.51	1923.35	23.58	0.07
2203 A07 10 saki1	pGEM	15316.68	1858.47	24.94	0.18
2203 A07 1000 ato1	pGEM	39348.23	1348.68	23.54	0.05
2203 A07 1000 saki1	pGEM	19486.91	612.30	24.58	0.05
2203 A07 200 ato1	pGEM	37832.10	3022.88	23.60	0.12
2203 A07 200 saki1	pGEM	19500.11	685.32	24.57	0.05
2203 A07 3000 ato1	pGEM	36415.52	3451.54	23.65	0.14
2203 A07 3000 saki1	pGEM	20127.31	535.25	24.53	0.04
2203 A07 SCM ato1	pGEM	37040.91	747.92	23.62	0.03
2203 A07 SCM saki1	pGEM	18309.29	439.24	24.67	0.04
2203 A11 1000 ato1	pGEM	37622.56	875.91	23.60	0.03
2203 A11 1000 saki1	pGEM	15664.56	727.80	24.90	0.07
2203 A11 200 ato1	pGEM	41488.86	4515.06	23.46	0.17
2203 A11 200 saki1	pGEM	17146.81	590.99	24.77	0.05
2203 A11 3000 ato1	pGEM	42027.33	2358.55	23.44	0.08
2203 A11 3000 saki1	pGEM	15527.75	390.87	24.91	0.04
2203 A11 SCM ato1	pGEM	61277.15	5995.48	22.88	0.15
2203 A11 SCM saki1	pGEM	247.71	25.66	31.04	0.16
Comf2E4	pGEM	19514.66	912.56	24.57	0.07

Plate Layout

	1	2	3	4	5	6	7	8	9	10	11	12
A	S pGEM<br>2E6<br>FAM-NFQ-MGB<br>CT: 17.84	S pGEM<br>2E6<br>FAM-NFQ-MGB<br>CT: 17.54	2203 A07 10 saki1<br>U pGEM<br>1.69E4<br>FAM-NFQ-MGB<br>CT: 24.78	2203 A07 10 saki1<br>U pGEM<br>1.57E4<br>FAM-NFQ-MGB<br>CT: 24.89	2203 A07 10 saki1<br>U pGEM<br>1.33E4<br>FAM-NFQ-MGB<br>CT: 25.14	2203 A07 3000 saki1<br>U pGEM<br>2.01E4<br>FAM-NFQ-MGB<br>CT: 24.53	2203 A11 SCM saki1<br>U pGEM<br>263.99<br>FAM-NFQ-MGB<br>CT: 30.94	2203 A11 SCM saki1<br>U pGEM<br>261.02<br>FAM-NFQ-MGB<br>CT: 30.96	2203 A11 SCM saki1<br>U pGEM<br>218.13<br>FAM-NFQ-MGB<br>CT: 31.23			
B	S pGEM<br>2E5<br>FAM-NFQ-MGB<br>CT: 20.9	S pGEM<br>2E5<br>FAM-NFQ-MGB<br>CT: 20.88	2203 A07 10 ato1<br>U pGEM<br>3.74E4<br>FAM-NFQ-MGB<br>CT: 23.61	2203 A07 10 ato1<br>U pGEM<br>4.04E4<br>FAM-NFQ-MGB<br>CT: 23.5	2203 A07 10 ato1<br>U pGEM<br>3.68E4<br>FAM-NFQ-MGB<br>CT: 23.63	2203 A07 3000 saki1<br>U pGEM<br>2.07E4<br>FAM-NFQ-MGB<br>CT: 24.49	2203 A11 SCM ato1<br>U pGEM<br>5.5E4<br>FAM-NFQ-MGB<br>CT: 23.04	2203 A11 SCM ato1<br>U pGEM<br>6.2E4<br>FAM-NFQ-MGB<br>CT: 22.86	2203 A11 SCM ato1<br>U pGEM<br>6.69E4<br>FAM-NFQ-MGB<br>CT: 22.75			
C	S pGEM<br>2E4<br>FAM-NFQ-MGB<br>CT: 24.58	S pGEM<br>2E4<br>FAM-NFQ-MGB<br>CT: 24.4	2203 A07 SCM saki1<br>U pGEM<br>1.83E4<br>FAM-NFQ-MGB<br>CT: 24.66	2203 A07 SCM saki1<br>U pGEM<br>1.87E4<br>FAM-NFQ-MGB<br>CT: 24.63	2203 A07 SCM saki1<br>U pGEM<br>1.79E4<br>FAM-NFQ-MGB<br>CT: 24.7	2203 A07 3000 saki1<br>U pGEM<br>1.96E4<br>FAM-NFQ-MGB<br>CT: 24.56	2203 A11 200 saki1<br>U pGEM<br>1.78E4<br>FAM-NFQ-MGB<br>CT: 24.71	2203 A11 200 saki1<br>U pGEM<br>1.68E4<br>FAM-NFQ-MGB<br>CT: 24.79	2203 A11 200 saki1<br>U pGEM<br>1.68E4<br>FAM-NFQ-MGB<br>CT: 24.8			
D	! S pGEM<br>2E3<br>FAM-NFQ-MGB<br>CT: 28.94	! S pGEM<br>2E3<br>FAM-NFQ-MGB<br>CT: 27.88	2203 A07 SCM ato1<br>U pGEM<br>3.77E4<br>FAM-NFQ-MGB<br>CT: 23.6	2203 A07 SCM ato1<br>U pGEM<br>3.72E4<br>FAM-NFQ-MGB<br>CT: 23.62	2203 A07 SCM ato1<br>U pGEM<br>3.62E4<br>FAM-NFQ-MGB<br>CT: 23.66	2203 A07 3000 ato1<br>U pGEM<br>3.89E4<br>FAM-NFQ-MGB<br>CT: 23.55	2203 A11 200 ato1<br>U pGEM<br>4.41E4<br>FAM-NFQ-MGB<br>CT: 23.36	2203 A11 200 ato1<br>U pGEM<br>3.63E4<br>FAM-NFQ-MGB<br>CT: 23.66	2203 A11 200 ato1<br>U pGEM<br>4.4E4<br>FAM-NFQ-MGB<br>CT: 23.37			
E	S pGEM<br>200<br>FAM-NFQ-MGB<br>CT: 31.95	S pGEM<br>200<br>FAM-NFQ-MGB<br>CT: 31.4	2203 A07 200 saki1<br>U pGEM<br>2E4<br>FAM-NFQ-MGB<br>CT: 24.53	2203 A07 200 saki1<br>U pGEM<br>1.97E4<br>FAM-NFQ-MGB<br>CT: 24.56	2203 A07 200 saki1<br>U pGEM<br>1.87E4<br>FAM-NFQ-MGB<br>CT: 24.63	2203 A07 3000 ato1<br>U pGEM<br>3.25E4<br>FAM-NFQ-MGB<br>CT: 23.82	2203 A11 1000 saki1<br>U pGEM<br>1.52E4<br>FAM-NFQ-MGB<br>CT: 24.95	2203 A11 1000 saki1<br>U pGEM<br>1.53E4<br>FAM-NFQ-MGB<br>CT: 24.93	2203 A11 1000 saki1<br>U pGEM<br>1.65E4<br>FAM-NFQ-MGB<br>CT: 24.82			
F	S pGEM<br>20<br>FAM-NFQ-MGB<br>CT: 34.33	S pGEM<br>20<br>FAM-NFQ-MGB<br>CT: 34.26	2203 A07 200 ato1<br>U pGEM<br>4.13E4<br>FAM-NFQ-MGB<br>CT: 23.46	2203 A07 200 ato1<br>U pGEM<br>3.64E4<br>FAM-NFQ-MGB<br>CT: 23.65	2203 A07 200 ato1<br>U pGEM<br>3.58E4<br>FAM-NFQ-MGB<br>CT: 23.67	2203 A07 3000 ato1<br>U pGEM<br>3.79E4<br>FAM-NFQ-MGB<br>CT: 23.59	2203 A11 1000 ato1<br>U pGEM<br>3.83E4<br>FAM-NFQ-MGB<br>CT: 23.57	2203 A11 1000 ato1<br>U pGEM<br>3.79E4<br>FAM-NFQ-MGB<br>CT: 23.59	2203 A11 1000 ato1<br>U pGEM<br>3.66E4<br>FAM-NFQ-MGB<br>CT: 23.64			
G			2203 A07 1000 saki1<br>U pGEM<br>1.99E4<br>FAM-NFQ-MGB<br>CT: 24.55	2203 A07 1000 saki1<br>U pGEM<br>1.88E4<br>FAM-NFQ-MGB<br>CT: 24.63	2203 A07 1000 saki1<br>U pGEM<br>1.98E4<br>FAM-NFQ-MGB<br>CT: 24.55		2203 A11 3000 saki1<br>U pGEM<br>1.51E4<br>FAM-NFQ-MGB<br>CT: 24.96	2203 A11 3000 saki1<br>U pGEM<br>1.58E4<br>FAM-NFQ-MGB<br>CT: 24.89	2203 A11 3000 saki1<br>U pGEM<br>1.57E4<br>FAM-NFQ-MGB<br>CT: 24.89			N pGEM<br>FAM-NFQ-MGB<br>CT: 36.97
H	Comf2E4<br>U pGEM<br>1.89E4<br>FAM-NFQ-MGB<br>CT: 24.62	Comf2E4<br>U pGEM<br>2.02E4<br>FAM-NFQ-MGB<br>CT: 24.52	2203 A07 1000 ato1<br>U pGEM<br>3.95E4<br>FAM-NFQ-MGB<br>CT: 23.53	2203 A07 1000 ato1<br>U pGEM<br>4.06E4<br>FAM-NFQ-MGB<br>CT: 23.49	2203 A07 1000 ato1<br>U pGEM<br>3.79E4<br>FAM-NFQ-MGB<br>CT: 23.59		2203 A11 3000 ato1<br>U pGEM<br>3.97E4<br>FAM-NFQ-MGB<br>CT: 23.52	2203 A11 3000 ato1<br>U pGEM<br>4.19E4<br>FAM-NFQ-MGB<br>CT: 23.44	2203 A11 3000 ato1<br>U pGEM<br>4.45E4<br>FAM-NFQ-MGB<br>CT: 23.35			N pGEM<br>FAM-NFQ-MGB<br>CT: 35.97

Standard Curves

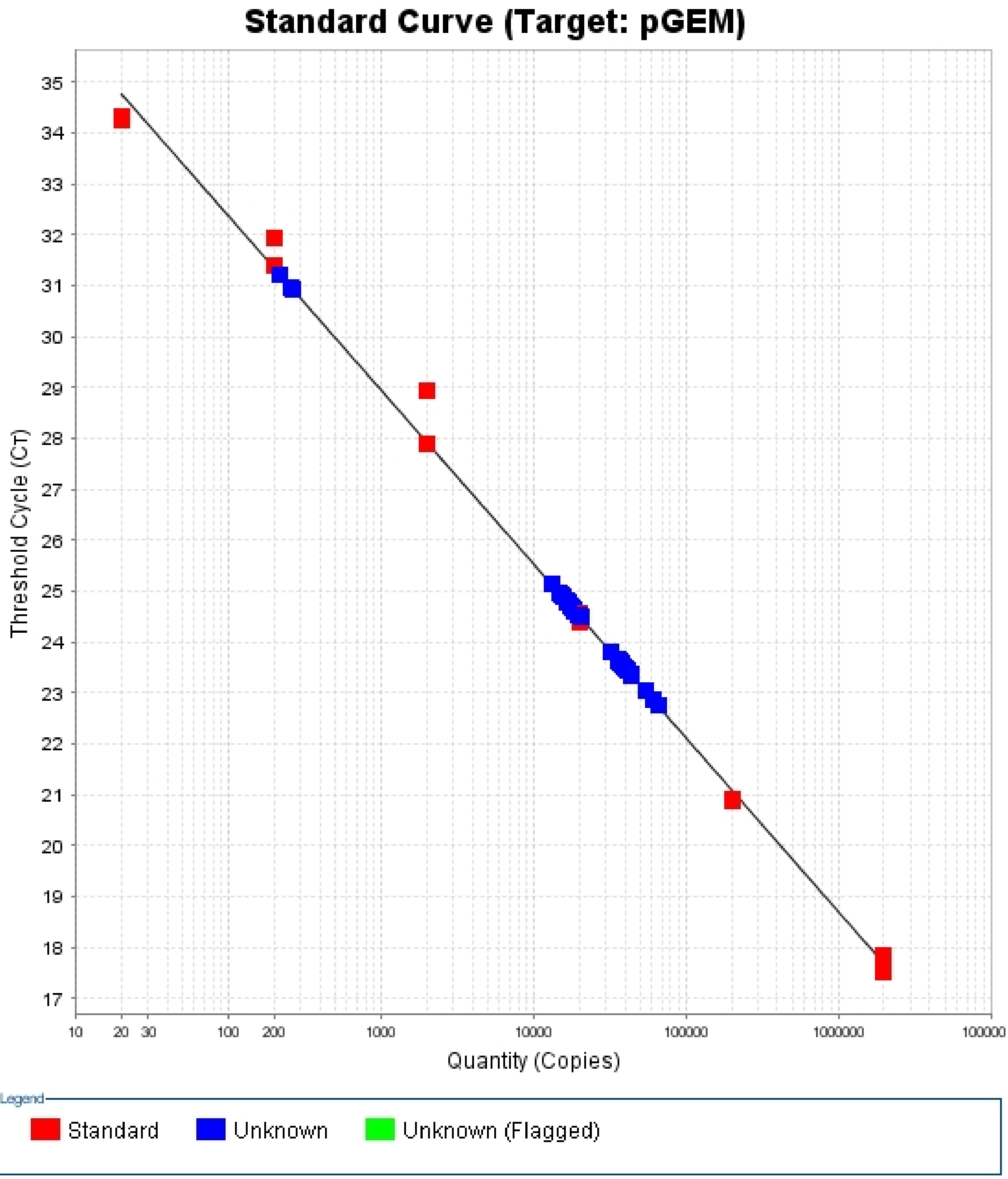

slope:-3.409 Y-Intercept:39.201 R^2:0.995 Eff%:96.471

Results Table

Well		Sample	Target	Task	Quantity	C
A1			pGEM	STANDARD	2000000	17.8381
A2			pGEM	STANDARD	2000000	17.5365
A3		2203 A07 10	pGEM	UNKNOWN	16922.207	24.7842
A4		2203 A07 10	pGEM	UNKNOWN	15747.0537	24.8907
A5		2203 A07 10	pGEM	UNKNOWN	13280.7803	25.143
A6		2203 A07 3000	pGEM	UNKNOWN	20068.4238	24.5317
A7		2203 A11 SCM	pGEM	UNKNOWN	263.9926	30.9447
A8		2203 A11 SCM	pGEM	UNKNOWN	261.016	30.9615
A9		2203 A11 SCM	pGEM	UNKNOWN	218.1262	31.2273
B1			pGEM	STANDARD	200000	20.9022
B2			pGEM	STANDARD	200000	20.8763
B3		2203 A07 10	pGEM	UNKNOWN	37408.1055	23.6096
B4		2203 A07 10	pGEM	UNKNOWN	40391.1289	23.496
B5		2203 A07 10	pGEM	UNKNOWN	36796.2891	23.634
B6		2203 A07 3000	pGEM	UNKNOWN	20689.5684	24.4865
B7		2203 A11 SCM	pGEM	UNKNOWN	54964.9336	23.0398
B8		2203 A11 SCM	pGEM	UNKNOWN	61971.0117	22.8621
B9		2203 A11 SCM	pGEM	UNKNOWN	66895.5078	22.7489
C1			pGEM	STANDARD	20000	24.5778
C2			pGEM	STANDARD	20000	24.3971
C3		2203 A07 SCM	pGEM	UNKNOWN	18342.2324	24.6649
C4		2203 A07 SCM	pGEM	UNKNOWN	18731.1328	24.6338
C5		2203 A07 SCM	pGEM	UNKNOWN	17854.5117	24.7048
C6		2203 A07 3000	pGEM	UNKNOWN	19623.9316	24.5648
C7		2203 A11 200	pGEM	UNKNOWN	17827.9219	24.707
C8		2203 A11 200	pGEM	UNKNOWN	16842.832	24.7911
C9		2203 A11 200	pGEM	UNKNOWN	16769.6816	24.7976
D1			pGEM	STANDARD	2000	28.9409
D2			pGEM	STANDARD	2000	27.8833
D3		2203 A07 SCM	pGEM	UNKNOWN	37703.5391	23.5979

Well		Sample	Target	Task	Quantity	C
D4		2203 A07 SCM	pGEM	UNKNOWN	37189.2617	23.6183
D5		2203 A07 SCM	pGEM	UNKNOWN	36229.9258	23.657
D6		2203 A07 3000	pGEM	UNKNOWN	38871.7656	23.5527
D7		2203 A11 200	pGEM	UNKNOWN	44147.0586	23.3643
D8		2203 A11 200	pGEM	UNKNOWN	36275.6445	23.6551
D9		2203 A11 200	pGEM	UNKNOWN	44043.8516	23.3678
E1			pGEM	STANDARD	200	31.946
E2			pGEM	STANDARD	200	31.402
E3		2203 A07 200	pGEM	UNKNOWN	20033.1172	24.5343
E4		2203 A07 200	pGEM	UNKNOWN	19740.1484	24.5561
E5		2203 A07 200	pGEM	UNKNOWN	18727.0566	24.6341
E6		2203 A07 3000	pGEM	UNKNOWN	32469.2617	23.8192
E7		2203 A11 1000	pGEM	UNKNOWN	15160.8965	24.9469
E8		2203 A11 1000	pGEM	UNKNOWN	15333.7891	24.9301
E9		2203 A11 1000	pGEM	UNKNOWN	16499	24.8217
F1			pGEM	STANDARD	20	34.333
F2			pGEM	STANDARD	20	34.2634
F3		2203 A07 200	pGEM	UNKNOWN	41305.2109	23.4628
F4		2203 A07 200	pGEM	UNKNOWN	36397.0547	23.6501
F5		2203 A07 200	pGEM	UNKNOWN	35794.0273	23.6749
F6		2203 A07 3000	pGEM	UNKNOWN	37905.5312	23.59
F7		2203 A11 1000	pGEM	UNKNOWN	38335.332	23.5733
F8		2203 A11 1000	pGEM	UNKNOWN	37887.6172	23.5907
F9		2203 A11 1000	pGEM	UNKNOWN	36644.7383	23.6401
G3		2203 A07 1000	pGEM	UNKNOWN	19877.9883	24.5458
G4		2203 A07 1000	pGEM	UNKNOWN	18781.2656	24.6298
G5		2203 A07 1000	pGEM	UNKNOWN	19801.4727	24.5515
G7		2203 A11 3000	pGEM	UNKNOWN	15077.1543	24.9551
G8		2203 A11 3000	pGEM	UNKNOWN	15775.4766	24.8881
G9		2203 A11 3000	pGEM	UNKNOWN	15730.6113	24.8923
G12			pGEM	NTC		36.9719
H1		Comf2E4	pGEM	UNKNOWN	18869.3867	24.6229

Well		Sample	Target	Task	Quantity	C
H2		Comf2E4	pGEM	UNKNOWN	20159.9355	24.5249
H3		2203 A07 1000	pGEM	UNKNOWN	39527.3086	23.528
H4		2203 A07 1000	pGEM	UNKNOWN	40598.4219	23.4884
H5		2203 A07 1000	pGEM	UNKNOWN	37918.9609	23.5895
H7		2203 A11 3000	pGEM	UNKNOWN	39741.1172	23.52
H8		2203 A11 3000	pGEM	UNKNOWN	41888.7617	23.4421
H9		2203 A11 3000	pGEM	UNKNOWN	44452.1172	23.3541
H12			pGEM	NTC		35.9692

QC Summary

Total Wells	96	Processed Wells	70	Targets Used	1
Well Setup	70	Flagged Wells	2	Samples Used	19

Flag	Name	Frequency	Locations
AMPNC	Amplification in negative control	0	
BADROX	Bad passive reference signal	0	
BLFAIL	Baseline algorithm failed	0	
CTFAIL	C algorithm failed	0	
EXPFAIL	Exponential algorithm failed	0	
HIGHSD	High standard deviation in replicate group	2	D1, D2
NOAMP	No amplification	0	
NOISE	Noise higher than others in plate	0	
NOSIGNAL	No signal in well	0	
OFFSCALE	Fluorescence is offscale	0	
OUTLIERRG	Outlier in replicate group	0	
SPIKE	Noise spikes	0	
THOLDFAIL	Thresholding algorithm failed	0	
