# Supplementary material for: Ecological impact of algal-preying protists aplanochytrids (Labyrinthulea) as a source of docosahexaenoic acid for copepods in marine environments
Source: ISME Commun. 2026 Jun 26;6(1):ycag178. doi: 10.1093/ismeco/ycag178 (PMC13685785; doi:10.1093/ismeco/ycag178)
Supplement: Supplementary file 2 [file supplementary_materials_ycag178.zip › Morimoto_ISME_C_3rdrev_supplemental_data.zip!/Morimoto_ISME_C_3rdrev_supplemental_data/Supplemental_Data_2/Standard Curve/DNA recovery/2107/DnaRec2107Aline_A21_N=2-2kaime.pdf]

Experiment Results Report

230719DnaRec2107AlineA21N=2-2kaime

Experiment Summary

Experiment Name	: 230719DnaRec2107AlineA21N=2-2kaime
Experiment Type	: Quantitation - Standard Curve
File Name	: 230719DnaRec2107AlineA21N=2-2kaime.eds
Run Started	: 2023 Jul 19 5:55:57 PM
Run Finished	: 2023 Jul 19 7:30:33 PM
Run Duration	: 94 minutes 35 seconds
Date Modified	: 2023 Jul 20 10:50:33 AM
User	:
Number of wells used	: 40
Number of wells with results	: 40
Instrument Name	:
Instrument Type	: Applied Biosystems StepOnePlus™ Instrument
Comments	:

Results Summary

Sample	Target	Quantity (Mean)	Quantity (Std Dev)	C (Mean)	C (Std Dev)
2107 A21 1000m ato	pGEM	26236.05	719.17	26.04	0.04
2107 A21 1000m	pGEM	16862.10	980.90	26.70	0.09
2107 A21 200m ato	pGEM	28325.64	679.86	25.93	0.04
2107 A21 200m saki	pGEM	18406.00	1203.55	26.57	0.10
2107 A21 3000m ato	pGEM	30415.91	1753.48	25.82	0.09
2107 A21 3000m	pGEM	6413.32	311.06	28.15	0.07
2107 A21 SCM ato	pGEM	29643.71	1910.86	25.86	0.10
2107 A21 SCM saki	pGEM	15598.70	1136.95	26.82	0.11
Comf2E4	pGEM	22607.30	1162.10	26.26	0.08

Plate Layout

	1	2	3	4	5	6	7	8	9	10	11	12
A	S pGEM<br>2E6<br>FAM-NFQ-MGB<br>CT: 19.77	S pGEM<br>2E6<br>FAM-NFQ-MGB<br>CT: 19.71	2107 A21 SCM saki<br>U pGEM<br>1.56E4<br>FAM-NFQ-MGB<br>CT: 26.82	2107 A21 SCM saki<br>U pGEM<br>1.67E4<br>FAM-NFQ-MGB<br>CT: 26.71	2107 A21 SCM saki<br>U pGEM<br>1.45E4<br>FAM-NFQ-MGB<br>CT: 26.93							
B	S pGEM<br>2E5<br>FAM-NFQ-MGB<br>CT: 22.78	S pGEM<br>2E5<br>FAM-NFQ-MGB<br>CT: 22.7	2107 A21 SCM ato<br>U pGEM<br>3.03E4<br>FAM-NFQ-MGB<br>CT: 25.83	2107 A21 SCM ato<br>U pGEM<br>2.75E4<br>FAM-NFQ-MGB<br>CT: 25.97	2107 A21 SCM ato<br>U pGEM<br>3.12E4<br>FAM-NFQ-MGB<br>CT: 25.78							
C	S pGEM<br>2E4<br>FAM-NFQ-MGB<br>CT: 26.59	S pGEM<br>2E4<br>FAM-NFQ-MGB<br>CT: 26.29	2107 A21 200m saki<br>U pGEM<br>1.77E4<br>FAM-NFQ-MGB<br>CT: 26.63	2107 A21 200m saki<br>U pGEM<br>1.98E4<br>FAM-NFQ-MGB<br>CT: 26.46	2107 A21 200m saki<br>U pGEM<br>1.77E4<br>FAM-NFQ-MGB<br>CT: 26.63							
D	S pGEM<br>2E3<br>FAM-NFQ-MGB<br>CT: 29.97	S pGEM<br>2E3<br>FAM-NFQ-MGB<br>CT: 30.06	2107 A21 200m ato<br>U pGEM<br>2.76E4<br>FAM-NFQ-MGB<br>CT: 25.97	2107 A21 200m ato<br>U pGEM<br>2.89E4<br>FAM-NFQ-MGB<br>CT: 25.9	2107 A21 200m ato<br>U pGEM<br>2.85E4<br>FAM-NFQ-MGB<br>CT: 25.92							
E	S pGEM<br>200<br>FAM-NFQ-MGB<br>CT: 33.25	S pGEM<br>200<br>FAM-NFQ-MGB<br>CT: 33.36	2107 A21 1000m saki<br>U pGEM<br>1.8E4<br>FAM-NFQ-MGB<br>CT: 26.6	2107 A21 1000m saki<br>U pGEM<br>1.63E4<br>FAM-NFQ-MGB<br>CT: 26.75	2107 A21 1000m saki<br>U pGEM<br>1.63E4<br>FAM-NFQ-MGB<br>CT: 26.75							
F	S pGEM<br>20<br>FAM-NFQ-MGB<br>CT: 36.58	S pGEM<br>20<br>FAM-NFQ-MGB<br>CT: 36.93	2107 A21 1000m ato<br>U pGEM<br>2.61E4<br>FAM-NFQ-MGB<br>CT: 26.05	2107 A21 1000m ato<br>U pGEM<br>2.56E4<br>FAM-NFQ-MGB<br>CT: 26.08	2107 A21 1000m ato<br>U pGEM<br>2.7E4<br>FAM-NFQ-MGB<br>CT: 26							
G			2107 A21 3000m saki<br>U pGEM<br>6.2E3<br>FAM-NFQ-MGB<br>CT: 28.2	2107 A21 3000m saki<br>U pGEM<br>6.77E3<br>FAM-NFQ-MGB<br>CT: 28.06	2107 A21 3000m saki<br>U pGEM<br>6.27E3<br>FAM-NFQ-MGB<br>CT: 28.18							N pGEM<br>FAM-NFQ-MGB<br>CT: Undetermined
H	Comf2E4<br>U pGEM<br>2.34E4<br>FAM-NFQ-MGB<br>CT: 26.21	Comf2E4<br>U pGEM<br>2.18E4<br>FAM-NFQ-MGB<br>CT: 26.32	2107 A21 3000m ato<br>U pGEM<br>2.89E4<br>FAM-NFQ-MGB<br>CT: 25.89	2107 A21 3000m ato<br>U pGEM<br>2.99E4<br>FAM-NFQ-MGB<br>CT: 25.84	2107 A21 3000m ato<br>U pGEM<br>3.24E4<br>FAM-NFQ-MGB<br>CT: 25.73							N pGEM<br>FAM-NFQ-MGB<br>CT: Undetermined

Standard Curves

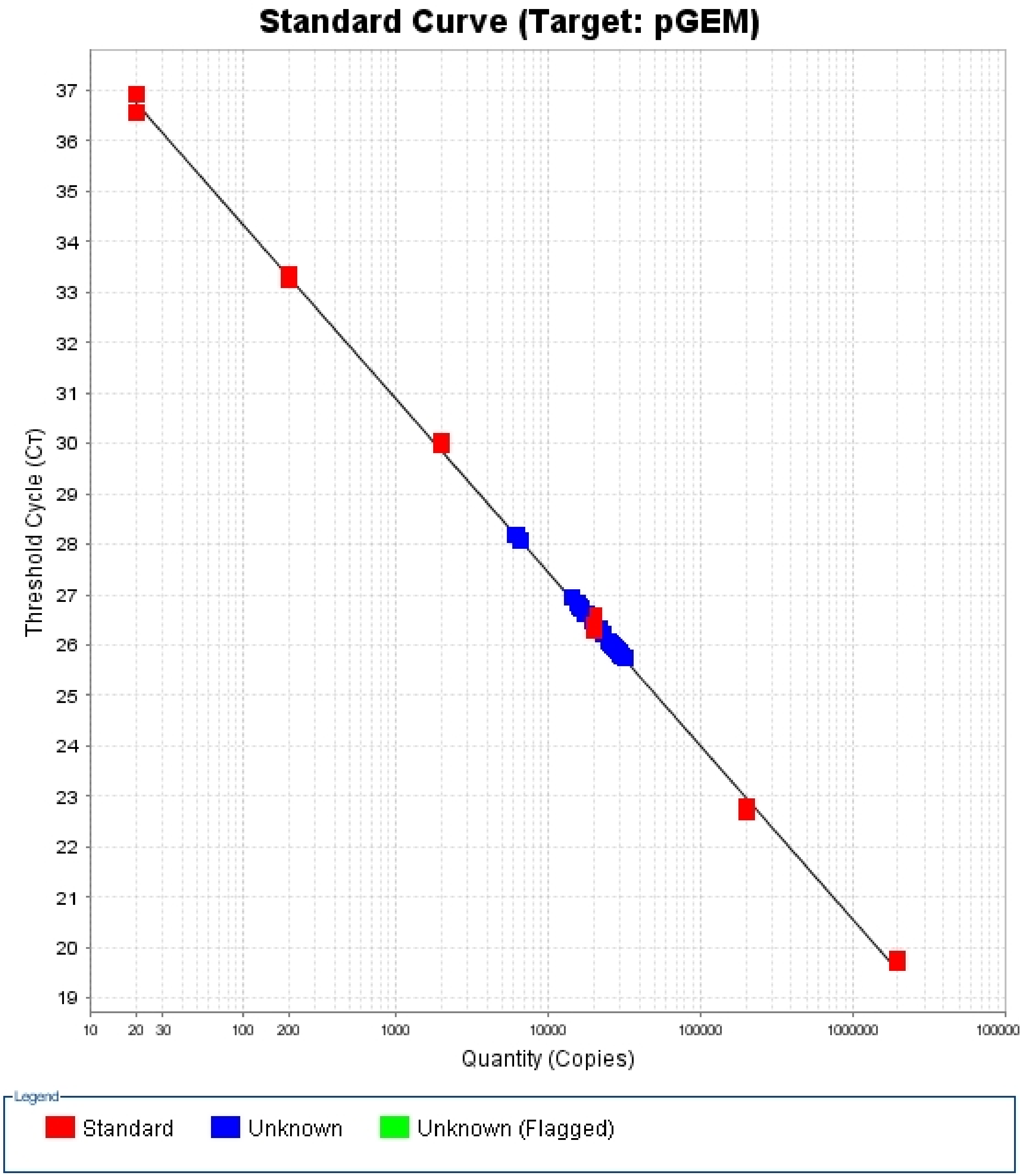

slope:-3.438 Y-Intercept:41.236 R^2:0.999 Eff%:95.355

Results Table

Well		Sample	Target	Task	Quantity	C
A1			pGEM	STANDARD	2000000	19.7745
A2			pGEM	STANDARD	2000000	19.7105
A3		2107 A21 SCM	pGEM	UNKNOWN	15572.1855	26.8204
A4		2107 A21 SCM	pGEM	UNKNOWN	16748.6758	26.7116
A5		2107 A21 SCM	pGEM	UNKNOWN	14475.2314	26.9295
B1			pGEM	STANDARD	200000	22.7787
B2			pGEM	STANDARD	200000	22.6992
B3		2107 A21 SCM	pGEM	UNKNOWN	30285.4668	25.8271
B4		2107 A21 SCM	pGEM	UNKNOWN	27494.5801	25.9714
B5		2107 A21 SCM	pGEM	UNKNOWN	31151.0898	25.785
C1			pGEM	STANDARD	20000	26.5851
C2			pGEM	STANDARD	20000	26.2865
C3		2107 A21 200m	pGEM	UNKNOWN	17711.6387	26.6282
C4		2107 A21 200m	pGEM	UNKNOWN	19795.7402	26.462
C5		2107 A21 200m	pGEM	UNKNOWN	17710.6191	26.6282
D1			pGEM	STANDARD	2000	29.9731
D2			pGEM	STANDARD	2000	30.0569
D3		2107 A21 200m	pGEM	UNKNOWN	27575.1172	25.9671
D4		2107 A21 200m	pGEM	UNKNOWN	28900.25	25.897
D5		2107 A21 200m	pGEM	UNKNOWN	28501.5605	25.9177
E1			pGEM	STANDARD	200	33.2549
E2			pGEM	STANDARD	200	33.3617
E3		2107 A21	pGEM	UNKNOWN	17994.6172	26.6045
E4		2107 A21	pGEM	UNKNOWN	16310.5742	26.7512
E5		2107 A21	pGEM	UNKNOWN	16281.1016	26.7539
F1			pGEM	STANDARD	20	36.5795
F2			pGEM	STANDARD	20	36.93
F3		2107 A21	pGEM	UNKNOWN	26066.8633	26.0511
F4		2107 A21	pGEM	UNKNOWN	25616.5684	26.0771
F5		2107 A21	pGEM	UNKNOWN	27024.7305	25.9972

Well		Sample	Target	Task	Quantity	C
G3		2107 A21	pGEM	UNKNOWN	6197.9321	28.1962
G4		2107 A21	pGEM	UNKNOWN	6769.9365	28.0643
G5		2107 A21	pGEM	UNKNOWN	6272.0889	28.1784
G12			pGEM	NTC		Undetermined
H1		Comf2E4	pGEM	UNKNOWN	23429.0332	26.2104
H2		Comf2E4	pGEM	UNKNOWN	21785.5723	26.319
H3		2107 A21	pGEM	UNKNOWN	28944.3945	25.8947
H4		2107 A21	pGEM	UNKNOWN	29947.2363	25.8438
H5		2107 A21	pGEM	UNKNOWN	32356.1074	25.7283
H12			pGEM	NTC		Undetermined

QC Summary

Total Wells	96	Processed Wells	40	Targets Used	1
Well Setup	40	Flagged Wells	0	Samples Used	9

Flag	Name	Frequency	Locations
AMPNC	Amplification in negative control	0	
BADROX	Bad passive reference signal	0	
BLFAIL	Baseline algorithm failed	0	
CTFAIL	C algorithm failed	0	
EXPFAIL	Exponential algorithm failed	0	
HIGHSD	High standard deviation in replicate group	0	
NOAMP	No amplification	0	
NOISE	Noise higher than others in plate	0	
NOSIGNAL	No signal in well	0	
OFFSCALE	Fluorescence is offscale	0	
OUTLIERRG	Outlier in replicate group	0	
SPIKE	Noise spikes	0	
THOLDFAIL	Thresholding algorithm failed	0	
