# Supplementary material for: Ecological impact of algal-preying protists aplanochytrids (Labyrinthulea) as a source of docosahexaenoic acid for copepods in marine environments
Source: ISME Commun. 2026 Jun 26;6(1):ycag178. doi: 10.1093/ismeco/ycag178 (PMC13685785; doi:10.1093/ismeco/ycag178)
Supplement: Supplementary file 2 [file supplementary_materials_ycag178.zip › Morimoto_ISME_C_3rdrev_supplemental_data.zip!/Morimoto_ISME_C_3rdrev_supplemental_data/Supplemental_Data_2/Standard Curve/DNA recovery/2107/DnaRec2107AlineA04-A07N=2.pdf]

Experiment Results Report
230718DnaRec2107AlineA04-A07N=2

Experiment Summary

Experiment Name	:	230718DnaRec2107AlineA04-A07N=2
Experiment Type	:	Quantitation - Standard Curve
File Name	:	230718DnaRec2107AlineA04-A07N=2.eds
Run Started	:	2023 Jul 18 2:27:15 PM
Run Finished	:	2023 Jul 18 4:01:50 PM
Run Duration	:	94 minutes 35 seconds
Date Modified	:	2023 Jul 19 11:37:12 AM
User	:	
Number of wells used	:	70
Number of wells with results	:	70
Instrument Name	:	
Instrument Type	:	Applied Biosystems StepOnePlus™ Instrument
Comments	:	

Results Summary

Sample	Target	Quantity (Mean)	Quantity (Std Dev)	C (Mean)	C (Std Dev)
2107 A04 1000m ato	pGEM	30247.10	1411.77	24.87	0.07
2107 A04 1000m	pGEM	22386.52	2420.11	25.34	0.17
2107 A04 10m ato	pGEM	29618.48	2514.09	24.90	0.13
2107 A04 10m saki	pGEM	23117.94	12355.59	25.41	0.76
2107 A04 200m ato	pGEM	34008.55	2522.89	24.69	0.11
2107 A04 200m saki	pGEM	25147.91	1442.31	25.15	0.09
2107 A04 3000m ato	pGEM	30233.50	447.18	24.87	0.02
2107 A04 3000m	pGEM	17789.90	535.17	25.69	0.05
2107 A04 SCM ato	pGEM	32367.52	4482.27	24.77	0.22
2107 A04 SCM saki	pGEM	23189.29	3464.00	25.29	0.22
2107 A07 1000m ato	pGEM	30077.99	270.58	24.87	0.01
2107 A07 1000m	pGEM	27158.58	932.89	25.03	0.05
2107 A07 200m ato	pGEM	33150.04	4161.87	24.73	0.20
2107 A07 200m saki	pGEM	22729.31	3329.33	25.32	0.24
2107 A07 3000m ato	pGEM	35075.75	710.60	24.64	0.03
2107 A07 3000m	pGEM	28191.54	1626.38	24.98	0.09
2107 A07 SCM ato	pGEM	30342.73	1429.76	24.86	0.07
2107 A07 SCM saki	pGEM	17229.48	1098.72	25.74	0.10
Comf2E4	pGEM	19156.74	898.83	25.57	0.07

Plate Layout

	1	2	3	4	5	6	7	8	9	10	11	12
A	S pGEM<br>2E6<br>FAM-NFQ-MGB<br>CT: 18.89	S pGEM<br>2E6<br>FAM-NFQ-MGB<br>CT: 18.39	⚠2<br>2107 A04 10m saki<br>U pGEM<br>3.74E4<br>FAM-NFQ-MGB<br>CT: 24.54	⚠1<br>2107 A04 10m saki<br>U pGEM<br>1.58E4<br>FAM-NFQ-MGB<br>CT: 25.87	⚠1<br>2107 A04 10m saki<br>U pGEM<br>1.62E4<br>FAM-NFQ-MGB<br>CT: 25.83	⚠1<br>2107 A04 SCM saki<br>U pGEM<br>2.72E4<br>FAM-NFQ-MGB<br>CT: 25.03	2107 A04 SCM saki<br>U pGEM<br>2.11E4<br>FAM-NFQ-MGB<br>CT: 25.42	2107 A04 SCM saki<br>U pGEM<br>2.12E4<br>FAM-NFQ-MGB<br>CT: 25.41	2107 A07 SCM saki<br>U pGEM<br>1.84E4<br>FAM-NFQ-MGB<br>CT: 25.64	2107 A07 SCM saki<br>U pGEM<br>1.62E4<br>FAM-NFQ-MGB<br>CT: 25.83	2107 A07 SCM saki<br>U pGEM<br>1.72E4<br>FAM-NFQ-MGB<br>CT: 25.74	
B	S pGEM<br>2E5<br>FAM-NFQ-MGB<br>CT: 21.5	S pGEM<br>2E5<br>FAM-NFQ-MGB<br>CT: 21.74	2107 A04 10m ato<br>U pGEM<br>3.19E4<br>FAM-NFQ-MGB<br>CT: 24.78	2107 A04 10m ato<br>U pGEM<br>2.69E4<br>FAM-NFQ-MGB<br>CT: 25.05	2107 A04 10m ato<br>U pGEM<br>3.01E4<br>FAM-NFQ-MGB<br>CT: 24.88	2107 A04 SCM ato<br>U pGEM<br>2.76E4<br>FAM-NFQ-MGB<br>CT: 25.01	2107 A04 SCM ato<br>U pGEM<br>3.65E4<br>FAM-NFQ-MGB<br>CT: 24.58	2107 A04 SCM ato<br>U pGEM<br>3.31E4<br>FAM-NFQ-MGB<br>CT: 24.73	2107 A07 SCM ato<br>U pGEM<br>2.88E4<br>FAM-NFQ-MGB<br>CT: 24.94	2107 A07 SCM ato<br>U pGEM<br>3.16E4<br>FAM-NFQ-MGB<br>CT: 24.8	2107 A07 SCM ato<br>U pGEM<br>3.07E4<br>FAM-NFQ-MGB<br>CT: 24.84	
C	S pGEM<br>2E4<br>FAM-NFQ-MGB<br>CT: 25.62	S pGEM<br>2E4<br>FAM-NFQ-MGB<br>CT: 25.22				2107 A04 200m saki<br>U pGEM<br>2.35E4<br>FAM-NFQ-MGB<br>CT: 25.25	2107 A04 200m saki<br>U pGEM<br>2.56E4<br>FAM-NFQ-MGB<br>CT: 25.12	2107 A04 200m saki<br>U pGEM<br>2.63E4<br>FAM-NFQ-MGB<br>CT: 25.08	2107 A07 200m saki<br>U pGEM<br>1.9E4<br>FAM-NFQ-MGB<br>CT: 25.58	2107 A07 200m saki<br>U pGEM<br>2.54E4<br>FAM-NFQ-MGB<br>CT: 25.14	2107 A07 200m saki<br>U pGEM<br>2.38E4<br>FAM-NFQ-MGB<br>CT: 25.23	
D	S pGEM<br>2E3<br>FAM-NFQ-MGB<br>CT: 28.99	S pGEM<br>2E3<br>FAM-NFQ-MGB<br>CT: 29.22				2107 A04 200m ato<br>U pGEM<br>3.24E4<br>FAM-NFQ-MGB<br>CT: 24.76	2107 A04 200m ato<br>U pGEM<br>3.69E4<br>FAM-NFQ-MGB<br>CT: 24.56	2107 A04 200m ato<br>U pGEM<br>3.27E4<br>FAM-NFQ-MGB<br>CT: 24.74	2107 A07 200m ato<br>U pGEM<br>3.39E4<br>FAM-NFQ-MGB<br>CT: 24.69	2107 A07 200m ato<br>U pGEM<br>3.69E4<br>FAM-NFQ-MGB<br>CT: 24.56	2107 A07 200m ato<br>U pGEM<br>2.87E4<br>FAM-NFQ-MGB<br>CT: 24.95	
E	S pGEM<br>200<br>FAM-NFQ-MGB<br>CT: 32.95	S pGEM<br>200<br>FAM-NFQ-MGB<br>CT: 32.72				2107 A04 1000m saki<br>U pGEM<br>1.96E4<br>FAM-NFQ-MGB<br>CT: 25.54	2107 A04 1000m saki<br>U pGEM<br>2.38E4<br>FAM-NFQ-MGB<br>CT: 25.24	2107 A04 1000m saki<br>U pGEM<br>2.38E4<br>FAM-NFQ-MGB<br>CT: 25.24	2107 A07 1000m saki<br>U pGEM<br>2.82E4<br>FAM-NFQ-MGB<br>CT: 24.97	2107 A07 1000m saki<br>U pGEM<br>2.69E4<br>FAM-NFQ-MGB<br>CT: 25.04	2107 A07 1000m saki<br>U pGEM<br>2.64E4<br>FAM-NFQ-MGB<br>CT: 25.08	
F	⚠1<br>S pGEM<br>20<br>FAM-NFQ-MGB<br>CT: 35.14	⚠1<br>S pGEM<br>20<br>FAM-NFQ-MGB<br>CT: 37.02				2107 A04 1000m ato<br>U pGEM<br>3.01E4<br>FAM-NFQ-MGB<br>CT: 24.87	2107 A04 1000m ato<br>U pGEM<br>2.89E4<br>FAM-NFQ-MGB<br>CT: 24.94	2107 A04 1000m ato<br>U pGEM<br>3.17E4<br>FAM-NFQ-MGB<br>CT: 24.79	2107 A07 1000m ato<br>U pGEM<br>3.01E4<br>FAM-NFQ-MGB<br>CT: 24.87	2107 A07 1000m ato<br>U pGEM<br>2.98E4<br>FAM-NFQ-MGB<br>CT: 24.89	2107 A07 1000m ato<br>U pGEM<br>3.03E4<br>FAM-NFQ-MGB<br>CT: 24.86	
G						2107 A04 3000m saki<br>U pGEM<br>1.79E4<br>FAM-NFQ-MGB<br>CT: 25.67	2107 A04 3000m saki<br>U pGEM<br>1.72E4<br>FAM-NFQ-MGB<br>CT: 25.74	2107 A04 3000m saki<br>U pGEM<br>1.82E4<br>FAM-NFQ-MGB<br>CT: 25.65	2107 A07 3000m saki<br>U pGEM<br>2.76E4<br>FAM-NFQ-MGB<br>CT: 25.01	2107 A07 3000m saki<br>U pGEM<br>3E4<br>FAM-NFQ-MGB<br>CT: 24.88	2107 A07 3000m saki<br>U pGEM<br>2.69E4<br>FAM-NFQ-MGB<br>CT: 25.04	N pGEM<br>FAM-NFQ-MGB<br>CT: Undetermined
H	Comf2E4<br>U pGEM<br>1.85E4<br>FAM-NFQ-MGB<br>CT: 25.62	Comf2E4<br>U pGEM<br>1.98E4<br>FAM-NFQ-MGB<br>CT: 25.52				2107 A04 3000m ato<br>U pGEM<br>3.07E4<br>FAM-NFQ-MGB<br>CT: 24.84	2107 A04 3000m ato<br>U pGEM<br>3.03E4<br>FAM-NFQ-MGB<br>CT: 24.86	2107 A04 3000m ato<br>U pGEM<br>2.98E4<br>FAM-NFQ-MGB<br>CT: 24.89	2107 A07 3000m ato<br>U pGEM<br>3.43E4<br>FAM-NFQ-MGB<br>CT: 24.67	2107 A07 3000m ato<br>U pGEM<br>3.57E4<br>FAM-NFQ-MGB<br>CT: 24.61	2107 A07 3000m ato<br>U pGEM<br>3.52E4<br>FAM-NFQ-MGB<br>CT: 24.63	N pGEM<br>FAM-NFQ-MGB<br>CT: Undetermined

Standard Curves

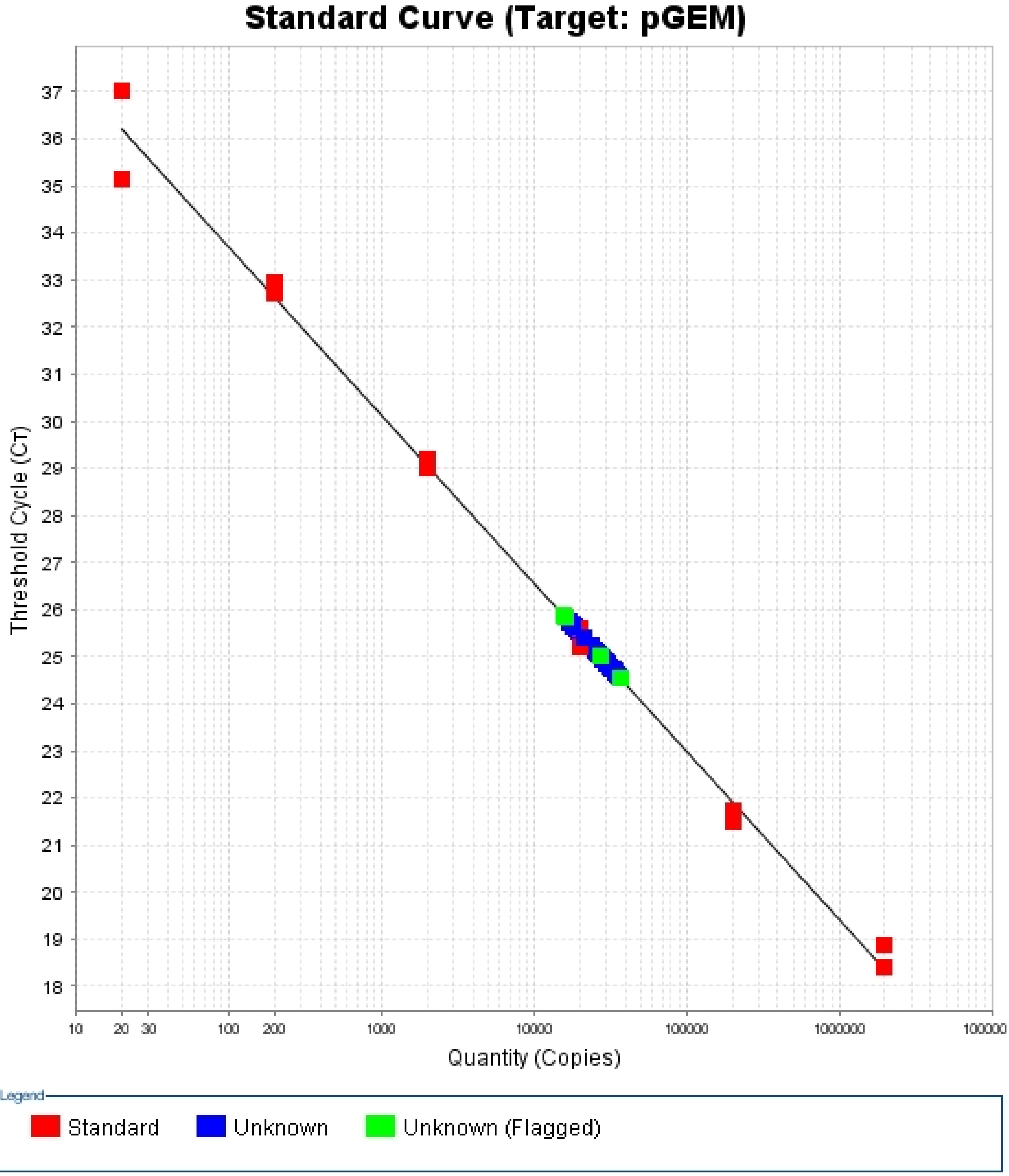

slope:-3.558 Y-Intercept:40.807 R^2:0.994 Eff%:91.017

Results Table

Well		Sample	Target	Task	Quantity	C
A1			pGEM	STANDARD	2000000	18.8914
A2			pGEM	STANDARD	2000000	18.3921
A3		2107 A04 10m	pGEM	UNKNOWN	37383.5273	24.5379
A4		2107 A04 10m	pGEM	UNKNOWN	15811.0801	25.8675
A5		2107 A04 10m	pGEM	UNKNOWN	16159.2061	25.8339
A6		2107 A04 SCM	pGEM	UNKNOWN	27188.8145	25.0299
A7		2107 A04 SCM	pGEM	UNKNOWN	21142.9785	25.4185
A8		2107 A04 SCM	pGEM	UNKNOWN	21236.0703	25.4117
A9		2107 A07 SCM	pGEM	UNKNOWN	18353.8242	25.6371
A10		2107 A07 SCM	pGEM	UNKNOWN	16158.3086	25.834
A11		2107 A07 SCM	pGEM	UNKNOWN	17176.3066	25.7396
B1			pGEM	STANDARD	200000	21.4977
B2			pGEM	STANDARD	200000	21.7404
B3		2107 A04 10m	pGEM	UNKNOWN	31888.8027	24.7836
B4		2107 A04 10m	pGEM	UNKNOWN	26916.5176	25.0455
B5		2107 A04 10m	pGEM	UNKNOWN	30050.125	24.8753
B6		2107 A04 SCM	pGEM	UNKNOWN	27582.4375	25.0077
B7		2107 A04 SCM	pGEM	UNKNOWN	36468.2852	24.5762
B8		2107 A04 SCM	pGEM	UNKNOWN	33051.8203	24.7282
B9		2107 A07 SCM	pGEM	UNKNOWN	28771.1855	24.9425
B10		2107 A07 SCM	pGEM	UNKNOWN	31566.5527	24.7992
B11		2107 A07 SCM	pGEM	UNKNOWN	30690.4648	24.8427
C1			pGEM	STANDARD	20000	25.6199
C2			pGEM	STANDARD	20000	25.2179
C6		2107 A04 200m	pGEM	UNKNOWN	23534.9824	25.2529
C7		2107 A04 200m	pGEM	UNKNOWN	25595.0859	25.1233
C8		2107 A04 200m	pGEM	UNKNOWN	26313.668	25.0805
C9		2107 A07 200m	pGEM	UNKNOWN	18993.8066	25.5842
C10		2107 A07 200m	pGEM	UNKNOWN	25383.8008	25.1361
C11		2107 A07 200m	pGEM	UNKNOWN	23810.3398	25.2349

Well		Sample	Target	Task	Quantity	C
D1			pGEM	STANDARD	2000	28.9916
D2			pGEM	STANDARD	2000	29.2155
D6		2107 A04 200m	pGEM	UNKNOWN	32401.7461	24.7589
D7		2107 A04 200m	pGEM	UNKNOWN	36916.3711	24.5573
D8		2107 A04 200m	pGEM	UNKNOWN	32707.5215	24.7444
D9		2107 A07 200m	pGEM	UNKNOWN	33909.1445	24.6886
D10		2107 A07 200m	pGEM	UNKNOWN	36880.1133	24.5589
D11		2107 A07 200m	pGEM	UNKNOWN	28660.8711	24.9485
E1			pGEM	STANDARD	200	32.9477
E2			pGEM	STANDARD	200	32.7207
E6		2107 A04	pGEM	UNKNOWN	19592.1055	25.5362
E7		2107 A04	pGEM	UNKNOWN	23764.3262	25.2379
E8		2107 A04	pGEM	UNKNOWN	23803.1094	25.2354
E9		2107 A07	pGEM	UNKNOWN	28180.1406	24.9746
E10		2107 A07	pGEM	UNKNOWN	26943.7773	25.0439
E11		2107 A07	pGEM	UNKNOWN	26351.8301	25.0782
F1			pGEM	STANDARD	20	35.1423
F2			pGEM	STANDARD	20	37.0193
F6		2107 A04	pGEM	UNKNOWN	30113.4746	24.8721
F7		2107 A04	pGEM	UNKNOWN	28906.8906	24.9352
F8		2107 A04	pGEM	UNKNOWN	31720.9238	24.7917
F9		2107 A07	pGEM	UNKNOWN	30099.1289	24.8728
F10		2107 A07	pGEM	UNKNOWN	29797.4648	24.8884
F11		2107 A07	pGEM	UNKNOWN	30337.377	24.8606
G6		2107 A04	pGEM	UNKNOWN	17927.4102	25.6734
G7		2107 A04	pGEM	UNKNOWN	17199.3906	25.7375
G8		2107 A04	pGEM	UNKNOWN	18242.8965	25.6465
G9		2107 A07	pGEM	UNKNOWN	27602.6699	25.0066
G10		2107 A07	pGEM	UNKNOWN	30030.3223	24.8763
G11		2107 A07	pGEM	UNKNOWN	26941.6152	25.044
G12			pGEM	NTC		Undetermined
H1		Comf2E4	pGEM	UNKNOWN	18521.1758	25.6231

Well		Sample	Target	Task	Quantity	C
H2		Comf2E4	pGEM	UNKNOWN	19792.3086	25.5205
H6		2107 A04	pGEM	UNKNOWN	30671.3379	24.8437
H7		2107 A04	pGEM	UNKNOWN	30251.627	24.865
H8		2107 A04	pGEM	UNKNOWN	29777.5352	24.8894
H9		2107 A07	pGEM	UNKNOWN	34315.75	24.6702
H10		2107 A07	pGEM	UNKNOWN	35723.6172	24.6081
H11		2107 A07	pGEM	UNKNOWN	35187.8711	24.6314
H12			pGEM	NTC		Undetermined

QC Summary

Total Wells	96	Processed Wells	70	Targets Used	1
Well Setup	70	Flagged Wells	6	Samples Used	19

Flag	Name	Frequency	Locations
AMPNC	Amplification in negative control	0	
BADROX	Bad passive reference signal	0	
BLFAIL	Baseline algorithm failed	0	
CTFAIL	C algorithm failed	0	
EXPFAIL	Exponential algorithm failed	0	
HIGHSD	High standard deviation in replicate group	5	A3, A4, A5, F1, F2
NOAMP	No amplification	0	
NOISE	Noise higher than others in plate	0	
NOSIGNAL	No signal in well	0	
OFFSCALE	Fluorescence is offscale	0	
OUTLIERRG	Outlier in replicate group	2	A3, A6
SPIKE	Noise spikes	0	
THOLDFAIL	Thresholding algorithm failed	0	
