# Supplementary material for: Ecological impact of algal-preying protists aplanochytrids (Labyrinthulea) as a source of docosahexaenoic acid for copepods in marine environments
Source: ISME Commun. 2026 Jun 26;6(1):ycag178. doi: 10.1093/ismeco/ycag178 (PMC13685785; doi:10.1093/ismeco/ycag178)
Supplement: Supplementary file 2 [file supplementary_materials_ycag178.zip › Morimoto_ISME_C_3rdrev_supplemental_data.zip!/Morimoto_ISME_C_3rdrev_supplemental_data/Supplemental_Data_2/Standard Curve/DNA recovery/2107/DnaRec2107AlineA01-A07N=1.pdf]

Experiment Results Report

230714DnaRec2107AlineA01-A07N=1

Experiment Summary

Experiment Name	:	230714DnaRec2107AlineA01-A07N=1
Experiment Type	:	Quantitation - Standard Curve
File Name	:	230714DnaRec2107AlineA01-A07N=1.eds
Run Started	:	2023 Jul 14 4:00:30 PM
Run Finished	:	2023 Jul 14 5:35:06 PM
Run Duration	:	94 minutes 36 seconds
Date Modified	:	2023 Jul 14 5:40:10 PM
User	:	
Number of wells used	:	88
Number of wells with results	:	88
Instrument Name	:	
Instrument Type	:	Applied Biosystems StepOnePlus™ Instrument
Comments	:	

Results Summary

Sample	Target	Quantity (Mean)	Quantity (Std Dev)	C (Mean)	C (Std Dev)
2107 A01 10m ato	pGEM	36401.26	2255.33	25.67	0.09
2107 A01 10m ato2	pGEM	25138.36	811.94	26.23	0.05
2107 A01 10m saki	pGEM	24112.36	1959.25	26.29	0.12
2107 A01 10m saki2	pGEM	27491.24	1516.87	26.09	0.08
2107 A01 BTM ato	pGEM	33210.89	589.00	25.81	0.03
2107 A01 BTM saki	pGEM	36808.79	1039.30	25.66	0.04
2107 A01 SCM ato	pGEM	35875.61	2669.58	25.70	0.11
2107 A01 SCM saki	pGEM	30907.17	3956.25	25.92	0.19
2107 A04 1000m ato	pGEM	36992.21	4488.27	25.65	0.18
2107 A04 1000m	pGEM	33663.45	1223.43	25.79	0.05
2107 A04 10m ato	pGEM	38217.42	2733.49	25.60	0.11
2107 A04 10m saki	pGEM	22623.53	3800.30	26.40	0.24
2107 A04 200m ato	pGEM	36160.48	3192.39	25.69	0.13
2107 A04 200m saki	pGEM	30208.21	871.09	25.95	0.04
2107 A04 3000m ato	pGEM	37439.65	1917.50	25.63	0.08
2107 A04 3000m	pGEM	31209.03	1842.12	25.90	0.09
2107 A07 1000m ato	pGEM	33969.56	1099.37	25.78	0.05
2107 A07 1000m	pGEM	31315.01	1232.45	25.90	0.06
2107 A07 200m ato	pGEM	36701.55	566.44	25.66	0.02
2107 A07 200m saki	pGEM	29376.24	4741.20	26.01	0.25
2107 A07 3000m ato	pGEM	40712.33	6166.74	25.52	0.23
2107 A07 3000m	pGEM	28673.80	2022.24	26.03	0.10
2107 A07 SCM ato	pGEM	36019.62	1925.73	25.69	0.08
2107 A07 SCM saki	pGEM	21152.03	867.40	26.49	0.06
Comf2E4	pGEM	20420.73	212.77	26.54	0.02

Plate Layout

	1	2	3	4	5	6	7	8	9	10	11	12
A	S pGEM<br>2E6<br>FAM-NFQ-MGB<br>CT: 19.89	S pGEM<br>2E6<br>FAM-NFQ-MGB<br>CT: 19.47	2107 A01 10m saki<br>U pGEM<br>2.3E4<br>FAM-NFQ-MGB<br>CT: 26.36	2107 A01 10m saki<br>U pGEM<br>2.3E4<br>FAM-NFQ-MGB<br>CT: 26.36	2107 A01 10m saki<br>U pGEM<br>2.64E4<br>FAM-NFQ-MGB<br>CT: 26.15	2107 A04 10m saki<br>U pGEM<br>2.01E4<br>FAM-NFQ-MGB<br>CT: 26.56	2107 A04 10m saki<br>U pGEM<br>2.7E4<br>FAM-NFQ-MGB<br>CT: 26.12	2107 A04 10m saki<br>U pGEM<br>2.07E4<br>FAM-NFQ-MGB<br>CT: 26.51	2107 A07 SCM saki<br>U pGEM<br>2.03E4<br>FAM-NFQ-MGB<br>CT: 26.54	2107 A07 SCM saki<br>U pGEM<br>2.21E4<br>FAM-NFQ-MGB<br>CT: 26.42	2107 A07 SCM saki<br>U pGEM<br>2.11E4<br>FAM-NFQ-MGB<br>CT: 26.49	
B	S pGEM<br>2E5<br>FAM-NFQ-MGB<br>CT: 23.11	S pGEM<br>2E5<br>FAM-NFQ-MGB<br>CT: 23.01	2107 A01 10m ato<br>U pGEM<br>3.49E4<br>FAM-NFQ-MGB<br>CT: 25.74	2107 A01 10m ato<br>U pGEM<br>3.9E4<br>FAM-NFQ-MGB<br>CT: 25.57	2107 A01 10m ato<br>U pGEM<br>3.53E4<br>FAM-NFQ-MGB<br>CT: 25.72	2107 A04 10m ato<br>U pGEM<br>3.67E4<br>FAM-NFQ-MGB<br>CT: 25.66	2107 A04 10m ato<br>U pGEM<br>4.14E4<br>FAM-NFQ-MGB<br>CT: 25.48	2107 A04 10m ato<br>U pGEM<br>3.66E4<br>FAM-NFQ-MGB<br>CT: 25.66	2107 A07 SCM ato<br>U pGEM<br>3.53E4<br>FAM-NFQ-MGB<br>CT: 25.72	2107 A07 SCM ato<br>U pGEM<br>3.45E4<br>FAM-NFQ-MGB<br>CT: 25.75	2107 A07 SCM ato<br>U pGEM<br>3.82E4<br>FAM-NFQ-MGB<br>CT: 25.6	
C	S pGEM<br>2E4<br>FAM-NFQ-MGB<br>CT: 26.66	S pGEM<br>2E4<br>FAM-NFQ-MGB<br>CT: 26.46	2107 A01 SCM saki<br>U pGEM<br>2.95E4<br>FAM-NFQ-MGB<br>CT: 25.99	2107 A01 SCM saki<br>U pGEM<br>3.54E4<br>FAM-NFQ-MGB<br>CT: 25.71	2107 A01 SCM saki<br>U pGEM<br>2.79E4<br>FAM-NFQ-MGB<br>CT: 26.07	2107 A04 200m saki<br>U pGEM<br>2.96E4<br>FAM-NFQ-MGB<br>CT: 25.98	2107 A04 200m saki<br>U pGEM<br>2.98E4<br>FAM-NFQ-MGB<br>CT: 25.97	2107 A04 200m saki<br>U pGEM<br>3.12E4<br>FAM-NFQ-MGB<br>CT: 25.9	2107 A07 200m saki<br>U pGEM<br>2.4E4<br>FAM-NFQ-MGB<br>CT: 26.3	2107 A07 200m saki<br>U pGEM<br>3.28E4<br>FAM-NFQ-MGB<br>CT: 25.83	2107 A07 200m saki<br>U pGEM<br>3.14E4<br>FAM-NFQ-MGB<br>CT: 25.89	
D	S pGEM<br>2E3<br>FAM-NFQ-MGB<br>CT: 29.98	S pGEM<br>2E3<br>FAM-NFQ-MGB<br>CT: 30.02	2107 A01 SCM ato<br>U pGEM<br>3.33E4<br>FAM-NFQ-MGB<br>CT: 25.81	2107 A01 SCM ato<br>U pGEM<br>3.86E4<br>FAM-NFQ-MGB<br>CT: 25.58	2107 A01 SCM ato<br>U pGEM<br>3.58E4<br>FAM-NFQ-MGB<br>CT: 25.7	2107 A04 200m ato<br>U pGEM<br>3.34E4<br>FAM-NFQ-MGB<br>CT: 25.8	2107 A04 200m ato<br>U pGEM<br>3.97E4<br>FAM-NFQ-MGB<br>CT: 25.54	2107 A04 200m ato<br>U pGEM<br>3.54E4<br>FAM-NFQ-MGB<br>CT: 25.71	2107 A07 200m ato<br>U pGEM<br>3.73E4<br>FAM-NFQ-MGB<br>CT: 25.63	2107 A07 200m ato<br>U pGEM<br>3.65E4<br>FAM-NFQ-MGB<br>CT: 25.67	2107 A07 200m ato<br>U pGEM<br>3.63E4<br>FAM-NFQ-MGB<br>CT: 25.68	
E	S pGEM<br>200<br>FAM-NFQ-MGB<br>CT: 33.82	S pGEM<br>200<br>FAM-NFQ-MGB<br>CT: 33.59	2107 A01 BTM saki<br>U pGEM<br>3.65E4<br>FAM-NFQ-MGB<br>CT: 25.67	2107 A01 BTM saki<br>U pGEM<br>3.8E4<br>FAM-NFQ-MGB<br>CT: 25.61	2107 A01 BTM saki<br>U pGEM<br>3.6E4<br>FAM-NFQ-MGB<br>CT: 25.69	2107 A04 1000m saki<br>U pGEM<br>3.25E4<br>FAM-NFQ-MGB<br>CT: 25.84	2107 A04 1000m saki<br>U pGEM<br>3.49E4<br>FAM-NFQ-MGB<br>CT: 25.73	2107 A04 1000m saki<br>U pGEM<br>3.36E4<br>FAM-NFQ-MGB<br>CT: 25.79	2107 A07 1000m saki<br>U pGEM<br>3.25E4<br>FAM-NFQ-MGB<br>CT: 25.84	2107 A07 1000m saki<br>U pGEM<br>3.15E4<br>FAM-NFQ-MGB<br>CT: 25.89	2107 A07 1000m saki<br>U pGEM<br>3E4<br>FAM-NFQ-MGB<br>CT: 25.96	
F	S pGEM<br>20<br>FAM-NFQ-MGB<br>CT: 36.96	S pGEM<br>20<br>FAM-NFQ-MGB<br>CT: 36.56	2107 A01 BTM ato<br>U pGEM<br>3.26E4<br>FAM-NFQ-MGB<br>CT: 25.84	2107 A01 BTM ato<br>U pGEM<br>3.34E4<br>FAM-NFQ-MGB<br>CT: 25.8	2107 A01 BTM ato<br>U pGEM<br>3.37E4<br>FAM-NFQ-MGB<br>CT: 25.79	2107 A04 1000m ato<br>U pGEM<br>3.26E4<br>FAM-NFQ-MGB<br>CT: 25.84	2107 A04 1000m ato<br>U pGEM<br>4.16E4<br>FAM-NFQ-MGB<br>CT: 25.47	2107 A04 1000m ato<br>U pGEM<br>3.68E4<br>FAM-NFQ-MGB<br>CT: 25.65	2107 A07 1000m ato<br>U pGEM<br>3.34E4<br>FAM-NFQ-MGB<br>CT: 25.8	2107 A07 1000m ato<br>U pGEM<br>3.52E4<br>FAM-NFQ-MGB<br>CT: 25.72	2107 A07 1000m ato<br>U pGEM<br>3.33E4<br>FAM-NFQ-MGB<br>CT: 25.81	
G			2107 A01 10m saki2<br>U pGEM<br>2.78E4<br>FAM-NFQ-MGB<br>CT: 26.07	2107 A01 10m saki2<br>U pGEM<br>2.88E4<br>FAM-NFQ-MGB<br>CT: 26.02	2107 A01 10m saki2<br>U pGEM<br>2.58E4<br>FAM-NFQ-MGB<br>CT: 26.19	2107 A04 3000m saki<br>U pGEM<br>3.03E4<br>FAM-NFQ-MGB<br>CT: 25.95	2107 A04 3000m saki<br>U pGEM<br>3E4<br>FAM-NFQ-MGB<br>CT: 25.96	2107 A04 3000m saki<br>U pGEM<br>3.33E4<br>FAM-NFQ-MGB<br>CT: 25.8	2107 A07 3000m saki<br>U pGEM<br>2.71E4<br>FAM-NFQ-MGB<br>CT: 26.12	2107 A07 3000m saki<br>U pGEM<br>3.09E4<br>FAM-NFQ-MGB<br>CT: 25.92	2107 A07 3000m saki<br>U pGEM<br>2.8E4<br>FAM-NFQ-MGB<br>CT: 26.06	N pGEM<br>FAM-NFQ-MGB<br>CT: Undetermined
H	Comf2E4<br>U pGEM<br>2.03E4<br>FAM-NFQ-MGB<br>CT: 26.55	Comf2E4<br>U pGEM<br>2.06E4<br>FAM-NFQ-MGB<br>CT: 26.53	2107 A01 10m ato2<br>U pGEM<br>2.54E4<br>FAM-NFQ-MGB<br>CT: 26.21	2107 A01 10m ato2<br>U pGEM<br>2.42E4<br>FAM-NFQ-MGB<br>CT: 26.28	2107 A01 10m ato2<br>U pGEM<br>2.58E4<br>FAM-NFQ-MGB<br>CT: 26.19	2107 A04 3000m ato<br>U pGEM<br>3.52E4<br>FAM-NFQ-MGB<br>CT: 25.72	2107 A04 3000m ato<br>U pGEM<br>3.83E4<br>FAM-NFQ-MGB<br>CT: 25.6	2107 A04 3000m ato<br>U pGEM<br>3.88E4<br>FAM-NFQ-MGB<br>CT: 25.58	2107 A07 3000m ato<br>U pGEM<br>4.6E4<br>FAM-NFQ-MGB<br>CT: 25.32	2107 A07 3000m ato<br>U pGEM<br>3.39E4<br>FAM-NFQ-MGB<br>CT: 25.78	2107 A07 3000m ato<br>U pGEM<br>4.22E4<br>FAM-NFQ-MGB<br>CT: 25.45	N pGEM<br>FAM-NFQ-MGB<br>CT: Undetermined

Standard Curves

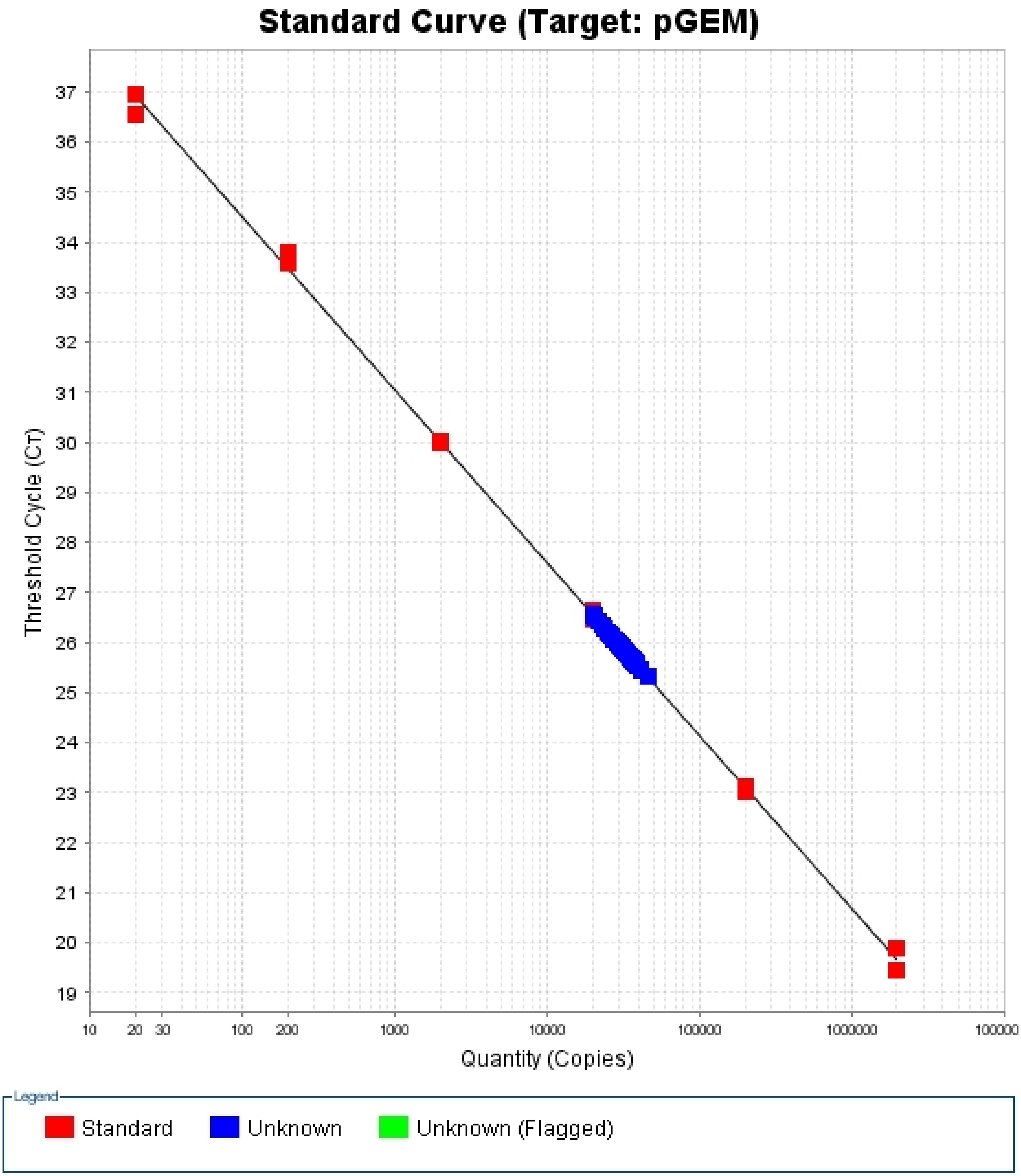

slope:-3.45 Y-Intercept:41.409 R^2:0.999 Eff%:94.904

Results Table

Well	Sample	Target	Task	Quantity	C
A1		pGEM	STANDARD	2000000	19.8908
A2		pGEM	STANDARD	2000000	19.4723
A3	2107 A01 10m	pGEM	UNKNOWN	22982.4453	26.3605
A4	2107 A01 10m	pGEM	UNKNOWN	22979.9297	26.3607
A5	2107 A01 10m	pGEM	UNKNOWN	26374.7109	26.1542
A6	2107 A04 10m	pGEM	UNKNOWN	20136.4219	26.5586
A7	2107 A04 10m	pGEM	UNKNOWN	26998.0586	26.1192
A8	2107 A04 10m	pGEM	UNKNOWN	20736.1172	26.5147
A9	2107 A07 SCM	pGEM	UNKNOWN	20330.709	26.5443
A10	2107 A07 SCM	pGEM	UNKNOWN	22059.1426	26.422
A11	2107 A07 SCM	pGEM	UNKNOWN	21066.2383	26.491
B1		pGEM	STANDARD	200000	23.1119
B2		pGEM	STANDARD	200000	23.0119
B3	2107 A01 10m	pGEM	UNKNOWN	34879.9258	25.7354
B4	2107 A01 10m	pGEM	UNKNOWN	38992.4102	25.5684
B5	2107 A01 10m	pGEM	UNKNOWN	35331.4531	25.7161
B6	2107 A04 10m	pGEM	UNKNOWN	36703.043	25.6591
B7	2107 A04 10m	pGEM	UNKNOWN	41372.9375	25.4796
B8	2107 A04 10m	pGEM	UNKNOWN	36576.2852	25.6642
B9	2107 A07 SCM	pGEM	UNKNOWN	35340	25.7158
B10	2107 A07 SCM	pGEM	UNKNOWN	34525.8516	25.7507
B11	2107 A07 SCM	pGEM	UNKNOWN	38193.0195	25.5994
C1		pGEM	STANDARD	20000	26.658
C2		pGEM	STANDARD	20000	26.457
C3	2107 A01 SCM	pGEM	UNKNOWN	29456.377	25.9886
C4	2107 A01 SCM	pGEM	UNKNOWN	35384.0195	25.7139
C5	2107 A01 SCM	pGEM	UNKNOWN	27881.1309	26.071
C6	2107 A04 200m	pGEM	UNKNOWN	29576.5254	25.9825
C7	2107 A04 200m	pGEM	UNKNOWN	29846.166	25.9689
C8	2107 A04 200m	pGEM	UNKNOWN	31201.9316	25.9024

Well		Sample	Target	Task	Quantity	C
C9		2107 A07 200m	pGEM	UNKNOWN	23962.1367	26.298
C10		2107 A07 200m	pGEM	UNKNOWN	32786.5781	25.8281
C11		2107 A07 200m	pGEM	UNKNOWN	31380.0059	25.8938
D1			pGEM	STANDARD	2000	29.9756
D2			pGEM	STANDARD	2000	30.0185
D3		2107 A01 SCM	pGEM	UNKNOWN	33255.2539	25.8069
D4		2107 A01 SCM	pGEM	UNKNOWN	38591.8242	25.5839
D5		2107 A01 SCM	pGEM	UNKNOWN	35779.7617	25.6972
D6		2107 A04 200m	pGEM	UNKNOWN	33390.2617	25.8008
D7		2107 A04 200m	pGEM	UNKNOWN	39651.7188	25.5433
D8		2107 A04 200m	pGEM	UNKNOWN	35439.4609	25.7115
D9		2107 A07 200m	pGEM	UNKNOWN	37337.6602	25.6334
D10		2107 A07 200m	pGEM	UNKNOWN	36515.3477	25.6667
D11		2107 A07 200m	pGEM	UNKNOWN	36251.6562	25.6776
E1			pGEM	STANDARD	200	33.8194
E2			pGEM	STANDARD	200	33.5891
E3		2107 A01 BTM	pGEM	UNKNOWN	36509.3984	25.667
E4		2107 A01 BTM	pGEM	UNKNOWN	37964.9258	25.6084
E5		2107 A01 BTM	pGEM	UNKNOWN	35952.0508	25.69
E6		2107 A04	pGEM	UNKNOWN	32491.7383	25.8417
E7		2107 A04	pGEM	UNKNOWN	34932.7539	25.7331
E8		2107 A04	pGEM	UNKNOWN	33565.8672	25.7929
E9		2107 A07	pGEM	UNKNOWN	32468.793	25.8427
E10		2107 A07	pGEM	UNKNOWN	31459.5918	25.8901
E11		2107 A07	pGEM	UNKNOWN	30016.6543	25.9604
F1			pGEM	STANDARD	20	36.961
F2			pGEM	STANDARD	20	36.5616
F3		2107 A01 BTM	pGEM	UNKNOWN	32560.1738	25.8385
F4		2107 A01 BTM	pGEM	UNKNOWN	33364.9805	25.8019
F5		2107 A01 BTM	pGEM	UNKNOWN	33707.5391	25.7866
F6		2107 A04	pGEM	UNKNOWN	32596.291	25.8369
F7		2107 A04	pGEM	UNKNOWN	41567.4531	25.4726

Well		Sample	Target	Task	Quantity	C
F8		2107 A04	pGEM	UNKNOWN	36812.9023	25.6546
F9		2107 A07	pGEM	UNKNOWN	33388.1367	25.8009
F10		2107 A07	pGEM	UNKNOWN	35237.543	25.7201
F11		2107 A07	pGEM	UNKNOWN	33282.9922	25.8056
G3		2107 A01 10m	pGEM	UNKNOWN	27834.5742	26.0735
G4		2107 A01 10m	pGEM	UNKNOWN	28807.0176	26.022
G5		2107 A01 10m	pGEM	UNKNOWN	25832.123	26.1854
G6		2107 A04	pGEM	UNKNOWN	30313.7578	25.9457
G7		2107 A04	pGEM	UNKNOWN	29985.6465	25.962
G8		2107 A04	pGEM	UNKNOWN	33327.6719	25.8036
G9		2107 A07	pGEM	UNKNOWN	27053.2695	26.1162
G10		2107 A07	pGEM	UNKNOWN	30940.043	25.915
G11		2107 A07	pGEM	UNKNOWN	28028.084	26.0631
G12			pGEM	NTC		Undetermined
H1		Comf2E4	pGEM	UNKNOWN	20270.2715	26.5487
H2		Comf2E4	pGEM	UNKNOWN	20571.1797	26.5266
H3		2107 A01 10m	pGEM	UNKNOWN	25363.959	26.2128
H4		2107 A01 10m	pGEM	UNKNOWN	24237.4766	26.2809
H5		2107 A01 10m	pGEM	UNKNOWN	25813.6504	26.1865
H6		2107 A04	pGEM	UNKNOWN	35248.5781	25.7196
H7		2107 A04	pGEM	UNKNOWN	38259.1406	25.5968
H8		2107 A04	pGEM	UNKNOWN	38811.2344	25.5754
H9		2107 A07	pGEM	UNKNOWN	45975.6406	25.3215
H10		2107 A07	pGEM	UNKNOWN	33927.1484	25.7769
H11		2107 A07	pGEM	UNKNOWN	42234.2109	25.4487
H12			pGEM	NTC		Undetermined

QC Summary

Total Wells	96	Processed Wells	88	Targets Used	1
Well Setup	88	Flagged Wells	0	Samples Used	25

Flag	Name	Frequency	Locations
AMPNC	Amplification in negative control	0	
BADROX	Bad passive reference signal	0	
BLFAIL	Baseline algorithm failed	0	
CTFAIL	C algorithm failed	0	
EXPFAIL	Exponential algorithm failed	0	
HIGHSD	High standard deviation in replicate group	0	
NOAMP	No amplification	0	
NOISE	Noise higher than others in plate	0	
NOSIGNAL	No signal in well	0	
OFFSCALE	Fluorescence is offscale	0	
OUTLIERRG	Outlier in replicate group	0	
SPIKE	Noise spikes	0	
THOLDFAIL	Thresholding algorithm failed	0	
