# Supplementary material for: Ecological impact of algal-preying protists aplanochytrids (Labyrinthulea) as a source of docosahexaenoic acid for copepods in marine environments
Source: ISME Commun. 2026 Jun 26;6(1):ycag178. doi: 10.1093/ismeco/ycag178 (PMC13685785; doi:10.1093/ismeco/ycag178)
Supplement: Supplementary file 2 [file supplementary_materials_ycag178.zip › Morimoto_ISME_C_3rdrev_supplemental_data.zip!/Morimoto_ISME_C_3rdrev_supplemental_data/Supplemental_Data_2/Standard Curve/DNA recovery/2107/DnaRec2107AlineA01-A07N=3.pdf]

Experiment Results Report

230720DnaRec2107AlineA01-A07N=3

Experiment Summary

Experiment Name	: 230720DnaRec2107AlineA01-A07N=3
Experiment Type	: Quantitation - Standard Curve
File Name	: 230720DnaRec2107AlineA01-A07N=3.eds
Run Started	: 2023 Jul 20 11:47:58 AM
Run Finished	: 2023 Jul 20 1:27:13 PM
Run Duration	: 99 minutes 14 seconds
Date Modified	: 2023 Jul 20 2:00:10 PM
User	:
Number of wells used	: 86
Number of wells with results	: 86
Instrument Name	:
Instrument Type	: Applied Biosystems StepOnePlus™ Instrument
Comments	:

Results Summary

Sample	Target	Quantity (Mean)	Quantity (Std Dev)	C (Mean)	C (Std Dev)
2107 A01 10m ato	pGEM	27673.26	384.53	25.56	0.02
2107 A01 10m saki	pGEM	11508.39	343.39	26.88	0.05
2107 A01 BTM ato	pGEM	24137.03	1511.61	25.76	0.10
2107 A01 BTM saki	pGEM	13434.25	371.49	26.64	0.04
2107 A01 SCM ato	pGEM	25648.31	515.50	25.67	0.03
2107 A01 SCM saki	pGEM	15766.50	989.49	26.40	0.09
2107 A04 1000m ato	pGEM	23662.52	1817.52	25.79	0.12
2107 A04 1000m	pGEM	11994.96	413.96	26.82	0.05
2107 A04 10m ato	pGEM	27052.06	1127.90	25.59	0.06
2107 A04 10m saki	pGEM	8951.25	241.00	27.26	0.04
2107 A04 200m ato	pGEM	27221.68	1079.10	25.58	0.06
2107 A04 200m saki	pGEM	12643.04	961.10	26.74	0.12
2107 A04 3000m ato	pGEM	21477.59	1924.60	25.94	0.13
2107 A04 3000m	pGEM	9109.55	1722.45	27.25	0.27
2107 A04 SCM ato	pGEM	28781.68	1305.54	25.50	0.07
2107 A04 SCM saki	pGEM	11266.83	861.60	26.91	0.12
2107 A07 1000m ato	pGEM	24705.65	2923.83	25.73	0.18
2107 A07 1000m	pGEM	15603.51	975.36	26.42	0.10
2107 A07 200m ato	pGEM	24332.77	916.39	25.75	0.06
2107 A07 200m saki	pGEM	14669.97	1873.22	26.52	0.20
2107 A07 3000m ato	pGEM	27235.14	704.64	25.58	0.04
2107 A07 3000m	pGEM	12409.48	547.88	26.76	0.07
2107 A07 SCM ato	pGEM	27001.66	1231.26	25.59	0.07
2107 A07 SCM saki	pGEM	13184.72	338.84	26.67	0.04

Plate Layout

	1	2	3	4	5	6	7	8	9	10	11	12
A	S pGEM<br>2E6<br>FAM-NFQ-MGB<br>CT: 19.05	S pGEM<br>2E6<br>FAM-NFQ-MGB<br>CT: 19.11	2107 A01 10m saki<br>U pGEM<br>1.16E4<br>FAM-NFQ-MGB<br>CT: 26.86	2107 A01 10m saki<br>U pGEM<br>1.18E4<br>FAM-NFQ-MGB<br>CT: 26.84	2107 A01 10m saki<br>U pGEM<br>1.11E4<br>FAM-NFQ-MGB<br>CT: 26.93	2107 A04 SCM saki<br>U pGEM<br>1.2E4<br>FAM-NFQ-MGB<br>CT: 26.82	2107 A04 SCM saki<br>U pGEM<br>1.03E4<br>FAM-NFQ-MGB<br>CT: 27.04	2107 A04 SCM saki<br>U pGEM<br>1.15E4<br>FAM-NFQ-MGB<br>CT: 26.88	2107 A07 SCM saki<br>U pGEM<br>1.33E4<br>FAM-NFQ-MGB<br>CT: 26.66	2107 A07 SCM saki<br>U pGEM<br>1.28E4<br>FAM-NFQ-MGB<br>CT: 26.72	2107 A07 SCM saki<br>U pGEM<br>1.35E4<br>FAM-NFQ-MGB<br>CT: 26.64	
B	S pGEM<br>2E5<br>FAM-NFQ-MGB<br>CT: 22.65	S pGEM<br>2E5<br>FAM-NFQ-MGB<br>CT: 22.44	2107 A01 10m ato<br>U pGEM<br>2.76E4<br>FAM-NFQ-MGB<br>CT: 25.56	2107 A01 10m ato<br>U pGEM<br>2.74E4<br>FAM-NFQ-MGB<br>CT: 25.57	2107 A01 10m ato<br>U pGEM<br>2.81E4<br>FAM-NFQ-MGB<br>CT: 25.53	2107 A04 SCM ato<br>U pGEM<br>2.84E4<br>FAM-NFQ-MGB<br>CT: 25.52	2107 A04 SCM ato<br>U pGEM<br>3.02E4<br>FAM-NFQ-MGB<br>CT: 25.42	2107 A04 SCM ato<br>U pGEM<br>2.77E4<br>FAM-NFQ-MGB<br>CT: 25.55	2107 A07 SCM ato<br>U pGEM<br>2.83E4<br>FAM-NFQ-MGB<br>CT: 25.52	2107 A07 SCM ato<br>U pGEM<br>2.68E4<br>FAM-NFQ-MGB<br>CT: 25.6	2107 A07 SCM ato<br>U pGEM<br>2.59E4<br>FAM-NFQ-MGB<br>CT: 25.66	
C	S pGEM<br>2E4<br>FAM-NFQ-MGB<br>CT: 26.03	S pGEM<br>2E4<br>FAM-NFQ-MGB<br>CT: 26.04	2107 A01 SCM saki<br>U pGEM<br>1.5E4<br>FAM-NFQ-MGB<br>CT: 26.48	2107 A01 SCM saki<br>U pGEM<br>1.69E4<br>FAM-NFQ-MGB<br>CT: 26.3	2107 A01 SCM saki<br>U pGEM<br>1.54E4<br>FAM-NFQ-MGB<br>CT: 26.44	2107 A04 200m saki<br>U pGEM<br>1.35E4<br>FAM-NFQ-MGB<br>CT: 26.63	2107 A04 200m saki<br>U pGEM<br>1.28E4<br>FAM-NFQ-MGB<br>CT: 26.72	2107 A04 200m saki<br>U pGEM<br>1.16E4<br>FAM-NFQ-MGB<br>CT: 26.86	2107 A07 200m saki<br>U pGEM<br>1.26E4<br>FAM-NFQ-MGB<br>CT: 26.74	2107 A07 200m saki<br>U pGEM<br>1.62E4<br>FAM-NFQ-MGB<br>CT: 26.36	2107 A07 200m saki<br>U pGEM<br>1.52E4<br>FAM-NFQ-MGB<br>CT: 26.46	
D	S pGEM<br>2E3<br>FAM-NFQ-MGB<br>CT: 29.57	S pGEM<br>2E3<br>FAM-NFQ-MGB<br>CT: 29.6	2107 A01 SCM ato<br>U pGEM<br>2.51E4<br>FAM-NFQ-MGB<br>CT: 25.7	2107 A01 SCM ato<br>U pGEM<br>2.6E4<br>FAM-NFQ-MGB<br>CT: 25.65	2107 A01 SCM ato<br>U pGEM<br>2.58E4<br>FAM-NFQ-MGB<br>CT: 25.66	2107 A04 200m ato<br>U pGEM<br>2.6E4<br>FAM-NFQ-MGB<br>CT: 25.65	2107 A04 200m ato<br>U pGEM<br>2.82E4<br>FAM-NFQ-MGB<br>CT: 25.53	2107 A04 200m ato<br>U pGEM<br>2.74E4<br>FAM-NFQ-MGB<br>CT: 25.57	2107 A07 200m ato<br>U pGEM<br>2.54E4<br>FAM-NFQ-MGB<br>CT: 25.69	2107 A07 200m ato<br>U pGEM<br>2.37E4<br>FAM-NFQ-MGB<br>CT: 25.79	2107 A07 200m ato<br>U pGEM<br>2.39E4<br>FAM-NFQ-MGB<br>CT: 25.77	
E	S pGEM<br>200<br>FAM-NFQ-MGB<br>CT: 33.06	S pGEM<br>200<br>FAM-NFQ-MGB<br>CT: 33.28	2107 A01 BTM saki<br>U pGEM<br>1.37E4<br>FAM-NFQ-MGB<br>CT: 26.61	2107 A01 BTM saki<br>U pGEM<br>1.35E4<br>FAM-NFQ-MGB<br>CT: 26.63	2107 A01 BTM saki<br>U pGEM<br>1.3E4<br>FAM-NFQ-MGB<br>CT: 26.69	2107 A04 1000m saki<br>U pGEM<br>1.22E4<br>FAM-NFQ-MGB<br>CT: 26.79	2107 A04 1000m saki<br>U pGEM<br>1.23E4<br>FAM-NFQ-MGB<br>CT: 26.78	2107 A04 1000m saki<br>U pGEM<br>1.15E4<br>FAM-NFQ-MGB<br>CT: 26.87	2107 A07 1000m saki<br>U pGEM<br>1.64E4<br>FAM-NFQ-MGB<br>CT: 26.35	2107 A07 1000m saki<br>U pGEM<br>1.59E4<br>FAM-NFQ-MGB<br>CT: 26.39	2107 A07 1000m saki<br>U pGEM<br>1.45E4<br>FAM-NFQ-MGB<br>CT: 26.53	
F	S pGEM<br>20<br>FAM-NFQ-MGB<br>CT: 35.98	S pGEM<br>20<br>FAM-NFQ-MGB<br>CT: 36.55	2107 A01 BTM ato<br>U pGEM<br>2.46E4<br>FAM-NFQ-MGB<br>CT: 25.73	2107 A01 BTM ato<br>U pGEM<br>2.25E4<br>FAM-NFQ-MGB<br>CT: 25.87	2107 A01 BTM ato<br>U pGEM<br>2.54E4<br>FAM-NFQ-MGB<br>CT: 25.69	2107 A04 1000m ato<br>U pGEM<br>2.19E4<br>FAM-NFQ-MGB<br>CT: 25.91	2107 A04 1000m ato<br>U pGEM<br>2.55E4<br>FAM-NFQ-MGB<br>CT: 25.68	2107 A04 1000m ato<br>U pGEM<br>2.36E4<br>FAM-NFQ-MGB<br>CT: 25.8	2107 A07 1000m ato<br>U pGEM<br>2.75E4<br>FAM-NFQ-MGB<br>CT: 25.57	2107 A07 1000m ato<br>U pGEM<br>2.17E4<br>FAM-NFQ-MGB<br>CT: 25.93	2107 A07 1000m ato<br>U pGEM<br>2.5E4<br>FAM-NFQ-MGB<br>CT: 25.71	
G			2107 A04 10m saki<br>U pGEM<br>8.8E3<br>FAM-NFQ-MGB<br>CT: 27.28	2107 A04 10m saki<br>U pGEM<br>9.23E3<br>FAM-NFQ-MGB<br>CT: 27.21	2107 A04 10m saki<br>U pGEM<br>8.83E3<br>FAM-NFQ-MGB<br>CT: 27.28	2107 A04 3000m saki<br>U pGEM<br>1.11E4<br>FAM-NFQ-MGB<br>CT: 26.93	2107 A04 3000m saki<br>U pGEM<br>7.89E3<br>FAM-NFQ-MGB<br>CT: 27.45	2107 A04 3000m saki<br>U pGEM<br>8.36E3<br>FAM-NFQ-MGB<br>CT: 27.36	2107 A07 3000m saki<br>U pGEM<br>1.2E4<br>FAM-NFQ-MGB<br>CT: 26.82	2107 A07 3000m saki<br>U pGEM<br>1.3E4<br>FAM-NFQ-MGB<br>CT: 26.69	2107 A07 3000m saki<br>U pGEM<br>1.22E4<br>FAM-NFQ-MGB<br>CT: 26.79	
H	N pGEM<br>FAM-NFQ-MGB<br>CT: Undetermined	N pGEM<br>FAM-NFQ-MGB<br>CT: Undetermined	2107 A04 10m ato<br>U pGEM<br>2.63E4<br>FAM-NFQ-MGB<br>CT: 25.63	2107 A04 10m ato<br>U pGEM<br>2.65E4<br>FAM-NFQ-MGB<br>CT: 25.62	2107 A04 10m ato<br>U pGEM<br>2.83E4<br>FAM-NFQ-MGB<br>CT: 25.52	2107 A04 3000m ato<br>U pGEM<br>2.35E4<br>FAM-NFQ-MGB<br>CT: 25.8	2107 A04 3000m ato<br>U pGEM<br>2.13E4<br>FAM-NFQ-MGB<br>CT: 25.95	2107 A04 3000m ato<br>U pGEM<br>1.97E4<br>FAM-NFQ-MGB<br>CT: 26.07	2107 A07 3000m ato<br>U pGEM<br>2.64E4<br>FAM-NFQ-MGB<br>CT: 25.63	2107 A07 3000m ato<br>U pGEM<br>2.76E4<br>FAM-NFQ-MGB<br>CT: 25.56	2107 A07 3000m ato<br>U pGEM<br>2.76E4<br>FAM-NFQ-MGB<br>CT: 25.56	

Standard Curves

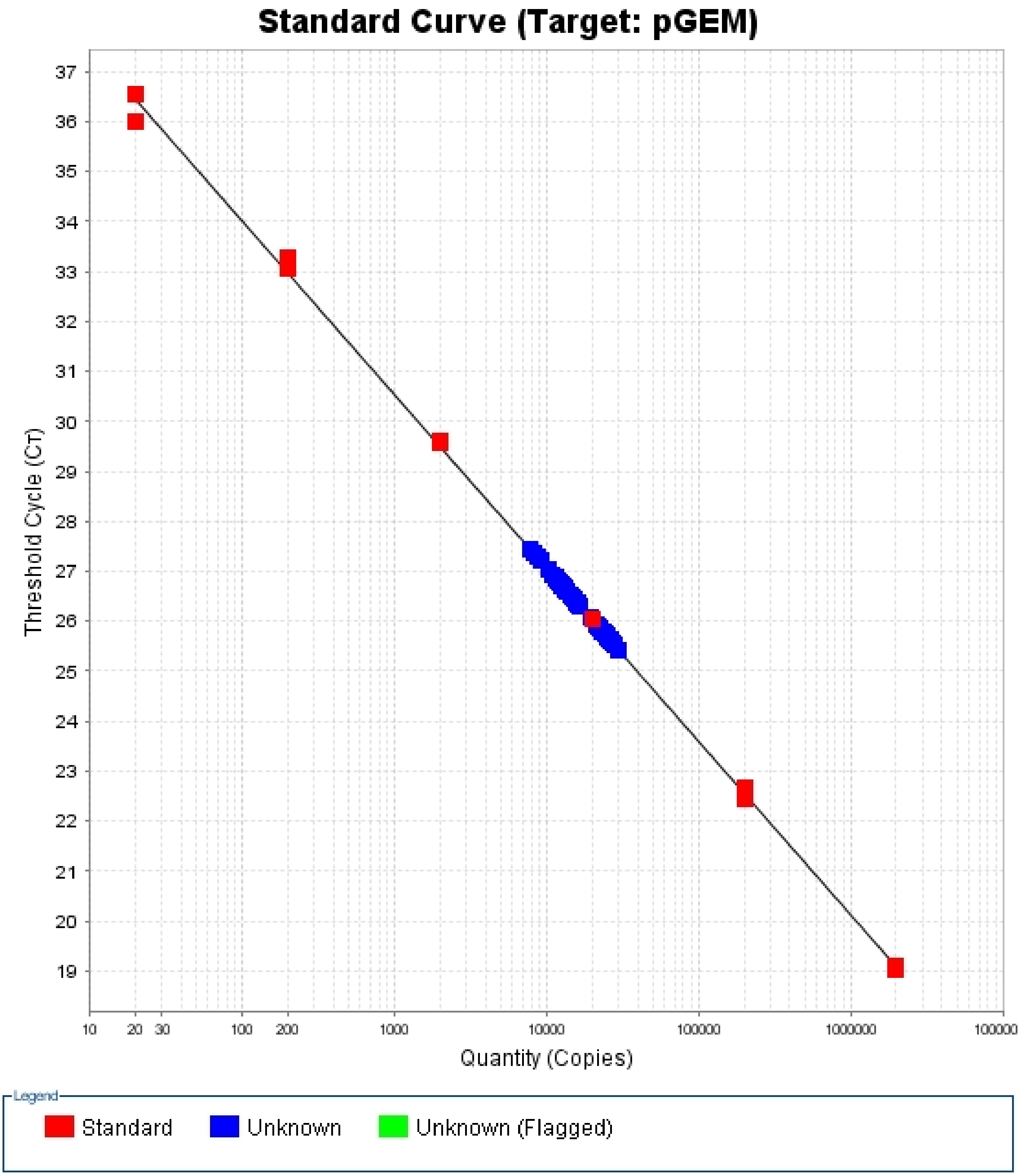

slope:-3.467 Y-Intercept:40.958 R^2:0.999 Eff%:94.271

Results Table

Well		Sample	Target	Task	Quantity	C
A1			pGEM	STANDARD	2000000	19.0489
A2			pGEM	STANDARD	2000000	19.107
A3		2107 A01 10m	pGEM	UNKNOWN	11625.5088	26.8616
A4		2107 A01 10m	pGEM	UNKNOWN	11777.8906	26.842
A5		2107 A01 10m	pGEM	UNKNOWN	11121.7588	26.9283
A6		2107 A04 SCM	pGEM	UNKNOWN	11986.5762	26.8155
A7		2107 A04 SCM	pGEM	UNKNOWN	10312.124	27.0421
A8		2107 A04 SCM	pGEM	UNKNOWN	11501.7871	26.8777
A9		2107 A07 SCM	pGEM	UNKNOWN	13278.4482	26.6614
A10		2107 A07 SCM	pGEM	UNKNOWN	12808.8867	26.7156
A11		2107 A07 SCM	pGEM	UNKNOWN	13466.834	26.6402
B1			pGEM	STANDARD	200000	22.6513
B2			pGEM	STANDARD	200000	22.4367
B3		2107 A01 10m	pGEM	UNKNOWN	27556.6289	25.562
B4		2107 A01 10m	pGEM	UNKNOWN	27360.5391	25.5728
B5		2107 A01 10m	pGEM	UNKNOWN	28102.6016	25.5325
B6		2107 A04 SCM	pGEM	UNKNOWN	28360.5312	25.5187
B7		2107 A04 SCM	pGEM	UNKNOWN	30245.8164	25.4218
B8		2107 A04 SCM	pGEM	UNKNOWN	27738.6953	25.5521
B9		2107 A07 SCM	pGEM	UNKNOWN	28325.2422	25.5206
B10		2107 A07 SCM	pGEM	UNKNOWN	26789.4219	25.6045
B11		2107 A07 SCM	pGEM	UNKNOWN	25890.3047	25.6559
C1			pGEM	STANDARD	20000	26.0269
C2			pGEM	STANDARD	20000	26.0396
C3		2107 A01 SCM	pGEM	UNKNOWN	14998.8818	26.478
C4		2107 A01 SCM	pGEM	UNKNOWN	16883.2266	26.2997
C5		2107 A01 SCM	pGEM	UNKNOWN	15417.4082	26.4365
C6		2107 A04 200m	pGEM	UNKNOWN	13539.8848	26.6321
C7		2107 A04 200m	pGEM	UNKNOWN	12760.71	26.7213
C8		2107 A04 200m	pGEM	UNKNOWN	11628.5137	26.8612

Well		Sample	Target	Task	Quantity	C
C9		2107 A07 200m	pGEM	UNKNOWN	12600.1289	26.7404
C10		2107 A07 200m	pGEM	UNKNOWN	16248.752	26.3574
C11		2107 A07 200m	pGEM	UNKNOWN	15161.0283	26.4618
D1			pGEM	STANDARD	2000	29.5656
D2			pGEM	STANDARD	2000	29.5968
D3		2107 A01 SCM	pGEM	UNKNOWN	25065.3594	25.7047
D4		2107 A01 SCM	pGEM	UNKNOWN	26044.0371	25.647
D5		2107 A01 SCM	pGEM	UNKNOWN	25835.5312	25.6591
D6		2107 A04 200m	pGEM	UNKNOWN	26048.5234	25.6468
D7		2107 A04 200m	pGEM	UNKNOWN	28171.8848	25.5288
D8		2107 A04 200m	pGEM	UNKNOWN	27444.6387	25.5681
D9		2107 A07 200m	pGEM	UNKNOWN	25380.2383	25.6859
D10		2107 A07 200m	pGEM	UNKNOWN	23679.1348	25.7904
D11		2107 A07 200m	pGEM	UNKNOWN	23938.9395	25.7739
E1			pGEM	STANDARD	200	33.0572
E2			pGEM	STANDARD	200	33.2808
E3		2107 A01 BTM	pGEM	UNKNOWN	13743.6719	26.6096
E4		2107 A01 BTM	pGEM	UNKNOWN	13536.832	26.6324
E5		2107 A01 BTM	pGEM	UNKNOWN	13022.2412	26.6908
E6		2107 A04	pGEM	UNKNOWN	12151.3379	26.795
E7		2107 A04	pGEM	UNKNOWN	12307.9609	26.7757
E8		2107 A04	pGEM	UNKNOWN	11525.5879	26.8746
E9		2107 A07	pGEM	UNKNOWN	16368.335	26.3464
E10		2107 A07	pGEM	UNKNOWN	15937.0703	26.3866
E11		2107 A07	pGEM	UNKNOWN	14505.125	26.5284
F1			pGEM	STANDARD	20	35.9803
F2			pGEM	STANDARD	20	36.5486
F3		2107 A01 BTM	pGEM	UNKNOWN	24568.8066	25.7348
F4		2107 A01 BTM	pGEM	UNKNOWN	22456.502	25.8702
F5		2107 A01 BTM	pGEM	UNKNOWN	25385.7676	25.6856
F6		2107 A04	pGEM	UNKNOWN	21888.5469	25.9088
F7		2107 A04	pGEM	UNKNOWN	25520.6602	25.6776

Well		Sample	Target	Task	Quantity	C
F8		2107 A04	pGEM	UNKNOWN	23578.3633	25.7968
F9		2107 A07	pGEM	UNKNOWN	27480.3984	25.5662
F10		2107 A07	pGEM	UNKNOWN	21652.6406	25.9251
F11		2107 A07	pGEM	UNKNOWN	24983.8984	25.7096
G3		2107 A04 10m	pGEM	UNKNOWN	8799.4541	27.281
G4		2107 A04 10m	pGEM	UNKNOWN	9229.1426	27.2092
G5		2107 A04 10m	pGEM	UNKNOWN	8825.1709	27.2766
G6		2107 A04	pGEM	UNKNOWN	11080.0117	26.934
G7		2107 A04	pGEM	UNKNOWN	7890.2402	27.4452
G8		2107 A04	pGEM	UNKNOWN	8358.4062	27.3584
G9		2107 A07	pGEM	UNKNOWN	11973.1162	26.8172
G10		2107 A07	pGEM	UNKNOWN	13024.3525	26.6905
G11		2107 A07	pGEM	UNKNOWN	12230.9707	26.7851
H1			pGEM	NTC		Undetermined
H2			pGEM	NTC		Undetermined
H3		2107 A04 10m	pGEM	UNKNOWN	26298.4258	25.6324
H4		2107 A04 10m	pGEM	UNKNOWN	26508.9883	25.6204
H5		2107 A04 10m	pGEM	UNKNOWN	28348.752	25.5193
H6		2107 A04	pGEM	UNKNOWN	23492.1797	25.8023
H7		2107 A04	pGEM	UNKNOWN	21282.8125	25.951
H8		2107 A04	pGEM	UNKNOWN	19657.7891	26.0706
H9		2107 A07	pGEM	UNKNOWN	26421.4961	25.6253
H10		2107 A07	pGEM	UNKNOWN	27641.6816	25.5574
H11		2107 A07	pGEM	UNKNOWN	27642.2422	25.5573

QC Summary

Total Wells	96	Processed Wells	86	Targets Used	1
Well Setup	86	Flagged Wells	0	Samples Used	24

Flag	Name	Frequency	Locations
AMPNC	Amplification in negative control	0	
BADROX	Bad passive reference signal	0	
BLFAIL	Baseline algorithm failed	0	
CTFAIL	C algorithm failed	0	
EXPFAIL	Exponential algorithm failed	0	
HIGHSD	High standard deviation in replicate group	0	
NOAMP	No amplification	0	
NOISE	Noise higher than others in plate	0	
NOSIGNAL	No signal in well	0	
OFFSCALE	Fluorescence is offscale	0	
OUTLIERRG	Outlier in replicate group	0	
SPIKE	Noise spikes	0	
THOLDFAIL	Thresholding algorithm failed	0	
