# Supplementary material for: Ecological impact of algal-preying protists aplanochytrids (Labyrinthulea) as a source of docosahexaenoic acid for copepods in marine environments
Source: ISME Commun. 2026 Jun 26;6(1):ycag178. doi: 10.1093/ismeco/ycag178 (PMC13685785; doi:10.1093/ismeco/ycag178)
Supplement: Supplementary file 2 [file supplementary_materials_ycag178.zip › Morimoto_ISME_C_3rdrev_supplemental_data.zip!/Morimoto_ISME_C_3rdrev_supplemental_data/Supplemental_Data_2/Standard Curve/DNA recovery/2107/DnaRec2107AlineA11-A21N=1.pdf]

Experiment Results Report

230714DnaRec2107AlineA11-A21N=1

Experiment Summary

Experiment Name	:	230714DnaRec2107AlineA11-A21N=1
Experiment Type	:	Quantitation - Standard Curve
File Name	:	230714DnaRec2107AlineA11-A21N=1.eds
Run Started	:	2023 Jul 14 6:28:52 PM
Run Finished	:	2023 Jul 14 8:03:28 PM
Run Duration	:	94 minutes 35 seconds
Date Modified	:	2023 Jul 14 9:50:25 PM
User	:	
Number of wells used	:	76
Number of wells with results	:	76
Instrument Name	:	
Instrument Type	:	Applied Biosystems StepOnePlus™ Instrument
Comments	:	

Results Summary

Sample	Target	Quantity (Mean)	Quantity (Std Dev)	C (Mean)	C (Std Dev)
2107 A11 1000m ato	pGEM	43240.02	1481.22	24.92	0.05
2107 A11 1000m	pGEM	20586.19	1037.31	26.03	0.07
2107 A11 10m ato	pGEM	40836.09	766.54	25.01	0.03
2107 A11 10m saki	pGEM	22024.24	771.80	25.92	0.05
2107 A11 200m ato	pGEM	42132.56	2852.14	24.96	0.10
2107 A11 200m saki	pGEM	28533.41	522.06	25.54	0.03
2107 A11 3000m ato	pGEM	42494.20	1649.34	24.95	0.06
2107 A11 3000m	pGEM	14431.95	620.27	26.55	0.06
2107 A11 SCM ato	pGEM	39338.55	4817.05	25.07	0.18
2107 A11 SCM saki	pGEM	22236.19	1031.99	25.91	0.07
2107 A21 1000m ato	pGEM	43015.82	1117.42	24.93	0.04
2107 A21 1000m	pGEM	10110.69	364.40	27.08	0.05
2107 A21 10m ato	pGEM	42154.97	2810.86	24.96	0.10
2107 A21 10m saki	pGEM	14938.04	1125.89	26.50	0.11
2107 A21 200m ato	pGEM	46933.66	5952.75	24.81	0.19
2107 A21 200m saki	pGEM	11540.80	858.18	26.89	0.11
2107 A21 3000m ato	pGEM	34979.61	4536.85	25.24	0.19
2107 A21 3000m	pGEM	13790.61	945.22	26.62	0.10
2107 A21 SCM ato	pGEM	46408.72	4215.81	24.82	0.13
2107 A21 SCM saki	pGEM	13089.98	361.89	26.70	0.04
Comf2E4	pGEM	21652.33	1857.59	25.95	0.13

Plate Layout

	1	2	3	4	5	6	7	8	9	10	11	12
A	S pGEM<br>2E6<br>FAM-NFQ-MGB<br>CT: 19.52	S pGEM<br>2E6<br>FAM-NFQ-MGB<br>CT: 19.04	2107 A11 10m saki<br>U pGEM<br>2.27E4<br>FAM-NFQ-MGB<br>CT: 25.88	2107 A11 10m saki<br>U pGEM<br>2.12E4<br>FAM-NFQ-MGB<br>CT: 25.98	2107 A11 10m saki<br>U pGEM<br>2.22E4<br>FAM-NFQ-MGB<br>CT: 25.91	2107 A21 10m saki<br>U pGEM<br>1.37E4<br>FAM-NFQ-MGB<br>CT: 26.63	2107 A21 10m saki<br>U pGEM<br>1.6E4<br>FAM-NFQ-MGB<br>CT: 26.4	2107 A21 10m saki<br>U pGEM<br>1.51E4<br>FAM-NFQ-MGB<br>CT: 26.48	2107 A11 3000m saki<br>U pGEM<br>1.44E4<br>FAM-NFQ-MGB<br>CT: 26.55	2107 A21 3000m saki<br>U pGEM<br>1.27E4<br>FAM-NFQ-MGB<br>CT: 26.74		
B	S pGEM<br>2E5<br>FAM-NFQ-MGB<br>CT: 22.64	S pGEM<br>2E5<br>FAM-NFQ-MGB<br>CT: 22.43	2107 A11 10m ato<br>U pGEM<br>4.12E4<br>FAM-NFQ-MGB<br>CT: 24.99	2107 A11 10m ato<br>U pGEM<br>4.14E4<br>FAM-NFQ-MGB<br>CT: 24.99	2107 A11 10m ato<br>U pGEM<br>4E4<br>FAM-NFQ-MGB<br>CT: 25.04	2107 A21 10m ato<br>U pGEM<br>3.96E4<br>FAM-NFQ-MGB<br>CT: 25.05	2107 A21 10m ato<br>U pGEM<br>4.51E4<br>FAM-NFQ-MGB<br>CT: 24.86	2107 A21 10m ato<br>U pGEM<br>4.18E4<br>FAM-NFQ-MGB<br>CT: 24.97	2107 A11 3000m saki<br>U pGEM<br>1.5E4<br>FAM-NFQ-MGB<br>CT: 26.49	2107 A21 3000m saki<br>U pGEM<br>1.41E4<br>FAM-NFQ-MGB<br>CT: 26.58		
C	S pGEM<br>2E4<br>FAM-NFQ-MGB<br>CT: 26.09	S pGEM<br>2E4<br>FAM-NFQ-MGB<br>CT: 25.73	2107 A11 SCM saki<br>U pGEM<br>2.25E4<br>FAM-NFQ-MGB<br>CT: 25.89	2107 A11 SCM saki<br>U pGEM<br>2.31E4<br>FAM-NFQ-MGB<br>CT: 25.85	2107 A11 SCM saki<br>U pGEM<br>2.11E4<br>FAM-NFQ-MGB<br>CT: 25.99	2107 A21 SCM saki<br>U pGEM<br>1.27E4<br>FAM-NFQ-MGB<br>CT: 26.74	2107 A21 SCM saki<br>U pGEM<br>1.31E4<br>FAM-NFQ-MGB<br>CT: 26.7	2107 A21 SCM saki<br>U pGEM<br>1.35E4<br>FAM-NFQ-MGB<br>CT: 26.66	2107 A11 3000m saki<br>U pGEM<br>1.38E4<br>FAM-NFQ-MGB<br>CT: 26.62	2107 A21 3000m saki<br>U pGEM<br>1.45E4<br>FAM-NFQ-MGB<br>CT: 26.54		
D	S pGEM<br>2E3<br>FAM-NFQ-MGB<br>CT: 29.73	S pGEM<br>2E3<br>FAM-NFQ-MGB<br>CT: 29.64	2107 A11 SCM ato<br>U pGEM<br>3.79E4<br>FAM-NFQ-MGB<br>CT: 25.12	2107 A11 SCM ato<br>U pGEM<br>4.47E4<br>FAM-NFQ-MGB<br>CT: 24.87	2107 A11 SCM ato<br>U pGEM<br>3.54E4<br>FAM-NFQ-MGB<br>CT: 25.22	2107 A21 SCM ato<br>U pGEM<br>4.49E4<br>FAM-NFQ-MGB<br>CT: 24.86	2107 A21 SCM ato<br>U pGEM<br>5.12E4<br>FAM-NFQ-MGB<br>CT: 24.67	2107 A21 SCM ato<br>U pGEM<br>4.31E4<br>FAM-NFQ-MGB<br>CT: 24.92	2107 A11 3000m ato<br>U pGEM<br>4.2E4<br>FAM-NFQ-MGB<br>CT: 24.97	2107 A21 3000m ato<br>U pGEM<br>4.02E4<br>FAM-NFQ-MGB<br>CT: 25.03		
E	S pGEM<br>200<br>FAM-NFQ-MGB<br>CT: 33.17	S pGEM<br>200<br>FAM-NFQ-MGB<br>CT: 33.13	2107 A11 200m saki<br>U pGEM<br>2.87E4<br>FAM-NFQ-MGB<br>CT: 25.53	2107 A11 200m saki<br>U pGEM<br>2.79E4<br>FAM-NFQ-MGB<br>CT: 25.57	2107 A11 200m saki<br>U pGEM<br>2.89E4<br>FAM-NFQ-MGB<br>CT: 25.52	2107 A21 200m saki<br>U pGEM<br>1.07E4<br>FAM-NFQ-MGB<br>CT: 27	2107 A21 200m saki<br>U pGEM<br>1.16E4<br>FAM-NFQ-MGB<br>CT: 26.88	2107 A21 200m saki<br>U pGEM<br>1.24E4<br>FAM-NFQ-MGB<br>CT: 26.78	2107 A11 3000m ato<br>U pGEM<br>4.12E4<br>FAM-NFQ-MGB<br>CT: 24.99	2107 A21 3000m ato<br>U pGEM<br>3.21E4<br>FAM-NFQ-MGB<br>CT: 25.36		
F	S pGEM<br>20<br>FAM-NFQ-MGB<br>CT: 36.27	S pGEM<br>20<br>FAM-NFQ-MGB<br>CT: 35.95	2107 A11 200m ato<br>U pGEM<br>4.54E4<br>FAM-NFQ-MGB<br>CT: 24.85	2107 A11 200m ato<br>U pGEM<br>4.06E4<br>FAM-NFQ-MGB<br>CT: 25.02	2107 A11 200m ato<br>U pGEM<br>4.04E4<br>FAM-NFQ-MGB<br>CT: 25.02	2107 A21 200m ato<br>U pGEM<br>4.03E4<br>FAM-NFQ-MGB<br>CT: 25.02	2107 A21 200m ato<br>U pGEM<br>5.19E4<br>FAM-NFQ-MGB<br>CT: 24.65	2107 A21 200m ato<br>U pGEM<br>4.86E4<br>FAM-NFQ-MGB<br>CT: 24.75	2107 A11 3000m ato<br>U pGEM<br>4.43E4<br>FAM-NFQ-MGB<br>CT: 24.88	2107 A21 3000m ato<br>U pGEM<br>3.26E4<br>FAM-NFQ-MGB<br>CT: 25.34		
G			2107 A11 1000m saki<br>U pGEM<br>1.99E4<br>FAM-NFQ-MGB<br>CT: 26.08	2107 A11 1000m saki<br>U pGEM<br>2.18E4<br>FAM-NFQ-MGB<br>CT: 25.94	2107 A11 1000m saki<br>U pGEM<br>2.01E4<br>FAM-NFQ-MGB<br>CT: 26.06	2107 A21 1000m saki<br>U pGEM<br>9.9E3<br>FAM-NFQ-MGB<br>CT: 27.11	2107 A21 1000m saki<br>U pGEM<br>9.9E3<br>FAM-NFQ-MGB<br>CT: 27.11	2107 A21 1000m saki<br>U pGEM<br>1.05E4<br>FAM-NFQ-MGB<br>CT: 27.02				N pGEM<br>FAM-NFQ-MGB<br>CT: Undetermined
H	Comf2E4<br>U pGEM<br>2.03E4<br>FAM-NFQ-MGB<br>CT: 26.04	Comf2E4<br>U pGEM<br>2.3E4<br>FAM-NFQ-MGB<br>CT: 25.86	2107 A11 1000m ato<br>U pGEM<br>4.44E4<br>FAM-NFQ-MGB<br>CT: 24.88	2107 A11 1000m ato<br>U pGEM<br>4.38E4<br>FAM-NFQ-MGB<br>CT: 24.9	2107 A11 1000m ato<br>U pGEM<br>4.16E4<br>FAM-NFQ-MGB<br>CT: 24.98	2107 A21 1000m ato<br>U pGEM<br>4.18E4<br>FAM-NFQ-MGB<br>CT: 24.97	2107 A21 1000m ato<br>U pGEM<br>4.34E4<br>FAM-NFQ-MGB<br>CT: 24.92	2107 A21 1000m ato<br>U pGEM<br>4.39E4<br>FAM-NFQ-MGB<br>CT: 24.9				N pGEM<br>FAM-NFQ-MGB<br>CT: Undetermined

Standard Curves

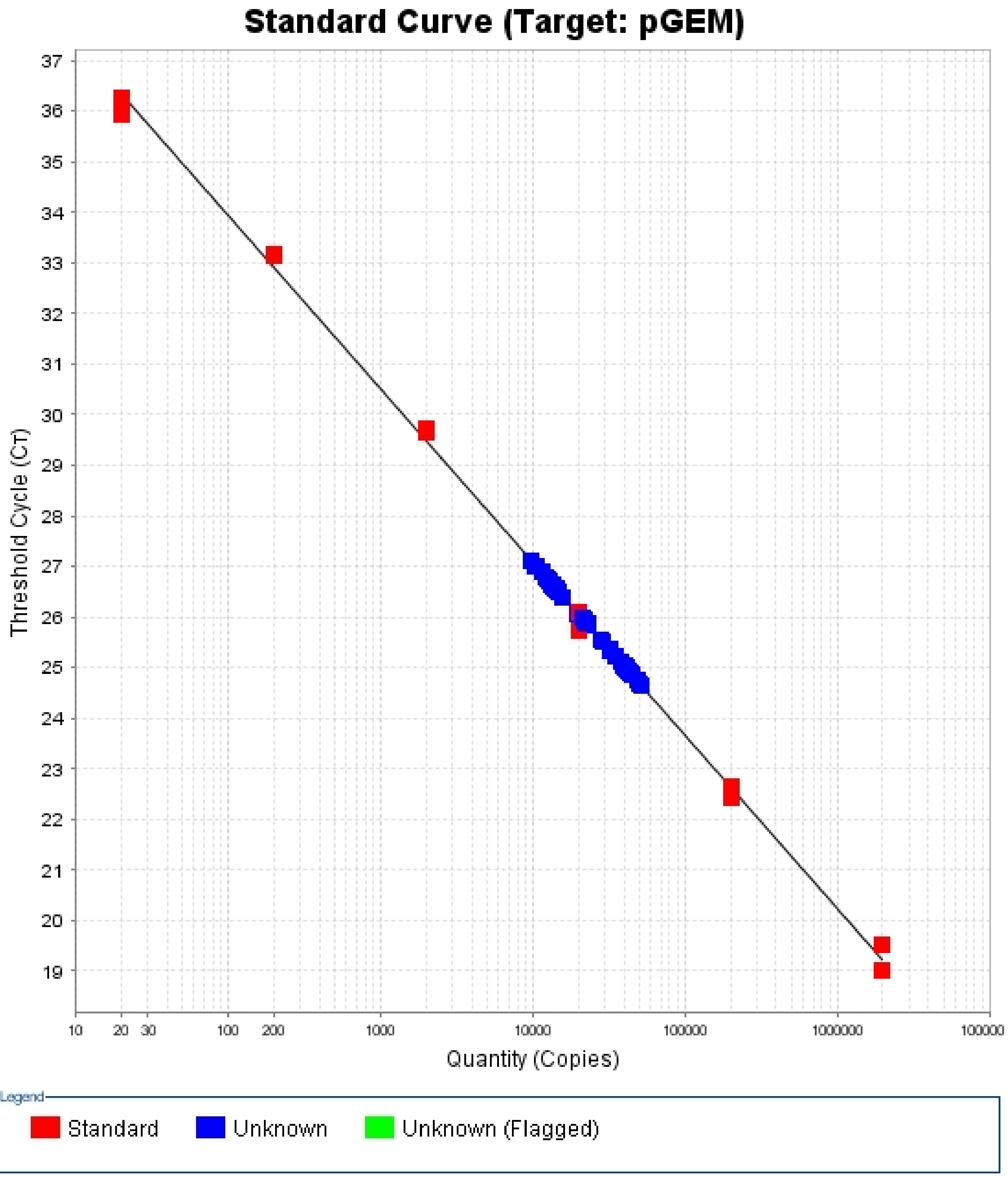

slope:-3.423 Y-Intercept:40.788 R^2:0.998 Eff%:95.964

Results Table

Well		Sample	Target	Task	Quantity	C
A1			pGEM	STANDARD	2000000	19.518
A2			pGEM	STANDARD	2000000	19.0354
A3		2107 A11 10m	pGEM	UNKNOWN	22673.9609	25.881
A4		2107 A11 10m	pGEM	UNKNOWN	21171.1055	25.9829
A5		2107 A11 10m	pGEM	UNKNOWN	22227.6445	25.9105
A6		2107 A21 10m	pGEM	UNKNOWN	13732.7217	26.6263
A7		2107 A21 10m	pGEM	UNKNOWN	15962.6309	26.4027
A8		2107 A21 10m	pGEM	UNKNOWN	15118.7695	26.4834
A9		2107 A11	pGEM	UNKNOWN	14444.123	26.5512
A10		2107 A21	pGEM	UNKNOWN	12722.0205	26.7399
B1			pGEM	STANDARD	200000	22.6405
B2			pGEM	STANDARD	200000	22.4343
B3		2107 A11 10m	pGEM	UNKNOWN	41162.7188	24.9946
B4		2107 A11 10m	pGEM	UNKNOWN	41385.2148	24.9866
B5		2107 A11 10m	pGEM	UNKNOWN	39960.3438	25.0387
B6		2107 A21 10m	pGEM	UNKNOWN	39563.5469	25.0535
B7		2107 A21 10m	pGEM	UNKNOWN	45143.1133	24.8574
B8		2107 A21 10m	pGEM	UNKNOWN	41758.2461	24.9732
B9		2107 A11	pGEM	UNKNOWN	15046.0391	26.4906
B10		2107 A21	pGEM	UNKNOWN	14132.4668	26.5837
C1			pGEM	STANDARD	20000	26.0913
C2			pGEM	STANDARD	20000	25.7274
C3		2107 A11 SCM	pGEM	UNKNOWN	22467.1719	25.8946
C4		2107 A11 SCM	pGEM	UNKNOWN	23133.127	25.8512
C5		2107 A11 SCM	pGEM	UNKNOWN	21108.2812	25.9873
C6		2107 A21 SCM	pGEM	UNKNOWN	12743.0479	26.7375
C7		2107 A21 SCM	pGEM	UNKNOWN	13061.7373	26.7008
C8		2107 A21 SCM	pGEM	UNKNOWN	13465.1699	26.6556
C9		2107 A11	pGEM	UNKNOWN	13805.6748	26.6184
C10		2107 A21	pGEM	UNKNOWN	14517.334	26.5437

Well	Sample	Target	Task	Quantity	C
D1		pGEM	STANDARD	2000	29.7334
D2		pGEM	STANDARD	2000	29.6438
D3	2107 A11 SCM	pGEM	UNKNOWN	37919.5977	25.1166
D4	2107 A11 SCM	pGEM	UNKNOWN	44705.6953	24.8719
D5	2107 A11 SCM	pGEM	UNKNOWN	35390.3477	25.2192
D6	2107 A21 SCM	pGEM	UNKNOWN	44915.8008	24.8649
D7	2107 A21 SCM	pGEM	UNKNOWN	51167.8438	24.6712
D8	2107 A21 SCM	pGEM	UNKNOWN	43142.5234	24.9248
D9	2107 A11	pGEM	UNKNOWN	41986.6523	24.9651
D10	2107 A21	pGEM	UNKNOWN	40211.9258	25.0293
E1		pGEM	STANDARD	200	33.1667
E2		pGEM	STANDARD	200	33.1294
E3	2107 A11 200m	pGEM	UNKNOWN	28702.5488	25.5305
E4	2107 A11 200m	pGEM	UNKNOWN	27947.752	25.5701
E5	2107 A11 200m	pGEM	UNKNOWN	28949.9336	25.5178
E6	2107 A21 200m	pGEM	UNKNOWN	10673.5381	27.0009
E7	2107 A21 200m	pGEM	UNKNOWN	11559.2744	26.8824
E8	2107 A21 200m	pGEM	UNKNOWN	12389.5957	26.7793
E9	2107 A11	pGEM	UNKNOWN	41158.2852	24.9948
E10	2107 A21	pGEM	UNKNOWN	32139.5469	25.3624
F1		pGEM	STANDARD	20	36.273
F2		pGEM	STANDARD	20	35.9525
F3	2107 A11 200m	pGEM	UNKNOWN	45424.4102	24.8482
F4	2107 A11 200m	pGEM	UNKNOWN	40573.082	25.016
F5	2107 A11 200m	pGEM	UNKNOWN	40400.1875	25.0224
F6	2107 A21 200m	pGEM	UNKNOWN	40333.625	25.0248
F7	2107 A21 200m	pGEM	UNKNOWN	51896.4688	24.6502
F8	2107 A21 200m	pGEM	UNKNOWN	48570.8828	24.7486
F9	2107 A11	pGEM	UNKNOWN	44337.668	24.8842
F10	2107 A21	pGEM	UNKNOWN	32587.3398	25.3418
G3	2107 A11	pGEM	UNKNOWN	19868.7441	26.0773
G4	2107 A11	pGEM	UNKNOWN	21775.5508	25.9411

Well		Sample	Target	Task	Quantity	C
G5		2107 A11	pGEM	UNKNOWN	20114.2637	26.059
G6		2107 A21	pGEM	UNKNOWN	9903.5029	27.1122
G7		2107 A21	pGEM	UNKNOWN	9897.1133	27.1132
G8		2107 A21	pGEM	UNKNOWN	10531.4492	27.0208
G12			pGEM	NTC		Undetermined
H1		Comf2E4	pGEM	UNKNOWN	20338.8184	26.0425
H2		Comf2E4	pGEM	UNKNOWN	22965.8418	25.862
H3		2107 A11	pGEM	UNKNOWN	44357.9258	24.8835
H4		2107 A11	pGEM	UNKNOWN	43802.0898	24.9022
H5		2107 A11	pGEM	UNKNOWN	41560.0312	24.9803
H6		2107 A21	pGEM	UNKNOWN	41768.1094	24.9729
H7		2107 A21	pGEM	UNKNOWN	43355.0195	24.9175
H8		2107 A21	pGEM	UNKNOWN	43924.3398	24.8981
H12			pGEM	NTC		Undetermined

QC Summary

Total Wells	96	Processed Wells	76	Targets Used	1
Well Setup	76	Flagged Wells	0	Samples Used	21

Flag	Name	Frequency	Locations
AMPNC	Amplification in negative control	0	
BADROX	Bad passive reference signal	0	
BLFAIL	Baseline algorithm failed	0	
CTFAIL	C algorithm failed	0	
EXPFAIL	Exponential algorithm failed	0	
HIGHSD	High standard deviation in replicate group	0	
NOAMP	No amplification	0	
NOISE	Noise higher than others in plate	0	
NOSIGNAL	No signal in well	0	
OFFSCALE	Fluorescence is offscale	0	
OUTLIERRG	Outlier in replicate group	0	
SPIKE	Noise spikes	0	
THOLDFAIL	Thresholding algorithm failed	0	
