# Supplementary material for: Ecological impact of algal-preying protists aplanochytrids (Labyrinthulea) as a source of docosahexaenoic acid for copepods in marine environments
Source: ISME Commun. 2026 Jun 26;6(1):ycag178. doi: 10.1093/ismeco/ycag178 (PMC13685785; doi:10.1093/ismeco/ycag178)
Supplement: Supplementary file 2 [file supplementary_materials_ycag178.zip › Morimoto_ISME_C_3rdrev_supplemental_data.zip!/Morimoto_ISME_C_3rdrev_supplemental_data/Supplemental_Data_2/Standard Curve/DNA recovery/2107/DnaRec2107AlineA11-A21N=2.pdf]

Experiment Results Report
230719DnaRec2107AlineA11-A21N=2

Experiment Summary

Experiment Name	:	230719DnaRec2107AlineA11-A21N=2
Experiment Type	:	Quantitation - Standard Curve
File Name	:	230719DnaRec2107AlineA11-A21N=2.eds
Run Started	:	2023 Jul 19 12:24:02 PM
Run Finished	:	2023 Jul 19 1:58:43 PM
Run Duration	:	94 minutes 40 seconds
Date Modified	:	2023 Jul 20 10:50:39 AM
User	:	
Number of wells used	:	62
Number of wells with results	:	62
Instrument Name	:	
Instrument Type	:	Applied Biosystems StepOnePlus™ Instrument
Comments	:	

Results Summary

Sample	Target	Quantity (Mean)	Quantity (Std Dev)	C (Mean)	C (Std Dev)
2107 A11 1000m ato	pGEM	38681.53	3139.73	25.20	0.13
2107 A11 1000m	pGEM	24643.72	457.85	25.88	0.03
2107 A11 10m ato	pGEM	34961.58	1538.90	25.35	0.07
2107 A11 10m saki	pGEM	15347.42	11892.25	27.32	2.21
2107 A11 200m ato	pGEM	33681.29	1951.69	25.41	0.09
2107 A11 200m saki	pGEM	24054.78	988.41	25.92	0.06
2107 A11 3000m ato	pGEM	29444.68	1144.70	25.61	0.06
2107 A11 3000m	pGEM	13256.44	1758.85	26.83	0.21
2107 A11 SCM ato	pGEM	33417.53	777.79	25.42	0.04
2107 A11 SCM saki	pGEM	25714.56	1917.52	25.82	0.11
2107 A21 1000m ato	pGEM	23614.14	12498.04	26.06	0.84
2107 A21 1000m	pGEM	14560.82	6552.26	26.76	0.71
2107 A21 200m ato	pGEM	23787.24	10563.12	26.01	0.70
2107 A21 200m saki	pGEM	16219.74	7586.74	26.60	0.74
2107 A21 3000m ato	pGEM	27309.85	11362.96	25.79	0.65
2107 A21 3000m	pGEM	5145.20	2612.90	28.36	0.81
2107 A21 SCM ato	pGEM	24142.29	8149.14	25.96	0.52
2107 A21 SCM saki	pGEM	13832.11	7712.39	26.89	0.90
Comf2E4	pGEM	26014.60	795.49	25.80	0.05

Plate Layout

	1	2	3	4	5	6	7	8	9	10	11	12
A	S pGEM<br>2E6<br>FAM-NFQ-MGB<br>CT: 19.91	S pGEM<br>2E6<br>FAM-NFQ-MGB<br>CT: 19.42	2107 A11 10m saki<br>U pGEM<br>2.4E4<br>FAM-NFQ-MGB<br>CT: 25.92	2107 A11 10m saki<br>U pGEM<br>1.78E3<br>FAM-NFQ-MGB<br>CT: 29.87	2107 A11 10m saki<br>U pGEM<br>2.03E4<br>FAM-NFQ-MGB<br>CT: 26.18	2107 A11 3000m saki<br>U pGEM<br>1.12E4<br>FAM-NFQ-MGB<br>CT: 27.07	2107 A21 SCM saki<br>U pGEM<br>8.38E3<br>FAM-NFQ-MGB<br>CT: 27.52	2107 A21 SCM saki<br>U pGEM<br>1.93E4<br>FAM-NFQ-MGB<br>CT: 26.25				
B	S pGEM<br>2E5<br>FAM-NFQ-MGB<br>CT: 22.46	S pGEM<br>2E5<br>FAM-NFQ-MGB<br>CT: 22.29	2107 A11 10m ato<br>U pGEM<br>3.54E4<br>FAM-NFQ-MGB<br>CT: 25.33	2107 A11 10m ato<br>U pGEM<br>3.62E4<br>FAM-NFQ-MGB<br>CT: 25.3	2107 A11 10m ato<br>U pGEM<br>3.32E4<br>FAM-NFQ-MGB<br>CT: 25.43	2107 A11 3000m saki<br>U pGEM<br>1.4E4<br>FAM-NFQ-MGB<br>CT: 26.74	2107 A21 SCM ato<br>U pGEM<br>1.84E4<br>FAM-NFQ-MGB<br>CT: 26.33	2107 A21 SCM ato<br>U pGEM<br>2.99E4<br>FAM-NFQ-MGB<br>CT: 25.59				
C	S pGEM<br>2E4<br>FAM-NFQ-MGB<br>CT: 26.19	S pGEM<br>2E4<br>FAM-NFQ-MGB<br>CT: 25.78	2107 A11 SCM saki<br>U pGEM<br>2.5E4<br>FAM-NFQ-MGB<br>CT: 25.86	2107 A11 SCM saki<br>U pGEM<br>2.79E4<br>FAM-NFQ-MGB<br>CT: 25.69	2107 A11 SCM saki<br>U pGEM<br>2.42E4<br>FAM-NFQ-MGB<br>CT: 25.91	2107 A11 3000m saki<br>U pGEM<br>1.45E4<br>FAM-NFQ-MGB<br>CT: 26.68	2107 A21 200m saki<br>U pGEM<br>1.09E4<br>FAM-NFQ-MGB<br>CT: 27.13	2107 A21 200m saki<br>U pGEM<br>2.16E4<br>FAM-NFQ-MGB<br>CT: 26.08				
D	S pGEM<br>2E3<br>FAM-NFQ-MGB<br>CT: 29.6	S pGEM<br>2E3<br>FAM-NFQ-MGB<br>CT: 29.47	2107 A11 SCM ato<br>U pGEM<br>3.25E4<br>FAM-NFQ-MGB<br>CT: 25.46	2107 A11 SCM ato<br>U pGEM<br>3.39E4<br>FAM-NFQ-MGB<br>CT: 25.4	2107 A11 SCM ato<br>U pGEM<br>3.38E4<br>FAM-NFQ-MGB<br>CT: 25.4	2107 A11 3000m ato<br>U pGEM<br>2.83E4<br>FAM-NFQ-MGB<br>CT: 25.67	2107 A21 200m ato<br>U pGEM<br>1.63E4<br>FAM-NFQ-MGB<br>CT: 26.51	2107 A21 200m ato<br>U pGEM<br>3.13E4<br>FAM-NFQ-MGB<br>CT: 25.52				
E	S pGEM<br>200<br>FAM-NFQ-MGB<br>CT: 33.18	S pGEM<br>200<br>FAM-NFQ-MGB<br>CT: 33.12	2107 A11 200m saki<br>U pGEM<br>2.3E4<br>FAM-NFQ-MGB<br>CT: 25.98	2107 A11 200m saki<br>U pGEM<br>2.42E4<br>FAM-NFQ-MGB<br>CT: 25.91	2107 A11 200m saki<br>U pGEM<br>2.5E4<br>FAM-NFQ-MGB<br>CT: 25.86	2107 A11 3000m ato<br>U pGEM<br>3.05E4<br>FAM-NFQ-MGB<br>CT: 25.56	2107 A21 1000m saki<br>U pGEM<br>9.93E3<br>FAM-NFQ-MGB<br>CT: 27.26	2107 A21 1000m saki<br>U pGEM<br>1.92E4<br>FAM-NFQ-MGB<br>CT: 26.26				
F	S pGEM<br>20<br>FAM-NFQ-MGB<br>CT: 36.97	S pGEM<br>20<br>FAM-NFQ-MGB<br>CT: 36.96	2107 A11 200m ato<br>U pGEM<br>3.54E4<br>FAM-NFQ-MGB<br>CT: 25.33	2107 A11 200m ato<br>U pGEM<br>3.16E4<br>FAM-NFQ-MGB<br>CT: 25.51	2107 A11 200m ato<br>U pGEM<br>3.41E4<br>FAM-NFQ-MGB<br>CT: 25.39	2107 A11 3000m ato<br>U pGEM<br>2.95E4<br>FAM-NFQ-MGB<br>CT: 25.61	2107 A21 1000m ato<br>U pGEM<br>1.48E4<br>FAM-NFQ-MGB<br>CT: 26.66	2107 A21 1000m ato<br>U pGEM<br>3.25E4<br>FAM-NFQ-MGB<br>CT: 25.46				
G			2107 A11 1000m saki<br>U pGEM<br>2.44E4<br>FAM-NFQ-MGB<br>CT: 25.9	2107 A11 1000m saki<br>U pGEM<br>2.52E4<br>FAM-NFQ-MGB<br>CT: 25.85	2107 A11 1000m saki<br>U pGEM<br>2.44E4<br>FAM-NFQ-MGB<br>CT: 25.9		2107 A21 3000m saki<br>U pGEM<br>3.3E3<br>FAM-NFQ-MGB<br>CT: 28.93	2107 A21 3000m saki<br>U pGEM<br>6.99E3<br>FAM-NFQ-MGB<br>CT: 27.79				N pGEM<br>FAM-NFQ-MGB<br>CT: Undetermined
H	Comf2E4<br>U pGEM<br>2.66E4<br>FAM-NFQ-MGB<br>CT: 25.77	Comf2E4<br>U pGEM<br>2.55E4<br>FAM-NFQ-MGB<br>CT: 25.83	2107 A11 1000m ato<br>U pGEM<br>4.13E4<br>FAM-NFQ-MGB<br>CT: 25.1	2107 A11 1000m ato<br>U pGEM<br>3.95E4<br>FAM-NFQ-MGB<br>CT: 25.16	2107 A11 1000m ato<br>U pGEM<br>3.52E4<br>FAM-NFQ-MGB<br>CT: 25.34		2107 A21 3000m ato<br>U pGEM<br>1.93E4<br>FAM-NFQ-MGB<br>CT: 26.25	2107 A21 3000m ato<br>U pGEM<br>3.53E4<br>FAM-NFQ-MGB<br>CT: 25.33				N pGEM<br>FAM-NFQ-MGB<br>CT: Undetermined

Standard Curves

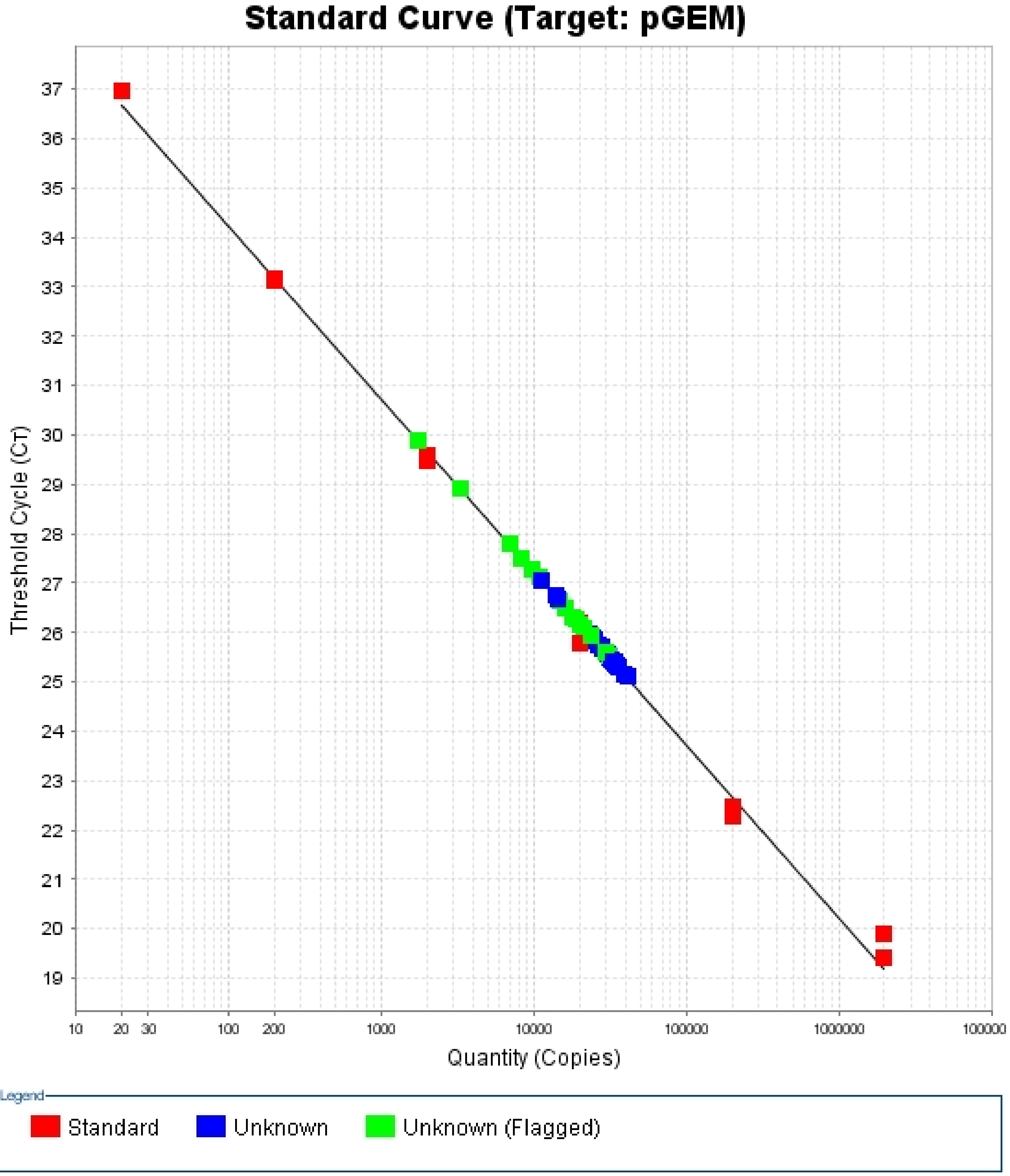

slope:-3.496 Y-Intercept:41.236 R^2:0.997 Eff%:93.204

Results Table

Well		Sample	Target	Task	Quantity	C
A1			pGEM	STANDARD	2000000	19.9137
A2			pGEM	STANDARD	2000000	19.4202
A3		2107 A11 10m	pGEM	UNKNOWN	23963.1035	25.9233
A4		2107 A11 10m	pGEM	UNKNOWN	1779.2812	29.8717
A5		2107 A11 10m	pGEM	UNKNOWN	20299.875	26.1752
A6		2107 A11	pGEM	UNKNOWN	11246.7676	27.0719
A7		2107 A21 SCM	pGEM	UNKNOWN	8378.624	27.5189
A8		2107 A21 SCM	pGEM	UNKNOWN	19285.5879	26.253
B1			pGEM	STANDARD	200000	22.4552
B2			pGEM	STANDARD	200000	22.2863
B3		2107 A11 10m	pGEM	UNKNOWN	35426.2852	25.3297
B4		2107 A11 10m	pGEM	UNKNOWN	36214.5742	25.2963
B5		2107 A11 10m	pGEM	UNKNOWN	33243.8867	25.4262
B6		2107 A11	pGEM	UNKNOWN	14007.3291	26.7386
B7		2107 A21 SCM	pGEM	UNKNOWN	18379.9766	26.3261
B8		2107 A21 SCM	pGEM	UNKNOWN	29904.6055	25.587
C1			pGEM	STANDARD	20000	26.1931
C2			pGEM	STANDARD	20000	25.7805
C3		2107 A11 SCM	pGEM	UNKNOWN	25012.6309	25.8582
C4		2107 A11 SCM	pGEM	UNKNOWN	27884.1426	25.6932
C5		2107 A11 SCM	pGEM	UNKNOWN	24246.9062	25.9054
C6		2107 A11	pGEM	UNKNOWN	14515.2119	26.6845
C7		2107 A21 200m	pGEM	UNKNOWN	10855.1006	27.1257
C8		2107 A21 200m	pGEM	UNKNOWN	21584.3711	26.082
D1			pGEM	STANDARD	2000	29.599
D2			pGEM	STANDARD	2000	29.4728
D3		2107 A11 SCM	pGEM	UNKNOWN	32520.4277	25.4596
D4		2107 A11 SCM	pGEM	UNKNOWN	33902.9023	25.3964
D5		2107 A11 SCM	pGEM	UNKNOWN	33829.2617	25.3997
D6		2107 A11	pGEM	UNKNOWN	28256.3789	25.6731

Well		Sample	Target	Task	Quantity	C
D7		2107 A21 200m	pGEM	UNKNOWN	16317.9863	26.5068
D8		2107 A21 200m	pGEM	UNKNOWN	31256.4961	25.5198
E1			pGEM	STANDARD	200	33.184
E2			pGEM	STANDARD	200	33.1175
E3		2107 A11 200m	pGEM	UNKNOWN	23019.7559	25.9843
E4		2107 A11 200m	pGEM	UNKNOWN	24155.7676	25.9111
E5		2107 A11 200m	pGEM	UNKNOWN	24988.8281	25.8597
E6		2107 A11	pGEM	UNKNOWN	30540.127	25.555
E7		2107 A21	pGEM	UNKNOWN	9927.6738	27.2613
E8		2107 A21	pGEM	UNKNOWN	19193.9668	26.2603
F1			pGEM	STANDARD	20	36.9711
F2			pGEM	STANDARD	20	36.956
F3		2107 A11 200m	pGEM	UNKNOWN	35385.7695	25.3314
F4		2107 A11 200m	pGEM	UNKNOWN	31552.2773	25.5055
F5		2107 A11 200m	pGEM	UNKNOWN	34105.8359	25.3874
F6		2107 A11	pGEM	UNKNOWN	29537.543	25.6057
F7		2107 A21	pGEM	UNKNOWN	14776.6865	26.6574
F8		2107 A21	pGEM	UNKNOWN	32451.5859	25.4629
G3		2107 A11	pGEM	UNKNOWN	24388.0898	25.8966
G4		2107 A11	pGEM	UNKNOWN	25172.3086	25.8486
G5		2107 A11	pGEM	UNKNOWN	24370.7578	25.8977
G7		2107 A21	pGEM	UNKNOWN	3297.6008	28.9348
G8		2107 A21	pGEM	UNKNOWN	6992.8032	27.7934
G12			pGEM	NTC		Undetermined
H1		Comf2E4	pGEM	UNKNOWN	26577.0977	25.7661
H2		Comf2E4	pGEM	UNKNOWN	25452.1074	25.8318
H3		2107 A11	pGEM	UNKNOWN	41314.9375	25.0962
H4		2107 A11	pGEM	UNKNOWN	39522.7891	25.1635
H5		2107 A11	pGEM	UNKNOWN	35206.8711	25.3391
H7		2107 A21	pGEM	UNKNOWN	19275.0273	26.2539
H8		2107 A21	pGEM	UNKNOWN	35344.6758	25.3332
H12			pGEM	NTC		Undetermined

QC Summary

Total Wells	96	Processed Wells	62	Targets Used	1
Well Setup	62	Flagged Wells	19	Samples Used	19

Flag	Name	Frequency	Locations
AMPNC	Amplification in negative control	0	
BADROX	Bad passive reference signal	0	
BLFAIL	Baseline algorithm failed	0	
CTFAIL	C algorithm failed	0	
EXPFAIL	Exponential algorithm failed	0	
HIGHSD	High standard deviation in replicate group	19	A3, A4, A5, A7, A8, B7, B8, C7, C8, D7, D8, E7, E8, F7, F8, G7, G8, H7, H8
NOAMP	No amplification	0	
NOISE	Noise higher than others in plate	0	
NOSIGNAL	No signal in well	0	
OFFSCALE	Fluorescence is offscale	0	
OUTLIERRG	Outlier in replicate group	0	
SPIKE	Noise spikes	0	
THOLDFAIL	Thresholding algorithm failed	0	
