# Supplementary material for: Ecological impact of algal-preying protists aplanochytrids (Labyrinthulea) as a source of docosahexaenoic acid for copepods in marine environments
Source: ISME Commun. 2026 Jun 26;6(1):ycag178. doi: 10.1093/ismeco/ycag178 (PMC13685785; doi:10.1093/ismeco/ycag178)
Supplement: Supplementary file 2 [file supplementary_materials_ycag178.zip › Morimoto_ISME_C_3rdrev_supplemental_data.zip!/Morimoto_ISME_C_3rdrev_supplemental_data/Supplemental_Data_2/Standard Curve/DNA recovery/2107/DnaRec2107AlineA11-A21N=3.pdf]

Experiment Results Report
230720DnaRec2107AlineA11-A21N=3

Experiment Summary

Experiment Name	: 230720DnaRec2107AlineA11-A21N=3
Experiment Type	: Quantitation - Standard Curve
File Name	: 230720DnaRec2107AlineA11-A21N=3.eds
Run Started	: 2023 Jul 20 2:41:20 PM
Run Finished	: 2023 Jul 20 4:15:55 PM
Run Duration	: 94 minutes 35 seconds
Date Modified	: 2023 Jul 20 4:33:38 PM
User	:
Number of wells used	: 74
Number of wells with results	: 74
Instrument Name	:
Instrument Type	: Applied Biosystems StepOnePlus™ Instrument
Comments	:

Results Summary

Sample	Target	Quantity (Mean)	Quantity (Std Dev)	C (Mean)	C (Std Dev)
2107 A11 1000m ato	pGEM	22251.45	412.19	25.53	0.03
2107 A11 1000m	pGEM	11712.91	391.93	26.52	0.05
2107 A11 10m ato	pGEM	24709.17	1280.37	25.37	0.08
2107 A11 10m saki	pGEM	9373.31	662.33	26.86	0.11
2107 A11 200m ato	pGEM	25542.74	1361.41	25.32	0.08
2107 A11 200m saki	pGEM	10512.61	713.15	26.68	0.10
2107 A11 3000m ato	pGEM	21317.27	786.80	25.59	0.06
2107 A11 3000m	pGEM	6412.37	1139.80	27.46	0.28
2107 A11 SCM ato	pGEM	24612.79	1080.69	25.37	0.07
2107 A11 SCM saki	pGEM	8512.16	274.51	27.01	0.05
2107 A21 1000m ato	pGEM	22782.91	600.97	25.49	0.04
2107 A21 1000m	pGEM	7774.94	702.45	27.15	0.14
2107 A21 10m ato	pGEM	21530.02	378.63	25.58	0.03
2107 A21 10m saki	pGEM	12297.73	1004.09	26.44	0.12
2107 A21 200m ato	pGEM	23102.16	647.76	25.47	0.04
2107 A21 200m saki	pGEM	10283.79	297.62	26.72	0.04
2107 A21 3000m ato	pGEM	20429.44	1047.62	25.66	0.08
2107 A21 3000m	pGEM	8115.37	1037.59	27.09	0.21
2107 A21 SCM ato	pGEM	21798.24	339.31	25.56	0.02
2107 A21 SCM saki	pGEM	8999.96	1665.31	26.94	0.30

Plate Layout

	1	2	3	4	5	6	7	8	9	10	11	12
A	S pGEM<br>2E6<br>FAM-NFQ-MGB<br>CT: 18.59	S pGEM<br>2E6<br>FAM-NFQ-MGB<br>CT: 18.2	2107 A11 10m saki<br>U pGEM<br>9.22E3<br>FAM-NFQ-MGB<br>CT: 26.88	2107 A11 10m saki<br>U pGEM<br>1.01E4<br>FAM-NFQ-MGB<br>CT: 26.74	2107 A11 10m saki<br>U pGEM<br>8.8E3<br>FAM-NFQ-MGB<br>CT: 26.96	2107 A11 3000m saki<br>U pGEM<br>5.2E3<br>FAM-NFQ-MGB<br>CT: 27.76	2107 A21 10m saki<br>U pGEM<br>1.17E4<br>FAM-NFQ-MGB<br>CT: 26.51	2107 A21 10m saki<br>U pGEM<br>1.17E4<br>FAM-NFQ-MGB<br>CT: 26.51	2107 A21 10m saki<br>U pGEM<br>1.35E4<br>FAM-NFQ-MGB<br>CT: 26.3	2107 A21 3000m saki<br>U pGEM<br>6.92E3<br>FAM-NFQ-MGB<br>CT: 27.33		
B	S pGEM<br>2E5<br>FAM-NFQ-MGB<br>CT: 22.28	S pGEM<br>2E5<br>FAM-NFQ-MGB<br>CT: 22.18	2107 A11 10m ato<br>U pGEM<br>2.41E4<br>FAM-NFQ-MGB<br>CT: 25.41	2107 A11 10m ato<br>U pGEM<br>2.62E4<br>FAM-NFQ-MGB<br>CT: 25.28	2107 A11 10m ato<br>U pGEM<br>2.39E4<br>FAM-NFQ-MGB<br>CT: 25.42	2107 A11 3000m saki<br>U pGEM<br>6.57E3<br>FAM-NFQ-MGB<br>CT: 27.41	2107 A21 10m ato<br>U pGEM<br>2.14E4<br>FAM-NFQ-MGB<br>CT: 25.59	2107 A21 10m ato<br>U pGEM<br>2.12E4<br>FAM-NFQ-MGB<br>CT: 25.6	2107 A21 10m ato<br>U pGEM<br>2.2E4<br>FAM-NFQ-MGB<br>CT: 25.55	2107 A21 3000m saki<br>U pGEM<br>8.75E3<br>FAM-NFQ-MGB<br>CT: 26.96		
C	S pGEM<br>2E4<br>FAM-NFQ-MGB<br>CT: 25.84	S pGEM<br>2E4<br>FAM-NFQ-MGB<br>CT: 25.72	2107 A11 SCM saki<br>U pGEM<br>8.28E3<br>FAM-NFQ-MGB<br>CT: 27.05	2107 A11 SCM saki<br>U pGEM<br>8.82E3<br>FAM-NFQ-MGB<br>CT: 26.95	2107 A11 SCM saki<br>U pGEM<br>8.44E3<br>FAM-NFQ-MGB<br>CT: 27.02	2107 A11 3000m saki<br>U pGEM<br>7.47E3<br>FAM-NFQ-MGB<br>CT: 27.21	2107 A21 SCM saki<br>U pGEM<br>1.03E4<br>FAM-NFQ-MGB<br>CT: 26.71	2107 A21 SCM saki<br>U pGEM<br>9.51E3<br>FAM-NFQ-MGB<br>CT: 26.84	2107 A21 SCM saki<br>U pGEM<br>7.14E3<br>FAM-NFQ-MGB<br>CT: 27.28	2107 A21 3000m saki<br>U pGEM<br>8.68E3<br>FAM-NFQ-MGB<br>CT: 26.98		
D	S pGEM<br>2E3<br>FAM-NFQ-MGB<br>CT: 29.33	S pGEM<br>2E3<br>FAM-NFQ-MGB<br>CT: 29.34	2107 A11 SCM ato<br>U pGEM<br>2.47E4<br>FAM-NFQ-MGB<br>CT: 25.37	2107 A11 SCM ato<br>U pGEM<br>2.57E4<br>FAM-NFQ-MGB<br>CT: 25.31	2107 A11 SCM ato<br>U pGEM<br>2.35E4<br>FAM-NFQ-MGB<br>CT: 25.44	2107 A11 3000m ato<br>U pGEM<br>2.18E4<br>FAM-NFQ-MGB<br>CT: 25.56	2107 A21 SCM ato<br>U pGEM<br>2.14E4<br>FAM-NFQ-MGB<br>CT: 25.59	2107 A21 SCM ato<br>U pGEM<br>2.21E4<br>FAM-NFQ-MGB<br>CT: 25.54	2107 A21 SCM ato<br>U pGEM<br>2.19E4<br>FAM-NFQ-MGB<br>CT: 25.55	2107 A21 3000m ato<br>U pGEM<br>2E4<br>FAM-NFQ-MGB<br>CT: 25.69		
E	S pGEM<br>200<br>FAM-NFQ-MGB<br>CT: 32.79	S pGEM<br>200<br>FAM-NFQ-MGB<br>CT: 33.31	2107 A11 200m saki<br>U pGEM<br>1.04E4<br>FAM-NFQ-MGB<br>CT: 26.69	2107 A11 200m saki<br>U pGEM<br>1.13E4<br>FAM-NFQ-MGB<br>CT: 26.58	2107 A11 200m saki<br>U pGEM<br>9.84E3<br>FAM-NFQ-MGB<br>CT: 26.78	2107 A11 3000m ato<br>U pGEM<br>2.04E4<br>FAM-NFQ-MGB<br>CT: 25.66	2107 A21 200m saki<br>U pGEM<br>9.99E3<br>FAM-NFQ-MGB<br>CT: 26.76	2107 A21 200m saki<br>U pGEM<br>1.03E4<br>FAM-NFQ-MGB<br>CT: 26.72	2107 A21 200m saki<br>U pGEM<br>1.06E4<br>FAM-NFQ-MGB<br>CT: 26.67	2107 A21 3000m ato<br>U pGEM<br>1.96E4<br>FAM-NFQ-MGB<br>CT: 25.72		
F	S pGEM<br>20<br>FAM-NFQ-MGB<br>CT: 35.98	S pGEM<br>20<br>FAM-NFQ-MGB<br>CT: 36.02	2107 A11 200m ato<br>U pGEM<br>2.55E4<br>FAM-NFQ-MGB<br>CT: 25.32	2107 A11 200m ato<br>U pGEM<br>2.42E4<br>FAM-NFQ-MGB<br>CT: 25.4	2107 A11 200m ato<br>U pGEM<br>2.69E4<br>FAM-NFQ-MGB<br>CT: 25.23	2107 A11 3000m ato<br>U pGEM<br>2.17E4<br>FAM-NFQ-MGB<br>CT: 25.57	2107 A21 200m ato<br>U pGEM<br>2.37E4<br>FAM-NFQ-MGB<br>CT: 25.43	2107 A21 200m ato<br>U pGEM<br>2.32E4<br>FAM-NFQ-MGB<br>CT: 25.46	2107 A21 200m ato<br>U pGEM<br>2.24E4<br>FAM-NFQ-MGB<br>CT: 25.52	2107 A21 3000m ato<br>U pGEM<br>2.16E4<br>FAM-NFQ-MGB<br>CT: 25.57		
G			2107 A11 1000m saki<br>U pGEM<br>1.2E4<br>FAM-NFQ-MGB<br>CT: 26.48	2107 A11 1000m saki<br>U pGEM<br>1.19E4<br>FAM-NFQ-MGB<br>CT: 26.49	2107 A11 1000m saki<br>U pGEM<br>1.13E4<br>FAM-NFQ-MGB<br>CT: 26.58		2107 A21 1000m saki<br>U pGEM<br>7.37E3<br>FAM-NFQ-MGB<br>CT: 27.23	2107 A21 1000m saki<br>U pGEM<br>8.59E3<br>FAM-NFQ-MGB<br>CT: 26.99	2107 A21 1000m saki<br>U pGEM<br>7.37E3<br>FAM-NFQ-MGB<br>CT: 27.23			
H	N pGEM<br>FAM-NFQ-MGB<br>CT: Undetermined	N pGEM<br>FAM-NFQ-MGB<br>CT: Undetermined	2107 A11 1000m ato<br>U pGEM<br>2.19E4<br>FAM-NFQ-MGB<br>CT: 25.55	2107 A11 1000m ato<br>U pGEM<br>2.21E4<br>FAM-NFQ-MGB<br>CT: 25.54	2107 A11 1000m ato<br>U pGEM<br>2.27E4<br>FAM-NFQ-MGB<br>CT: 25.5		2107 A21 1000m ato<br>U pGEM<br>2.29E4<br>FAM-NFQ-MGB<br>CT: 25.48	2107 A21 1000m ato<br>U pGEM<br>2.33E4<br>FAM-NFQ-MGB<br>CT: 25.46	2107 A21 1000m ato<br>U pGEM<br>2.21E4<br>FAM-NFQ-MGB<br>CT: 25.54			

Standard Curves

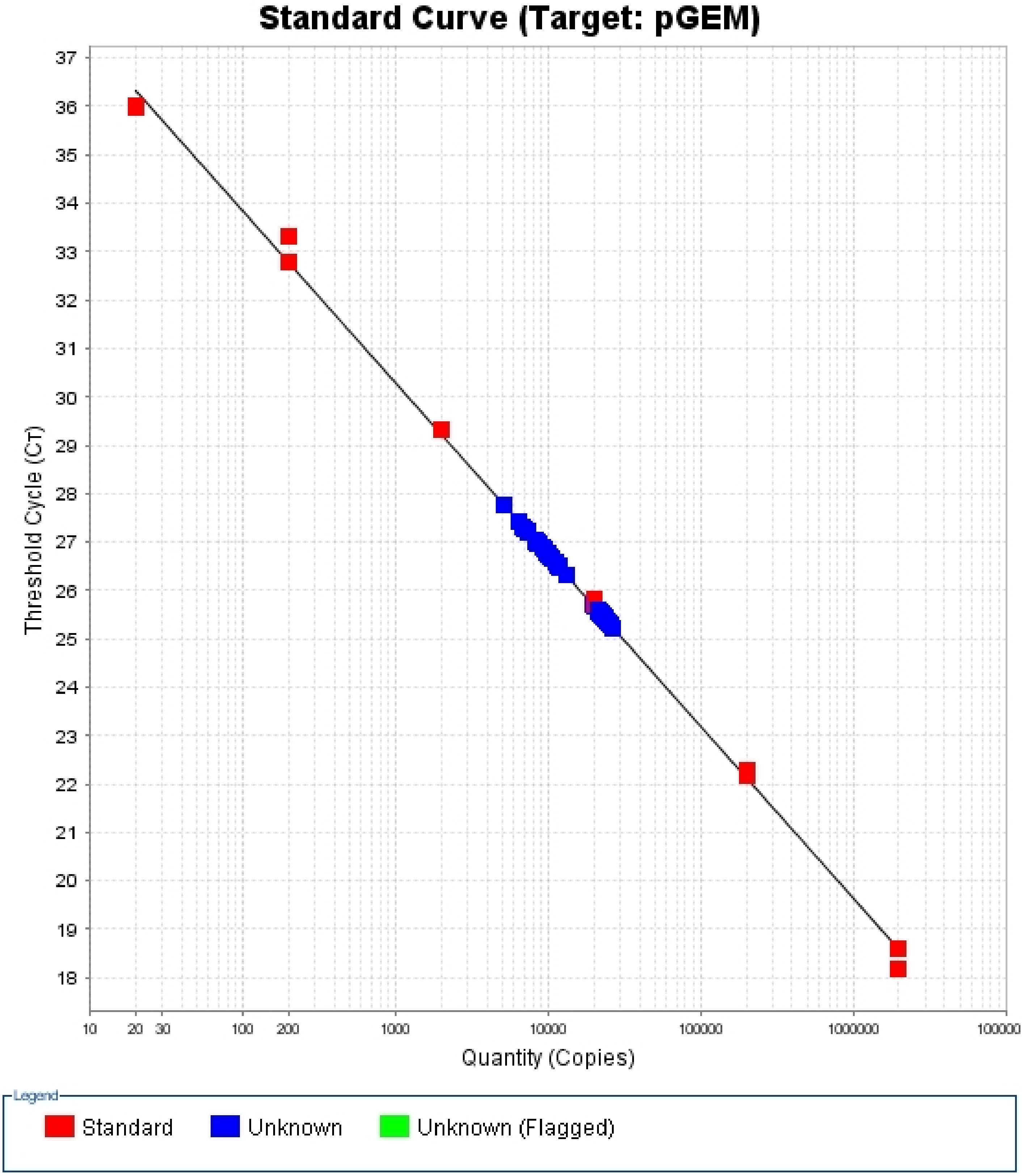

slope:-3.544 Y-Intercept:40.935 R^2:0.998 Eff%:91.496

Results Table

Well		Sample	Target	Task	Quantity	C
A1			pGEM	STANDARD	2000000	18.5871
A2			pGEM	STANDARD	2000000	18.1986
A3		2107 A11 10m	pGEM	UNKNOWN	9222.5244	26.8831
A4		2107 A11 10m	pGEM	UNKNOWN	10098.0322	26.7435
A5		2107 A11 10m	pGEM	UNKNOWN	8799.3652	26.9554
A6		2107 A11	pGEM	UNKNOWN	5203.1255	27.7641
A7		2107 A21 10m	pGEM	UNKNOWN	11714.1201	26.515
A8		2107 A21 10m	pGEM	UNKNOWN	11721.9326	26.514
A9		2107 A21 10m	pGEM	UNKNOWN	13457.1494	26.3015
A10		2107 A21	pGEM	UNKNOWN	6918.0278	27.3256
B1			pGEM	STANDARD	200000	22.2772
B2			pGEM	STANDARD	200000	22.1761
B3		2107 A11 10m	pGEM	UNKNOWN	24074.6133	25.4062
B4		2107 A11 10m	pGEM	UNKNOWN	26182.8848	25.277
B5		2107 A11 10m	pGEM	UNKNOWN	23869.9961	25.4194
B6		2107 A11	pGEM	UNKNOWN	6567.0332	27.4058
B7		2107 A21 10m	pGEM	UNKNOWN	21395.916	25.5878
B8		2107 A21 10m	pGEM	UNKNOWN	21236.6875	25.5993
B9		2107 A21 10m	pGEM	UNKNOWN	21957.4551	25.5479
B10		2107 A21	pGEM	UNKNOWN	8751.084	26.9638
C1			pGEM	STANDARD	20000	25.8361
C2			pGEM	STANDARD	20000	25.7236
C3		2107 A11 SCM	pGEM	UNKNOWN	8282.4385	27.0486
C4		2107 A11 SCM	pGEM	UNKNOWN	8816.1631	26.9524
C5		2107 A11 SCM	pGEM	UNKNOWN	8437.8633	27.0199
C6		2107 A11	pGEM	UNKNOWN	7466.938	27.2081
C7		2107 A21 SCM	pGEM	UNKNOWN	10348.5557	26.7058
C8		2107 A21 SCM	pGEM	UNKNOWN	9512.7686	26.8354
C9		2107 A21 SCM	pGEM	UNKNOWN	7138.5498	27.2773
C10		2107 A21	pGEM	UNKNOWN	8677.0078	26.9769

Well	Sample	Target	Task	Quantity	C
D1		pGEM	STANDARD	2000	29.3347
D2		pGEM	STANDARD	2000	29.3397
D3	2107 A11 SCM	pGEM	UNKNOWN	24676.2539	25.3682
D4	2107 A11 SCM	pGEM	UNKNOWN	25660.3418	25.308
D5	2107 A11 SCM	pGEM	UNKNOWN	23501.7598	25.4433
D6	2107 A11	pGEM	UNKNOWN	21849.4004	25.5555
D7	2107 A21 SCM	pGEM	UNKNOWN	21429.2969	25.5854
D8	2107 A21 SCM	pGEM	UNKNOWN	22096.9102	25.5382
D9	2107 A21 SCM	pGEM	UNKNOWN	21868.5234	25.5541
D10	2107 A21	pGEM	UNKNOWN	20037.7031	25.6887
E1		pGEM	STANDARD	200	32.7859
E2		pGEM	STANDARD	200	33.3061
E3	2107 A11 200m	pGEM	UNKNOWN	10439.4766	26.6923
E4	2107 A11 200m	pGEM	UNKNOWN	11259.5039	26.5759
E5	2107 A11 200m	pGEM	UNKNOWN	9838.8477	26.7835
E6	2107 A11	pGEM	UNKNOWN	20413.4941	25.6601
E7	2107 A21 200m	pGEM	UNKNOWN	9988.834	26.7602
E8	2107 A21 200m	pGEM	UNKNOWN	10278.5186	26.7162
E9	2107 A21 200m	pGEM	UNKNOWN	10584.0117	26.6711
E10	2107 A21	pGEM	UNKNOWN	19634.1289	25.72
F1		pGEM	STANDARD	20	35.9779
F2		pGEM	STANDARD	20	36.0196
F3	2107 A11 200m	pGEM	UNKNOWN	25480.9316	25.3188
F4	2107 A11 200m	pGEM	UNKNOWN	24213.2871	25.3974
F5	2107 A11 200m	pGEM	UNKNOWN	26933.9961	25.2335
F6	2107 A11	pGEM	UNKNOWN	21688.9199	25.5668
F7	2107 A21 200m	pGEM	UNKNOWN	23705.6875	25.43
F8	2107 A21 200m	pGEM	UNKNOWN	23183.0332	25.4643
F9	2107 A21 200m	pGEM	UNKNOWN	22417.7559	25.516
F10	2107 A21	pGEM	UNKNOWN	21616.4727	25.572
G3	2107 A11	pGEM	UNKNOWN	11978.9395	26.4806
G4	2107 A11	pGEM	UNKNOWN	11896.9688	26.4911

Well		Sample	Target	Task	Quantity	C
G5		2107 A11	pGEM	UNKNOWN	11262.8252	26.5755
G7		2107 A21	pGEM	UNKNOWN	7369.5156	27.2283
G8		2107 A21	pGEM	UNKNOWN	8586.0586	26.9931
G9		2107 A21	pGEM	UNKNOWN	7369.251	27.2284
H1			pGEM	NTC		Undetermined
H2			pGEM	NTC		Undetermined
H3		2107 A11	pGEM	UNKNOWN	21905.166	25.5516
H4		2107 A11	pGEM	UNKNOWN	22141.8066	25.535
H5		2107 A11	pGEM	UNKNOWN	22707.3828	25.4962
H7		2107 A21	pGEM	UNKNOWN	22908.7891	25.4826
H8		2107 A21	pGEM	UNKNOWN	23310.9668	25.4558
H9		2107 A21	pGEM	UNKNOWN	22128.9707	25.5359

QC Summary

Total Wells	96	Processed Wells	74	Targets Used	1
Well Setup	74	Flagged Wells	0	Samples Used	20

Flag	Name	Frequency	Locations
AMPNC	Amplification in negative control	0	
BADROX	Bad passive reference signal	0	
BLFAIL	Baseline algorithm failed	0	
CTFAIL	C algorithm failed	0	
EXPFAIL	Exponential algorithm failed	0	
HIGHSD	High standard deviation in replicate group	0	
NOAMP	No amplification	0	
NOISE	Noise higher than others in plate	0	
NOSIGNAL	No signal in well	0	
OFFSCALE	Fluorescence is offscale	0	
OUTLIERRG	Outlier in replicate group	0	
SPIKE	Noise spikes	0	
THOLDFAIL	Thresholding algorithm failed	0	
