# Supplementary material for: Ecological impact of algal-preying protists aplanochytrids (Labyrinthulea) as a source of docosahexaenoic acid for copepods in marine environments
Source: ISME Commun. 2026 Jun 26;6(1):ycag178. doi: 10.1093/ismeco/ycag178 (PMC13685785; doi:10.1093/ismeco/ycag178)
Supplement: Supplementary file 2 [file supplementary_materials_ycag178.zip › Morimoto_ISME_C_3rdrev_supplemental_data.zip!/Morimoto_ISME_C_3rdrev_supplemental_data/Supplemental_Data_2/Standard Curve/DNA recovery/2201/DnaRec2201AlineA01=A07N=1.pdf]

Experiment Results Report
230626DnaRec2201AlineA01-A07N=1

Experiment Summary

Experiment Name	:	230626DnaRec2201AlineA01-A07N=1
Experiment Type	:	Quantitation - Standard Curve
File Name	:	230626DnaRec2201AlineA01-A07N=1.eds
Run Started	:	2023 Jun 26 5:08:21 PM
Run Finished	:	2023 Jun 26 6:43:01 PM
Run Duration	:	94 minutes 40 seconds
Date Modified	:	2023 Jun 27 11:04:36 AM
User	:	
Number of wells used	:	82
Number of wells with results	:	82
Instrument Name	:	
Instrument Type	:	Applied Biosystems StepOnePlus™ Instrument
Comments	:	

Results Summary

Sample	Target	Quantity (Mean)	Quantity (Std Dev)	C (Mean)	C (Std Dev)
2201 A01 10m ato1	pGEM	37347.87	488.73	25.01	0.02
2201 A01 10m saki1	pGEM	25687.30	1459.61	25.58	0.09
2201 A01 SCM ato1	pGEM	39967.50	1124.13	24.90	0.04
2201 A01 SCM saki1	pGEM	29628.18	25.38	25.36	0.00
2201 A04 1000m	pGEM	41096.77	69.47	24.86	0.00
2201 A04 1000m	pGEM	18961.69	455.64	26.04	0.04
2201 A04 10m ato2	pGEM	4.59	2.99	38.98	0.97
2201 A04 10m saki2	pGEM	20712.24	1490.12	25.91	0.11
2201 A04 200m ato1	pGEM	37337.01	1443.31	25.01	0.06
2201 A04 200m	pGEM	22649.87	292.68	25.77	0.02
2201 A04 3000m	pGEM	38096.99	929.85	24.98	0.04
2201 A04 3000m	pGEM	21473.67	1272.35	25.85	0.09
2201 A04 SCM ato1	pGEM	35466.94	2145.03	25.09	0.09
2201 A04 SCM saki1	pGEM	25751.51	2565.11	25.58	0.15
2201 A07 1000m	pGEM	41893.25	498.96	24.83	0.02
2201 A07 1000m	pGEM	20323.28	773.80	25.94	0.06
2201 A07 200 ato1	pGEM	39716.09	3103.16	24.91	0.12
2201 A07 200 saki1	pGEM	28371.26	943.05	25.43	0.05
2201 A07 3000m	pGEM	44047.08	2395.44	24.75	0.08
2201 A07 3000m	pGEM	26733.70	3391.41	25.52	0.20
2201 A07 SCM ato1	pGEM	37441.69	2399.01	25.00	0.10
2201 A07 SCM saki1	pGEM	28173.13	1906.57	25.44	0.10
Comf2E4	pGEM	22416.41	2536.84	25.79	0.17

Plate Layout

	1	2	3	4	5	6	7	8	9	10	11	12
A	S pGEM<br>2E6<br>FAM-NFQ-MGB<br>CT: 18.91	S pGEM<br>2E6<br>FAM-NFQ-MGB<br>CT: 18.65	2201 A01 10m saki1<br>U pGEM<br>2.61E4<br>FAM-NFQ-MGB<br>CT: 25.55	2201 A01 10m saki1<br>U pGEM<br>2.69E4<br>FAM-NFQ-MGB<br>CT: 25.51	2201 A01 10m saki1<br>U pGEM<br>2.41E4<br>FAM-NFQ-MGB<br>CT: 25.68	2201 A04 1000m saki1<br>U pGEM<br>1.86E4<br>FAM-NFQ-MGB<br>CT: 26.07	2201 A04 1000m saki1<br>U pGEM<br>1.88E4<br>FAM-NFQ-MGB<br>CT: 26.06	2201 A04 1000m saki1<br>U pGEM<br>1.95E4<br>FAM-NFQ-MGB<br>CT: 26	2201 A07 1000m saki1<br>U pGEM<br>2.05E4<br>FAM-NFQ-MGB<br>CT: 25.92	2201 A07 1000m saki1<br>U pGEM<br>2.1E4<br>FAM-NFQ-MGB<br>CT: 25.89	2201 A07 1000m saki1<br>U pGEM<br>1.95E4<br>FAM-NFQ-MGB<br>CT: 26	
B	1<br>S pGEM<br>2E5<br>FAM-NFQ-MGB<br>CT: 23.01	1<br>S pGEM<br>2E5<br>FAM-NFQ-MGB<br>CT: 22.1	2201 A01 10m ato1<br>U pGEM<br>3.68E4<br>FAM-NFQ-MGB<br>CT: 25.03	2201 A01 10m ato1<br>U pGEM<br>3.77E4<br>FAM-NFQ-MGB<br>CT: 24.99	2201 A01 10m ato1<br>U pGEM<br>3.76E4<br>FAM-NFQ-MGB<br>CT: 25	2201 A04 1000m ato1<br>U pGEM<br>4.1E4<br>FAM-NFQ-MGB<br>CT: 24.86	2201 A04 1000m ato1<br>U pGEM<br>4.12E4<br>FAM-NFQ-MGB<br>CT: 24.86	2201 A04 1000m ato1<br>U pGEM<br>4.11E4<br>FAM-NFQ-MGB<br>CT: 24.86	2201 A07 1000m ato1<br>U pGEM<br>4.14E4<br>FAM-NFQ-MGB<br>CT: 24.85	2201 A07 1000m ato1<br>U pGEM<br>4.19E4<br>FAM-NFQ-MGB<br>CT: 24.83	2201 A07 1000m ato1<br>U pGEM<br>4.24E4<br>FAM-NFQ-MGB<br>CT: 24.81	
C	S pGEM<br>2E4<br>FAM-NFQ-MGB<br>CT: 26.04	S pGEM<br>2E4<br>FAM-NFQ-MGB<br>CT: 25.77	2201 A01 SCM saki1<br>U pGEM<br>2.96E4<br>FAM-NFQ-MGB<br>CT: 25.36	2201 A01 SCM saki1<br>U pGEM<br>2.96E4<br>FAM-NFQ-MGB<br>CT: 25.36	2201 A01 SCM saki1<br>U pGEM<br>2.96E4<br>FAM-NFQ-MGB<br>CT: 25.36	2201 A04 3000m saki1<br>U pGEM<br>2.28E4<br>FAM-NFQ-MGB<br>CT: 25.76	2201 A04 3000m saki1<br>U pGEM<br>2.14E4<br>FAM-NFQ-MGB<br>CT: 25.86	2201 A04 3000m saki1<br>U pGEM<br>2.03E4<br>FAM-NFQ-MGB<br>CT: 25.94	2201 A07 3000m saki1<br>U pGEM<br>2.28E4<br>FAM-NFQ-MGB<br>CT: 25.76	2201 A07 3000m saki1<br>U pGEM<br>2.85E4<br>FAM-NFQ-MGB<br>CT: 25.42	2201 A07 3000m saki1<br>U pGEM<br>2.89E4<br>FAM-NFQ-MGB<br>CT: 25.4	
D	S pGEM<br>2E3<br>FAM-NFQ-MGB<br>CT: 29.32	S pGEM<br>2E3<br>FAM-NFQ-MGB<br>CT: 29.84	2201 A01 SCM ato1<br>U pGEM<br>3.87E4<br>FAM-NFQ-MGB<br>CT: 24.95	2201 A01 SCM ato1<br>U pGEM<br>4.04E4<br>FAM-NFQ-MGB<br>CT: 24.88	2201 A01 SCM ato1<br>U pGEM<br>4.08E4<br>FAM-NFQ-MGB<br>CT: 24.87	2201 A04 3000m ato1<br>U pGEM<br>3.79E4<br>FAM-NFQ-MGB<br>CT: 24.98	2201 A04 3000m ato1<br>U pGEM<br>3.91E4<br>FAM-NFQ-MGB<br>CT: 24.93	2201 A04 3000m ato1<br>U pGEM<br>3.73E4<br>FAM-NFQ-MGB<br>CT: 25.01	2201 A07 3000m ato1<br>U pGEM<br>4.19E4<br>FAM-NFQ-MGB<br>CT: 24.83	2201 A07 3000m ato1<br>U pGEM<br>4.36E4<br>FAM-NFQ-MGB<br>CT: 24.77	2201 A07 3000m ato1<br>U pGEM<br>4.66E4<br>FAM-NFQ-MGB<br>CT: 24.67	
E	S pGEM<br>200<br>FAM-NFQ-MGB<br>CT: 32.9	S pGEM<br>200<br>FAM-NFQ-MGB<br>CT: 33.53	2201 A04 SCM saki1<br>U pGEM<br>2.87E4<br>FAM-NFQ-MGB<br>CT: 25.41	2201 A04 SCM saki1<br>U pGEM<br>2.46E4<br>FAM-NFQ-MGB<br>CT: 25.64	2201 A04 SCM saki1<br>U pGEM<br>2.39E4<br>FAM-NFQ-MGB<br>CT: 25.69	2201 A07 SCM saki1<br>U pGEM<br>3.04E4<br>FAM-NFQ-MGB<br>CT: 25.32	2201 A07 SCM saki1<br>U pGEM<br>2.72E4<br>FAM-NFQ-MGB<br>CT: 25.49	2201 A07 SCM saki1<br>U pGEM<br>2.7E4<br>FAM-NFQ-MGB<br>CT: 25.5	2201 A04 10m saki2<br>U pGEM<br>2.22E4<br>FAM-NFQ-MGB<br>CT: 25.8	2201 A04 10m saki2<br>U pGEM<br>2.07E4<br>FAM-NFQ-MGB<br>CT: 25.91	2201 A04 10m saki2<br>U pGEM<br>1.92E4<br>FAM-NFQ-MGB<br>CT: 26.02	
F	S pGEM<br>20<br>FAM-NFQ-MGB<br>CT: 36.01	S pGEM<br>20<br>FAM-NFQ-MGB<br>CT: 36.54	2201 A04 SCM ato1<br>U pGEM<br>3.71E4<br>FAM-NFQ-MGB<br>CT: 25.02	2201 A04 SCM ato1<br>U pGEM<br>3.3E4<br>FAM-NFQ-MGB<br>CT: 25.19	2201 A04 SCM ato1<br>U pGEM<br>3.63E4<br>FAM-NFQ-MGB<br>CT: 25.05	2201 A07 SCM ato1<br>U pGEM<br>3.5E4<br>FAM-NFQ-MGB<br>CT: 25.11	2201 A07 SCM ato1<br>U pGEM<br>3.98E4<br>FAM-NFQ-MGB<br>CT: 24.91	2201 A07 SCM ato1<br>U pGEM<br>3.76E4<br>FAM-NFQ-MGB<br>CT: 25	1<br>2201 A04 10m ato2<br>U pGEM<br>7.97<br>FAM-NFQ-MGB<br>CT: 37.92	1<br>2201 A04 10m ato2<br>U pGEM<br>2.29<br>FAM-NFQ-MGB<br>CT: 39.83	1<br>2201 A04 10m ato2<br>U pGEM<br>3.51<br>FAM-NFQ-MGB<br>CT: 39.17	
G			2201 A04 200m saki1<br>U pGEM<br>2.23E4<br>FAM-NFQ-MGB<br>CT: 25.79	2201 A04 200m saki1<br>U pGEM<br>2.28E4<br>FAM-NFQ-MGB<br>CT: 25.76	2201 A04 200m saki1<br>U pGEM<br>2.28E4<br>FAM-NFQ-MGB<br>CT: 25.76	2201 A07 200 saki1<br>U pGEM<br>2.93E4<br>FAM-NFQ-MGB<br>CT: 25.38	2201 A07 200 saki1<br>U pGEM<br>2.74E4<br>FAM-NFQ-MGB<br>CT: 25.48	2201 A07 200 saki1<br>U pGEM<br>2.84E4<br>FAM-NFQ-MGB<br>CT: 25.42				N pGEM<br>FAM-NFQ-MGB<br>CT: Undetermined
H	Comf2E4<br>U pGEM<br>2.06E4<br>FAM-NFQ-MGB<br>CT: 25.91	Comf2E4<br>U pGEM<br>2.42E4<br>FAM-NFQ-MGB<br>CT: 25.67	2201 A04 200m ato1<br>U pGEM<br>3.89E4<br>FAM-NFQ-MGB<br>CT: 24.94	2201 A04 200m ato1<br>U pGEM<br>3.7E4<br>FAM-NFQ-MGB<br>CT: 25.02	2201 A04 200m ato1<br>U pGEM<br>3.61E4<br>FAM-NFQ-MGB<br>CT: 25.06	2201 A07 200 ato1<br>U pGEM<br>4.32E4<br>FAM-NFQ-MGB<br>CT: 24.78	2201 A07 200 ato1<br>U pGEM<br>3.72E4<br>FAM-NFQ-MGB<br>CT: 25.01	2201 A07 200 ato1<br>U pGEM<br>3.87E4<br>FAM-NFQ-MGB<br>CT: 24.95				N pGEM<br>FAM-NFQ-MGB<br>CT: Undetermined

Standard Curves

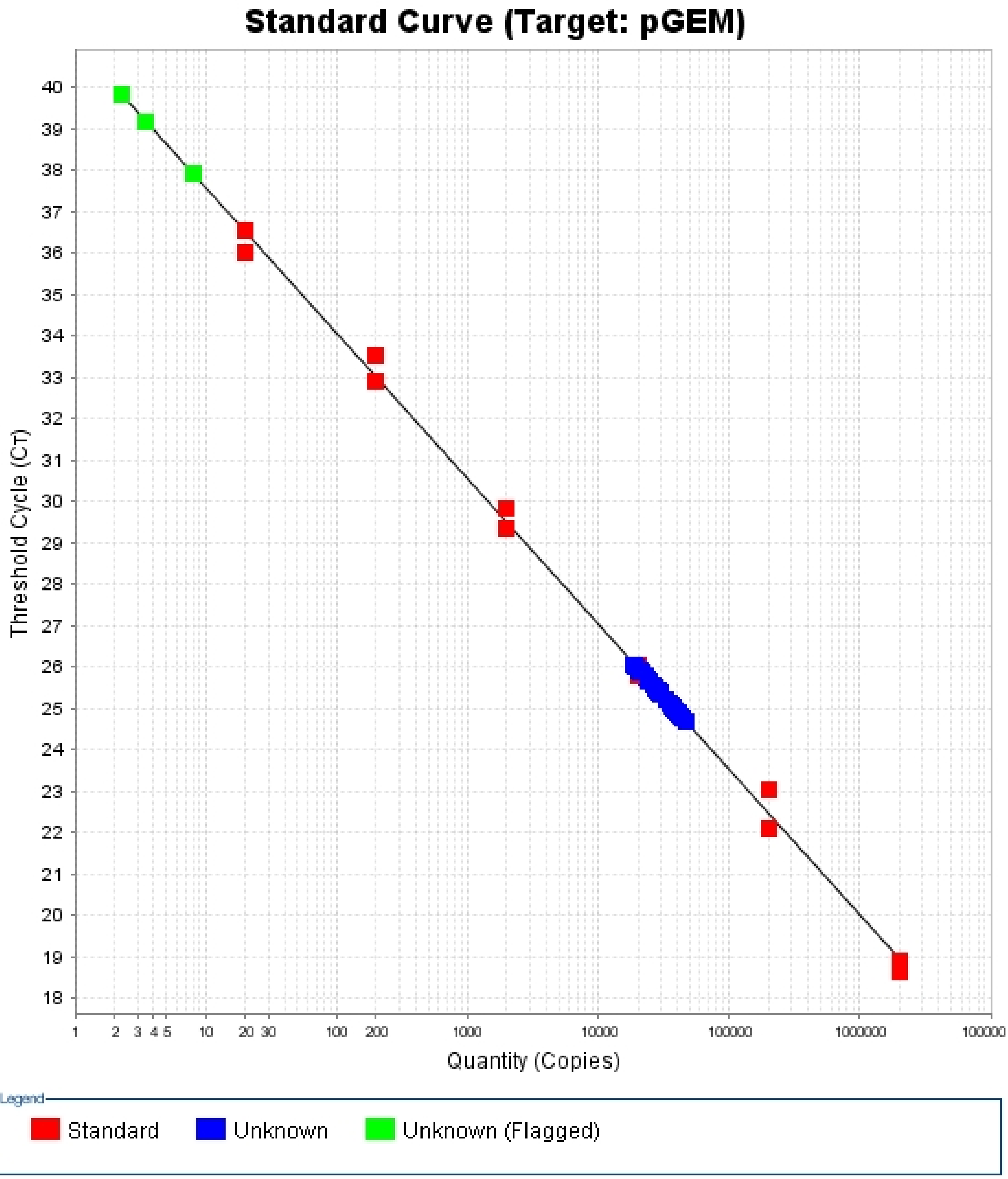

slope:-3.519 | Y-Intercept:41.094 | R^2:0.997 | Eff%:92.392

Results Table

Well		Sample	Target	Task	Quantity	C
A1			pGEM	STANDARD	2000000	18.9054
A2			pGEM	STANDARD	2000000	18.6451
A3		2201 A01 10m	pGEM	UNKNOWN	26069.127	25.5546
A4		2201 A01 10m	pGEM	UNKNOWN	26918.0449	25.5057
A5		2201 A01 10m	pGEM	UNKNOWN	24074.7266	25.6763
A6		2201 A04	pGEM	UNKNOWN	18630.6484	26.068
A7		2201 A04	pGEM	UNKNOWN	18773.0566	26.0564
A8		2201 A04	pGEM	UNKNOWN	19481.3535	25.9998
A9		2201 A07	pGEM	UNKNOWN	20503.5664	25.9216
A10		2201 A07	pGEM	UNKNOWN	20991.0137	25.8857
A11		2201 A07	pGEM	UNKNOWN	19475.25	26.0003
B1			pGEM	STANDARD	200000	23.0079
B2			pGEM	STANDARD	200000	22.1022
B3		2201 A01 10m	pGEM	UNKNOWN	36786.6094	25.0284
B4		2201 A01 10m	pGEM	UNKNOWN	37679.5039	24.9917
B5		2201 A01 10m	pGEM	UNKNOWN	37577.4961	24.9958
B6		2201 A04	pGEM	UNKNOWN	41018.5703	24.8619
B7		2201 A04	pGEM	UNKNOWN	41151.3281	24.857
B8		2201 A04	pGEM	UNKNOWN	41120.4219	24.8582
B9		2201 A07	pGEM	UNKNOWN	41373.8594	24.8488
B10		2201 A07	pGEM	UNKNOWN	41937.0117	24.8281
B11		2201 A07	pGEM	UNKNOWN	42368.8945	24.8124
C1			pGEM	STANDARD	20000	26.0434
C2			pGEM	STANDARD	20000	25.772
C3		2201 A01 SCM	pGEM	UNKNOWN	29645.6113	25.3582
C4		2201 A01 SCM	pGEM	UNKNOWN	29599.0625	25.3606
C5		2201 A01 SCM	pGEM	UNKNOWN	29639.877	25.3585
C6		2201 A04	pGEM	UNKNOWN	22795.8379	25.7597
C7		2201 A04	pGEM	UNKNOWN	21367.3418	25.8586
C8		2201 A04	pGEM	UNKNOWN	20257.8203	25.9401

Well	Sample	Target	Task	Quantity	C
C9	2201 A07	pGEM	UNKNOWN	22824.877	25.7577
C10	2201 A07	pGEM	UNKNOWN	28482.002	25.4194
C11	2201 A07	pGEM	UNKNOWN	28894.2266	25.3974
D1		pGEM	STANDARD	2000	29.3236
D2		pGEM	STANDARD	2000	29.843
D3	2201 A01 SCM	pGEM	UNKNOWN	38689.1758	24.9513
D4	2201 A01 SCM	pGEM	UNKNOWN	40411.5742	24.8847
D5	2201 A01 SCM	pGEM	UNKNOWN	40801.7695	24.87
D6	2201 A04	pGEM	UNKNOWN	37852.9648	24.9847
D7	2201 A04	pGEM	UNKNOWN	39124.5117	24.9342
D8	2201 A04	pGEM	UNKNOWN	37313.4883	25.0066
D9	2201 A07	pGEM	UNKNOWN	41921.4688	24.8287
D10	2201 A07	pGEM	UNKNOWN	43577.0977	24.7695
D11	2201 A07	pGEM	UNKNOWN	46642.6836	24.6656
E1		pGEM	STANDARD	200	32.8992
E2		pGEM	STANDARD	200	33.534
E3	2201 A04 SCM	pGEM	UNKNOWN	28684.2402	25.4085
E4	2201 A04 SCM	pGEM	UNKNOWN	24644.459	25.6405
E5	2201 A04 SCM	pGEM	UNKNOWN	23925.8203	25.6857
E6	2201 A07 SCM	pGEM	UNKNOWN	30370.2539	25.3213
E7	2201 A07 SCM	pGEM	UNKNOWN	27195.0176	25.49
E8	2201 A07 SCM	pGEM	UNKNOWN	26954.1172	25.5036
E9	2201 A04 10m	pGEM	UNKNOWN	22209.7461	25.7995
E10	2201 A04 10m	pGEM	UNKNOWN	20697.3535	25.9073
E11	2201 A04 10m	pGEM	UNKNOWN	19229.6094	26.0197
F1		pGEM	STANDARD	20	36.0099
F2		pGEM	STANDARD	20	36.5406
F3	2201 A04 SCM	pGEM	UNKNOWN	37064.375	25.0169
F4	2201 A04 SCM	pGEM	UNKNOWN	33028.9258	25.193
F5	2201 A04 SCM	pGEM	UNKNOWN	36307.5273	25.0484
F6	2201 A07 SCM	pGEM	UNKNOWN	34984.5039	25.1051
F7	2201 A07 SCM	pGEM	UNKNOWN	39777.9492	24.9089

Well		Sample	Target	Task	Quantity	C
F8		2201 A07 SCM	pGEM	UNKNOWN	37562.6328	24.9964
F9		2201 A04 10m	pGEM	UNKNOWN	7.9697	37.9221
F10		2201 A04 10m	pGEM	UNKNOWN	2.2893	39.8284
F11		2201 A04 10m	pGEM	UNKNOWN	3.5112	39.1748
G3		2201 A04 200m	pGEM	UNKNOWN	22312.6035	25.7924
G4		2201 A04 200m	pGEM	UNKNOWN	22799.8203	25.7594
G5		2201 A04 200m	pGEM	UNKNOWN	22837.1875	25.7569
G6		2201 A07 200	pGEM	UNKNOWN	29289.9258	25.3766
G7		2201 A07 200	pGEM	UNKNOWN	27405.5918	25.4782
G8		2201 A07 200	pGEM	UNKNOWN	28418.2637	25.4228
G12			pGEM	NTC		Undetermined
H1		Comf2E4	pGEM	UNKNOWN	20622.5996	25.9128
H2		Comf2E4	pGEM	UNKNOWN	24210.2305	25.6677
H3		2201 A04 200m	pGEM	UNKNOWN	38922.8711	24.9421
H4		2201 A04 200m	pGEM	UNKNOWN	36987.8477	25.02
H5		2201 A04 200m	pGEM	UNKNOWN	36100.3047	25.0571
H6		2201 A07 200	pGEM	UNKNOWN	43190.4883	24.7831
H7		2201 A07 200	pGEM	UNKNOWN	37219.9492	25.0105
H8		2201 A07 200	pGEM	UNKNOWN	38737.832	24.9494
H12			pGEM	NTC		Undetermined

QC Summary

Total Wells	96	Processed Wells	82	Targets Used	1
Well Setup	82	Flagged Wells	5	Samples Used	23

Flag	Name	Frequency	Locations
AMPNC	Amplification in negative control	0	
BADROX	Bad passive reference signal	0	
BLFAIL	Baseline algorithm failed	0	
CTFAIL	C algorithm failed	0	
EXPFAIL	Exponential algorithm failed	0	
HIGHSD	High standard deviation in replicate group	5	B1, B2, F9, F10, F11
NOAMP	No amplification	0	
NOISE	Noise higher than others in plate	0	
NOSIGNAL	No signal in well	0	
OFFSCALE	Fluorescence is offscale	0	
OUTLIERRG	Outlier in replicate group	0	
SPIKE	Noise spikes	0	
THOLDFAIL	Thresholding algorithm failed	0	
