# Supplementary material for: Ecological impact of algal-preying protists aplanochytrids (Labyrinthulea) as a source of docosahexaenoic acid for copepods in marine environments
Source: ISME Commun. 2026 Jun 26;6(1):ycag178. doi: 10.1093/ismeco/ycag178 (PMC13685785; doi:10.1093/ismeco/ycag178)
Supplement: Supplementary file 2 [file supplementary_materials_ycag178.zip › Morimoto_ISME_C_3rdrev_supplemental_data.zip!/Morimoto_ISME_C_3rdrev_supplemental_data/Supplemental_Data_2/Standard Curve/DNA recovery/2201/DnaRecALine2201A11-A21N=1.pdf]

# Experiment Results Report

## 230627ALine2201A11-A21N=1

## Experiment Summary

| | | |
|---|---|---|
| Experiment Name | : | 230627ALine2201A11-A21N=1 |
| Experiment Type | : | Quantitation - Standard Curve |
| File Name | : | 230627ALine2201A11-A21N=1.eds |
| Run Started | : | 2023 Jun 27 12:15:47 PM |
| Run Finished | : | 2023 Jun 27 1:50:25 PM |
| Run Duration | : | 94 minutes 38 seconds |
| Date Modified | : | 2023 Jun 27 2:11:09 PM |
| User | : | |
| Number of wells used | : | 64 |
| Number of wells with results | : | 64 |
| Instrument Name | : | |
| Instrument Type | : | Applied Biosystems StepOnePlus™ Instrument |
| Comments | : | |

# Results Summary

| Sample | Target | Quantity (Mean) | Quantity (Std Dev) | C (Mean) | C (Std Dev) |
|---|---|---|---|---|---|
| 2201 A011 200m | pGEM | 23685.89 | 505.87 | 25.58 | 0.03 |
| 2201 A011SCM | pGEM | 11794.43 | 1690.27 | 26.61 | 0.20 |
| 2201 A11 1000m | pGEM | 25776.07 | 10727.93 | 25.53 | 0.57 |
| 2201 A11 1000m | pGEM | 11289.07 | 3521.47 | 26.71 | 0.43 |
| 2201 A11 200m | pGEM | 10369.77 | 923.78 | 26.80 | 0.13 |
| 2201 A11 3000m | pGEM | 19309.30 | 1218.18 | 25.88 | 0.09 |
| 2201 A11 3000m | pGEM | 8436.16 | 1217.29 | 27.11 | 0.21 |
| 2201 A11 SCM ato1 | pGEM | 19149.26 | 778.05 | 25.89 | 0.06 |
| 2201 A21 1000m | pGEM | 24974.48 | 3797.35 | 25.51 | 0.23 |
| 2201 A21 1000m | pGEM | 6765.17 | 387.70 | 27.42 | 0.08 |
| 2201 A21 200 ato1 | pGEM | 20630.97 | 1302.39 | 25.78 | 0.09 |
| 2201 A21 200 saki1 | pGEM | 8856.02 | 555.61 | 27.03 | 0.09 |
| 2201 A21 3000m | pGEM | 20468.84 | 1930.60 | 25.79 | 0.14 |
| 2201 A21 3000m | pGEM | 10798.02 | 9127.73 | 27.06 | 1.15 |
| 2201 A21 SCM ato1 | pGEM | 20549.24 | 759.80 | 25.79 | 0.05 |
| 2201 A21 SCM saki1 | pGEM | 8034.66 | 483.53 | 27.17 | 0.09 |
| Comf2E4 | pGEM | 11976.90 | 1142.94 | 26.58 | 0.14 |

# Plate Layout

| | 1 | 2 | 3 | 4 | 5 | 6 | 7 | 8 | 9 | 10 | 11 | 12 |
|---|---|---|---|---|---|---|---|---|---|---|---|---|
| A | S pGEM<br>2E6<br>FAM-NFQ-MGB<br>CT: 19.22 | S pGEM<br>2E6<br>FAM-NFQ-MGB<br>CT: 18.98 | 2201 A011SCM saki1<br>U pGEM<br>1.37E4<br>FAM-NFQ-MGB<br>CT: 26.38 | 2201 A011SCM saki1<br>U pGEM<br>1.05E4<br>FAM-NFQ-MGB<br>CT: 26.77 | 2201 A011SCM saki1<br>U pGEM<br>1.11E4<br>FAM-NFQ-MGB<br>CT: 26.69 | 2201 A21 SCM saki1<br>U pGEM<br>7.8E3<br>FAM-NFQ-MGB<br>CT: 27.21 | 2201 A21 SCM saki1<br>U pGEM<br>8.59E3<br>FAM-NFQ-MGB<br>CT: 27.07 | 2201 A21 SCM saki1<br>U pGEM<br>7.72E3<br>FAM-NFQ-MGB<br>CT: 27.23 | | | | |
| B | S pGEM<br>2E5<br>FAM-NFQ-MGB<br>CT: 22.34 | S pGEM<br>2E5<br>FAM-NFQ-MGB<br>CT: 22.28 | 2201 A11 SCM ato1<br>U pGEM<br>1.96E4<br>FAM-NFQ-MGB<br>CT: 25.85 | 2201 A11 SCM ato1<br>U pGEM<br>1.96E4<br>FAM-NFQ-MGB<br>CT: 25.85 | 2201 A11 SCM ato1<br>U pGEM<br>1.83E4<br>FAM-NFQ-MGB<br>CT: 25.96 | 2201 A21 SCM ato1<br>U pGEM<br>2.04E4<br>FAM-NFQ-MGB<br>CT: 25.8 | 2201 A21 SCM ato1<br>U pGEM<br>1.99E4<br>FAM-NFQ-MGB<br>CT: 25.83 | 2201 A21 SCM ato1<br>U pGEM<br>2.14E4<br>FAM-NFQ-MGB<br>CT: 25.73 | | | | |
| C | S pGEM<br>2E4<br>FAM-NFQ-MGB<br>CT: 25.86 | S pGEM<br>2E4<br>FAM-NFQ-MGB<br>CT: 25.71 | 2201 A11 200m saki1<br>U pGEM<br>9.38E3<br>FAM-NFQ-MGB<br>CT: 26.94 | 2201 A11 200m saki1<br>U pGEM<br>1.12E4<br>FAM-NFQ-MGB<br>CT: 26.68 | 2201 A11 200m saki1<br>U pGEM<br>1.05E4<br>FAM-NFQ-MGB<br>CT: 26.77 | 2201 A21 200 saki1<br>U pGEM<br>8.3E3<br>FAM-NFQ-MGB<br>CT: 27.12 | 2201 A21 200 saki1<br>U pGEM<br>9.41E3<br>FAM-NFQ-MGB<br>CT: 26.94 | 2201 A21 200 saki1<br>U pGEM<br>8.86E3<br>FAM-NFQ-MGB<br>CT: 27.03 | | | | |
| D | S pGEM<br>2E3<br>FAM-NFQ-MGB<br>CT: 29.31 | S pGEM<br>2E3<br>FAM-NFQ-MGB<br>CT: 29.37 | 2201 A011 200m ato1<br>U pGEM<br>2.31E4<br>FAM-NFQ-MGB<br>CT: 25.61 | 2201 A011 200m ato1<br>U pGEM<br>2.41E4<br>FAM-NFQ-MGB<br>CT: 25.55 | 2201 A011 200m ato1<br>U pGEM<br>2.38E4<br>FAM-NFQ-MGB<br>CT: 25.57 | 2201 A21 200 ato1<br>U pGEM<br>2.05E4<br>FAM-NFQ-MGB<br>CT: 25.79 | 2201 A21 200 ato1<br>U pGEM<br>2.2E4<br>FAM-NFQ-MGB<br>CT: 25.68 | 2201 A21 200 ato1<br>U pGEM<br>1.94E4<br>FAM-NFQ-MGB<br>CT: 25.87 | | | | |
| E | S pGEM<br>200<br>FAM-NFQ-MGB<br>CT: 32.62 | S pGEM<br>200<br>FAM-NFQ-MGB<br>CT: 32.67 | 2201 A11 1000m saki1<br>U pGEM<br>9.53E3<br>FAM-NFQ-MGB<br>CT: 26.92 | 2201 A11 1000m saki1<br>U pGEM<br>8.99E3<br>FAM-NFQ-MGB<br>CT: 27 | 2201 A11 1000m saki1<br>U pGEM<br>1.53E4<br>FAM-NFQ-MGB<br>CT: 26.22 | 2201 A21 1000m saki1<br>U pGEM<br>6.36E3<br>FAM-NFQ-MGB<br>CT: 27.51 | 2201 A21 1000m saki1<br>U pGEM<br>7.14E3<br>FAM-NFQ-MGB<br>CT: 27.34 | 2201 A21 1000m saki1<br>U pGEM<br>6.8E3<br>FAM-NFQ-MGB<br>CT: 27.42 | | | | |
| F | S pGEM<br>20<br>FAM-NFQ-MGB<br>CT: 35.91 | S pGEM<br>20<br>FAM-NFQ-MGB<br>CT: 35.98 | ⚠1<br>2201 A11 1000m ato1<br>U pGEM<br>3.82E4<br>FAM-NFQ-MGB<br>CT: 24.87 | ⚠1<br>2201 A11 1000m ato1<br>U pGEM<br>1.91E4<br>FAM-NFQ-MGB<br>CT: 25.89 | ⚠1<br>2201 A11 1000m ato1<br>U pGEM<br>2E4<br>FAM-NFQ-MGB<br>CT: 25.82 | 2201 A21 1000m ato1<br>U pGEM<br>2.1E4<br>FAM-NFQ-MGB<br>CT: 25.76 | 2201 A21 1000m ato1<br>U pGEM<br>2.85E4<br>FAM-NFQ-MGB<br>CT: 25.3 | 2201 A21 1000m ato1<br>U pGEM<br>2.55E4<br>FAM-NFQ-MGB<br>CT: 25.47 | | | | |
| G | | | 2201 A11 3000m saki1<br>U pGEM<br>9.81E3<br>FAM-NFQ-MGB<br>CT: 26.87 | 2201 A11 3000m saki1<br>U pGEM<br>7.98E3<br>FAM-NFQ-MGB<br>CT: 27.18 | 2201 A11 3000m saki1<br>U pGEM<br>7.51E3<br>FAM-NFQ-MGB<br>CT: 27.27 | ⚠1<br>2201 A21 3000m saki1<br>U pGEM<br>2.13E4<br>FAM-NFQ-MGB<br>CT: 25.73 | ⚠1<br>2201 A21 3000m saki1<br>U pGEM<br>5.36E3<br>FAM-NFQ-MGB<br>CT: 27.77 | ⚠1<br>2201 A21 3000m saki1<br>U pGEM<br>5.7E3<br>FAM-NFQ-MGB<br>CT: 27.67 | | | | N pGEM<br>FAM-NFQ-MGB<br>CT: Undetermined |
| H | Comf2E4<br>U pGEM<br>1.12E4<br>FAM-NFQ-MGB<br>CT: 26.68 | Comf2E4<br>U pGEM<br>1.28E4<br>FAM-NFQ-MGB<br>CT: 26.48 | 2201 A11 3000m ato1<br>U pGEM<br>2.05E4<br>FAM-NFQ-MGB<br>CT: 25.79 | 2201 A11 3000m ato1<br>U pGEM<br>1.94E4<br>FAM-NFQ-MGB<br>CT: 25.87 | 2201 A11 3000m ato1<br>U pGEM<br>1.81E4<br>FAM-NFQ-MGB<br>CT: 25.97 | 2201 A21 3000m ato1<br>U pGEM<br>1.88E4<br>FAM-NFQ-MGB<br>CT: 25.92 | 2201 A21 3000m ato1<br>U pGEM<br>2.01E4<br>FAM-NFQ-MGB<br>CT: 25.82 | 2201 A21 3000m ato1<br>U pGEM<br>2.26E4<br>FAM-NFQ-MGB<br>CT: 25.65 | | | | N pGEM<br>FAM-NFQ-MGB<br>CT: Undetermined |

# Standard Curves

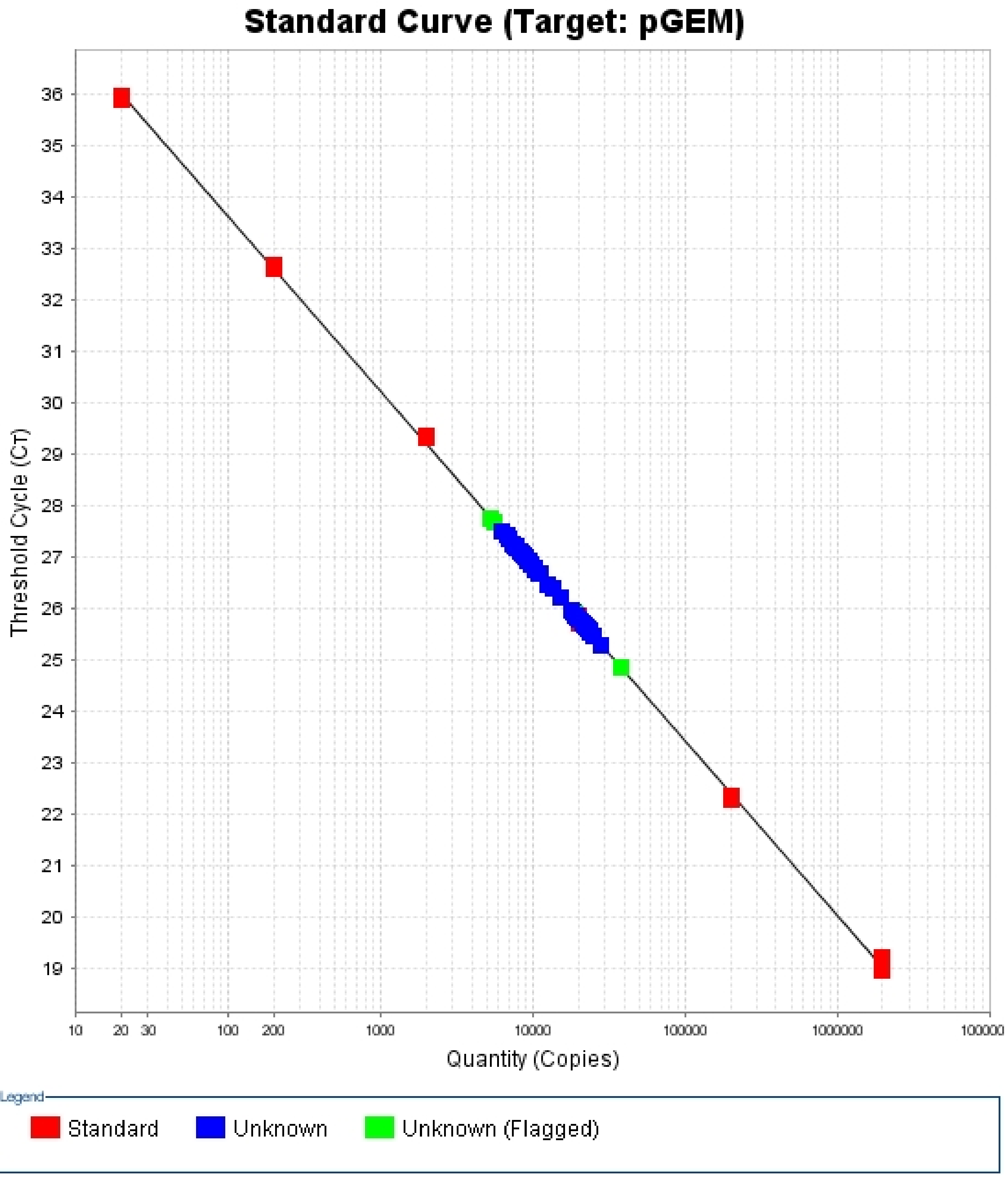


slope:-3.394 Y-Intercept:40.422 $R^2$:1.0 Eff%:97.078

# Results Table

| Well | | Sample | Target | Task | Quantity | C |
|---|---|---|---|---|---|---|
| A1 | | | pGEM | STANDARD | 2000000 | 19.2174 |
| A2 | | | pGEM | STANDARD | 2000000 | 18.9833 |
| A3 | | 2201 A011SCM | pGEM | UNKNOWN | 13718.0039 | 26.3801 |
| A4 | | 2201 A011SCM | pGEM | UNKNOWN | 10546.4355 | 26.7677 |
| A5 | | 2201 A011SCM | pGEM | UNKNOWN | 11118.8584 | 26.6898 |
| A6 | | 2201 A21 SCM | pGEM | UNKNOWN | 7797.4624 | 27.2128 |
| A7 | | 2201 A21 SCM | pGEM | UNKNOWN | 8590.9766 | 27.07 |
| A8 | | 2201 A21 SCM | pGEM | UNKNOWN | 7715.5322 | 27.2284 |
| B1 | | | pGEM | STANDARD | 200000 | 22.342 |
| B2 | | | pGEM | STANDARD | 200000 | 22.2786 |
| B3 | | 2201 A11 SCM | pGEM | UNKNOWN | 19591.9062 | 25.8548 |
| B4 | | 2201 A11 SCM | pGEM | UNKNOWN | 19604.9922 | 25.8538 |
| B5 | | 2201 A11 SCM | pGEM | UNKNOWN | 18250.8789 | 25.9593 |
| B6 | | 2201 A21 SCM | pGEM | UNKNOWN | 20370.8457 | 25.7973 |
| B7 | | 2201 A21 SCM | pGEM | UNKNOWN | 19894.5137 | 25.8322 |
| B8 | | 2201 A21 SCM | pGEM | UNKNOWN | 21382.3613 | 25.7259 |
| C1 | | | pGEM | STANDARD | 20000 | 25.8565 |
| C2 | | | pGEM | STANDARD | 20000 | 25.7113 |
| C3 | | 2201 A11 200m | pGEM | UNKNOWN | 9383.6816 | 26.9399 |
| C4 | | 2201 A11 200m | pGEM | UNKNOWN | 11215.0957 | 26.6771 |
| C5 | | 2201 A11 200m | pGEM | UNKNOWN | 10510.5234 | 26.7727 |
| C6 | | 2201 A21 200 | pGEM | UNKNOWN | 8299.9707 | 27.1208 |
| C7 | | 2201 A21 200 | pGEM | UNKNOWN | 9411.1885 | 26.9356 |
| C8 | | 2201 A21 200 | pGEM | UNKNOWN | 8856.8965 | 27.025 |
| D1 | | | pGEM | STANDARD | 2000 | 29.312 |
| D2 | | | pGEM | STANDARD | 2000 | 29.3718 |
| D3 | | 2201 A011 | pGEM | UNKNOWN | 23123.2598 | 25.6105 |
| D4 | | 2201 A011 | pGEM | UNKNOWN | 24103.1855 | 25.5493 |
| D5 | | 2201 A011 | pGEM | UNKNOWN | 23831.2305 | 25.5661 |
| D6 | | 2201 A21 200 | pGEM | UNKNOWN | 20503.6035 | 25.7877 |

| Well | | Sample | Target | Task | Quantity | C |
|---|---|---|---|---|---|---|
| D7 | | 2201 A21 200 | pGEM | UNKNOWN | 21992.3633 | 25.6844 |
| D8 | | 2201 A21 200 | pGEM | UNKNOWN | 19396.9395 | 25.8695 |
| E1 | | | pGEM | STANDARD | 200 | 32.6219 |
| E2 | | | pGEM | STANDARD | 200 | 32.669 |
| E3 | | 2201 A11 | pGEM | UNKNOWN | 9532.1445 | 26.9167 |
| E4 | | 2201 A11 | pGEM | UNKNOWN | 8991.7412 | 27.0028 |
| E5 | | 2201 A11 | pGEM | UNKNOWN | 15343.3193 | 26.2151 |
| E6 | | 2201 A21 | pGEM | UNKNOWN | 6362.5112 | 27.5126 |
| E7 | | 2201 A21 | pGEM | UNKNOWN | 7135.9395 | 27.3435 |
| E8 | | 2201 A21 | pGEM | UNKNOWN | 6797.0645 | 27.4152 |
| F1 | | | pGEM | STANDARD | 20 | 35.9144 |
| F2 | | | pGEM | STANDARD | 20 | 35.9775 |
| F3 | | 2201 A11 | pGEM | UNKNOWN | 38152.6289 | 24.8724 |
| F4 | | 2201 A11 | pGEM | UNKNOWN | 19136.2148 | 25.8895 |
| F5 | | 2201 A11 | pGEM | UNKNOWN | 20039.3555 | 25.8215 |
| F6 | | 2201 A21 | pGEM | UNKNOWN | 20959.4492 | 25.7553 |
| F7 | | 2201 A21 | pGEM | UNKNOWN | 28508.2578 | 25.3019 |
| F8 | | 2201 A21 | pGEM | UNKNOWN | 25455.752 | 25.4689 |
| G3 | | 2201 A11 | pGEM | UNKNOWN | 9814.6279 | 26.8737 |
| G4 | | 2201 A11 | pGEM | UNKNOWN | 7984.9932 | 27.1778 |
| G5 | | 2201 A11 | pGEM | UNKNOWN | 7508.8682 | 27.2684 |
| G6 | | 2201 A21 | pGEM | UNKNOWN | 21335.9277 | 25.7291 |
| G7 | | 2201 A21 | pGEM | UNKNOWN | 5356.3276 | 27.7663 |
| G8 | | 2201 A21 | pGEM | UNKNOWN | 5701.8037 | 27.6742 |
| G12 | | | pGEM | NTC | | Undetermined |
| H1 | | Comf2E4 | pGEM | UNKNOWN | 11168.7227 | 26.6832 |
| H2 | | Comf2E4 | pGEM | UNKNOWN | 12785.0859 | 26.4839 |
| H3 | | 2201 A11 | pGEM | UNKNOWN | 20502.3301 | 25.7878 |
| H4 | | 2201 A11 | pGEM | UNKNOWN | 19358.1484 | 25.8725 |
| H5 | | 2201 A11 | pGEM | UNKNOWN | 18067.4316 | 25.9742 |
| H6 | | 2201 A21 | pGEM | UNKNOWN | 18774.0957 | 25.9176 |
| H7 | | 2201 A21 | pGEM | UNKNOWN | 20061.9805 | 25.8198 |

| Well | | Sample | Target | Task | Quantity | C |
|---|---|---|---|---|---|---|
| H8 | | 2201 A21 | pGEM | UNKNOWN | 22570.4434 | 25.6462 |
| H12 | | | pGEM | NTC | | Undetermined |

# QC Summary

| | | | | | |
|---|---|---|---|---|---|
| Total Wells | 96 | Processed Wells | 64 | Targets Used | 1 |
| Well Setup | 64 | Flagged Wells | 6 | Samples Used | 17 |

| Flag | Name | Frequency | Locations |
|---|---|---|---|
| AMPNC | Amplification in negative control | 0 | |
| BADROX | Bad passive reference signal | 0 | |
| BLFAIL | Baseline algorithm failed | 0 | |
| CTFAIL | C algorithm failed | 0 | |
| EXPFAIL | Exponential algorithm failed | 0 | |
| HIGHSD | High standard deviation in replicate group | 6 | F3, F4, F5, G6, G7, G8 |
| NOAMP | No amplification | 0 | |
| NOISE | Noise higher than others in plate | 0 | |
| NOSIGNAL | No signal in well | 0 | |
| OFFSCALE | Fluorescence is offscale | 0 | |
| OUTLIERRG | Outlier in replicate group | 0 | |
| SPIKE | Noise spikes | 0 | |
| THOLDFAIL | Thresholding algorithm failed | 0 | |
