# Supplementary material for: Ecological impact of algal-preying protists aplanochytrids (Labyrinthulea) as a source of docosahexaenoic acid for copepods in marine environments
Source: ISME Commun. 2026 Jun 26;6(1):ycag178. doi: 10.1093/ismeco/ycag178 (PMC13685785; doi:10.1093/ismeco/ycag178)
Supplement: Supplementary file 2 [file supplementary_materials_ycag178.zip › Morimoto_ISME_C_3rdrev_supplemental_data.zip!/Morimoto_ISME_C_3rdrev_supplemental_data/Supplemental_Data_2/Standard Curve/DNA recovery/2201/DnaRec2201AlineA11-A21N=3.pdf]

Experiment Results Report

230628DnaRec2201AlineA11-A21N=3

Experiment Summary

Experiment Name	:	230628DnaRec2201AlineA11-A21N=3
Experiment Type	:	Quantitation - Standard Curve
File Name	:	230628DnaRec2201AlineA11-A21N=3.eds
Run Started	:	2023 Jun 28 2:27:08 PM
Run Finished	:	2023 Jun 28 4:01:42 PM
Run Duration	:	94 minutes 34 seconds
Date Modified	:	2023 Jun 28 4:29:43 PM
User	:	
Number of wells used	:	64
Number of wells with results	:	64
Instrument Name	:	
Instrument Type	:	Applied Biosystems StepOnePlus™ Instrument
Comments	:	

Results Summary

Sample	Target	Quantity (Mean)	Quantity (Std Dev)	C (Mean)	C (Std Dev)
2201 A011 200m	pGEM	26173.99	1519.33	25.35	0.09
2201 A011SCM	pGEM	13333.25	1084.56	26.36	0.12
2201 A11 1000m	pGEM	25898.42	1297.88	25.36	0.08
2201 A11 1000m	pGEM	14897.43	525.69	26.19	0.05
2201 A11 200m	pGEM	13389.24	281.86	26.35	0.03
2201 A11 3000m	pGEM	28080.81	674.73	25.24	0.04
2201 A11 3000m	pGEM	12354.24	153.50	26.47	0.02
2201 A11 SCM ato1	pGEM	27746.27	2233.64	25.26	0.12
2201 A21 1000m	pGEM	27337.20	954.29	25.28	0.05
2201 A21 1000m	pGEM	8143.25	607.25	27.10	0.11
2201 A21 200 ato1	pGEM	28026.32	3167.58	25.25	0.17
2201 A21 200 saki1	pGEM	11166.47	414.55	26.62	0.06
2201 A21 3000m	pGEM	27818.31	1081.59	25.26	0.06
2201 A21 3000m	pGEM	7100.71	883.12	27.31	0.18
2201 A21 SCM ato1	pGEM	27266.57	1078.03	25.29	0.06
2201 A21 SCM saki1	pGEM	9550.12	671.06	26.86	0.10
Comf2E4	pGEM	27620.01	1056.64	25.27	0.06

Plate Layout

	1	2	3	4	5	6	7	8	9	10	11	12
A	S pGEM<br>2E6<br>FAM-NFQ-MGB<br>CT: 19.43	S pGEM<br>2E6<br>FAM-NFQ-MGB<br>CT: 18.78	2201 A011SCM saki1<br>U pGEM<br>1.44E4<br>FAM-NFQ-MGB<br>CT: 26.24	2201 A011SCM saki1<br>U pGEM<br>1.22E4<br>FAM-NFQ-MGB<br>CT: 26.48	2201 A011SCM saki1<br>U pGEM<br>1.34E4<br>FAM-NFQ-MGB<br>CT: 26.35	2201 A21 SCM saki1<br>U pGEM<br>8.94E3<br>FAM-NFQ-MGB<br>CT: 26.95	2201 A21 SCM saki1<br>U pGEM<br>9.43E3<br>FAM-NFQ-MGB<br>CT: 26.87	2201 A21 SCM saki1<br>U pGEM<br>1.03E4<br>FAM-NFQ-MGB<br>CT: 26.75				
B	S pGEM<br>2E5<br>FAM-NFQ-MGB<br>CT: 22.03	S pGEM<br>2E5<br>FAM-NFQ-MGB<br>CT: 21.86	2201 A11 SCM ato1<br>U pGEM<br>2.65E4<br>FAM-NFQ-MGB<br>CT: 25.33	2201 A11 SCM ato1<br>U pGEM<br>3.03E4<br>FAM-NFQ-MGB<br>CT: 25.13	2201 A11 SCM ato1<br>U pGEM<br>2.64E4<br>FAM-NFQ-MGB<br>CT: 25.33	2201 A21 SCM ato1<br>U pGEM<br>2.61E4<br>FAM-NFQ-MGB<br>CT: 25.35	2201 A21 SCM ato1<br>U pGEM<br>2.76E4<br>FAM-NFQ-MGB<br>CT: 25.27	2201 A21 SCM ato1<br>U pGEM<br>2.81E4<br>FAM-NFQ-MGB<br>CT: 25.24				
C	S pGEM<br>2E4<br>FAM-NFQ-MGB<br>CT: 25.87	S pGEM<br>2E4<br>FAM-NFQ-MGB<br>CT: 25.39	2201 A11 200m saki1<br>U pGEM<br>1.31E4<br>FAM-NFQ-MGB<br>CT: 26.38	2201 A11 200m saki1<br>U pGEM<br>1.36E4<br>FAM-NFQ-MGB<br>CT: 26.32	2201 A11 200m saki1<br>U pGEM<br>1.34E4<br>FAM-NFQ-MGB<br>CT: 26.34	2201 A21 200 saki1<br>U pGEM<br>1.16E4<br>FAM-NFQ-MGB<br>CT: 26.56	2201 A21 200 saki1<br>U pGEM<br>1.1E4<br>FAM-NFQ-MGB<br>CT: 26.64	2201 A21 200 saki1<br>U pGEM<br>1.09E4<br>FAM-NFQ-MGB<br>CT: 26.66				
D	S pGEM<br>2E3<br>FAM-NFQ-MGB<br>CT: 29.14	S pGEM<br>2E3<br>FAM-NFQ-MGB<br>CT: 29.15	2201 A011 200m ato1<br>U pGEM<br>2.68E4<br>FAM-NFQ-MGB<br>CT: 25.31	2201 A011 200m ato1<br>U pGEM<br>2.73E4<br>FAM-NFQ-MGB<br>CT: 25.28	2201 A011 200m ato1<br>U pGEM<br>2.44E4<br>FAM-NFQ-MGB<br>CT: 25.45	2201 A21 200 ato1<br>U pGEM<br>2.51E4<br>FAM-NFQ-MGB<br>CT: 25.41	2201 A21 200 ato1<br>U pGEM<br>3.14E4<br>FAM-NFQ-MGB<br>CT: 25.08	2201 A21 200 ato1<br>U pGEM<br>2.77E4<br>FAM-NFQ-MGB<br>CT: 25.26				
E	S pGEM<br>200<br>FAM-NFQ-MGB<br>CT: 33.35	S pGEM<br>200<br>FAM-NFQ-MGB<br>CT: 33.2	2201 A11 1000m saki1<br>U pGEM<br>1.54E4<br>FAM-NFQ-MGB<br>CT: 26.14	2201 A11 1000m saki1<br>U pGEM<br>1.49E4<br>FAM-NFQ-MGB<br>CT: 26.19	2201 A11 1000m saki1<br>U pGEM<br>1.44E4<br>FAM-NFQ-MGB<br>CT: 26.24	2201 A21 1000m saki1<br>U pGEM<br>7.52E3<br>FAM-NFQ-MGB<br>CT: 27.21	2201 A21 1000m saki1<br>U pGEM<br>8.73E3<br>FAM-NFQ-MGB<br>CT: 26.99	2201 A21 1000m saki1<br>U pGEM<br>8.18E3<br>FAM-NFQ-MGB<br>CT: 27.09				
F	S pGEM<br>20<br>FAM-NFQ-MGB<br>CT: 35.98	S pGEM<br>20<br>FAM-NFQ-MGB<br>CT: 35.48	2201 A11 1000m ato1<br>U pGEM<br>2.71E4<br>FAM-NFQ-MGB<br>CT: 25.29	2201 A11 1000m ato1<br>U pGEM<br>2.45E4<br>FAM-NFQ-MGB<br>CT: 25.44	2201 A11 1000m ato1<br>U pGEM<br>2.6E4<br>FAM-NFQ-MGB<br>CT: 25.35	2201 A21 1000m ato1<br>U pGEM<br>2.63E4<br>FAM-NFQ-MGB<br>CT: 25.34	2201 A21 1000m ato1<br>U pGEM<br>2.77E4<br>FAM-NFQ-MGB<br>CT: 25.26	2201 A21 1000m ato1<br>U pGEM<br>2.81E4<br>FAM-NFQ-MGB<br>CT: 25.24				
G			2201 A11 3000m saki1<br>U pGEM<br>1.25E4<br>FAM-NFQ-MGB<br>CT: 26.45	2201 A11 3000m saki1<br>U pGEM<br>1.22E4<br>FAM-NFQ-MGB<br>CT: 26.48	2201 A11 3000m saki1<br>U pGEM<br>1.23E4<br>FAM-NFQ-MGB<br>CT: 26.48	2201 A21 3000m saki1<br>U pGEM<br>6.35E3<br>FAM-NFQ-MGB<br>CT: 27.46	2201 A21 3000m saki1<br>U pGEM<br>6.88E3<br>FAM-NFQ-MGB<br>CT: 27.35	2201 A21 3000m saki1<br>U pGEM<br>8.07E3<br>FAM-NFQ-MGB<br>CT: 27.11				N pGEM<br>FAM-NFQ-MGB<br>CT: Undetermined
H	Comf2E4<br>U pGEM<br>2.69E4<br>FAM-NFQ-MGB<br>CT: 25.31	Comf2E4<br>U pGEM<br>2.84E4<br>FAM-NFQ-MGB<br>CT: 25.23	2201 A11 3000m ato1<br>U pGEM<br>2.89E4<br>FAM-NFQ-MGB<br>CT: 25.2	2201 A11 3000m ato1<br>U pGEM<br>2.78E4<br>FAM-NFQ-MGB<br>CT: 25.26	2201 A11 3000m ato1<br>U pGEM<br>2.76E4<br>FAM-NFQ-MGB<br>CT: 25.27	2201 A21 3000m ato1<br>U pGEM<br>2.66E4<br>FAM-NFQ-MGB<br>CT: 25.32	2201 A21 3000m ato1<br>U pGEM<br>2.87E4<br>FAM-NFQ-MGB<br>CT: 25.21	2201 A21 3000m ato1<br>U pGEM<br>2.81E4<br>FAM-NFQ-MGB<br>CT: 25.24				N pGEM<br>FAM-NFQ-MGB<br>CT: Undetermined

Standard Curves

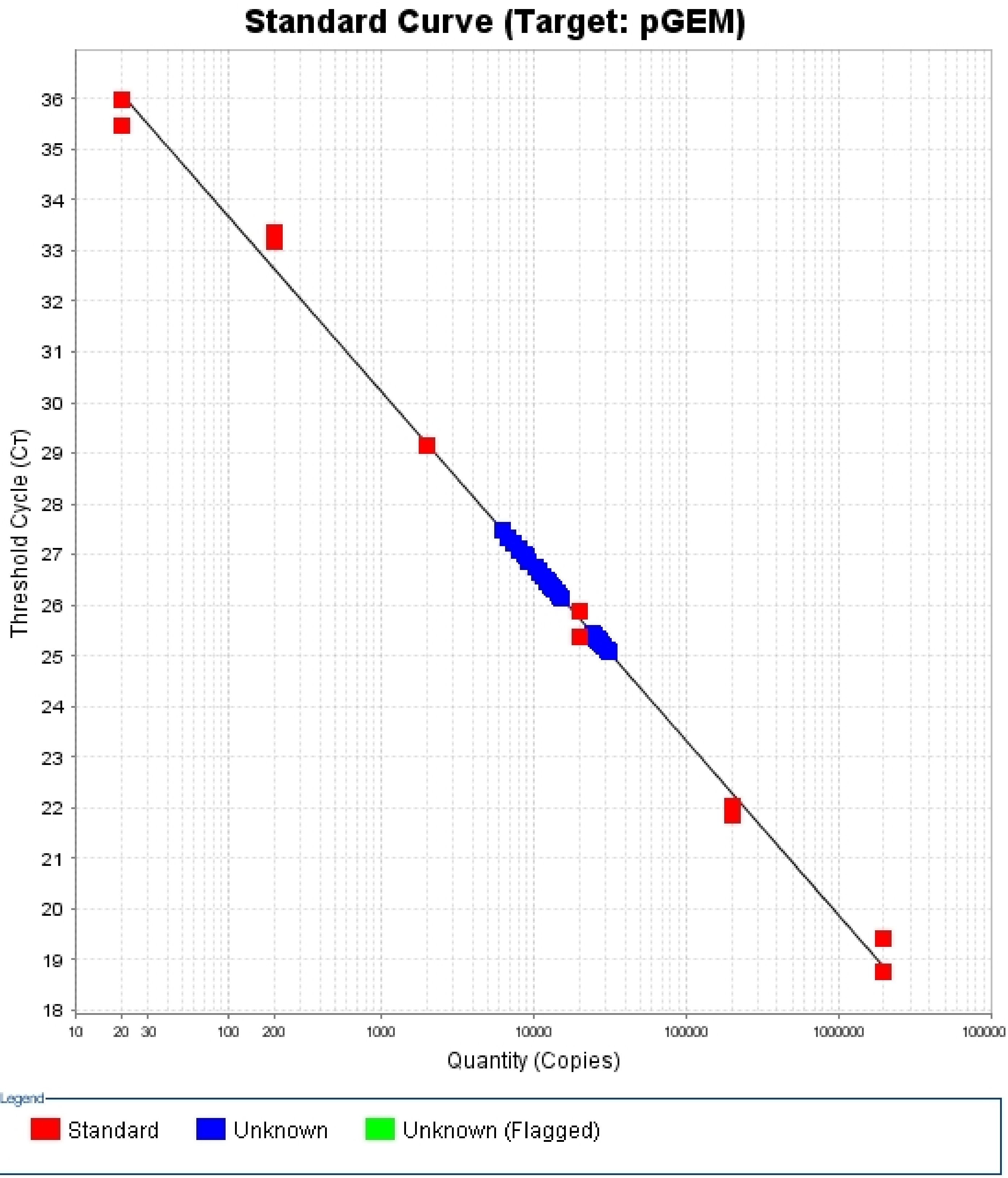

slope:-3.446 Y-Intercept:40.569 R^2:0.995 Eff%:95.074

Results Table

Well		Sample	Target	Task	Quantity	C
A1			pGEM	STANDARD	2000000	19.43
A2			pGEM	STANDARD	2000000	18.7806
A3		2201 A011SCM	pGEM	UNKNOWN	14398.7812	26.2399
A4		2201 A011SCM	pGEM	UNKNOWN	12230.6191	26.4842
A5		2201 A011SCM	pGEM	UNKNOWN	13370.335	26.3508
A6		2201 A21 SCM	pGEM	UNKNOWN	8944.3652	26.9525
A7		2201 A21 SCM	pGEM	UNKNOWN	9434.5264	26.8726
A8		2201 A21 SCM	pGEM	UNKNOWN	10271.4736	26.7454
B1			pGEM	STANDARD	200000	22.033
B2			pGEM	STANDARD	200000	21.8625
B3		2201 A11 SCM	pGEM	UNKNOWN	26515.8984	25.3261
B4		2201 A11 SCM	pGEM	UNKNOWN	30324.5625	25.1253
B5		2201 A11 SCM	pGEM	UNKNOWN	26398.3516	25.3328
B6		2201 A21 SCM	pGEM	UNKNOWN	26060.5488	25.3521
B7		2201 A21 SCM	pGEM	UNKNOWN	27602.584	25.266
B8		2201 A21 SCM	pGEM	UNKNOWN	28136.5762	25.2374
C1			pGEM	STANDARD	20000	25.865
C2			pGEM	STANDARD	20000	25.39
C3		2201 A11 200m	pGEM	UNKNOWN	13085.4951	26.3831
C4		2201 A11 200m	pGEM	UNKNOWN	13642.3408	26.3207
C5		2201 A11 200m	pGEM	UNKNOWN	13439.8877	26.3431
C6		2201 A21 200	pGEM	UNKNOWN	11637.5322	26.5585
C7		2201 A21 200	pGEM	UNKNOWN	11004.6045	26.6422
C8		2201 A21 200	pGEM	UNKNOWN	10857.2666	26.6624
D1			pGEM	STANDARD	2000	29.1415
D2			pGEM	STANDARD	2000	29.1487
D3		2201 A011	pGEM	UNKNOWN	26764.6738	25.3122
D4		2201 A011	pGEM	UNKNOWN	27309.2773	25.282
D5		2201 A011	pGEM	UNKNOWN	24448.0234	25.4477
D6		2201 A21 200	pGEM	UNKNOWN	25054.4258	25.411

Well		Sample	Target	Task	Quantity	C
D7		2201 A21 200	pGEM	UNKNOWN	31358.7363	25.0751
D8		2201 A21 200	pGEM	UNKNOWN	27665.8047	25.2626
E1			pGEM	STANDARD	200	33.3476
E2			pGEM	STANDARD	200	33.1958
E3		2201 A11	pGEM	UNKNOWN	15420.1914	26.1374
E4		2201 A11	pGEM	UNKNOWN	14903.2344	26.1884
E5		2201 A11	pGEM	UNKNOWN	14368.8623	26.243
E6		2201 A21	pGEM	UNKNOWN	7517.1016	27.2126
E7		2201 A21	pGEM	UNKNOWN	8729.6494	26.9888
E8		2201 A21	pGEM	UNKNOWN	8183.0132	27.0856
F1			pGEM	STANDARD	20	35.9767
F2			pGEM	STANDARD	20	35.4811
F3		2201 A11	pGEM	UNKNOWN	27113.8828	25.2928
F4		2201 A11	pGEM	UNKNOWN	24531.4219	25.4426
F5		2201 A11	pGEM	UNKNOWN	26049.957	25.3527
F6		2201 A21	pGEM	UNKNOWN	26264.584	25.3404
F7		2201 A21	pGEM	UNKNOWN	27654.9102	25.2632
F8		2201 A21	pGEM	UNKNOWN	28092.1094	25.2397
G3		2201 A11	pGEM	UNKNOWN	12529.9922	26.448
G4		2201 A11	pGEM	UNKNOWN	12246.4668	26.4822
G5		2201 A11	pGEM	UNKNOWN	12286.2549	26.4774
G6		2201 A21	pGEM	UNKNOWN	6350.6709	27.465
G7		2201 A21	pGEM	UNKNOWN	6877.397	27.3457
G8		2201 A21	pGEM	UNKNOWN	8074.0493	27.1057
G12			pGEM	NTC		Undetermined
H1		Comf2E4	pGEM	UNKNOWN	26872.8555	25.3061
H2		Comf2E4	pGEM	UNKNOWN	28367.166	25.2251
H3		2201 A11	pGEM	UNKNOWN	28851.8223	25.1998
H4		2201 A11	pGEM	UNKNOWN	27792.3965	25.2558
H5		2201 A11	pGEM	UNKNOWN	27598.2227	25.2663
H6		2201 A21	pGEM	UNKNOWN	26627.8594	25.3198
H7		2201 A21	pGEM	UNKNOWN	28740.5801	25.2056

Well		Sample	Target	Task	Quantity	C
H8		2201 A21	pGEM	UNKNOWN	28086.4883	25.24
H12			pGEM	NTC		Undetermined

QC Summary

Total Wells	96	Processed Wells	64	Targets Used	1
Well Setup	64	Flagged Wells	0	Samples Used	17

Flag	Name	Frequency	Locations
AMPNC	Amplification in negative control	0	
BADROX	Bad passive reference signal	0	
BLFAIL	Baseline algorithm failed	0	
CTFAIL	C algorithm failed	0	
EXPFAIL	Exponential algorithm failed	0	
HIGHSD	High standard deviation in replicate group	0	
NOAMP	No amplification	0	
NOISE	Noise higher than others in plate	0	
NOSIGNAL	No signal in well	0	
OFFSCALE	Fluorescence is offscale	0	
OUTLIERRG	Outlier in replicate group	0	
SPIKE	Noise spikes	0	
THOLDFAIL	Thresholding algorithm failed	0	
