# Supplementary material for: Ecological impact of algal-preying protists aplanochytrids (Labyrinthulea) as a source of docosahexaenoic acid for copepods in marine environments
Source: ISME Commun. 2026 Jun 26;6(1):ycag178. doi: 10.1093/ismeco/ycag178 (PMC13685785; doi:10.1093/ismeco/ycag178)
Supplement: Supplementary file 2 [file supplementary_materials_ycag178.zip › Morimoto_ISME_C_3rdrev_supplemental_data.zip!/Morimoto_ISME_C_3rdrev_supplemental_data/Supplemental_Data_2/Standard Curve/DNA recovery/2201/DnaRec2201AlineA11-A21N=2.pdf]

Experiment Results Report

230627DnaRec2201AlineA11-A21N=2

Experiment Summary

Experiment Name	:	230627DnaRec2201AlineA11-A21N=2
Experiment Type	:	Quantitation - Standard Curve
File Name	:	230627DnaRec2201AlineA11-A21N=2.eds
Run Started	:	2023 Jun 27 5:49:45 PM
Run Finished	:	2023 Jun 27 7:24:22 PM
Run Duration	:	94 minutes 36 seconds
Date Modified	:	2023 Jun 28 10:51:20 AM
User	:	
Number of wells used	:	64
Number of wells with results	:	64
Instrument Name	:	
Instrument Type	:	Applied Biosystems StepOnePlus™ Instrument
Comments	:	

Results Summary

Sample	Target	Quantity (Mean)	Quantity (Std Dev)	C (Mean)	C (Std Dev)
2201 A011 200m	pGEM	18262.96	853.59	25.42	0.07
2201 A011SCM	pGEM	10622.36	249.40	26.25	0.04
2201 A11 1000m	pGEM	17323.76	1718.17	25.51	0.15
2201 A11 1000m	pGEM	9805.00	652.21	26.37	0.10
2201 A11 200m	pGEM	9690.61	314.11	26.39	0.05
2201 A11 3000m	pGEM	17613.36	289.85	25.48	0.03
2201 A11 3000m	pGEM	4110.68	71.71	27.69	0.03
2201 A11 SCM ato2	pGEM	19939.78	42.09	25.29	0.00
2201 A21 1000m	pGEM	17739.25	199.01	25.47	0.02
2201 A21 1000m	pGEM	5639.72	354.65	27.21	0.09
2201 A21 200 ato2	pGEM	18089.86	944.90	25.44	0.08
2201 A21 200 saki2	pGEM	7405.22	113.77	26.79	0.02
2201 A21 3000m	pGEM	17883.67	1051.32	25.46	0.09
2201 A21 3000m	pGEM	5124.16	252.75	27.35	0.08
2201 A21 SCM ato2	pGEM	19818.91	398.55	25.30	0.03
2201 A21 SCM saki2	pGEM	7730.69	153.45	26.73	0.03
Comf2E4	pGEM	17769.39	1405.86	25.47	0.12

Plate Layout

	1	2	3	4	5	6	7	8	9	10	11	12
A	S pGEM<br>2E6<br>FAM-NFQ-MGB<br>CT: 18.55	S pGEM<br>2E6<br>FAM-NFQ-MGB<br>CT: 18.33	2201 A011SCM saki2<br>U pGEM<br>1.09E4<br>FAM-NFQ-MGB<br>CT: 26.2	2201 A011SCM saki2<br>U pGEM<br>1.05E4<br>FAM-NFQ-MGB<br>CT: 26.27	2201 A011SCM saki2<br>U pGEM<br>1.05E4<br>FAM-NFQ-MGB<br>CT: 26.26	2201 A21 SCM saki2<br>U pGEM<br>7.56E3<br>FAM-NFQ-MGB<br>CT: 26.76	2201 A21 SCM saki2<br>U pGEM<br>7.8E3<br>FAM-NFQ-MGB<br>CT: 26.71	2201 A21 SCM saki2<br>U pGEM<br>7.84E3<br>FAM-NFQ-MGB<br>CT: 26.71				
B	S pGEM<br>2E5<br>FAM-NFQ-MGB<br>CT: 21.74	S pGEM<br>2E5<br>FAM-NFQ-MGB<br>CT: 21.57	2201 A11 SCM ato2<br>U pGEM<br>1.99E4<br>FAM-NFQ-MGB<br>CT: 25.29	2201 A11 SCM ato2<br>U pGEM<br>2E4<br>FAM-NFQ-MGB<br>CT: 25.29	2201 A11 SCM ato2<br>U pGEM<br>1.99E4<br>FAM-NFQ-MGB<br>CT: 25.29	2201 A21 SCM ato2<br>U pGEM<br>1.99E4<br>FAM-NFQ-MGB<br>CT: 25.29	2201 A21 SCM ato2<br>U pGEM<br>1.94E4<br>FAM-NFQ-MGB<br>CT: 25.33	2201 A21 SCM ato2<br>U pGEM<br>2.02E4<br>FAM-NFQ-MGB<br>CT: 25.27				
C	S pGEM<br>2E4<br>FAM-NFQ-MGB<br>CT: 25.36	S pGEM<br>2E4<br>FAM-NFQ-MGB<br>CT: 25.21	2201 A11 200m saki2<br>U pGEM<br>9.34E3<br>FAM-NFQ-MGB<br>CT: 26.44	2201 A11 200m saki2<br>U pGEM<br>9.93E3<br>FAM-NFQ-MGB<br>CT: 26.35	2201 A11 200m saki2<br>U pGEM<br>9.8E3<br>FAM-NFQ-MGB<br>CT: 26.37	2201 A21 200 saki2<br>U pGEM<br>7.27E3<br>FAM-NFQ-MGB<br>CT: 26.82	2201 A21 200 saki2<br>U pGEM<br>7.46E3<br>FAM-NFQ-MGB<br>CT: 26.78	2201 A21 200 saki2<br>U pGEM<br>7.48E3<br>FAM-NFQ-MGB<br>CT: 26.78				
D	S pGEM<br>2E3<br>FAM-NFQ-MGB<br>CT: 28.69	S pGEM<br>2E3<br>FAM-NFQ-MGB<br>CT: 28.78	2201 A011 200m ato2<br>U pGEM<br>1.81E4<br>FAM-NFQ-MGB<br>CT: 25.43	2201 A011 200m ato2<br>U pGEM<br>1.75E4<br>FAM-NFQ-MGB<br>CT: 25.49	2201 A011 200m ato2<br>U pGEM<br>1.92E4<br>FAM-NFQ-MGB<br>CT: 25.35	2201 A21 200 ato2<br>U pGEM<br>1.73E4<br>FAM-NFQ-MGB<br>CT: 25.51	2201 A21 200 ato2<br>U pGEM<br>1.91E4<br>FAM-NFQ-MGB<br>CT: 25.35	2201 A21 200 ato2<br>U pGEM<br>1.79E4<br>FAM-NFQ-MGB<br>CT: 25.46				
E	S pGEM<br>200<br>FAM-NFQ-MGB<br>CT: 32.03	S pGEM<br>200<br>FAM-NFQ-MGB<br>CT: 32.23	2201 A11 1000m saki2<br>U pGEM<br>1.02E4<br>FAM-NFQ-MGB<br>CT: 26.31	2201 A11 1000m saki2<br>U pGEM<br>1.02E4<br>FAM-NFQ-MGB<br>CT: 26.31	2201 A11 1000m saki2<br>U pGEM<br>9.05E3<br>FAM-NFQ-MGB<br>CT: 26.49	2201 A21 1000m saki2<br>U pGEM<br>5.42E3<br>FAM-NFQ-MGB<br>CT: 27.27	2201 A21 1000m saki2<br>U pGEM<br>6.05E3<br>FAM-NFQ-MGB<br>CT: 27.1	2201 A21 1000m saki2<br>U pGEM<br>5.45E3<br>FAM-NFQ-MGB<br>CT: 27.26				
F	S pGEM<br>20<br>FAM-NFQ-MGB<br>CT: 35.98	S pGEM<br>20<br>FAM-NFQ-MGB<br>CT: 35.93	2201 A11 1000m ato2<br>U pGEM<br>1.78E4<br>FAM-NFQ-MGB<br>CT: 25.46	2201 A11 1000m ato2<br>U pGEM<br>1.54E4<br>FAM-NFQ-MGB<br>CT: 25.68	2201 A11 1000m ato2<br>U pGEM<br>1.88E4<br>FAM-NFQ-MGB<br>CT: 25.38	2201 A21 1000m ato2<br>U pGEM<br>1.76E4<br>FAM-NFQ-MGB<br>CT: 25.48	2201 A21 1000m ato2<br>U pGEM<br>1.77E4<br>FAM-NFQ-MGB<br>CT: 25.47	2201 A21 1000m ato2<br>U pGEM<br>1.8E4<br>FAM-NFQ-MGB<br>CT: 25.45				
G			2201 A11 3000m saki2<br>U pGEM<br>4.16E3<br>FAM-NFQ-MGB<br>CT: 27.67	2201 A11 3000m saki2<br>U pGEM<br>4.14E3<br>FAM-NFQ-MGB<br>CT: 27.68	2201 A11 3000m saki2<br>U pGEM<br>4.03E3<br>FAM-NFQ-MGB<br>CT: 27.72	2201 A21 3000m saki2<br>U pGEM<br>5.24E3<br>FAM-NFQ-MGB<br>CT: 27.32	2201 A21 3000m saki2<br>U pGEM<br>4.83E3<br>FAM-NFQ-MGB<br>CT: 27.44	2201 A21 3000m saki2<br>U pGEM<br>5.3E3<br>FAM-NFQ-MGB<br>CT: 27.3				N pGEM<br>FAM-NFQ-MGB<br>CT: Undetermined
H	Comf2E4<br>U pGEM<br>1.68E4<br>FAM-NFQ-MGB<br>CT: 25.55	Comf2E4<br>U pGEM<br>1.88E4<br>FAM-NFQ-MGB<br>CT: 25.38	2201 A11 3000m ato2<br>U pGEM<br>1.73E4<br>FAM-NFQ-MGB<br>CT: 25.5	2201 A11 3000m ato2<br>U pGEM<br>1.79E4<br>FAM-NFQ-MGB<br>CT: 25.46	2201 A11 3000m ato2<br>U pGEM<br>1.77E4<br>FAM-NFQ-MGB<br>CT: 25.47	2201 A21 3000m ato2<br>U pGEM<br>1.87E4<br>FAM-NFQ-MGB<br>CT: 25.38	2201 A21 3000m ato2<br>U pGEM<br>1.67E4<br>FAM-NFQ-MGB<br>CT: 25.56	2201 A21 3000m ato2<br>U pGEM<br>1.82E4<br>FAM-NFQ-MGB<br>CT: 25.43				N pGEM<br>FAM-NFQ-MGB<br>CT: Undetermined

Standard Curves

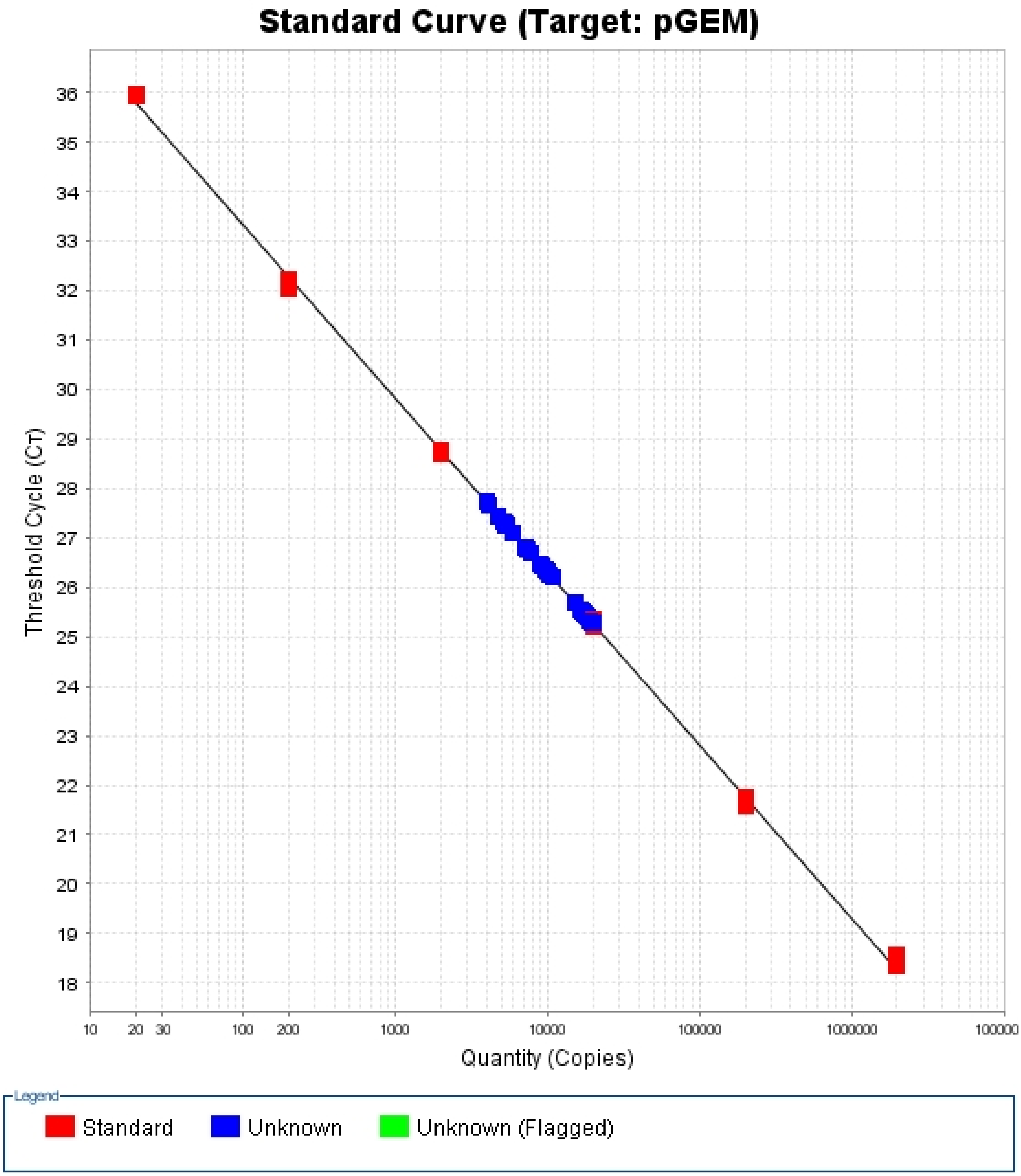

slope:-3.498 Y-Intercept:40.328 R^2:0.999 Eff%:93.146

Results Table

Well		Sample	Target	Task	Quantity	C
A1			pGEM	STANDARD	2000000	18.5504
A2			pGEM	STANDARD	2000000	18.3339
A3		2201 A011SCM	pGEM	UNKNOWN	10908.6592	26.2047
A4		2201 A011SCM	pGEM	UNKNOWN	10452.2666	26.2696
A5		2201 A011SCM	pGEM	UNKNOWN	10506.1455	26.2618
A6		2201 A21 SCM	pGEM	UNKNOWN	7555.1787	26.7627
A7		2201 A21 SCM	pGEM	UNKNOWN	7797.3921	26.7148
A8		2201 A21 SCM	pGEM	UNKNOWN	7839.5049	26.7066
B1			pGEM	STANDARD	200000	21.742
B2			pGEM	STANDARD	200000	21.5702
B3		2201 A11 SCM	pGEM	UNKNOWN	19899.8867	25.2915
B4		2201 A11 SCM	pGEM	UNKNOWN	19983.7637	25.2851
B5		2201 A11 SCM	pGEM	UNKNOWN	19935.6973	25.2887
B6		2201 A21 SCM	pGEM	UNKNOWN	19919.3105	25.29
B7		2201 A21 SCM	pGEM	UNKNOWN	19379.7598	25.3317
B8		2201 A21 SCM	pGEM	UNKNOWN	20157.668	25.2719
C1			pGEM	STANDARD	20000	25.3624
C2			pGEM	STANDARD	20000	25.2062
C3		2201 A11 200m	pGEM	UNKNOWN	9336.1104	26.4412
C4		2201 A11 200m	pGEM	UNKNOWN	9934.3018	26.3468
C5		2201 A11 200m	pGEM	UNKNOWN	9801.4121	26.3673
C6		2201 A21 200	pGEM	UNKNOWN	7274.5767	26.8202
C7		2201 A21 200	pGEM	UNKNOWN	7458.6172	26.7822
C8		2201 A21 200	pGEM	UNKNOWN	7482.479	26.7774
D1			pGEM	STANDARD	2000	28.687
D2			pGEM	STANDARD	2000	28.7758
D3		2201 A011	pGEM	UNKNOWN	18136.6641	25.4324
D4		2201 A011	pGEM	UNKNOWN	17479.5566	25.4885
D5		2201 A011	pGEM	UNKNOWN	19172.6602	25.348
D6		2201 A21 200	pGEM	UNKNOWN	17275.3809	25.5063

Well	Sample	Target	Task	Quantity	C
D7	2201 A21 200	pGEM	UNKNOWN	19125.8242	25.3517
D8	2201 A21 200	pGEM	UNKNOWN	17868.3672	25.455
E1		pGEM	STANDARD	200	32.0279
E2		pGEM	STANDARD	200	32.2266
E3	2201 A11	pGEM	UNKNOWN	10181.1182	26.3095
E4	2201 A11	pGEM	UNKNOWN	10182	26.3094
E5	2201 A11	pGEM	UNKNOWN	9051.8945	26.4881
E6	2201 A21	pGEM	UNKNOWN	5417.9302	27.2678
E7	2201 A21	pGEM	UNKNOWN	6048.751	27.1005
E8	2201 A21	pGEM	UNKNOWN	5452.481	27.2582
F1		pGEM	STANDARD	20	35.9784
F2		pGEM	STANDARD	20	35.9325
F3	2201 A11	pGEM	UNKNOWN	17772.8047	25.4632
F4	2201 A11	pGEM	UNKNOWN	15425.6533	25.6783
F5	2201 A11	pGEM	UNKNOWN	18772.8086	25.38
F6	2201 A21	pGEM	UNKNOWN	17557.75	25.4817
F7	2201 A21	pGEM	UNKNOWN	17707.9414	25.4687
F8	2201 A21	pGEM	UNKNOWN	17952.0645	25.4479
G3	2201 A11	pGEM	UNKNOWN	4161.0947	27.6688
G4	2201 A11	pGEM	UNKNOWN	4142.3604	27.6756
G5	2201 A11	pGEM	UNKNOWN	4028.5938	27.7179
G6	2201 A21	pGEM	UNKNOWN	5237.1357	27.3194
G7	2201 A21	pGEM	UNKNOWN	4834.6279	27.4409
G8	2201 A21	pGEM	UNKNOWN	5300.7163	27.3011
G12		pGEM	NTC		Undetermined
H1	Comf2E4	pGEM	UNKNOWN	16775.2949	25.5509
H2	Comf2E4	pGEM	UNKNOWN	18763.4766	25.3808
H3	2201 A11	pGEM	UNKNOWN	17291.0059	25.5049
H4	2201 A11	pGEM	UNKNOWN	17852.4902	25.4564
H5	2201 A11	pGEM	UNKNOWN	17696.584	25.4697
H6	2201 A21	pGEM	UNKNOWN	18737.6504	25.3829
H7	2201 A21	pGEM	UNKNOWN	16709.4785	25.5569

Well		Sample	Target	Task	Quantity	C
H8		2201 A21	pGEM	UNKNOWN	18203.873	25.4268
H12			pGEM	NTC		Undetermined

QC Summary

Total Wells	96	Processed Wells	64	Targets Used	1
Well Setup	64	Flagged Wells	0	Samples Used	17

Flag	Name	Frequency	Locations
AMPNC	Amplification in negative control	0	
BADROX	Bad passive reference signal	0	
BLFAIL	Baseline algorithm failed	0	
CTFAIL	C algorithm failed	0	
EXPFAIL	Exponential algorithm failed	0	
HIGHSD	High standard deviation in replicate group	0	
NOAMP	No amplification	0	
NOISE	Noise higher than others in plate	0	
NOSIGNAL	No signal in well	0	
OFFSCALE	Fluorescence is offscale	0	
OUTLIERRG	Outlier in replicate group	0	
SPIKE	Noise spikes	0	
THOLDFAIL	Thresholding algorithm failed	0	
