# Supplementary material for: Ecological impact of algal-preying protists aplanochytrids (Labyrinthulea) as a source of docosahexaenoic acid for copepods in marine environments
Source: ISME Commun. 2026 Jun 26;6(1):ycag178. doi: 10.1093/ismeco/ycag178 (PMC13685785; doi:10.1093/ismeco/ycag178)
Supplement: Supplementary file 2 [file supplementary_materials_ycag178.zip › Morimoto_ISME_C_3rdrev_supplemental_data.zip!/Morimoto_ISME_C_3rdrev_supplemental_data/Supplemental_Data_2/Standard Curve/DNA recovery/2201/DnaRec2201AlineA01-A07N=3.pdf]

Experiment Results Report
230628DnaRec2201AlineA01-A07N=3

Experiment Summary

Experiment Name	:	230628DnaRec2201AlineA01-A07N=3
Experiment Type	:	Quantitation - Standard Curve
File Name	:	230628DnaRec2201AlineA01-A07N=3.eds
Run Started	:	2023 Jun 28 11:54:32 AM
Run Finished	:	2023 Jun 28 1:29:15 PM
Run Duration	:	94 minutes 43 seconds
Date Modified	:	2023 Jun 28 1:39:37 PM
User	:	
Number of wells used	:	82
Number of wells with results	:	82
Instrument Name	:	
Instrument Type	:	Applied Biosystems StepOnePlus™ Instrument
Comments	:	

Results Summary

Sample	Target	Quantity (Mean)	Quantity (Std Dev)	C (Mean)	C (Std Dev)
2201 A01 10m ato1	pGEM	20012.66	1114.32	25.58	0.09
2201 A01 10m saki1	pGEM	9813.84	73.20	26.67	0.01
2201 A01 SCM ato1	pGEM	17542.56	196.14	25.78	0.02
2201 A01 SCM saki1	pGEM	10734.59	539.28	26.53	0.08
2201 A04 1000m	pGEM	18965.93	949.19	25.66	0.08
2201 A04 1000m	pGEM	7806.74	578.26	27.03	0.12
2201 A04 10m ato1	pGEM	17923.21	1042.94	25.75	0.09
2201 A04 10m saki1	pGEM	9169.98	518.44	26.78	0.09
2201 A04 200m ato1	pGEM	20331.42	1042.62	25.55	0.08
2201 A04 200m	pGEM	8186.02	276.61	26.95	0.05
2201 A04 3000m	pGEM	18606.60	1117.76	25.69	0.09
2201 A04 3000m	pGEM	5196.63	290.53	27.65	0.09
2201 A04 SCM ato1	pGEM	17920.38	532.55	25.75	0.05
2201 A04 SCM saki1	pGEM	8466.23	42.29	26.90	0.01
2201 A07 1000m	pGEM	19263.83	623.98	25.63	0.05
2201 A07 1000m	pGEM	11268.35	1198.41	26.47	0.17
2201 A07 200 ato1	pGEM	17736.78	795.15	25.76	0.07
2201 A07 200 saki1	pGEM	10875.80	603.81	26.52	0.09
2201 A07 3000m	pGEM	19058.03	858.73	25.65	0.07
2201 A07 3000m	pGEM	10852.67	249.84	26.52	0.04
2201 A07 SCM ato1	pGEM	18238.93	508.52	25.72	0.04
2201 A07 SCM saki1	pGEM	10227.48	778.47	26.61	0.12
Comf2E4	pGEM	17881.64	2105.90	25.75	0.18

Plate Layout

	1	2	3	4	5	6	7	8	9	10	11	12
A	S pGEM<br>2E6<br>FAM-NFQ-MGB<br>CT: 18.82	S pGEM<br>2E6<br>FAM-NFQ-MGB<br>CT: 18.63	2201 A01 10m saki1<br>U pGEM<br>9.8E3<br>FAM-NFQ-MGB<br>CT: 26.67	2201 A01 10m saki1<br>U pGEM<br>9.89E3<br>FAM-NFQ-MGB<br>CT: 26.66	2201 A01 10m saki1<br>U pGEM<br>9.75E3<br>FAM-NFQ-MGB<br>CT: 26.68	2201 A04 200m saki1<br>U pGEM<br>7.88E3<br>FAM-NFQ-MGB<br>CT: 27.01	2201 A04 200m saki1<br>U pGEM<br>8.25E3<br>FAM-NFQ-MGB<br>CT: 26.94	2201 A04 200m saki1<br>U pGEM<br>8.42E3<br>FAM-NFQ-MGB<br>CT: 26.91	2201 A07 200 saki1<br>U pGEM<br>1.02E4<br>FAM-NFQ-MGB<br>CT: 26.62	2201 A07 200 saki1<br>U pGEM<br>1.12E4<br>FAM-NFQ-MGB<br>CT: 26.47	2201 A07 200 saki1<br>U pGEM<br>1.13E4<br>FAM-NFQ-MGB<br>CT: 26.46	
B	S pGEM<br>2E5<br>FAM-NFQ-MGB<br>CT: 21.95	S pGEM<br>2E5<br>FAM-NFQ-MGB<br>CT: 21.89	2201 A01 10m ato1<br>U pGEM<br>2.12E4<br>FAM-NFQ-MGB<br>CT: 25.49	2201 A01 10m ato1<br>U pGEM<br>1.9E4<br>FAM-NFQ-MGB<br>CT: 25.65	2201 A01 10m ato1<br>U pGEM<br>1.98E4<br>FAM-NFQ-MGB<br>CT: 25.59	2201 A04 200m ato1<br>U pGEM<br>2.06E4<br>FAM-NFQ-MGB<br>CT: 25.53	2201 A04 200m ato1<br>U pGEM<br>1.92E4<br>FAM-NFQ-MGB<br>CT: 25.64	2201 A04 200m ato1<br>U pGEM<br>2.12E4<br>FAM-NFQ-MGB<br>CT: 25.48	2201 A07 200 ato1<br>U pGEM<br>1.7E4<br>FAM-NFQ-MGB<br>CT: 25.82	2201 A07 200 ato1<br>U pGEM<br>1.76E4<br>FAM-NFQ-MGB<br>CT: 25.77	2201 A07 200 ato1<br>U pGEM<br>1.86E4<br>FAM-NFQ-MGB<br>CT: 25.69	
C	S pGEM<br>2E4<br>FAM-NFQ-MGB<br>CT: 25.26	S pGEM<br>2E4<br>FAM-NFQ-MGB<br>CT: 25.46	2201 A01 SCM saki1<br>U pGEM<br>1.04E4<br>FAM-NFQ-MGB<br>CT: 26.58	2201 A01 SCM saki1<br>U pGEM<br>1.14E4<br>FAM-NFQ-MGB<br>CT: 26.45	2201 A01 SCM saki1<br>U pGEM<br>1.05E4<br>FAM-NFQ-MGB<br>CT: 26.57	2201 A04 1000m saki1<br>U pGEM<br>7.14E3<br>FAM-NFQ-MGB<br>CT: 27.16	2201 A04 1000m saki1<br>U pGEM<br>8.19E3<br>FAM-NFQ-MGB<br>CT: 26.95	2201 A04 1000m saki1<br>U pGEM<br>8.09E3<br>FAM-NFQ-MGB<br>CT: 26.97	2201 A07 1000m saki1<br>U pGEM<br>9.91E3<br>FAM-NFQ-MGB<br>CT: 26.66	2201 A07 1000m saki1<br>U pGEM<br>1.22E4<br>FAM-NFQ-MGB<br>CT: 26.34	2201 A07 1000m saki1<br>U pGEM<br>1.17E4<br>FAM-NFQ-MGB<br>CT: 26.4	
D	S pGEM<br>2E3<br>FAM-NFQ-MGB<br>CT: 29.05	S pGEM<br>2E3<br>FAM-NFQ-MGB<br>CT: 29.16	2201 A01 SCM ato1<br>U pGEM<br>1.78E4<br>FAM-NFQ-MGB<br>CT: 25.76	2201 A01 SCM ato1<br>U pGEM<br>1.74E4<br>FAM-NFQ-MGB<br>CT: 25.79	2201 A01 SCM ato1<br>U pGEM<br>1.75E4<br>FAM-NFQ-MGB<br>CT: 25.78	2201 A04 1000m ato1<br>U pGEM<br>1.81E4<br>FAM-NFQ-MGB<br>CT: 25.73	2201 A04 1000m ato1<br>U pGEM<br>2E4<br>FAM-NFQ-MGB<br>CT: 25.58	2201 A04 1000m ato1<br>U pGEM<br>1.89E4<br>FAM-NFQ-MGB<br>CT: 25.67	2201 A07 1000m ato1<br>U pGEM<br>1.91E4<br>FAM-NFQ-MGB<br>CT: 25.65	2201 A07 1000m ato1<br>U pGEM<br>2E4<br>FAM-NFQ-MGB<br>CT: 25.58	2201 A07 1000m ato1<br>U pGEM<br>1.87E4<br>FAM-NFQ-MGB<br>CT: 25.68	
E	S pGEM<br>200<br>FAM-NFQ-MGB<br>CT: 32.74	S pGEM<br>200<br>FAM-NFQ-MGB<br>CT: 32.57	2201 A04 10m saki1<br>U pGEM<br>8.72E3<br>FAM-NFQ-MGB<br>CT: 26.85	2201 A04 10m saki1<br>U pGEM<br>9.74E3<br>FAM-NFQ-MGB<br>CT: 26.68	2201 A04 10m saki1<br>U pGEM<br>9.05E3<br>FAM-NFQ-MGB<br>CT: 26.8	2201 A04 3000m saki1<br>U pGEM<br>4.96E3<br>FAM-NFQ-MGB<br>CT: 27.72	2201 A04 3000m saki1<br>U pGEM<br>5.52E3<br>FAM-NFQ-MGB<br>CT: 27.56	2201 A04 3000m saki1<br>U pGEM<br>5.11E3<br>FAM-NFQ-MGB<br>CT: 27.67	2201 A07 3000m saki1<br>U pGEM<br>1.11E4<br>FAM-NFQ-MGB<br>CT: 26.48	2201 A07 3000m saki1<br>U pGEM<br>1.07E4<br>FAM-NFQ-MGB<br>CT: 26.54	2201 A07 3000m saki1<br>U pGEM<br>1.08E4<br>FAM-NFQ-MGB<br>CT: 26.53	
F	S pGEM<br>20<br>FAM-NFQ-MGB<br>CT: 36	S pGEM<br>20<br>FAM-NFQ-MGB<br>CT: 36.65	2201 A04 10m ato1<br>U pGEM<br>1.87E4<br>FAM-NFQ-MGB<br>CT: 25.68	2201 A04 10m ato1<br>U pGEM<br>1.67E4<br>FAM-NFQ-MGB<br>CT: 25.85	2201 A04 10m ato1<br>U pGEM<br>1.83E4<br>FAM-NFQ-MGB<br>CT: 25.72	2201 A04 3000m ato1<br>U pGEM<br>1.73E4<br>FAM-NFQ-MGB<br>CT: 25.8	2201 A04 3000m ato1<br>U pGEM<br>1.93E4<br>FAM-NFQ-MGB<br>CT: 25.63	2201 A04 3000m ato1<br>U pGEM<br>1.92E4<br>FAM-NFQ-MGB<br>CT: 25.64	2201 A07 3000m ato1<br>U pGEM<br>1.94E4<br>FAM-NFQ-MGB<br>CT: 25.62	2201 A07 3000m ato1<br>U pGEM<br>1.97E4<br>FAM-NFQ-MGB<br>CT: 25.6	2201 A07 3000m ato1<br>U pGEM<br>1.81E4<br>FAM-NFQ-MGB<br>CT: 25.73	
G			2201 A04 SCM saki1<br>U pGEM<br>8.43E3<br>FAM-NFQ-MGB<br>CT: 26.91	2201 A04 SCM saki1<br>U pGEM<br>8.46E3<br>FAM-NFQ-MGB<br>CT: 26.9	2201 A04 SCM saki1<br>U pGEM<br>8.51E3<br>FAM-NFQ-MGB<br>CT: 26.89	2201 A07 SCM saki1<br>U pGEM<br>1.07E4<br>FAM-NFQ-MGB<br>CT: 26.54	2201 A07 SCM saki1<br>U pGEM<br>9.33E3<br>FAM-NFQ-MGB<br>CT: 26.75	2201 A07 SCM saki1<br>U pGEM<br>1.07E4<br>FAM-NFQ-MGB<br>CT: 26.54				N pGEM<br>FAM-NFQ-MGB<br>CT: Undetermined
H	Comf2E4<br>U pGEM<br>1.64E4<br>FAM-NFQ-MGB<br>CT: 25.88	Comf2E4<br>U pGEM<br>1.94E4<br>FAM-NFQ-MGB<br>CT: 25.63	2201 A04 SCM ato1<br>U pGEM<br>1.85E4<br>FAM-NFQ-MGB<br>CT: 25.69	2201 A04 SCM ato1<br>U pGEM<br>1.77E4<br>FAM-NFQ-MGB<br>CT: 25.77	2201 A04 SCM ato1<br>U pGEM<br>1.76E4<br>FAM-NFQ-MGB<br>CT: 25.78	2201 A07 SCM ato1<br>U pGEM<br>1.81E4<br>FAM-NFQ-MGB<br>CT: 25.73	2201 A07 SCM ato1<br>U pGEM<br>1.88E4<br>FAM-NFQ-MGB<br>CT: 25.67	2201 A07 SCM ato1<br>U pGEM<br>1.78E4<br>FAM-NFQ-MGB<br>CT: 25.76				N pGEM<br>FAM-NFQ-MGB<br>CT: Undetermined

Standard Curves

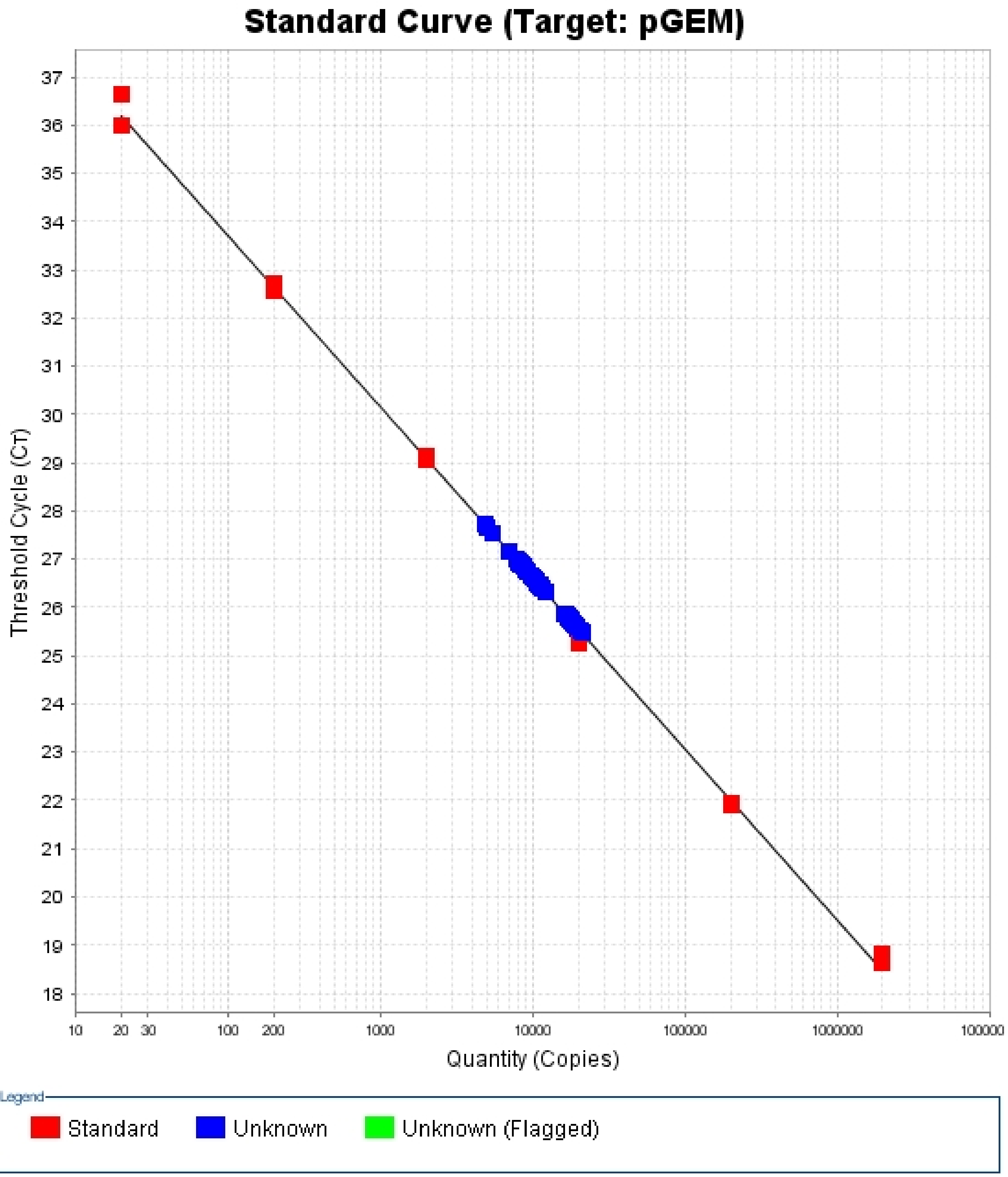

slope:-3.541 Y-Intercept:40.806 R^2:0.999 Eff%:91.608

Results Table

Well		Sample	Target	Task	Quantity	C
A1			pGEM	STANDARD	2000000	18.8156
A2			pGEM	STANDARD	2000000	18.6317
A3		2201 A01 10m	pGEM	UNKNOWN	9798.2725	26.6739
A4		2201 A01 10m	pGEM	UNKNOWN	9893.5742	26.6591
A5		2201 A01 10m	pGEM	UNKNOWN	9749.6836	26.6816
A6		2201 A04 200m	pGEM	UNKNOWN	7882.3315	27.0085
A7		2201 A04 200m	pGEM	UNKNOWN	8252.166	26.938
A8		2201 A04 200m	pGEM	UNKNOWN	8423.5615	26.9064
A9		2201 A07 200	pGEM	UNKNOWN	10179.6221	26.6152
A10		2201 A07 200	pGEM	UNKNOWN	11190.9961	26.4696
A11		2201 A07 200	pGEM	UNKNOWN	11256.7881	26.4605
B1			pGEM	STANDARD	200000	21.9531
B2			pGEM	STANDARD	200000	21.8875
B3		2201 A01 10m	pGEM	UNKNOWN	21212.3574	25.4862
B4		2201 A01 10m	pGEM	UNKNOWN	19009.9941	25.6548
B5		2201 A01 10m	pGEM	UNKNOWN	19815.625	25.5909
B6		2201 A04 200m	pGEM	UNKNOWN	20575.7734	25.533
B7		2201 A04 200m	pGEM	UNKNOWN	19188.3223	25.6404
B8		2201 A04 200m	pGEM	UNKNOWN	21230.1504	25.4849
B9		2201 A07 200	pGEM	UNKNOWN	17027.8613	25.8241
B10		2201 A07 200	pGEM	UNKNOWN	17585.9277	25.7745
B11		2201 A07 200	pGEM	UNKNOWN	18596.5586	25.6886
C1			pGEM	STANDARD	20000	25.2612
C2			pGEM	STANDARD	20000	25.4593
C3		2201 A01 SCM	pGEM	UNKNOWN	10383.0498	26.5848
C4		2201 A01 SCM	pGEM	UNKNOWN	11355.4961	26.4471
C5		2201 A01 SCM	pGEM	UNKNOWN	10465.2402	26.5727
C6		2201 A04	pGEM	UNKNOWN	7141.2456	27.1604
C7		2201 A04	pGEM	UNKNOWN	8186.5439	26.9503
C8		2201 A04	pGEM	UNKNOWN	8092.4443	26.9681

Well	Sample	Target	Task	Quantity	C
C9	2201 A07	pGEM	UNKNOWN	9908.1387	26.6568
C10	2201 A07	pGEM	UNKNOWN	12168.7725	26.3407
C11	2201 A07	pGEM	UNKNOWN	11728.1533	26.3975
D1		pGEM	STANDARD	2000	29.0509
D2		pGEM	STANDARD	2000	29.1559
D3	2201 A01 SCM	pGEM	UNKNOWN	17762.9453	25.7591
D4	2201 A01 SCM	pGEM	UNKNOWN	17387.1523	25.792
D5	2201 A01 SCM	pGEM	UNKNOWN	17477.5742	25.784
D6	2201 A04	pGEM	UNKNOWN	18079.2617	25.7319
D7	2201 A04	pGEM	UNKNOWN	19967.2305	25.5792
D8	2201 A04	pGEM	UNKNOWN	18851.293	25.6676
D9	2201 A07	pGEM	UNKNOWN	19103.0723	25.6472
D10	2201 A07	pGEM	UNKNOWN	19952.4512	25.5803
D11	2201 A07	pGEM	UNKNOWN	18735.9551	25.6771
E1		pGEM	STANDARD	200	32.7402
E2		pGEM	STANDARD	200	32.5688
E3	2201 A04 10m	pGEM	UNKNOWN	8720.4834	26.8531
E4	2201 A04 10m	pGEM	UNKNOWN	9737.1396	26.6836
E5	2201 A04 10m	pGEM	UNKNOWN	9052.3154	26.7957
E6	2201 A04	pGEM	UNKNOWN	4956.605	27.7219
E7	2201 A04	pGEM	UNKNOWN	5519.6167	27.5565
E8	2201 A04	pGEM	UNKNOWN	5113.6641	27.674
E9	2201 A07	pGEM	UNKNOWN	11134.3691	26.4774
E10	2201 A07	pGEM	UNKNOWN	10657.9189	26.5446
E11	2201 A07	pGEM	UNKNOWN	10765.7207	26.5291
F1		pGEM	STANDARD	20	35.9961
F2		pGEM	STANDARD	20	36.6457
F3	2201 A04 10m	pGEM	UNKNOWN	18744.998	25.6763
F4	2201 A04 10m	pGEM	UNKNOWN	16749.9355	25.8494
F5	2201 A04 10m	pGEM	UNKNOWN	18274.6816	25.7154
F6	2201 A04	pGEM	UNKNOWN	17320.3633	25.7979
F7	2201 A04	pGEM	UNKNOWN	19342.3496	25.6281

Well		Sample	Target	Task	Quantity	C
F8		2201 A04	pGEM	UNKNOWN	19157.0762	25.6429
F9		2201 A07	pGEM	UNKNOWN	19435.5137	25.6207
F10		2201 A07	pGEM	UNKNOWN	19663.3633	25.6028
F11		2201 A07	pGEM	UNKNOWN	18075.2266	25.7323
G3		2201 A04 SCM	pGEM	UNKNOWN	8429.6338	26.9053
G4		2201 A04 SCM	pGEM	UNKNOWN	8456.5264	26.9004
G5		2201 A04 SCM	pGEM	UNKNOWN	8512.5215	26.8903
G6		2201 A07 SCM	pGEM	UNKNOWN	10675.8721	26.542
G7		2201 A07 SCM	pGEM	UNKNOWN	9328.585	26.7495
G8		2201 A07 SCM	pGEM	UNKNOWN	10677.9912	26.5417
G12			pGEM	NTC		Undetermined
H1		Comf2E4	pGEM	UNKNOWN	16392.543	25.8826
H2		Comf2E4	pGEM	UNKNOWN	19370.7285	25.6258
H3		2201 A04 SCM	pGEM	UNKNOWN	18531.9492	25.6939
H4		2201 A04 SCM	pGEM	UNKNOWN	17670.2598	25.7671
H5		2201 A04 SCM	pGEM	UNKNOWN	17558.9453	25.7769
H6		2201 A07 SCM	pGEM	UNKNOWN	18144.8359	25.7264
H7		2201 A07 SCM	pGEM	UNKNOWN	18787.9199	25.6728
H8		2201 A07 SCM	pGEM	UNKNOWN	17784.0215	25.7573
H12			pGEM	NTC		Undetermined

QC Summary

Total Wells	96	Processed Wells	82	Targets Used	1
Well Setup	82	Flagged Wells	0	Samples Used	23

Flag	Name	Frequency	Locations
AMPNC	Amplification in negative control	0	
BADROX	Bad passive reference signal	0	
BLFAIL	Baseline algorithm failed	0	
CTFAIL	C algorithm failed	0	
EXPFAIL	Exponential algorithm failed	0	
HIGHSD	High standard deviation in replicate group	0	
NOAMP	No amplification	0	
NOISE	Noise higher than others in plate	0	
NOSIGNAL	No signal in well	0	
OFFSCALE	Fluorescence is offscale	0	
OUTLIERRG	Outlier in replicate group	0	
SPIKE	Noise spikes	0	
THOLDFAIL	Thresholding algorithm failed	0	
