# Supplementary material for: Ecological impact of algal-preying protists aplanochytrids (Labyrinthulea) as a source of docosahexaenoic acid for copepods in marine environments
Source: ISME Commun. 2026 Jun 26;6(1):ycag178. doi: 10.1093/ismeco/ycag178 (PMC13685785; doi:10.1093/ismeco/ycag178)
Supplement: Supplementary file 2 [file supplementary_materials_ycag178.zip › Morimoto_ISME_C_3rdrev_supplemental_data.zip!/Morimoto_ISME_C_3rdrev_supplemental_data/Supplemental_Data_2/Standard Curve/DNA recovery/2201/DnaRec2201AlineA01-A07N=2.pdf]

# Experiment Results Report

## 230627DnaRec2201AlineA01-A07N=2

## Experiment Summary

| | | |
|---|---|---|
| Experiment Name | : | 230627DnaRec2201AlineA01-A07N=2 |
| Experiment Type | : | Quantitation - Standard Curve |
| File Name | : | 230627DnaRec2201AlineA01-A07N=2.eds |
| Run Started | : | 2023 Jun 27 3:05:55 PM |
| Run Finished | : | 2023 Jun 27 4:40:34 PM |
| Run Duration | : | 94 minutes 39 seconds |
| Date Modified | : | 2023 Jun 27 5:05:13 PM |
| User | : | |
| Number of wells used | : | 82 |
| Number of wells with results | : | 80 |
| Instrument Name | : | |
| Instrument Type | : | Applied Biosystems StepOnePlus™ Instrument |
| Comments | : | |

# Results Summary

| Sample | Target | Quantity (Mean) | Quantity (Std Dev) | C (Mean) | C (Std Dev) |
|---|---|---|---|---|---|
| 2201 A01 10m ato1 | pGEM | 21431.16 | 817.92 | 25.93 | 0.06 |
| 2201 A01 10m saki1 | pGEM | 12906.30 | 788.85 | 26.70 | 0.09 |
| 2201 A01 SCM ato1 | pGEM | 19025.21 | 792.58 | 26.11 | 0.06 |
| 2201 A01 SCM saki1 | pGEM | 14781.22 | 808.09 | 26.50 | 0.08 |
| 2201 A04 1000m | pGEM | 21977.14 | 2563.13 | 25.89 | 0.18 |
| 2201 A04 1000m | pGEM | 17676.59 | 1304.18 | 26.22 | 0.11 |
| 2201 A04 10m ato1 | pGEM | 27031.54 | 2409.70 | 25.57 | 0.14 |
| 2201 A04 10m saki1 | pGEM | 14384.17 | 95.22 | 26.54 | 0.01 |
| 2201 A04 200m ato1 | pGEM | 20567.68 | 373.35 | 25.99 | 0.03 |
| 2201 A04 200m | pGEM | 13455.99 | 572.97 | 26.64 | 0.06 |
| 2201 A04 3000m | pGEM | 19120.04 | 879.88 | 26.10 | 0.07 |
| 2201 A04 3000m | pGEM | 1294.06 | 69.60 | 30.23 | 0.08 |
| 2201 A04 SCM ato1 | pGEM | 20955.21 | 224.87 | 25.96 | 0.02 |
| 2201 A04 SCM saki1 | pGEM | 15305.68 | 149.58 | 26.44 | 0.01 |
| 2201 A07 1000m | pGEM | 21901.23 | 1777.79 | 25.90 | 0.13 |
| 2201 A07 1000m | pGEM | 13764.57 | 1266.27 | 26.61 | 0.15 |
| 2201 A07 200 ato1 | pGEM | 19953.66 | 275.70 | 26.04 | 0.02 |
| 2201 A07 200 saki1 | pGEM | 12301.79 | 88.58 | 26.78 | 0.01 |
| 2201 A07 3000m | pGEM | 26265.37 | 549.85 | 25.61 | 0.03 |
| 2201 A07 3000m | pGEM | 13514.99 | 997.09 | 26.63 | 0.11 |
| 2201 A07 SCM ato1 | pGEM | 20639.22 | 228.50 | 25.98 | 0.02 |
| 2201 A07 SCM saki1 | pGEM | 14321.75 | 792.22 | 26.54 | 0.09 |

# Plate Layout

| | 1 | 2 | 3 | 4 | 5 | 6 | 7 | 8 | 9 | 10 | 11 | 12 |
|---|---|---|---|---|---|---|---|---|---|---|---|---|
| A | S pGEM<br>2E6<br>FAM-NFQ-MGB<br>CT: 19.1 | S pGEM<br>2E6<br>FAM-NFQ-MGB<br>CT: 18.94 | 2201 A01 10m saki1<br>U pGEM<br>1.38E4<br>FAM-NFQ-MGB<br>CT: 26.6 | 2201 A01 10m saki1<br>U pGEM<br>1.26E4<br>FAM-NFQ-MGB<br>CT: 26.74 | 2201 A01 10m saki1<br>U pGEM<br>1.23E4<br>FAM-NFQ-MGB<br>CT: 26.78 | 2201 A04 200m saki1<br>U pGEM<br>1.29E4<br>FAM-NFQ-MGB<br>CT: 26.7 | 2201 A04 200m saki1<br>U pGEM<br>1.41E4<br>FAM-NFQ-MGB<br>CT: 26.57 | 2201 A04 200m saki1<br>U pGEM<br>1.33E4<br>FAM-NFQ-MGB<br>CT: 26.65 | 2201 A07 200 saki1<br>U pGEM<br>1.24E4<br>FAM-NFQ-MGB<br>CT: 26.77 | 2201 A07 200 saki1<br>U pGEM<br>1.24E4<br>FAM-NFQ-MGB<br>CT: 26.77 | 2201 A07 200 saki1<br>U pGEM<br>1.22E4<br>FAM-NFQ-MGB<br>CT: 26.79 | |
| B | S pGEM<br>2E5<br>FAM-NFQ-MGB<br>CT: 22.31 | S pGEM<br>2E5<br>FAM-NFQ-MGB<br>CT: 22.24 | 2201 A01 10m ato1<br>U pGEM<br>2.16E4<br>FAM-NFQ-MGB<br>CT: 25.92 | 2201 A01 10m ato1<br>U pGEM<br>2.22E4<br>FAM-NFQ-MGB<br>CT: 25.87 | 2201 A01 10m ato1<br>U pGEM<br>2.06E4<br>FAM-NFQ-MGB<br>CT: 25.99 | 2201 A04 200m ato1<br>U pGEM<br>2.06E4<br>FAM-NFQ-MGB<br>CT: 25.99 | 2201 A04 200m ato1<br>U pGEM<br>2.02E4<br>FAM-NFQ-MGB<br>CT: 26.02 | 2201 A04 200m ato1<br>U pGEM<br>2.09E4<br>FAM-NFQ-MGB<br>CT: 25.96 | 2201 A07 200 ato1<br>U pGEM<br>1.99E4<br>FAM-NFQ-MGB<br>CT: 26.04 | 2201 A07 200 ato1<br>U pGEM<br>2.03E4<br>FAM-NFQ-MGB<br>CT: 26.01 | 2201 A07 200 ato1<br>U pGEM<br>1.97E4<br>FAM-NFQ-MGB<br>CT: 26.05 | |
| C | S pGEM<br>2E4<br>FAM-NFQ-MGB<br>CT: 26.17 | S pGEM<br>2E4<br>FAM-NFQ-MGB<br>CT: 25.9 | 2201 A01 SCM saki1<br>U pGEM<br>1.47E4<br>FAM-NFQ-MGB<br>CT: 26.5 | 2201 A01 SCM saki1<br>U pGEM<br>1.4E4<br>FAM-NFQ-MGB<br>CT: 26.58 | 2201 A01 SCM saki1<br>U pGEM<br>1.56E4<br>FAM-NFQ-MGB<br>CT: 26.41 | 2201 A04 1000m saki1<br>U pGEM<br>1.69E4<br>FAM-NFQ-MGB<br>CT: 26.29 | 2201 A04 1000m saki1<br>U pGEM<br>1.69E4<br>FAM-NFQ-MGB<br>CT: 26.29 | 2201 A04 1000m saki1<br>U pGEM<br>1.92E4<br>FAM-NFQ-MGB<br>CT: 26.1 | 2201 A07 1000m saki1<br>U pGEM<br>1.23E4<br>FAM-NFQ-MGB<br>CT: 26.78 | 2201 A07 1000m saki1<br>U pGEM<br>1.45E4<br>FAM-NFQ-MGB<br>CT: 26.52 | 2201 A07 1000m saki1<br>U pGEM<br>1.45E4<br>FAM-NFQ-MGB<br>CT: 26.53 | |
| D | S pGEM<br>2E3<br>FAM-NFQ-MGB<br>CT: 29.79 | S pGEM<br>2E3<br>FAM-NFQ-MGB<br>CT: 29.99 | 2201 A01 SCM ato1<br>U pGEM<br>1.98E4<br>FAM-NFQ-MGB<br>CT: 26.04 | 2201 A01 SCM ato1<br>U pGEM<br>1.9E4<br>FAM-NFQ-MGB<br>CT: 26.11 | 2201 A01 SCM ato1<br>U pGEM<br>1.83E4<br>FAM-NFQ-MGB<br>CT: 26.17 | 2201 A04 1000m ato1<br>U pGEM<br>1.97E4<br>FAM-NFQ-MGB<br>CT: 26.06 | 2201 A04 1000m ato1<br>U pGEM<br>2.47E4<br>FAM-NFQ-MGB<br>CT: 25.71 | 2201 A04 1000m ato1<br>U pGEM<br>2.15E4<br>FAM-NFQ-MGB<br>CT: 25.92 | 2201 A07 1000m ato1<br>U pGEM<br>1.99E4<br>FAM-NFQ-MGB<br>CT: 26.04 | 2201 A07 1000m ato1<br>U pGEM<br>2.32E4<br>FAM-NFQ-MGB<br>CT: 25.81 | 2201 A07 1000m ato1<br>U pGEM<br>2.27E4<br>FAM-NFQ-MGB<br>CT: 25.84 | |
| E | S pGEM<br>200<br>FAM-NFQ-MGB<br>CT: 33.24 | S pGEM<br>200<br>FAM-NFQ-MGB<br>CT: 32.97 | 2201 A04 10m saki1<br>U pGEM<br>1.44E4<br>FAM-NFQ-MGB<br>CT: 26.53 | 2201 A04 10m saki1<br>U pGEM<br>1.45E4<br>FAM-NFQ-MGB<br>CT: 26.53 | 2201 A04 10m saki1<br>U pGEM<br>1.43E4<br>FAM-NFQ-MGB<br>CT: 26.55 | 2201 A04 3000m saki1<br>U pGEM<br>1.21E3<br>FAM-NFQ-MGB<br>CT: 30.32 | 2201 A04 3000m saki1<br>U pGEM<br>1.32E3<br>FAM-NFQ-MGB<br>CT: 30.19 | 2201 A04 3000m saki1<br>U pGEM<br>1.34E3<br>FAM-NFQ-MGB<br>CT: 30.17 | 2201 A07 3000m saki1<br>U pGEM<br>1.25E4<br>FAM-NFQ-MGB<br>CT: 26.76 | 2201 A07 3000m saki1<br>U pGEM<br>1.36E4<br>FAM-NFQ-MGB<br>CT: 26.62 | 2201 A07 3000m saki1<br>U pGEM<br>1.45E4<br>FAM-NFQ-MGB<br>CT: 26.53 | |
| F | ⚠ 1<br>S pGEM<br>20<br>FAM-NFQ-MGB<br>CT: 35.95 | ⚠ 1<br>S pGEM<br>20<br>FAM-NFQ-MGB<br>CT: 36.95 | 2201 A04 10m ato1<br>U pGEM<br>2.74E4<br>FAM-NFQ-MGB<br>CT: 25.55 | 2201 A04 10m ato1<br>U pGEM<br>2.45E4<br>FAM-NFQ-MGB<br>CT: 25.72 | 2201 A04 10m ato1<br>U pGEM<br>2.93E4<br>FAM-NFQ-MGB<br>CT: 25.45 | 2201 A04 3000m ato1<br>U pGEM<br>1.81E4<br>FAM-NFQ-MGB<br>CT: 26.18 | 2201 A04 3000m ato1<br>U pGEM<br>1.98E4<br>FAM-NFQ-MGB<br>CT: 26.05 | 2201 A04 3000m ato1<br>U pGEM<br>1.95E4<br>FAM-NFQ-MGB<br>CT: 26.07 | 2201 A07 3000m ato1<br>U pGEM<br>2.57E4<br>FAM-NFQ-MGB<br>CT: 25.65 | 2201 A07 3000m ato1<br>U pGEM<br>2.63E4<br>FAM-NFQ-MGB<br>CT: 25.61 | 2201 A07 3000m ato1<br>U pGEM<br>2.68E4<br>FAM-NFQ-MGB<br>CT: 25.58 | |
| G | | | 2201 A04 SCM saki1<br>U pGEM<br>1.53E4<br>FAM-NFQ-MGB<br>CT: 26.45 | 2201 A04 SCM saki1<br>U pGEM<br>1.55E4<br>FAM-NFQ-MGB<br>CT: 26.42 | 2201 A04 SCM saki1<br>U pGEM<br>1.52E4<br>FAM-NFQ-MGB<br>CT: 26.45 | 2201 A07 SCM saki1<br>U pGEM<br>1.49E4<br>FAM-NFQ-MGB<br>CT: 26.49 | 2201 A07 SCM saki1<br>U pGEM<br>1.34E4<br>FAM-NFQ-MGB<br>CT: 26.64 | 2201 A07 SCM saki1<br>U pGEM<br>1.47E4<br>FAM-NFQ-MGB<br>CT: 26.51 | | | | N pGEM<br>FAM-NFQ-MGB<br>CT: Undetermined |
| H | Comf2E4 | Comf2E4 | 2201 A04 SCM ato1<br>U pGEM<br>2.08E4<br>FAM-NFQ-MGB<br>CT: 25.97 | 2201 A04 SCM ato1<br>U pGEM<br>2.12E4<br>FAM-NFQ-MGB<br>CT: 25.94 | 2201 A04 SCM ato1<br>U pGEM<br>2.08E4<br>FAM-NFQ-MGB<br>CT: 25.97 | 2201 A07 SCM ato1<br>U pGEM<br>2.05E4<br>FAM-NFQ-MGB<br>CT: 26 | 2201 A07 SCM ato1<br>U pGEM<br>2.05E4<br>FAM-NFQ-MGB<br>CT: 25.99 | 2201 A07 SCM ato1<br>U pGEM<br>2.09E4<br>FAM-NFQ-MGB<br>CT: 25.96 | | | | N pGEM<br>FAM-NFQ-MGB<br>CT: Undetermined |

# Standard Curves

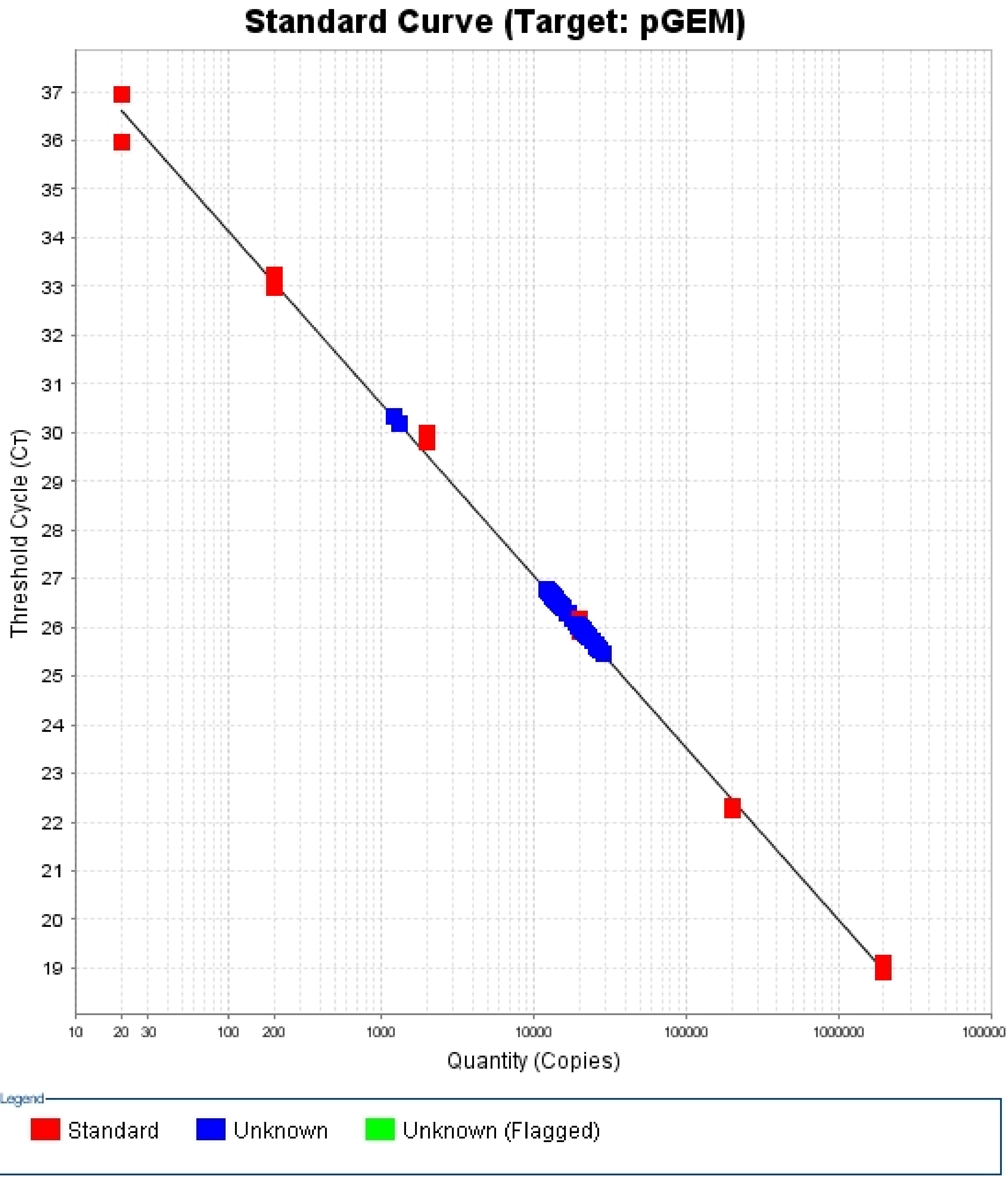


slope:-3.528 Y-Intercept:41.206 $R^2$:0.998 Eff%:92.057

# Results Table

| Well | | Sample | Target | Task | Quantity | C |
|---|---|---|---|---|---|---|
| A1 | | | pGEM | STANDARD | 2000000 | 19.0956 |
| A2 | | | pGEM | STANDARD | 2000000 | 18.9415 |
| A3 | | 2201 A01 10m | pGEM | UNKNOWN | 13797.5098 | 26.6004 |
| A4 | | 2201 A01 10m | pGEM | UNKNOWN | 12623.7627 | 26.7366 |
| A5 | | 2201 A01 10m | pGEM | UNKNOWN | 12297.6201 | 26.7767 |
| A6 | | 2201 A04 200m | pGEM | UNKNOWN | 12947.2842 | 26.6978 |
| A7 | | 2201 A04 200m | pGEM | UNKNOWN | 14076.6914 | 26.5697 |
| A8 | | 2201 A04 200m | pGEM | UNKNOWN | 13343.9854 | 26.6516 |
| A9 | | 2201 A07 200 | pGEM | UNKNOWN | 12350.7451 | 26.7701 |
| A10 | | 2201 A07 200 | pGEM | UNKNOWN | 12355.0811 | 26.7696 |
| A11 | | 2201 A07 200 | pGEM | UNKNOWN | 12199.5381 | 26.789 |
| B1 | | | pGEM | STANDARD | 200000 | 22.3095 |
| B2 | | | pGEM | STANDARD | 200000 | 22.2385 |
| B3 | | 2201 A01 10m | pGEM | UNKNOWN | 21567.4414 | 25.9159 |
| B4 | | 2201 A01 10m | pGEM | UNKNOWN | 22172.3848 | 25.8735 |
| B5 | | 2201 A01 10m | pGEM | UNKNOWN | 20553.6641 | 25.9897 |
| B6 | | 2201 A04 200m | pGEM | UNKNOWN | 20554.8672 | 25.9896 |
| B7 | | 2201 A04 200m | pGEM | UNKNOWN | 20200.8945 | 26.0162 |
| B8 | | 2201 A04 200m | pGEM | UNKNOWN | 20947.2734 | 25.9606 |
| B9 | | 2201 A07 200 | pGEM | UNKNOWN | 19889.4277 | 26.04 |
| B10 | | 2201 A07 200 | pGEM | UNKNOWN | 20255.8105 | 26.012 |
| B11 | | 2201 A07 200 | pGEM | UNKNOWN | 19715.7441 | 26.0534 |
| C1 | | | pGEM | STANDARD | 20000 | 26.1744 |
| C2 | | | pGEM | STANDARD | 20000 | 25.9006 |
| C3 | | 2201 A01 SCM | pGEM | UNKNOWN | 14710.1582 | 26.5022 |
| C4 | | 2201 A01 SCM | pGEM | UNKNOWN | 14011.0137 | 26.5768 |
| C5 | | 2201 A01 SCM | pGEM | UNKNOWN | 15622.5 | 26.41 |
| C6 | | 2201 A04 | pGEM | UNKNOWN | 16904.1484 | 26.2892 |
| C7 | | 2201 A04 | pGEM | UNKNOWN | 16943.2676 | 26.2857 |
| C8 | | 2201 A04 | pGEM | UNKNOWN | 19182.3633 | 26.0955 |

| Well | Sample | Target | Task | Quantity | C |
|---|---|---|---|---|---|
| C9 | 2201 A07 | pGEM | UNKNOWN | 12302.5342 | 26.7761 |
| C10 | 2201 A07 | pGEM | UNKNOWN | 14512.1514 | 26.523 |
| C11 | 2201 A07 | pGEM | UNKNOWN | 14479.0234 | 26.5265 |
| D1 | | pGEM | STANDARD | 2000 | 29.7914 |
| D2 | | pGEM | STANDARD | 2000 | 29.994 |
| D3 | 2201 A01 SCM | pGEM | UNKNOWN | 19836.2676 | 26.0441 |
| D4 | 2201 A01 SCM | pGEM | UNKNOWN | 18986.8535 | 26.1112 |
| D5 | 2201 A01 SCM | pGEM | UNKNOWN | 18252.502 | 26.1716 |
| D6 | 2201 A04 | pGEM | UNKNOWN | 19659.0117 | 26.0579 |
| D7 | 2201 A04 | pGEM | UNKNOWN | 24729.7285 | 25.7062 |
| D8 | 2201 A04 | pGEM | UNKNOWN | 21542.6758 | 25.9177 |
| D9 | 2201 A07 | pGEM | UNKNOWN | 19869.1348 | 26.0416 |
| D10 | 2201 A07 | pGEM | UNKNOWN | 23169.2715 | 25.8061 |
| D11 | 2201 A07 | pGEM | UNKNOWN | 22665.2773 | 25.8398 |
| E1 | | pGEM | STANDARD | 200 | 33.2356 |
| E2 | | pGEM | STANDARD | 200 | 32.9671 |
| E3 | 2201 A04 10m | pGEM | UNKNOWN | 14404.7969 | 26.5344 |
| E4 | 2201 A04 10m | pGEM | UNKNOWN | 14467.3848 | 26.5277 |
| E5 | 2201 A04 10m | pGEM | UNKNOWN | 14280.3242 | 26.5477 |
| E6 | 2201 A04 | pGEM | UNKNOWN | 1214.679 | 30.3238 |
| E7 | 2201 A04 | pGEM | UNKNOWN | 1322.881 | 30.1931 |
| E8 | 2201 A04 | pGEM | UNKNOWN | 1344.6078 | 30.1681 |
| E9 | 2201 A07 | pGEM | UNKNOWN | 12471.1064 | 26.7552 |
| E10 | 2201 A07 | pGEM | UNKNOWN | 13616.3125 | 26.6206 |
| E11 | 2201 A07 | pGEM | UNKNOWN | 14457.5557 | 26.5288 |
| F1 | | pGEM | STANDARD | 20 | 35.9511 |
| F2 | | pGEM | STANDARD | 20 | 36.9464 |
| F3 | 2201 A04 10m | pGEM | UNKNOWN | 27370.9043 | 25.5508 |
| F4 | 2201 A04 10m | pGEM | UNKNOWN | 24470.1523 | 25.7224 |
| F5 | 2201 A04 10m | pGEM | UNKNOWN | 29253.5684 | 25.4488 |
| F6 | 2201 A04 | pGEM | UNKNOWN | 18119.1914 | 26.1828 |
| F7 | 2201 A04 | pGEM | UNKNOWN | 19771.8535 | 26.0491 |

| Well | | Sample | Target | Task | Quantity | C |
|---|---|---|---|---|---|---|
| F8 | | 2201 A04 | pGEM | UNKNOWN | 19469.0605 | 26.0727 |
| F9 | | 2201 A07 | pGEM | UNKNOWN | 25722.498 | 25.6459 |
| F10 | | 2201 A07 | pGEM | UNKNOWN | 26251.6641 | 25.6147 |
| F11 | | 2201 A07 | pGEM | UNKNOWN | 26821.9473 | 25.5818 |
| G3 | | 2201 A04 SCM | pGEM | UNKNOWN | 15252.375 | 26.4467 |
| G4 | | 2201 A04 SCM | pGEM | UNKNOWN | 15474.6133 | 26.4246 |
| G5 | | 2201 A04 SCM | pGEM | UNKNOWN | 15190.0576 | 26.453 |
| G6 | | 2201 A07 SCM | pGEM | UNKNOWN | 14874.0684 | 26.4852 |
| G7 | | 2201 A07 SCM | pGEM | UNKNOWN | 13414.0654 | 26.6435 |
| G8 | | 2201 A07 SCM | pGEM | UNKNOWN | 14677.1074 | 26.5057 |
| G12 | | | pGEM | NTC | | Undetermined |
| H3 | | 2201 A04 SCM | pGEM | UNKNOWN | 20813.2617 | 25.9704 |
| H4 | | 2201 A04 SCM | pGEM | UNKNOWN | 21214.4824 | 25.9412 |
| H5 | | 2201 A04 SCM | pGEM | UNKNOWN | 20837.8887 | 25.9686 |
| H6 | | 2201 A07 SCM | pGEM | UNKNOWN | 20481.2598 | 25.9951 |
| H7 | | 2201 A07 SCM | pGEM | UNKNOWN | 20535.1758 | 25.991 |
| H8 | | 2201 A07 SCM | pGEM | UNKNOWN | 20901.2227 | 25.964 |
| H12 | | | pGEM | NTC | | Undetermined |

# QC Summary

| | | | | | |
|---|---|---|---|---|---|
| Total Wells | 96 | Processed Wells | 82 | Targets Used | 1 |
| Well Setup | 82 | Flagged Wells | 2 | Samples Used | 23 |

| Flag | Name | Frequency | Locations |
|---|---|---|---|
| AMPNC | Amplification in negative control | 0 | |
| BADROX | Bad passive reference signal | 0 | |
| BLFAIL | Baseline algorithm failed | 0 | |
| CTFAIL | C algorithm failed | 0 | |
| EXPFAIL | Exponential algorithm failed | 0 | |
| HIGHSD | High standard deviation in replicate group | 2 | F1, F2 |
| NOAMP | No amplification | 0 | |
| NOISE | Noise higher than others in plate | 0 | |
| NOSIGNAL | No signal in well | 0 | |
| OFFSCALE | Fluorescence is offscale | 0 | |
| OUTLIERRG | Outlier in replicate group | 0 | |
| SPIKE | Noise spikes | 0 | |
| THOLDFAIL | Thresholding algorithm failed | 0 | |
